# Hydrogen radical-shuttle (HRS)-enabled photoredox synthesis of indanones via decarboxylative annulation

**DOI:** 10.1038/s41467-021-25594-4

**Published:** 2021-09-06

**Authors:** Bo Yang, Shi-Jun Li, Yongdong Wang, Yu Lan, Shifa Zhu

**Affiliations:** 1 Key Laboratory of Functional Molecular Engineering of Guangdong Province, School of Chemistry and Chemical Engineering, South China University of Technology, Guangzhou, China; 2 Singfar Laboratories, Guangzhou, China; 3 Green Catalysis Center, and College of Chemistry, Zhengzhou University, Zhengzhou, Henan China; 4 School of Chemistry and Chemical Engineering, and Chongqing Key Laboratory of Theoretical and Computational Chemistry, Chongqing University, Chongqing, China

**Keywords:** Photocatalysis, Synthetic chemistry methodology

## Abstract

Hydrogen atom transfer (HAT) process is a powerful and effective strategy for activating C-H bonds followed by further functionalization. Intramolecular 1,n (n = 5 or 6)-HATs are common and frequently encountered in organic synthesis. However, intramolecular 1,n (n = 2 or 3)-HAT is very challenging due to slow kinetics. Compared to proton-shuttle process, which is well established for organic synthesis, hydrogen radical-shuttle (HRS) is unexplored. In this work, a HRS-enabled decarboxylative annulation of carbonyl compounds via photoredox catalysis for the synthesis of indanones is developed. This protocol features broad substrate scope, excellent functional group tolerance, internal hydrogen radical transfer, atom- and step-economy. Critical to the success of this process is the introduction of water, acting as both HRS and hydrogen source, which was demonstrated by mechanistic experiments and density functional theory (DFT) calculations. Importantly, this mechanistically distinctive HAT provides a complement to that of typical proton-shuttle-promoted, representing a breakthrough in hydrogen radical transfer, especially in the inherently challenging 1,2- or 1,3-HAT.

## Abstract (web summary)

Although hydrogen atom transfer is widely observed in synthetic organic chemistry, intramolecular hydrogen atom transfer between atoms separated by fewer than four bonds is kinetically slow. Here the authors show a method to form indanones, with hydrogen atoms shuttled across short distances by water.

## Introduction

**Fig. 1:**
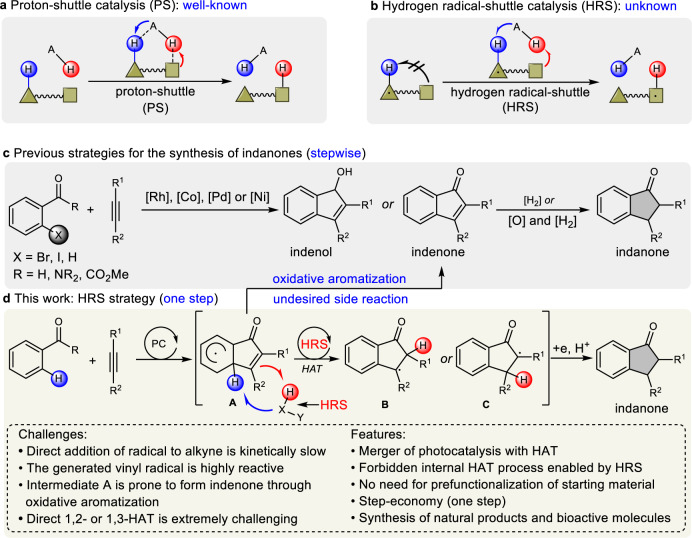
Proton/hydrogen radical-shuttle catalysis and indanone scaffold synthetic strategy. **a** Proton-shuttle catalysis model. **b** Hydrogen radical-shuttle catalysis model. **c** Traditional synthetic strategies for indanones by annulation reaction of alkynes. **d** HRS-enabled strategy for indanone synthesis (this work). PC photocatalyst.

As a powerful and effective strategy, hydrogen atom transfer (HAT) catalysis has been demonstrated as an ideal platform for C–H bonds functionalizations, majorly involving proton shift and hydrogen radical transfer^1–12^. When it comes to proton transfer, proton-shuttle (PS) catalysis has been well developed for the past decades, providing a highly efficient strategy for C–H functionalization, especially in transition-metal-catalyzed C–H activation^13–15^ and insertion of carbenes into heteroatom–hydrogen bonds^16–24^ (Fig. 1a). It has been recognized that PS catalysts, such as water^16^, acid^13, 17–23^ and alcohol^24^, could lower the reaction barrier by forming cyclic molecular complexes that involve lower ring strain and facilitate intra- or intermolecular HAT. In the latter process (hydrogen radical transfer), a reactive radical species, traditionally, was needed to abstract hydrogen from C–H bond to generate the corresponding carbon-centered radical intermediate^6–8, 11, 25–28^, triggering the following functionalization process. Based on the great achievements in PS catalysis, we wondered whether a similar hydrogen radical-shuttle (HRS) strategy could be used to complete the HAT process (Fig. 1b). Notably, the core difference between HRS-promoted HAT and that of polarity-reversal-catalyzed^29, 30^ is that hydrogen radical transfer occurs from a neutral position to another non-radical site. To the best of our knowledge, no successful examples utilizing this strategy have been reported. Importantly, this would be another complementary process to that of PS catalysis. With this HRS strategy in mind, we engaged to develop practical approaches for important scaffolds synthesis via a radical pathway.

Considering the prevalence of indanones and their derivatives in pharmaceuticals and biologically active natural products^31–35^, a lot of efforts have been devoted to developing effective strategies for indanones synthesis^36–40^. Traditionally, indanones were prepared from the corresponding indenols or indenones. Among a variety of approaches, transition-metal catalyzed annulation of *ortho*-halogenated carbonyl compounds and alkynes is one of the highly efficient and general strategies to construct indenone scaffolds^41–45^. For example, Yamamoto^46^ and Cheng^47, 48^ reported the cyclization of *ortho*-halogenated carbonyl compounds and alkynes to construct indenols, respectively. Kong^49^ reported the indanones synthesis based on hydrogen auto-transfer strategy through nickel catalysis. Notwithstanding great achievements that have been made, these methods typically suffered from the prefunctionalization of the corresponding starting materials. Direct C–H bond functionalization to access indenones through Rh-catalyzed procedures has also been developed^50–52^. However, among these traditional strategies, a stepwise process has to be adopted because additional oxidation and/or reduction processes are often required when converting the indenols or indenones to indanones (Fig. 1c). Therefore, developing a direct C–H annulation of carbonyl compounds with alkynes for indanones synthesis in one step is highly appealing and desirable.

Recently, aryl C*sp*^2^–H functionalization involving a radical process has emerged as an ideal and powerful strategy to construct C–C bonds, along with diminished cost and waste^53, 54^. These methods rely on certain carbon radicals trapped by arenes and followed by the aromatization process, which might provide an alternative protocol for the direct annulation of carbonyl compounds to construct indanones. In addition, acyl radicals produced efficiently from α-oxocarboxylic acid, aldehyde, acyl halide, and so on via radical pathway^55^, have been well researched with alkenes. Inspired by these developments, we anticipated that if we could utilize the electron-deficient vinyl radicals, generated from acyl radical addition to alkynes, to achieve direct construction of indanones through the dearomatic radical intermediate **A**. However, the typical oxidative aromatization strategy from intermediate **A** to the desired indanone product is often problematic due to (i) the electron-deficient indenone is readily prone to [2 + 2] cycloaddition under photoredox conditions^56^ and (ii) the Giese-type reaction of acyl radical with indenone would be the main side reaction^57^. Moreover, the following external reduction steps were also required from indenone to indanone. To overcome these obstacles, we questioned if it is possible to merge HAT with single electron transfer (SET), resulting in the generation of intermediate **B** or **C**. We recognized that such a merger might realize rearomatization of intermediate **A** and avoid the generation of indenone, providing an aromatization model and an ideal strategy for the direct construction of indanones without additional prefunctionalization of substrates and external steps. However, the direct addition of radicals to unactivated alkynes is kinetically slow^58, 59^ and the generation of corresponding high-energy vinyl radical intermediate is highly reactive, which can participate in various undesirable open-shell pathways. In addition, intermediate **A** is prone to form indenone through oxidation/elimination steps. More importantly, as the critical problem in our design, the HAT (1,2- or 1,3-HAT) strategy forming **B** or **C** is challenging^11, 60, 61^ due to the high activation energy, which could be attributed to the increased C–H–C/heteroatom strain. According to the analysis above, an HRS-enabled HAT strategy, we speculated, might be an ideal protocol to circumvent this problem (Fig. 1d). A suitable HRS catalyst was required to modulate the reactivity of intermediate **A**, thereby providing an opportunity for rearomatization and hydrogen radical transfer of **A** simultaneously, furnishing the effective synthesis of indanones.

In this work, we report an HRS-enabled decarboxylative annulation of carbonyl compounds for the synthesis of indanones via photocatalysis with excellent functional group tolerance, broad substrate scope as well as an atom- and step-economy. The key to the success of this protocol is the application of water molecules, functioning as both solvent and HRS and promoting the hydrogen radical transfer in formal 1,3-HAT process, which was demonstrated by mechanistic experiments and DFT calculations.

## Results and discussion

### Reaction development

**Fig. 2:**
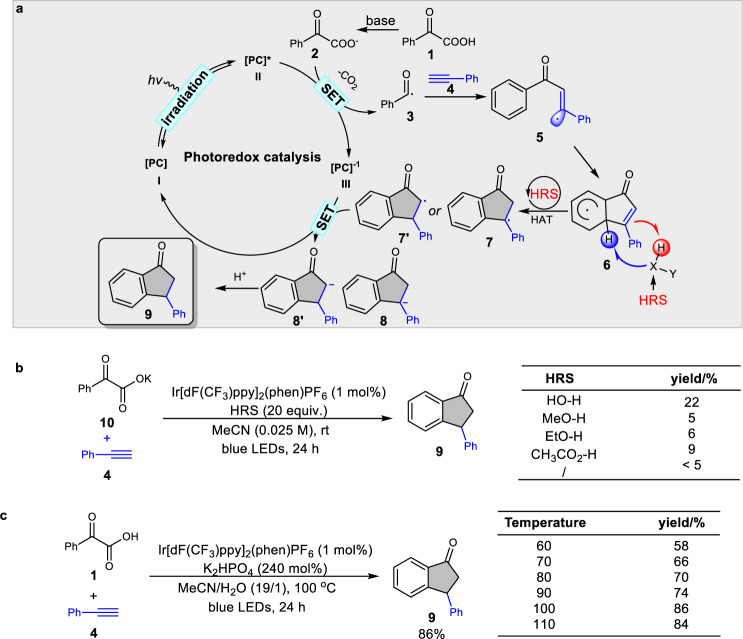
Reaction development. **a** Proposed mechanism for synthesis of indanone. **b** Evaluation of possible HRS catalyst. **c** Investigation of temperature’s effect on the reaction. PC photocatalyst, HRS hydrogen radical-shuttle.

From a design perspective, with benzoylformic acid **1** as acyl radical precursor, we envisioned that this HRS-promoted HAT/SET strategy could be outlined as Fig. 2a. Irradiation of photocatalyst **PC** (**I)** with visible light generates the long-lived excited state **II**, which is a strong oxidant, capable of oxidizing **2** to form a nucleophilic acyl radical **3**^57^ and a reduced state **III**. Meanwhile, the alkyne **4** reacts readily with acyl radical **3** to form the vinyl radical **5**. The open-shell radical **5** is expected to rapidly engage in addition to the aryl ring, generating the dearomatic radical **6**. At this stage, we hoped that this radical species **6** would undergo the critical hydrogen radical transfer step to generate key intermediate **7** or **7**′ assisted by HRS. Single-electron reduction of radical **7** or **7**′ by **III** to afford carbon anion **8** or **8**′, followed by protonation to afford the indanone **9**.

Our initial efforts sought to evaluate different potential HRSs which are effective for the direct assemble of indanones with potassium 2-oxo-2-phenylacetate **10** and phenylacetylene **4** as model substrates, along with Ir[dF(CF_3_)ppy]_2_(phen)PF_6_ as the photocatalyst under N_2_ with illumination by blue LEDs. After a series of explorations on several potential HRS catalysts (H_2_O, MeOH, EtOH, and acetic acid), to our delight, 22% yield of indanone was isolated with water as an additive (Fig. 2b). A trace amount of desired indanone **9** was detected without water.

Further screening of the reaction conditions using benzoylformic acid **1** and phenylacetylene **4** as model substrates found that the indanone **9** could be isolated in 86% yield using Ir[dF(CF_3_)ppy]_2_(phen)PF_6_ as the photocatalyst and water as HRS under N_2_ with illumination by blue LEDs at 100 °C (Fig. 2c). Control experiments revealed that the photocatalyst, visible light, and water were all essential components for achieving the high efficiency of this reaction (see Supplementary information for details).

### Substrate scope investigation

**Fig. 3:**
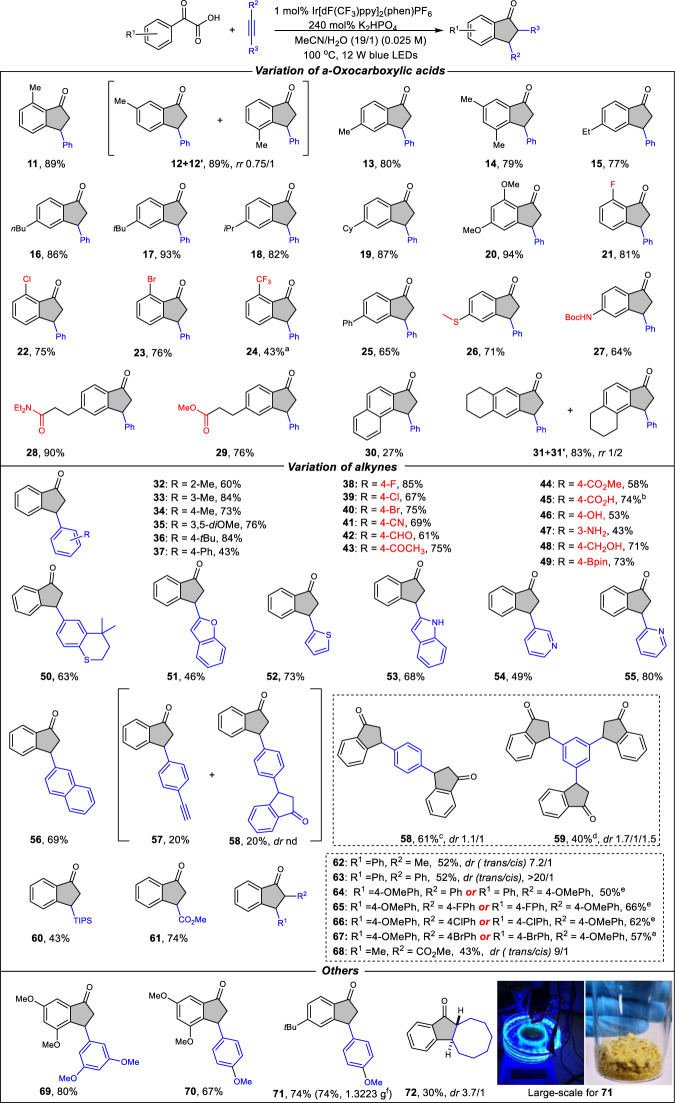
Exploration of substrate scope. Reactions were performed with acid (1.0 mmol), alkyne (0.5 mmol), Ir[dF(CF_3_)ppy]_2_(phen)PF_6_ (1 mol%), K_2_HPO_4_ (2.4 equiv.) MeCN (19 mL) and H_2_O (1 mL). 24 h, 100 °C, 12 W blue LEDs. Isolated yields. Regioselectivity ratio (rr) determined by ^1^H NMR. ^a^48 h. ^b^K_2_HPO_4_ (3.4 equiv.) was used. ^c^alkyne (0.25 mmol) was used. ^d^alkyne (0.167 mmol) was used. ^e^a mixture of regio- and diastereo-isomers, which is difficult to isolate. ^f^acid (12.0 mmol), alkyne (6.0 mmol), MeCN (120 mL), H_2_O (6 mL), 48 h.

With the optimized conditions in hand, we next evaluated the variations of 2-oxo-2-arylacetic acids and alkynes that are applicable to the developed reaction (Fig. 3). With respect to the 2-oxo-2-arylacetic acid partner, we observed moderate to excellent yields of the desired products (**11–31**) with a wide range of substrates bearing different substituents. The methyl groups at the *ortho-, meta-*, and *para-*positions on the phenyl ring of 2-oxo-2-arylacetic acid could be tolerated (**11–13**). With respect to the *meta*-substituted 2-oxo-2-phenylacetic acid, regioisomers **12** and **12**′ were obtained with 0.75/1*rr*. A range of 2-oxo-2-phenylacetic acids bearing both the electron-donating and electron-withdrawing substituents on the phenyl ring, no matter for 1°-, 2°-, 3°-alkyl substituents or linear, cyclic substituents, were amenable substrates (**14–24**). The strong electron-withdrawing substituents decrease the conversion and yields. This observation may be ascribed to the reduced reductive quenching ability toward photoexcited [Ir]^*^. The *sp*^2^-hybridized phenyl-substituted 2-oxo-2-phenylacetic acid underwent smoothly to give a 65% yield of indanones (**25**). Notably, 2-oxo-2-phenylacetic acid with additional functionalities was also compatible with this protocol. For example, various functional groups, such as ether, halides, trifluoromethyl, easily-oxidized thioether, ester, and amide remain intact to furnish the corresponding products (**26–29**). In addition to substituted 2-oxo-2-phenylacetic acid-type substrate, 2-(naphthalen-2-yl)-2-oxoacetic acid could also be successfully converted into the desired product **30** in reasonable yield. Interestingly, **31** and **31**′ could be obtained in 83% yield with region-selectivity (*rr* 1/2) from the corresponding substrates. Having established that this transformation tolerates various 2-oxo-2-arylacetic acid substrates, we then turned our attention towards evaluating the scope of the alkyne components. For the simple aromatic alkynes with alkyl or phenyl substituents, the corresponding products (**32–37**) were isolated in 43–84% yields. Evaluation of a series of alkynes that contained various functional groups, such as fluoro, chloro, bromo, nitrile, aldehyde, ketone, ester, acid, phenol, free amine and alcohol, provided indanones **38–48** in 43–85% yield, potentially allowing for the subsequent orthogonal functionalization. Notably, alkynes with synthetic handles, such as halides (**38**–**40**) and boronic ester **49**, were readily incorporated into the accessible indanone scaffolds, which highlights their potential applications for the incorporation of these scaffolds into more complex targets. Additionally, the developed protocol was also tolerant of the alkyne containing easily oxidized thioether, as demonstrated by **50**, which was isolated in 63% yield. Considering that heteroaryl-substituted compounds are highly desirable building blocks in drug discovery, we also evaluated a range of heteroaryl-substituted alkynes that would provide access to heteroaryl-substituted indanones. Although these scaffolds traditionally required multistep syntheses, the developed protocol allows for the construction of heteroaryl-substituted indanones in a single step from readily available precursors. For example, a wide range of five- and six-membered heteroaryl alkynes, such as benzofuran, thiophene, indole, and pyridine-derived substrates were functionalized with high efficiency (**51–55**). When 2-naphthyl alkyne was subjected to the standard conditions, a reaction occurred to afford the desired product **56** in 69% yield. Of particular note is that when 1,4-diethynylbenzene was subjected to the standard conditions, mono-cyclization product **57** could be obtained in 20% yield, accompanied with an equal amount of dicyclization product **58**. Dicyclization product **58** could be selectively produced in 61% yield with 1.1/1 *dr* when an excess amount of acid partner was used. Moreover, tricyclization compound **59** could be obtained in one step under the same reaction conditions by using 1,3,5-triethynylbenzene as an alkyne component. To further explore the scope of this reaction, other kinds of alkynes were also tested. The silicon-substituted alkynes and methyl propiolate were also suitable substrates to provide the titled products (**60**–**61**). Besides the terminal alkynes, the internal alkynes, including aromatic alkynes and alkyl alkynes, could also be successfully transformed into 2,3-disubstituted indanones with diastereometric ratios ranging from 7.2/1 to 20/1 (**62–68**). We also evaluated 2-oxo-2-phenylacetic acids and aromatic alkynes both with electron-donating substituents under standard conditions, providing the corresponding products in 67–80% yield (**69**–**71**). Importantly, the reaction could be reproduced on a 6 mmol scale to provide gram quantities of **71** in an increased concentration. There was almost no change in the chemical yield, suggesting that large-scale chemical production might be possible. It is noteworthy that cyclic internal alkyne, cyclooctyne, could also be transformed to the corresponding indanone **72** in 30% yield with 3.7/1 *dr*.

**Fig. 4:**
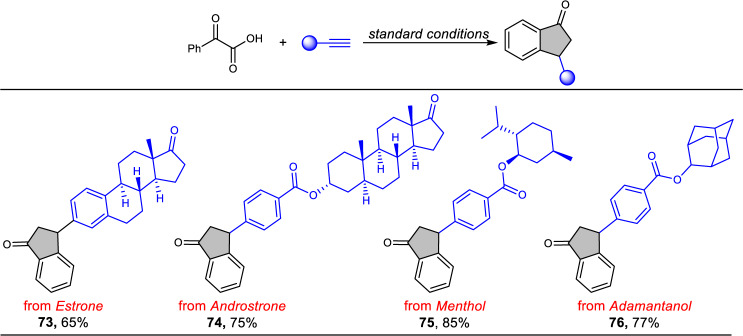
Late-stage functionalization of complex molecules. Reactions conditions: acid (1.0 mmol), alkyne (0.5 mmol), Ir[dF(CF_3_)ppy]_2_(phen)PF_6_ (1 mol%), K_2_HPO_4_ (2.4 equiv.) MeCN (19 mL) and H_2_O (1 mL). 24 h, 100 °C, 12 W blue LEDs. Isolated yields. The diastereometric ratio could not be determined by ^1^H NMR analysis.

To explore its utility for late-stage functionalization of complex molecules, several natural products or bioactive molecules-derived alkynes were tested for this developed reaction system. As shown in Fig. 4, the estrone-derived alkyne could be efficiently transformed into the indanone **73** in a 65% yield. In addition, aryl alkyne with an ester-linked androstrone also participated in this transformation smoothly, furnishing **74** in 75% yield. Similarly, the corresponding alkyne derived from menthol and adamantanol were both suitable alkyne partners for this protocol, affording the desired products **75** and **76** in 85% and 77% yield, respectively. These results show great potential for the structural modification of an array of complex biological molecules in medicinal chemistry.

**Fig. 5:**
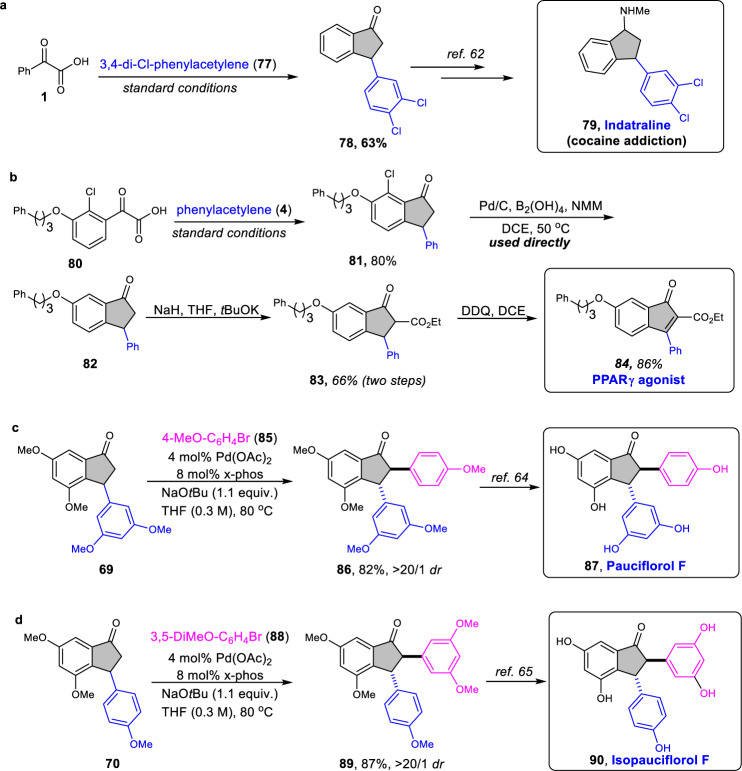
Synthetic applications of indanones. **a** Key intermediate for indatraline construction could be afforded by the developed protocol. **b** Synthesis of PPARγ agonist using this strategy. NMM 4-methylmorpholine. **c** Powerful protocol to prepare pauciflorol F. **d** Formal synthesis Isopauciflorol F.

To further showcase the synthetic utility of this developed strategy, we next made efforts on the synthesis of indanone-containing natural products, biologically and pharmaceutically molecules. For example, the 3-substituted indanone-1-one **78**, prepared using this method in 63% yield, was the key intermediate in the synthesis of indatraline **79**^62^, an approved and antidepressant drug (Fig. 5a). Moreover, synthesis of *PPAR γ* agonist **84**^63^ could be achieved via oxidated dehydrogenation of corresponding indanone **83**, which was prepared in three steps from α-oxocarboxylic acid **80** and **4** using our developed protocol, followed by dehalogenation and α-esterification (Fig. 5b). Importantly, pauciflorol F **87**^64^ and isopauciflorol F **90**^65^, both are natural products and bioactive molecules, could also be selectively assembled by using different alkynes and aryl halides (Fig. 5c and d).

**Fig. 6:**
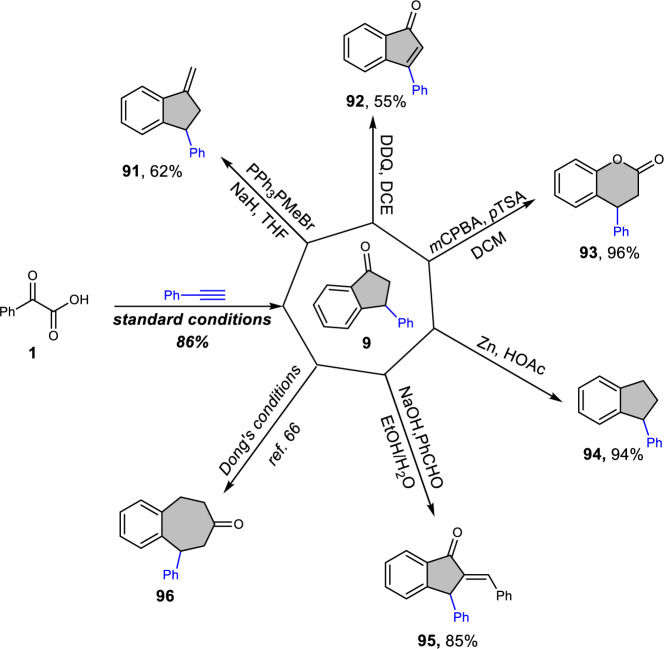
Further transformations of indanones. The indanone **9** was prepared from **1** under standard conditions. The indanone **9** was efficiently transformed to diverse compounds, such as alkene **91**, indenone **92**, lactone **93**, 1-phenyl-2,3-dihydro-1*H*-indene **94**, (*E*)-2-benzylidene-3-phenyl-2,3-dihydro-1*H*-inden-1-one **95**, and benzocycloheptenone **96**, respectively.

With the indanone scaffolds in hand, further chemical transformations were also performed to demonstrate the potential applications of these molecules (Fig. 6). Taking indanone **9** as an example, the terminal alkene **91**, 3-phenyl-1*H*-inden-1-one **92**, lactone **93,** and 1-phenyl-2,3-dihydro-1*H*-indene **94** could be obtained via the Wittig reaction, oxidation by 2,3-dichloro-5,6-dicyano-1,4-benzoquinone (DDQ), Baeyer–Villiger oxidation, and reduction by Zn/HOAc system, respectively. The methylene group of **9** reacted with an aldehyde to give (*E*)-2-benzylidene-3-phenyl-2,3-dihydro-1*H*-inden-1-one **95** efficiently. Particularly, benzocycloheptenone **96** can be prepared straightforwardly via the two-carbon ring expansion strategy with inexpensive ethylene developed by Dong′s group^66^.

**Fig. 7:**
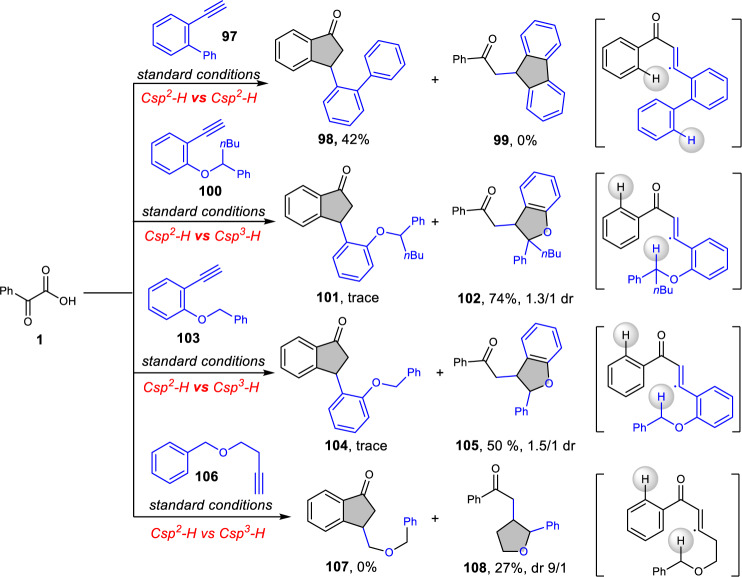
Mechanistic probes for chemo- and regio-selectivity. Competitions between different C–H bonds. The indanone **98** was formed in the presence of another C*sp*^2^–H bond. Only furan-derived products (**102**, **105** and **108**) could be isolated when weaker C*sp*^3^–H bonds have existed.

Given that the robust efficiency observed, we next turned our attention to a deeper exploration of the chemo- and regio-selectivity of this water-mediated reaction by including different type C–H bonds within various molecular probes (Fig. 7). An initial competition between multiple aryl C–H bonds within a single intermediate illustrates the absolute propensity for generation of indanone **98** (**98** vs. **99**). This observation indicates that the carbonyl group plays a key role during the cyclization process to provide a five-membered ring product. We next tested the competition of aryl C–H bonds and weaker C*sp*^3^–H bond involved in the intermediate. In these cases, the HAT between vinyl radical and weaker C–H bonds outcompetes water-mediated pathways affording the furan-derived products with little indanones detected (**101** vs. **102**, **104** vs. **105**, **107** vs. **108**). These phenomena not only showed obvious chemo-selectivity in the presence of weaker C–H bonds but also deliver strong evidence for the existence of vinyl radical. Moreover, the deuterium substitution experiment with D_2_O using substrate **100** was performed and found that no deuterated benzylic product was formed (see Supplementary Fig. 1 for details). The result indicated that the corresponding product was formed through an intramolecular 1,5-HAT followed by a Giese addition, which is not a water-mediated pathway.

**Fig. 8:**
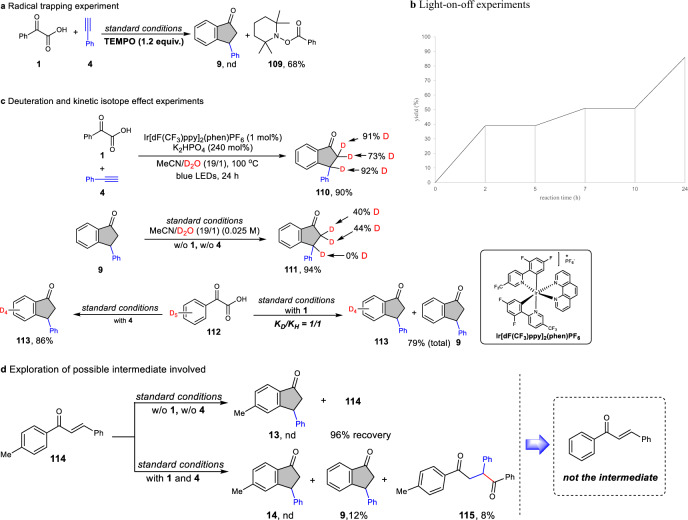
Mechanistic studies. **a** Radical-trapping experiment with 2,2,6,6-tetramethylpiperidinooxy (TEMPO). **b** Light-on–off the experiment. **c** Deuterated and kinetic isotope experiments. **d** Exploration of possible intermediate involved.

To better understand the detailed mechanism of the reaction, a series of mechanistic studies were performed (Fig. 8). In the presence of radical trap TEMPO, the reaction was completely shut down (Fig. 8a), indicating that a radical intermediate might be involved in this transformation. More importantly, acyl-trapped product, 2,2,6,6-tetramethylpiperidin-1-yl benzoate **109** was isolated in high yield, further supporting the reaction proceeds through a radical decarboxylation pathway and the intermediacy of an acyl radical. The light-on–off experiment demonstrates the radical chain mechanism is less likely involved (Fig. 8b). To verify if H_2_O was involved in the reaction as proposed, D_2_O was used and subjected to the optimal reaction conditions, deuterated product **110** was observed in 90% yield when D_2_O was utilized in place of H_2_O, demonstrating the benzylic site of hydrogen is originated from water (Fig. 8c, top). To further test whether deuterated product **110** was generated from the indanone **9** under standard conditions through H/D exchange with D_2_O, indanone **9** was subjected to the reaction conditions with D_2_O instead of H_2_O (Fig. 8c, middle). Interestingly, a deuterated indanone **111** was formed, in which only the CH_2_ of indanone was deuterated through H/D exchange with D_2_O and no deuterated benzylic product was formed. This experiment suggested that the benzylic C–H cannot be deuterated through H/D exchange with D_2_O under standard conditions. To verify whether the 1,5-HAT is involved in the reaction, deuterated phenylglyoxylic acid **112** was used as substrate under standard conditions (Fig. 8c, bottom, left). The absolute indanone **113** without deuterium transfer was obtained in 86% yield, demonstrating that the reaction did not proceed via 1,5-HAT pathway. In addition, it also indicated that no obvious direct hydrogen transfer occurred from the aryl position to the corresponding methylene and benzylic site of indanone, which is not in line with the 1,2- and 1,3-HAT. Taken together, the hydrogen atom of benzylic C–H of indanone should come from water during the catalytic reaction process, neither from the aryl C–H via 1,5-HAT nor from water through H/D exchange after reaction completion. Kinetic isotope experiments (KIE) were also performed to have more insight into the reaction mechanism (Fig. 8c, bottom, right). Since no obvious KIE effect (*K*_D_/*K*_H_ = 1/1) was detected when the equivalent of **1** and **112** were subjected to the reaction conditions with alkyne **4**, according to the intermolecular competition experiment, aryl C–H bond cleavage was not likely the rate-determining step. Additionally, when ketone **114** was performed under standard conditions, no cyclized product **13** was detected but with a recovery of **114** in 96% (Fig. 8d, top). Furthermore, when chalcone **114** was added as an additive to the model reaction, only the predictable indanone **9** and Giese-type reaction product **115** could be monitored (Fig. 8d, bottom). These control experiments strongly indicated that the chalcone **114** is less likely the intermediate involved in the reaction.

**Fig. 9:**
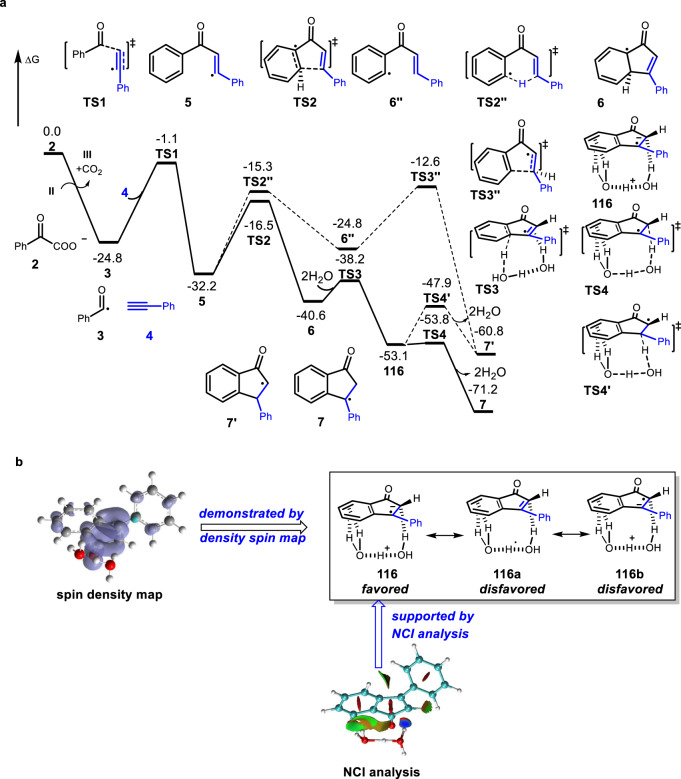
The free energy barrier for the reaction and the spin density and noncovalent interaction (NCI) analysis for the intermediate **116**. **a** Computational studies. **b** The spin density map and NCI analysis for the intermediate **116**.

DFT calculation was subsequently employed to provide further insight into the mechanism of this decarboxylative annulation reaction (Fig. 9a). According to the computational calculations, an intermolecular radical addition with phenylacetylene **4** takes place via transition state **TS1** with a free energy barrier of 23.7 kcal/mol to afford the vinyl radical **5**. Then an intramolecular radical addition would occur via transition state **TS2** with a free energy barrier of 15.7 kcal/mol to achieve annulation and form a dearomatized intermediate **6**. Because of the similar BDE values of C–H bonds between alkenes and arenes, we considered an alternative intramolecular 1,5-hydrogen shift theoretically via transition state **TS2**″. However, the relative free energy of generated phenyl radical **6**″ is 7.4 kcal/mol higher than that of vinyl radical **5**. Moreover, the relative free energy for the corresponding annulation transition state **TS3**″ is also 3.9 kcal/mol higher than that of **TS2**. This analysis revealed that the generation of intermediate **6** is a favorable pathway. These calculations were highly consistent with our experimental observations (Fig. 8c). Next, we focused on understanding the exact role of water in the formation of intermediate **7** or **7**′. When dearomatized intermediate **6** is formed, from calculations, a water-assisted stepwise 1,3-hydrogen transfer would provide a more stable benzylic radical **7** with rearomatization. In this process, two water molecules were used to achieve dehydrogenation via transition state **TS3** to afford a complex radical intermediate **116** with a free energy barrier of only 2.4 kcal/mol (see Supplementary Fig. 9 for other water molecules assisted pathway). Two other possible resonance structures of intermediate **116** could be drawn as electron-neutral indenone with hydrated hydrogen radical **116a** and zwitterionic in indenolate radical with protonated water **116b**. The spin density map of intermediate **116** clearly revealed that spin density is majorly located at indenone moiety. Meanwhile, the electrostatic potential map also exhibits a charge-separated character (Fig. 9b). Therefore, zwitterionic resonance structure **116** has a more appreciable contribution for this intermediate. Interestingly, noncovalent interaction (NCI) analysis of intermediate **116** also revealed a strong hydroxyl–π interaction between indenolate radical and hydrated proton, which explained the stability of this intermediate. When intermediate **116** is formed, a rapid protonation takes place via transition state **TS4** resulting in the formation of benzylic radical **7** with the release of two water molecules (see Supplementary Data 1 for the coordination of all the structures involved in the computational calculations). Therefore, as we designed, water acts as a hydrogen radical-shuttle catalyst, promoting the favorable 1,3-hydrogen transfer with the formation of intermediate **7**.

In summary, we have developed a decarboxylative annulation for indanones synthesis via photoredox/HAT catalysis with water as hydrogen radical-shuttle (HRS). This protocol provides a powerful platform to construct indanones with broad substrate scope, excellent functional group tolerance, internal hydrogen radical transfer, atom- and step-economy, using simple and available 2-oxo-2-phenylacetic acids and readily available alkynes. Moreover, the exact role of water in this developed strategy was demonstrated by mechanistic experiments and DFT calculations, as we designed, facilitating the hydrogen transfer and acting as the hydrogen source. Namely, acting as a HRS catalyst was critical to the success of this process. Additionally, the key intermediate **116** was further demonstrated by spin density map and NCI analysis. Most importantly, to the best of our knowledge, this system provides an aromatization model, representing a breakthrough in hydrogen radical transfer assisted by HRS. This hydrogen transfer is mechanistically distinctive from that of typical PS-promoted, providing a complementary process in hydrogen transfer and a feasible solution in achieving 1,2-, or 1,3-HAT. We expect this strategy could be widely adopted and further promote the development of direct functionalization of aryl C*sp*^2^-H via HRS-assisted hydrogen transfer.

## Methods

### Materials

Unless otherwise noted, all the materials were obtained commercially and used without further purification. All the solvents were treated according to general methods. Flash column chromatography was performed over silica gel (300–400 mesh). See Supplementary Methods for experimental details.

### General procedures for the indanones synthesis

To an oven-dried 50 mL flask, Ir[dF(CF_3_)ppy]_2_(phen)PF_6_ (0.005 mmol), 2-aryl-2-oxocarboxylic acid (1.0 mmol) and K_2_HPO_4_ (1.2 mmol) were added sequentially under N_2_. The flask was evacuated and back-filled with N_2_ for three times, then alkyne (0.5 mmol), H_2_O (1 mL), and MeCN (19 mL) was added. The reaction mixture was irradiated by 12 W blue LEDs at a distance of 5 cm for 24 h at 100 °C. The reaction mixture was cooled to rt and filtered through a short pad of silica using ethyl acetate. The filtrate was concentrated in vacuo before it was purified by flash chromatography on silica gel to afford the desired indanone product.

### Computational method

All the calculations in this study were performed using the Gaussian 16 program package.^67^ The All the geometries were optimized at the M06-2X^68^/6–31 G(*d*,*p*) and SDD for Ir level, and the solvent effect was utilized the polarizable continuum model using integral equation formalism model (IEFPCM) in hexane solvent.^69^ All the optimized stationary points had been identified as minima (zero imaginary frequencies) and transition states (one imaginary frequency), via the vibrational analysis. The solution-translational entropy correction has been calculated with the THERMO program.^70^

## Supplementary information

Supplementary Information

Description of Additional Supplementary Files

Supplementary Data 1

## Data Availability

The authors declare that the data relating to the characterization of materials and products, general methods, optimization studies, experimental procedures, mechanistic studies, HRMS data and NMR spectra, computational studies are available within the article and its Supplementary Information as well as supplementary data.
