# Supplementary material for: Hydrogen radical-shuttle (HRS)-enabled photoredox synthesis of indanones via decarboxylative annulation
Source: Nat Commun. 2021 Sep 6;12:5257. doi: 10.1038/s41467-021-25594-4 (PMC8421331; doi:10.1038/s41467-021-25594-4)
Supplement: Supplementary file 1 — Supplementary Information [file 41467_2021_25594_MOESM1_ESM.pdf]

SUPPLEMENTARY INFORMATION

Hydrogen Radical-Shuttle (HRS)-Enabled Photoredox Synthesis of Indanones via Decarboxylative Annulation

Bo Yang[1,2], Shi-Jun Li[3], Yongdong Wang[2]*, Yu Lan[3,4]*, Shifa Zhu[1]*

[1]Key Laboratory of Functional Molecular Engineering of Guangdong Province, School of Chemistry and Chemical Engineering, South China University of Technology, Guangzhou 510640, China. *email: zhusf@scut.edu.cn

[2]Singfar Laboratories, Guangzhou, 510670, China. *email: ydwang@vip.163.com

[3]Green Catalysis Center, and College of Chemistry, Zhengzhou University, 100 Science Avenue, Zhengzhou 450001 Henan, China. *email: lanyu@cqu.edu.cn

[4]School of Chemistry and Chemical Engineering, and Chongqing Key Laboratory of Theoretical and Computational Chemistry, Chongqing University, Chongqing 400030, China.

Table of Contents

I. Supplementary Methods 3

II. Supplementary Discussion 63

III. Supplementary NMR Spectra 74

IV. Supplementary References 169

I. Supplementary Methods

General information

THF and 1,4-dioxane were distilled from sodium benzophenone ketyl prior to use. Acetonitrile, DCM, *i*Pr_2NH and 1,2-dichloroethane were distilled from calcium hydride. Methanol was used directly. Unless otherwise noted, all the corresponding ketones and alkynes from suppliers were used directly without further purification. The water was used directly from suppliers. The Ir[dF(CF_3)ppy]$_2$(phen)PF_6 was prepared according to the literature (Reference: Lowry, M. S.; Goldsmith, J. I.; Slinker, J. D.; Rohl, R.; Pascal, R. A.; Malliaras, G. G.; Bernhard, S. *Chem. Mater.* **2005**, *17*, 5712.). NMR spectra were recorded on a Bruker-500 instrument. ^1H NMR chemical shifts were referenced to the tetramethylsilane (0 ppm); ^{13}C NMR chemical shifts were referenced to the solvent resonance (77.00 ppm, $CDCl_3$). The following abbreviations (or combinations thereof) were used to explain multiplicities: s = singlet, d = doublet, t = triplet, m = multiplet. Infrared (IR) spectra are recorded on a Nicolet 210 spectrophotometer and were recorded in potassium bromide (KBr) pellet. Mass spectra (HRMS) were obtained using Agilent UHD Accurate Mass Q-TOF LC/MS (ESI) mass spectrometer. Melting points were determined using a hot stage apparatus. All manipulations were conducted under Schlenk tubes. Unless noted, all the α-Oxocarboxylic acids were prepared from oxidation of the corresponding methyl ketones by SeO_2 according to the general procedures. The initial explorations were performed with a WP-TEC-1020HSL LED flow reactor (WATTCAS, China) and the systematic photochemical reactions were performed using Light Emitting Diode strip (LEDs) (long for 1 meter).

General procedure for evaluation of potential HRS

To an oven-dried 50 mL flask, Ir[dF(CF_3)ppy]$_2$(phen)PF_6 (0.005 mmol) and **10** (1.0 mmol) were added sequentially under N_2. The flask was evaculated and back-filled with N_2 for three times, then alkyne **4** (0.5 mmol), HRS (10.0 mmol) and MeCN (19 mL) was added. The reaction mixture was irradiated by 12W blue LEDS at a distance of 5 cm for 24 h at rt. The reaction mixture was filtered through a short pad of silica using EA. The filtrate was concentrated in *vacuo* before it was purified by flash chromatography on silica gel to afford indanone **9**.

Supplementary Table 1. Evaluation of potential HRS

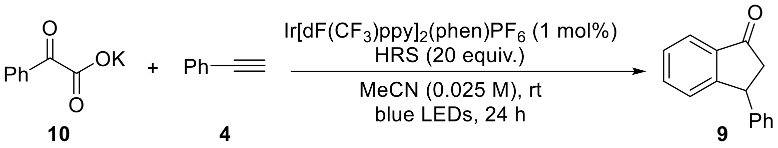

Entry	HRS	Yield (%)
1	H_2O	22
2	MeOH	5
3	EtOH	6
4	CH_3CO_2H	9
5	/	<5

Optimization of Reaction Conditions

Supplementary Table 2. Optimization of Solvents[a]

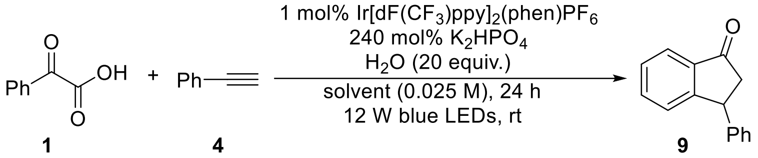

Entry	Solvent	Isolated yield (%)
1	$PhCF_3$	14
2	$CHCl_3$	<10
3	PhCl	32
4	DCE	19
5	MeOH	<10
6	toluene	19
7	acetone	34
8	THF	<10
9	Dioxane	17
10	MeCN	30
11	DMF	<10
12	DMSO	<10
13	DCM	35
14[b]	MeCN	41
15[b]	acetone	36
16[b]	DCM	25

[a] Standard conditions: 1 mmol of acid **1**, 0.5 mmol of alkyne **4**, 1 mol% of Ir[dF(CF_3)ppy]$_2$(phen)PF_6, K_2HPO_4 (2.4 equiv.), H_2O (20 equiv.), solvent (0.025 M) , under the irradiation of 12 W blue LEDS under N_2 atmosphere for 24 h at room temperature. [b] Solvent/H_2O (19/1) (0.025 M).

Supplementary Table 3. Optimization of Photocatalysts[a]

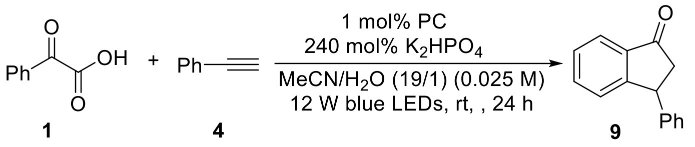

Entry	PC	Isolated yield (%)
1	Ir[dF(CF_3)(pp)$_2$](Phen)PF_6	41
2	Ir(ppy)$_2$(dtbpy)PF_6	<10
3	4CzIPN	16
4	Ir[dF(CF_3)(pp)$_2$](dtbpy)PF_6	22

[a] Standard conditions: 1 mmol of acid **1**, 0.5 mmol of alkyne **4**, 1 mol% of PC, K_2HPO_4 (2.4 equiv.), MeCN/H_2O (19/1) (0.025 M), under the irradiation of 12 W blue LEDS under N_2 atmosphere for 24 h at room temperature.

Supplementary Table 4. Optimization of Bases.[a]

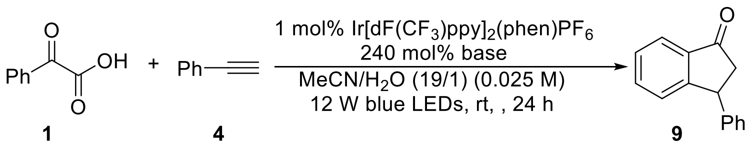

Entry	Base	Isolated yield (%)
1	K_2HPO_4	41
2	Cs_2CO_3	18
3	TMG	17
4	Imidazole	11
4	K_2CO_3	24
5	Na_2CO_3	28
6	pyridine	<10
7	2,6-lutidine	<10
8	K_3PO_4	16
9[b]	K_3PO_4	21

[a] Standard conditions: 1 mmol of acid **1**, 0.5 mmol of alkyne **4**, 1 mol% of Ir[dF(CF_3)(ppy)$_2$(phen)]PF_6, base (2.4 equiv.), MeCN/H_2O (19/1) (0.025 M) , under the irradiation of 12 W blue LEDS under N_2 atmosphere for 24 h at room temperature. [b] 100 mol% K_3PO_4.

Supplementary Table 5. Optimization of Different Temperatures[a]

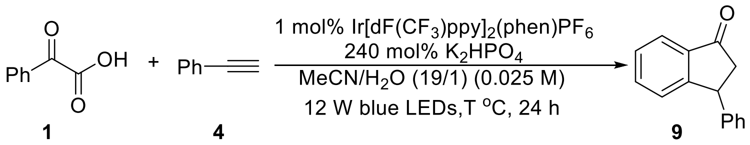

**Entry**	***T*** **(°C)**	**Isolated yield (%)**
1	50	57
2	60	58
3	70	66
4[b]	70	65
5	80	70
6	90	74
7	100	86
8	110	84

[a] Standard conditions: 1 mmol of acid **1**, 0.5 mmol of alkyne **4**, 1 mol% of Ir[dF(CF_3)(ppy)$_2$(phen)]PF_6, K_2HPO_4 (2.4 equiv.), MeCN/H_2O (19/1) (0.025 M) , under the irradiation of 12 W blue LEDS under N_2 atmosphere for 24 h at desired temperature. [b] 40 h.

Supplementary Table 6. Optimization of equivalents of acid[a]

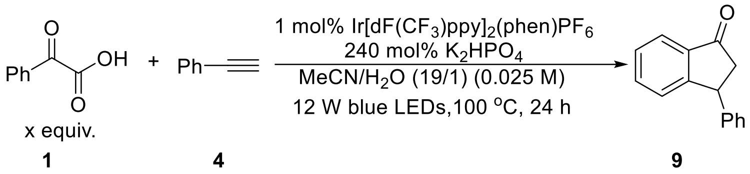

**Entry**	**x**	**Isolated yield (%)**
1	2.0	86
2	1.5	74
3	1.2	67

[a] Standard conditions: acid **1** (x equiv.), 0.5 mmol of alkyne **4**, 1 mol% of Ir[dF(CF_3)(ppy)$_2$(phen)]PF_6, K_2HPO_4 (2.4 equiv.), MeCN/H_2O (19/1) (0.025 M) , under the irradiation of 12 W blue LEDS under 100 °C for 24 h.

Supplementary Table 7. Optimization of Concentration[a]

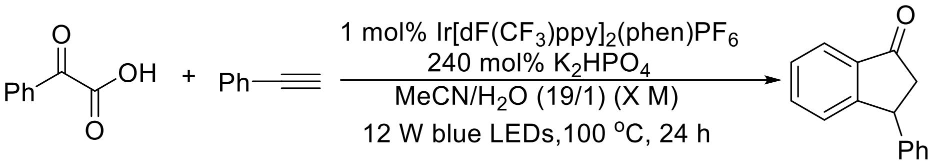

1 **4** **9**

Entry	X	Isolated yield (%)
1	0.025	86
2	0.05	81
3	0.1	66

[a] Standard conditions: 1 mmol of acid **1**, 0.5 mmol of alkyne **4**, 1 mol% of Ir[dF(CF_3)(ppy)$_2$(phen)]PF_6, K_2HPO_4 (2.4 equiv.), MeCN/H_2O (19/1) (X M) , under the irradiation of 12 W blue LEDS under 100 °C for 24 h.

General Procedure for Preparation of Substituted α-Keto Acids.

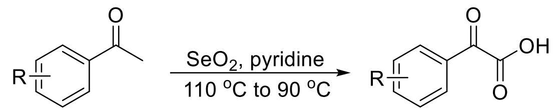

All kinds of substituted α-keto acids were prepared from oxidation of corresponding methyl ketones with SeO_2 according to the reported literature.[1]

To a solution of ketone (10 mmol) in pyridine (10 mL) was added SeO_2 (1.5 equiv.), the reaction mixture was heated for 2 h at 110 °C and then 90 °C for 9 hours. After cooled to room temperature, the reaction mixture was filtered through a short pad of celite. The most of the solvent was evaporated and the mixture was acidified by HCl (2N). The mixture was extracted with EA and the organic layers were dried over anhydrous Na_2SO_4 and filtered to afford the crude product which was purified by chromatography through silica gel to obtain the corresponding a-oxocarboxylic acids.

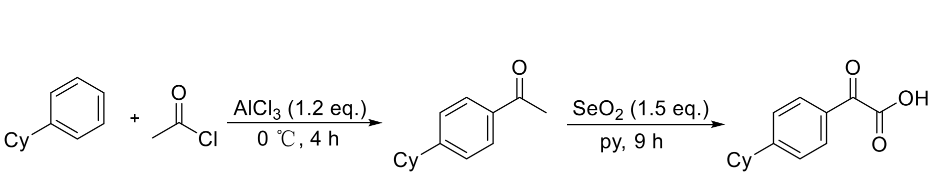

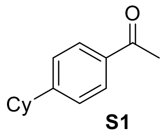

S1

1-(4-Cyclohexylphenyl)ethan-1-one (**S1**).[2] To an oven-dried 50 mL flask, cyclohexylbenzene (1.9231 g, 12.0 mmol) and carbon tetrachloride (12 mL) were added sequentially at 0 °C under N_2. The aluminum chloride (1.9201 g, 14.4 mmol) was added slowly. The reaction mixture was stirred for 4 hours at 0 °C. The reaction mixture was quenched by water and extracted by DCM. The combined organic layers were dried over anhydrous Na_2SO_4 and filtrated. The filtration was condensed to provide a crude product which was purified

by flash column chromatography (PE/EA = 30/1) on silica gel to afford **S1** (2.8900 g, 14.25 mmol, 95% yield) as a white solid. ^{1}H NMR (500 MHz, $CDCl_3$) δ 7.88 (d, *J* = 8.5 Hz, 2H), 7.28 (d, *J* = 8.5 Hz, 2H), 2.62-2.50 (m, 4H), 1.94-1.70 (m, 5H), 1.50-1.19 (m, 5H).

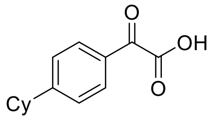

S2

2-(4-Cyclohexylphenyl)-2-oxoacetic acid (S2).[3] To a solution of ketone **S1** (2.0231 g, 10 mmol) in pyridine (10 mL) was added SeO_2 (1.6644 g, 15.0 mmol.), the reaction mixture was heated for 2 h at 110 °C and then 90 °C for 9 hours. After cooled to room temperature, the reaction mixture was filtered through a short pad of celite. The most of the solvent was evaporated and the mixture was acidified by HCl (2N). The mixture was extracted with EA and the organic layers were dried over anhydrous Na_2SO_4 and filtered to afford the crude product which was purified by flash chromatography on silica gel to obtain the corresponding corresponding acid **S2** (1.8100 g, 7.8 mmol, 78% yield) as a pale yellow solid. ^{1}H NMR (500 MHz, $CDCl_3$) δ 8.18 (d, *J* = 8.0 Hz, 2H), 7.35 (d, *J* = 8.5 Hz, 2H), 2.68-2.50 (m, 1H), 1.99-1.69 (m, 5H), 1.55 -1.18 (m, 5H).

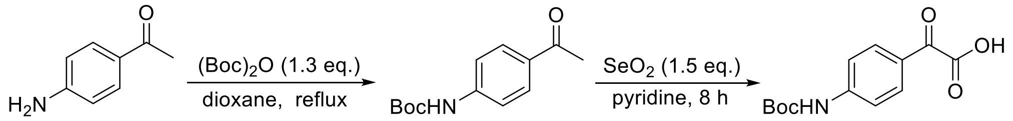

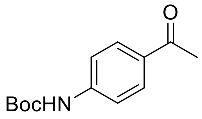

S3

***Tert*-butyl (4-acetylphenyl)carbamate (S3).**[4] To an oven-dried 50 mL flask, 4-aminoacetophenone (1.3510 g, 10.0 mmol), di-*tert*-butyl dicarbonate (2.8373 g, 13.0 mmol) and 1,4-dioxane (20 mL) were added sequentially at rt under N_2. The reaction mixture was refluxed for 8 hours. The reaction mixture was condensed to provide a crude product which was purified by flash column chromatography (PE/EA = 5/1) on silica gel to afford **S3** (1.7050 g, 7.30 mmol, 73% yield) as a white solid. ^{1}H NMR (500 MHz, $CDCl_3$) δ 7.91 (d, *J* = 8.5 Hz, 2H), 7.51 (d, *J* = 8.5 Hz, 2H), 7.29 (s, 1H), 2.57 (s, 3H), 1.51 (s, 9H).

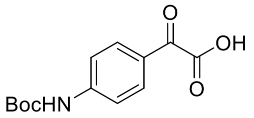

S4

2-(4-((*Tert*-butoxycarbonyl)amino)phenyl)-2-oxoacetic acid (S4).[5] To a solution of ketone (705.8 mg, 3.0 mmol) in pyridine (3 mL) was added SeO_2 (499.1 mg, 4.5 mmol.), the reaction mixture was heated for 2 h at 110 °C and then 90 °C for 9 hours. After cooled to room temperature, the reaction mixture was filtered through a short pad of celite. The most of the solvent was evaporated and the mixture was acidified by HCl (2N). The mixture was extracted with EA and the organic layers were dried over anhydrous Na_2SO_4 and filtered to afford the crude product which was purified by chromatography through silica gel to obtain the corresponding a-oxocarboxylic acids **S4** (1.8100 g, 7.8 mmol, 78% yield) as

a yellow solid. ^{1}H NMR (500 MHz, $CDCl_3$) δ 9.95 (s, 1H), 7.86 (d, *J* = 9.0 Hz, 2H), 7.70 (d, *J* = 9.0 Hz, 2H), 1.49 (s, 9H).

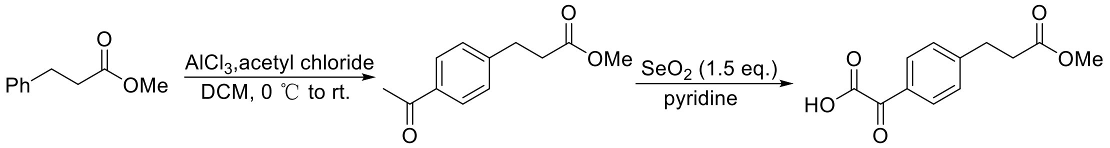

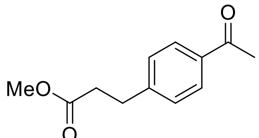

S5

Methyl 3-(4-acetylphenyl)propanoate (S5).[6] To a oven-dried 50 mL flask, phenyl propionic methyl ester (17.8228 g, 100.0 mmol) and DCM (200 mL) were added sequentially at 0 °C under N_2. The aluminum chloride (26.6680 g, 200.0 mmol) was added slowly. The reaction mixture was stirred overnight at 0 °C to rt. The reaction mixture was quenched by water and extracted by DCM. The combined organic layers were dried over anhydrous Na_2SO_4 and filtrated. The filtration was condensed to provide a crude product which was purified by flash column chromatography (PE/EA = 5/1) on silica gel to afford **S5** (16.4150 g, 80.0 mmol, 80% yield) as a yellow oil. ^{1}H NMR (500 MHz, $CDCl_3$) δ 7.88 (d, *J* = 8.5 Hz, 2H), 7.29 (d, *J* = 8.5 Hz, 2H), 3.65 (s, 3H), 3.00 (t, *J* = 7.5 Hz, 2H), 2.65 (t, *J* = 7.5 Hz, 2H), 2.56 (s, 3H).

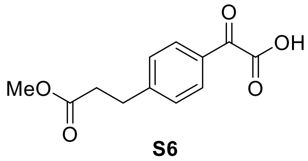

S6

2-(4-(3-Methoxy-3-oxopropyl)phenyl)-2-oxoacetic acid (S6). To a solution of ketone (1.0310 g, 5.0 mmol) in pyridine (5 mL) was added SeO_2 (832.2 mg, 7.5 mmol.), the reaction mixture was heated for 2 h at 110 °C and then 90 °C for 9 hours. After cooled to room temperature, the reaction mixture was filtered through a short pad of celite. The most of the solvent was evaporated and the mixture was acidified by HCl (2N). The mixture was extracted with EA and the organic layers were dried over anhydrous Na_2SO_4 and filtered to afford the crude product which was purified by flash chromatography on silica gel to obtain the corresponding acid **S6** (822.0 m g, 3.48 mmol, 70% yield) as a yellow oil. IR *v* 2955, 2566, 1729, 1605, 1440, 1367 cm^{-1}; ^{1}H NMR (500 MHz, $CDCl_3$) δ 10.04 (s, 1H), 8.12 (d, *J* = 8.0 Hz, 2H), 7.36 (d, *J* = 8.0 Hz, 2H), 3.69 (s, 3H), 3.04 (t, *J* = 7.5 Hz, 2H), 2.71 (t, *J* = 7.5 Hz, 2H); ^{13}C NMR (126 MHz, $CDCl_3$) δ 185.1, 173.6, 163.7, 148.6, 130.9, 130.1, 128.8, 51.9, 34.7, 30.7; HRMS (ESI-TOF) Calcd. for $C_{12}H_{13}O_5$ [M+H]$^+$: 237.0685; Found 237.0764.

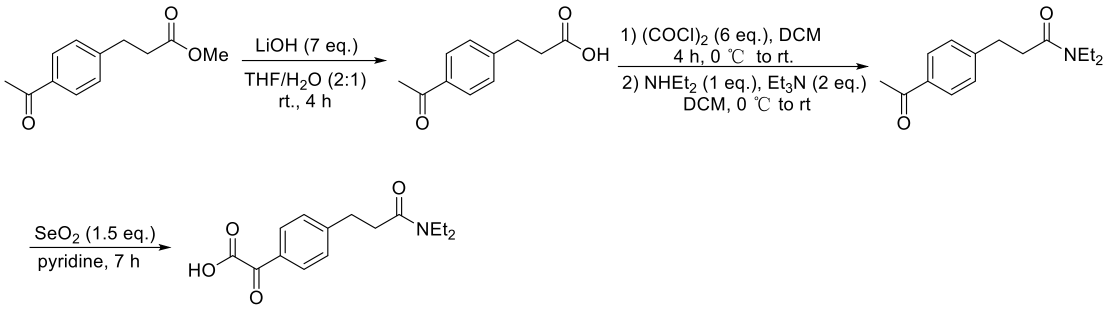

To a 50 mL flask, **S5** (2.0620 g, 10.0 mmol), THF (20 mL), H_20 (10 mL) and LiOH (1.6760 g, 70.0 mmol) were added sequentially at rt. The reaction mixture was stirred at room temperature and monitored by thin layer chromatography (TLC). When the starting material was consumed by TLC, HCl (1 N) was added to acidify the reaction mixture and the reaction mixture was extracted with EA. The combined organic layers were dried by Na_2SO_4. After filtration, the filtrate was condensed to afford a crude acid product **S7** which was used directly without further purification.

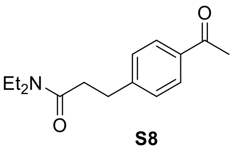

S8

***N, N*-Diethyl-3-(4-(2-oxopropanoyl)phenyl)propanamide (S8)**. To an oven-dried 50 mL flask, the crude acid (961.1 mg, 5.0 mmol) and DCM (12 mL) were added sequentially at 0 °C under N_2. The oxalyl chloride (2.53 mL, 30.0 mmol) was added slowly. The reaction mixture was allowed to be warmed to rt and stirred for 4 h at rt. Then diethylamine (365.0 mg, 5.0 mmol) and triethylamine (1.0120 g, 10.0 mmol) were added slowly at 0 °C. The mixture was stirred overnight at 0 °C to rt. The reaction mixture was quenched by water and extracted by DCM. The combined organic layers were dried over anhydrous Na_2SO_4 and filtrated. The filtration was condensed to provide a crude product which was purified by flash column chromatograpy to afford **S8** (1.2050 g, 4.35 mmol, 87% yield) as a yellow oil. IR *v* 2973, 2933, 1680, 1638, 1431, 1268 cm^{-1}; ^1H NMR (500 MHz, $CDCl_3$) δ 7.88 (d, *J* = 8.0 Hz, 2H), 7.33 (d, *J* = 9.5 Hz, 2H), 3.38 (q, *J* = 7.0 Hz, 2H), 3.25 (q, *J* = 7.0 Hz, 2H), 3.05 (t, *J* = 7.5 Hz, 2H), 2.63 (t, *J* = 7.5 Hz, 2H), 2.58 (s, 3H), 1.15-1.07 (m, 6H); ^{13}C NMR (126 MHz, $CDCl_3$) δ 197.5, 170.4, 147.2, 135.0, 128.5, 128.3, 41.7, 40.0, 34.1, 31.2, 26.3, 14.0, 12.8; HRMS (ESI-TOF) Calcd. for $C_{15}H_{22}NO_2$ $[M+H]^+$: 248.1651; Found 248.1648.

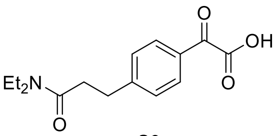

S9

2-(4-(3-(Diethylamino)-3-oxopropyl)phenyl)-2-oxoacetic acid (S9). Prepared according to the general procedure affording **S9** (1.2050 g, 4.35 mmol, 87% yield) as a yellow solid. mp = 104-105 °C. IR *v* 2934, 1729, 1681, 1567, 981 cm^{-1}; ^1H NMR (500 MHz, DMSO-d_6) δ 7.84 (d, *J* = 8.5 Hz, 2H), 7.48 (d, *J* = 8.0

Hz, 2H), 3.24 (q, *J* = 7.0 Hz, 4H), 2.93 (t, *J* = 7.5 Hz, 2H), 2.63 (t, *J* = 7.5 Hz, 2H), 1.03 (t, *J* = 7.0 Hz, 3H), 0.98 (t, *J* = 7.0 Hz, 3H); ^{13}C NMR (126 MHz, DMSO) δ 188.5, 169.9, 166.4, 149.9, 129.9, 129.6, 129.4, 41.3, 33.2, 31.0, 14.2, 13.1; HRMS (ESI-TOF) Calcd. for $C_{15}H_{20}NO_4$ $[M+H]^+$: 278.1392; Found 278.1389.

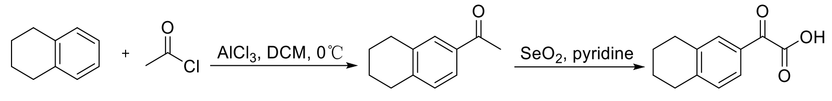

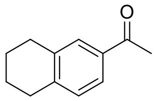

S10

1-(5,6,7,8-Tetrahydronaphthalen-2-yl)ethan-1-one (S10). [7] To an oven-dried flask cooled under N_2, tetralin (2.64 g, 20.0 mmol), $AlCl_3$ (4.0 g, 30 mmol) and DCM (20 mL) were added. Acetyl chloride (1.7 mL, 24.0 mmol) was added slowly at 0 °C. The reaction mixture was stirred at 0 °C to rt overnight. The reaction was quenched by HCl (1N) at 0 °C, extracted by DCM and dried over anhydrous Na_2SO_4. After filtration, the filtrate was condensed and purified by column chromatography (PE/EA = 20:1) on silica gel to obtain ketone **S10** (1.8 g, 10.1 mmol, 51% yield) as a yellow oil. ^{1}H NMR (500 MHz, $CDCl_3$) δ 7.67-7.62 (m, 2H), 7.10 (d, *J* = 8.5 Hz, 1H), 2.84-2.74 (m, 4H), 2.54 (s, 3H), 1.83-1.75 (m, 4H).

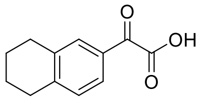

S11

2-Oxo-2-(5,6,7,8-tetrahydronaphthalen-2-yl)acetic acid (S11). Prepared according to the general procedure affording **S11** (1.9000 g, 9.31 mmol, 93% yield) as a yellow solid. mp: 63-65 °C. IR *v* 3456, 1640, 1400 cm^{-1}; ^{1}H NMR (500 MHz, $CDCl_3$) δ 8.79 (s, 1H), 7.93-7.85 (m, 2H), 7.16 (d, *J* = 8.5 Hz, 1H), 2.86-2.74 (m, 4H), 1.86-1.73 (m, 4H); ^{13}C NMR (126 MHz, $CDCl_3$) δ 185.3, 164.4, 146.4, 138.0, 131.7, 129.7, 129.2, 127.7, 29.9, 29.1, 22.7, 22.5; HRMS (ESI-TOF) Calcd. for $C_{12}H_{12}O_3Na$ $[M+Na]^+$: 227.0684; found 227.0682.

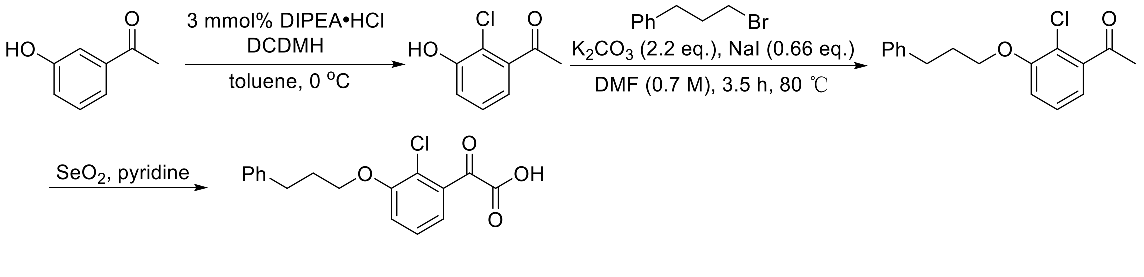

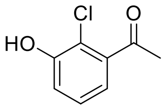

S12

1-(2-Chloro-3-hydroxyphenyl)ethan-1-one (S12).[8] To a solution of the *m*-acetylphenol (2.4515 g, 18 mmol), catalyst DIPEA·HCl (74.0 mg, 0.537 mmol) in toluene (45 mL) was added DCDMH (3.5461 mg, 18.0 mmol) at 0 °C in the absence of light. The mixture was stirred at 0 °C for 4 h and quenched by saturated aqueous Na_2SO_3. The solution was diluted with water and extracted with CH_2Cl_2 (3 × 30 mL). The combined organic layers were washed with brine, dried with anhydrous Na_2SO_4, filtered and concentrated under *vacuum*. The residue was purified by column chromatography on silica gel to yield the corresponding *ortho*-chlorinated product **S12** (2.5081 g, 14.70 mmol, 82% yield) as a white solid. ^{1}H NMR (500 MHz, $CDCl_3$) δ 7.24 (d, *J* = 8.0 Hz, 1H), 7.17-7.13 (m, 2H), 6.03 (s, 1H), 2.62 (s, 3H).

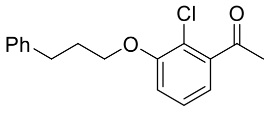

S13

1-(2-Chloro-3-(3-phenylpropoxy)phenyl)ethan-1-one (S13). To a stirred solution of *ortho*-chlorinated substrate **S12** (1.7056 g, 10.0 mmol) in DMF (15 mL) was added K_2CO_3 (2.9990 g, 21.7 mmol), NaI (989.3 mg, 6.6 mmol) and 1-bromo-3-phenylpropane (2.9860 g, 15.0 mmol). The mixture was stirred at rt for 4 hours. Then the mixture was quenched by H_2O and extracted with ethyl acetate. The combined organic layers were dried over anhydrous Na_2SO_4 and filtered. The filtrate was condensed and the residue was purified by flash column chromatography to obtain the desired product **S13** (2.5790 g, 8.95 mmol, 90% yield) as a yellow oil. IR *v* 1637, 1400, 1269 cm^{-1}; ^{1}H NMR (500 MHz, $CDCl_3$) δ 7.30-7.25 (m, 2H), 7.24-7.17 (m, 4H), 7.02 (dd, *J* = 7.5, 1.5 Hz, 1H), 6.93 (dd, *J* = 8.0, 1.5 Hz, 1H), 4.01 (t, *J* = 6.0 Hz, 2H), 2.86 (t, *J* = 7.5 Hz, 2H), 2.62 (s, 3H), 2.19-2.12 (m, 2H); ^{13}C NMR (126 MHz, $CDCl_3$) δ 201.1, 154.8, 141.3, 141.1, 128.45, 128.37, 127.5, 125.9, 120.0, 119.7, 114.9, 68.1, 31.8, 30.8, 30.5; HRMS (ESI-TOF) Calcd. for $C_{17}H_{17}ClO_2Na$ [M+Na]$^+$: 311.0815; Found 311.0811.

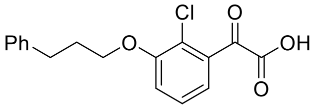

80

2-(2-Chloro-3-(3-phenylpropoxy)phenyl)-2-oxoacetic acid (80). Prepared according to the general procedure affording 80 (1.0940 g, 3.40 mmol, 69% yield) as a yellow solid. mp 137-138 °C. IR *v* 3700, 2925, 1706, 1571, 1446, 1273 cm^{-1}; ^{1}H NMR (500 MHz, DMSO-*d*$_6$) δ 7.48-7.43 (m, 1H), 7.39 (dd, *J* = 8.5, 1.5 Hz, 1H), 7.31-7.27 (m, 3H), 7.25-7.22 (m, 2H), 7.21-7.17 (m, 1H), 4.10 (t, *J* = 6.5 Hz, 2H), 2.79 (t, *J* = 7.5 Hz, 2H), 2.11-2.04 (m, 2H); ^{13}C NMR (126 MHz, DMSO-*d*$_6$) δ 189.1, 164.6, 154.2, 141.1, 135.1, 128.4, 128.3, 125.9, 122.2, 120.4, 117.7, 68.2, 31.3, 30.1; HRMS (ESI-TOF) Calcd. for $C_{17}H_{16}ClO_4$ [M+H]$^+$: 319.0737; Found 319.0729.

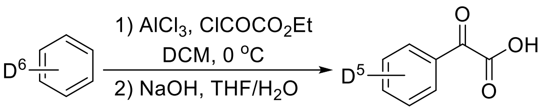

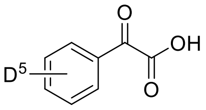

112

2-Oxo-2-(phenyl-*d*$_5$)acetic acid (112).[9] To an oven-dried 100 mL flask, C_6D_6 (1.0 mL, 11.0 m=mol), ethyl oxalyl chloride (2.2 g, 16.5 mmol) and DCM (25 mL) were added. The resulting mixture was cooled to 0 °C and stirred for 10 min. Then $AlCl_3$ (2.8 g, 21.0 mmol) was added over 30 min. When the reaction mixture turned red-brown, the reaction was quenched by water. Then the mixture was extracted with DCM and the combined organic layers were dried over anhydrous Na_2SO_4 and the solvent was removed under reduced pressure. The resulting product was used directly without further purification. The crude product was dissolved in THF (10 mL) and H_2O (10 mL). The NaOH (0.8 g, 20.0 mmol) was added. The mixture was stirred for for 5 hours at 40 °C. Then the organic solvent was removed and the residue was acidified with aqueous HCl (2N). Then the mixture was extracted with ethyl acetate. The combined organic layers were dried over anhydrous Na_2SO_4 and evaporated to provide the desired product **112** (1.2800 g, 8.25 mmol, 75% yield).

Procedures for Synthesis of Alkynes.

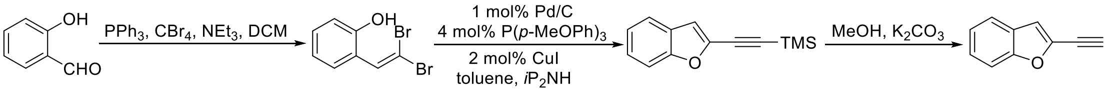

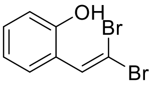

S14

2-(2,2-Dibromovinyl)phenol (S14).[10] To a 500 mL flask were added PPh_3 (23.60 g, 90.0 mmol) and CH_2Cl_2 (90 mL). The mixture was cooled to 0 °C, after which CBr_4 (14.90 g, 45.0 mmol) dissolved in CH_2Cl_2 (90 mL) was added and stirred for 10 min. NEt_3 (12.5 mL, 90.0 mmol) was added dropwise and stirred for an additional 5 minutes, after which salicyaldehyde (1.57 mL, 15.0 mmol) in CH_2Cl_2 (90 mL) was added dropwise. The mixture was stirred for an additional 30 min at 0 °C, then warmed to rt and stirred for 1 h. The reaction was quenched by saturated aqueous NH_4Cl solution. The phases were then separated, and the aqueous layer was extracted with CH_2Cl_2 (100 mL). The combined organic layers were concentrated to approximately 10% of the original volume. The residue was dissolved in Et_2O (100 mL) and filtered over a short pad of celite. The resulting solution was concentrated and purified by column chromatography on silica gel (PE/EtOAc: 15/1) to yield **S14** (1.2883 g, 4.64 mmol, 86%)

as a yellow solid. ^{1}H NMR (500 MHz, $CDCl_3$) δ 7.61-7.47 (m, 2H), 7.22 (d, J = 7.5 Hz, 1H), 6.95 (dd, J = 7.5, 7.0 Hz, 1H), 6.82 (d, J = 8.0 Hz, 1H), 5.10 (s, 1H).

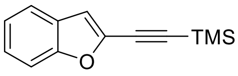

S15

(Benzofuran-2-ylethynyl)trimethylsilane (S15).[11] A reaction tube was charged with 2-(2,2-dibromovinyl)phenol (978.8 mg, 3.52 mmol), 10% Pd/C (76.3 mg), P(p-MeOPh)$_3$ (51.5 mg, 0.14 mmol) and CuI (18.6 mg, 0.098 mmol). Then the flask was evacuated and purged with N_2 three times. To this mixture were added toluene (10 mL), iPr_2NH (1.3 mL, 9.0 mmol) and trimethylsilylacetylene (5 ml, 35.0 mmol), and then heated to 100 °C with stirring overnight. The reaction mixture was then cooled to rt and H_2O (10 mL) was added. The mixture was extracted with EtOAc, and combined extracts were washed with sat. NH_4Cl and brine, then dried and solvent was removed in *vacuo*. The resulting crude material was purified by flash chromatography eluting with PE to afford **S15** as a yellow oil. ^{1}H NMR (500 MHz, $CDCl_3$) δ 7.53 (d, J = 8.0 Hz, 1H), 7.43 (d, J = 8.5 Hz, 1H), 7.34-7.29 (m, 1H), 7.23-7.19 (m, 1H), 6.94 (s, 1H), 0.29 (s, 9H).

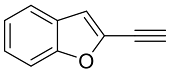

S16

2-Ethynylbenzofuran (S16).[12] To a stirred solution of **S15** (311.7 mg, 1.45 mmol) in methanol (3 mL) was added potassium carbonate (608.0 mg, 4.4 mmol). The reaction mixture was stirred for 5 hours. The mixture was filtered and evaporated before it was purified by flash chromatography eluting with PE to afford **S16** (119.4 mg, 0.84 mmol, 58% yield) as a yellow oil. ^{1}H NMR (500 MHz, $CDCl_3$) δ 7.59-7.52 (m, 1H), 7.46 (dd, J = 8.4, 1.0 Hz, 1H), 7.37-7.32 (m, 1H), 7.25 (ddd, J = 9.5, 5.5, 1.0 Hz, 1H), 7.01 (d, J = 0.8 Hz, 1H), 3.50 (s, 1H).

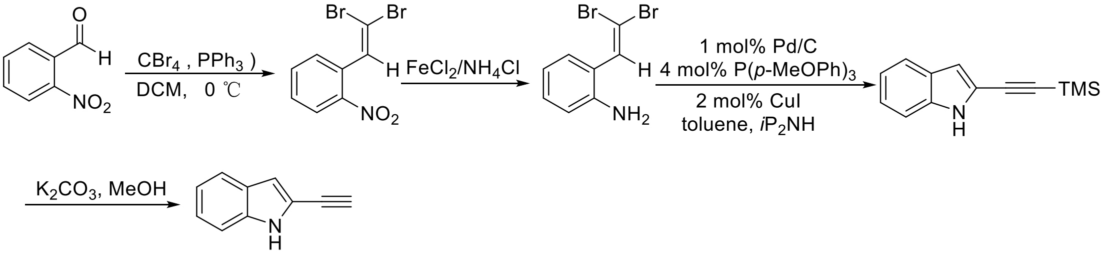

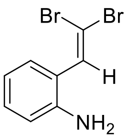

S17

1-(2,2-Dibromovinyl)aniline (S17).[13] To a stirred solution of 2-nitro benzaldehyde (3.0220 g, 20.0 mmol) and tetrabromomethane (9.9480 g, 30.0 mmol) in dichloromethane (90 mL) at 0 °C was added triphenyl phosphine (15.7370 g, 60.0 mmol) slowly in small lots so that internal temperature was at 0°C. After addition, the mixture was stirred for another 30 min at 0 °C before warmed to rt and stirred for another 1h. The reaction

mixture was filtered through a short pad of silica gel and the silica gel was washed with dichloromethane until no product was found. Solvent was removed under *vacum* to give a solid mixture of desired product and triphenyl phosphine oxide. To the mixture was added absolute ethanol (60 mL) under nitrogen followed by the addition of iron powder (3.3600 g, 60.0 mmol) and ammonium chloride (3.2086 g, 60.0 mmol). The resulted suspension was heated to 90 °C under nitrogen for 1 h. The mixture was cooled to rt and most of the solvent was removed under vacuum. The residue was dissolved in EtOAc and pass through a short pad of celite and washed with EtOAc. To the filtrate was added saturated potassium carbonate solution and the resulted mixture was extracted with EtOAc. The combined organic layers were washed with brine and dried over anhydrous sodium sulfate. Solvent was removed under vacuum. The crude product was purified by flash chromatography on silica gel to afford the desired compounds **S17** (3.9882 g, 14.4 mmol, 72% yield) as a brown liquid. ^{1}H NMR (500 MHz, $CDCl_3$) δ 7.32 (s, 1H), 7.29 (d, *J* = 7.5 Hz, 1H), 7.17-7.12 (m, 1H), 6.80-6.74 (m, 1H), 6.68 (d, *J* = 8.0 Hz, 1H), 3.59 (s, 2H).

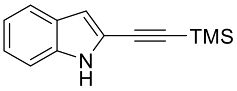

S18

2-((Trimethylsilyl)ethynyl)-1*H*-indole (S18).[11] A reaction tube was charged with 2-(2,2-dibromovinyl)aniline (1.1070 g, 4.0 mmol), 10% Pd/C (80.1 mg), P(*p*-OMeP)$_3$ (56.4 mg, 0.16 mmol), and CuI (15.2 mg, 0.08 mmol), and was evacuated and purged with argon three times. To this mixture were added toluene (15 mL) iPr$_2$NH (1.5 mL, 10.3 mmol) and trimethylsilylacetylene (5.6 ml, 40.0 mmol), and then heated to 100 ºC with stirring for 12 h. The reaction mixture was then cooled to rt and H_2O (20 mL) added. The mixture was extracted with EtOAc, and combined extracts were washed with sat. NH_4Cl and brine, then dried and solvent was removed in *vacuo*. The resulting crude material was purified by flash chromatography eluting with 10% EtOAc in PE to afford **S18** (512.0 mg, 2.4 mmol, 57%) as a pale brown solid. ^{1}H NMR (500 MHz, $CDCl_3$) δ 8.17 (s, 1H), 7.58 (d, *J* = 8.0 Hz, 1H), 7.30 (dd, *J* = 8.0, 1.0 Hz, 1H), 7.22 (td, *J* = 8.0, 1.0 Hz, 1H), 7.11 (td, *J* = 8.0, 1.0 Hz, 1H), 6.78 (d, *J* = 1.0 Hz, 1H), 0.28 (s, 9H).

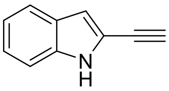

S19

2-Ethynyl-1*H*-indole (S19).[14] To a stirred solution of **S18** (256.0 mg, 1.20 mmol) in methanol (3 mL) was added potassium carbonate (414.6 mg, 3.0 mmol). The reaction mixture was stirred overnight. The mixture was filtered and evaporated before it was purified by flash chromatography to afford **S19** (118.6 mg, 0.84 mmol, 70% yield) as brown solid. ^{1}H NMR (500 MHz, $CDCl_3$) δ 8.01 (s, 1H), 7.56 (dd, *J* = 8.0, 1.0 Hz, 1H), 7.24-7.16

(m, 2H), 7.13-7.08 (m, 1H), 6.80 (d, *J* = 2.0 Hz, 1H), 3.26 (s, 1H).

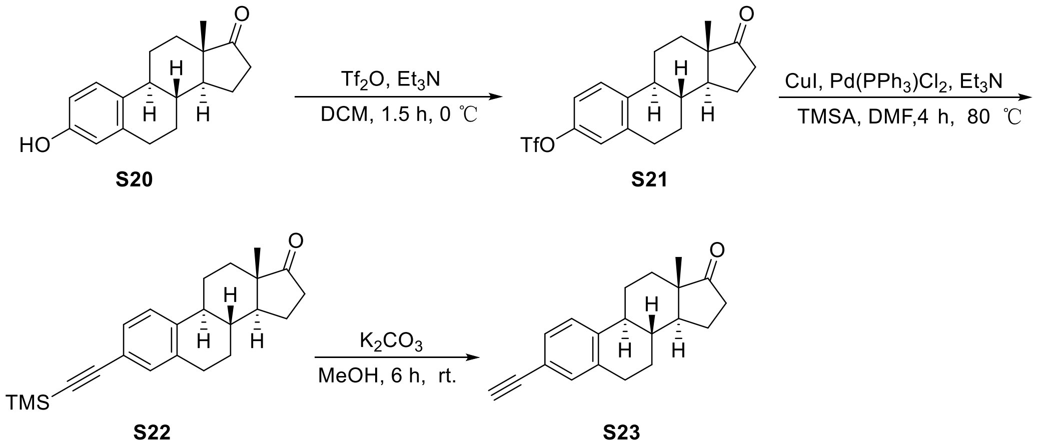

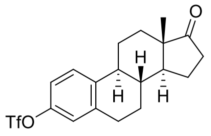

S21

(8*R*,9*S*,13*S*,14*S*)-13-methyl-17-oxo-7,8,9,11,12,13,14,15,16,17-decahydro-6*H*-cyclopenta[a]phenanthren-3-yl trifluoromethanesulfonate (S21).[15] To **S20** (1.8927 g, 7.0 mmol) in dry DCM (30 mL) at 0 ºC was added triethylamine (1.95 mL, 14.0 mmol) and trifluoromethanesulfonic anhydride (1.41 mL, 8.4 mmol). The reaction mixture was stirred at 0 ºC for 1.5 hours before the addition of water. The phases were separated and the aqueous phase was extracted with DCM (3 × 40 mL). The combined organic phases are washed with brine and dried over Na_2SO_4. The filtrate was concentrated in *vacuo* and the residue was purified by flash column chromatography on silica gel to afford **S21** (2.80 g, 6.93 mmol, 99% yield) as a solid. ^{1}H NMR (500 MHz, $CDCl_3$) δ 7.35 (d, *J* = 8.5 Hz, 1H), 7.05-7.02 (m, 1H), 7.00 (s, 1H), 2.97-2.93 (m, 2H), 2.52 (dd, *J* = 18.5, 8.5 Hz, 1H), 2.42-2.39 (m, 1H), 2.33-2.27 (m, 1H), 2.20-2.05 (m, 3H),2.01-1.96 (m, 1H), 1.70-1.42 (m, 6H), 0.93 (s, 3H).

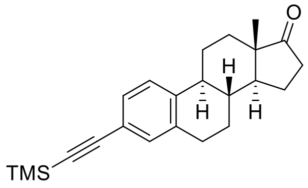

S22

(8*R*,9*S*,13*S*,14*S*)-13-methyl-3-((trimethylsilyl)ethynyl)-6,7,8,9,11,12,13,14,15,16-decahydro-17*H*-cyclopenta[a]phenanthren-17-one(S22).[12] To a mixture of **S21** (1.8910 g, 4.7 mmol), $Pd(PPh_3)_2Cl_2$ (329.9 mg, 0.47 mmol) and CuI (89.0 mg, 0.47 mmol) in DMF (47 mL) was added Et_3N (1.95 mL, 14.1 mmol), and Trimethylsilylacetylene (1.0 mL, 7.1 mmol), then the reaction was stirred at 80 ºC for 4 h. Monitored by TLC, when the reaction was completed, the mixture was quenched with water and extracted with EtOAc (50 mL x 3). The combined organic phases were washed with brine and dried over Na_2SO_4. The filtrate was concentrated in *vacuo* and the residue was purified by flash column chromatography on silica gel to afford **S22** (1.2500 g, 5.27 mmol, 76% yield) as a white solid. ^{1}H NMR (500 MHz,

CDCl3) δ 7.25-7.18 (m, 3H), 2.88-2.83 (m, 2H), 2.49 (dd, J = 19.0, 9.0 Hz, 1H), 2.41-2.36 (m, 1H), 2.29-2.24 (m, 1H), 2.14-1.93 (m, 4H), 1.62-1.38 (m, 6H), 0.90 (s, 3H), 0.24 (s, 9H).

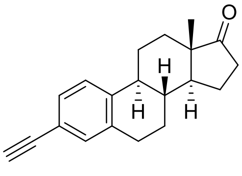

S23

(8*R*,9*S*,13*S*,14*S*)-3-ethynyl-13-methyl-6,7,8,9,11,12,13,14,15,16-decahydro-17*H*-cyclopenta[a]phenanthren-17-one (S23).[12] To **S19** (701.1 mg, 2.0 mmol) in MeOH (20 mL) was added K_2CO_3 (552.8 mg, 4.0 mmol). The reaction mixture was stirred at 25 °C for 4 h. Monitored by TLC, when the reaction was completed, the mixture was quenched with water and extracted with EtOAc (20 mL x 3). The combined organic phases are washed with brine and dried over Na_2SO_4. The filtrate was concentrated in vacuo and the residue was purified by flash column chromatography to afford **S23** (500.0 mg, 1.80 mmol, 90% yield) as a white solid. ^{1}H NMR (500 MHz, CDCl3) δ 7.29-7.22 (m, 3H), 3.02 (s, 1H), 2.90-2.86 (m, 2H), 2.51 (dd, J = 19.0, 9.0 Hz,1H), 2.42-2.38 (m, 1H), 2.31-2.26 (m, 1H), 2.18-1.94 (m, 4H), 1.67-1.36 (m, 6H), 0.91 (s, 3H).

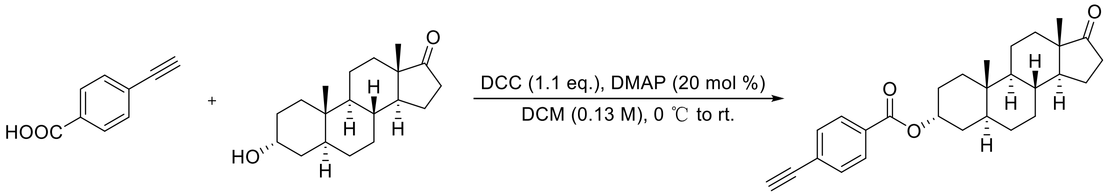

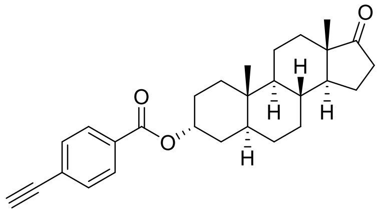

S24

(3*R*,5*S*,8*R*,9*S*,10*S*,13*S*,14*S*)-10,13-dimethyl-17-oxohexadecahydro-1*H*-cyclopenta[a]phenanthren-3-yl 4-ethynylbenzoate (S24). To an oven-dried 50 mL flask, 4-ethynylbenzoic acid (292.0 mg, 2.0 mmol), androstrone (528.6 mg, 1.82 mmol), DMAP (44.5 mg, 0.364 mmol) and DCM (14 mL) were added sequentially under N_2 at 0 °C. Then DCC (412.6 mg, 2.0 mmol) was added and stirred for 10 min at this temperature and stirred at rt overnight. The reaction mixture filtered through a short pad of silica gel and washed by DCM. The filtrate was concentrated in *vacuo* before it was purified by flash chromatography on silica gel to afford the desired compound **S24** (560.0 mg, 1.35 mmol, 74%) as a white solid. mp = 140-141 °C. IR *v* 2931, 1738, 1713, 1306, 1274 cm^{-1}; ^{1}H NMR (500 MHz, CDCl3) δ 8.00 (d, J = 8.5 Hz, 2H), 7.56 (d, J = 8.5 Hz, 2H), 5.30-5.26 (m, 1H), 3.24 (s, 1H), 2.44 (dd, J = 19.5, 9.0 Hz, 1H), 2.12-2.02 (m, 1H), 1.97-1.79 (m, 5H), 1.78-1.45 (m, 8H), 1.40-1.23 (m, 6H), 1.10-0.97 (m, 1H), 0.87 (d, J = 2.0 Hz, 6H); ^{13}C NMR (126 MHz, CDCl3) δ 221.1, 165.1, 132.0, 131.0, 129.3, 126.5, 82.8, 79.9, 70.8, 54.4, 51.4, 47.7, 40.4, 36.0, 35.7, 35.0, 33.1,

32.8, 31.5, 30.7, 28.0, 26.2, 21.7, 20.0, 13.8, 11.3; HRMS (ESI-TOF) Calcd. for $C_{28}H_{35}O_3$ $[M+H]^+$: 419.2586; Found 419.2585.

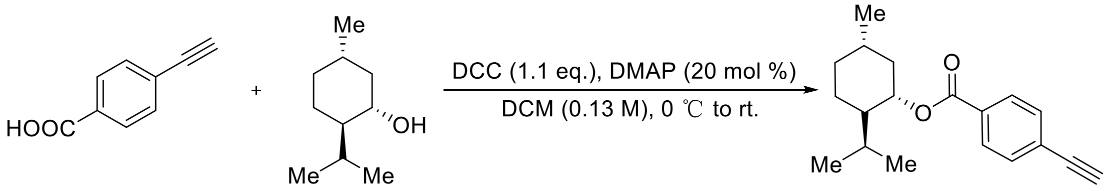

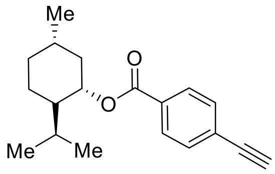

S25

(1*S*,2*S*,5*S*)-2-isopropyl-5-methylcyclohexyl 4-ethynylbenzoate (S25). To an oven-dried 50 mL flask, 4-ethynyl-benzoic acid (292.0 mg, 2.0 mmol), D-Menthol (284.5 mg, 1.82 mmol), DMAP (44.5 mg, 0.364 mmol) and DCM (14 mL) were added sequentially under N_2 at 0 °C. Then DCC (412.6 mg, 2.0 mmol) was added and stirred for 10 min at this temperature and stirred at rt overnight. The reaction mixture filtered through a short pad of silica gel and washed by DCM. The filtrate was concentrated in *vacuo* before it was purified by flash chromatography on silica gel to afford the desired compound **S25** (430 mg, 1.51 mmol, 83%) as a yellow oil. IR *v* 3298, 1714,, 1272, 1111 cm^{-1}; ^1H NMR (500 MHz, $CDCl_3$) δ 8.00 (d, J = 8.0 Hz, 2H), 7.55 (d, J = 8.0 Hz, 2H), 4.93 (td, J = 10.5, 4.0 Hz, 1H), 3.23 (s, 1H), 2.17-2.07 (m, 1H), 2.01-1.87 (m, 1H), 1.78-1.66 (m, 2H), 1.63-1.46 (m, 2H), 1.20-1.02 (m, 2H); 0.98-0.87 (m, 7H), 0.79 (d, J = 7.0 Hz, 3H); ^{13}C NMR (126 MHz, $CDCl_3$) δ 165.3, 131.9, 130.8, 129.4, 126.5, 82.8, 79.8, 75.1, 47.2, 40.9, 34.2, 31.4, 26.5, 23.6, 22.0, 20.7, 16.5; HRMS (ESI-TOF) Calcd. for $C_{19}H_{25}O_2$ $[M+H]^+$: 285.1855; Found 285.1854.

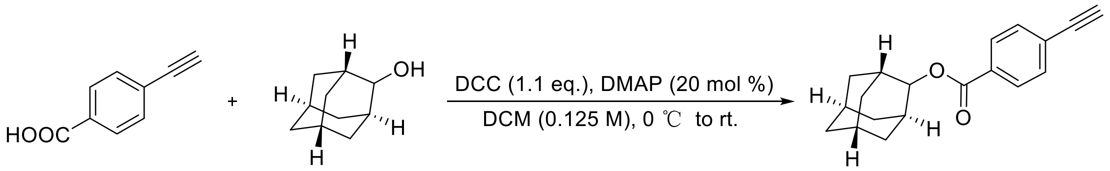

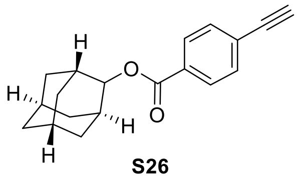

S26

(1*r*,3*r*,5*r*,7*r*)-adamantan-2-yl 4-ethynylbenzoate (S26). To an oven-dried 50 mL flask, 4-ethynyl-benzoic acid (292.0 mg, 2.0 mmol), 2-Adamantanol (277.0 mg, 1.82 mmol), DMAP (44.5 mg, 0.364 mmol) and DCM (14 mL) were added sequentially under N_2 at 0 °C. Then DCC (412.6 mg, 2.0 mmol) was added and stirred for 10 min at this temperature and stirred at rt overnight. The reaction mixture filtered through a short pad of silica gel and washed by DCM. The filtrate was concentrated in *vacuo* before it was purified by flash chromatography on silica gel to afford the desired compound **S26** (400 mg, 1.43 mmol, 79%) as a white solid. mp = 128-129 °C. IR

v 3249, 2906, 2851, 1716, 1271, 1114 cm^{-1}; ^{1}H NMR (500 MHz, $CDCl_3$) δ 8.04 (d, J = 8.5 Hz, 2H), 7.55 (d, J = 8.5 Hz, 2H), 5.21-5.16 (m, 1H), 3.24 (s, 1H), 2.22-2.09 (m, 4H), 1.95-1.74 (m, 8H), 1.69-1.59 (m, 2H); ^{13}C NMR (126 MHz, $CDCl_3$) δ 165.0, 132.0, 131.1, 129.4, 126.4, 82.9, 79.8, 77.8, 37.3, 36.3, 31.99, 31.97, 27.3, 27.0; HRMS (ESI-TOF) Calcd. for $C_{19}H_{21}O_2$ [M+H]$^+$: 281.1542; Found 281.1540.

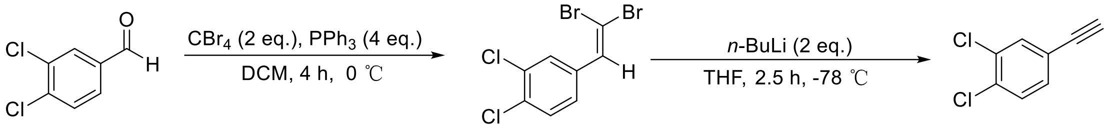

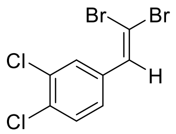

S27

1,2-Dichloro-4-(2,2-dibromovinyl)benzene (S27).[16] To a stirred solution of tetrabromomethane (13.2645 g, 40.0 mmol) in anhydrous DCM (130 mL), PPh_3 (20.9838 g, 80.0 mmol, dissolved in 12 mL DCM) was added dropwise at 0 °C. The reaction mixture was stirred for 1.5 h at 0 °C and 3,4-dichlorobenzaldehyde (3.4810 g, 20.0 mmol,) was added slowly and stirred for another 2.5 h. After completion of the reaction (monitored by TLC), the reaction mixture was diluted with petroleum ether and filtered through a short pad of silica gel. The organic layers was evaporated under reduced pressure to afford a crude residue. This crude residue was purified through column chromatography using PE as eluents to afford the alkene **S27** (6.52 g, 19.8 mmol, 99 % yield).as a yellow oil. ^{1}H NMR (500 MHz, $CDCl_3$) δ 7.63 (d, J = 2.0 Hz, 1H), 7.42 (d, J = 8.5 Hz, 1H), 7.37 (s, 1H), 7.35 (dd, J = 8.5, 2.0 Hz, 1H).

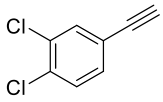

77

1,2-Dichloro-4-ethynylbenzene (77).[17] To an oven-dried flask cooled under N_2 was added **S27** (1.6400 g, 5.0 mmol) and THF (12 mL). After cooled to -78 °C, *n*BuLi (2.5 M) (4 mL, 10 mmol) was added dropwise and stirred for another 2.5 h. After completion of the reaction (monitored by TLC), the reaction mixture was quenched by Sat. NH4Cl and extracted with EA. The combined organic layers were dried over anhydrous Na_2SO_4, filtered and condensed under *vacuo*. The residue was purified through column chromatography using PE as eluents to afford alkyne **77** (580.0 mg, 3.40 mmol, 68%) as a yellow solid. ^{1}H NMR (500 MHz, $CDCl_3$) δ 7.57 (d, J = 2.0 Hz, 1H), 7.39 (d, J = 8.5 Hz, 1H), 7.31 (dd, J = 8.5, 2.0 Hz, 1H), 3.14 (s, 1H).

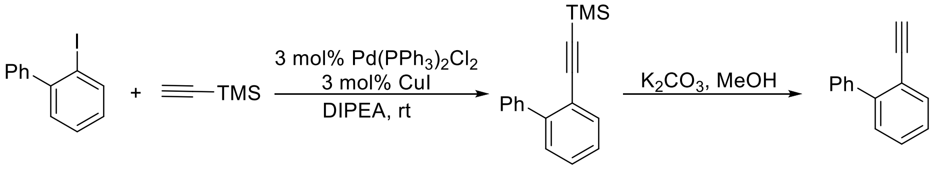

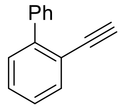

97

2-Ethynyl-1,1'-biphenyl (97).[18] To an 100 mL oven-dried flask cooled under N_2 atmosphere, CuI (58.7 mg, 0.3 mmol), $Pd(PPh)_3Cl_2$ (210.1 mg, 0.3 mmol), 2-Iodobiphenyl (2.8459 g, 10.16 mmol), DIPEA (50 mL) and trimethylsilylacetylene (1.7 mL, 12.0 mmol) were added. The reaction was stirred overnight at room temperature. The mixture was filtrated, and the filtration was condensed to provide a crude product which was purified by column chromatography (PE) on silica gel to afford a crude product. The crude product was then dissolved in MeOH (3 mL), and K_2CO_3 (414.6 mg, 3.0 mmol) was added. The reaction was stirred overnight at rt. After filtration, the filtrate was condensed and the residue was purified by column chromatography (PE) on silica gel to obtain the desired alkyne **97** (205.3 mg, 1.15 mmol) as a yellow oil. ^1H NMR (500 MHz, $CDCl_3$) δ 7.61 (d, J = 8.0 Hz, 1H), 7.59-7.55 (m, 2H), 7.44-7.40 (m, 2H), 7.40-7.33 (m, 3H), 7.32-7.26 (m, 1H), 3.02 (s, 1H).

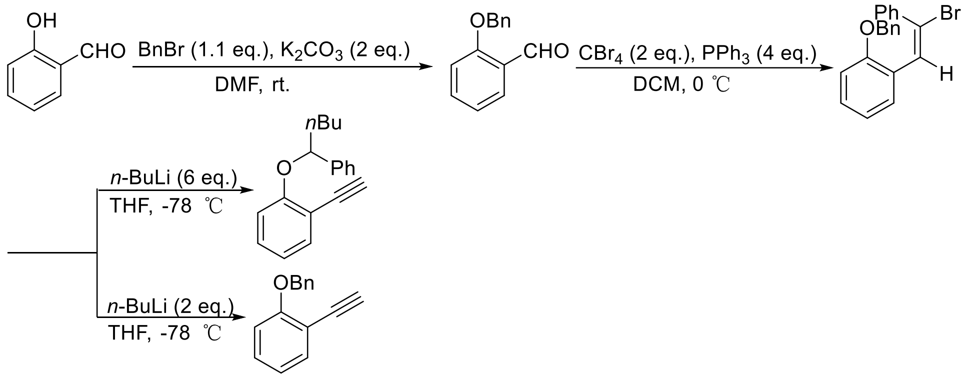

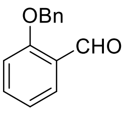

S28

2-(Benzyloxy)benzaldehyde (S28).[19] To a 50 mL oven-dried flask cooled under N_2 atmosphere, salicyaldehyde (3.6640 g, 30 mmol) (bromomethyl)benzene (5.6441 g, 33.0 mmol), potassium carbonate (8.2929 g, 60 mmol) and DMF (30 mL) were added. The reaction was stirred for 3 hours at rt. When the starting material was consumed (monitored by TLC), the reaction mixture was filtered and the filtrate was concentrated to give a crude product which was purified by flash column chromatography (PE/EA = 10/1) to obtain the desired product **S28** (6.300 g, 29.68 mmol, 99%) as a white solid. ^1H NMR (500 MHz, $CDCl_3$) δ 10.55 (s, 1H), 7.85 (dd, J = 8.0, 2.0 Hz, 1H), 7.54-7.48 (m, 1H), 7.46-7.36 (m, 4H), 7.36-7.30 (m, 1H), 7.06-6.99 (m, 2H), 5.17 (s, 2H).

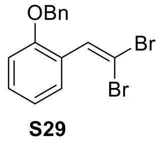

S29

1-(Benzyloxy)-2-(2,2-dibromovinyl)benzene (S29).[20] To a 250 mL oven-dried flask cooled under N_2 atmosphere, carbon tetrabromide (9.9480 g, 30 mmol), triphenylphosphine (15.7380 g, 60.0 mmol) and dichloromethane (100 mL) were

added at 0 °C. The reaction was stirred for 1.5 h at 0 °C. Then **S28** (3.1810 g, 15.0 mmol) (in 20 mL DCM) was added dropwise. When the starting material was consumed (monitored by TLC), the reaction mixture was diluted with petroleum ether and filtered through a short pad of celite. The filtrate was concentrated to give a crude product which was purified by flash column chromatography (PE/EA = 20/1) to obtain the desired product **S29** (4.1560 g, 11.36 mmol, 76%) as a yellow oil. ^{1}H NMR (500 MHz, $CDCl_3$) δ 7.70 (dd, *J* = 8.0, 1.0 Hz, 1H), 7.65 (s, 1H), 7.41-7.33 (m, 4H), 7.33 -7.22 (m, 2H), 6.97-6.92 (m, 1H), 6.88 (d, *J* = 8.0 Hz, 1H), 5.06 (s, 2H).

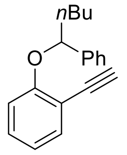

100

1-Ethynyl-2-((1-phenylpentyl)oxy)benzene (100). To a 50 mL oven-dried flask cooled under N_2 atmosphere, **S29** (1.8290 g, 5.0 mmol) and THF (12 mL) were added at -78 °C. *n*BuLi (2.5 M, 6 mL) was added dropwise. Then the reaction mixture was stirred at -78 °C for 2.5 h. The mixture was quenched by sat. NH_4Cl and extracted with ethyl acetate. The combined organic layers were dried over anhydrous Na_2SO_4, filtered and concentrated to obtain the crude product which was purified by chromatography (PE/EA = 50/1) on silica gel to obtain the desired compound **100** (1.1750 g, 4.44 mmol, 89% yield) as a yellow oil. IR *v* 3296, 2955, 2932, 1595, 1484, 1248 cm^{-1}; ^{1}H NMR (500 MHz, $CDCl_3$) δ ^{1}H NMR (500 MHz, $CDCl_3$) δ 7.48 (d, *J* = 7.5 Hz, 1H), 7.45-7.34 (m, 4H), 7.29 (dd, *J* = 7.5, 7.0 Hz, 1H), 7.16-7.09 (m, 1H), 6.85 (t, *J* = 7.5 Hz, 1H), 6.72 (d, *J* = 8.5 Hz, 1H), 5.21 (t, *J* = 6.0 Hz, 1H), 3.36 (s, 1H), 2.18-2.07 (m, 1H), 1.98-1.88 (m, 1H), 1.66-1.57 (m, 1H), 1.53-1.36 (m, 3H), 0.96 (t, *J* = 7.0 Hz, 3H); ^{13}C NMR (126 MHz, $CDCl_3$) δ 159.4, 141.8, 133.9, 129.7, 128.4, 127.4, 125.9, 120.2, 114.1, 112.3, 80.98, 80.96, 80.3, 38.3, 27.6, 22.5, 14.0; HRMS (ESI-TOF) Calcd. for $C_{19}H_{20}ONa$ [M+Na]: 287.1412; Found 287.1417.

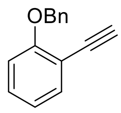

103

1-(Benzyloxy)-2-ethynylbenzene (103).[20] To a 50 mL oven-dried flask cooled under N_2 atmosphere, **S29** (439.0 mg, 1.2 mmol) and THF (5 mL) were added at -78 °C. *n*BuLi (2.5 M, 0.96 mL) was added dropwise. Then the reaction mixture was stirred at -78 °C for 3.5 h. The mixture was quenched by sat. NH_4Cl and extracted with ethyl acetate. The combined organic layers were dried over anhydrous Na_2SO_4, filtered and concentrated to obtain the crude product which was purified by chromatography (PE/EA = 50/1) on silica gel to obtain the desired compound **103** (1.1750 g, 4.44 mmol, 89% yield) as a colorless oil. ^{1}H NMR (500 MHz, $CDCl_3$) δ 7.50-7.42 (m, 3H), 7.38-7.33 (m, 2H), 7.31-7.26 (m, 1H), 7.26-7.20 (m, 1H), 6.92-6.85 (m, 2H), 5.17 (s, 2H), 3.29 (s, 1H).

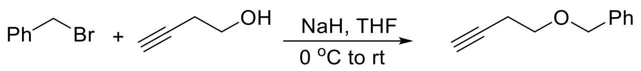

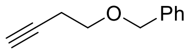

106

((But-3-yn-1-yloxy)methyl)benzene (106).[20] To a 50 mL oven-dried flask cooled under N_2 atmosphere, 3-butyn-1-ol (1.20 mL, 15.6 mmol) and THF (10 mL) were added. After cooled to 0 °C. Sodium hydride (60%, 625.0 mg, 15.6 mmol), tetrabutylammonium iodide (481.0 mg, 1.3 mmol) and benzyl bromide (1.54 mL, 13.0 mmol) was added sequentially. Then the reaction mixture was stirred at 0 °C for rt overnight. The mixture was quenched by sat. NH_4Cl and extracted with diethyl ether. The combined organic layers were washed by brine and dried over anhydrous Na_2SO_4. After filtration, the filtrate was evaporated in *vacuo* to provide a crude product which was purified by column chromatography (PE/EA = 15/1) on silica gel to afford **106** (1.4258 g, 8.90 mmol, 68% yield) as a yellow oil. ^{1}H NMR (500 MHz, $CDCl_3$) δ 7.34 (d, J = 4.0 Hz, 4H), 7.31-7.25 (m, 1H), 4.56 (s, 2H), 3.60 (t, J = 7.0 Hz, 2H), 2.50 (td, J = 7.0, 2.5 Hz, 2H), 1.99 (t, J = 2.5 Hz, 1H).

General Procedure for HRS-Enabled Synthesis of Indanones.

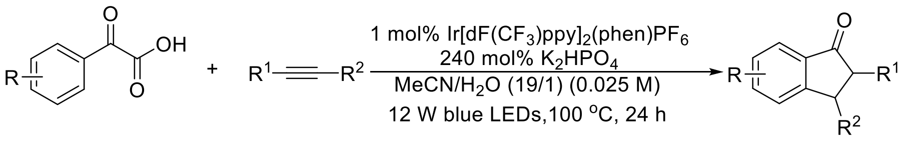

To an oven-dried 50 mL flask, Ir[dF(CF_3)ppy]$_2$(phen)PF_6 (0.005 mmol), acid (1.0 mmol) and K_2HPO_4 (1.2 mmol) were added sequentially under N_2. The flask was evaculated and back-filled with N_2 for three times, then alkynes (0.5 mmol), H_2O (1 mL) and MeCN (19 mL) was added. The reaction mixture was irradiated by 12 W blue LEDS at a distance of 5 cm for 24 h at 100 °C. The reaction mixture was cooled to rt and filtered through a short pad of silica using EA. The filtrate was concentrated in *vacuo* before it was purified by flash chromatography on silica gel to afford indanone products.

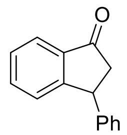

9

3-Phenyl-2,3-dihydro-1*H*-inden-1-one (9).[21] Prepared according to the general procedure employing Ir[dF(CF_3)ppy]$_2$(phen)PF_6 (5.2 mg, 0.0059 mmol), 2-Oxo-2-phenylacetate **1** (153.9 mg, 1.0 mmol), phenylacetylene **4** (0.5 mmol, 55 μL), K_2HPO_4 (210.4 mg, 1.2 mmol), MeCN (19 mL) and H_2O (1 mL). After 24 h, the reaction mixture was cooled to rt and filtered through a short pad of silica using EA. The filtrate was concentrated in *vacuo* before it was purified by flash chromatography (PE/EA = 15/1) on silica gel to afford **9** (89.6 mg, 0.43 mmol, 86% yield) as a yellow solid. ^{1}H NMR (500 MHz, $CDCl_3$) δ 7.81 (d, J = 8.0 Hz, 1H), 7.60-

7.53 (m, 1H), 7.41 (dd, *J* = 7.5, 7.5 Hz, 1H), 7.34-7.26 (m, 3H), 7.25-7.22 (m, 1H), 7.15-7.09 (m, 2H), 4.57 (dd, *J* = 8.0, 4.0 Hz, 1H), 3.23 (dd, *J* = 19.0, 8.0 Hz, 1H), 2.69 (dd, *J* = 19.0, 4.0 Hz, 1H); ^{13}C NMR (126 MHz, $CDCl_3$) δ 205.9, 157.9, 143.6, 136.7, 135.0, 128.9, 127.8, 127.6, 126.9, 126.8, 123.3, 46.8, 44.4.

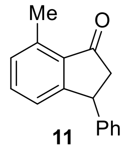
11

7-Methyl-3-phenyl-2,3-dihydro-1*H*-inden-1-one (11).[22] Prepared according to the general procedure employing Ir[dF(CF_3)ppy]$_2$(phen)PF_6 (5.5 mg, 0.005 mmol), 2-(2-Methylphenyl)-2-oxoacetic acid (153.9 mg, 1.0 mmol), phenylacetylene **4** (0.5 mmol, 55 μL), K_2HPO_4 (208.8 mg, 1.2 mmol) , MeCN (19 mL) and H_2O (1 mL). After 24 h, the reaction mixture was cooled to rt and filtered through a short pad of silica using EA. The filtrate was concentrated in *vacuo* before it was purified by flash chromatography (PE/EA = 15/1) on silica gel to afford **11** (99.4 mg, 0.45 mmol, 89% yield) as a yellow oil. ^1H NMR (500 MHz, $CDCl_3$) δ 7.38 (t, *J* = 7.5 Hz, 1H), 7.32-7.26 (m, 2H), 7.25-7.20 (m, 1H), 7.14-7.09 (m, 3H), 7.05 (d, *J* = 7.5 Hz, 1H), 4.48 (dd, *J* = 8.5, 5.0 Hz, 1H), 3.17 (dd, *J* = 19.0, 8.5 Hz, 1H), 2.71-2.62 (m, 4H); ^{13}C NMR (126 MHz, $CDCl_3$) δ 206.8, 158.7, 144.0, 138.4, 134.2, 134.0, 129.5, 128.7, 127.6, 126.8, 124.2, 47.2, 43.8, 18.3.

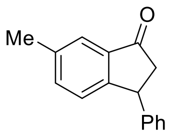
12

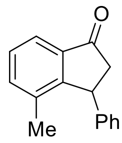
12'

6-Methyl-3-phenyl-2,3-dihydro-1*H*-inden-1-one (12).[22]**4-Methyl-3-phenyl-2,3-dihydro-1*H*-inden-1-one (12').** Prepared according to the general procedure employing Ir[dF(CF_3)ppy]$_2$(phen)PF_6 (5.3 mg, 0.005 mmol), 2-oxo-2-(*m*-tolyl)acetic acid (164.2 mg, 1.0 mmol), phenylacetylene **4** (0.5 mmol, 55 μL), K_2HPO_4 (209.1 mg, 1.2 mmol), MeCN (19 mL) and H_2O (1 mL). After 24 h, the reaction mixture was cooled to rt and filtered through a short pad of silica using EA. The filtrate was concentrated in *vacuo* before it was purified by flash chromatography (PE/EA = 15/1) on silica gel to afford **12** and **12**' (98.7 mg, 0.44 mmol, total 89% yield) as a yellow oil. The ration of **12** and **12'** was determined by ^1H NMR analysis (0.75/1 *rr*). ^1H NMR (500 MHz, $CDCl_3$) δ 7.68 (d, *J* = 10.0 Hz, 0.56H), 7.60 (s, 0.42H), 7.41-7.20 (m, 4H), 7.20-7.12 (m, 1H), 7.12-7.08 (m, 1H), 7.01 (d, *J* = 5.0 Hz, 1H), 4.57 (dd, J = 10.0, 5.0 Hz, 0.56H), 4.51 (dd, *J* = 10.0, 5.0 Hz, 0.44H), 3.26-3.17 (m, 1H), 2.66 (dd, *J* = 17.5, 2.5 Hz, 0.44H), 2.59 (dd, *J* = 20.0, 5.0 Hz, 0.56H), 2.40 (s, 1.3H), 2.01 (s, 1.7H); ^{13}C NMR (126 MHz, $CDCl_3$) δ

206.5, 205.9, 155.5, 155.3, 143.8, 143.6, 137.8, 137.1, 136.8, 136.7, 136.3, 136.3, 128.78, 128.76, 128.3, 127.5, 127.2, 126.8, 126.6, 126.5, 123.2, 120.9, 47.5, 47.1, 44.0, 43.8, 21.0, 18.3.

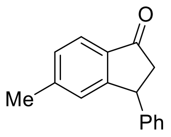

13

5-Methyl-3-phenyl-2,3-dihydro-1*H*-inden-1-one (13).[23] Prepared according to the general procedure employing Ir[dF(CF_3)ppy]$_2$(phen)PF_6 (5.5 mg, 0.005 mmol), 2-oxo-2-(*p*-tolyl)acetic acid (165.0 mg, 1.0 mmol), phenylacetylene **4** (0.5 mmol, 55 μL), K_2HPO_4 (209.7 mg, 1.2 mmol), MeCN (19 mL) and H_2O (1 mL). After 24 h, the reaction mixture was cooled to rt and filtered through a short pad of silica using EA. The filtrate was concentrated in *vacuo* before it was purified by flash chromatography (PE/EA = 15/1) on silica gel to afford **13** (88.6 mg, 0.40 mmol, 80% yield) as a yellow oil. ^{1}H NMR (500 MHz, $CDCl_3$) δ 7.69 (d, *J* = 10.0 Hz, 1H), 7.30 (dd, *J* = 15.0, 7.5 Hz, 2H), 7.25-7.18 (m, 2H), 7.11 (d, *J* = 5.0 Hz, 2H), 7.04 (s, 1H), 4.50 (dd, *J* = 10.0, 5.0 Hz, 1H), 3.19 (dd, *J* = 17.5, 7.5 Hz, 1H), 2.66 (dd, *J* = 20.0, 5.0 Hz, 1H), 2.35 (s, 3H); ^{13}C NMR (126 MHz, $CDCl_3$) δ 205.3, 158.3, 146.2, 143.7, 134.4, 129.1, 128.7, 127.5, 126.9, 126.8, 123.1, 46.9, 44.2, 21.9.

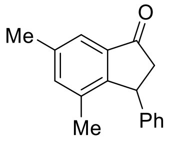

14

4,6-Dimethyl-3-phenyl-2,3-dihydro-1*H*-inden-1-one (14).[24] Prepared according to the general procedure employing Ir[dF(CF_3)ppy]$_2$(phen)PF_6 (5.5 mg, 0.005 mmol), 2-(3,5-dimethylphenyl)-2-oxoacetic acid (179.0 mg, 1.0 mmol), phenylacetylene **4** (0.5 mmol, 55 μL), K_2HPO_4 (208.1 mg, 1.2 mmol), MeCN (19 mL) and H_2O (1 mL). After 24 h, the reaction mixture was cooled to rt and filtered through a short pad of silica using EA. The filtrate was concentrated in *vacuo* before it was purified by flash chromatography (PE/EA = 15/1) on silica gel to afford **14** (93.5 mg, 0.396 mmol, 79% yield) as a yellow oil. ^{1}H NMR (500 MHz, $CDCl_3$) δ 7.48 (s, 1H), 7.28-7.22 (m, 2H), 7.21-7.16 (m, 2H), 7.01 (d, *J* = 7.5 Hz, 2H), 4.52 (dd, *J* = 8.5, 2.0 Hz, 1H), 3.21 (dd, *J* = 19.0, 8.0 Hz, 1H), 2.58 (dd, *J* = 19.0, 2.5 Hz, 1H), 2.39 (s, 3H), 1.97 (s, 3H); ^{13}C NMR (126 MHz, $CDCl_3$) δ 206.5, 153.0, 143.8, 138.2, 137.6, 137.3, 136.2, 128.7, 127.2, 126.5, 120.8, 47.8, 43.4, 20.9, 18.1.

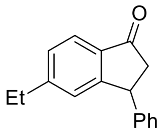

15

5-Ethyl-3-phenyl-2,3-dihydro-1*H*-inden-1-one (15). Prepared according to the general procedure employing Ir[dF(CF_3)ppy]$_2$(phen)PF_6 (5.1 mg, 0.005 mmol), 2-(4-ethylphenyl)-2-oxoacetic acid (179.6 mg, 1.0 mmol), phenylacetylene **4** (0.5 mmol, 55 μL), K_2HPO_4 (210.0 mg, 1.2 mmol), MeCN (19 mL) and H_2O (1 mL). After 24 h, the reaction mixture was cooled to rt and filtered through a short pad of silica using EA. The filtrate

was concentrated in *vacuo* before it was purified by flash chromatography (PE/EA = 15/1) on silica gel to afford **15** (91.5 mg, 0.388 mmol, 77% yield) as a yellow oil. IR *v* 2965, 2928, 1711, 1604 cm^{-1}; ^{1}H NMR (500 MHz, $CDCl_3$) δ 7.72 (d, *J* = 7.5 Hz, 1H), 7.33-7.27 (m, 2H), 7.26-7.21 (m, 2H), 7.16-7.10 (m, 2H), 7.07 (s, 1H), 4.52 (dd, *J* = 8.0, 4.0 Hz, 1H), 3.20 (dd, *J* = 19.0, 8.0 Hz, 1H), 2.72 - 2.61 (m, 3H), 1.19 (t, *J* = 7.5 Hz, 3H); ^{13}C NMR (126 MHz, $CDCl_3$) δ 205.3, 158.4, 152.4, 143.8, 134.7, 128.7, 128.0, 127.5, 126.8, 125.8, 123.2, 47.0, 44.3, 29.2, 15.2; HRMS (ESI-TOF) Calcd. for $C_{17}H_{16}ONa$ $[M+Na]^+$: 259.1099; Found 259.1099.

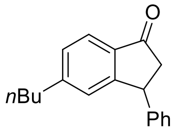

16

5-Butyl-3-phenyl-2,3-dihydro-1*H*-inden-1-one (16). Prepared according to the general procedure employing Ir[dF(CF_3)ppy]$_2$(phen)PF_6 (5.1 mg, 0.005 mmol), 2-(4-butylphenyl)-2-oxoacetic acid (206.2 mg, 1.0 mmol), phenylacetylene **4** (0.5 mmol, 55 μL), K_2HPO_4 (210.0 mg, 1.2 mmol), MeCN (19 mL) and H_2O (1 mL). After 24 h, the reaction mixture was cooled to rt and filtered through a short pad of silica using EA. The filtrate was concentrated in *vacuo* before it was purified by flash chromatography (PE/EA = 15/1) on silica gel to afford **16** (113.1 mg, 0.428 mmol, 86% yield) as a yellow oil. IR *v* 2955, 2927, 1713, 1605 cm^{-1}; ^{1}H NMR (500 MHz, $CDCl_3$) δ 7.72 (d, *J* = 8.0 Hz, 1H), 7.30 (dd, *J* = 7.2, 7.2 Hz, 2H), 7.26-7.19 (m, 2H), 7.12 (d, *J* = 7.5 Hz, 2H), 7.05 (s, 1H), 4.52 (dd, *J* = 7.5, 3.5 Hz, 1H), 3.19 (dd, *J* = 18.0, 8.0 Hz, 1H), 2.71-2.54 (m, 3H), 1.63-1.49 (m, 2H), 1.38-1.22 (m, 2H), 0.88 (t, *J* = 7.5 Hz, 3H); ^{13}C NMR (126 MHz, $CDCl_3$) δ 205.3, 158.3, 151.2, 143.8, 134.7, 128.7, 128.4, 127.5, 126.7, 126.3, 123.1, 47.0, 44.2, 36.0, 33.2, 22.2, 13.7; HRMS (ESI-TOF) Calcd. for $C_{19}H_{20}ONa$ $[M+Na]^+$: 287.1412; Found 287.1409.

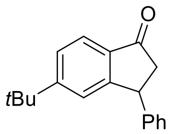

17

5-(*Tert*-butyl)-3-phenyl-2,3-dihydro-1*H*-inden-1-one (17). Prepared according to the general procedure employing Ir[dF(CF_3)ppy]$_2$(phen)PF_6 (5.1 mg, 0.005 mmol), 2-(4-(*tert*-butyl)phenyl)-2-oxoacetic acid (206.3 mg, 1.0 mmol), phenylacetylene **4** (0.5 mmol, 55 μL), K_2HPO_4 (210.0 mg, 1.2 mmol), MeCN (19 mL) and H_2O (1 mL). After 24 h, the reaction mixture was cooled to rt and filtered through a short pad of silica using EA. The filtrate was concentrated in *vacuo* before it was purified by flash chromatography (PE/EA = 15/1) on silica gel to afford **17** (123.1 mg, 0.466 mmol, 93% yield) as a yellow solid. mp = 119-120 °C. IR *v* 2964, 1710, 1605 cm^{-1}; ^{1}H NMR (500 MHz, $CDCl_3$) δ 7.75 (d, *J* = 8.5 Hz, 1H), 7.47 (dd, *J* = 8.5, 1.0 Hz, 1H), 7.33-7.27 (m, 2H), 7.26-7.20 (m, 2H), 7.13 (d, *J* = 7.0 Hz, 2H), 4.55 (dd,

J = 8.0, 4.0 Hz, 1H), 3.20 (dd, J = 19.0, 8.0 Hz, 1H), 2.65 (dd, J = 19.0, 3.5 Hz, 1H), 1.28 (s, 9H); ^{13}C NMR (126 MHz, $CDCl_3$) δ 205.4, 159.3, 157.9, 143.8, 134.5, 128.8, 127.5, 126.7, 125.6, 123.2, 122.9, 47.2, 44.4, 35.4, 31.1; HRMS (ESI-TOF) Calcd. for $C_{19}H_{20}ONa$ $[M+Na]^+$: 287.1412; Found 287.1409.

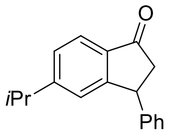

18

5-Isopropyl-3-phenyl-2,3-dihydro-1*H*-inden-1-one (18). Prepared according to the general procedure employing Ir[dF(CF_3)ppy]$_2$(phen)PF_6 (5.3 mg, 0.005 mmol), 2-(4-isopropylphenyl)-2-oxoacetic acid (206.3 mg, 1.0 mmol), phenylacetylene **4** (0.5 mmol, 55 μL), K_2HPO_4 (209.1 mg, 1.2 mmol), MeCN (19 mL) and H_2O (1 mL). After 24 h, the reaction mixture was cooled to rt and filtered through a short pad of silica using EA. The filtrate was concentrated in *vacuo* before it was purified by flash chromatography (PE/EA = 15/1) on silica gel to afford **18** (102.5 mg, 0.409 mmol, 82% yield) as a yellow solid. mp = 97-98 °C. IR *v* 2961, 2925, 1711, 1605, 700 cm^{-1}; ^{1}H NMR (500 MHz, $CDCl_3$) δ 7.74 (d, J = 8.0 Hz, 1H), 7.34-7.27 (m, 3H), 7.25-7.21 (m, 1H), 7.15 -7.11 (m, 2H), 7.10 (s, 1H), 4.53 (dd, J = 8.0, 3.5 Hz, 1H), 3.20 (dd, J = 19.0, 8.0 Hz, 1H), 2.96-2.86 (m, 1H), 2.66 (dd, J = 19.5, 4.0 Hz, 1H), 1.22 (d, J = 7.0 Hz, 3H), 1.20 (d, J = 7.0 Hz, 3H); ^{13}C NMR (126 MHz, $CDCl_3$) δ 205.4, 158.3, 157.1, 143.8, 134.9, 128.8, 127.6, 126.8, 126.5, 124.5, 123.3, 47.1, 44.4, 34.6, 23.8, 23.5; HRMS (ESI-TOF) Calcd. for $C_{18}H_{18}ONa$ [M+Na]+: 273.1255; Found 273.1251.

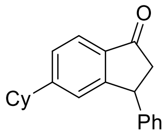

19

5-Cyclohexyl-3-phenyl-2,3-dihydro-1*H*-inden-1-one (19). Prepared according to the general procedure employing Ir[dF(CF_3)ppy]$_2$(phen)PF_6 (5.0 mg, 0.005 mmol), 2-(4-cyclohexylphenyl)-2-oxoacetic acid **S2** (229.0 mg, 0.99 mmol), phenylacetylene **4** (0.5 mmol, 55 μL), K_2HPO_4 (210.0 mg, 1.2 mmol), MeCN (19 mL) and H_2O (1 mL). After 24 h, the reaction mixture was cooled to rt and filtered through a short pad of silica using EA. The filtrate was concentrated in *vacuo* before it was purified by flash chromatography (PE/EA = 15/1) on silica gel to afford **19** (125.7 mg, 0.433 mmol, 87% yield) as a yellow solid. mp = 113 °C; IR *v* 3027, 2924, 2851, 1713, 1494 cm^{-1}; ^{1}H NMR (500 MHz, $CDCl_3$) δ 7.73 (d, J = 8.0 Hz, 1H), 7.34-7.27 (m, 2H), 7.26-7.19 (m, 2H), 7.13 (s, 1H), 7.09 (d, J = 16.0 Hz, 2H), 4.52 (dd, J = 8.0, 3.5 Hz, 1H), 3.19 (dd, J = 19.0, 8.0 Hz, 1H), 2.64 (dd, J = 19.0, 4.0 Hz, 1H), 2.57-2.46 (m, 1H), 1.89-1.75 (m, 4H), 1.71 (d, J = 12.5 Hz, 1H), 1.47-1.27 (m, 4H), 1.26-1.12 (m, 1H); ^{13}C NMR (126 MHz, $CDCl_3$) δ 205.3, 158.2, 156.1, 143.8, 134.9, 128.7, 127.5, 126.9, 126.7, 124.8, 123.2, 47.0, 45.0, 44.3, 34.2, 33.8, 26.55, 26.53, 25.8; HRMS (ESI-TOF) Calcd. for $C_{21}H_{22}ONa$ $[M+Na]^+$: 313.1568;

Found 313.1565.

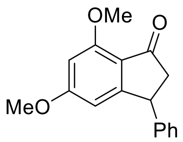

20

5,7-Dimethoxy-3-phenyl-2,3-dihydro-1*H*-inden-1-one (20). Prepared according to the general procedure employing Ir[dF(CF_3)ppy]$_2$(phen)PF_6 (5.5 mg, 0.005 mmol), 2-(2,4-dimethoxyphenyl)-2-oxoacetic acid (211.0 mg, 1.0 mmol), phenylacetylene **4** (0.5 mmol, 55 μL), K_2HPO_4 (210.0 mg, 1.2 mmol), MeCN (19 mL) and H_2O (1 mL). After 24 h, the reaction mixture was cooled to rt and filtered through a short pad of silica using EA. The filtrate was concentrated in *vacuo* before it was purified by flash chromatography (PE/EA = 2/1) on silica gel to afford **20** (125.9 mg, 0.469 mmol, 94% yield) as a yellow oil. IR *v* 2929, 2841, 1698, 1600, 1493, 1457 cm^{-1}; ^1H NMR (500 MHz, $CDCl_3$) δ 7.30 (dd, J = 7.5, 7.5 Hz, 2H), 7.25-7.19 (m, 1H), 7.13 (d, J = 7.0 Hz, 2H), 6.33 (s, 1H), 6.22 (s, 1H), 4.40 (dd, J = 8.5, 3.5 Hz, 1H), 3.94 (s, 3H), 3.75 (s, 3H), 3.15 (dd, J = 19.0, 8.5 Hz, 1H), 2.64 (dd, J = 19.0, 4.0 Hz, 1H); ^{13}C NMR (126 MHz, $CDCl_3$) δ 201.6, 167.0, 162.9, 158.9, 143.5, 128.7, 127.4, 126.7, 119.1, 101.7, 97.7, 55.7, 55.6, 47.3, 44.1; HRMS (ESI-TOF) Calcd. for $C_{17}H_{16}O_3Na$ $[M+Na]^+$: 291.0997; Found 291.0994.

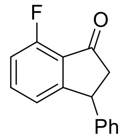

21

7-Fluoro-3-phenyl-2,3-dihydro-1*H*-inden-1-one (21).[25] Prepared according to the general procedure employing Ir[dF(CF_3)ppy]$_2$(phen)PF_6 (5.4 mg, 0.005 mmol), 2-(2-fluorophenyl)-2-oxoacetic acid (168.6 mg, 0.99 mmol), phenylacetylene **4** (0.5 mmol, 55 μL), K_2HPO_4 (210.0 mg, 1.2 mmol), MeCN (19 mL) and H_2O (1 mL). After 24 h, the reaction mixture was cooled to rt and filtered through a short pad of silica using EA. The filtrate was concentrated in *vacuo* before it was purified by flash chromatography (PE/EA = 20/1) on silica gel to afford **21** (94.1 mg, 0.416 mmol, 81% yield) as a yellow solid. ^1H NMR (500 MHz, $CDCl_3$) δ 7.56-7.49 (m, 1H), 7.36-7.29 (m, 2H), 7.29-7.23 (m, 1H), 7.16-7.11 (m, 2H), 7.06-6.98 (m, 2H), 4.57 (dd, J = 8.5, 4.0 Hz, 1H), 3.24 (dd, J = 19.5, 8.5 Hz, 1H), 2.73 (dd, J = 19.0, 4.0 Hz, 1H); ^{13}C NMR (126 MHz, $CDCl_3$) δ 202.0, 160.0 (d, J = 2.0 Hz), 158.5 (d, J = 264.7 Hz), 143.0, 136.9 (d, J = 8.2 Hz), 129.0, 127.5, 127.1, 124.5 (d, J = 12.9 Hz), 122.6 (d, J = 4.2 Hz), 114.6 (d, J = 19.2 Hz), 47.2, 44.3 (d, J = 1.1 Hz); ^{19}F NMR (471 MHz, $CDCl_3$) δ -115.02.

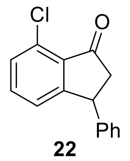
22

7-Chloro-3-phenyl-2,3-dihydro-1*H*-inden-1-one (22).[22] Prepared according to the general procedure employing Ir[dF(CF_3)ppy]$_2$(phen)PF_6 (5.2 mg, 0.005 mmol), 2-(2-chlorophenyl)-2-oxoacetic acid (183.1 mg, 0.99 mmol), phenylacetylene **4** (0.5 mmol, 55 μL), K_2HPO_4 (210.0 mg, 1.2 mmol), MeCN (19 mL) and H_2O (1 mL). After 24 h, the reaction mixture was cooled to rt and filtered through a short pad of silica using EA. The filtrate was concentrated in *vacuo* before it was purified by flash chromatography (PE/EA = 10/1) on silica gel to afford **22** (90.8 mg, 0.374 mmol, 75% yield) as a yellow solid. ^{1}H NMR (500 MHz, $CDCl_3$) δ 7.44 (t, *J* = 8.0 Hz, 1H), 7.32 (dd, *J* = 14.5, 7.5 Hz, 3H), 7.28-7.22 (m, 1H), 7.16-7.07 (m, 3H), 4.51 (dd, *J* = 8.5, 4.0 Hz, 1H), 3.24 (dd, *J* = 19.0, 8.0 Hz, 1H), 2.73 (dd, *J* = 19.0, 4.0 Hz, 1H); ^{13}C NMR (126 MHz, $CDCl_3$) δ 202.4, 160.2, 143.0, 135.2, 132.4, 131.4, 129.4, 128.9, 127.5, 127.0, 125.3, 47.3, 43.5.

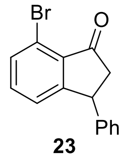
23

7-Bromo-3-phenyl-2,3-dihydro-1*H*-inden-1-one (23).[25] Prepared according to the general procedure employing Ir[dF(CF_3)ppy]$_2$(phen)PF_6 (5.3 mg, 0.005 mmol), 2-(2-bromophenyl)-2-oxoacetic acid (229.0 mg, 1.0 mmol), phenylacetylene **4** (0.5 mmol, 55 μL), K_2HPO_4 (210.0 mg, 1.2 mmol), MeCN (19 mL) and H_2O (1 mL). After 24 h, the reaction mixture was cooled to rt and filtered through a short pad of silica using EA. The filtrate was concentrated in *vacuo* before it was purified by flash chromatography (PE/EA = 20/1) on silica gel to afford **23** (109.0 mg, 0.380 mmol, 76% yield) as a yellow oil. ^{1}H NMR (500 MHz, $CDCl_3$) δ 7.55 (d, *J* = 8.0 Hz, 1H), 7.39-7.28 (m, 3H), 7.27-7.22 (m, 1H), 7.19 (d, *J* = 7.5 Hz, 1H), 7.14-7.08 (m, 2H), 4.50 (dd, *J* = 8.5, 4.5 Hz, 1H), 3.25 (dd, *J* = 19.0, 8.0 Hz, 1H), 2.74 (dd, *J* = 19.0, 4.0 Hz, 1H); ^{13}C NMR (126 MHz, $CDCl_3$) δ 202.7, 160.5, 143.0, 135.3, 133.8, 132.8, 128.9, 127.6, 127.1, 125.9, 119.1, 47.4, 43.3.

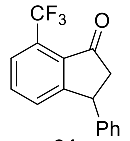
24

3-Phenyl-7-(trifluoromethyl)-2,3-dihydro-1*H*-inden-1-one (24). Prepared according to the general procedure employing Ir[dF(CF_3)ppy]$_2$(phen)PF_6 (5.3 mg, 0.005 mmol), 2-oxo-2-(2-(trifluoromethyl)phenyl)acetic acid (215.0 mg, 1.0 mmol), phenylacetylene **4** (0.5 mmol, 55 μL), K_2HPO_4 (210.0 mg, 1.2 mmol), MeCN (19 mL) and H_2O (1 mL). After 24 h, the reaction mixture was cooled to rt and filtered through a short pad of silica using EA. The filtrate was concentrated in *vacuo* before it was purified by flash chromatography (PE/EA = 10/1) on silica gel to afford **24** (60.0 mg, 0.217 mmol, 43% yield) as a yellow solid. mp = 97-

98 °C. IR *v* 2921, 1723, 1596, 1314, 1138 cm^{-1}; ^{1}H NMR (500 MHz, $CDCl_3$) δ 7.71 (d, *J* = 7.5 Hz, 1H), 7.63 (t, *J* = 7.5 Hz, 1H), 7.46 (d, *J* = 8.0 Hz, 1H), 7.33 (dd, *J* = 7.5, 7.0 Hz, 2H), 7.28 (d, *J* = 7.0 Hz, 1H), 7.13 (d, *J* = 7.0 Hz, 2H), 4.58 (dd, *J* = 8.0, 4.0 Hz, 1H), 3.28 (dd, *J* = 19.0, 8.0 Hz, 1H), 2.78 (dd, *J* = 19.0, 4.2 Hz, 1H); ^{13}C NMR (126 MHz, $CDCl_3$) δ 201.6, 160.0, 142.8, 134.3, 133.3, 130.8, 129.1, 127.6, 127.3, 126.6 (q, *J* = 34.8 Hz), 125.6 (q, *J* = 6.0 Hz), 121.6 (q, *J* = 274.2 Hz); 47.0, 44.2; ^{19}F NMR (471 MHz, $CDCl_3$) δ -61.35. HRMS (ESI-TOF) Calcd. for $C_{16}H_{11}F_3ONa$ $[M+Na]^+$: 299.0660; Found 299.0657.

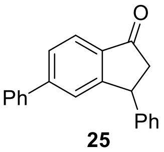

25

3,5-Diphenyl-2,3-dihydro-1*H*-inden-1-one (25).[26] Prepared according to the general procedure employing Ir[dF(CF_3)ppy]$_2$(phen)PF_6 (5.3 mg, 0.005 mmol), 2-([1,1'-biphenyl]-4-yl)-2-oxoacetic acid (224.3 mg, 1.0 mmol), phenylacetylene **4** (0.5 mmol, 55 μL), K_2HPO_4 (210.0 mg, 1.2 mmol), MeCN (19 mL) and H_2O (1 mL). After 24 h, the reaction mixture was cooled to rt and filtered through a short pad of silica using EA. The filtrate was concentrated in *vacuo* before it was purified by flash chromatography (PE/EA = 15/1) on silica gel to afford **25** (92.8 mg, 0.326 mmol, 65% yield) as a yellow solid. ^{1}H NMR (500 MHz, $CDCl_3$) δ 7.87 (d, *J* = 8.0 Hz, 1H), 7.65 (d, *J* = 8.0 Hz, 1H), 7.54 (d, *J* = 7.0 Hz, 2H), 7.45 (s, 1H), 7.41 (t, *J* = 7.0 Hz, 2H), 7.39-7.29 (m, 3H), 7.27-7.22 (m, 1H), 7.17 (d, *J* = 7.5 Hz, 2H), 4.62 (dd, *J* = 8.0, 3.5 Hz, 1H), 3.27 (dd, *J* = 19.5, 8.0 Hz, 1H), 2.74 (dd, *J* = 19.0, 4.0 Hz, 1H); ^{13}C NMR (126 MHz, $CDCl_3$) δ 205.5, 158.6, 148.1, 143.6, 140.0, 135.6, 128.92, 128.86, 128.3, 127.6, 127.4, 127.3, 127.0, 125.2, 123.8, 47.2, 44.5.

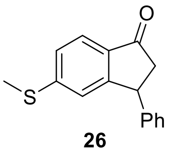

26

5-(Methylthio)-3-phenyl-2,3-dihydro-1*H*-inden-1-one (26). Prepared according to the general procedure employing Ir[dF(CF_3)ppy]$_2$(phen)PF_6 (5.5 mg, 0.005 mmol), 2-(4-(methylthio)phenyl)-2-oxoacetic acid (196.6 mg, 1.0 mmol), phenylacetylene **4** (0.5 mmol, 55 μL), K_2HPO_4 (210.0 mg, 1.2 mmol), MeCN (19 mL) and H_2O (1 mL). After 24 h, the reaction mixture was cooled to rt and filtered through a short pad of silica using EA. The filtrate was concentrated in *vacuo* before it was purified by flash chromatography (PE/EA = 15/1) on silica gel to afford **26** (89.8 mg, 0.353 mmol, 71% yield) as a yellow solid. mp = 77-78 °C. IR *v* 2955, 2921, 1704, 1587, 1063 cm^{-1}; ^{1}H NMR (500 MHz, $CDCl_3$) δ 7.67 (d, *J* = 8.0 Hz, 1H), 7.29 (dd, *J* = 8.0, 7.5 Hz, 2H), 7.25-7.18 (m, 2H), 7.11 (d, *J* = 7.0 Hz, 2H), 7.00 (s, 1H), 4.49 (dd, *J* = 8.0, 4.0 Hz, 1H), 3.18 (dd, *J* = 19.0, 8.0 Hz, 1H), 2.64 (dd, *J* = 19.0, 4.0 Hz, 1H), 2.40 (s, 3H); ^{13}C NMR (126 MHz, $CDCl_3$) δ 204.3, 158.3, 148.5, 143.3, 133.3, 128.8, 127.4, 126.8, 124.8,

123.2, 122.0, 46.7, 44.1, 14.6; HRMS (ESI-TOF) Calcd. for $C_{16}H_{14}OSNa$ [M+Na]$^+$: 277.0663; Found 277.0663.

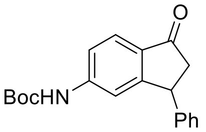

27

***Tert*-butyl (1-oxo-3-phenyl-2,3-dihydro-1*H*-inden-5-yl)carbamate (27).** Prepared according to the general procedure employing Ir[dF(CF_3)ppy]$_2$(phen)PF_6 (5.2 mg, 0.005 mmol), 2-(4-((*tert*-butoxycarbonyl)amino)phenyl)-2-oxoacetic acid **S4** (229.6 mg, 0.87 mmol), phenylacetylene **4** (0.5 mmol, 55 μL), K_2HPO_4 (210.0 mg, 1.2 mmol), MeCN (19 mL) and H_2O (1 mL). After 24 h, the reaction mixture was cooled to rt and filtered through a short pad of silica using EA. The filtrate was concentrated in *vacuo* before it was purified by flash chromatography (PE/EA = 4/1) on silica gel to afford **27** (104.1 mg, 0.322 mmol, 64% yield) as a yellow solid. mp = 171-172 °C. IR *v* 3300, 2925, 1732, 1694, 1595, 1536, 1154 cm^{-1}; ^{1}H NMR (500 MHz, $CDCl_3$) δ 7.72 (d, *J* = 8.4 Hz, 1H), 7.38 (d, *J* = 8.5 Hz, 1H), 7.32 (s, 1H), 7.31-7.25 (m, 2H), 7.24-7.18 (m, 1H), 7.10 (d, *J* = 7.0 Hz, 2H), 7.03 (s, 1H), 4.49 (dd, *J* = 8.0, 3.5 Hz, 1H), 3.18 (dd, *J* = 19.0, 8.0 Hz, 1H), 2.65 (dd, *J* = 19.0, 3.5 Hz, 1H), 1.46 (s, 9H); ^{13}C NMR (126 MHz, $CDCl_3$) δ 204.5, 159.9, 152.0, 145.0, 143.6, 131.4, 128.8, 127.5, 126.8, 124.5, 118.2, 114.8, 81.3, 46.9, 44.4, 28.1; HRMS (ESI-TOF) Calcd. for $C_{20}H_{21}NO_3Na$ [M+Na]$^+$: 346.1419; Found 346.1416.

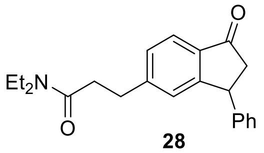

28

***N*,*N*-Diethyl-3-(1-oxo-3-phenyl-2,3-dihydro-1*H*-inden-5-yl)propanamide (28)**. Prepared according to the general procedure employing Ir[dF(CF_3)ppy]$_2$(phen)PF_6 (5.4 mg, 0.005 mmol), **S9** (278.2 mg, 1.0 mmol), phenylacetylene **4** (0.5 mmol, 55 μL), K_2HPO_4 (210.0 mg, 1.2 mmol), MeCN (19 mL) and H_2O (1 mL). After 24 h, the reaction mixture was cooled to rt and filtered through a short pad of silica using EA. The filtrate was concentrated in *vacuo* before it was purified by flash chromatography (PE/EA = 15/1) on silica gel to afford **28** (151.5 mg, 0.452 mmol, 90% yield) as a yellow oil. IR *v* 2970, 2930, 1710, 1638, 1481 cm^{-1}; ^{1}H NMR (500 MHz, $CDCl_3$) δ 7.72 (d, *J* = 7.5 Hz, 1H), 7.35-7.26 (m, 3H), 7.26-7.21 (m, 1H), 7.14-7.08 (m, 3H), 4.53 (dd, *J* = 8.0, 3.5 Hz, 1H), 3.41-3.26 (m, 2H), 3.25-3.13 (m, 3H), 3.01 (t, *J* = 7.5 Hz, 2H), 2.67 (dd, *J* = 19.0, 4.0 Hz, 1H), 2.63-2.49 (m, 2H), 1.09-1.01 (m, 6H); ^{13}C NMR (126 MHz, $CDCl_3$) δ 205.3, 170.4, 158.4, 149.8, 143.6, 134.9, 128.7, 128.5, 127.5, 126.8, 126.4, 123.3, 46.9, 44.2, 41.8, 40.2, 34.1, 31.7, 14.2, 12.9; HRMS (ESI-TOF) Calcd. for $C_{22}H_{25}NO_2Na$ [M+Na]$^+$: 358.1783; Found 358.1784.

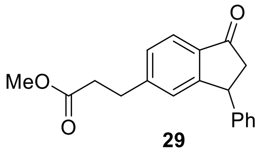

Methyl 3-(1-oxo-3-phenyl-2,3-dihydro-1*H*-inden-5-yl)propanoate (29). Prepared according to the general procedure employing Ir[dF(CF_3)ppy]$_2$(phen)PF_6 (5.3 mg, 0.005 mmol), **S6** (236.1 mg, 1.0 mmol), phenylacetylene **4** (0.5 mmol, 55 μL), K_2HPO_4 (210.0 mg, 1.2 mmol), MeCN (19 mL) and H_2O (1 mL). After 24 h, the reaction mixture was cooled to rt and filtered through a short pad of silica using EA. The filtrate was concentrated in *vacuo* before it was purified by flash chromatography (PE/EA = 4/1) on silica gel to afford **29** (111.4 mg, 0.378 mmol, 76% yield) as a yellow solid. mp = 74-76 °C. IR *v* 3027, 2951, 1711, 1606, 1435, 702 cm^{-1}; ^{1}H NMR (500 MHz, $CDCl_3$) δ 7.73 (d, *J* = 8.0 Hz, 1H), 7.34-7.20 (m, 4H), 7.11 (d, *J* = 7.0 Hz, 2H), 7.08 (s, 1H), 4.53 (dd, *J* = 7.5, 3.5 Hz, 1H), 3.59 (s, 3H), 3.20 (dd, *J* = 19.0, 8.0 Hz, 1H), 2.96 (t, *J* = 8.0 Hz, 2H), 2.67 (dd, *J* = 19.5, 4.0 Hz, 1H), 2.63-2.54 (m, 2H); ^{13}C NMR (126 MHz, $CDCl_3$) δ 205.2, 172.6, 158.4, 148.5, 143.6, 135.1, 128.8, 128.3, 127.5, 126.8, 126.3, 123.4, 51.5, 46.9, 44.2, 35.0, 31.1; HRMS (ESI-TOF) Calcd. for $C_{19}H_{18}O_3Na$ $[M+Na]^+$: 317.1154; Found 317.1151.

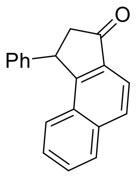

1-Phenyl-1,2-dihydro-3*H*-cyclopenta[*a*]naphthalen-3-one (30). [27] Prepared according to the general procedure employing Ir[dF(CF_3)ppy]$_2$(phen)PF_6 (5.5 mg, 0.005 mmol), 2-(naphthalen-2-yl)-2-oxoacetic acid (200.5 mg, 1.0 mmol), phenylacetylene **4** (0.5 mmol, 55 μL), K_2HPO_4 (210.0 mg, 1.2 mmol), MeCN (19 mL) and H_2O (1 mL). After 24 h, the reaction mixture was cooled to rt and filtered through a short pad of silica using EA. The filtrate was concentrated in *vacuo* before it was purified by flash chromatography (PE/EA = 15/1) on silica gel to afford **30** (35.1 mg, 0.136 mmol, 27% yield) as a yellow oil. ^{1}H NMR (500 MHz, $CDCl_3$) δ 7.92 (d, *J* = 8.5 Hz, 1H), 7.86 (dd, *J* = 30.0, 8.5 Hz, 2H), 7.72 (d, *J* = 8.0 Hz, 1H), 7.57 (t, *J* = 7.5 Hz, 1H), 7.39 (t, *J* = 8.0 Hz, 1H), 7.29-7.22 (m, 2H), 7.22-7.17 (m, 1H), 7.09 (d, *J* = 7.5 Hz, 2H), 5.00 (dd, *J* = 8.0, 2.5 Hz, 1H), 3.37 (dd, *J* = 19.0, 7.5 Hz, 1H), 2.71 (dd, *J* = 19.0, 2.5 Hz, 1H); ^{13}C NMR (126 MHz, $CDCl_3$) δ 205.8, 157.2, 144.2, 137.3, 135.4, 130.1, 129.6, 129.0, 128.97, 128.87, 127.3, 127.0, 126.9, 125.8, 119.1, 47.8, 43.7.

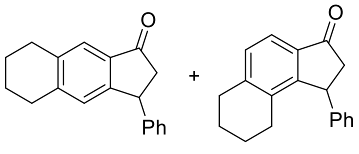

3-Phenyl-2,3,5,6,7,8-hexahydro-1*H*-cyclopenta[b]naphthalen-1-one (**31**) and **1-phenyl-1,2,6,7,8,9-hexahydro-3*H*-cyclopenta[a]naphthalen-3-one** (**31′**). Prepared

according to the general procedure employing Ir[dF(CF_3)ppy]$_2$(phen)PF_6 (5.3 mg, 0.005 mmol), α-oxocarboxylic acid **S11** (205.0 mg, 1.0 mmol), phenylacetylene **4** (0.5 mmol, 55 μL), K_2HPO_4 (210.0 mg, 1.2 mmol), MeCN (19 mL) and H_2O (1 mL). After 24 h, the reaction mixture was cooled to rt and filtered through a short pad of silica using EA. The filtrate was concentrated in *vacuo* before it was purified by flash chromatography (PE/EA = 15/1) on silica gel to afford a mixture of **31** and **31′** (108.6 mg, 0.414 mmol, 83% yield, *rr* 1/2) as a yellow oil. IR *v* 3026, 2930, 2859, 1710, 1591, 1324, 701 cm^{-1}; ^{1}H NMR (500 MHz, $CDCl_3$) δ 7.61-7.50 (m, 1H), 7.33-6.93 (m, 6H), 4.54-4.44 (m, 1H), 3.24-3.14 (m, 1H), 2.88-2.79 (m, 2H), 2.76 (t, *J* = 6.0 Hz, 0.64H), 2.65 (dd, *J* = 19.5, 4.0 Hz, 0.32H), 2.60-2.50 (m, 1.34H), 2.09-2.00 (m, 0.65H), 1.83-1.58 (m, 4H); ^{13}C NMR (126 MHz, $CDCl_3$) δ 206.3, 205.8, 156.1, 155.1, 145.9, 145.5, 144.1, 143.8, 137.4, 135.5, 135.0, 134.5, 129.8, 128.8, 127.5, 127.3, 126.8, 126.7, 126.5, 123.3, 120.2, 47.8, 47.1, 44.0, 43.6, 30.3, 30.2, 29.4, 25.9, 22.8, 22.7, 22.40, 22.36; HRMS (ESI-TOF) Calcd. for $C_{19}H_{18}ONa$ $[M+Na]^+$: 285.1255; found 285.1251.

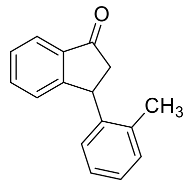

32

3-(*o*-tolyl)-2,3-dihydro-1*H*-inden-1-one (32).[21] Prepared according to the general procedure employing Ir[dF(CF_3)ppy]$_2$(phen)PF_6 (5.5 mg, 0.005 mmol), 2-Oxo-2-phenylacetate **1** (148.7 mg, 1.0 mmol), 2-methylphenylacetylene (0.5 mmol, 63 μL), K_2HPO_4 (210.0 mg, 1.2 mmol), MeCN (19 mL) and H_2O (1 mL). After 24 h, the reaction mixture was cooled to rt and filtered through a short pad of silica using EA. The filtrate was concentrated in *vacuo* before it was purified by flash chromatography (PE/EA = 15/1) on silica gel to afford **32** (68.4 mg, 0.308 mmol, 60% yield) as a yellow oil. ^{1}H NMR (500 MHz, $CDCl_3$) δ 7.83 (d, *J* = 8.0 Hz, 1H), 7.62-7.57 (m, 1H), 7.43 (dd, *J* = 7.5, 7.5 Hz, 1H), 7.30 (d, *J* = 7.5, 1H), 7.21 (d, *J* = 7.5, 1H), 7.17-7.12 (m, 1H), 7.08 (dd, *J* = 7.5, 7.5 Hz, 1H), 6.76 (d, *J* = 4.0 Hz, 1H), 4.83 (dd, *J* = 8.0, 4.0 Hz, 1H), 3.25 (dd, *J* = 19.0, 8.0 Hz, 1H), 2.57 (dd, *J* = 19.0, 4.0 Hz, 1H), 2.43 (s, 3H); ^{13}C NMR (126 MHz, $CDCl_3$) δ 206.0, 157.8, 142.0, 137.2, 135.8, 135.0, 130.6, 127.8, 126.9, 126.8, 126.6, 123.5, 45.8, 40.8, 19.9.

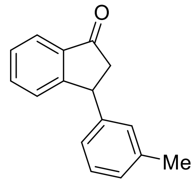

33

3-(m-tolyl)-2,3-dihydro-1*H*-inden-1-one (33).[28] Prepared according to the general procedure employing Ir[dF(CF_3)ppy]$_2$(phen)PF_6 (5.5 mg, 0.005 mmol), 2-oxo-2-phenylacetate **1** (150.2 mg, 1.0 mmol), 3-ethynyltoluene (0.5 mmol, 65 μL), K_2HPO_4 (210.0 mg, 1.2 mmol), MeCN (19 mL) and H_2O (1 mL). After 24 h, the reaction mixture was cooled to rt and filtered through a short pad of silica using EA. The filtrate was concentrated in vacuo before it was purified by flash chromatography (PE/EA = 15/1) on silica gel to afford **33** (93.7 mg, 0.421 mmol, 84% yield) as a yellow oil. ^{1}H NMR (500 MHz, $CDCl_3$) δ 7.80 (d, *J* = 7.5 Hz, 1H), 7.57-7.51 (m, 1H), 7.39 (dd, *J* = 7.5, 7.5 Hz, 1H), 7.26 (d, *J* = 8.0 Hz, 1H), 7.18 (dd, *J* = 7.5, 7.5 Hz, 1H), 7.04 (d, *J* = 7.5 Hz, 1H), 6.94-6.88 (m, 2H), 4.52 (dd, *J* = 8.0, 3.5 Hz, 1H), 3.19 (dd, *J* = 19.0, 8.0 Hz, 1H), 2.67 (dd, *J* = 19.5, 4.0 Hz, 1H), 2.29 (s, 3H); ^{13}C NMR (126 MHz, $CDCl_3$) δ 205.9, 157.9, 143.5, 138.4, 136.6, 134.9, 128.6, 128.2, 127.7, 127.6, 126.8, 124.6, 123.2, 46.7, 44.3, 21.3.

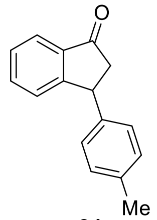

34

3-(*p*-tolyl)-2,3-dihydro-1*H*-inden-1-one (34).[28] Prepared according to the general procedure employing Ir[dF(CF_3)ppy]$_2$(phen)PF_6 (5.5 mg, 0.005 mmol), 2-oxo-2-phenylacetate **1** (149.5 mg, 1.0 mmol), 4-ethynyltoluene (0.5 mmol, 64 μL), K_2HPO_4 (209.8 mg, 1.2 mmol), MeCN (19 mL) and H_2O (1 mL). After 24 h, the reaction mixture was cooled to rt and filtered through a short pad of silica using EA. The filtrate was concentrated in *vacuo* before it was purified by flash chromatography (PE/EA = 15/1) on silica gel to afford **34** (80.6 mg, 0.363 mmol, 73% yield) as a yellow oil. ^{1}H NMR (500 MHz, $CDCl_3$) δ 7.79 (d, *J* = 8.0 Hz, 1H), 7.57-7.51 (m, 1H), 7.38 (dd, *J* = 7.5, 7.5 Hz, 1H), 7.25 (d, *J* = 7.5 Hz, 1H), 7.11 (d, *J* = 8.0 Hz, 2H), 7.00 (d, *J* = 8.0 Hz, 2H), 4.52 (dd, *J* = 8.0, 4.0 Hz, 1H), 3.19 (dd, *J* = 19.0, 8.0 Hz, 1H), 2.65 (dd, *J* = 19.0, 3.5 Hz, 1H), 2.31 (s, 3H); ^{13}C NMR (126 MHz, $CDCl_3$) δ 206.0, 158.1, 140.6, 136.6, 136.5, 134.9, 129.5, 127.7, 127.4, 126.7, 123.2, 46.8, 43.9, 20.9.

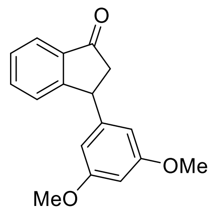

35

3-(3,5-Dimethoxyphenyl)-2,3-dihydro-1*H*-inden-1-one (35). [29] Prepared according to the general procedure employing Ir[dF(CF_3)ppy]$_2$(phen)PF_6 (5.5 mg, 0.005 mmol), 2-oxo-2-phenylacetate **1** (150.4 mg, 1.0 mmol), 3,5-dimethoxphenylacetylene (0.5 mmol, 64 μL), K_2HPO_4 (209.8 mg, 1.2 mmol), MeCN (19 mL) and H_2O (1 mL). After 24 h, the reaction mixture was cooled to rt and filtered through a short pad of silica using EA. The filtrate was concentrated in *vacuo* before it was purified by flash chromatography (PE/EA = 5/1) on silica gel to afford **35** (102.0 mg, 0.380 mmol, 76% yield) as a yellow oil. ^{1}H NMR (500 MHz, $CDCl_3$) δ 7.79 (d, *J* = 8.0 Hz, 1H), 7.59-7.53 (m, 1H), 7.40 (dd, *J* = 7.5, 7.0 Hz, 1H), 7.31 (d, *J* = 7.5 Hz, 1H), 6.34 (dd, *J* = 2.5, 2.0 Hz, 1H), 6.26 (d, *J* = 2.0 Hz, 2H), 4.50 (dd, *J* = 8.0, 3.5 Hz, 1H), 3.73 (s, 6H), 3.19 (dd, *J* = 19.5, 8.0 Hz, 1H), 2.69 (dd, *J* = 19.0, 3.5 Hz, 1H); ^{13}C NMR (126 MHz, $CDCl_3$) δ 205.7, 161.1, 157.4, 145.9, 136.6, 134.9, 127.8, 126.8, 123.3, 105.8, 98.4, 55.2, 46.4, 44.5.

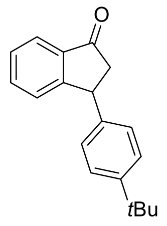

36

3-(4-(*Tert*-butyl)phenyl)-2,3-dihydro-1*H*-inden-1-one (36).[28] Prepared according to the general procedure employing Ir[dF(CF_3)ppy]$_2$(phen)PF_6 (5.5 mg, 0.005 mmol), 2-oxo-2-phenylacetate **1** (150.7 mg, 1.0 mmol), 4-*ter*-butylphenylacetylene (0.5 mmol, 64 μL), K_2HPO_4 (209.8 mg, 1.2 mmol), MeCN (19 mL) and H_2O (1 mL). After 24 h, the reaction mixture was cooled to rt and filtered through a short pad of silica using EA. The filtrate was concentrated in *vacuo* before it was purified by flash chromatography (PE/EA = 15/1) on silica gel to afford **36** (111.6 mg, 0.422 mmol, 84% yield) as a yellow oil. ^{1}H NMR (500 MHz, $CDCl_3$) δ 7.80 (d, *J* = 7.5 Hz, 1H), 7.54 (dd, *J* = 7.5, 7.0 Hz, 1H), 7.39 (dd, *J* = 7.5, 7.5 Hz, 1H), 7.32 (d, *J* = 8.5 Hz, 2H), 7.28 (d, *J* = 8.0 Hz, 1H), 7.05 (d, *J* = 8.0 Hz, 2H), 4.54 (dd, *J* = 8.0, 3.5 Hz, 1H), 3.20 (dd, *J* = 19.0, 8.0 Hz, 1H), 2.68 (dd, *J* = 19.0, 3.5 Hz, 1H), 1.30 (s, 9H); ^{13}C NMR (126 MHz, $CDCl_3$) δ 206.0, 158.0, 149.7, 140.4, 136.6, 134.9, 127.7, 127.2, 126.9, 125.7, 123.2, 46.8, 43.9, 34.4, 31.3.

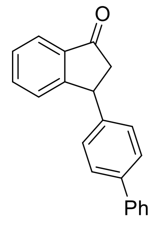
37

3-([1,1'-Biphenyl]-4-yl)-2,3-dihydro-1*H*-inden-1-one (37).[30] Prepared according to the general procedure employing Ir[dF(CF_3)ppy]$_2$(phen)PF_6 (5.5 mg, 0.005 mmol), 2-oxo-2-phenylacetate **1** (149.8 mg, 1.0 mmol), 4-ethynyl-1,1'-biphenyl (0.5 mmol, 64 μL), K_2HPO_4 (209.8 mg, 1.2 mmol), MeCN (19 mL) and H_2O (1 mL). After 24 h, the reaction mixture was cooled to rt and filtered through a short pad of silica using EA. The filtrate was concentrated in *vacuo* before it was purified by flash chromatography (PE/EA = 10/1) on silica gel to afford **37** (111.6 mg, 0.422 mmol, 84% yield) as a yellow solid. ^{1}H NMR (500 MHz, $CDCl_3$) δ 7.83 (d, *J* = 8.0 Hz, 1H), 7.63-7.49 (m, 5H), 7.42 (dd, *J* = 8.0, 7.5 Hz, 3H), 7.36-7.28 (m, 2H), 7.19 (d, *J* = 8.0 Hz, 2H), 4.61 (dd, *J* = 8.0, 3.5 Hz, 1H), 3.25 (dd, *J* = 19.0, 8.0 Hz, 1H), 2.72 (dd, *J* = 19.0, 3.5 Hz, 1H); ^{13}C NMR (126 MHz, $CDCl_3$) δ 205.9, 157.8, 142.7, 140.6, 139.9, 136.7, 135.1, 128.7, 128.0, 127.9, 127.6, 127.3, 127.0, 126.9, 123.4, 46.8, 44.1.

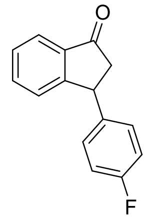
38

3-(4-Fluorophenyl)-2,3-dihydro-1*H*-inden-1-one (38).[21] Prepared according to the general procedure employing Ir[dF(CF_3)ppy]$_2$(phen)PF_6 (5.5 mg, 0.005 mmol), 2-oxo-2-phenylacetate **1** (149.9 mg, 1.0 mmol), 4-fluorophenylacetylene (0.5 mmol, 58 μL), K_2HPO_4 (209.8 mg, 1.2 mmol), MeCN (19 mL) and H_2O (1 mL). After 24 h, the reaction mixture was cooled to rt and filtered through a short pad of silica using EA. The filtrate was concentrated in *vacuo* before it was purified by flash chromatography (PE/EA = 10/1) on silica gel to afford **38** (96.0 mg, 0.424 mmol, 85% yield) as a yellow solid. ^{1}H NMR (500 MHz, $CDCl_3$) δ 7.80 (d, *J* = 7.5 Hz, 1H), 7.57 (dd, *J* = 7.5, 7.5 Hz, 1H), 7.42 (dd, *J* = 7.5, 7.5 Hz, 1H), 7.25 (d, *J* = 7.5 Hz, 1H), 7.12-7.04 (m, 2H), 7.03-6.93 (m, 2H), 4.57 (dd, *J* = 8.0, 3.5 Hz, 1H), 3.22 (dd, *J* = 19.0, 8.0 Hz, 1H), 2.63 (dd, *J* = 19.0, 3.5 Hz, 1H); ^{13}C NMR (126 MHz, $CDCl_3$) δ 205.5, 161.7 (d, *J* = 245.9 Hz), 157.5, 139.4 (d, *J* = 3.3 Hz), 136.6, 135.1, 129.0 (d, *J* = 8.0 Hz), 127.9, 126.7, 123.3, 115.6 (d, *J* = 21.4 Hz), 46.8, 43.6; ^{19}F NMR (471 MHz, $CDCl_3$) δ -115.66.

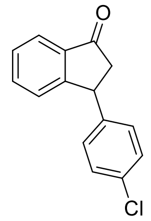
39

3-(4-Chlorophenyl)-2,3-dihydro-1*H*-inden-1-one (39).[21] Prepared according to the general procedure employing Ir[dF(CF_3)ppy]$_2$(phen)PF_6 (5.0 mg, 0.005 mmol), 2-Oxo-2-phenylacetate **1** (150.0 mg, 1.0 mmol), 4-chlorophenylacetylene (67.5 mg, 0.5 mmol), K_2HPO_4 (210.0 mg, 1.2 mmol), MeCN (19 mL) and H_2O (1 mL). After 24 h, the reaction mixture was cooled to rt and filtered through a short pad of silica using

EA. The filtrate was concentrated in *vacuo* before it was purified by flash chromatography (PE/EA = 15/1) on silica gel to afford **39** (81.9 mg, 0.337 mmol, 67% yield) as a yellow oil. ^{1}H NMR (500 MHz, $CDCl_3$) δ 7.81 (d, *J* = 7.5 Hz, 1H), 7.58 (dd, *J* = 7.5, 7.0 Hz, 1H), 7.42 (dd, *J* = 7.5, 7.5 Hz, 1H), 7.31-7.21 (m, 3H), 7.05 (d, *J* = 8.5 Hz, 2H), 4.56 (dd, *J* = 8.0, 3.5 Hz, 1H), 3.22 (dd, *J* = 19.0, 8.0 Hz, 1H), 2.62 (dd, *J* = 19.5, 3.8 Hz, 1H); ^{13}C NMR (126 MHz, $CDCl_3$) δ 205.4, 157.2, 142.1, 136.6, 135.1, 132.7, 129.0, 128.9, 128.0, 126.7, 123.4, 46.6, 43.7.

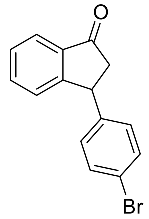

40

3-(4-Bromophenyl)-2,3-dihydro-1*H*-inden-1-one (40).[29] Prepared according to the general procedure employing Ir[dF(CF_3)ppy]$_2$(phen)PF_6 (5.0 mg, 0.005 mmol), 2-oxo-2-phenylacetate **1** (150.0 mg, 1.0 mmol), 4-bromophenylacetylene (94.4 mg, 0.521 mmol), K_2HPO_4 (210.0 mg, 1.2 mmol), MeCN (19 mL) and H_2O (1 mL). After 24 h, the reaction mixture was cooled to rt and filtered through a short pad of silica using EA. The filtrate was concentrated in vacuo before it was purified by flash chromatography (PE/EA = 15/1) on silica gel to afford **40** (107.9 mg, 0.376 mmol, 75% yield) as a yellow oil. ^{1}H NMR (500 MHz, $CDCl_3$) δ 7.80 (d, *J* = 7.5 Hz, 1H), 7.57 (dd, *J* = 7.5, 7.5 Hz, 1H), 7.48-7.36 (m, 3H), 7.24 (d, *J* = 7.5 Hz, 1H), 7.00 (d, *J* = 8.5 Hz, 2H), 4.54 (dd, *J* = 8.0, 3.5 Hz, 1H), 3.22 (dd, *J* = 19.0, 8.0 Hz, 1H), 2.62 (dd, *J* = 19.5, 4.0 Hz, 1H); ^{13}C NMR (126 MHz, $CDCl_3$) δ 205.3, 157.1, 142.6, 136.6, 135.1, 131.9, 129.3, 128.0, 126.6, 123.4, 120.7, 46.5, 43.8.

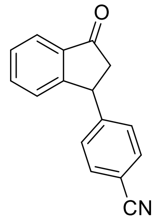

41

4-(3-Oxo-2,3-dihydro-1*H*-inden-1-yl)benzonitrile (41).[29] Prepared according to the general procedure employing Ir[dF(CF_3)ppy]$_2$(phen)PF_6 (5.0 mg, 0.005 mmol), 2-oxo-2-phenylacetate **1** (150.0 mg, 1.0 mmol), 4-ethynylbenzonitrile (63.9 mg, 0.5 mmol), K_2HPO_4 (209.4 mg, 1.2 mmol), MeCN (19 mL) and H_2O (1 mL). After 24 h, the reaction mixture was cooled to rt and filtered through a short pad of silica using EA. The filtrate was concentrated in *vacuo* before it was purified by flash chromatography (PE/EA = 15/1) on silica gel to afford **41** (80.9 mg, 0.347 mmol, 69% yield) as a yellow oil. ^{1}H NMR (500 MHz, $CDCl_3$) δ 7.83 (d, *J* = 7.5 Hz, 1H), 7.67-7.58 (m, 3H), 7.47 (dd, *J* = 8.0, 7.0 Hz, 1H), 7.28 -7.22 (m, 3H), 4.67 (dd, *J* = 8.5, 4.0 Hz, 1H), 3.27 (dd, *J* = 19.5, 8.5 Hz, 1H), 2.64 (dd, *J* = 19.0, 4.0 Hz, 1H); ^{13}C NMR (126 MHz, $CDCl_3$) δ 204.5, 156.2, 149.0, 136.6, 135.3, 132.6, 128.4, 128.3, 126.6, 123.6, 118.4, 110.9, 46.2, 44.2.

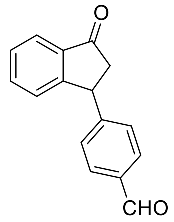

42

4-(3-Oxo-2,3-dihydro-1*H*-inden-1-yl)benzaldehyde (42). Prepared according to the general procedure employing Ir[dF(CF_3)ppy]$_2$(phen)PF_6 (5.0 mg, 0.005 mmol), 2-oxo-2-phenylacetate **1** (150.0 mg, 1.0 mmol), 4-ethynylbenzaldehyde (66.1 mg, 0.5 mmol), K_2HPO_4 (209.4 mg, 1.2 mmol), MeCN (19 mL) and H_2O (1 mL). After 24 h, the reaction mixture was cooled to rt and filtered through a short pad of silica using EA. The filtrate was concentrated in *vacuo* before it was purified by flash chromatography (PE/EA = 15/1) on silica gel to afford **42** (72.1 mg, 0.305 mmol, 61% yield) as a yellow oil. IR *v* 2955, 2922, 1707, 1603, 1463 cm^{-1}; ^{1}H NMR (500 MHz, $CDCl_3$) δ 9.99 (s, 1H), 7.84 (d, *J* = 8.0 Hz, 3H), 7.64-7.57 (m, 1H), 7.46 (dd, *J* = 7.5, 7.5 Hz, 1H), 7.31 (d, *J* = 8.0 Hz, 2H), 7.26 (d, *J* = 8.0 Hz, 1H), 4.68 (dd, *J* = 8.5, 4.0 Hz, 1H), 3.28 (dd, *J* = 19.0, 8.0 Hz, 1H), 2.69 (dd, *J* = 19.5, 4.0 Hz, 1H); ^{13}C NMR (126 MHz, $CDCl_3$) δ 205.0, 191.6, 156.7, 150.6, 136.7, 135.34, 135.30, 130.4, 128.33, 128.29, 126.7, 123.7, 46.4, 44.5; HRMS (ESI-TOF) Calcd. for $C_{16}H_{12}O_2Na$ [M+Na]$^+$: 259.0735; Found 259.0731.

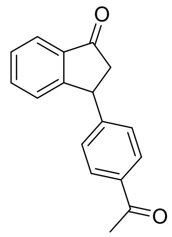

43

3-(4-Acetylphenyl)-2,3-dihydro-1*H*-inden-1-one (43). Prepared according to the general procedure employing Ir[dF(CF_3)ppy]$_2$(phen)PF_6 (5.1 mg, 0.005 mmol), 2-oxo-2-phenylacetate **1** (150.1 mg, 1.0 mmol), 4-ethynylacetophenone (72.3 mg, 0.5 mmol), K_2HPO_4 (209.4 mg, 1.2 mmol), MeCN (19 mL) and H_2O (1 mL). After 24 h, the reaction mixture was cooled to rt and filtered through a short pad of silica using EA. The filtrate was concentrated in *vacuo* before it was purified by flash chromatography (PE/EA = 3/1) on silica gel to afford **43** (94.1 mg, 0.376 mmol, 75% yield) as a yellow solid. mp = 116-117 °C. IR *v* 2955, 2922, 1711, 1679, 1267 cm^{-1}; ^{1}H NMR (500 MHz, $CDCl_3$) δ 7.91 (d, *J* = 8.5 Hz, 2H), 7.83 (d, *J* = 8.0 Hz, 1H), 7.62-7.56 (m, 1H), 7.45 (t, *J* = 7.5 Hz, 1H), 7.27-7.20 (m, 3H), 4.66 (dd, *J* = 8.0, 3.5 Hz, 1H), 3.25 (dd, *J* = 19.5, 8.0 Hz, 1H), 2.67 (dd, *J* = 19.0, 4.0 Hz, 1H), 2.58 (s, 3H); ^{13}C NMR (126 MHz, $CDCl_3$) δ 205.1, 197.4, 156.9, 149.0, 136.7, 135.9, 135.2, 129.0, 128.1, 127.8, 126.7, 123.5, 46.3, 44.2, 26.5; HRMS (ESI-TOF) Calcd. for $C_{17}H_{14}O_2Na$ [M+Na]$^+$: 273.0891; Found 273.0888.

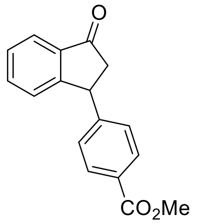

44

Methyl 4-(3-oxo-2,3-dihydro-1*H*-inden-1-yl)benzoate (44).[28] Prepared according to the general procedure employing Ir[dF(CF_3)ppy]$_2$(phen)PF_6 (5.1 mg, 0.005 mmol), 2-oxo-2-phenylacetate **1** (150.1 mg, 1.0 mmol), methyl 4-ethynylbenzoate (80.0 mg, 0.5 mmol), K_2HPO_4 (209.4 mg, 1.2 mmol), MeCN (19 mL) and H_2O (1 mL). After 24 h, the reaction mixture was cooled to rt and filtered through a short pad of silica using EA. The filtrate was concentrated in *vacuo* before it was purified by flash chromatography (PE/EA = 5/1) on silica gel to afford **44** (77.4 mg, 0.291 mmol, 58% yield) as a yellow oil. ^{1}H NMR (500 MHz, $CDCl_3$) δ 7.98 (d, *J* = 8.0 Hz, 2H), 7.82 (d, *J* = 8.0 Hz, 1H), 7.58 (t, *J* = 7.5 Hz, 1H), 7.47-7.40 (m, 1H), 7.24 (d, *J* = 7.5 Hz, 1H), 7.20 (d, *J* = 8.0 Hz, 2H), 4.64 (dd, *J* = 8.5, 4.0 Hz, 1H), 3.90 (s, 3H), 3.25 (dd, *J* = 19.0, 8.0 Hz, 1H), 2.67 (dd, *J* = 19.0, 3.5 Hz, 1H); ^{13}C NMR (126 MHz, $CDCl_3$) δ 205.2, 166.6, 156.9, 148.8, 136.6, 135.1, 130.1, 128.9, 128.0, 127.6, 126.6, 123.5, 52.0, 46.3, 44.2.

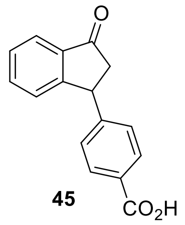

45

4-(3-Oxo-2,3-dihydro-1*H*-inden-1-yl)benzoic acid (45). Prepared according to the modified procedure employing Ir[dF(CF_3)ppy]$_2$(phen)PF_6 (5.3 mg, 0.005 mmol), 2-oxo-2-phenylacetate **1** (150.0 mg, 1.0 mmol), 4-ethynylbenzoic acid (73.6 mg, 0.5 mmol), K_2HPO_4 (296.0 mg, 1.7 mmol), MeCN (19 mL) and H_2O (1 mL). After 24 h, the reaction mixture was cooled to rt and filtered through a short pad of silica using EA. The filtrate was concentrated in *vacuo* to afford the crude product **45**. The crude **45** was dissolved in methanol (6 mL) with H_2SO_4 (Conc.) (0.16 mL). The mixture was stirred at 80 °C for 6 h. After cooled to room temperature, the reaction mixture was extracted with ethyl acetate. The combined organic layers were washed by Sat.$NaHCO_3$ and dried over anhydrous Na_2SO_4. After filtration, the filtrate was evaporated in *vacuo* and purified by column chromatography (PE/EA = 5/1) on silica gel to afford the corresponding ester **44** (90.4 mg, 0.339 mmol，68% yield over two steps) as a white solid.

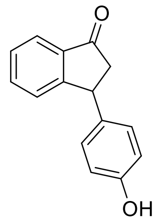

46

3-(4-Hydroxyphenyl)-2,3-dihydro-1*H*-inden-1-one (46).[31] Prepared according to the general procedure employing Ir[dF(CF_3)ppy]$_2$(phen)PF_6 (5.1 mg, 0.005 mmol), 2-oxo-2-phenylacetate **1** (150.0 mg, 1.0 mmol), 4-ethynylphenol (59.0 mg, 0.5 mmol), K_2HPO_4 (209.4 mg, 1.2 mmol), MeCN (19 mL) and H_2O (1 mL). After 24 h, the reaction mixture was cooled to rt and filtered through a short pad of silica using EA. The filtrate was concentrated in *vacuo* before it was purified by flash chromatography

(PE/EA = 2/1) on silica gel to afford **46** (59.0 mg, 0.263 mmol, 53% yield) as a brown solid. ^{1}H NMR (500 MHz, DMSO) δ 9.28 (s, 1H), 7.67 (d, *J* = 7.5 Hz, 1H), 7.62 (dd, *J* = 7.5, 7.5 Hz, 1H), 7.43 (dd, *J* = 7.5, 7.0 Hz, 1H), 7.24 (d, *J* = 7.5 Hz, 1H), 6.94 (d, *J* = 8.5 Hz, 2H), 6.69 (d, *J* = 8.5 Hz, 2H), 4.54 (dd, *J* = 8.0, 3.5 Hz, 1H), 3.16 (dd, *J* = 19.0, 8.0 Hz, 1H), 2.52 (dd, *J* = 19.0, 3.5 Hz, 1H); ^{13}C NMR (126 MHz, DMSO) δ 205.4, 158.5, 156.0, 136.0, 135.0, 133.9, 128.4, 127.7, 126.7, 122.6, 115.5, 46.2, 42.8.

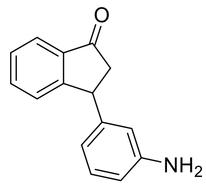

47

3-(3-Aminophenyl)-2,3-dihydro-1*H*-inden-1-one (47). Prepared according to the general procedure employing Ir[dF(CF$_3$)ppy]$_2$(phen)PF$_6$ (5.1 mg, 0.005 mmol), 2-oxo-2-phenylacetate **1** (150.0 mg, 1.0 mmol), 3-aminophenylacetylene (57 μL, 0.5 mmol), K_2HPO_4 (209.4 mg, 1.2 mmol), MeCN (19 mL) and H_2O (1 mL). After 24 h, the reaction mixture was cooled to rt and filtered through a short pad of silica using EA. The filtrate was concentrated in *vacuo* before it was purified by flash chromatography (PE/EA = 2/1) on silica gel to afford **47** (48.1 mg, 0.216 mmol, 43% yield) as a yellow solid. mp = 157-158 °C. IR *v* 2921, 1704, 1599, 1462 cm^{-1}; ^{1}H NMR (500 MHz, $CDCl_3$) δ 7.79 (d, *J* = 7.5 Hz, 1H), 7.60-7.54 (m, 1H), 7.40 (dd, *J* = 7.5, 7.5 Hz, 1H), 7.31 (d, *J* = 8.0 Hz, 1H), 7.09 (t, *J* = 8.0, 7.5 Hz, 1H), 6.58-6.52 (m, 2H), 6.39 (dd, *J* = 2.0, 1.5 Hz, 1H), 4.47 (dd, *J* = 8.0, 3.5 Hz, 1H), 3.18 (dd, *J* = 19.5, 8.0 Hz, 1H), 2.68 (dd, *J* = 19.5, 4.0 Hz, 1H); ^{13}C NMR (126 MHz, $CDCl_3$) δ 206.2, 158.0, 146.9, 144.9, 136.7, 135.0, 129.8, 127.8, 126.9, 123.3, 118.0, 114.0, 113.7, 46.7, 44.4; HRMS (ESI-TOF) Calcd. for $C_{15}H_{14}NONa$ [M+Na]$^+$: 246.0895; Found 246.0891.

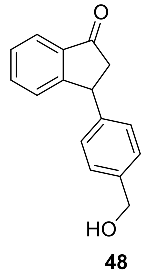

48

3-(4-(Hydroxymethyl)phenyl)-2,3-dihydro-1*H*-inden-1-one (48). Prepared according to the general procedure employing Ir[dF(CF$_3$)ppy]$_2$(phen)PF$_6$ (5.1 mg, 0.005 mmol), 2-oxo-2-phenylacetate **1** (150.0 mg, 1.0 mmol), 4-ethynylbenzyl alcohol (66.1 mg, 0.5 mmol), K_2HPO_4 (209.4 mg, 1.2 mmol), MeCN (19 mL) and H_2O (1 mL). After 24 h, the reaction mixture was cooled to rt and filtered through a short pad of silica using EA. The filtrate was concentrated in *vacuo* before it was purified by flash chromatography (PE/EA = 2/1) on silica gel to afford **48** (84.0 mg, 0.353 mmol, 71% yield) as a yellow solid. mp = 91-92 °C. IR *v* 3401,2955, 2924, 1600, 1704, 1462 cm^{-1}; ^{1}H NMR (500 MHz, $CDCl_3$) δ 7.80 (d, *J* = 8.0 Hz, 1H), 7.55 (dd, *J* = 7.0, 7.0 Hz, 1H), 7.40 (dd, *J* = 7.5, 7.5 Hz, 1H),

7.30 (d, J = 8.0 Hz, 2H), 7.24 (d, J = 8.0 Hz, 1H), 7.09 (d, J = 8.0 Hz, 2H), 4.65 (s, 2H), 4.56 (dd, J = 8.0, 3.5 Hz, 1H), 3.19 (dd, J = 19.5, 8.0 Hz, 1H), 2.63 (dd, J = 19.5, 3.5 Hz, 1H), 2.41 (s, 1H); ^{13}C NMR (126 MHz, $CDCl_3$) δ 206.1, 157.8, 142.9, 139.7, 136.6, 135.1, 127.8, 127.7, 127.5, 126.7, 123.4, 64.7, 46.7, 44.1; HRMS (ESI-TOF) Calcd. for $C_{16}H_{14}O_2Na$ $[M+Na]^+$: 261.0891; Found 261.0886.

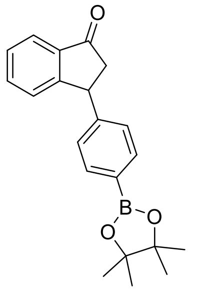
49

3-(4-(4,4,5,5-Tetramethyl-1,3,2-dioxaborolan-2-yl)phenyl)-2,3-dihydro-1*H*-inden-1-one (49). Prepared according to the general procedure employing Ir[dF(CF_3)ppy]$_2$(phen)PF_6 (5.1 mg, 0.005 mmol), 2-oxo-2-phenylacetate **1** (150.2 mg, 1.0 mmol), 4-ethynylbenzeneboronic acid pinacol ester (123.5 mg, 0.5 mmol), K_2HPO_4 (209.4 mg, 1.2 mmol), MeCN (19 mL) and H_2O (1 mL). After 24 h, the reaction mixture was cooled to rt and filtered through a short pad of silica using EA. The filtrate was concentrated in *vacuo* before it was purified by flash chromatography (PE/EA = 10/1) on silica gel to afford **49 (**121.1 mg, 0.362 mmol, 73% yield) as a yellow solid. mp = 142-143 °C. IR *v* 2978, 2928, 1714, 1610, 1144, 1089 cm^{-1}; ^{1}H NMR (500 MHz, $CDCl_3$) δ 7.81 (d, J = 7.5 Hz, 1H), 7.77 (d, J = 7.5 Hz, 2H), 7.54 (dd, J = 7.5, 7.0 Hz, 1H), 7.40 (dd, J = 7.5, 7.5 Hz, 1H), 7.23 (d, J = 7.5 Hz, 1H), 7.13 (d, J = 7.5 Hz, 2H), 4.58 (dd, J = 7.5, 3.5 Hz, 1H), 3.22 (dd, J = 19.0, 8.0 Hz, 1H), 2.69 (dd, J = 19.0, 3.5 Hz, 1H), 1.33 (s, 12H); ^{13}C NMR (126 MHz, $CDCl_3$) δ 205.7, 157.6, 146.7, 136.7, 135.3, 135.0, 127.8, 127.0, 126.8, 123.3, 83.7, 46.6, 44.5, 24.8, 24.7; HRMS (ESI-TOF) Calcd. for $C_{21}H_{23}BO_3Na$ $[M+Na]^+$:357.1638; Found 357.1640.

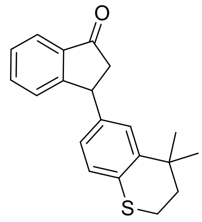
50

3-(4,4-Dimethylthiochroman-6-yl)-2,3-dihydro-1*H*-inden-1-one (50). Prepared according to the general procedure employing Ir[dF(CF_3)ppy]$_2$(phen)PF_6 (5.1 mg, 0.005 mmol), 2-oxo-2-phenylacetate **1** (150.5 mg, 1.0 mmol), 6-ethynyl-4,4-dimethylthiochroman (99.5 mg, 0.492 mmol), K_2HPO_4 (210.0 mg, 1.2 mmol), MeCN (19 mL) and H_2O (1 mL). After 24 h, the reaction mixture was cooled to rt and filtered through a short pad of silica using EA. The filtrate was concentrated in *vacuo* before it was purified by flash chromatography (PE/EA = 15/1) on silica gel to afford **50 (**94.8 mg, 0.307 mmol, 63% yield) as a yellow solid. mp = 94-95 °C. IR *v* 2960, 1711, 1604, 1475, 1065 cm^{-1}; ^{1}H NMR (500 MHz, $CDCl_3$) δ 7.79 (d, J = 8.0 Hz, 1H), 7.56 (dd, J = 7.0, 7.0 Hz, 1H), 7.40 (dd, J = 7.5, 7.5 Hz, 1H), 7.27 (d, J = 6.5 Hz, 1H), 7.16 (d, J = 1.5

Hz, 1H), 7.00 (d, J = 8.0 Hz, 1H), 6.71 (dd, J = 8.5, 2.0 Hz, 1H), 4.49 (dd, J = 8.0, 4.0 Hz, 1H), 3.20 (dd, J = 19.0, 8.0 Hz, 1H), 3.06- 2.96 (m, 2H), 2.65 (dd, J = 19.5, 4.0 Hz, 1H), 1.98-1.86 (m, 2H), 1.31 (s, 3H), 1.26 (s, 3H); ^{13}C NMR (126 MHz, $CDCl_3$) δ 206.0, 157.9, 142.3, 139.2, 136.6, 134.9, 130.3, 127.7, 127.0, 126.7, 125.9, 124.9, 123.3, 46.8, 44.1, 37.6, 33.0, 30.2, 30.1, 22.9; HRMS (ESI-TOF) Calcd. for $C_{20}H_{20}OSNa$ $[M+Na]^+$: 331.1133; Found 331.1130.

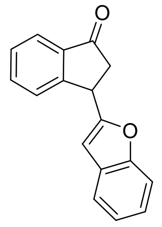

51

3-(Benzofuran-2-yl)-2,3-dihydro-1*H*-inden-1-one (51). Prepared according to the general procedure employing Ir[dF(CF_3)ppy]$_2$(phen)PF_6 (5.2 mg, 0.005 mmol), 2-oxo-2-phenylacetate **1** (150.6mg, 1.0 mmol), alkyne **S16** (71.3 mg, 0.5 mmol), K_2HPO_4 (210.0 mg, 1.2 mmol), MeCN (19 mL) and H_2O (1 mL). After 24 h, the reaction mixture was cooled to rt and filtered through a short pad of silica using EA. The filtrate was concentrated in *vacuo* before it was purified by flash chromatography (PE/EA = 15/1) on silica gel to afford **51 (**57.6 mg, 0.232 mmol, 46% yield) as a yellow solid. mp = 92-93 °C. IR v 2955, 2923, 1714, 1600, 1454 cm^{-1}; ^1H NMR (500 MHz, $CDCl_3$) δ 7.83 (d, J = 7.5 Hz, 1H), 7.64-7.60 (m, 1H), 7.59-7.54 (m, 1H), 7.51-7.43 (m, 2H), 7.41-7.37 (m, 1H), 7.24-7.16 (m, 2H), 6.50 (s, 1H), 4.80 (dd, J = 8.0, 4.0 Hz, 1H), 3.19 (dd, J = 19.0, 8.0 Hz, 1H), 2.99 (dd, J = 19.0, 4.0 Hz, 1H); ^{13}C NMR (126 MHz, $CDCl_3$) δ 204.4, 158.1, 155.0, 153.9, 136.5, 135.0, 128.5, 128.2, 126.6, 124.0, 123.8, 122.8, 120.7, 111.0, 102.9, 42.6, 38.1; HRMS (ESI-TOF) Calcd. for $C_{17}H_{13}O_2$ $[M+H]^+$: 249.0916; Found 249.0918.

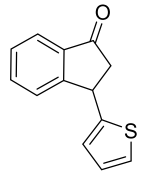

52

3-(Thiophen-2-yl)-2,3-dihydro-1*H*-inden-1-one (52).[21] Prepared according to the general procedure employing Ir[dF(CF_3)ppy]$_2$(phen)PF_6 (5.2 mg, 0.005 mmol), 2-oxo-2-phenylacetate **1** (150.6mg, 1.0 mmol), 2-ethynylthiophene (54.0 mg, 0.5 mmol), K_2HPO_4 (210.0 mg, 1.2 mmol), MeCN (19 mL) and H_2O (1 mL). After 24 h, the reaction mixture was cooled to rt and filtered through a short pad of silica using EA. The filtrate was concentrated in *vacuo* before it was purified by flash chromatography (PE/EA = 10/1) on silica gel to afford **52 (**78.1 mg, 0.365 mmol, 73% yield) as a yellow oil. ^1H NMR (500 MHz, $CDCl_3$) δ 7.79 (d, J = 7.5 Hz, 1H), 7.63- 7.57 (m, 1H), 7.48-7.39 (m, 2H), 7.18 (dd, J = 5.0, 1.0 Hz, 1H), 6.96-6.92 (m, 1H), 6.88-6.85 (m, 1H), 4.88 (dd, J = 8.0, 4.0 Hz, 1H), 3.25 (dd, J = 19.0, 8.0 Hz, 1H), 2.78 (dd, J = 19.0, 4.0 Hz, 1H); ^{13}C NMR (126 MHz, $CDCl_3$) δ 204.7, 156.6, 146.7, 136.0, 135.0, 128.2, 126.9, 126.6, 124.6, 124.2, 123.4, 47.1, 39.3.

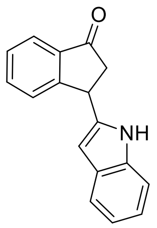

53

3-(1*H*-indol-2-yl)-2,3-dihydro-1*H*-inden-1-one (53).[29] Prepared according to the general procedure employing Ir[dF(CF_3)ppy]$_2$(phen)PF_6 (5.1 mg, 0.005 mmol), 2-oxo-2-phenylacetate **1** (106.4 mg, 0.709 mmol), alkyne **S19** (48.8 mg, 0.345 mmol), K_2HPO_4 (210.0 mg, 1.2 mmol), MeCN (13 mL) and H_2O (0.7 mL). After 24 h, the reaction mixture was cooled to rt and filtered through a short pad of silica using EA. The filtrate was concentrated in *vacuo* before it was purified by flash chromatography (PE/EA = 3/1) on silica gel to afford **53 (**57.8 mg, 0.234 mmol, 68% yield) as a yellow solid. ^{1}H NMR (500 MHz, $CDCl_3$) δ 8.32 (s, 1H), 7.71 (d, *J* = 7.5 Hz, 1H), 7.61-7.49 (m, 2H), 7.41 (d, *J* = 7.5 Hz, 1H), 7.37 (dd, *J* = 7.5, 7.5 Hz, 1H), 7.24 (d, *J* = 8.0 Hz, 1H), 7.15-7.04 (m, 2H), 6.34 (s, 1H), 4.75 (dd, *J* = 8.0, 3.5 Hz, 1H), 3.16 (dd, *J* = 19.5, 8.5 Hz, 1H), 2.77 (dd, *J* = 19.0, 3.5 Hz, 1H); ^{13}C NMR (126 MHz, $CDCl_3$) δ 205.4, 155.4, 139.5, 136.5, 136.4, 135.2, 128.4, 128.2, 126.8, 123.5, 121.8, 120.2, 119.9, 110.8, 100.8, 44.7, 37.9.

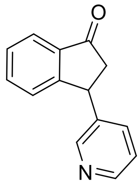

54

3-(Pyridin-3-yl)-2,3-dihydro-1*H*-inden-1-one (54). Prepared according to the general procedure employing Ir[dF(CF_3)ppy]$_2$(phen)PF_6 (5.2 mg, 0.005 mmol), 2-oxo-2-phenylacetate **1** (150.6mg, 1.0 mmol), 3-ethynylpyridine (51.0 mg, 0.5 mmol), K_2HPO_4 (210.0 mg, 1.2 mmol), MeCN (19 mL) and H_2O (1 mL). After 24 h, the reaction mixture was cooled to rt and filtered through a short pad of silica using EA. The filtrate was concentrated in *vacuo* before it was purified by flash chromatography (PE/EA = 2/1) on silica gel to afford **54 (**50.8 mg, 0.243 mmol, 49% yield) as a yellow oil. IR *v* 3031, 2923, 1713, 1602, 1478, 1285 cm^{-1}; ^{1}H NMR (500 MHz, $CDCl_3$) δ 8.52 (d, *J* = 4.0 Hz, 2H), 7.83 (d, *J* = 7.5 Hz, 1H), 7.66-7.55 (m, 1H), 7.46 (dd, *J* = 7.5, 7.5 Hz, 1H), 7.35 (d, *J* = 7.5 Hz, 1H), 7.28-7.21 (m, 2H), 4.62 (dd, *J* = 8.0, 3.5 Hz, 1H), 3.27 (dd, *J* = 19.0, 8.0 Hz, 1H), 2.66 (dd, *J* = 19.0, 3.5 Hz, 1H); ^{13}C NMR (126 MHz, $CDCl_3$) δ 204.8, 156.5, 149.2, 148.5, 139.1, 136.7, 135.3, 134.7, 128.2, 126.6, 123.9, 123.6, 46.3, 41.7; HRMS (ESI-TOF) Calcd. for $C_{14}H_{11}NONa$ [M+Na]$^+$: 232.0738; Found 232.0734.

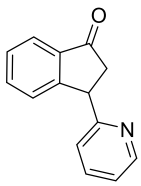

55

3-(Pyridin-2-yl)-2,3-dihydro-1*H*-inden-1-one (55). Prepared according to the general procedure employing Ir[dF(CF_3)ppy]$_2$(phen)PF_6 (5.2 mg, 0.005 mmol), 2-oxo-2-phenylacetate **1** (150.1 mg, 1.0 mmol), 2-ethynylpyridine (51.3 mg, 0.5 mmol), K_2HPO_4 (210.0 mg, 1.2 mmol), MeCN (19 mL) and H_2O (1 mL). After 24 h, the

reaction mixture was cooled to rt and filtered through a short pad of silica using EA. The filtrate was concentrated in *vacuo* before it was purified by flash chromatography (PE/EA = 2/1) on silica gel to afford **55** **(**83.4 mg, 0.399 mmol, 80% yield) as a yellow solid. mp = 100-101 °C. IR *v* 3008, 2920, 1713, 1601, 1471, 756 cm^{-1}; ^{1}H NMR (500 MHz, $CDCl_3$) δ 8.55 (d, *J* = 5.0 Hz, 1H), 7.81 (d, *J* = 7.5 Hz, 1H), 7.65 (td, *J* = 7.5, 2.0 Hz, 1H), 7.56 (td, *J* = 8.5, 1.0 Hz, 1H), 7.41 (dd, *J* = 7.5, 7.0 Hz, 1H), 7.30 (d, *J* = 7.5 Hz, 1H), 7.20-7.14 (m, 2H), 4.77 (dd, *J* = 8.0, 4.0 Hz, 1H), 3.20 (dd, *J* = 19.0, 8.0 Hz, 1H), 3.00 (dd, *J* = 19.0, 4.0 Hz, 1H); ^{13}C NMR (126 MHz, $CDCl_3$) δ 205.6, 162.0, 156.5, 149.8, 136.8, 136.6, 134.8, 127.9, 126.4, 123.6, 122.0, 121.9, 46.4, 44.4 ; HRMS (ESI-TOF) Calcd. for $C_{14}H_{11}NONa$ $[M+Na]^+$: 232.0738; Found 232.0734.

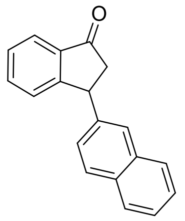

56

3-(Naphthalen-2-yl)-2,3-dihydro-1*H*-inden-1-one (56).[28] Prepared according to the general procedure employing Ir[dF(CF_3)ppy]$_2$(phen)PF_6 (5.1 mg, 0.005 mmol), 2-oxo-2-phenylacetate **1** (147.5 mg, 0.983 mmol), 2-ethynylnaphthalene (76.4 mg, 0.5 mmol), K_2HPO_4 (210.0 mg, 1.2 mmol), MeCN (19 mL) and H_2O (1 mL). After 24 h, the reaction mixture was cooled to rt and filtered through a short pad of silica using EA. The filtrate was concentrated in *vacuo* before it was purified by flash chromatography (PE/EA = 15/1) on silica gel to afford **56** **(**89.2 mg, 0.345 mmol, 69% yield) as a white solid. ^{1}H NMR (500 MHz, $CDCl_3$) δ 7.85 (d, *J* = 7.5 Hz, 1H), 7.82-7.72 (m, 3H), 7.65 (s, 1H), 7.55 (dd, *J* = 7.0, 7.0 Hz, 1H), 7.50-7.38 (m, 3H), 7.27 (d, *J* = 7.5 Hz, 1H), 7.12 (d, *J* = 8.5 Hz, 1H), 4.78-4.66 (m, 1H), 3.28 (dd, *J* = 19.0, 8.0 Hz, 1H), 2.77 (dd, *J* = 19.5, 2.5 Hz, 1H); ^{13}C NMR (126 MHz, $CDCl_3$) δ 205.9, 157.8, 140.8, 136.8, 135.1, 133.4, 132.4, 128.9, 127.9, 127.64, 127.56, 126.9, 126.3, 125.8, 125.4, 123.4, 46.6, 44.5.

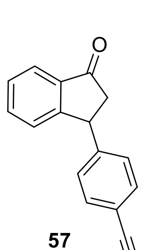

57

3-(4-Ethynylphenyl)-2,3-dihydro-1*H*-inden-1-one (57). Prepared according to the general procedure employing Ir[dF(CF_3)ppy]$_2$(phen)PF_6 (5.3 mg, 0.005 mmol), 2-oxo-2-phenylacetate **1** (147.5 mg, 0.983 mmol), 1,4-diethynylbenzene (63.2 mg, 0.5 mmol), K_2HPO_4 (210.0 mg, 1.2 mmol), MeCN (19 mL) and H_2O (1 mL). After 24 h, the reaction mixture was cooled to rt and filtered through a short pad of silica using EA. The filtrate was concentrated in *vacuo* before it was purified by flash chromatography (PE/EA = 10/1 to 3/1) on silica gel to afford **57** **(**23.3 mg, 0.100 mmol, 20% yield) as a yellow oil and **58**

(34.0 mg, 0.100, 20%) as a yellow solid. IR *v* 2922, 1708, 1598 cm^{-1}; ^{1}H NMR (500 MHz, $CDCl_3$) δ 7.82 (d, *J* = 7.5 Hz, 1H), 7.61-7.55 (m, 1H), 7.48-7.40 (m, 3H), 7.25 (d, *J* = 8.0 Hz, 1H), 7.08 (d, *J* = 8.0 Hz, 2H), 4.58 (dd, *J* = 8.0, 3.5 Hz, 1H), 3.23 (dd, *J* = 19.0, 8.0 Hz, 1H), 3.06 (s, 1H), 2.65 (dd, *J* = 19.0, 3.5 Hz, 1H); ^{13}C NMR (126 MHz, $CDCl_3$) δ 205.4, 157.2, 144.5, 136.7, 135.2, 132.7, 128.1, 127.6, 126.8, 123.5, 120.9, 83.2, 77.3, 46.6, 44.3; HRMS (ESI-TOF) Calcd. for $C_{17}H_{12}ONa$ $[M+Na]^+$: 255.0786; Found 255.0782.

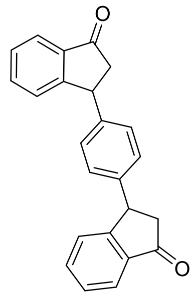

58

3,3'-(1,4-Phenylene)bis(2,3-dihydro-1*H*-inden-1-one) (58). Prepared according to the modified procedure employing $Ir[dF(CF_3)ppy]_2(phen)PF_6$ (5.0 mg, 0.005 mmol), 2-oxo-2-phenylacetate **1** (150.1 mg, 0.983 mmol), 1,4-diethynylbenzene (32.0 mg, 0.25 mmol), K_2HPO_4 (210.6 mg, 1.2 mmol), MeCN (19 mL) and H_2O (1 mL). After 24 h, the reaction mixture was cooled to rt and filtered through a short pad of silica using EA. The filtrate was concentrated in *vacuo* before it was purified by flash chromatography (PE/EA = 10/1 to 3/1) on silica gel to afford **58** (51.4 mg, 0.152 mmol, 61%, *dr* 1.1/1) as a yellow solid. IR *v* 3026, 2922, 1709, 1600 cm^{-1}; ^{1}H NMR (500 MHz, $CDCl_3$) δ 7.80 (d, *J* = 7.5 Hz, 2H), 7.60-7.54 (m, 2H), 7.45-7.38 (m, 2H), 7.28 (d, *J* = 8.0 Hz, 2H), 7.07 (s, 4H), 4.57 (dd, *J* = 8.0, 4.0 Hz, 2H), 3.21 (dd, *J* = 19.0, 8.0 Hz, 2H), 2.68 (dd, *J* = 4.0, 4.0 Hz, 1.05H), 2.64 (dd, *J* = 4.0, 4.0 Hz, 0.95H); ^{13}C NMR (126 MHz, $CDCl_3$) δ 205.79, 205.77, 157.6, 142.3, 136.6, 135.0, 128.0, 127.8, 126.77, 167.75, 123.3, 46.67, 46.66, 43.9; HRMS (ESI-TOF) Calcd. for $C_{24}H_{19}O_2$ $[M+H]^+$:339.1385; Found 339.1379.

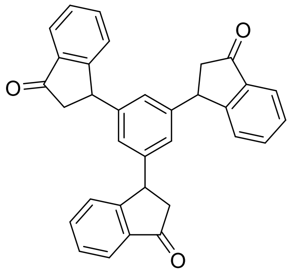

59

3,3',3''-(Benzene-1,3,5-triyl)tris(2,3-dihydro-1*H*-inden-1-one) (59). Prepared according to the modified procedure employing $Ir[dF(CF_3)ppy]_2(phen)PF_6$ (5.5 mg, 0.005 mmol), 2-oxo-2-phenylacetate **1** (150.5 mg, 1.0 mmol), 1,3,5-triethynylbenzene (25.2 mg, 0.167 mmol), K_2HPO_4 (210.0 mg, 1.2 mmol), MeCN (19 mL) and H_2O (1 mL). After 24 h, the reaction mixture was cooled to rt and filtered through a short pad of silica using EA. The filtrate was concentrated in *vacuo* before it was purified by flash chromatography (PE/EA = 3/1 to 1/1) on silica gel to afford **59 (**31.2 mg, 0.067 mmol, 40% yield, *dr* 1.7/1/1.5) as a yellow solid. IR *v* 2921, 1707, 1599 cm^{-1}; ^{1}H NMR (500 MHz,

$CDCl_3$) δ 7.79 (d, *J* = 7.5 Hz, 3H), 7.61-7.53 (m, 3H), 7.45-7.39 (m, 3H), 7.24-7.16 (m, 3H), 6.79-6.71 (m, 3H), 4.51-4.42 (m, 3H), 3.23-3.12 (m, 3H), 2.64 (dd, *J* = 16.0, 4.0 Hz, 1.23H), 2.61 (dd, *J* = 5.0, 4.0 Hz, 0.70H), 2.58 (dd, *J* = 8.5, 4.0 Hz, 1.04H); ^{13}C NMR (126 MHz, $CDCl_3$) δ 205.49, 205.46, 205.42, 157.28, 157.24, 157.19, 145.20, 145.17, 136.7, 135.11, 135.07, 135.06, 128.09, 128.06, 128.04, 126.7, 126.6, 126.5, 125.6, 125.4, 123.58, 123.56, 123.54, 46.7, 46.63, 46.58, 44.3, 44.24, 44.20; HRMS (ESI-TOF) Calcd. for $C_{33}H_{24}NO_3Na$ [M+Na]$^+$: 491.1623; Found 491.1621.

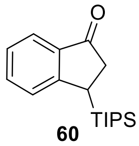

60

3-(Triisopropylsilyl)-2,3-dihydro-1*H*-inden-1-one (60). Prepared according to the general procedure employing Ir[dF(CF_3)ppy]$_2$(phen)PF_6 (5.3 mg, 0.005 mmol), 2-oxo-2-phenylacetate **1** (151.0 mg, 1.0 mmol), triisopropylsilylacetylene (55 μL, 0.5 mmol), K_2HPO_4 (210.0 mg, 1.2 mmol), MeCN (19 mL) and H_2O (1 mL). After 24 h, the reaction mixture was cooled to rt and filtered through h a short pad of silica using EA. The filtrate was concentrated in *vacuo* before it was purified by flash chromatography (PE/EA = 20/1) on silica gel to afford **60 (**62.3 mg, 0.216 mmol, 43% yield) as a white solid. mp = 72-73 °C. IR *v* 2942, 2864, 1706, 1463, 762 cm^{-1}; ^{1}H NMR (500 MHz, $CDCl_3$) δ 7.75 (d, *J* = 7.5 Hz, 1H), 7.52 (dd, *J* = 7.5, 7.5 Hz, 1H), 7.43 (d, *J* = 7.5 Hz, 1H), 7.31-7.25 (m, 1H), 3.17-3.12 (m, 1H), 2.91 (dd, *J* = 19.5, 8.5 Hz, 1H), 2.78 (dd, *J* = 19.5, 2.3 Hz, 1H), 1.27-1.16 (m, 3H), 1.07 (d, *J* = 7.5 Hz, 9H), 0.95 (d, *J* = 7.5 Hz, 9H); ^{13}C NMR (126 MHz, $CDCl_3$) δ 207.2, 159.5, 136.5, 134.3, 126.0, 125.8, 124.2, 40.0, 24.4, 18.8, 18.6, 11.4; HRMS (ESI-TOF) Calcd. for $C_{18}H_{28}OSiNa$ [M+Na]$^+$: 311.1807; Found 311.1806.

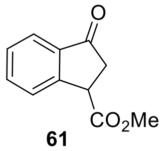

61

Methyl 3-oxo-2,3-dihydro-1*H*-indene-1-carboxylate (61).[32] Prepared according to the general procedure employing Ir[dF(CF_3)ppy]$_2$(phen)PF_6 (5.3 mg, 0.005 mmol), 2-oxo-2-phenylacetate **1** (150.3 mg, 1.0 mmol), methyl propiolate (45 μL, 0.5 mmol), K_2HPO_4 (210.0 mg, 1.2 mmol), MeCN (19 mL) and H_2O (1 mL). After 24 h, the reaction mixture was cooled to rt and filtered through a short pad of silica using EA. The filtrate was concentrated in *vacuo* before it was purified by flash chromatography (PE/EA = 3/1) on silica gel to afford **61 (**70.6 mg, 0.371 mmol, 74% yield) as a yellow oil. ^{1}H NMR (500 MHz, $CDCl_3$) δ 7.78 (d, *J* = 7.5 Hz, 1H), 7.70 (d, *J* = 8.0 Hz, 1H), 7.64 (dd, *J* = 7.5, 7.5 Hz, 1H), 7.46 (dd, *J* = 7.5, 7.5 Hz, 1H), 4.31 (dd, *J* = 8.0, 3.5 Hz, 1H), 3.79 (s, 3H), 3.15 (dd, *J* = 19.0, 3.5 Hz, 1H), 2.89 (dd, *J* = 19.0, 8.0 Hz, 1H); ^{13}C NMR (126 MHz, $CDCl_3$) δ 204.0, 172.2, 151.0, 136.3, 135.0, 128.8, 126.5, 123.9, 52.6, 43.6, 39.5.

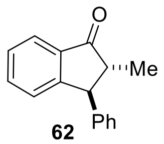

62

2-Methyl-3-phenyl-2,3-dihydro-1*H*-inden-1-one (62).[21] Prepared according to the general procedure employing Ir[dF(CF_3)ppy]$_2$(phen)PF_6 (5.3 mg, 0.005 mmol), 2-oxo-2-phenylacetate **1** (150.8 mg, 1.0 mmol), 1-phenyl-1-propyne (63 μL, 0.5 mmol), K_2HPO_4 (210.0 mg, 1.2 mmol), MeCN (19 mL) and H_2O (1 mL). After 24 h, the reaction mixture was cooled to rt and filtered through a short pad of silica using EA. The filtrate was concentrated in *vacuo* before it was purified by flash chromatography (PE/EA = 20/1) on silica gel to afford **62** (58.0 mg, 0.261 mmol, 52% yield, 7.2/1 *dr*) as a yellow oil. The *dr* was determined by ^{1}H NMR through crude mixture. ^{1}H NMR (500 MHz, $CDCl_3$) δ 7.86-7.79 (m, 1H), 7.62-7.54 (m, 1H), 7.47-7.40 (m, 1H), 7.37-7.32 (m, 2H), 7.32-7.27 (m, 1H), 7.24-7.20 (m, 1H), 7.19-7.14 (m, 1.85H), 6.89 (d, *J* = 7.0 Hz, 0.15H), 4.75 (d, *J* = 8.5 Hz, 0.09H), 4.03 (d, *J* = 5.0 Hz, 0.91H), 3.11-3.04 (m, 0.09H), 2.69-2.61 (m, 0.91H), 1.38 (d, *J* = 7.0 Hz, 2.76H), 0.84 (d, *J* = 7.5 Hz, 0.24H); ^{13}C NMR (126 MHz, $CDCl_3$) (major) δ 207.8, 156.0, 142.8, 136.3, 135.0, 128.9, 128.0, 127.9, 127.1, 126.5, 123.5, 53.8, 53.5, 14.1.

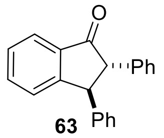

63

2,3-Diphenyl-2,3-dihydro-1*H*-inden-1-one (63). [33] Prepared according to the general procedure employing Ir[dF(CF_3)ppy]$_2$(phen)PF_6 (5.3 mg, 0.005 mmol), 2-oxo-2-phenylacetate **1** (148.9 mg, 1.0 mmol), diphenylacetylene (90.8 mg, 0.5 mmol), K_2HPO_4 (210.0 mg, 1.2 mmol), MeCN (19 mL) and H_2O (1 mL). After 24 h, the reaction mixture was cooled to rt and filtered through a short pad of silica using EA. The filtrate was concentrated in *vacuo* before it was purified by flash chromatography (PE/EA = 15/1) on silica gel to afford **63** (72.5 mg, 0.292 mmol, 73% yield, >20/1 *dr*) as a yellow oil. ^{1}H NMR (500 MHz, $CDCl_3$) δ 7.88 (d, *J* = 8.0 Hz, 1H), 7.64-7.59 (m, 1H), 7.46 (dd, *J* = 7.5, 7.5 Hz, 1H), 7.34-7.23 (m, 7H), 7.13-7.03 (m, 4H), 4.57 (d, *J* = 5.0 Hz, 1H), 3.81 (d, *J* = 5.0 Hz, 1H); ^{13}C NMR (126 MHz, $CDCl_3$) δ 205.1, 156.0, 142.4, 138.4, 136.1, 135.3, 128.84, 128.77, 128.3, 128.2, 127.8, 127.1, 126.6, 123.9, 64.5, 54.8.

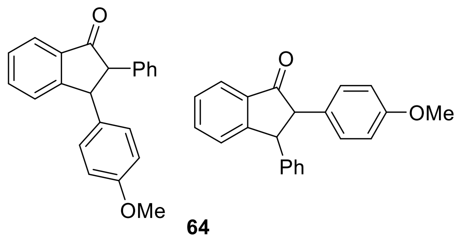

64

3-(4-Methoxyphenyl)-2-phenyl-2,3-dihydro-1*H*-inden-1-one and **2-(4-methoxyphenyl)-3-phenyl-2,3-dihydro-1*H*-inden-1-one (64)**. Prepared according to the general procedure employing Ir[dF(CF_3)ppy]$_2$(phen)PF_6 (5.3 mg, 0.005 mmol), 2-oxo-

2-phenylacetate **1** (151.0 mg, 1.0 mmol), 1-methoxy-4-(2-phenylethynyl)benzene (103.4 mg, 0.5 mmol), K_2HPO_4 (210.2 mg, 1.2 mmol), MeCN (19 mL) and H_2O (1 mL). After 24 h, the reaction mixture was cooled to rt and filtered through a short pad of silica using EA. The filtrate was concentrated in *vacuo* before it was purified by flash chromatography (PE/EA = 15/1) on silica gel to afford **64** (78.7 mg, 0.250 mmol, 50% yield) as a yellow oil. IR *v* 2955, 2924, 1710, 1604, 1512, 1462 cm^{-1}; ^{1}H NMR (500 MHz, $CDCl_3$) δ 7.87 (d, *J* = 7.5 Hz, 1H), 7.62 (t, *J* = 7.5 Hz, 1H), 7.50-7.44 (m, 1H), 7.34-7.22 (m, 4H), 7.04-6.97 (m, 2H), 7.04-6.97 (m, 2H), 6.87-6.81 (m, 2H), 4.52 (d, *J* = 5.0 Hz, 1H), 3.80-3.73 (m, 4H); ^{13}C NMR (126 MHz, $CDCl_3$) δ 205.4 (205.2), 158.70 (158.67), 156.3 (155.9), 142.4, 138.5, 136.10 (136.07), 135.31 (135.29), 134.4, 130.4, 129.3, 128.85 (128.83), 128.7, 128.3, 128.2 (128.14), 127.8, 127.1, 126.60 (126.57), 123.92 (123.88), 114.25 (114.23), 64.8 (63.9), 55.20 (55.19), 54.9, 54.1; HRMS (ESI-TOF) Calcd. for $C_{22}H_{18}O_2Na$ $[M+Na]^+$: 337.1204; Found 337.1203.

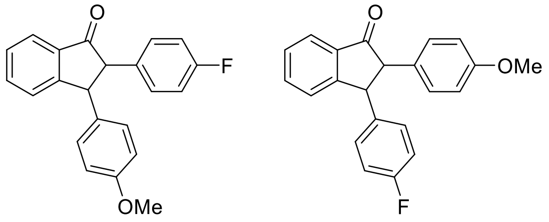

65

2-(4-Fluorophenyl)-3-(4-methoxyphenyl)-2,3-dihydro-1*H*-inden-1-one and 3-(4-Fluorophenyl)-2-(4-methoxyphenyl)-2,3-dihydro-1*H*-inden-1-one (65). Prepared according to the general procedure employing $Ir[dF(CF_3)ppy]_2(phen)PF_6$ (5.3 mg, 0.005 mmol), 2-oxo-2-phenylacetate **1** (147.6 mg, 1.0 mmol), 1-fluoro-4-((4-methoxyphenyl)ethynyl)benzene (113.2 mg, 0.5 mmol), K_2HPO_4 (210.0 mg, 1.2 mmol), MeCN (19 mL) and H_2O (1 mL). After 24 h, the reaction mixture was cooled to rt and filtered through a short pad of silica using EA. The filtrate was concentrated in *vacuo* before it was purified by flash chromatography (PE/EA = 15/1) on silica gel to afford a regioselective mixture of **65** (109.5 mg, 0.330 mmol, 66% yield) as a yellow oil. IR *v* 2956, 2926, 1714, 1601, 1510, 1250 cm^{-1}; ^{1}H NMR (500 MHz, $CDCl_3$) δ 7.88-7.83 (m, 1H), 7.65-7.58 (m, 1H), 7.49-7.42 (m, 1H), 7.29-7.25 (m, 1H), 7.08-7.02 (m, 2H), 7.01-6.93 (m, 4H), 6.84 (d, *J* = 8.0 Hz, 2H), 4.50 (d, *J* = 5.0 Hz, 0.41H), 4.45 (d, *J* = 5.0 Hz, 0.58 H), 3.79-3.72 (m, 3.59H), 3.69 (d, *J* = 5.0 Hz, 0.41H); ^{19}F NMR (471 MHz, $CDCl_3$) δ -115.30, -115.31, -115.39, -115.40; ^{13}C NMR (126 MHz, $CDCl_3$) δ 205.0, 204.9, 161.9 (d, *J* = 246.0 Hz), 161.8 (d, *J* = 246.0 Hz), 158.7, 156.0, 155.5, 138.10, 138.08, 136.0, 135.8, 135.4, 135.3, 134.03, 134.00, 130.0, 129.9, 129.8, 129.33, 129.29, 129.27, 128.8, 128.3, 128.2, 126.5, 126.4, 123.9, 123.8, 115.6 (d, *J* = 21.4 Hz), 115.5 (d, *J* = 21.4 Hz), 114.2, 64.0, 63.9,

55.09, 55.08, 54.1; HRMS (ESI-TOF) Calcd. for $C_{22}H_{18}FO_2$ [M+H]$^+$: 333.1291; Found 333.1289.

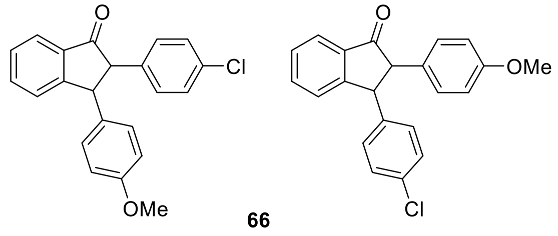

2-(4-Chlorophenyl)-3-(4-methoxyphenyl)-2,3-dihydro-1*H*-inden-1-one and **3-(4-Chlorophenyl)-2-(4-methoxyphenyl)-2,3-dihydro-1*H*-inden-1-one (66)**. Prepared according to the general procedure employing Ir[dF(CF_3)ppy]$_2$(phen)PF_6 (5.0 mg, 0.005 mmol), 2-oxo-2-phenylacetate **1** (150.9 mg, 1.0 mmol), 1-chloro-4-((4-methoxyphenyl)ethynyl)benzene (121.2 mg, 0.5 mmol), K_2HPO_4 (210.0 mg, 1.2 mmol), MeCN (19 mL) and H_2O (1 mL). After 24 h, the reaction mixture was cooled to rt and filtered through a short pad of silica using EA. The filtrate was concentrated in *vacuo* before it was purified by flash chromatography (PE/EA = 15/1) on silica gel to afford a regioselective mixture of **66 (**108.0 mg, 0.310 mmol, 62% yield) as a yellow solid. IR *v* 2931, 2837, 1714, 1601, 1512, 1249 cm^{-1}; ^{1}H NMR (500 MHz, $CDCl_3$) δ 7.92-7.81 (m, 1H), 7.68-7.58 (m, 1H), 7.53-7.43 (m, 1H), 7.34-7.25 (m, 3H), 7.08-6.93 (m, 4H), 6.90-6.79 (m, 2H), 4.87 (d, J = 3.5 Hz, 0.43 H), 4.45 (d, J = 4.0 Hz, 0.57 H), 3.84-3.72 (m, 3.44 H), 3.68 (d, J = 4.5 Hz, 0.55 H); ^{13}C NMR (126 MHz, $CDCl_3$) δ 205.0, 204.6, 158.8, 156.0, 155.2, 140.9, 136.8, 136.1, 135.9, 135.5, 135.4, 134.0, 133.0, 132.97, 130.0, 129.8, 129.4, 129.2, 129.0, 128.9, 128.8, 128.4, 128.3, 126.6, 126.4, 124.0, 123.9, 114.3, 64.2, 64.0, 55.22, 55.21, 54.3, 54.1; HRMS (ESI-TOF) Calcd. for $C_{22}H_{18}ClO_2$ [M+H]$^+$: 349.0995; Found 349.098.

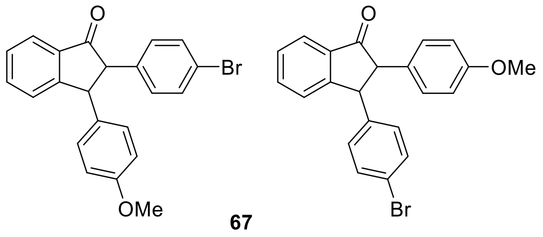

2-(4-Bromophenyl)-3-(4-methoxyphenyl)-2,3-dihydro-1*H*-inden-1-one and **3-(4-Bromophenyl)-2-(4-methoxyphenyl)-2,3-dihydro-1*H*-inden-1-one (67)**. Prepared according to the general procedure employing Ir[dF(CF_3)ppy]$_2$(phen)PF_6 (5.0 mg, 0.005 mmol), 2-oxo-2-phenylacetate **1** (151.0 mg, 1.0 mmol), 1-bromo-4-((4-methoxyphenyl)ethynyl)benzene (137.0 mg, 0.477 mmol), K_2HPO_4 (210.2 mg, 1.2 mmol), MeCN (19 mL) and H_2O (1 mL). After 24 h, the reaction mixture was cooled to rt and filtered through a short pad of silica using EA. The filtrate was concentrated in *vacuo* before it was purified by flash chromatography (PE/EA = 10/1) on silica gel to afford a regioselective mixture **67**

(111.7 mg, 0.284 mmol, 57% yield) as a yellow solid. IR *v* 2955, 2925, 1715, 1607, 1512, 1249 cmn^{-1}; ^{1}H NMR (500 MHz, $CDCl_3$) δ 7.87 (dd, *J* = 7.0, 7.0 Hz, 1H), 7.67-7.59 (m, 1H), 7.51-7.44 (m, 1H), 7.43 (d, *J* = 8.5 Hz, 2H), 7.31-7.24 (m, 1H), 7.03-6.92 (m, 4H), 6.85 (d, *J* = 8.0 Hz, 2H), 4.50-4.42 (m, 1H), 3.78 (d, *J* = 6.0 Hz, 3H), 3.72 (d, *J* = 5.0 Hz, 0.5H), 3.68 (d, *J* = 5.5 Hz, 0.5H); HRMS (ESI-TOF) Calcd. for $C_{22}H_{18}BrO_2$ $[M+H]^+$: 393.0490; Found 393.0492.

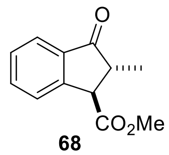
68

(*E*)-Methyl 2-methyl-3-oxo-2,3-dihydro-1*H*-indene-1-carboxylate (68).[34] Prepared according to the general procedure employing $Ir[dF(CF_3)ppy]_2(phen)PF_6$ (5.0 mg, 0.005 mmol), 2-oxo-2-phenylacetate **1** (150.9 mg, 1.0 mmol), methyl but-2-ynoate (49.1 mg, 0.5 mmol), K_2HPO_4 (210.0 mg, 1.2 mmol), MeCN (19 mL) and H_2O (1 mL). After 24 h, the reaction mixture was cooled to rt and filtered through a short pad of silica using EA. The filtrate was concentrated in *vacuo* and the crude mixture was monitored by ^{1}H NMR (*dr* = 9/1, trans/cis), and then purified by flash chromatography (PE/EA = 5/1) on silica gel to afford **68 (**43.8 mg, 0.214 mmol, 43% yield) as a white solid. ^{1}H NMR (500 MHz, $CDCl_3$) δ 7.96-7.89 (m, 0.08H), 7.83-7.75 (m, 0.92 H), 7.69-7.60 (m, 2H), 7.53-7.42 (m, 1H), 4.41 (d, *J* = 8.0 Hz, 0.08 H), 3.86 (d, *J* = 4.5 Hz, 0.90 H), 3.82 (s, 2.66 H), 3.70 (s, 0.23H), 3.16-3.07 (m, 0.89H), 3.05-2.96 (m, 0.10H), 1.39 (d, *J* = 7.5 Hz, 2.68 H), 1.28 (d, *J* = 7.5 Hz, 0.33 H); ^{13}C NMR (126 MHz, $CDCl_3$) δ 206.2, 172.2, 149.4, 135.7, 135.0, 128.8, 126.3, 124.1, 52.6, 52.2, 46.0, 15.2.

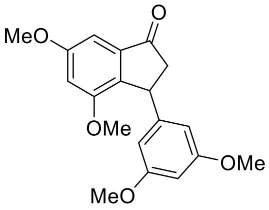
69

3-(3,5-Dimethoxyphenyl)-4,6-dimethoxy-2,3-dihydro-1*H*-inden-1-one (69).[35] Prepared according to the general procedure employing $Ir[dF(CF_3)ppy]_2(phen)PF_6$ (5.2 mg, 0.005 mmol), 2-(3,5-dimethoxyphenyl)-2-oxoacetic acid (216.1 mg, 1.0 mmol), 3,5-dimethoxyphenylacetylene (81.0 mg, 0.5 mmol), K_2HPO_4 (210.0 mg, 1.2 mmol), MeCN (19 mL) and H_2O (1 mL). After 24 h, the reaction mixture was cooled to rt and filtered through a short pad of silica using EA. The filtrate was concentrated in *vacuo* before it was purified by flash chromatography (PE/EA = 3/1) on silica gel to afford **69 (**132.0 mg, 0.402 mmol, 80% yield) as a yellow solid. ^{1}H NMR (500 MHz, $CDCl_3$) δ 6.84 (d, *J* = 2.0 Hz, 1H), 6.63 (d, *J* = 2.0 Hz, 1H), 6.29 (t, *J* = 2.0 Hz, 1H), 6.19 (d, *J* = 2.5 Hz, 2H), 4.49 (dd, *J* = 8.0, 2.0 Hz, 1H), 3.85 (s, 3H), 3.72 (s, 6H), 3.67 (s, 3H), 3.16 (dd, *J* = 19.0, 8.0 Hz, 1H), 2.59 (dd, *J* = 19.0, 2.0 Hz, 1H); ^{13}C NMR (126 MHz, $CDCl_3$) δ 206.0, 161.7, 160.7, 157.8, 146.5, 139.1, 138.9, 106.0, 105.2, 98.0,

95.8, 55.6, 55.5, 55.1, 47.4, 41.3.

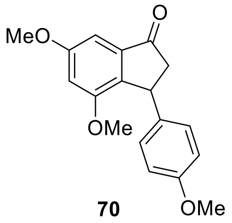

70

4,6-Dimethoxy-3-(4-methoxyphenyl)-2,3-dihydro-1*H*-inden-1-one (70).[28] Prepared according to the general procedure employing Ir[dF(CF_3)ppy]$_2$(phen)PF_6 (5.3 mg, 0.005 mmol), 2-(3,5-dimethoxyphenyl)-2-oxoacetic acid (217.7 mg, 1.0 mmol), 4-ethynylanisole (67.1 mg, 0.5 mmol), K_2HPO_4 (210.0 mg, 1.2 mmol), MeCN (19 mL) and H_2O (1 mL). After 24 h, the reaction mixture was cooled to rt and filtered through a short pad of silica using EA. The filtrate was concentrated in *vacuo* before it was purified by flash chromatography (PE/EA = 3/1) on silica gel to afford **70 (**100.3 mg, 0.336 mmol, 67% yield) as a yellow solid. ^{1}H NMR (500 MHz, $CDCl_3$) δ 6.96 (d, *J* = 8.5 Hz, 2H), 6.84 (d, *J* = 2.0 Hz, 1H), 6.79-6.76 (m, 2H), 6.62 (d, *J* = 2.5 Hz, 1H), 4.52 (dd, *J* = 7.5, 2.0 Hz, 1H), 3.85 (s, 3H), 3.76 (s, 3H), 3.65 (s, 3H), 3.18 (dd, *J* = 19.0, 8.0 Hz, 1H), 2.57 (dd, *J* = 19.0, 2.0 Hz, 1H); ^{13}C NMR (126 MHz, $CDCl_3$) δ 206.5, 161.6, 158.0, 157.8, 139.7, 138.9, 136.0, 127.9, 113.7, 106.1, 95.7, 55.7, 55.5, 55.1, 47.7, 40.4.

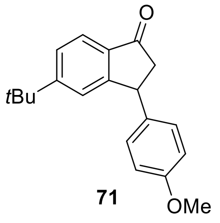

71

5-(*Tert*-butyl)-3-(4-methoxyphenyl)-2,3-dihydro-1*H*-inden-1-one (71). Prepared according to the general procedure employing Ir[dF(CF_3)ppy]$_2$(phen)PF_6 (5.3 mg, 0.005 mmol), 2-(4-(*tert*-butyl)phenyl)-2-oxoacetic acid (205.6 mg, 1.0 mmol), 4-ethynylanisole (66.1 mg, 0.5 mmol), K_2HPO_4 (210.0 mg, 1.2 mmol), MeCN (19 mL) and H_2O (1 mL). After 24 h, the reaction mixture was cooled to rt and filtered through a short pad of silica using EA. The filtrate was concentrated in *vacuo* before it was purified by flash chromatography (PE/EA = 10/1) on silica gel to afford **71 (**109.3 mg, 0.371 mmol, 74% yield) as a yellow solid. mp = 104-105 °C. IR *v* 2954, 1711, 1604, 1246 cm^{-1}; ^{1}H NMR (500 MHz, $CDCl_3$) δ 7.73 (d, *J* = 8.0 Hz, 1H), 7.47 (dd, *J* = 8.0, 1.0 Hz, 1H), 7.25 (s, 1H), 7.07-7.02 (m, 2H), 6.88-6.82 (m, 2H), 4.51 (dd, *J* = 8.0, 4.0 Hz, 1H), 3.79 (s, 3H), 3.19 (dd, *J* = 19.0, 8.0 Hz, 1H), 2.61 (dd, *J* = 19.0, 4.0 Hz, 1H), 1.29 (s, 9H); ^{13}C NMR (126 MHz, $CDCl_3$) δ 205.6, 159.3, 158.4, 158.3, 135.9, 134.4, 128.5, 125.5, 123.2, 122.9, 114.2, 55.2, 47.4, 43.7, 35.5, 31.1; HRMS (ESI-TOF) Calcd. for $C_{20}H_{23}O_2$ [M+H]$^+$: 295.1698; Found 295.1693.

Gram-Scale synthesis: Prepared according to the modified procedure employing Ir[dF(CF_3)ppy]$_2$(phen)PF_6 (62.7 mg, 0.061 mmol), 2-(4-(*tert*-butyl)phenyl)-2-oxoacetic acid (2.4762 g, 12.00 mmol), 4-ethynylanisole (800.0 mg, 6.053 mmol), K_2HPO_4 (2.4390 g, 14.0 mmol), MeCN (114 mL) and H_2O (6 mL). After 48 h, the reaction mixture was cooled to rt and filtered

through a short pad of silica using EA. The filtrate was concentrated in *vacuo* before it was purified by flash chromatography (PE/EA = 10/1) on silica gel to afford **71** (1.3223 g, 4.493 mmol, 74% yield) as a yellow solid.

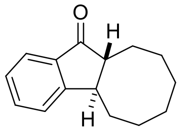

72

(4b*R*,10a*R*)-4b,5,6,7,8,9,10,10a-octahydro-11*H*-cycloocta[a]inden-11-one (72). [36] Prepared according to the general procedure employing Ir[dF(CF_3)ppy]$_2$(phen)PF_6 (5.2 mg, 0.005 mmol), 2-Oxo-2-phenylacetate **1** (153.2 mg, 1.0 mmol), cyclooctyne (54.1 mg, 0.5 mmol), K_2HPO_4 (209.1 mg, 1.2 mmol), MeCN (19 mL) and H_2O (1 mL). After 24 h, the reaction mixture was cooled to rt and filtered through a short pad of silica using EA. The filtrate was concentrated in *vacuo* and the crude mixture was monitored by ^{1}H NMR (*dr* = 3.7/1, trans/cis) and then purified by flash chromatography (PE/EA = 15/1) on silica gel to afford **72** (32.2 mg, 0.15 mmol, 30% yield, *dr* 3.7/1) as a white solid. ^{1}H NMR (500 MHz, $CDCl_3$) δ 7.77-7.68 (m, 1H), 7.60 (t, *J* = 7.5 Hz, 1H), 7.54-7.45 (m, 1H), 7.40-7.30 (m, 1H), 3.45-3.37 (m, 0.18H), 3.27-3.13 (m, 0.82H), 2.63-2.55 (m, 0.18H), 2.54-2.45 (m, 0.82H), 2.44-2.22 (m, 2H), 2.07-1.76 (m, 4H), 1.74-1.54 (m, 4H), 1.48-1.37 (m, 1H), 1.36-1.29 (m, 1H); ^{13}C NMR (126 MHz, $CDCl_3$) δ 209.2, 208.8, 159.9, 158.4, 135.7, 134.84, 134.77, 127.3, 125.8, 125.0, 123.5, 123.4, 53.4, 52.7, 44.2, 44.0, 35.2, 31.50, 31.48, 30.3, 30.2, 27.4, 26.9, 25.9, 25.0, 24.7, 24.0.

Late-Stage Functionalizations of Complex Molecules

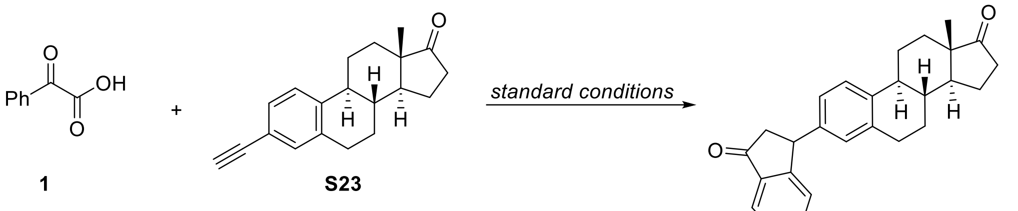

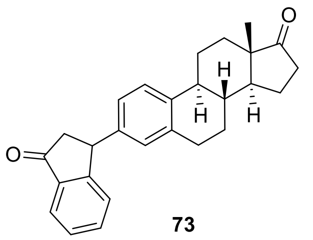

73

(8*R*,9*S*,13*S*,14*S*)-13-methyl-3-(3-oxo-2,3-dihydro-1*H*-inden-1-yl)-6,7,8,9,11,12,13,14,15,16-decahydro-17*H*-cyclopenta[*a*]phenanthren-17-one (73). Prepared according to the general procedure employing Ir[dF(CF_3)ppy]$_2$(phen)PF_6 (5.3 mg, 0.005 mmol), 2-oxo-2-phenylacetate **1** (149.7 mg, 1.0 mmol), **S23** (138.8 mg, 0.5 mmol), K_2HPO_4 (210.0 mg, 1.2 mmol), MeCN (19 mL) and H_2O (1 mL). After 24 h, the reaction mixture was cooled to rt and filtered through a short pad of silica using EA. The filtrate was concentrated in *vacuo* before it was purified by flash chromatography (PE/EA = 5/1) on silica gel to afford **73** (125.1 mg, 0.325 mmol, 65% yield) as a yellow solid. IR *v* 2925, 1737, 1711,

1602 cm^{-1}; ^{1}H NMR (500 MHz, $CDCl_3$) δ 7.80 (d, *J* = 7.5 Hz, 1H), 7.56 (dd, *J* = 7.5, 7.0 Hz, 1H), 7.40 (dd, *J* = 7.5, 7.5 Hz, 1H), 7.29 (d, *J* = 7.5 Hz, 1H), 7.23 (d, *J* = 6.0 Hz, 1H), 6.96-6.81 (m, 2H), 4.58-4.45 (m, 1H), 3.19 (dd, *J* = 19.5, 8.0 Hz, 1H), 2.94-2.79 (m, 2H), 2.67 (dd, *J* = 19.0, 3.0 Hz, 1H), 2.50 (dd, *J* = 19.5, 9.0 Hz, 1H), 2.44 - 2.35 (m, 1H), 2.33-2.22 (m, 1H), 2.19-2.09 (m, 1H), 2.09 -1.88 (m, 3H), 1.69-1.56 (m, 2H), 1.56-1.37 (m, 4H), 0.91 (s, 3H); ^{13}C NMR (126 MHz, $CDCl_3$) δ 206.09, 206.06, 158.0, 141.03, 141.00, 138.4, 137.0, 136.9, 136.6, 134.9, 128.1, 127.9, 127.7, 126.81, 126.80, 125.8, 125.7, 125.0, 124.9, 123.3, 50.4, 47.9, 46.8, 44.2, 43.9, 38.0, 35.7, 31.5, 29.3, 26.4, 25.6, 21.5, 13.8; HRMS (ESI-TOF) Calcd. for $C_{27}H_{28}O_2Na$ [M+Na]$^+$: 407.1987; Found 407.1984.

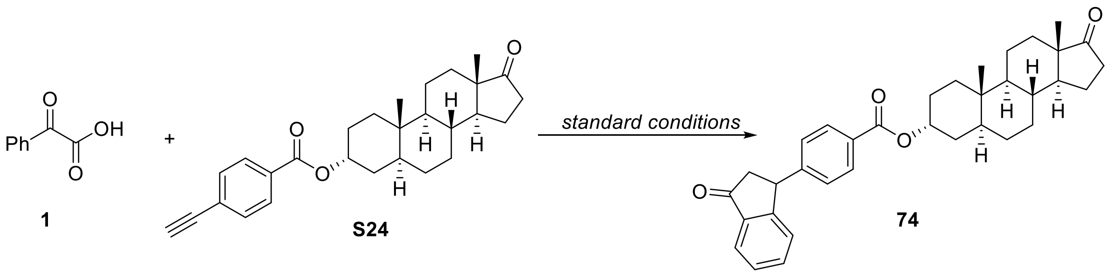

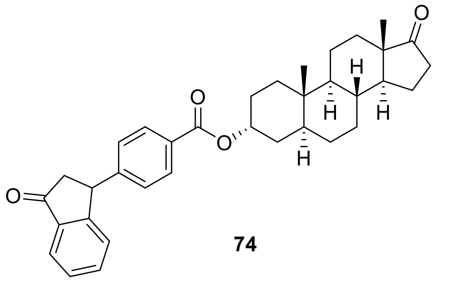

(3*R*,5*S*,8*R*,9*S*,10*S*,13*S*,14*S*)-10,13-dimethyl-17-oxohexadecahydro-1*H*-cyclopenta[*a*]phenanthren-3-yl 4-(3-oxo-2,3-dihydro-1*H*-inden-1-yl)benzoate (74). Prepared according to the general procedure employing Ir[dF(CF_3)ppy]$_2$(phen)PF_6 (5.3 mg, 0.005 mmol), 2-oxo-2-phenylacetate **1** (151.0 mg, 1.0 mmol), **S24** (209.5 mg, 0.5 mmol), K_2HPO_4 (209.8 mg, 1.2 mmol), MeCN (19 mL) and H_2O (1 mL). After 24 h, the reaction mixture was cooled to rt and filtered through a short pad of silica using EA. The filtrate was concentrated in *vacuo* before it was purified by flash chromatography (PE/EA = 3/1) on silica gel to afford **74 (**197.5 mg, 0.376 mmol, 75% yield) as a yellow solid. IR *v* 2925, 2855, 1737, 1713, 1607, 1277 cm^{-1}; ^{1}H NMR (500 MHz, $CDCl_3$) δ 8.01 (d, *J* = 8.0 Hz, 2H), 7.82 (d, *J* = 7.5 Hz, 1H), 7.58 (dd, *J* = 8.0, 7.0 Hz, 1H), 7.44 (dd, *J* = 7.5, 7.5 Hz, 1H), 7.28-7.18 (m, 3H), 5.31-5.24 (m, 1H), 4.66 (dd, *J* = 8.0, 3.5 Hz, 1H), 3.26 (dd, *J* = 19.0, 8.0 Hz, 1H), 2.67 (dd, *J* = 19.5, 3.5 Hz, 1H), 2.44 (dd, *J* = 19.0, 8.5 Hz, 1H), 2.11-2.00 (m, 1H), 1.98-1.74 (m, 5H), 1.73-1.56 (m, 6H), 1.54-1.44 (m, 1H), 1.41-1.17 (m, 6H), 1.09-0.94 (m, 1H), 0.87 (s, 6H), 0.85-0.78 (m, 1H); ^{13}C NMR (126 MHz, $CDCl_3$) δ 205.19, 205.17, 165.4, 157.0, 148.68, 148.67, 136.6, 135.1, 130.1, 129.8, 128.1, 127.6, 126.7, 123.5, 70.4, 54.4, 51.4, 47.7, 46.41, 46.39,

44.2, 40.4, 33.09, 33.08, 32.8, 31.4, 30.7, 28.0, 26.2, 21.6, 20.0, 13.7, 11.3; HRMS (ESI-TOF) Calcd. for $C_{35}H_{40}O_4Na$ $[M+Na]^+$: 547.2824; Found 547.2828.

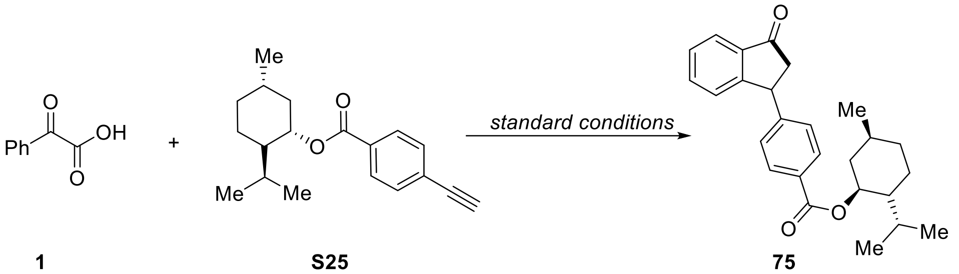

1 S25 75

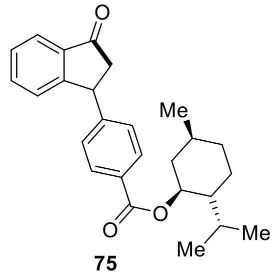

75

(1*S*,2*S*,5*S*)-2-isopropyl-5-methylcyclohexyl 4-(3-oxo-2,3-dihydro-1*H*-inden-1-yl)benzoate (75). Prepared according to the general procedure employing Ir[dF(CF_3)ppy]$_2$(phen)PF_6 (5.3 mg, 0.005 mmol), 2-Oxo-2-phenylacetate **1** (150.0 mg, 1.0 mmol), **S25** (142.1 mg, 0.5 mmol), K_2HPO_4 (209.8 mg, 1.2 mmol), MeCN (19 mL) and H_2O (1 mL). After 24 h, the reaction mixture was cooled to rt and filtered through a short pad of silica using EA. The filtrate was concentrated in *vacuo* before it was purified by flash chromatography (PE/EA = 10/1) on silica gel to afford **75 (**166.5 mg, 0.426 mmol, 85% yield) as a yellow solid. IR *v* 2955, 2925, 1714, 1607, 1273 cm^{-1}; ^{1}H NMR (500 MHz, $CDCl_3$) δ 8.00 (d, *J* = 8.0 Hz, 2H), 7.83 (d, *J* = 8.0 Hz, 1H), 7.58 (t, *J* = 7.5 Hz, 1H), 7.44 (t, *J* = 7.5 Hz, 1H), 7.25 (d, *J* = 1.0 Hz, 1H), 7.20 (d, *J* = 8.5 Hz, 2H), 4.92 (td, *J* = 11.0, 4.5 Hz, 1H), 4.65 (dd, *J* = 8.5, 4.0 Hz, 1H), 3.25 (dd, *J* = 19.5, 8.0 Hz, 1H), 2.73-2.63 (m, 1H), 2.16-2.06 (m, 1H), 2.00-1.87 (m, 1H), 1.77-1.67 (m, 2H), 1.63-1.48 (m, 2H), 1.19-1.02 (m, 2H), 0.95-0.86 (m, 7H), 0.81-0.76 (m, 3H); ^{13}C NMR (126 MHz, $CDCl_3$) δ 205.21 (205.20), 165.6, 157.1, 148.61 (148.60), 136.7, 135.15 (135.14), 130.2, 129.6, 128.1, 127.56 (127.55), 126.72 (126.71), 123.5, 74.8, 47.2, 46.45 (46.44), 44.30, 40.9, 34.2, 31.4, 26.49 (26.46), 23.61 (23.59), 22.0, 20.7, 16.51 (16.47); HRMS (ESI-TOF) Calcd. for $C_{26}H_{30}O_3Na$ $[M+Na]^+$: 413.2093; Found 413.2086.

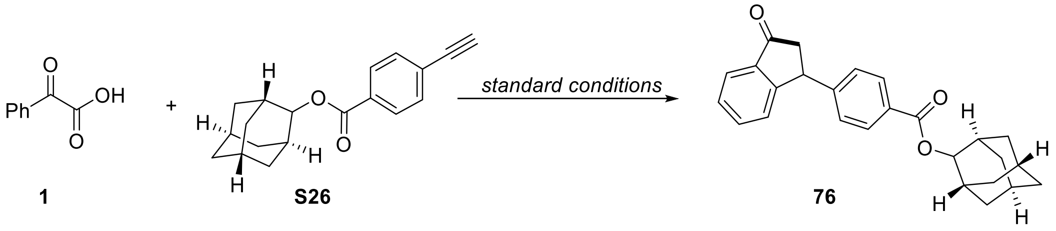

1 S26 76

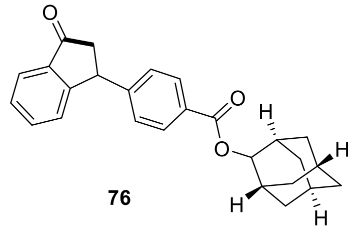

(1*r*,3*r*,5*r*,7*r*)-Adamantan-2-yl 4-(3-oxo-2,3-dihydro-1*H*-inden-1-yl)benzoate (76). Prepared according to the general procedure employing Ir[dF(CF_3)ppy]$_2$(phen)PF_6 (5.3 mg, 0.005 mmol), 2-Oxo-2-phenylacetate **1** (150.5 mg, 1.0 mmol), **S26** (140.0 mg, 0.5 mmol), K_2HPO_4 (209.5 mg, 1.2 mmol), MeCN (19 mL) and H_2O (1 mL). After 24 h, the reaction mixture was cooled to rt and filtered through a short pad of silica using EA. The filtrate was concentrated in *vacuo* before it was purified by flash chromatography (PE/EA = 10/1) on silica gel to afford **76 (**149.5 mg, 0.387 mmol, 77% yield) as a yellow solid. IR *v* 2907, 2854, 1713, 1609, 1278, 1100 cm^{-1}; ^{1}H NMR (500 MHz, $CDCl_3$) δ 8.04 (d, *J* = 8.0 Hz, 2H), 7.83 (d, *J* = 7.5 Hz, 1H), 7.58 (dd, *J* = 7.5, 7.0 Hz, 1H), 7.44 (dd, *J* = 7.5, 7.0 Hz, 1H), 7.30-7.15 (m, 3H), 5.21-5.12 (m, 1H), 4.65 (dd, *J* = 7.5, 3.5 Hz, 1H), 3.26 (dd, *J* = 19.5, 8.5 Hz, 1H), 2.68 (dd, *J* = 19.5, 3.5 Hz, 1H), 2.22-2.02 (m, 4H), 1.97-1.80 (m, 6H), 1.80-1.70 (m, 2H), 1.63 (d, *J* = 11.5 Hz, 2H); ^{13}C NMR (126 MHz, $CDCl_3$) δ 205.2, 165.4, 157.1, 148.6, 136.7, 135.2, 130.2, 130.0, 128.1, 127.6, 126.7, 123.6, 77.5, 46.5, 44.3, 37.3, 36.3, 32.0, 27.3, 27.0; HRMS (ESI-TOF) Calcd. for $C_{26}H_{27}O_3$ [M+H]$^+$: 387.1960; Found 387.1960.

Synthetic Applications of Indanones

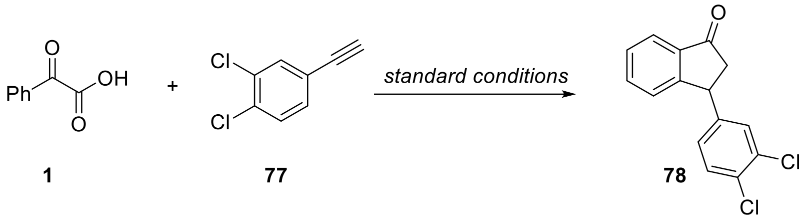

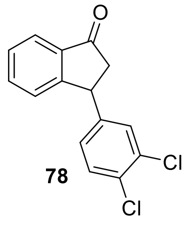

3-(3,4-Dichlorophenyl)-2,3-dihydro-1*H*-inden-1-one (78).[29] Prepared according to the general procedure employing Ir[dF(CF_3)ppy]$_2$(phen)PF_6 (5.2 mg, 0.005 mmol), 2-oxo-2-phenylacetate **1** (150.7 mg, 1.0 mmol), **77** (87.0 mg, 0.5 mmol), K_2HPO_4 (209.6 mg, 1.2 mmol), MeCN (19 mL) and H_2O (1 mL). After 24 h, the reaction mixture was cooled to rt and filtered through a short pad of silica using EA. The filtrate was concentrated in *vacuo* before it was purified by flash chromatography (PE/EA = 10/1) on silica gel to afford **78 (**86.5 mg, 0.312 mmol, 63% yield) as a yellow solid. ^{1}H NMR (500 MHz, $CDCl_3$) δ7.82 (d, *J* = 7.5 Hz, 1H), 7.60 (dd, *J* = 7.5, 7.5 Hz, 1H), 7.45 (dd, *J* = 7.5, 7.5 Hz, 1H), 7.38 (d, *J* = 8.5 Hz, 1H), 7.31-7.18 (m, 2H), 6.95 (dd, *J* = 8.5, 2.0 Hz, 1H), 4.55 (dd, *J* = 8.0, 3.5 Hz, 1H), 3.23 (dd, *J* = 18.5, 8.5 Hz, 1H), 2.62 (dd, *J* = 19.0, 3.5 Hz, 1H); ^{13}C NMR (126 MHz,

CDCl3) δ 204.8, 156.4, 143.9, 136.7, 135.3, 132.9, 131.0, 130.8, 129.6, 128.3, 126.9, 126.6, 123.6, 46.4, 43.5.

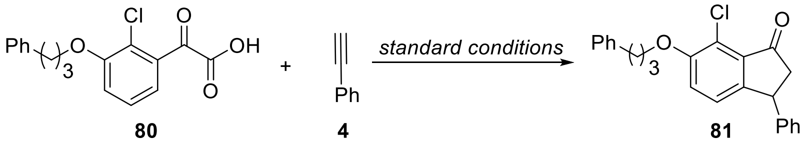

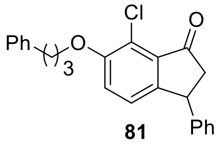

7-Chloro-3-phenyl-6-(3-phenylpropoxy)-2,3-dihydro-1*H*-inden-1-one (81). Prepared according to the general procedure employing Ir[dF(CF_3)ppy]$_2$(phen)PF_6 (5.4 mg, 0.005 mmol), 2-oxo-2-phenylacetate **1** (310.7 mg, 0.975 mmol), **4** (55 μL, 0.5 mmol), K_2HPO_4 (209.0 mg, 1.2 mmol), MeCN (19 mL) and H_2O (1 mL). After 24 h, the reaction mixture was cooled to rt and filtered through a short pad of silica using EA. The filtrate was concentrated in *vacuo* before it was purified by flash chromatography (PE/EA = 10/1) on silica gel to afford **81 (**149.9 mg, 0.398 mmol, 80% yield) as a yellow oil. IR *v* 2954, 2922, 1718, 1462 cm^{-1}; ^{1}H NMR (500 MHz, $CDCl_3$) δ 7.34-7.16 (m, 8H), 7.15-7.01 (m, 4H), 4.45 (dd, *J* = 8.0, 4.0 Hz, 1H), 4.03 (t, *J* = 6.0 Hz, 2H), 3.27 (dd, *J* = 19.0, 8.0 Hz, 1H), 2.88 (t, *J* = 7.5 Hz, 2H), 2.75 (dd, *J* = 19.0, 4.0 Hz, 1H), 2.22-2.11 (m, 2H); ^{13}C NMR (126 MHz, $CDCl_3$) δ 202.8, 154.5, 151.5, 143.6, 141.1, 133.6, 128.9, 128.5, 128.4, 127.5, 127.0, 126.0, 125.3, 120.2, 119.6, 68.8, 48.1, 42.8, 31.9, 30.6; HRMS (ESI-TOF) Calcd. for $C_{24}H_{22}ClO_2$ [M+H]$^+$: 377.1308; Found 377.1304.

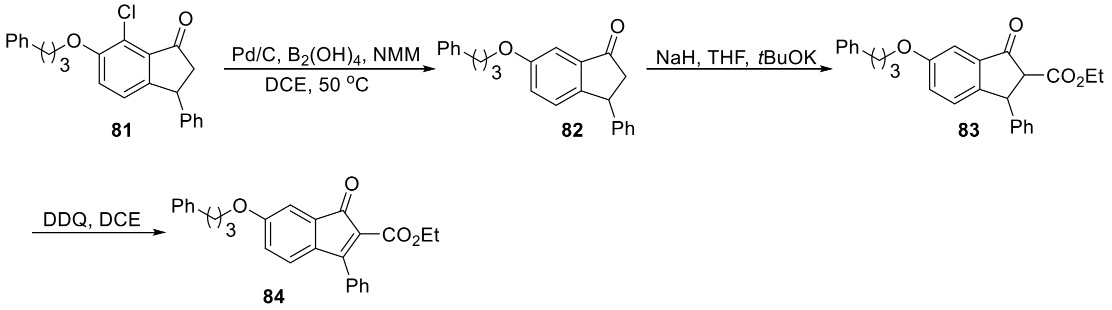

To a solution of **81** (147.0 mg, 0.390 mmol) in DCE (2.5 mL) in a 20 mL vial equipped with a micro stir bar, was added $B_2(OH)_4$ (80.0 mg, 0.892 mmol), 10% Pd/C (55.0 mg), and 4-methylmorpholine (NMM, 220 μL, 2.0 mmol). The vial was tightly capped and the reaction mixture was stirred at 50 °C for overnight. The mixture was then filtered through celite and the residue was washed with EtOAc (100 mL). The filtrate was concentrated under reduced pressure and passed through a short pad of silica gel using PE/EA (10/1) as eluents to afford a crude product **82**, which was used directly without further purification.

To an oven-dried flask cooled under N_2 was added NaH (60%, 38.4 mg, 0.96 mmol), diethyl carbonate (241µL, 2.0 mmol) and THF (5 mL). The flask was cooled to 0 °C and the crude **82** (dissolved in 3 mL THF) was added slowly. The reaction was stirred overnight. When the starting material was consumed, the reaction mixture was acidified with diluted HCl (1 N). After being extracted with EA, dried over anhydrous Na_2SO_4, filtered and condensed under reduced pressure, the residue was purified by flash column chromatography on silica gel to obtain **83** (110.0 mg, 68% yield) as a yellow oil over two steps.

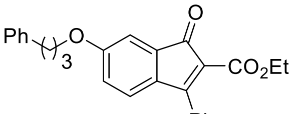

84

Ethyl 1-oxo-3-phenyl-6-(3-phenylpropoxy)-1*H*-indene-2-carboxylate (84).[37] To an oven-dried flask cooled under N_2 was added DDQ (45.5 mg, 0.20 mmol), **83** (54.7 mg, 0.132 mmol) and DCE (2 mL). The reaction mixture was stirred at 80 °C overnight. The reaction was diluted with DCM and filtered through a short pad of celite. The filtrate was condensed to give a crude product which was purified by flash column chromatography (PE/EA = 7/1) on silica gel to afford **84** (47.0 mg, 0.114 mmol, 86% yield) as a red solid. ^{1}H NMR (500 MHz, $CDCl_3$) δ 7.54-7.46 (m, 5H), 7.32-7.26 (m, 2H), 7.23-7.15 (m, 4H), 7.06 (d, *J* = 8.0 Hz, 1H), 6.80 (dd, *J* = 8.0, 2.0 Hz, 1H), 4.18 (q, *J* = 7.0 Hz, 2H), 4.00 (t, *J* = 6.5 Hz, 2H), 2.81 (t, *J* = 7.5 Hz, 2H), 2.18-2.08 (m, 2H), 1.15 (t, *J* = 7.0 Hz, 3H); ^{13}C NMR (126 MHz, $CDCl_3$) δ 191.9, 167.1, 162.9, 162.3, 141.1, 134.6, 133.0, 131.9, 130.3, 128.4, 128.3, 128.0, 126.0, 124.8, 122.6, 117.3, 111.1, 67.6, 60.6, 32.0, 30.6, 13.9.

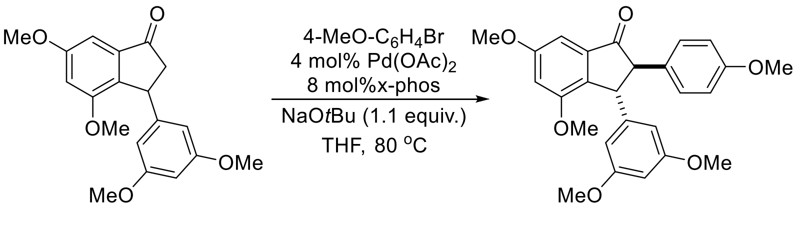

69 **86**

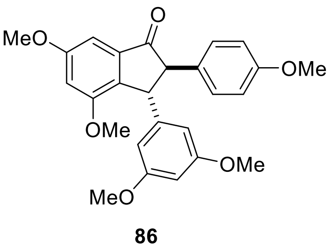

86

3-(3,5-Dimethoxyphenyl)-4,6-dimethoxy-2-(4-methoxyphenyl)-2,3-dihydro-1*H*-inden-1-one (86).[35] To an oven-dried 50 mL vial cooled under N_2, **69** (153.7 mg, 0.468 mmol), 4-bromoanisole (132.8 mg, 0.71 mmol), $Pd(OAc)_2$ (4.3 mg, 0.0188 mmol), X-Phos (17.3 mg, 0.038 mmol), NaO*t*Bu (51.1 mg, 0.532 mmol) and THF (4 mL) were added. The reaction was heated at 80 °C for overnight. The mixture was cooled to room

temperature and filtered through a short pad of silica gel while rinsing with EtOAc (20 mL). The solution was concentrated in *vacuo*, and the residue was purified through column chromatography (PE/EA = 4/1) on silica gel to afford **86** (167.1 mg, 0.385 mmol, 82% yield, >20/1 *dr*) as a red solid. ^{1}H NMR (500 MHz, $CDCl_3$) δ 7.02 (d, *J* = 8.5 Hz, 2H), 6.89 (d, *J* = 2.0 Hz, 1H), 6.83 (d, *J* = 8.5 Hz, 2H), 6.69 (d, *J* = 2.0 Hz, 1H), 6.31 (dd, *J* = 2.5, 2.0 Hz, 1H), 6.16 (d, *J* = 2.0 Hz, 2H), 4.44 (d, *J* = 3.0 Hz, 1H), 3.86 (s, 3H), 3.77 (s, 3H), 3.70 (s, 6H), 3.67 (s, 3H), 3.65 (d, *J* = 2.5 Hz, 1H); ^{13}C NMR (126 MHz, $CDCl_3$) δ 205.7, 162.0, 160.8, 158.6, 157.8, 145.9, 138.7, 137.5, 131.4, 128.8, 114.2, 106.4, 105.1, 98.1, 96.5, 64.1, 55.7, 55.6, 55.2, 51.9.

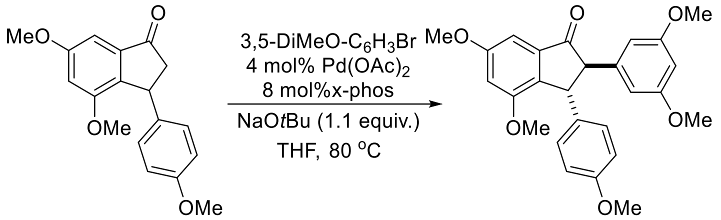

70 **89**

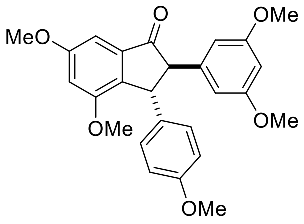

89

(2*R*,3*R*)-2-(3,5-dimethoxyphenyl)-4,6-dimethoxy-3-(4-methoxyphenyl)-2,3-dihydro-1*H*-inden-1-one (89).[38] To an oven-dried 20 mL vial cooled under N_2, indanone **70** (87.5 mg, 0.293 mmol), 3,5-dimethoxybromobenzene (96.2 mg, 0.443 mmol), $Pd(OAc)_2$ (2.7 mg, 0.012 mmol), X-Phos (11.1 mg, 0.024 mmol), NaO*t*Bu (31.1 mg, 0.33 mmol) and THF (5 mL) were added. The reaction was heated at 80 °C overnight. The mixture was cooled to room temperature and filtered through a short pad of silica gel while rinsing with EtOAc (20 mL). The solution was concentrated in *vacuo*, and the residue was purified through column chromatography (PE/EA = 4/1) on silica gel to afford **89** (113.8 mg, 0.262 mmol, 87% yield, >20/1 *dr*) as a red solid. ^{1}H NMR (500 MHz, $CDCl_3$) δ 6.94 (d, *J* = 9.0 Hz, 2H), 6.89 (d, *J* = 2.0 Hz, 1H), 6.78 (d, *J* = 8.5 Hz, 2H), 6.69 (d, *J* = 2.0 Hz, 1H), 6.35 (dd, *J* = 2.5, 2.0 Hz, 1H), 6.24 (d, *J* = 2.0 Hz, 2H), 4.51 (d, *J* = 2.5 Hz, 1H), 3.87 (s, 3H), 3.77 (s, 3H), 3.73 (s, 6H), 3.65 (s, 3H), 3.60 (d, *J* = 2.5 Hz, 1H); ^{13}C NMR (126 MHz, $CDCl_3$) δ 205.4, 161.9, 161.0, 158.2, 157.8, 141.5, 138.5, 138.4, 135.5, 127.9, 113.8, 106.6, 106.0, 98.9, 96.5, 65.3, 55.8, 55.6, 55.3, 55.2, 50.8.

Further Transformations of Indanones

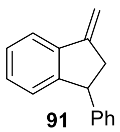

91

1-Methylene-3-phenyl-2,3-dihydro-1*H*-indene. To a solution of PPh_3MeBr (428.5 mg, 1.20 mmol) in THF (3 mL) was added NaH (60%, 48.0 mg, 1.20 mmol). The reaction mixture was refluxed for 30 min and then the 3-phenyl-2,3-dihydro-1*H*-inden-1-one **9** (83.2 mg, 0.40 mmol) in THF (3 mL) were added dropwise at 0 °C. The mixture was refluxed overnight. When the starting material was consumed (monitored by TLC), the reaction was diluted with PE. After filtration, the filtrate was condensed and the residue was purified by flash column chromatography on silica gel to obtain **91** (51.3 mg, 0.249 mmol, 62% yield) as a white solid. IR *ν* 3020, 2929, 1490, 1451 cm^{-1}; ^1H NMR (500 MHz, $CDCl_3$) δ 7.36-7.27 (m, 2H), 7.26-7.18 (m, 4H), 7.18-7.13 (m, 1H), 7.12-7.08 (m, 2H), 6.28-6.23 (m, 1H), 4.53 (s, 1H), 2.24-2.18 (m, 3H); ^{13}C NMR (126 MHz, $CDCl_3$) δ 148.7, 145.4, 140.1, 139.6, 134.5, 128.6, 127.7, 126.6, 125.2, 123.7, 119.0, 55.1, 12.9; HRMS (ESI-TOF) Calcd. for $C_{16}H_{15}$ $[M+H]^+$: 207.1174; Found 207.1174.

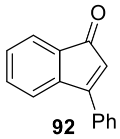

92

3-Phenyl-1*H*-inden-1-one (92).[39] To an oven-dried flask cooled under N_2 was added DDQ (137.4 mg, 0.60 mmol), 3-phenyl-2,3-dihydro-1*H*-inden-1-one **9** (83.0 mg, 0.40 mmol) and DCE (5 mL). The reaction mixture was stirred at 80 °C overnight. The reaction was diluted with DCM and filtered through a short pad of celite. The filtrate was condensed to give a crude product which was purified by flash column chromatography (PE/EA = 15/1) on silica gel to afford **92** (45.2 mg, 0.22 mmol, 55% yield) as a yellow oil. ^1H NMR (500 MHz, $CDCl_3$) δ 7.69-7.62 (m, 2H), 7.56-7.46 (m, 4H), 7.41-7.34 (m, 2H), 7.33-7.27 (m, 1H), 6.00 (s, 1H); ^{13}C NMR (126 MHz, $CDCl_3$) δ 197.0, 162.8, 143.9, 133.0, 132.8, 132.3, 130.5, 129.2, 128.9, 127.4, 123.0, 122.7, 121.6.

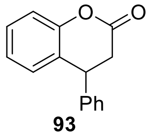

93

4-Phenylchroman-2-one (93).[21] To an oven-dried flask cooled under N_2 was added 3-phenyl-2,3-dihydro-1*H*-inden-1-one **9** (66.8 mg, 0.321 mmol), *m*CPBA (210.0 mg, 1.22 mmol), *p*TSA (13.8 mg, 0.072 mmol) and DCM (5 mL). The reaction mixture was refluxed overnight. The reaction was diluted quenched by Sat. $NaHCO_3$ and Sat. $Na_2S_2O_3$. Then the mixture was extracted with ethyl acetate. The combined organic layers were dried over anhydrous Na_2SO_4. After filtration, the filtrate was evaporated in *vacuo* and purified by flash column chromatography (PE/EA = 15/1) on silica gel to afford **93** (64.5 mg, 0.288 mmol, 96%) as a colorless oil. ^1H NMR (500 MHz, $CDCl_3$) δ 7.38-7.32 (m, 2H), 7.32-7.26 (m, 2H), 7.19-7.11 (m, 3H), 7.08 (dd, J = 7.5, 7.5 Hz, 1H), 6.98 (d, J = 7.5 Hz, 1H), 4.34 (dd, J = 7.0, 7.0 Hz, 1H), 3.12-2.96 (m, 2H).

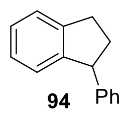

1-Phenyl-2,3-dihydro-1*H*-indene (94).[21] To an oven-dried flask cooled under N_2 was added 3-phenyl-2,3-dihydro-1*H*-inden-1-one **9** (62.5 mg, 0.30 mmol), Zn (941.6 mg, 14.40 mmol) and acetic acid (1.5 mL). The reaction was stirred at 100 °C overnight. After cooled at room temperature, the mixture was filtered through a short pad of celite. The filtration was condensed and purified by column chromatography (PE) on silica gel to afford **94** (55.0 mg, 0.283 mmol, 94% yield) as a colorless oil. ^{1}H NMR (500 MHz, $CDCl_3$) δ 7.32-7.24 (m, 3H), 7.22-7.13 (m, 4H), 7.11 (dd, J = 7.5, 7.0 Hz, 1H), 6.94 (d, J = 7.5 Hz, 1H), 4.32 (dd, J = 8.5, 8.0 Hz, 1H), 3.08-2.99 (m, 1H), 2.98-2.88 (m, 1H), 2.61-2.51 (m, 1H), 2.10-1.99 (m, 1H).

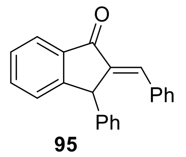

(*E*)-2-benzylidene-3-phenyl-2,3-dihydro-1*H*-inden-1-one (95). [40] 3-phenyl-2,3-dihydro-1*H*-inden-1-one **9** (83.5 mg, 0.40 mmol) and benzyaldehyde (54 μL, 0.52 mmol) were slowly added to a solution of aqueous NaOH (10%, 80 μL) and ethanol (2 mL) at 0°C. The reaction mixture was allowed to warm to room temperature and stirring continued. After 4h, EtOH was removed under *vacum*, reaction was quenched with water and extracted with EtOAc (3×10mL). Organic layers were combined and dried over anhydrous Na_2SO_4. The solvent was evaporated and the crude was purified by column chromatography (PE/EA = 10/1) on silica gel to yield **95** (101.0 mg, 0.341 mmol, 85% yield) as yellow solid. ^{1}H NMR (500 MHz, $CDCl_3$) δ 7.93 (d, J = 8.0 Hz, 1H), 7.85 (d, J = 1.5 Hz, 1H), 7.51 (dd, J = 7.5, 7.0 Hz, 1H), 7.48-7.42 (m, 2H), 7.41-7.33 (m, 2H), 7.26-7.18 (m, 7H), 7.15-7.09 (m, 1H), 5.34 (s, 1H).

Mechanistic Probes for Chemo- and Region-selectivity.

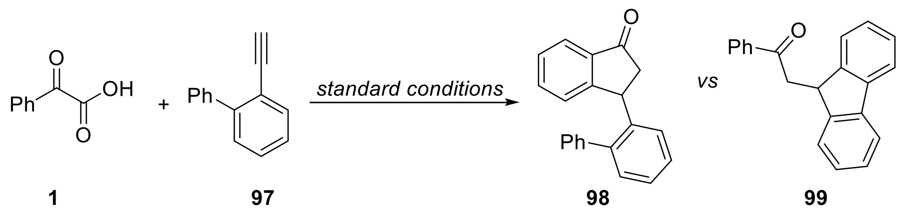

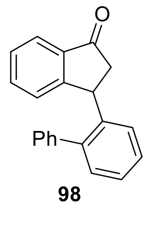

3-([1,1'-Biphenyl]-2-yl)-2,3-dihydro-1*H*-inden-1-one (98). Prepared according to the general procedure employing Ir[dF(CF_3)ppy]$_2$(phen)PF_6 (5.2 mg, 0.005 mmol), **1** (150.2 mg, 1.0 mmol), alkyne **97** (89.6 mg, 0.5 mmol), K_2HPO_4 (208.9 mg, 1.2 mmol), MeCN (19 mL) and H_2O (1 mL). After 24 h, the reaction mixture was cooled to rt and filtered through a short pad of silica using EA. The filtrate was concentrated in *vacuo* before it was purified by flash chromatography (PE/EA = 10/1) on silica gel to afford **98 (**59.1 mg, 0.208

mmol, 42% yield) as a yellow solid. mp = 125-127 °C. IR *v* 2922, 1713, 1598 cm^{-1}; ^1H NMR (500 MHz, $CDCl_3$) δ 7.76 (d, *J* = 7.5 Hz, 1H), 7.57-7.50 (m, 1H), 7.47-7.41 (m, 2H), 7.41-7.34 (m, 4H), 7.32 -7.24 (m, 3H), 7.20 (d, *J* = 7.5 Hz, 1H), 6.89-6.84 (m, 1H), 4.70 (dd, *J* = 7.5, 4.0 Hz, 1H), 3.05 (dd, *J* = 19.0, 8.0 Hz, 1H), 2.66 (dd, *J* = 19.5, 4.5 Hz, 1H); ^{13}C NMR (126 MHz, $CDCl_3$) δ 206.0, 158.6, 142.3, 141.5, 141.2, 136.9, 135.0, 129.9, 129.3, 128.3, 128.2, 127.6, 127.3, 127.2, 126.8, 126.4, 123.2, 47.6, 40.6; HRMS (ESI-TOF) Calcd. for $C_{21}H_{17}O$ $[M+H]^+$: 285.1279; Found 285.1282.

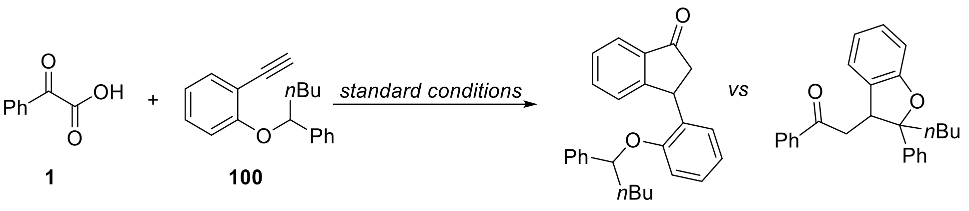

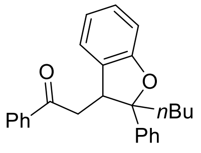

102

2-(2-Butyl-2-phenyl-2,3-dihydrobenzofuran-3-yl)-1-phenylethan-1-one (102). Prepared according to the general procedure employing Ir[dF(CF_3)ppy]$_2$(phen)PF_6 (5.1 mg, 0.005 mmol), **1** (150.1 mg, 1.0 mmol), alkyne **100** (104.2 mg, 0.394 mmol), K_2HPO_4 (209.0 mg, 1.2 mmol), MeCN (19 mL) and H_2O (1 mL). After 24 h, the reaction mixture was cooled to rt and filtered through a short pad of silica using EA. The filtrate was concentrated in *vacuo* before it was purified by flash chromatography (PE/EA = 15/1) on silica gel to afford **102** (108.6 mg, 0.293 mmol, 74% yield, 1.3/1 *dr*) as a yellow oil. IR *v* 2955, 2932, 1685, 1596, 1479 cm^{-1}; ^1H NMR (500 MHz, $CDCl_3$) δ 7.97 (d, *J* = 8.0 Hz, 0.86 H), 7.59-7.49 (m, 2.51H), 7.44 (dd, *J* = 8.0, 7.5 Hz, 0.85 H), 7.42-7.34 (m, 1.87H), 7.33-7.27 (m, 1H), 7.25-7.05 (m, 4.53H), 7.00 (d, *J* = 7.5 Hz, 0.48H), 6.93 (d, *J* = 8.0 Hz, 1H), 6.82 (dd, *J* = 7.5, 7.0 Hz, 0.59H), 6.76 (dd, *J* = 7.5, 7.5 Hz, 0.43H), 4.29 (dd, *J* = 7.0, 6.5 Hz, 0.41H), 4.21 (dd, *J* = 7.0, 6.5 Hz, 0.59H), 3.63 (dd, *J* = 18.0, 7.5 Hz, 0.41H), 3.15 (dd, *J* = 18.0, 6.5 Hz, 0.41H), 2.78 (dd, *J* = 17.5, 7.0 Hz, 0.59H), 2.66 (dd, *J* = 18.0, 6.5 Hz, 0.59H), 2.25-2.08 (m, 1.18H), 2.00-1.87 (m, 0.89H), 1.52-1.38 (m, 1H), 1.33-1.06 (m, 3H), 0.80 (t, *J* = 7.0 Hz, 1.70H), 0.74 (t, *J* = 7.0 Hz, 1.30H); ^{13}C NMR (126 MHz, $CDCl_3$) δ 198.6, 198.5, 158.2, 158.1, 145.5, 140.6, 136.75, 136.74, 133.2, 132.7, 131.1, 130.5, 128.6, 128.5, 128.4, 128.15, 128.15, 128.02, 127.97, 127.6, 126.9, 126.7, 126.2, 125.3, 125.1, 124.9, 120.8, 120.5, 109.7, 109.5, 94.4, 93.3, 48.9, 47.6, 42.5, 41.1, 40.1, 36.1, 26.1, 25.9, 22.9, 22.8, 13.9, 13.8; HRMS (ESI-TOF) Calcd. for $C_{26}H_{27}O_2$ $[M+H]^+$: 371.2011; Found 371.2006.

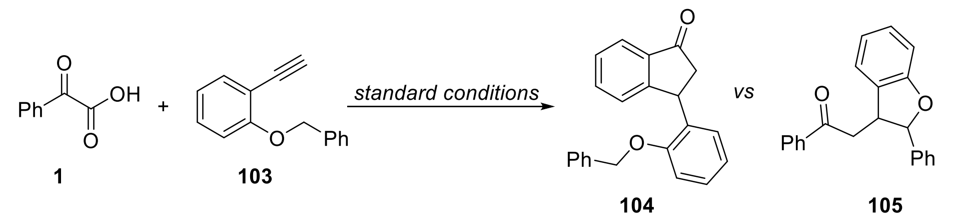

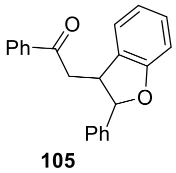

1-Phenyl-2-(2-phenyl-2,3-dihydrobenzofuran-3-yl)ethan-1-one (105). Prepared according to the general procedure employing Ir[dF(CF_3)ppy]$_2$(phen)PF_6 (5.2 mg, 0.005 mmol), **1** (150.1 mg, 1.0 mmol), alkyne **103** (104.1 mg, 0.50 mmol), K_2HPO_4 (209.0 mg, 1.2 mmol), MeCN (19 mL) and H_2O (1 mL). After 24 h, the reaction mixture was cooled to rt and filtered through a short pad of silica using EA. The filtrate was concentrated in *vacuo* before it was purified by flash chromatography (PE/EA = 15/1) on silica gel to afford indanone **105 (**78.6 mg, 0.25 mmol, 50% yield, 1.5/1 *dr*) as a yellow oil. IR *v* 2955, 2924, 1684, 1596, 1452 cm^{-1}; ^{1}H NMR (500 MHz, $CDCl_3$) δ 8.03-7.94 (m, 1H), 7.63-7.55 (m, 1.50H), 7.50-7.42 (m, 2.50H), 7.37-7.27 (m, 3H), 7.25-7.12 (m, 4H), 6.93 (dd, *J* = 8.0, 4.5 Hz, 1H), 6.88 (t, *J* = 7.5 Hz, 1H), 5.99 (d, *J* = 9.0 Hz, 0.6H), 5.43 (d, *J* = 5.0 Hz, 0.4H), 4.54-4.46 (m, 0.6H), 4.11-4.04 (m, 0.4H), 3.47 (dd, *J* = 18.0, 2.0 Hz, 0.4H), 3.42 (dd, *J* = 13.5, 3.0 Hz, 0.4H), 2.94 (dd, *J* = 18.0, 6.5 Hz, 0.6H), 2.85 (dd, *J* = 17.5, 7.5 Hz, 0.6H); ^{13}C NMR (126 MHz, $CDCl_3$) δ 198.4, 198.0, 159.5, 159.4, 141.2, 137.6, 136.7, 136.6, 133.4, 132.9, 130.3, 129.4, 128.8, 128.7, 128.6, 128.5, 128.28, 128.26, 128.03, 128.01, 127.9, 127.7, 126.6, 125.9, 124.90, 124.87, 121.0, 120.9, 109.6, 109.5, 89.4, 87.0, 46.2, 44.5, 41.7, 40.6; HRMS (ESI-TOF) Calcd. for $C_{22}H_{19}O_2$ [M+H]$^+$: 315.1385; Found 315.1386.

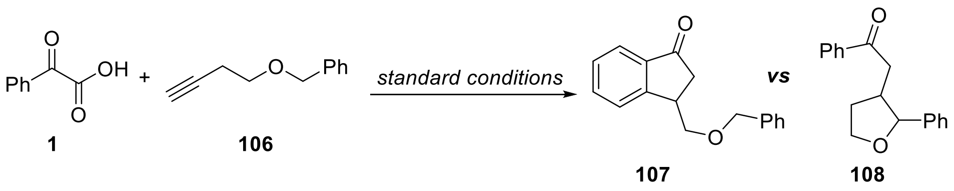

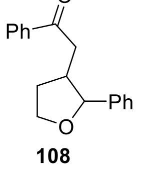

1-Phenyl-2-(2-phenyltetrahydrofuran-3-yl)ethan-1-one (108). Prepared according to the general procedure employing Ir[dF(CF_3)ppy]$_2$(phen)PF_6 (5.5 mg, 0.005 mmol), **1** (150.4 mg, 1.0 mmol), alkyne **106** (79.2 mg, 0.494 mmol), K_2HPO_4 (209.0 mg, 1.2 mmol), MeCN (19 mL) and H_2O (1 mL). After 24 h, the reaction mixture was cooled to rt and filtered through a short pad of silica using EA. The filtrate was

concentrated in *vacuo* before it was purified by flash chromatography (PE/EA = 15/1) on silica gel to afford **108** (36.1 mg, 0.136 mmol, 27% yield, 9/1 *dr*) as a yellow oil. IR *v* 2918, 1686, 1597, 1211 cm^{-1}; ^{1}H NMR (500 MHz, $CDCl_3$) δ 7.88 (d, *J* = 7.5 Hz, 2H), 7.55 (dd, *J* = 7.5, 7.5 Hz, 1H), 7.43 (dd, *J* = 8.0, 7.5 Hz, 2H), 7.41-7.37 (m, 2H), 7.35 (dd, *J* = 7.5, 7.5 Hz, 2H), 7.30-7.26 (m, 1H), 4.54 (d, *J* = 7.5 Hz, 1H), 4.18 (dd, *J* = 15.5, 7.5 Hz, 1H), 4.10-4.01 (m, 1H), 3.20 (dd, *J* = 17.0, 5.0 Hz, 1H), 3.01 (dd, *J* = 17.0, 9.5 Hz, 1H), 2.75-2.63 (m, 1H), 2.48-2.37 (m, 1H), 1.80-1.66 (m, 1H); ^{13}C NMR (126 MHz, $CDCl_3$) δ 198.7, 141.5, 136.8, 133.1, 128.6, 128.4, 128.0, 127.7, 126.3, 85.9, 68.0, 43.7, 40.8, 32.4; HRMS (ESI-TOF) Calcd. for $C_{18}H_{18}O_2Na$ $[M+Na]^+$: 289.1204; Found 289.1204.

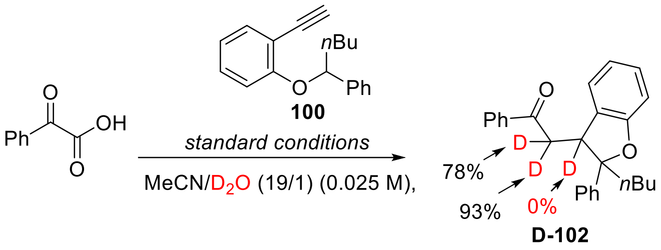

Prepared according to the general procedure employing $Ir[dF(CF_3)ppy]_2(phen)PF_6$ (5.0 mg, 0.005 mmol), **1** (150.3 mg, 1.0 mmol), alkyne **100** (104.1 mg, 0.394 mmol), K_2HPO_4 (209.5 mg, 1.2 mmol), MeCN (19 mL) and D_2O (1 mL). After 24 h, the reaction mixture was cooled to rt and filtered through a short pad of silica using EA. The filtrate was concentrated in *vacuo* before it was purified by flash chromatography (PE/EA = 15/1) on silica gel to afford **D-102** (105.7 mg, 0.285 mmol, 72% yield, 1.4/1 *dr*) as a yellow oil (**See Supplementary Fig. 1**). The result indicated that the corresponding product was formed through an intramolecular 1,5-HAT followed by a Giese addition, which is not a water-mediated pathway.

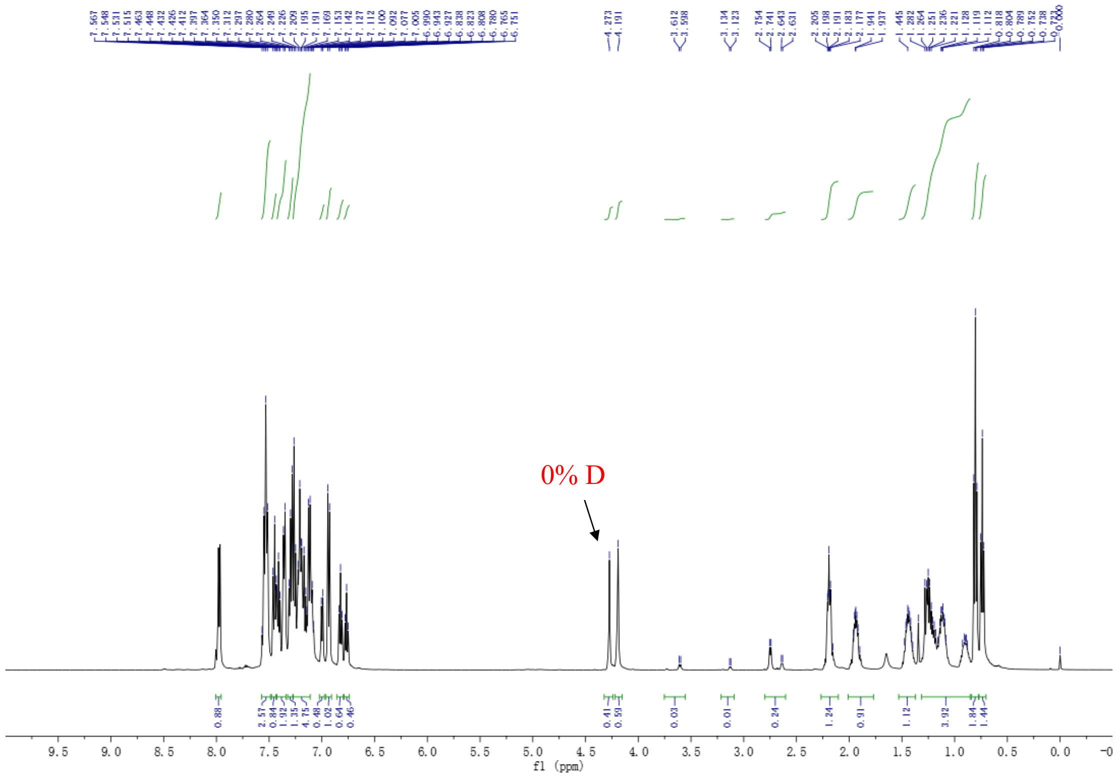

Supplementary Fig. 1. ^{1}H NMR spectrum of indanone **D-102**

II. Supplementary Discussion

Control Experiments

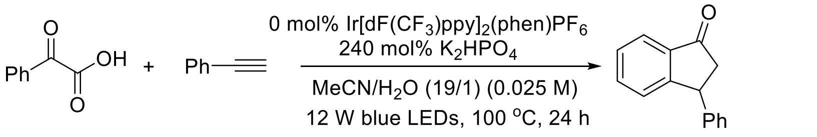

1 **4** ***9***, 0% nmr yield

To an oven-dried 50 mL flask, acid **1** (153.7 mg, 1.0 mmol) and K_2HPO_4 (210.2 mg, 1.2 mmol) were added sequentially under N_2 atmosphere. The flask was evaculated and back-filled with N_2 for three times, then alkyne **4** (55 μL, 0.5 mmol), H_2O (1 mL) and MeCN (19 mL) was added. The reaction mixture was irradiated by 12W blue LEDS at a distance of 5 cm for 24 h at 100 ^{o}C. The reaction mixture was cooled to rt and filtered through a short pad of silica using EA. The filtrate was concentrated in *vacuo*. The crude mixture was monitored by ^{1}H NMR (0% NMR yield) with mesitylene as an internal standard.

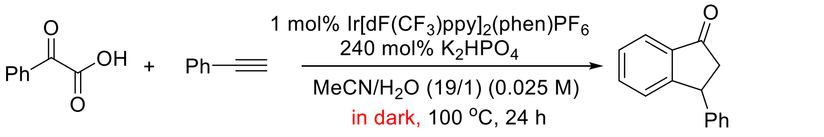

1 **4** ***9***, 0% nmr yield

To an oven-dried 50 mL flask, Ir[dF(CF_3)ppy]$_2$(phen)PF_6 (5.1 mg, 0.0059 mmol), acid **1** (153.6 mg, 1.0 mmol) and K_2HPO_4 (210.3 mg, 1.2 mmol) were added sequentially under N_2. The flask was evacuated and back-filled with N_2 for three times, then alkyne **4** (55 µL, 0.5 mmol), H_2O (1 mL) and MeCN (19 mL) was added. The reaction mixture was stirred without light at 100 °C for 24 h. The reaction mixture was cooled to rt and filtered through a short pad of silica using EA. The filtrate was concentrated in *vacuo*. The crude mixture was monitored by ^{1}H NMR (0% NMR yield) with mesitylene as an internal standard.

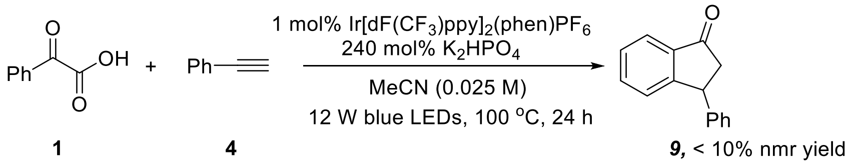

To an oven-dried 50 mL flask, Ir[dF(CF_3)ppy]$_2$(phen)PF_6 (5.3 mg, 0.0059 mmol), acid **1** (153.6 mg, 1.0 mmol) and K_2HPO_4 (210.2 mg, 1.2 mmol) were added sequentially under N_2 atmosphere. The flask was evacuated and back-filled with N_2 for three times, then alkyne **4** (55 µL, 0.5 mmol), and MeCN (19 mL) was added. The reaction mixture was irradiated by 12W blue LEDS at a distance of 5 cm for 24 h at 100 °C. The reaction mixture was cooled to rt and filtered through a short pad of silica using EA. The filtrate was concentrated in *vacuo*. The crude mixture was monitored by ^{1}H NMR (<10% NMR yield) with mesitylene as an internal standard.

Supplementary Mechanistic Studies

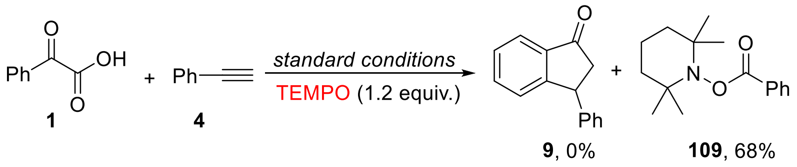

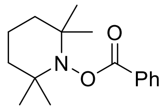

109

2,2,6,6-Tetramethylpiperidin-1-yl benzoate (109).[41] To a 50 mL flask, Ir[dF(CF_3)ppy]$_2$(phen)PF_6 (0.005 mmol), **1** (150.0 mg, 1.0 mmol), K_2HPO_4 (209.2 mg, 1.2 mmol) and TEMPO (93.6 mg, 0.6 mmol) were added sequentially under N_2. The flask was evacuated and back-filled with N_2 for three times, then phenylacetylene **4** (55 µL, 0.5 mmol), H_2O (1 mL) and MeCN (19 mL) was added. The reaction mixture was irradiated by 12 W blue LEDS at a distance of 5 cm for 24 h at 100 °C. The reaction mixture was cooled to rt and filtered through a short pad of silica using EA. The filtrate was concentrated in *vacuo* before it was purified by flash chromatography (PE/EA = 15/1) on silica gel

to afford **109** (106.0 mg, 0.406 mmol, 68% yield) as a white solid. ^{1}H NMR (500 MHz, $CDCl_3$) δ 8.08 (d, J = 7.5 Hz, 2H), 7.56 (dd, J = 7.5, 7.0 Hz, 1H), 7.46 (dd, J = 8.0, 7.5 Hz, 2H), 1.83-1.40(m, 6H), 1.28 (s, 6H), 1.12 (s, 6H); ^{13}C NMR (126 MHz, $CDCl_3$) δ 166.2, 132.7, 129.6, 129.4, 128.3, 60.2, 38.9, 31.8, 20.7, 16.9.

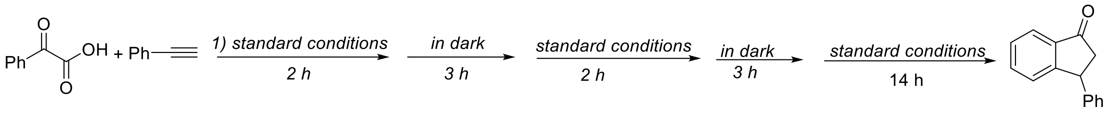

To a 50 mL flask, Ir[dF(CF_3)ppy]$_2$(phen)PF_6 (0.005 mmol), **1** (150.0 mg, 1.0 mmol), and K_2HPO_4 (209.2 mg, 1.2 mmol) were added sequentially under N_2. The flask was evaculated and back-filled with N_2 for three times, then phenylacetylene (55 µL, 0.5 mmol), H_2O (1 mL), acetophenone (36.0 mg, 0.3 mmol) and MeCN (19 mL) was added. The reaction mixture was irradiated by 12 W blue LEDS at a distance of 5 cm for 2 h at 100 °C, 3 hours without light, 2 h with LEDs, 3 h without light and 14 h with LEDs, sequentially. The reaction yield in different stage was determined by ^{1}H NMR analysis with acetophenone as internal standard. (**See Supplementary Fig. 2**.)

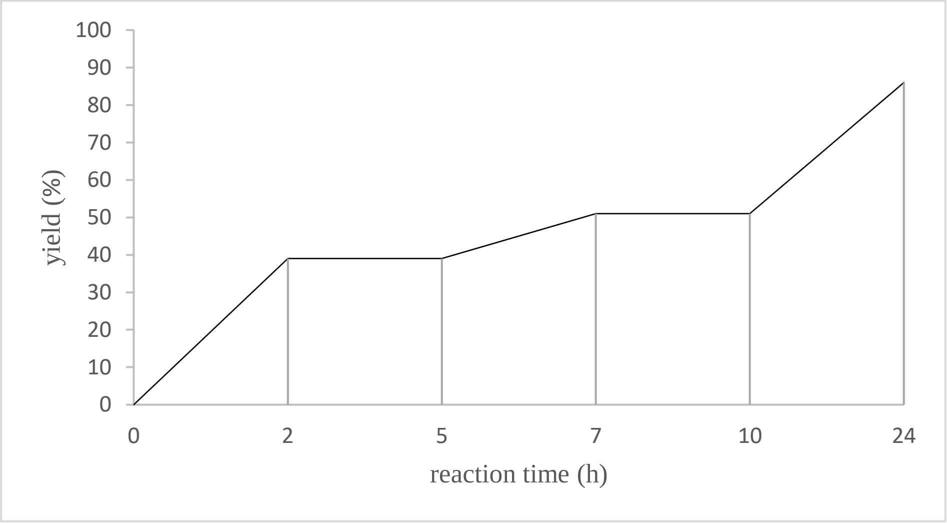

Supplementary Fig. 2. Light-on-off experiment

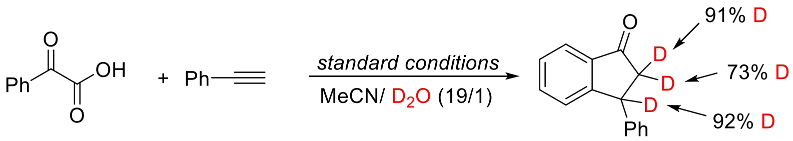

1 **4** **110**, 90% yield

To a 50 mL flask, Ir[dF(CF_3)ppy]$_2$(phen)PF_6 (5.4 mg, 0.005 mmol), **1** (151.0 mg, 1.0 mmol), and K_2HPO_4 (209.2 mg, 1.2 mmol) were added sequentially under N_2. The flask was evaculated and back-filled with N_2 for three times, then phenylacetylene **4** (55 µL, 0.5 mmol), D_2O (1 mL) and MeCN (19 mL) was added. The reaction mixture was irradiated by 12 W blue LEDS at a distance

of 5 cm for 24 h at 100 °C. The reaction mixture was cooled to rt and filtered through a short pad of silica using EA. The filtrate was concentrated in *vacuo* before it was purified by flash chromatography (PE/EA = 15/1) on silica gel to afford indanone **110** (95.5 mg, 0.456 mmol, 90% yield) as a yellow oil (**See Supplementary Fig. 3**).

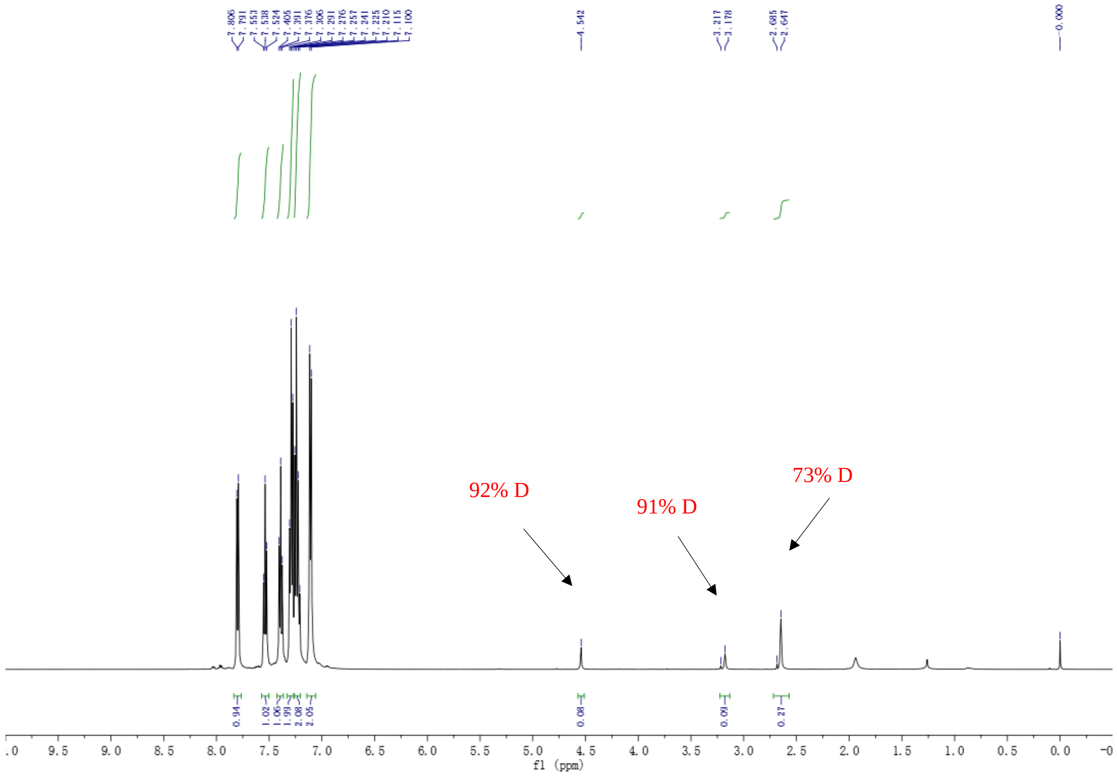

Supplementary Fig. 3. ^{1}H NMR spectrum of indanone **110**

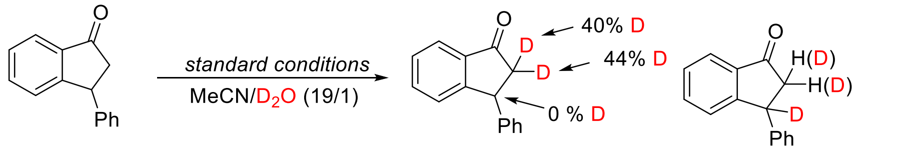

9 **111**, ***94%*** **S30**, ***not observed***

To a 50 mL flask, Ir[dF(CF_3)ppy]$_2$(phen)PF_6 (5.4 mg, 0.005 mmol), indanone **9** (104.1 mg, 0.5 mmol), and K_2HPO_4 (209.0 mg, 1.2 mmol) were added sequentially under N_2. The flask was evacuated and back-filled with N_2 for three times, then H_2O (1 mL) and MeCN (19 mL) was added. The reaction mixture was irradiated by 12 W blue LEDS at a distance of 5 cm for 24 h at 100 °C. The reaction mixture was cooled to rt and filtered through a short pad of silica using EA. The filtrate was concentrated in *vacuo* before it was purified by flash chromatography (PE/EA = 15/1) on silica gel to afford **111** (97.4 mg, 94% yield) as a yellow oil with no **S30** observed (**See Supplementary**

Fig. 4).

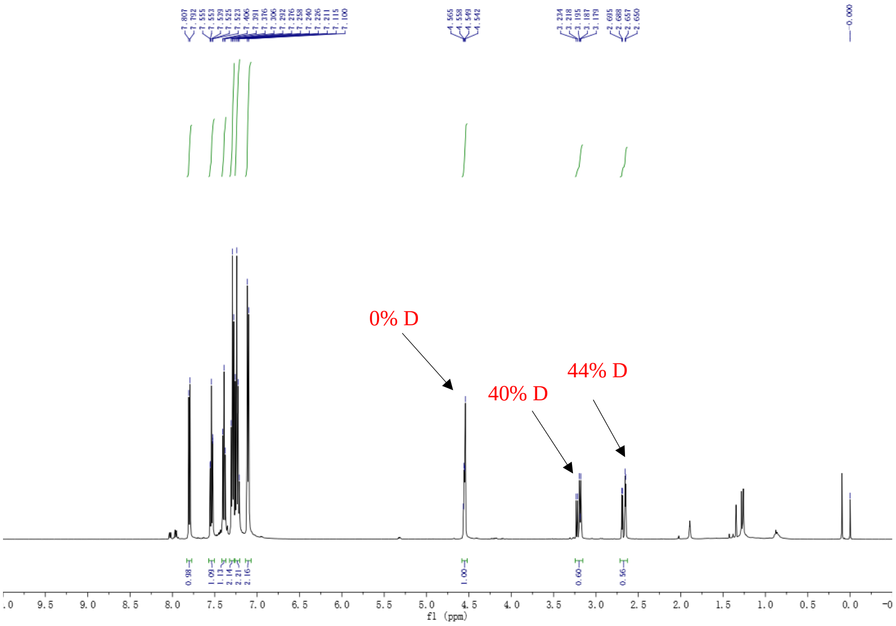

Supplementary Fig. 4. ^{1}H NMR spectrum of indanone **111**

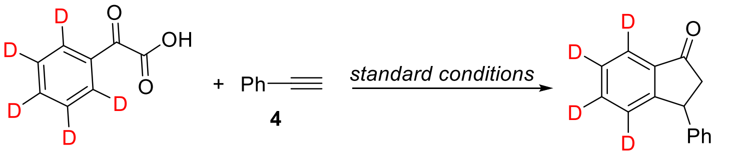

To a 50 mL flask, Ir[dF(CF_3)ppy]$_2$(phen)PF_6 (5.4 mg, 0.005 mmol), deuterated phenylglyoxylic acid **112** (150.0 mg, 1.0 mmol), and K_2HPO_4 (209.2 mg, 1.2 mmol) were added sequentially under N_2. The flask was evaculated and back-filled with N_2 for three times, then phenylacetylene **4** (55 μL, 0.5 mmol), H_2O (1 mL) and MeCN (19 mL) was added. The reaction mixture was irradiated by 12 W blue LEDS at a distance of 5 cm for 24 h at 100 ºC. The reaction mixture was cooled to rt and filtered through a short pad of silica using EA. The filtrate was concentrated in *vacuo* before it was purified by flash chromatography (PE/EA = 15/1) on silica gel to afford indanone **113** (91.5 mg, 0.431 mmol, 86% yield) as a yellow oil (**See Supplementary Fig. 5**).

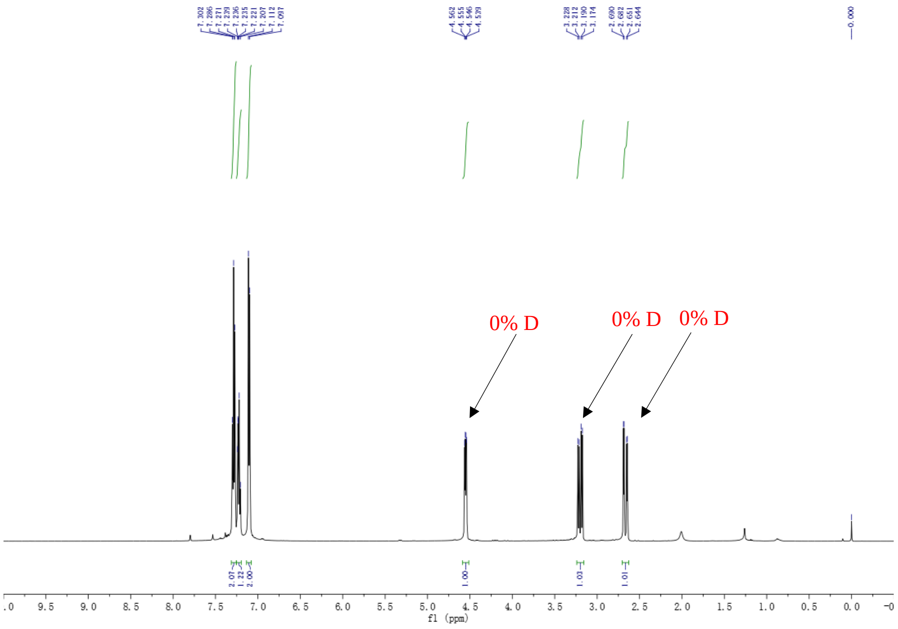

Supplementary Fig. 5. ^{1}H NMR spectrum of indanone **113**

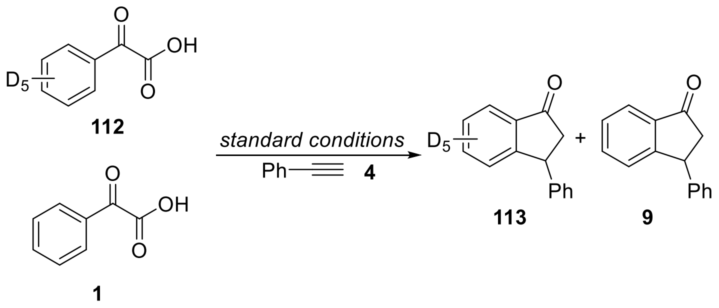

To a 50 mL flask, Ir[dF(CF$_3$)ppy]$_2$(phen)PF$_6$ (5.4 mg, 0.005 mmol), 2-oxo-2-phenylacetate **1** (150.4 mg, 1.0 mmol), deuterated phenylglyoxylic acid **112** (155.0 mg, 1.0 mmol) and K_2HPO_4 (389.0 mg, 2.23 mmol) were added sequentially under N_2. The flask was evaculated and back-filled with N_2 for three times, then phenylacetylene **4** (55 μL, 0.5 mmol), H_2O (1 mL) and MeCN (19 mL) was added. The reaction mixture was irradiated by 12 W blue LEDS at a distance of 5 cm for 24 h at 100 °C. The reaction mixture was cooled to rt and filtered through a short pad of silica using EA. The filtrate was concentrated in *vacuo* before it was purified by flash chromatography (PE/EA = 15/1) on silica gel to afford a mixture of indanone **113** and **9** (total: 82.4 mg, 0.392 mmol, 79% yield) as a yellow oil. The ratio of indanone **112** and **9** (1/1) was determined by ^{1}H NMR analysis (**See Supplementary Fig. 6**).

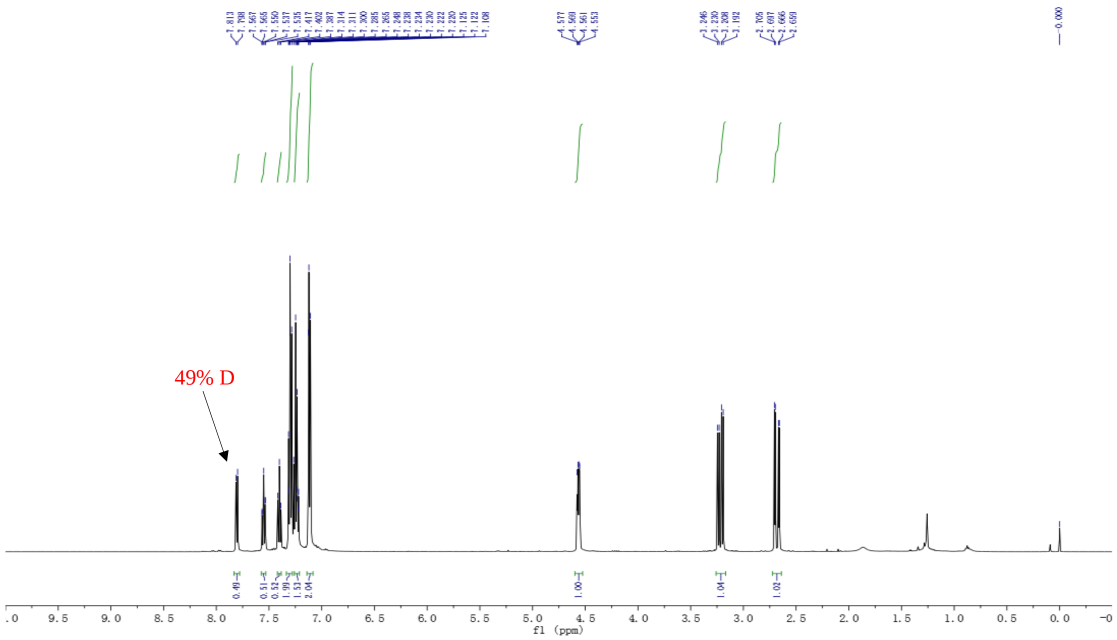

Supplementary Fig. 6. ^{1}H NMR spectrum of indanone mixture **113** and **9**

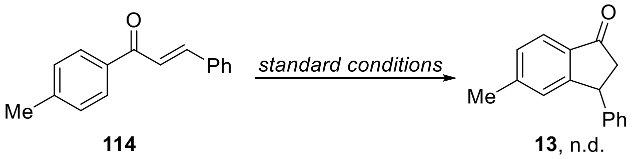

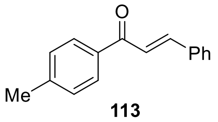

113

(*E*)-3-phenyl-1-(*p*-tolyl)prop-2-en-1-one (113). Prepared by Aldol reaction of the corresponding ketone and aldehyde according to the previously reported procedures.[42] To a stirred solution of benzaldehyde (2.23 mL, 21.9 mmol) in EtOH/H_2O (30 mL/20 mL) was added 4-methylacetophenone (2.67 mL, 20.0 mmol). After cooled to 0 °C, NaOH (1 N) (10 mL) was added slowly, Then the reaction was stirred overnight at rt. The mixture was diluted with H_2O, treated with HCl (1 N) and extracted with DCM. The combined organic layers were dried over anhydrous Na_2SO_4 and filtered. The filtration was condensed and purified through column chromatography (PE/EA = 15/1) to afford chalcone **114** (2.6674 g, 12.0 mmol, 60% yield) as a light yellow solid. The ^{1}H NMR is in accordance to the literature.[42]

To a 50 mL flask, Ir[dF(CF_3)ppy]$_2$(phen)PF_6 (5.3 mg, 0.005 mmol), **114** (112.2 mg, 0.5 mmol) and K_2HPO_4 (210.0 mg, 1.20 mmol) were added sequentially under N_2. The flask was evaculated and back-filled with N_2 for three times, then H_2O (1 mL) and MeCN (19 mL) was added. The reaction mixture was irradiated by 12 W blue LEDs at a distance of 5 cm for 24 h at 100 °C. The reaction mixture was cooled to rt and filtered through a short pad of silica using EA. The filtrate

was concentrated in *vacuo* and monitored through ^{1}H NMR analysis with mesitylene as an internal standard. The results showed that 96% recovery of **114** was detected with no cyclized product **13** observed.

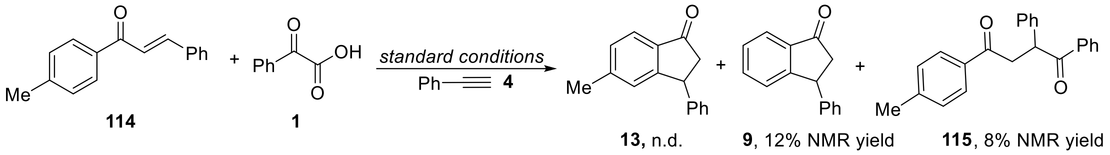

To a 50 mL flask, Ir[dF(CF_3)ppy]$_2$(phen)PF_6 (5.3 mg, 0.005 mmol), 2-oxo-2-phenylacetate **1** (89.9 mg, 0.60 mmol), chalcone **114** (110.0 mg, 0.5 mmol) and K_2HPO_4 (389.0 mg, 2.23 mmol) were added sequentially under N_2. The flask was evacuated and back-filled with N_2 for three times, then phenylacetylene **4** (55 μL, 0.5 mmol), H_2O (1.0 mL) and MeCN (19 mL) was added. The reaction mixture was irradiated by 12 W blue LEDS at a distance of 5 cm for 24 h at 100 ^{o}C. The reaction mixture was cooled to rt and filtered through a short pad of silica using EA. The filtrate was concentrated in *vacuo* before it was purified by flash chromatography (PE/EA = 15/1) on silica gel to afford a crude mixture. The mixture was analyzed by ^{1}H NMR, indicating only indanone **9** and ketone **115** could be detected (**See Supplementary Fig. 7**).

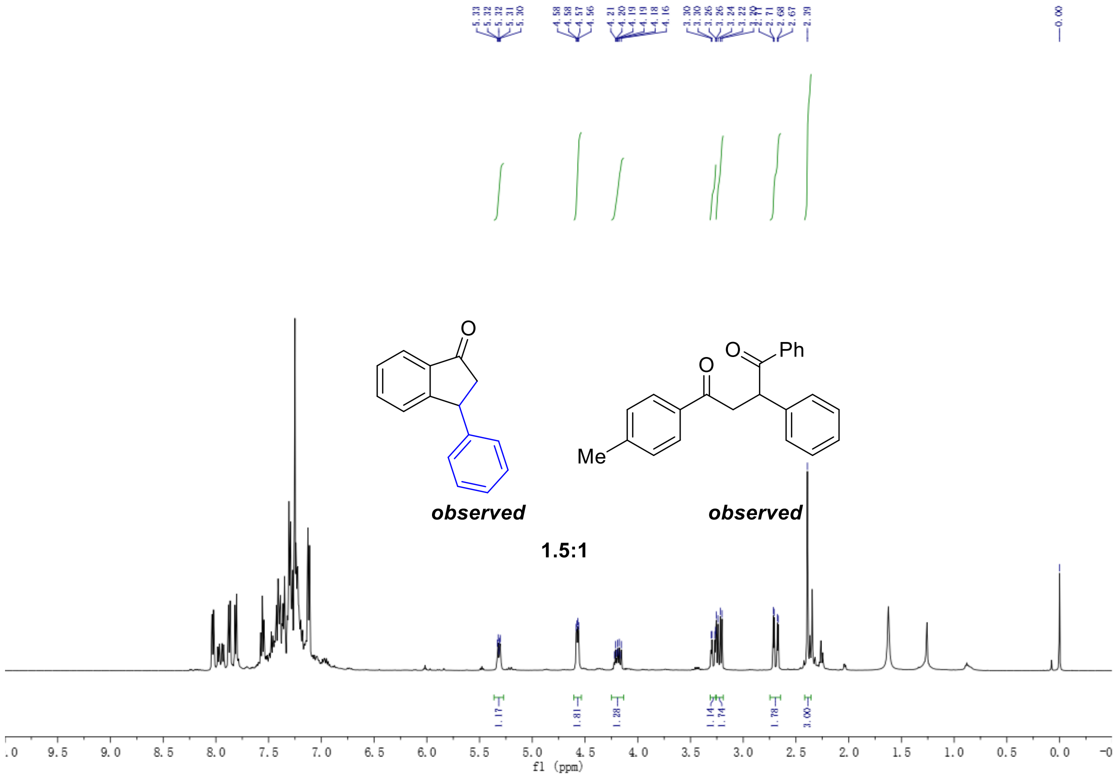

Supplementary Fig. 7. ^{1}H NMR spectrum of indanone mixture **114** and **9**

DFT Calculations

All the calculations in this study were performed using the Gaussian 16 program package[43]. The All the geometries were optimized at the M062X[44]/6-31G(d, p) and SDD for Ir level, and the solvent effect was utilized the polarizable continuum model using integral equation formalism model (IEFPCM) in hexane solvent.[45,46] All the optimized stationary points had been identified as minima (zero imaginary frequencies) and transition states (one imaginary frequency), via the vibrational analysis. The solution-translational entropy correction has been calculated with THERMO program.[47] Furthermore, TD-DFT study at the same level within the adiabatic approximation to predict the excitation energies was conducted.

The excitation of Ir(III) photocatalyst by visible light would provide a Sn excited state species II′. TD-DFT study were conducted at same level within the adiabatic approximation to predict the excitation energies. The calculated minimum absorption wave length of visible light is 317 nm, which is agreed with experimental conditions.

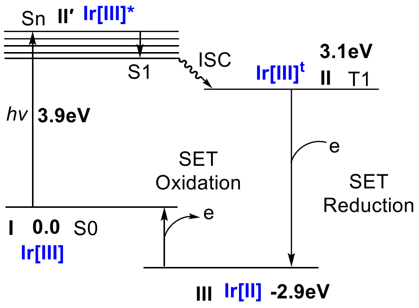

Supplementary Fig. 8. The photoredox process for the Ir[III] photocatalyst

In order to explore the influence of different water molecules assisted 1,3-hydrogen transfer process, three pathways with one, two and three water molecules have been performed and added in supplementary information, as shown in **Supplementary Fig.9**. For one water molecule promoted process, the free energy barrier would be 7.2 kcal/mol due to lack of the possible hydrogen with the indenone moiety, which would be higher than that of two water assisted process for 4.8 kcal/mol. Furthermore, the additional water could make the water chain bending, leading to the weak hydrogen bond. As shown in the **Supplementary Fig.9**, the length of hydrogen bond between terminal water and indenone moiety would be 2.30 and 2.45 Å, respectively for the **TS3** and **TS3-3H$_2$O**. The calculated results clearly show the hydrogen bond would be weakened by the additional

water. Moreover, the free energy barrier for the **TS3-3H$_2$O** would be higher than that of **TS3** for 1.9 kcal/mol. Hence, the two water-assisted 1,3-hydrogen transfer pathway would be most possible for the reaction.

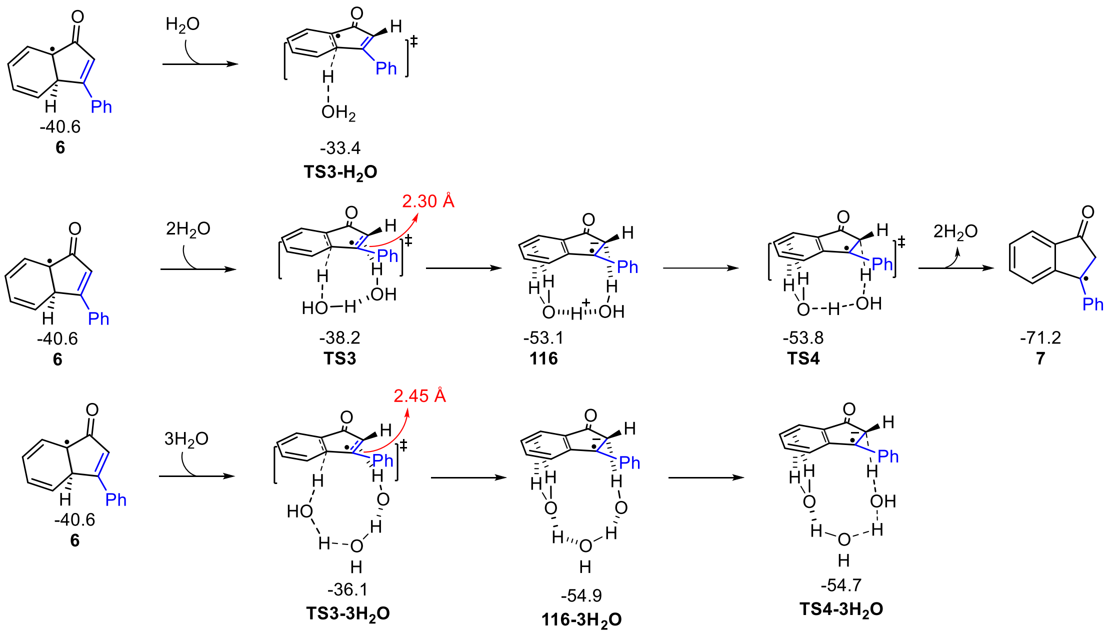

Supplementary Fig. 9. The free energies for the 1, 2 and 3 water assisted 1,3-hydrogen transfer process. (unit for the free energy: kcal/mol; unit for the length: Å)

Supplementary Table 8. The Gibbs free energy for the Ir compound in this study.

Species	Gibbs free energy	Species	Gibbs free energy
I	-2941.00332	III	-2941.11135
II	-2940.89090		

For the absorption calculation:

Excited State 1: Singlet-A 3.9077 eV 317.28 nm f=0.1894 <S**2>=0.000

209 -> 213 0.21184

210 -> 212 0.62873

This state for optimization and/or second-order correction.

Total Energy, E(TD-HF/TD-KS) = -2941.46677182

For the S1 calculation:

Excited State 1: Singlet-A 3.2829 eV 377.66 nm f=0.2174 <S**2>=0.000

210 -> 211 -0.66184

210 -> 219 -0.10412

This state for optimization and/or second-order correction.

Total Energy, E(TD-HF/TD-KS) = -2941.48338773

Supplementary Table 9. The free energy for the species in the reaction.

**2**	-533.37974	**CO_2**	-188.50915
**3**	-344.68964	**4**	-308.17685
**TS1**	-652.8682	**5**	-652.91783
**TS2**	-652.89284	**6**	-652.93125
**TS3**	-805.68596	**$2H_2O$**	-152.75858
**116**	-805.70972	**TS4**	-805.7108
**7**	-652.97987	**8**	-653.09907
**TS4′**	-805.70144	**7′**	-652.96348
**TS2″**	-652.89084	**6″**	-652.90547
**TS3″**	-652.8864	**TS3-H_2O**	-729.29428
**$(H_2O)_3$**	-229.14504	**TS3-$3H_2O$**	-882.06902
**116-$3H_2O$**	-882.09907	**TS4-$3H_2O$**	-882.09866

The coordination for all the structures involved in the calculation was provided in supplementary data file.

Supplementary NMR Spectra

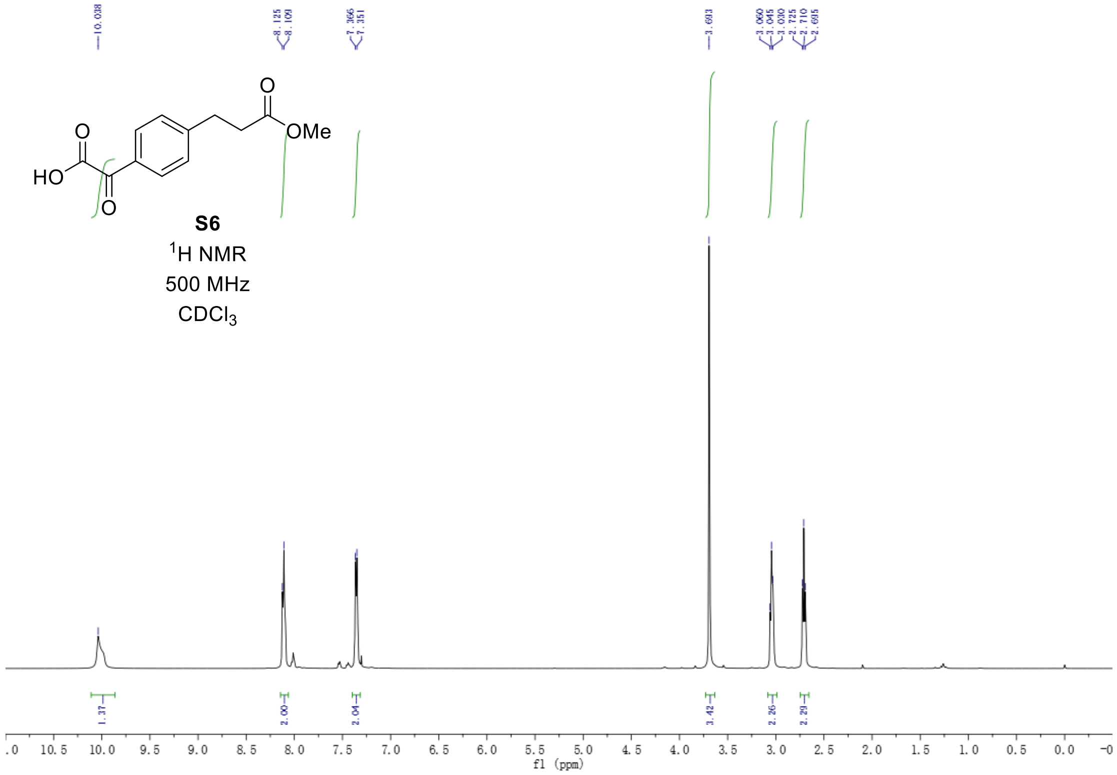

Supplementary Fig. 10 ^{1}H NMR spectrum of compound **S6**

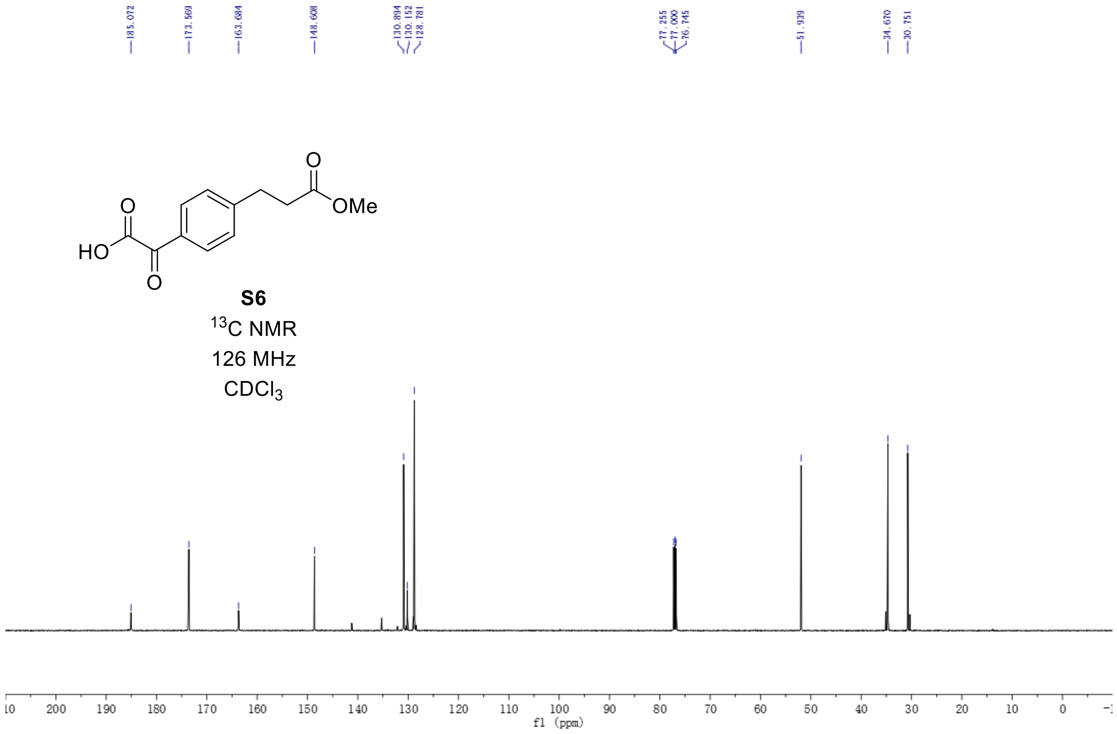

Supplementary Fig. 11 ^{13}C NMR spectrum of compound **S6**

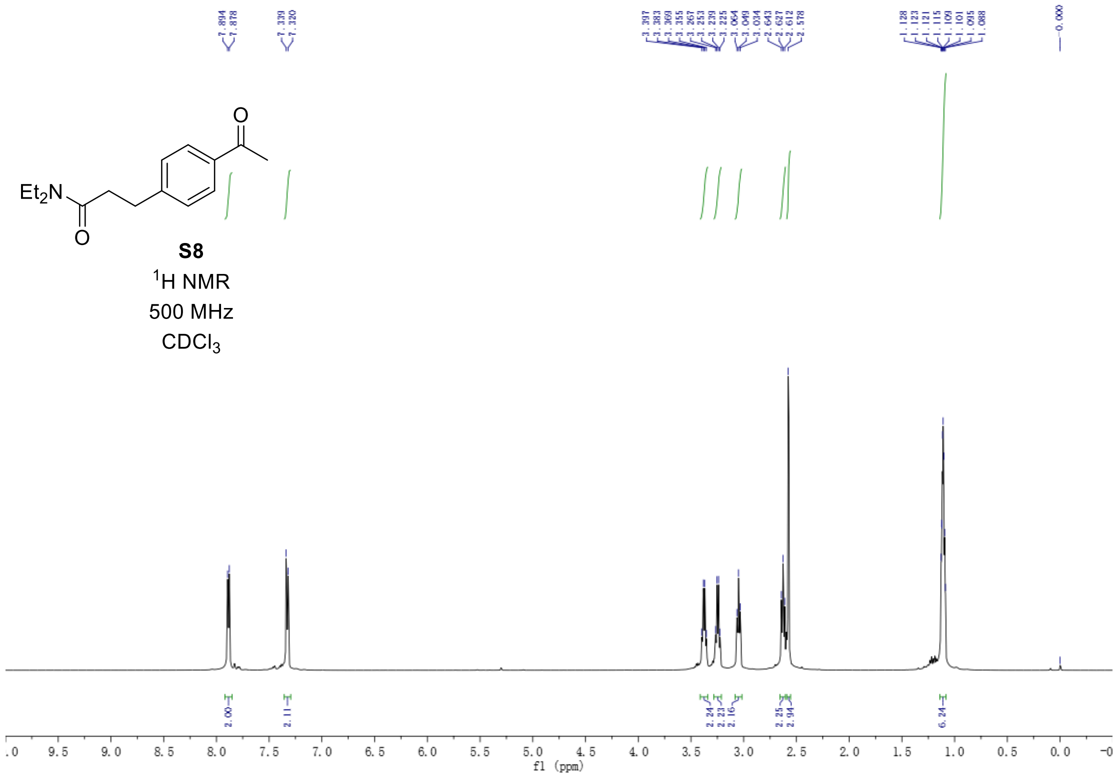

Supplementary Fig. 12 ^{1}H NMR spectrum of compound **S8**

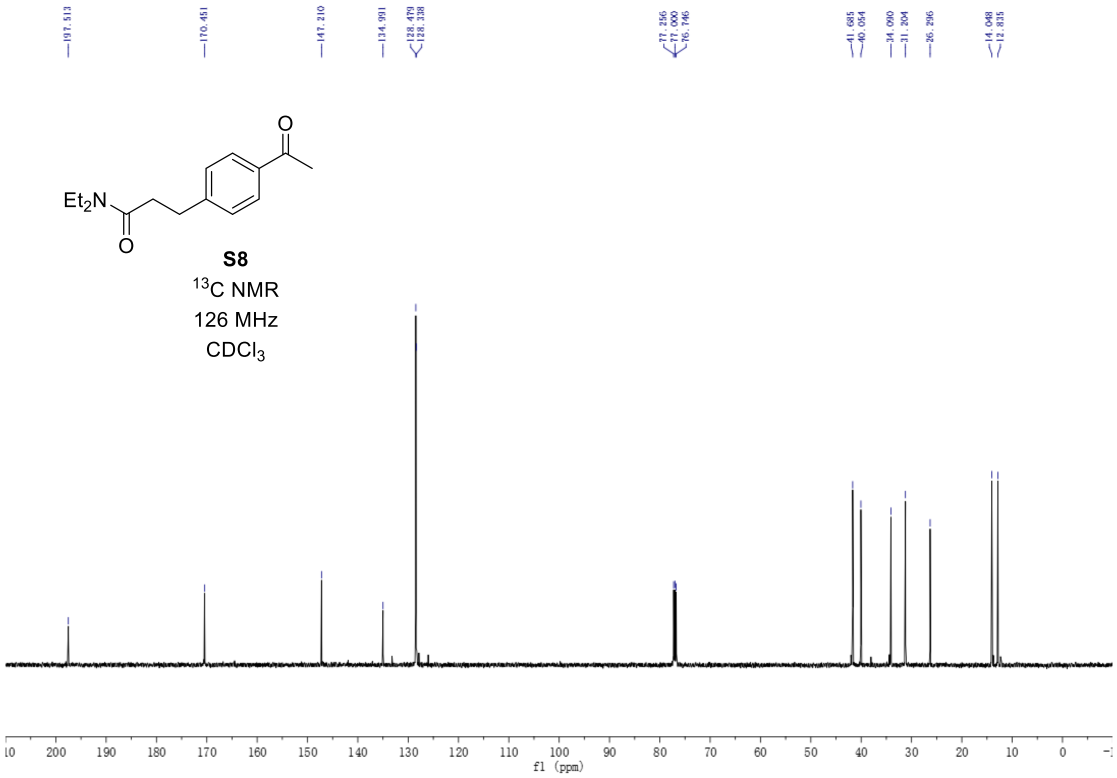

Supplementary Fig. 13 ^{13}C NMR spectrum of compound **S8**

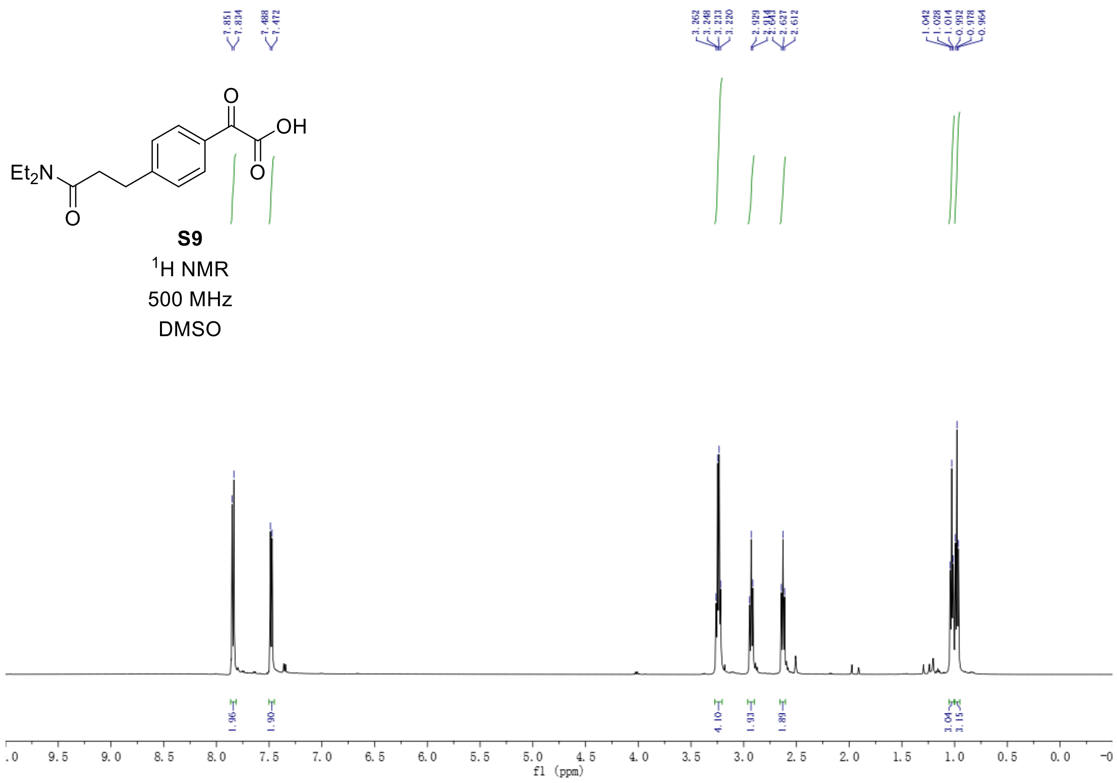

Supplementary Fig. 14 ^{1}H NMR spectrum of compound **S9**

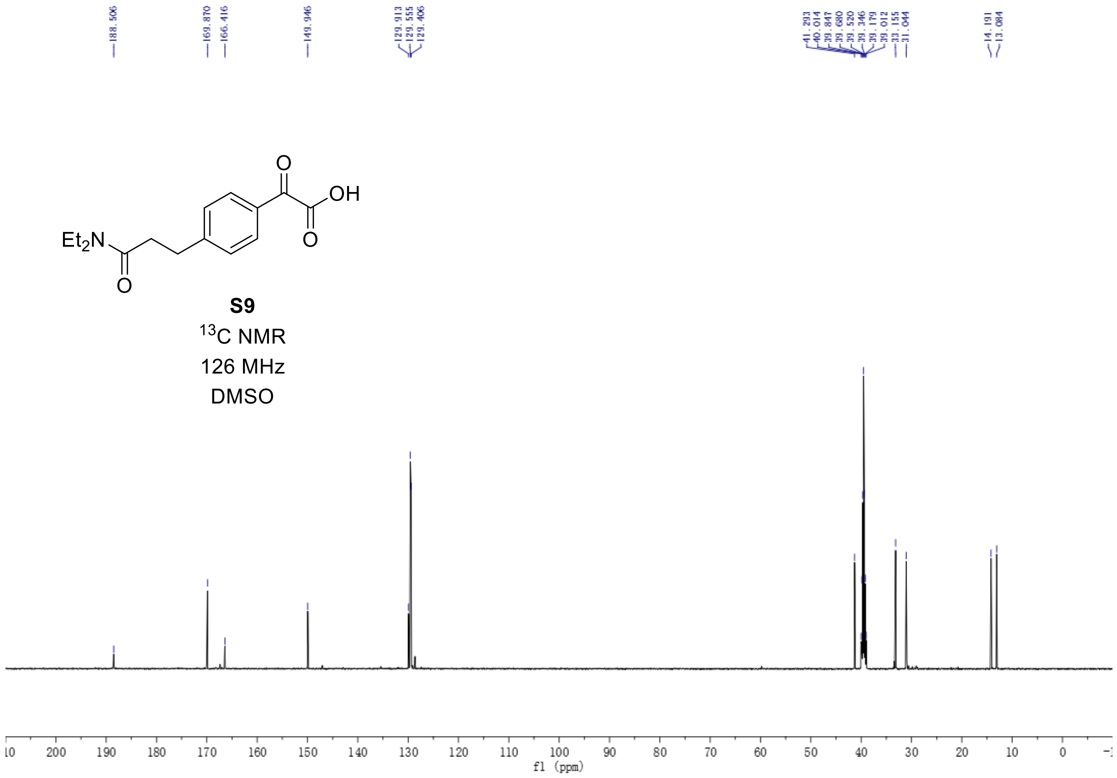

Supplementary Fig. 15 ^{13}C NMR spectrum of compound **S9**

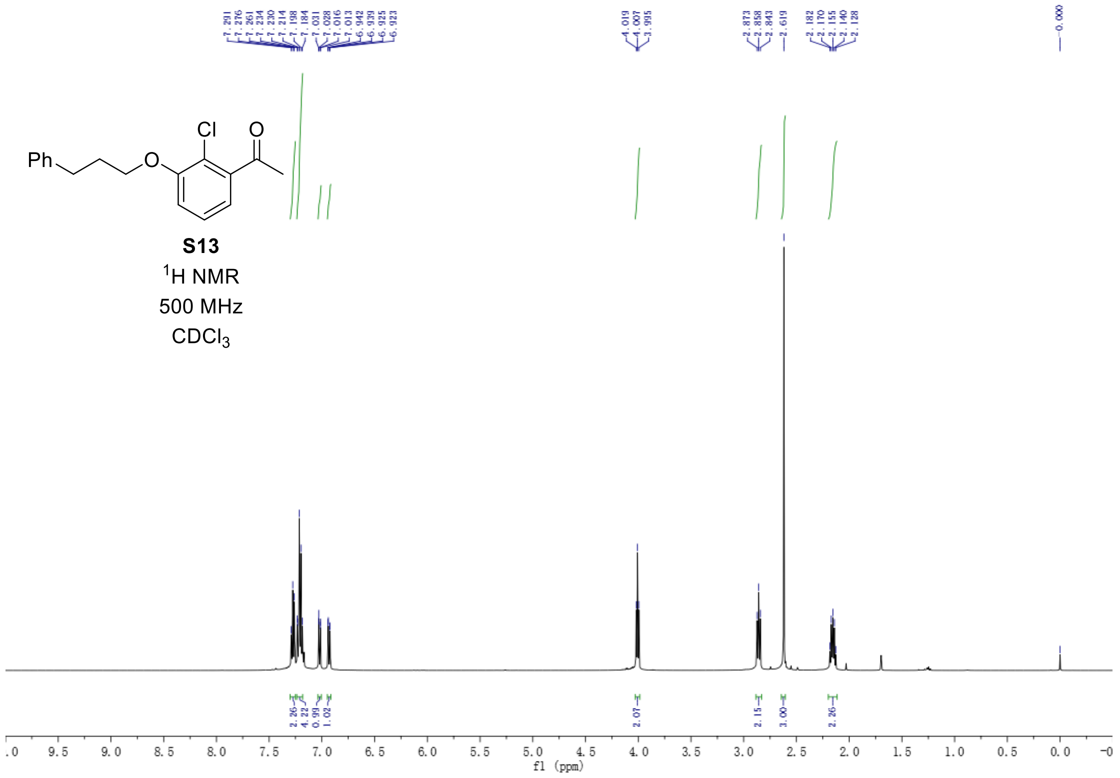

Supplementary Fig. 16 ^{1}H NMR spectrum of compound **S13**

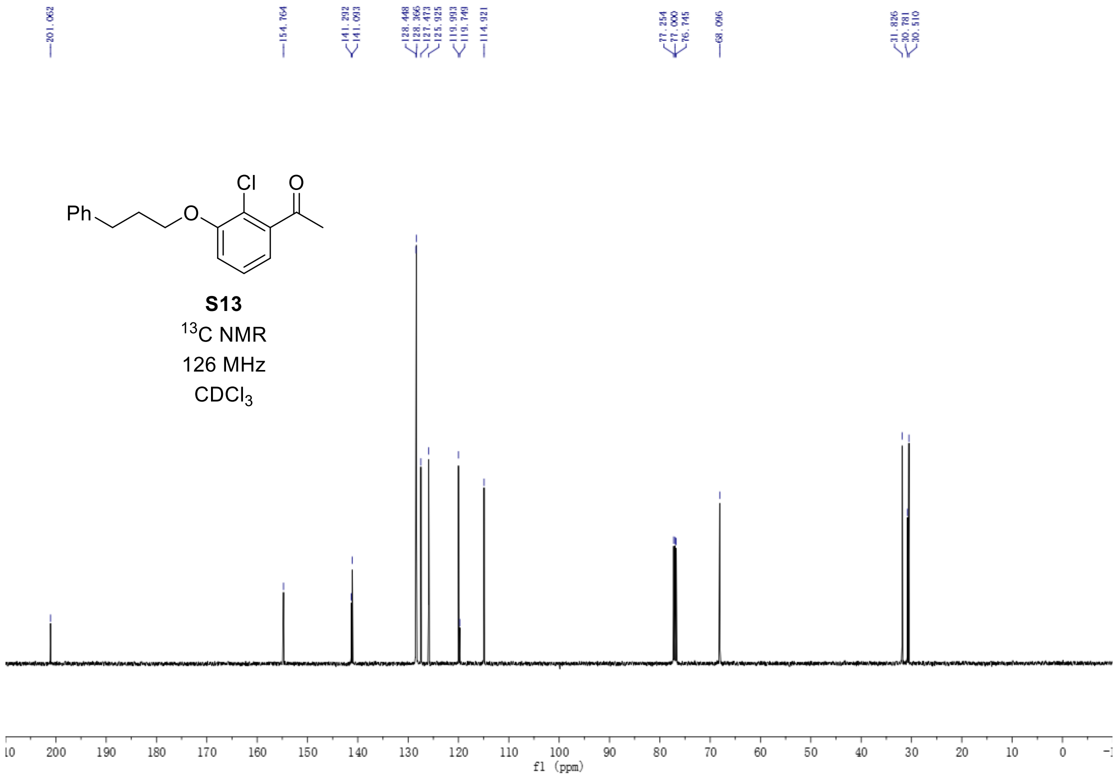

Supplementary Fig. 17 ^{13}C NMR spectrum of compound **S13**

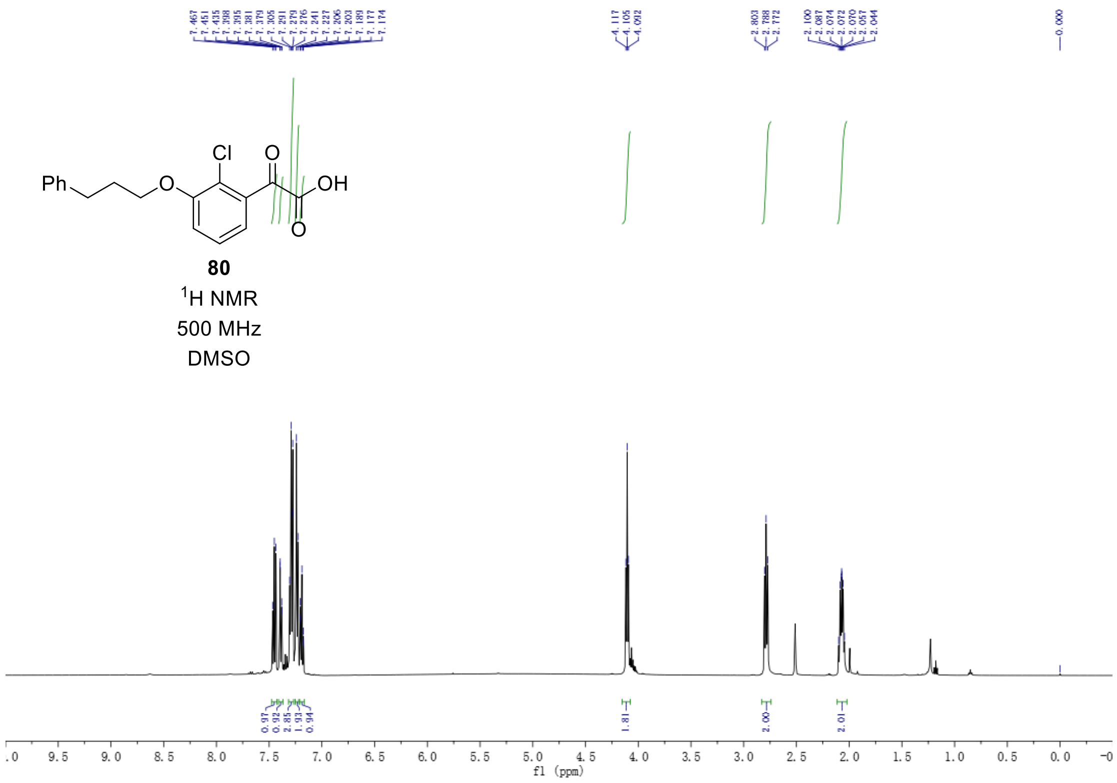

Supplementary Fig. 18 ^{1}H NMR spectrum of compound **80**

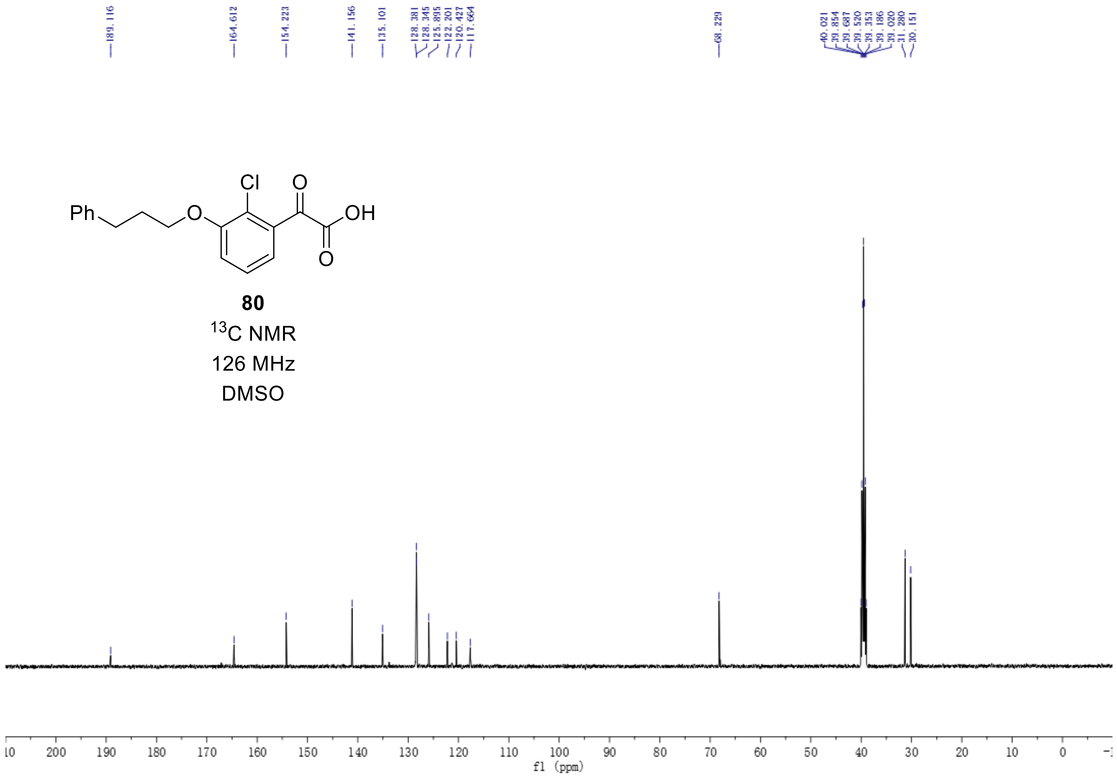

Supplementary Fig. 19 ^{13}C NMR spectrum of compound **80**

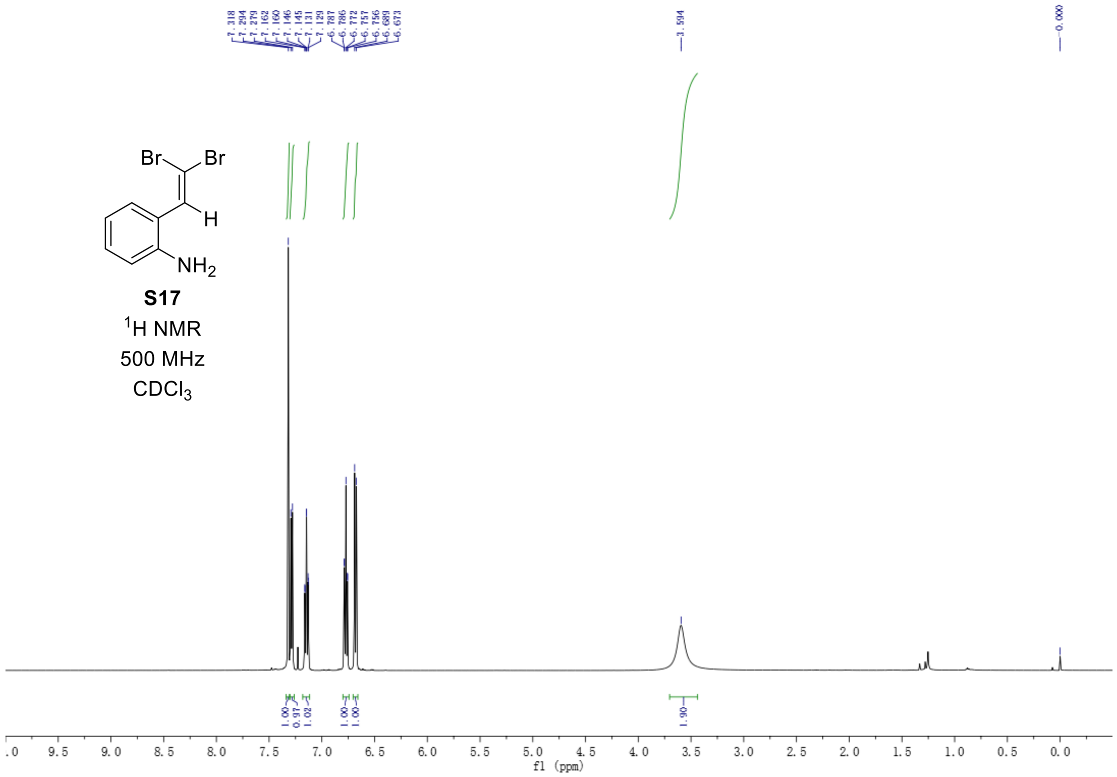

Supplementary Fig. 20 ^{1}H NMR spectrum of compound **S17**

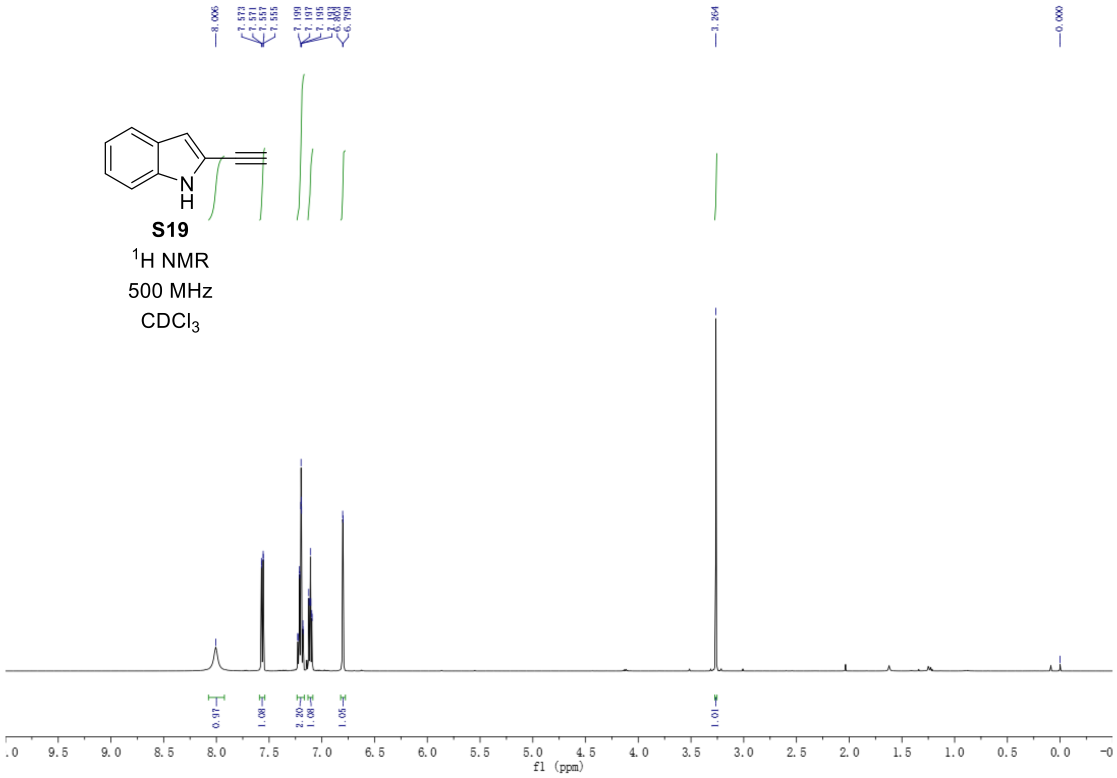

Supplementary Fig. 21 ^{13}C NMR spectrum of compound **S19**

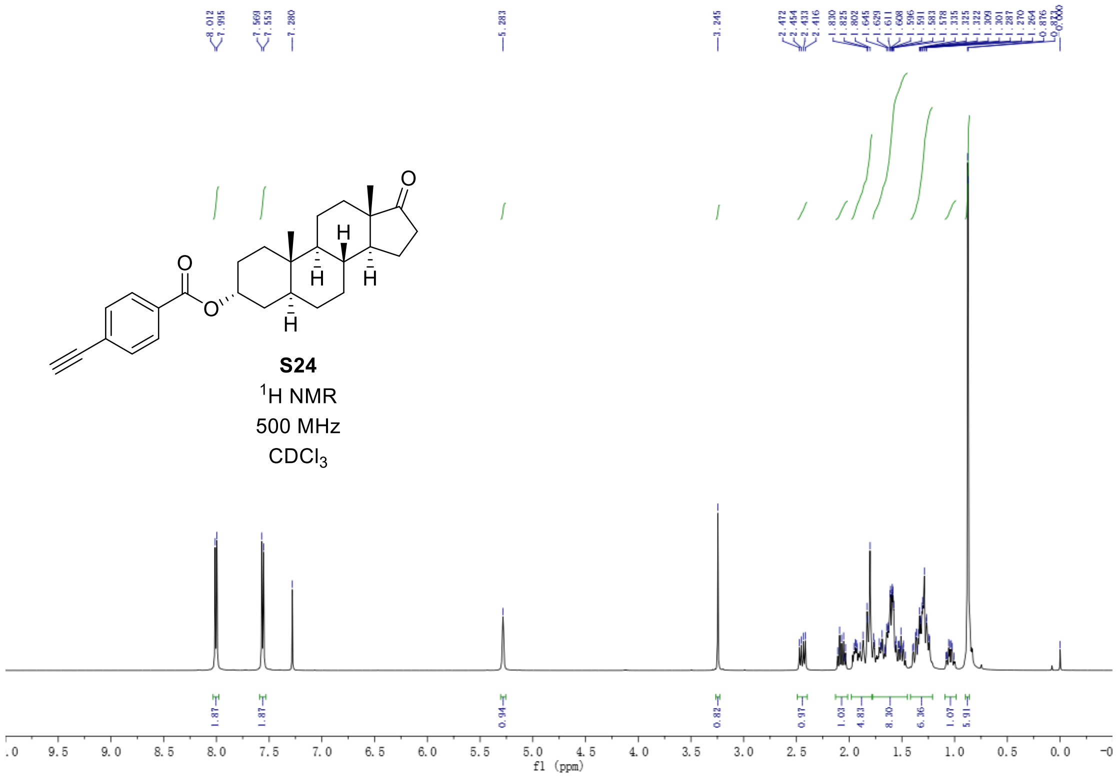

Supplementary Fig. 22 ^{1}H NMR spectrum of compound **S24**

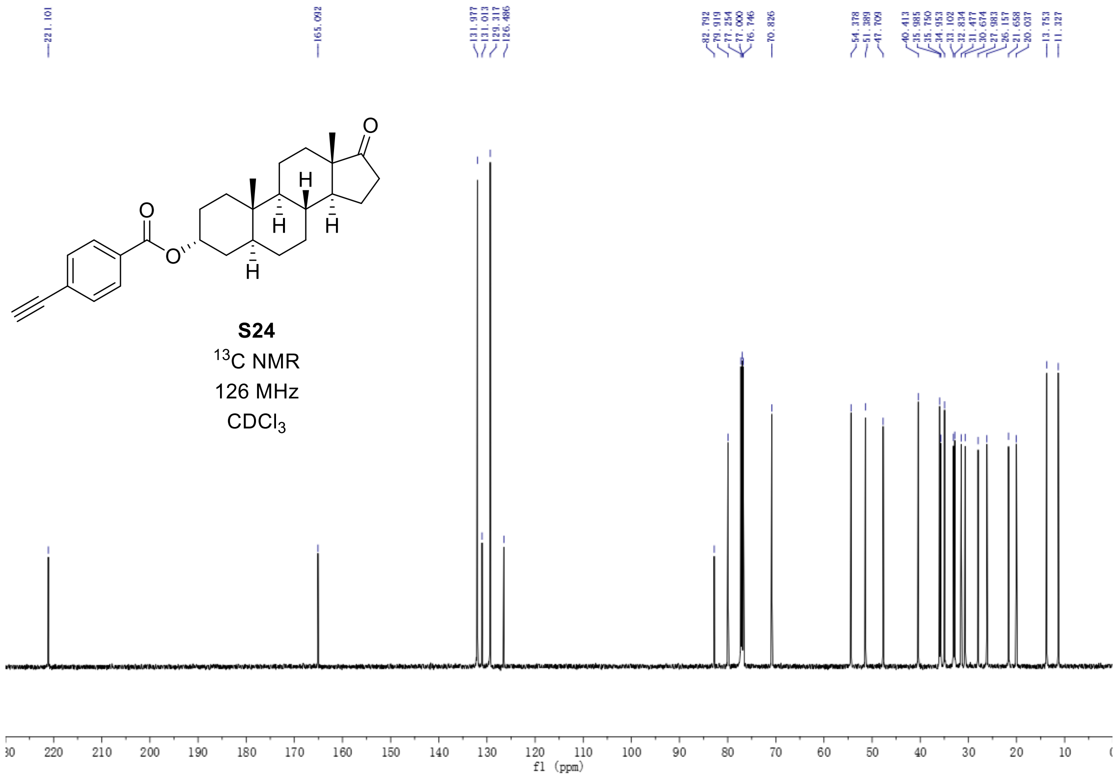

Supplementary Fig. 23 ^{13}C NMR spectrum of compound **S24**

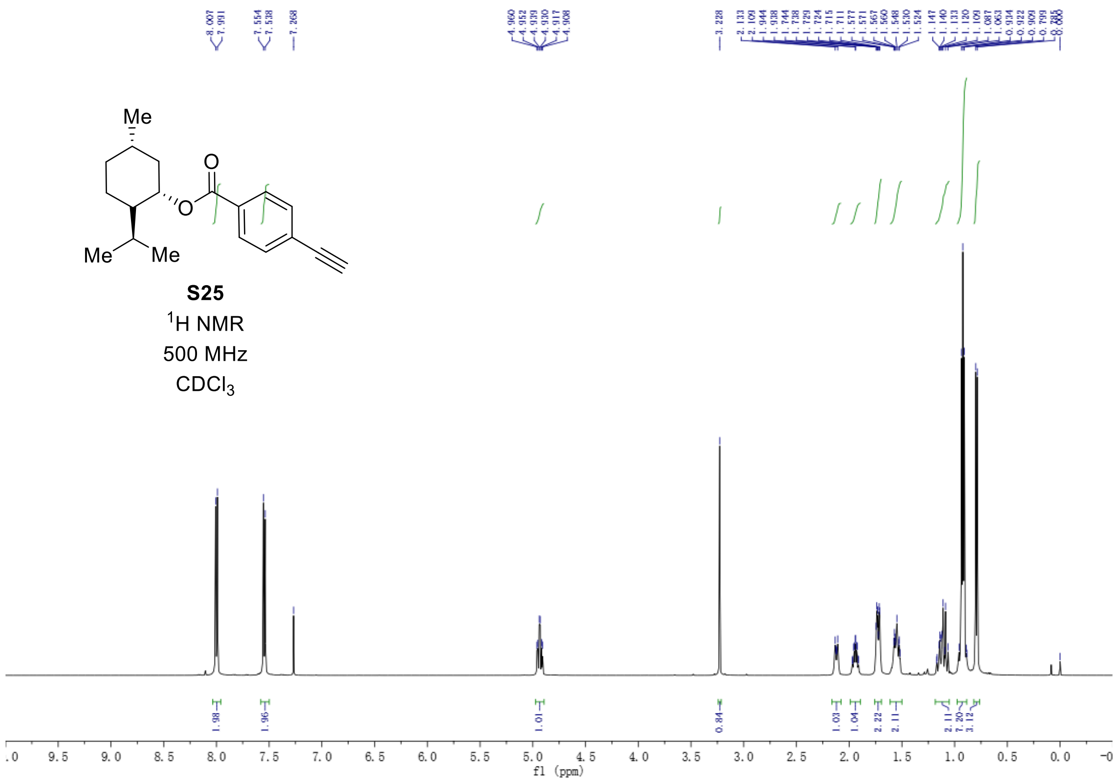

Supplementary Fig. 24 ^{1}H NMR spectrum of compound **S25**

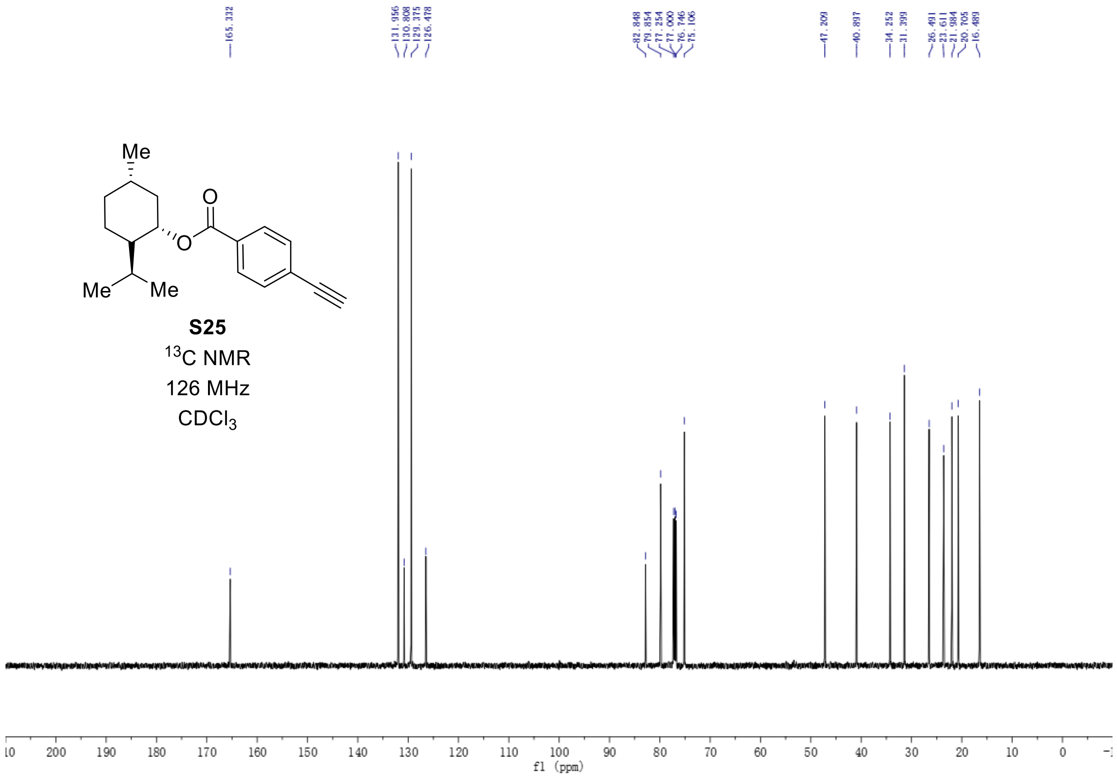

Supplementary Fig. 25 ^{13}C NMR spectrum of compound **S25**

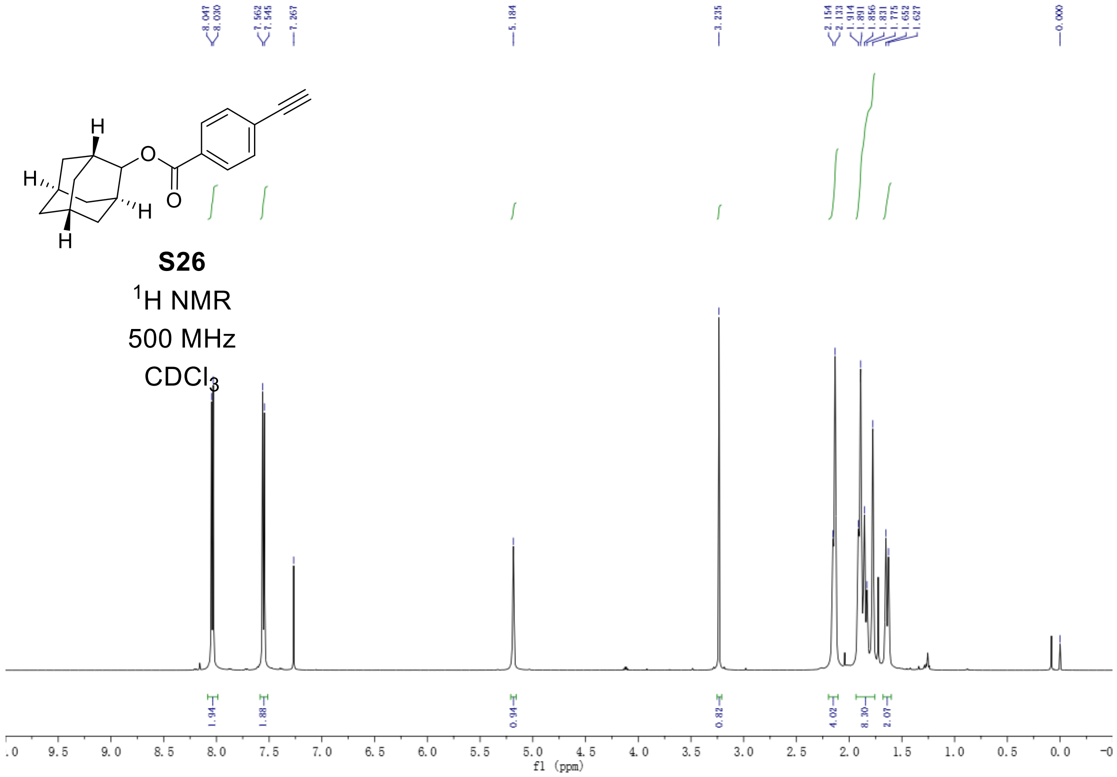

Supplementary Fig. 26 ^{1}H NMR spectrum of compound **S26**

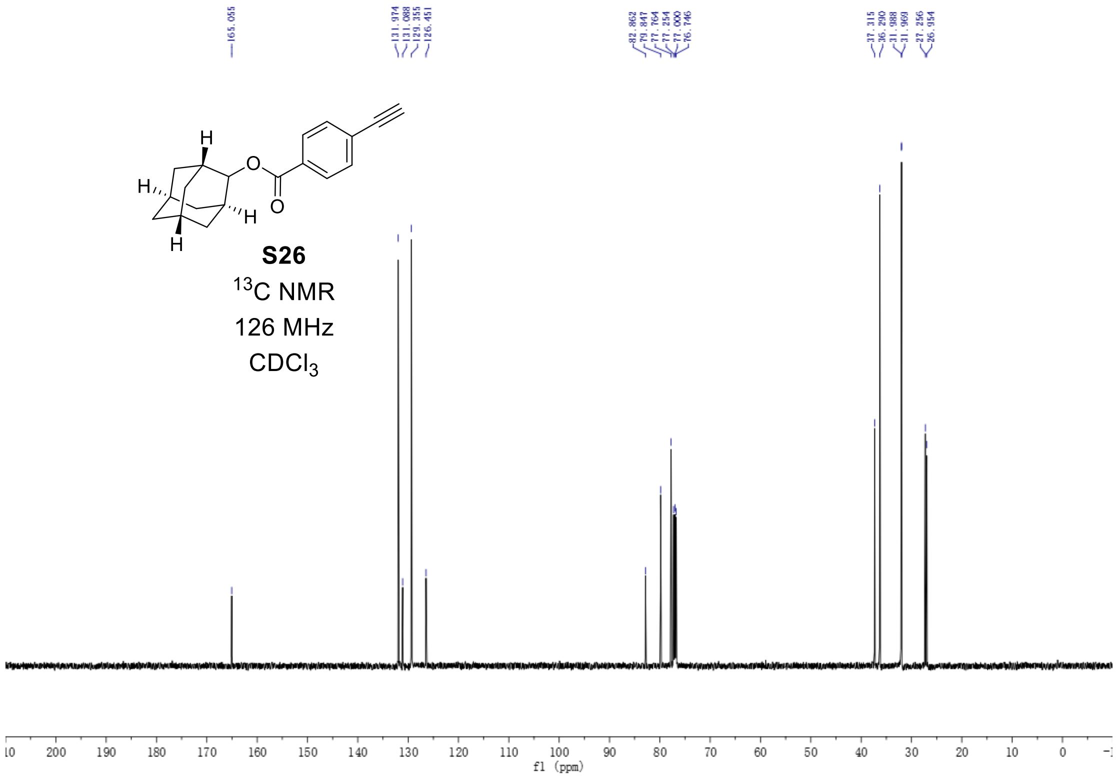

Supplementary Fig. 27 ^{13}C NMR spectrum of compound **S26**

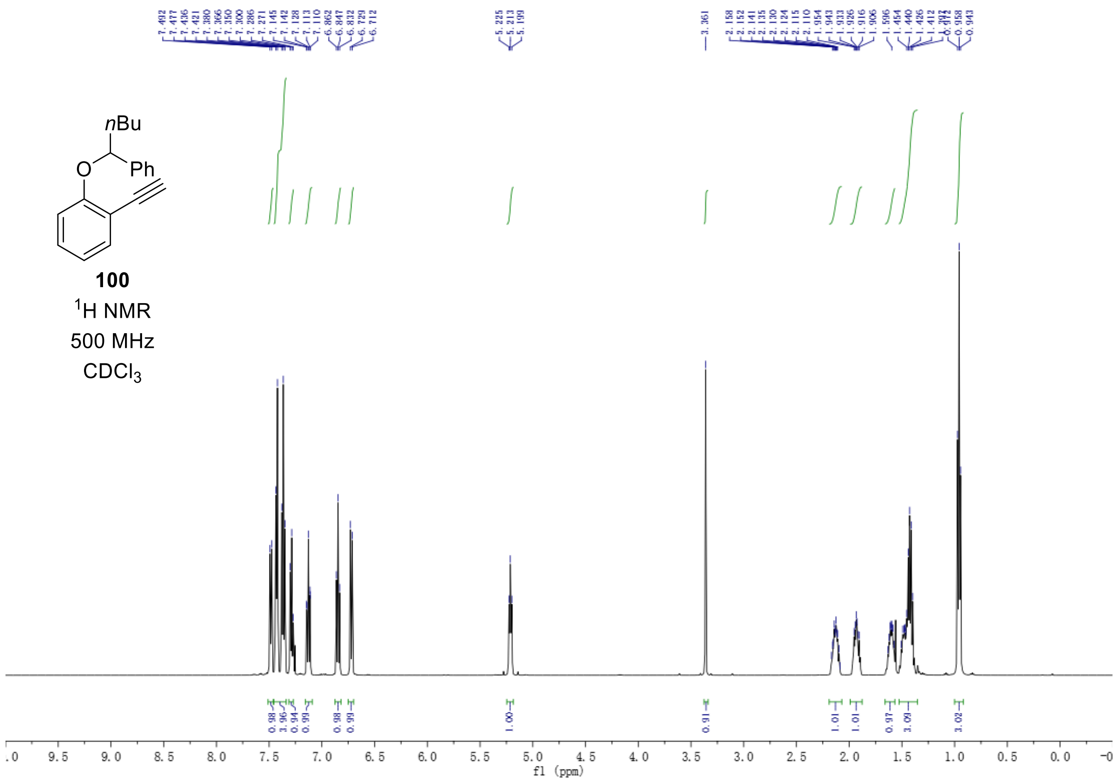

Supplementary Fig. 28 ^{1}H NMR spectrum of compound **100**

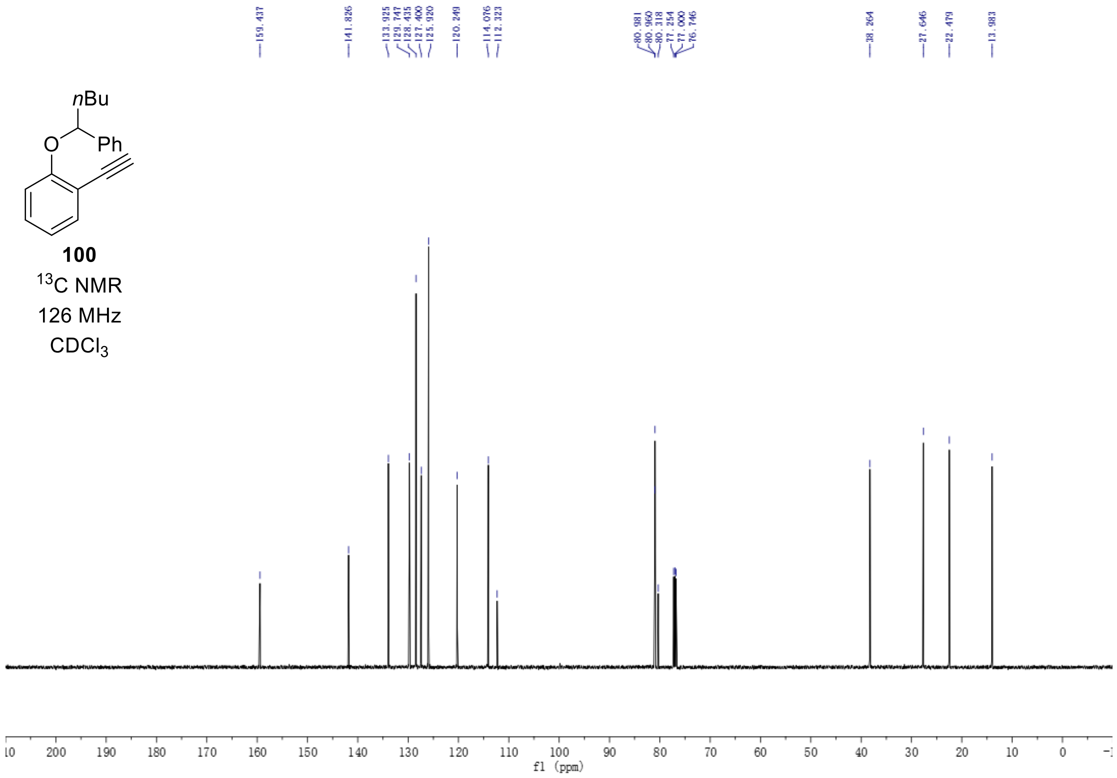

Supplementary Fig. 29 ^{13}C NMR spectrum of compound **100**

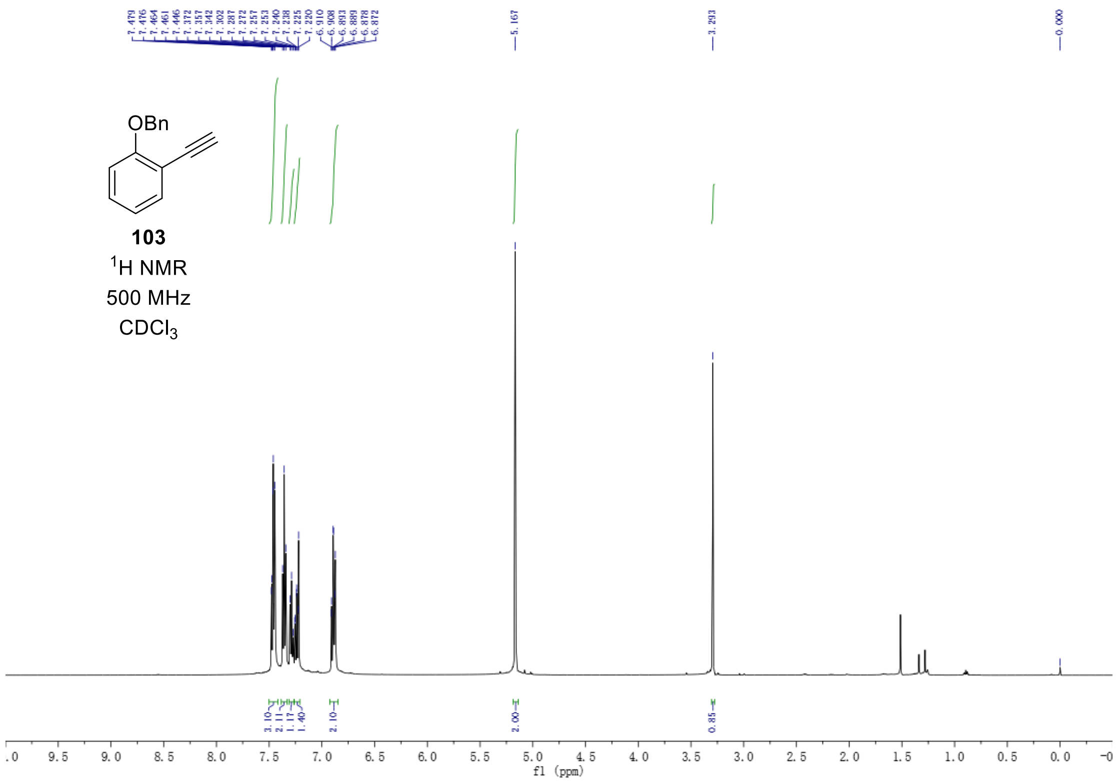

Supplementary Fig. 30 ^{1}H NMR spectrum of compound **103**

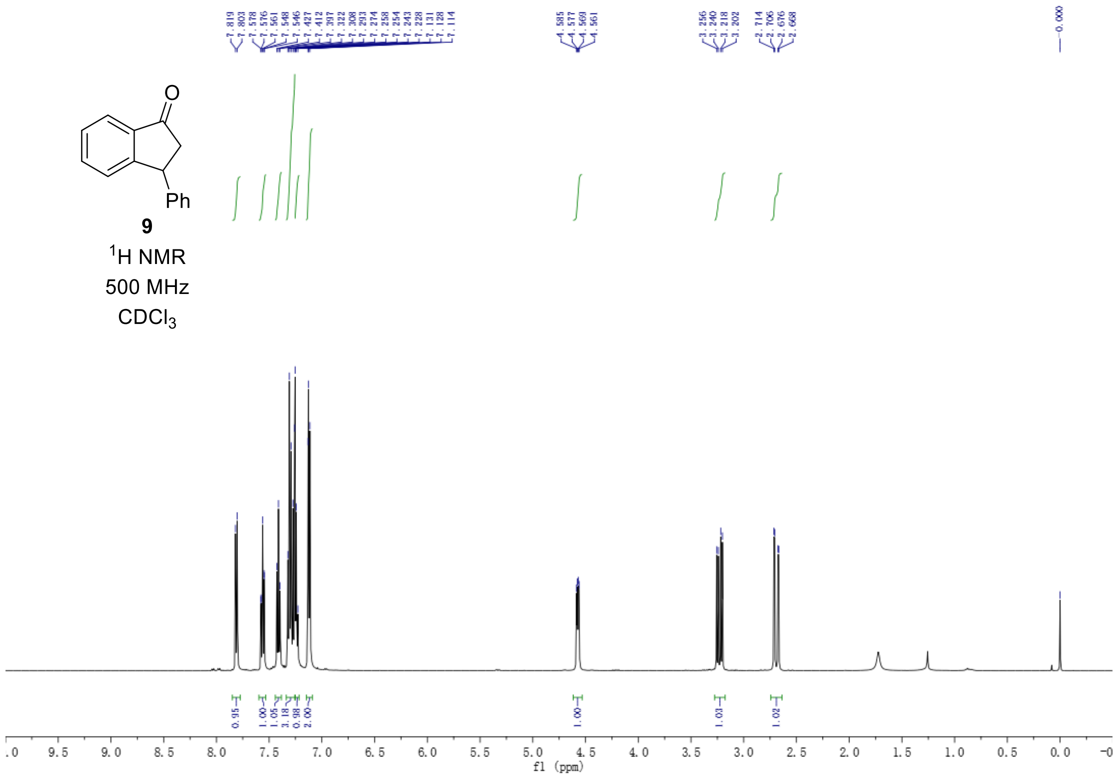

Supplementary Fig. 31 ^{1}H NMR spectrum of compound **9**

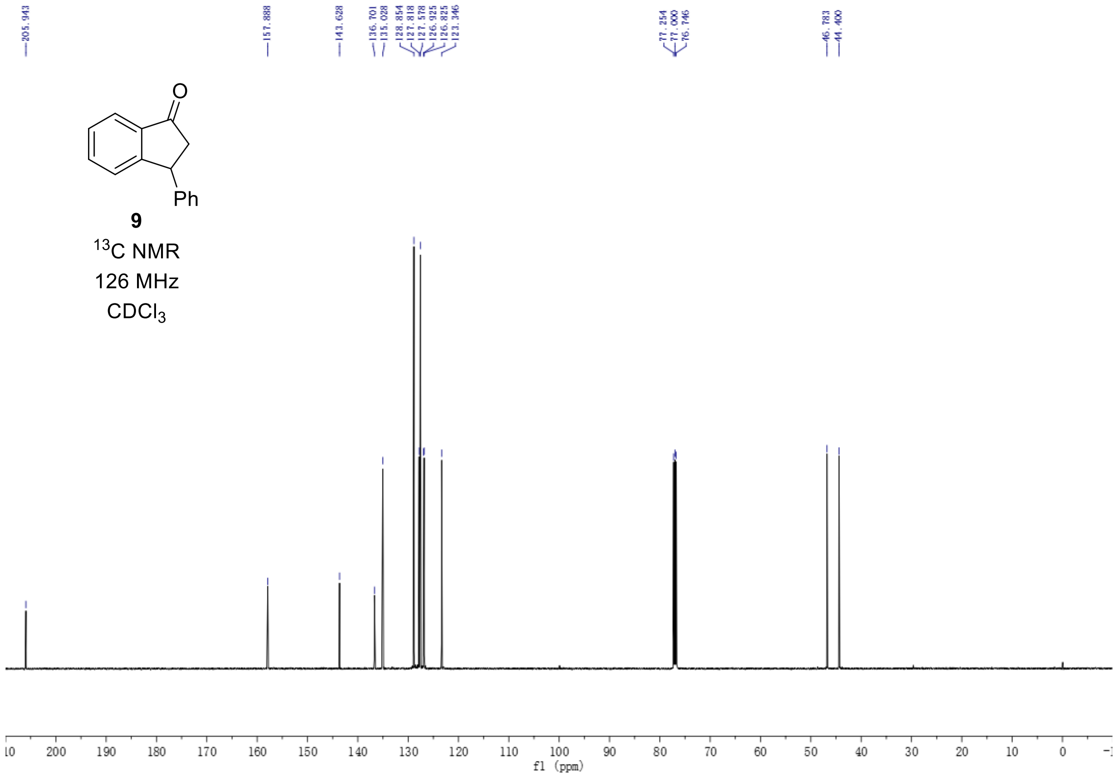

Supplementary Fig. 32 ^{13}C NMR spectrum of compound **9**

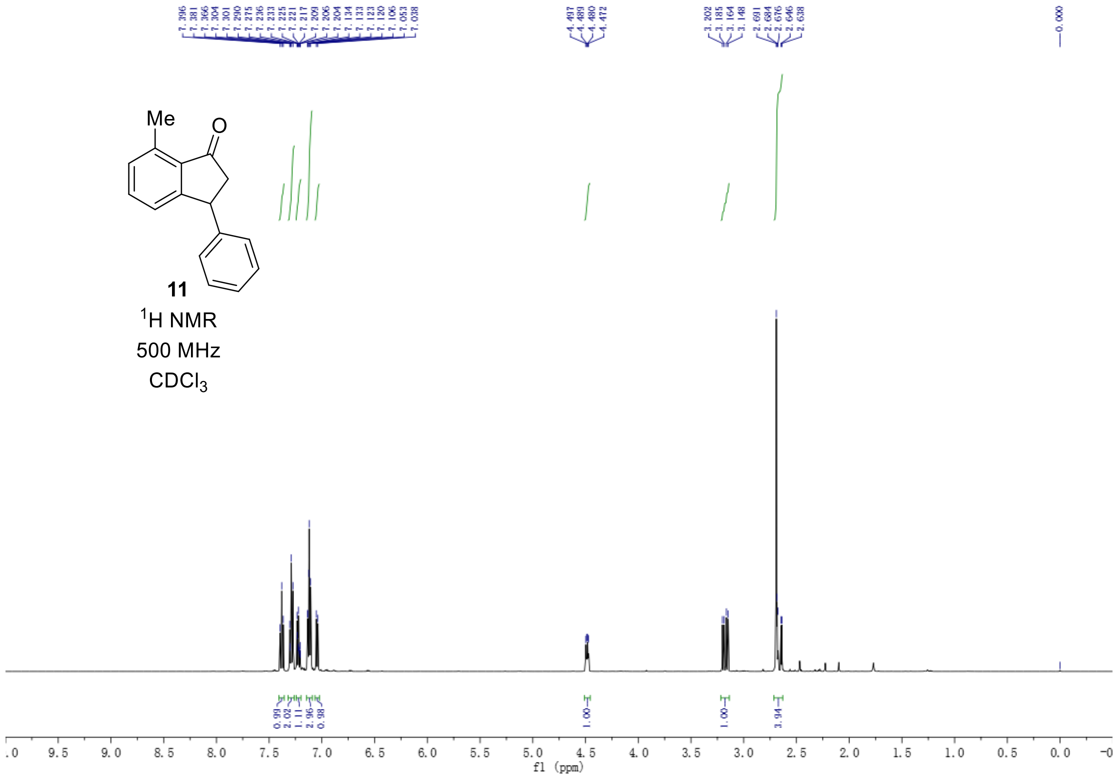

Supplementary Fig. 33 ^{1}H NMR spectrum of compound **11**

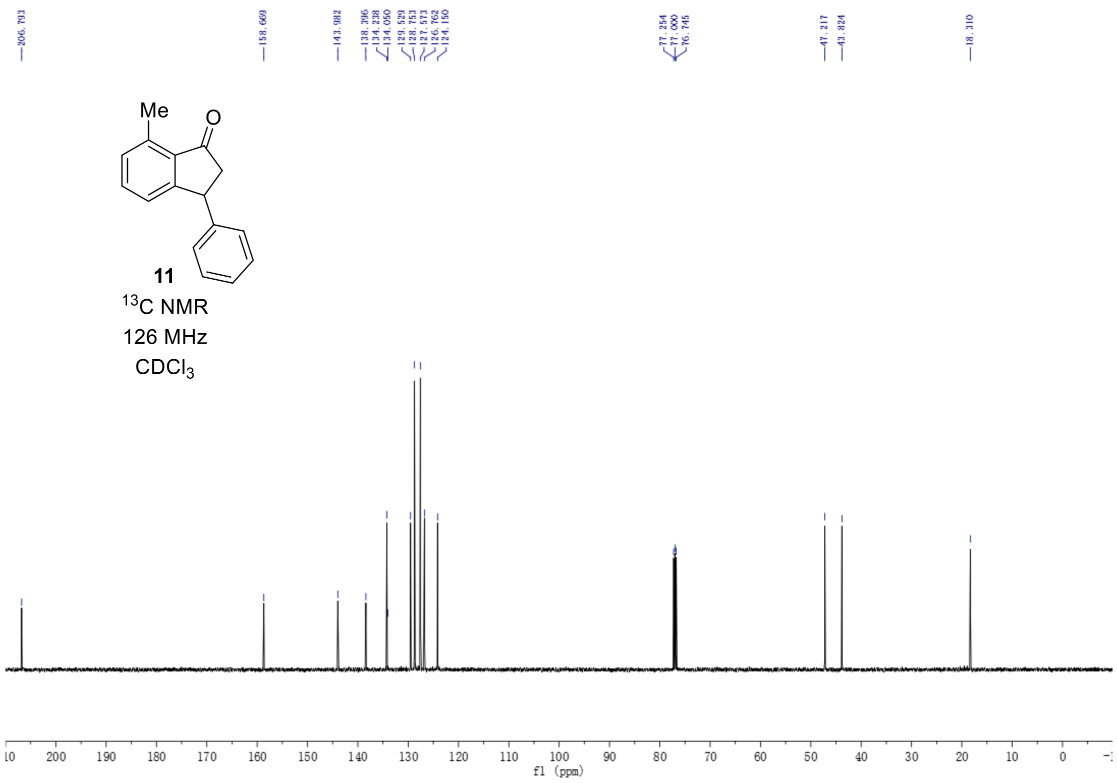

Supplementary Fig. 34 ^{13}C NMR spectrum of compound **11**

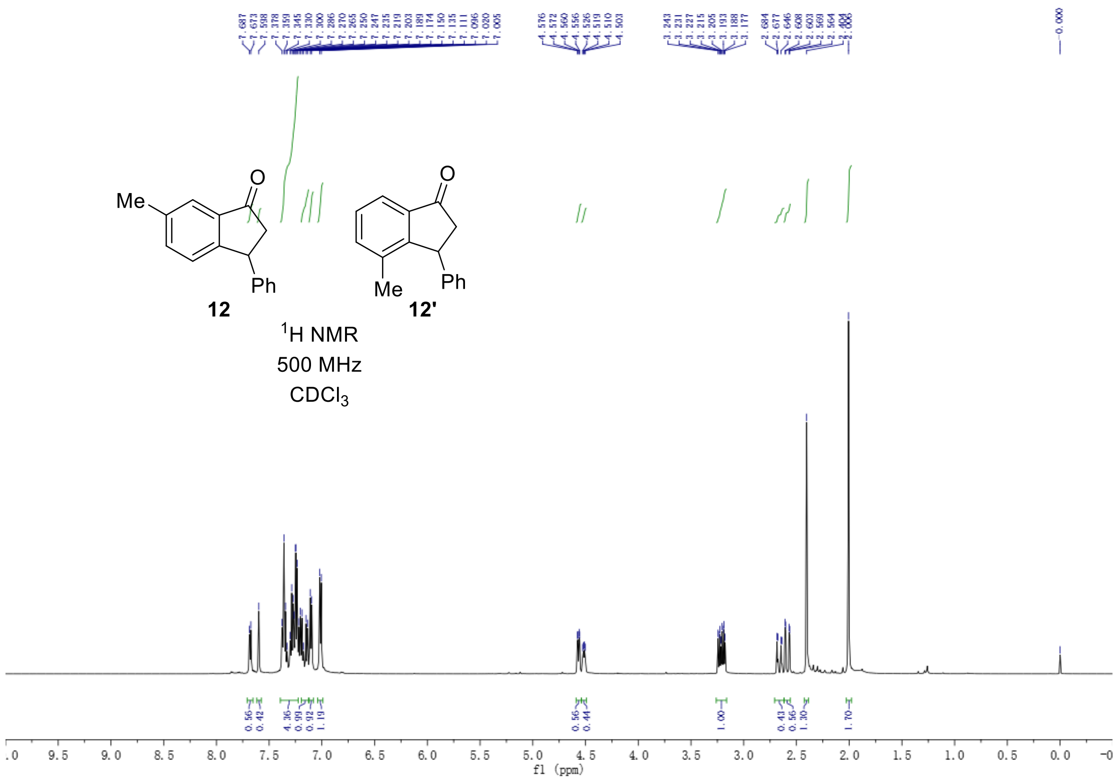

Supplementary Fig. 35 ^{1}H NMR spectrum of a mixture of **12** and **12’**

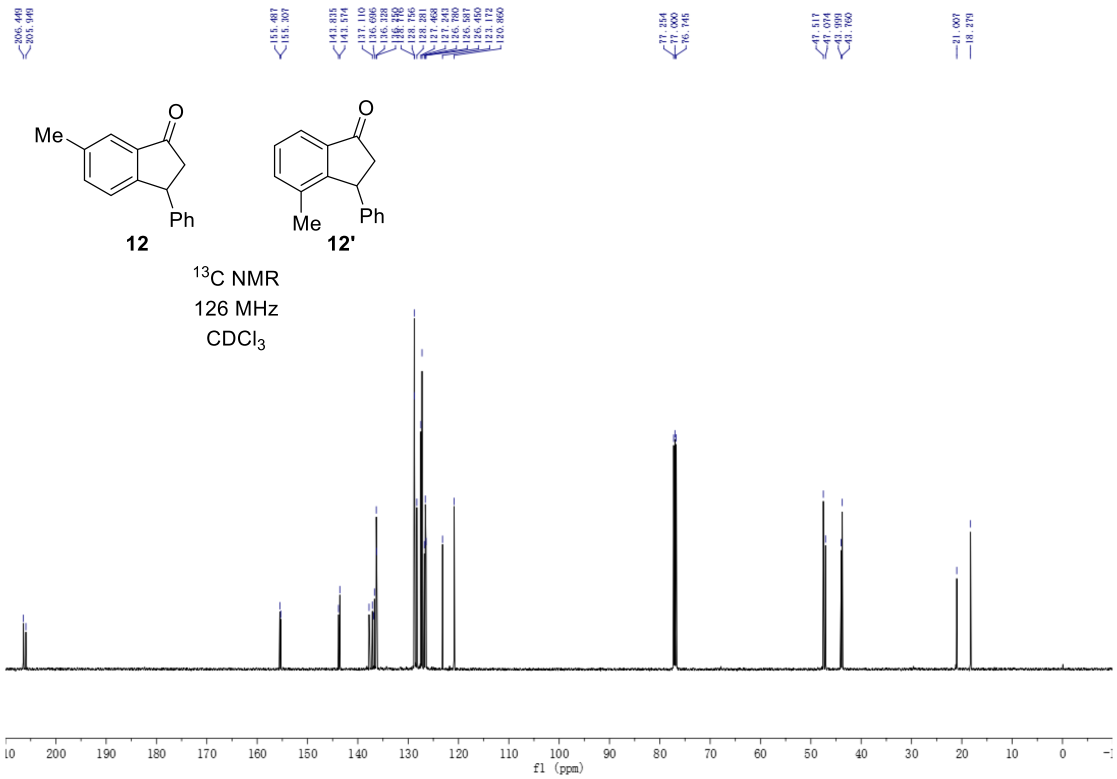

Supplementary Fig. 36 ^{13}C NMR spectrum of compound **12b** and **12'**

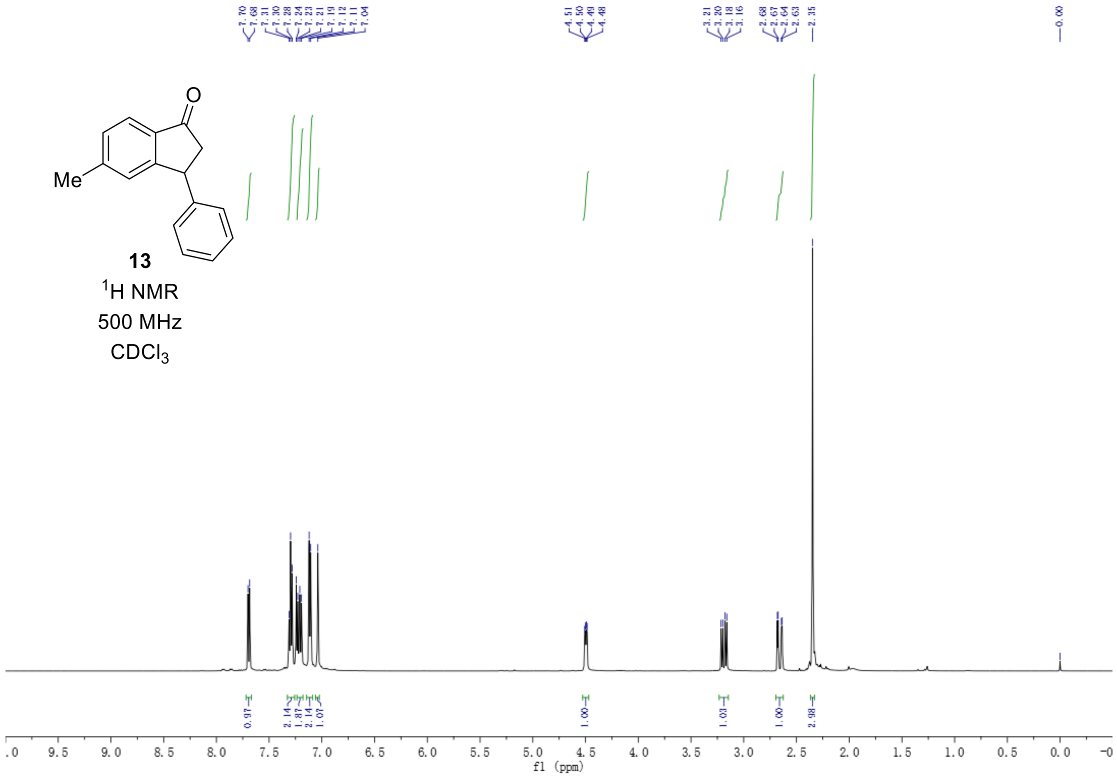

Supplementary Fig. 37 ^{1}H NMR spectrum of compound **13**

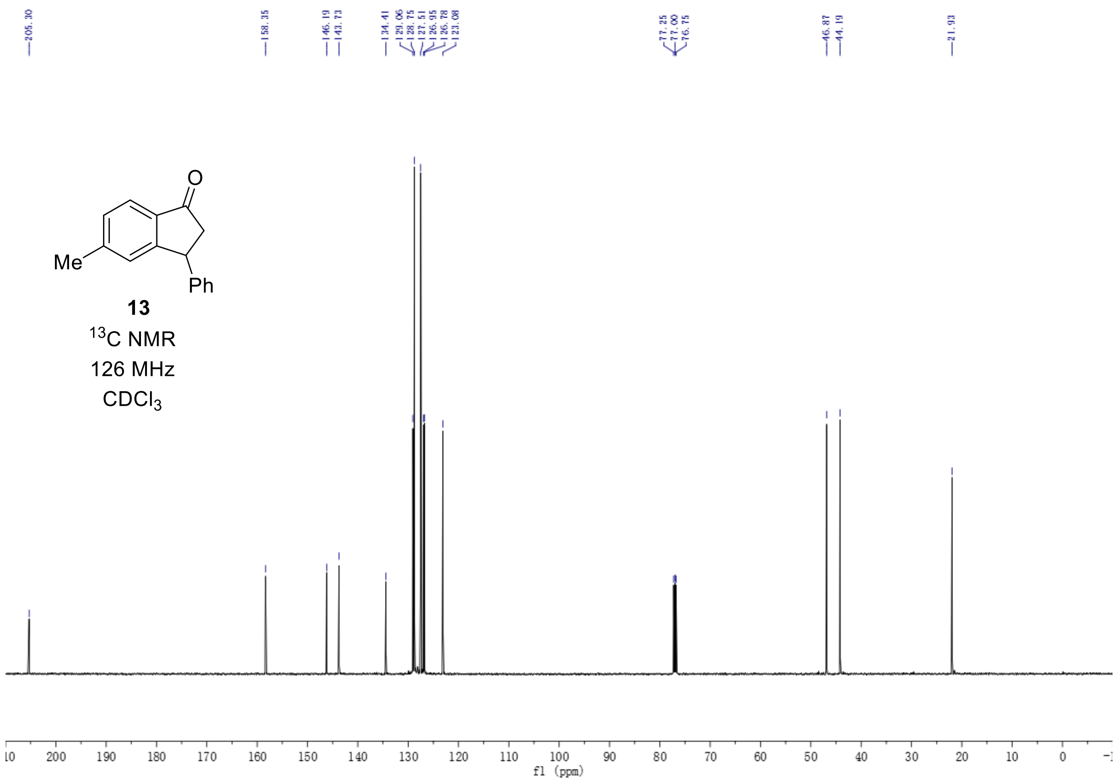

Supplementary Fig. 38 ^{13}C NMR spectrum of compound **13**

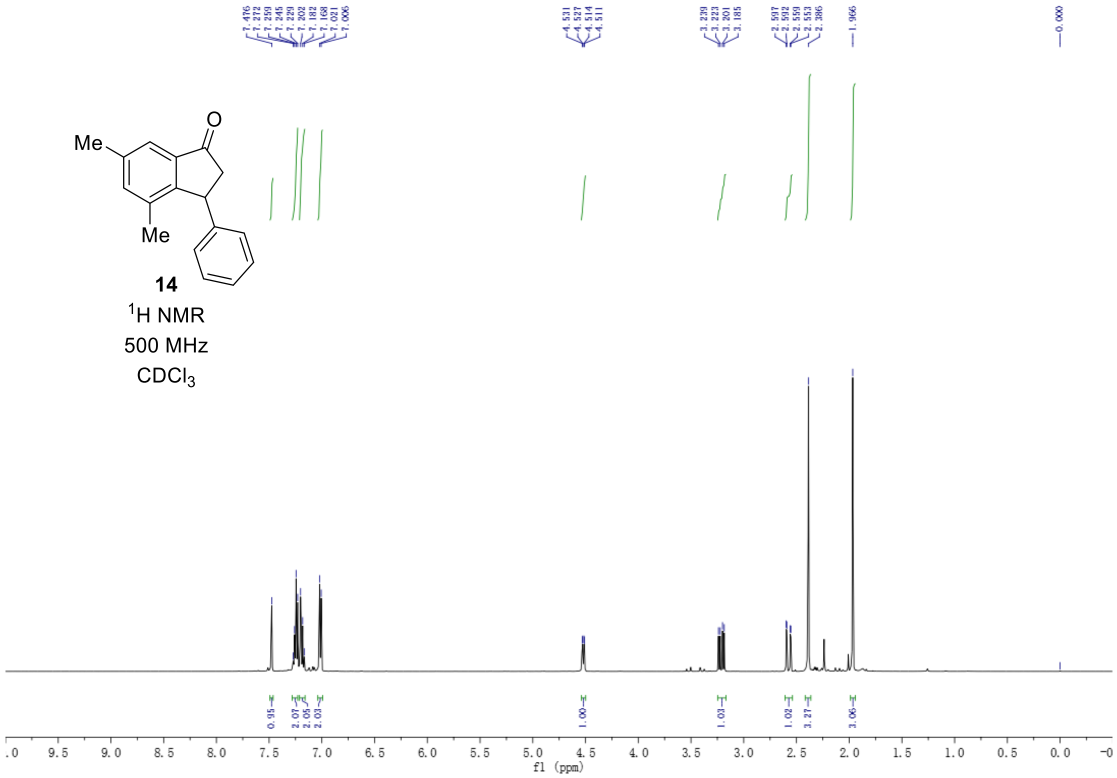

Supplementary Fig. 39 ^{1}H NMR spectrum of compound **14**

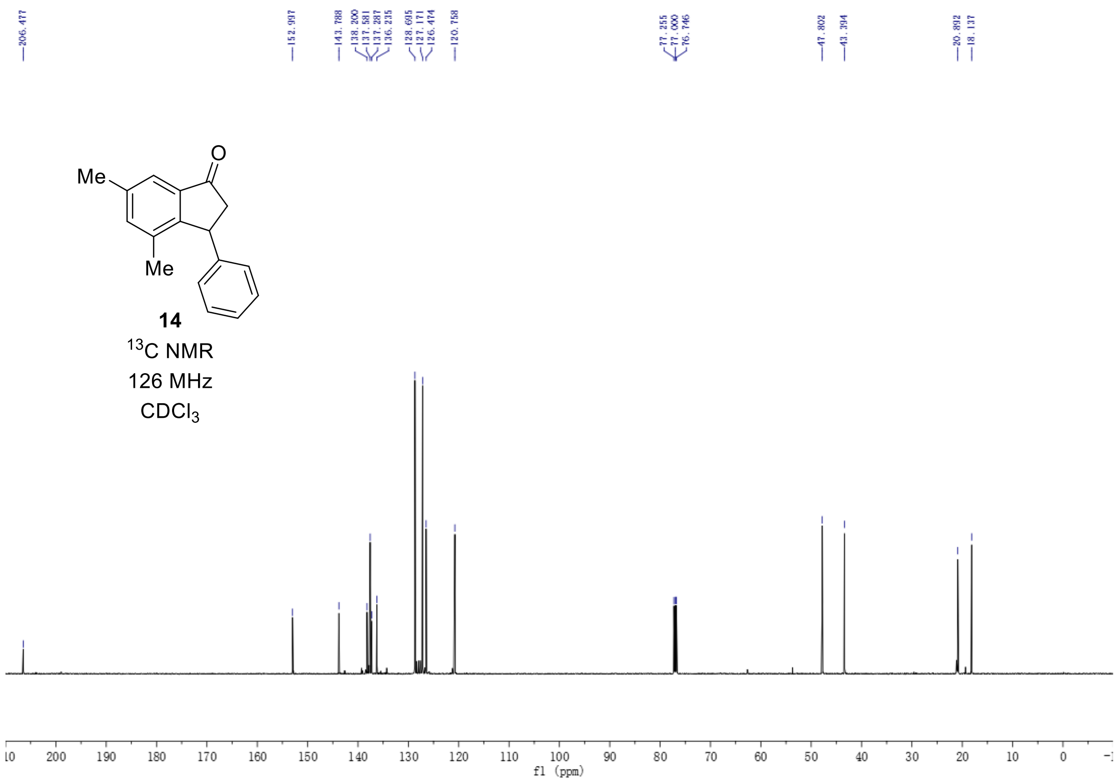

Supplementary Fig. 40 ^{13}C NMR spectrum of compound **14**

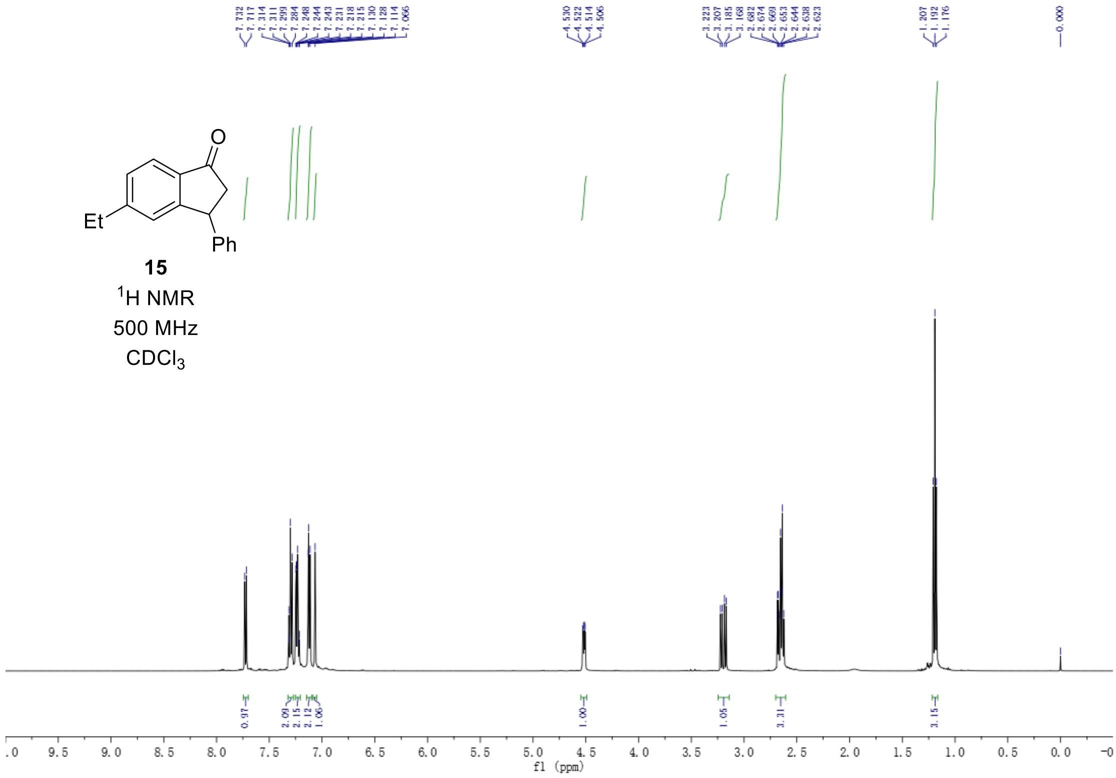

Supplementary Fig. 41$^{13=}$H NMR spectrum of compound **15**

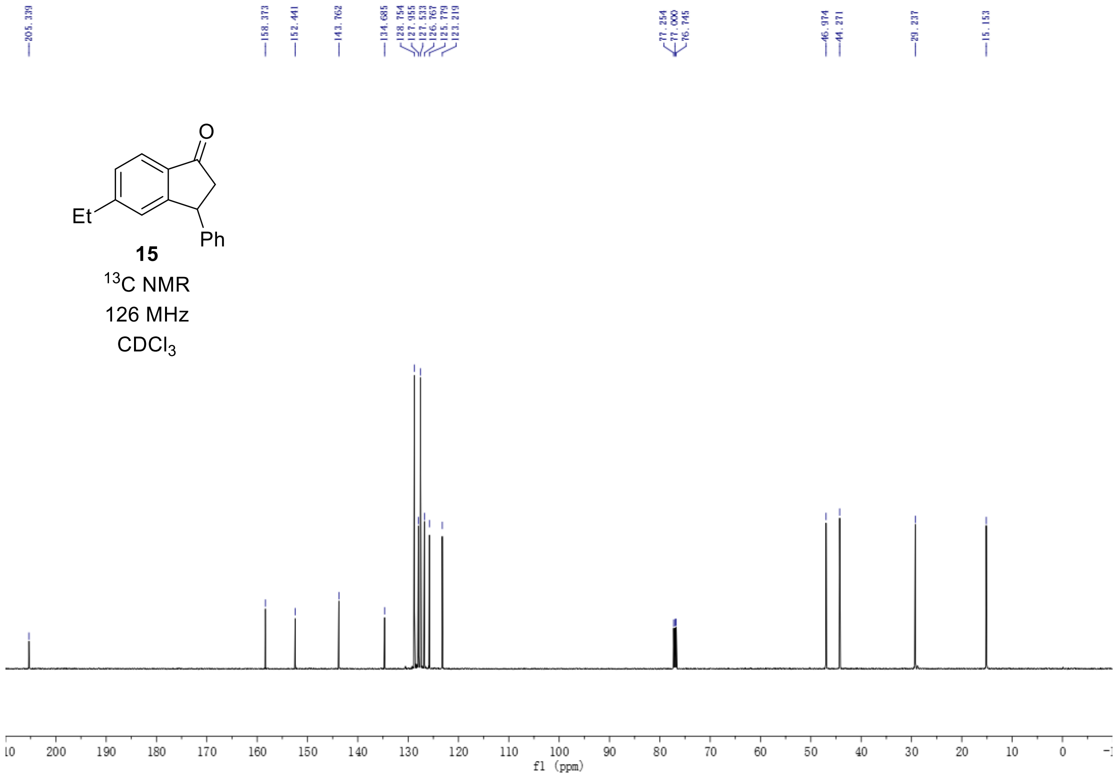

Supplementary Fig. 42 ^{13}C NMR spectrum of compound **15**

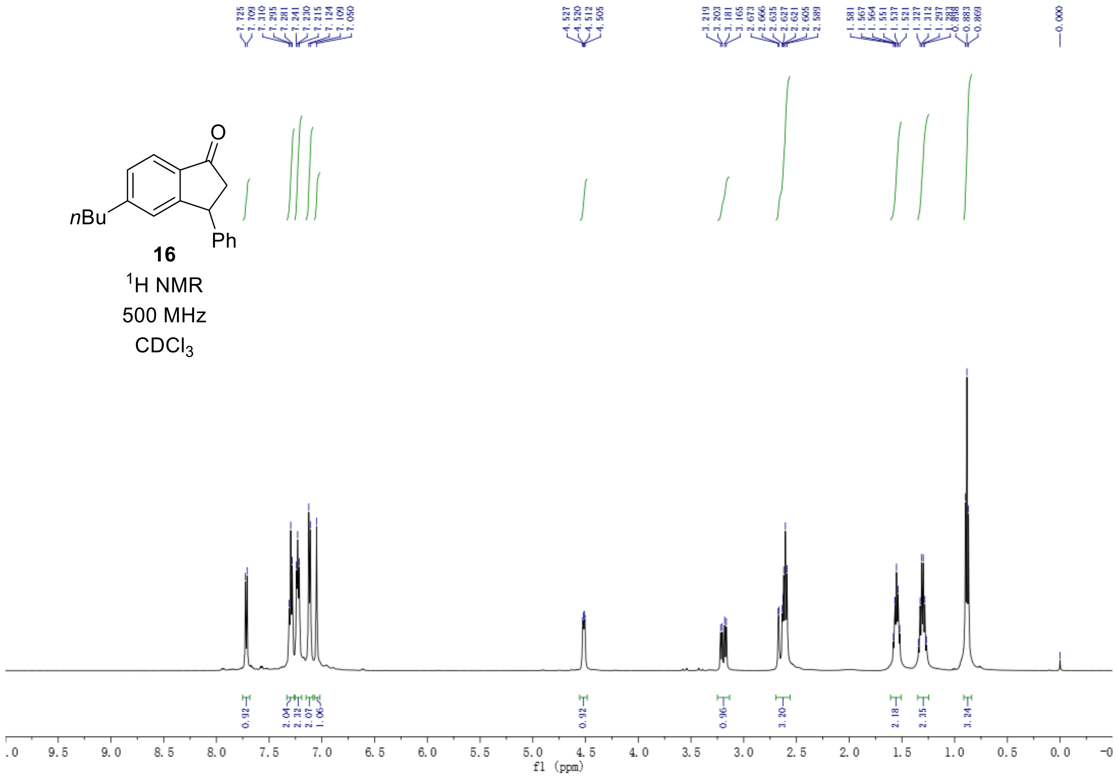

Supplementary Fig. 43 ^{1}H NMR spectrum of compound **16**

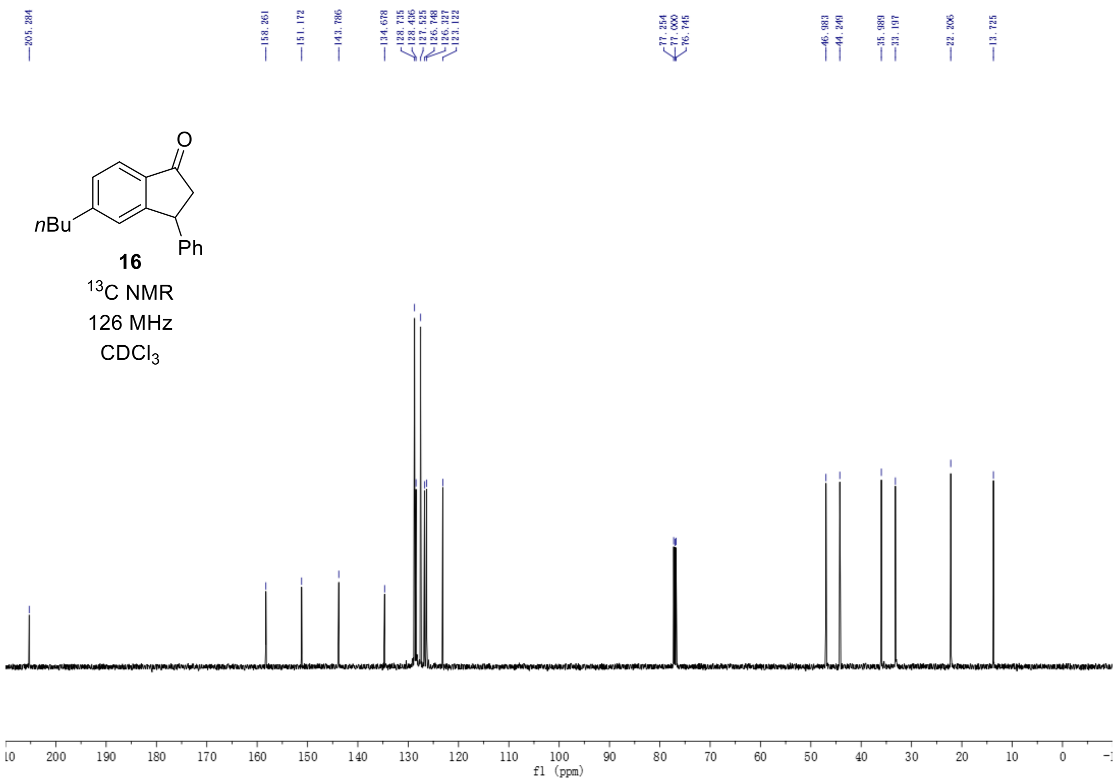

Supplementary Fig. 44 ^{13}C NMR spectrum of compound **16**

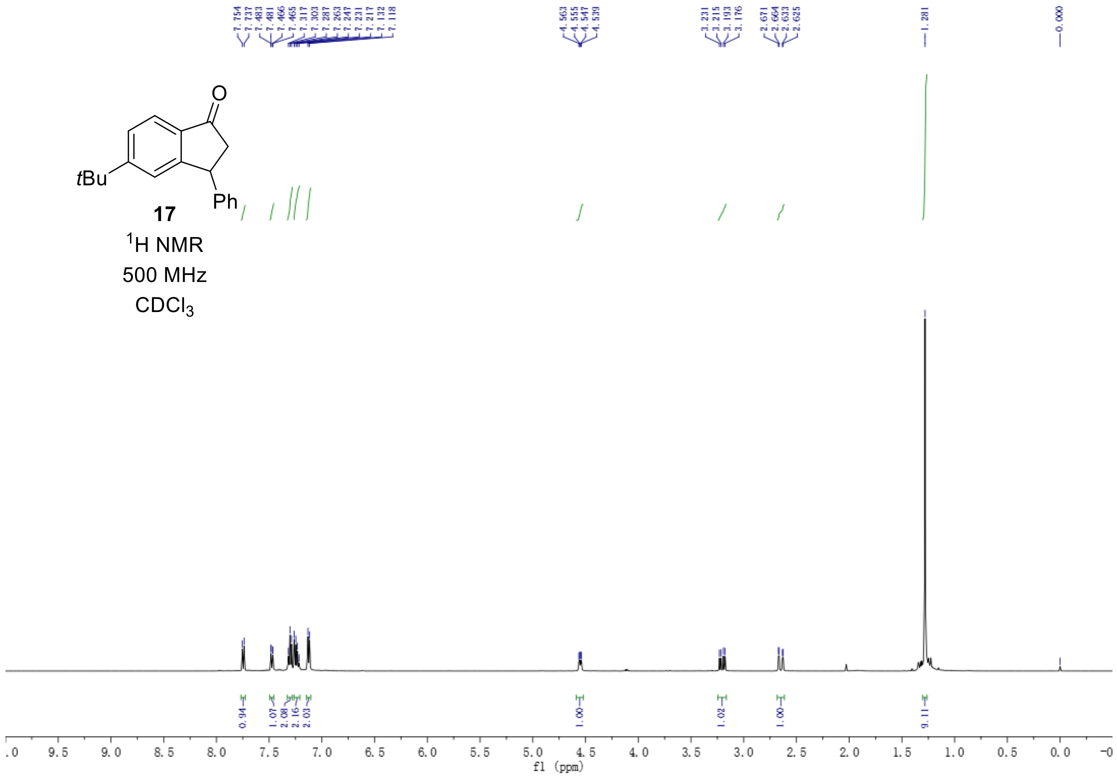

Supplementary Fig. 45 ^{1}H NMR spectrum of compound **17**

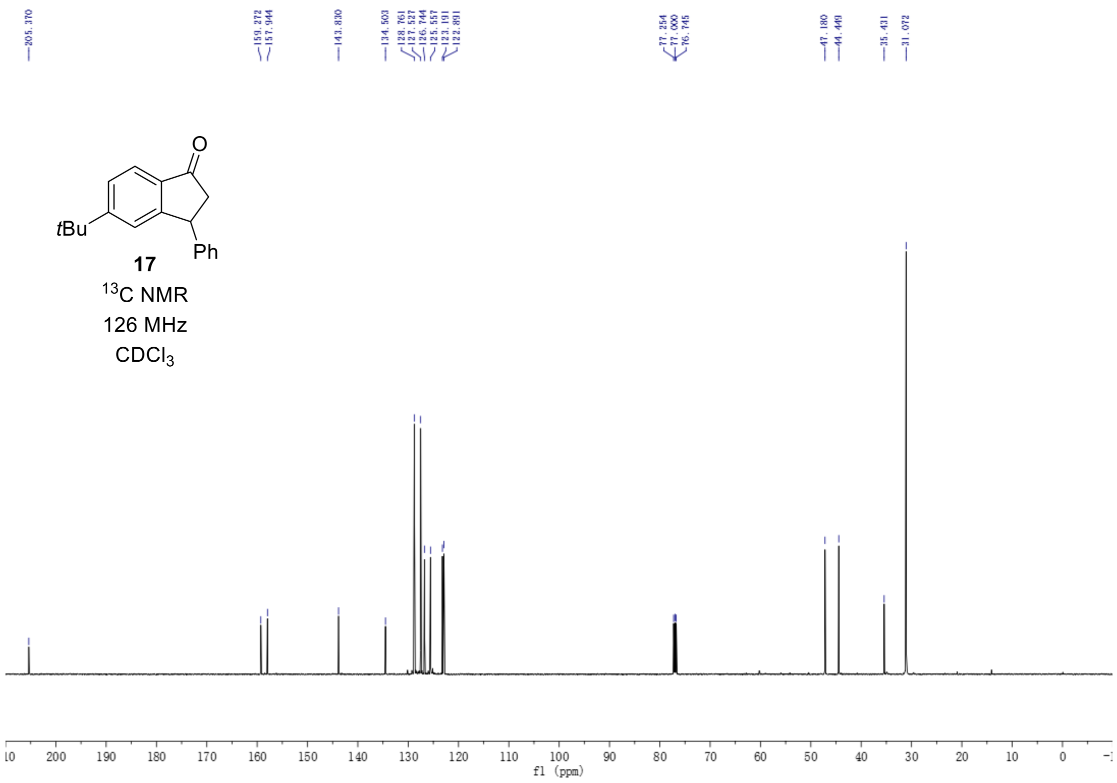

Supplementary Fig. 46 ^{13}C NMR spectrum of compound **17**

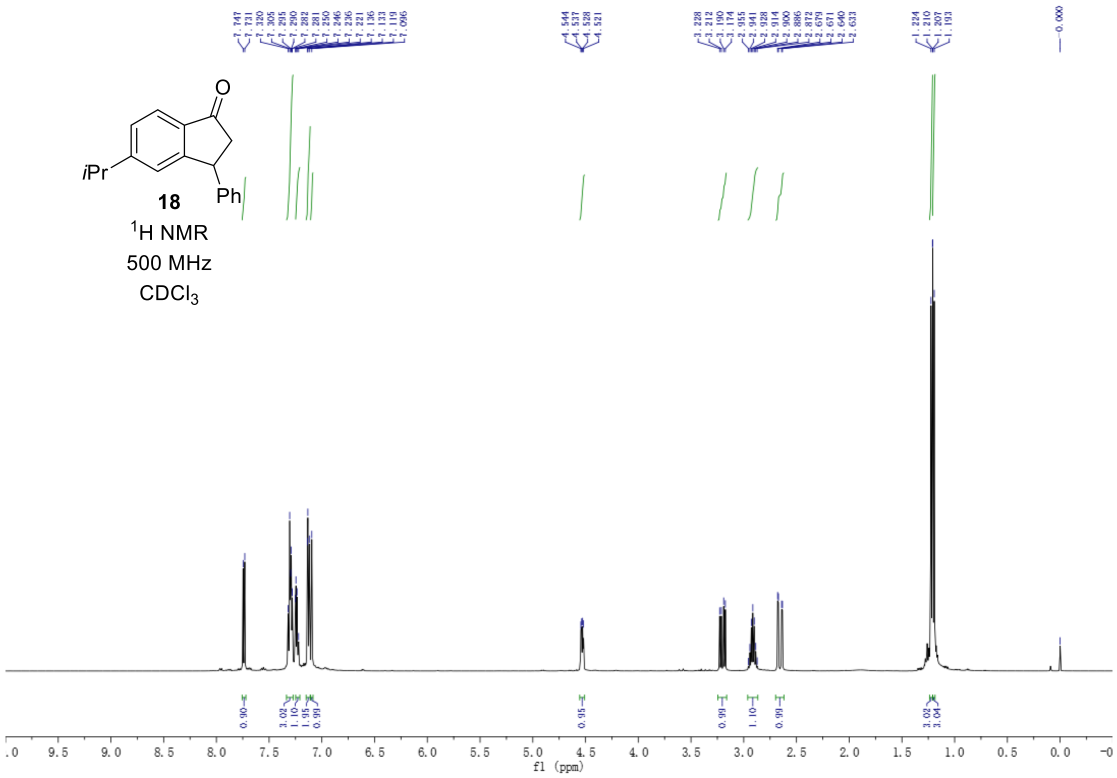

Supplementary Fig. 47 ^{1}H NMR spectrum of compound **18**

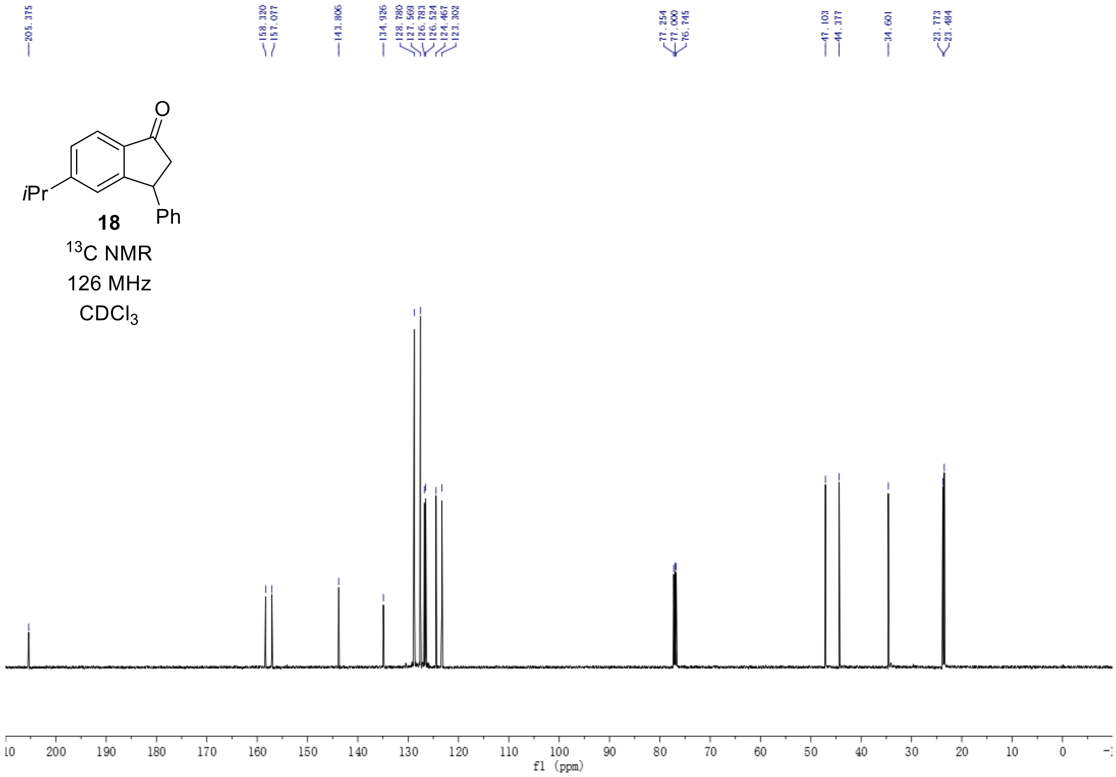

Supplementary Fig. 48 ^{13}C NMR spectrum of compound **18**

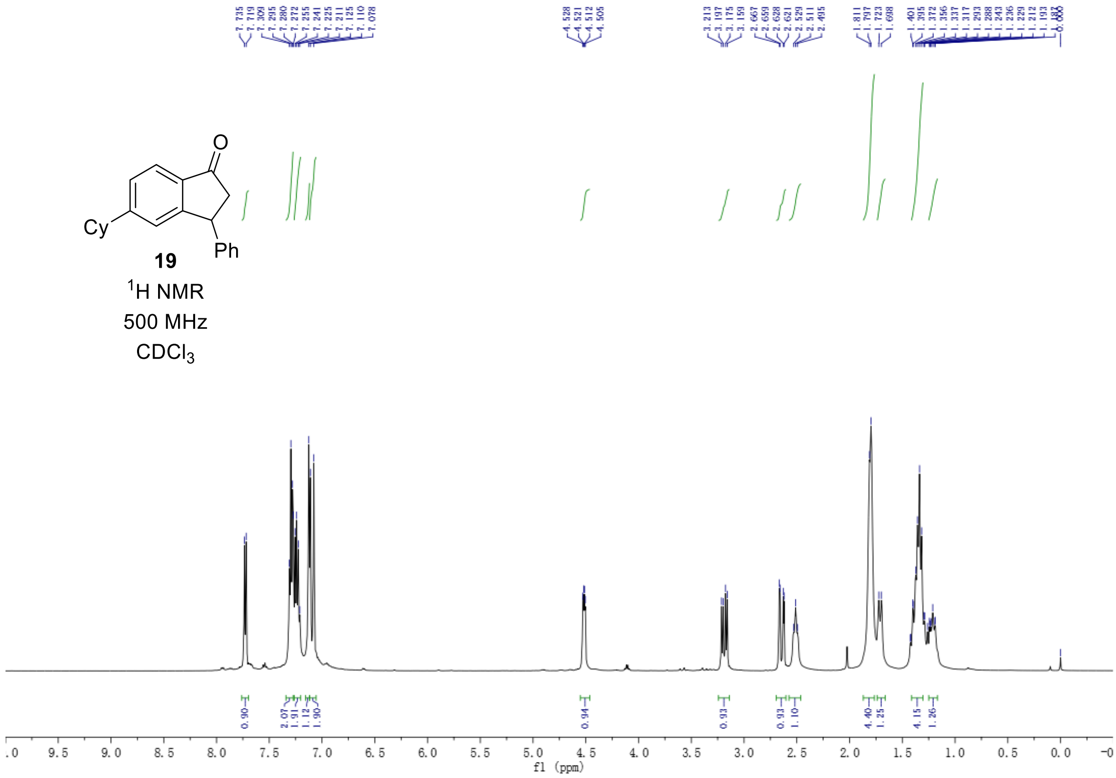

Supplementary Fig. 49 ^{1}H NMR spectrum of compound **19**

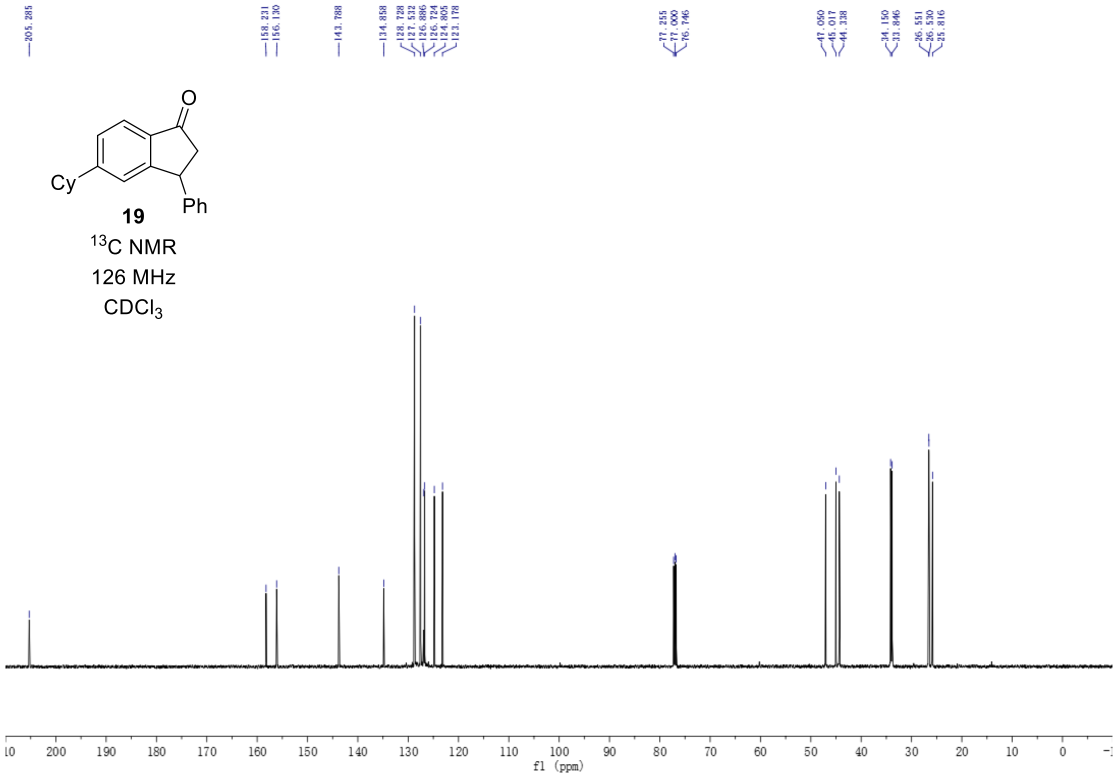

Supplementary Fig. 50 ^{13}C NMR spectrum of compound **19**

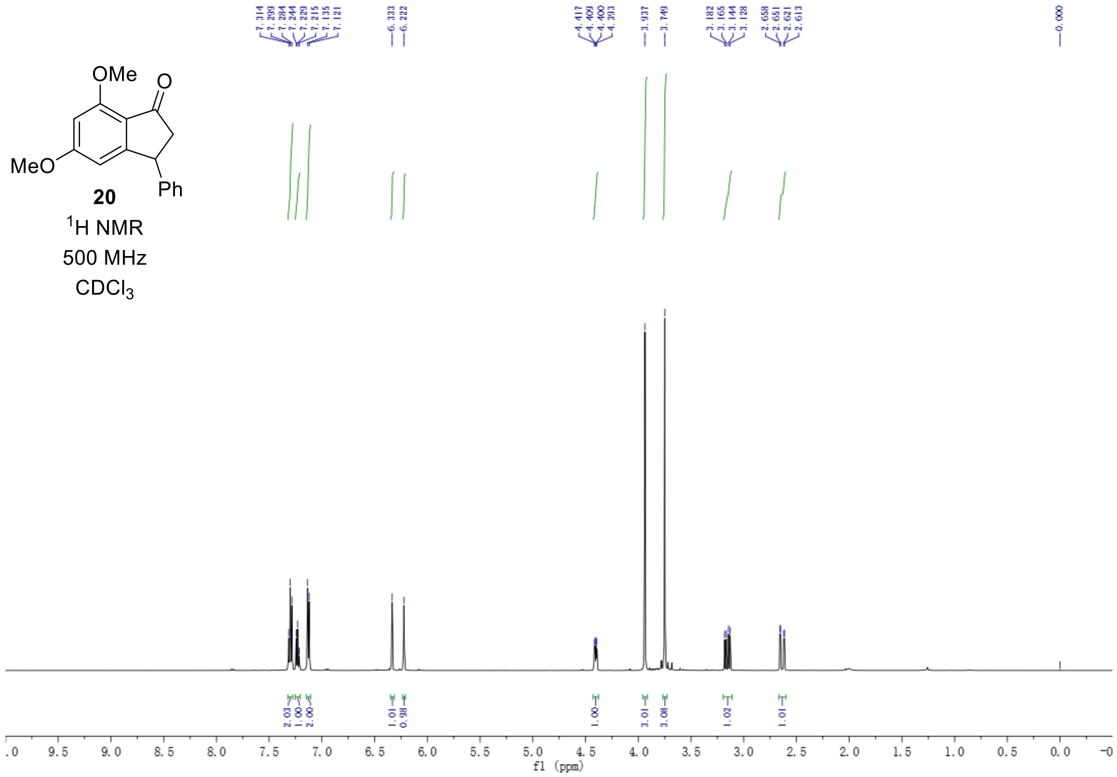

Supplementary Fig. 51 ^{1}H NMR spectrum of compound **20**

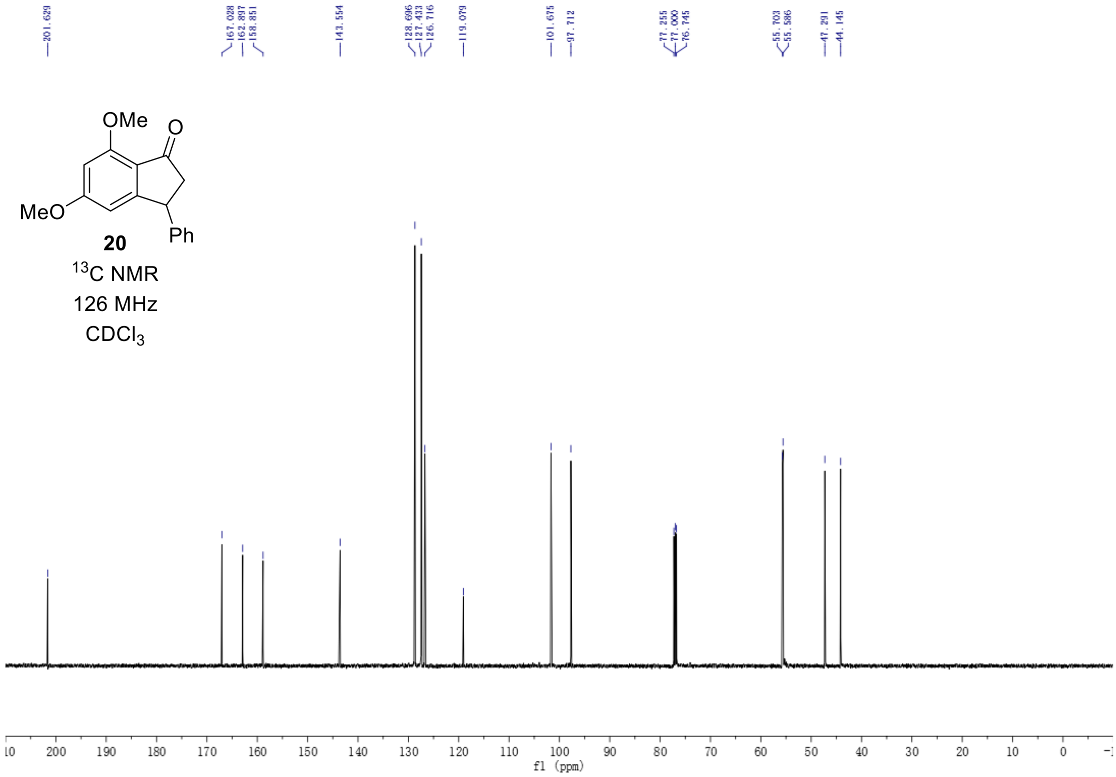

Supplementary Fig. 52 ^{13}C NMR spectrum of compound **20**

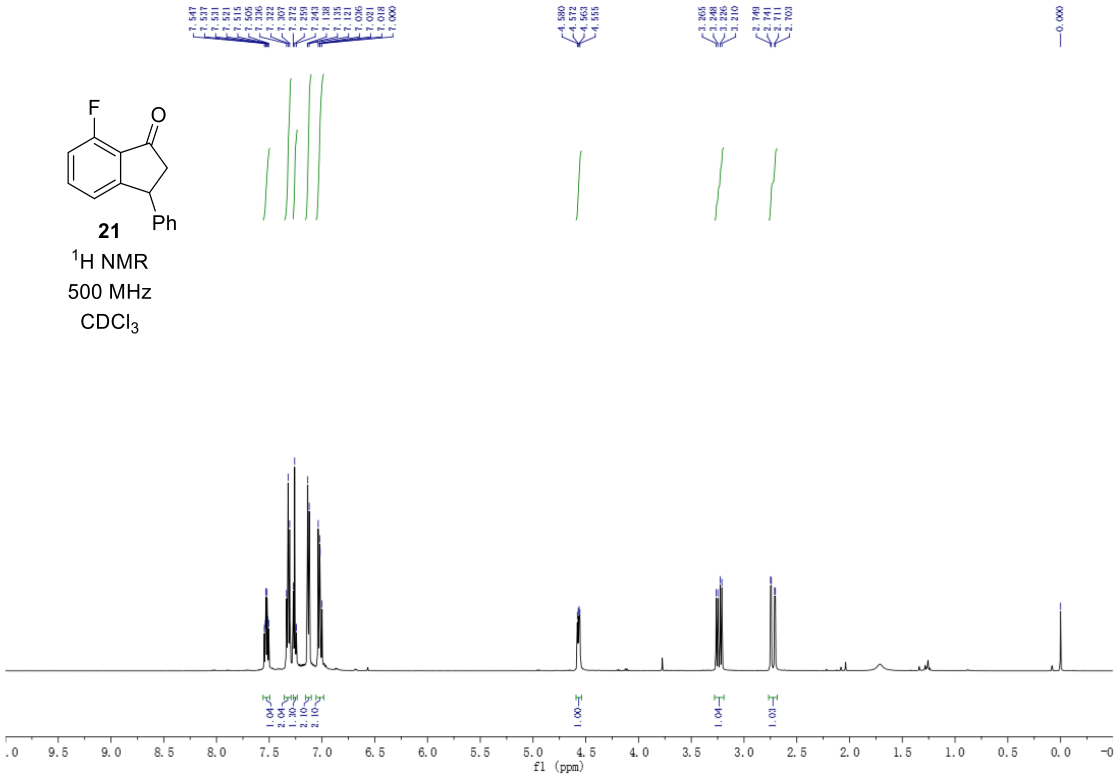

Supplementary Fig. 53 ^{1}H NMR spectrum of compound **21**

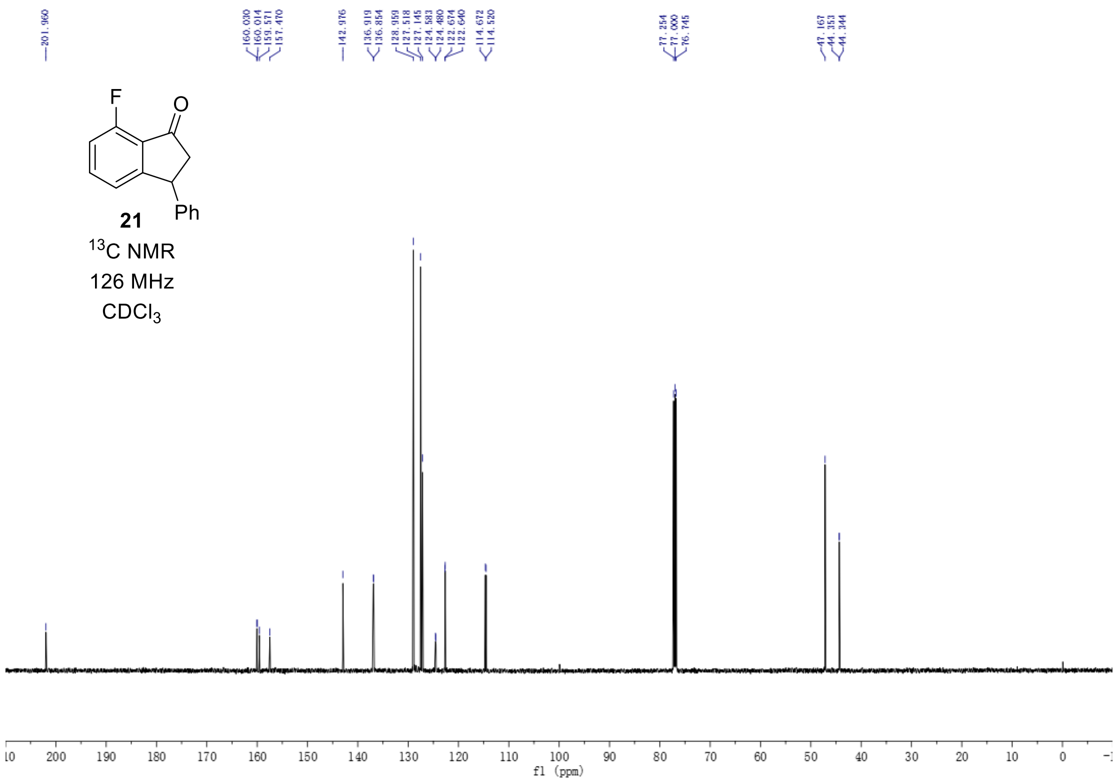

Supplementary Fig. 54 ^{13}C NMR spectrum of compound **21**

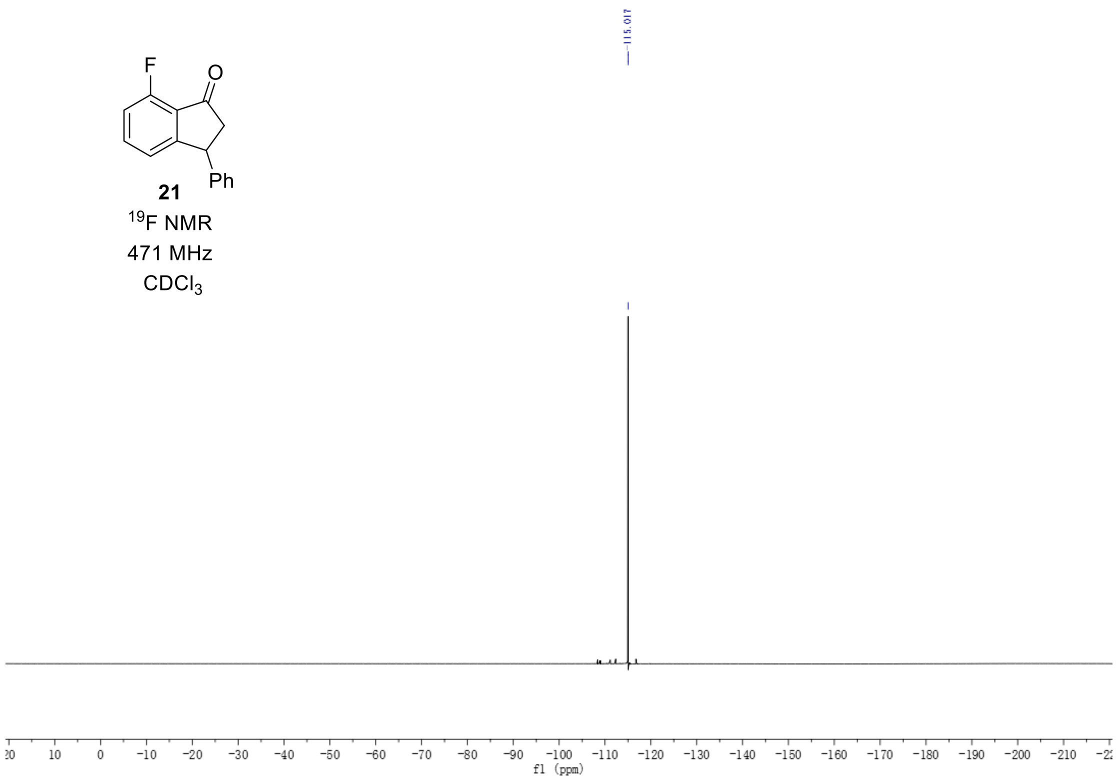

Supplementary Fig. 55 ^{19}F NMR spectrum of compound **21**

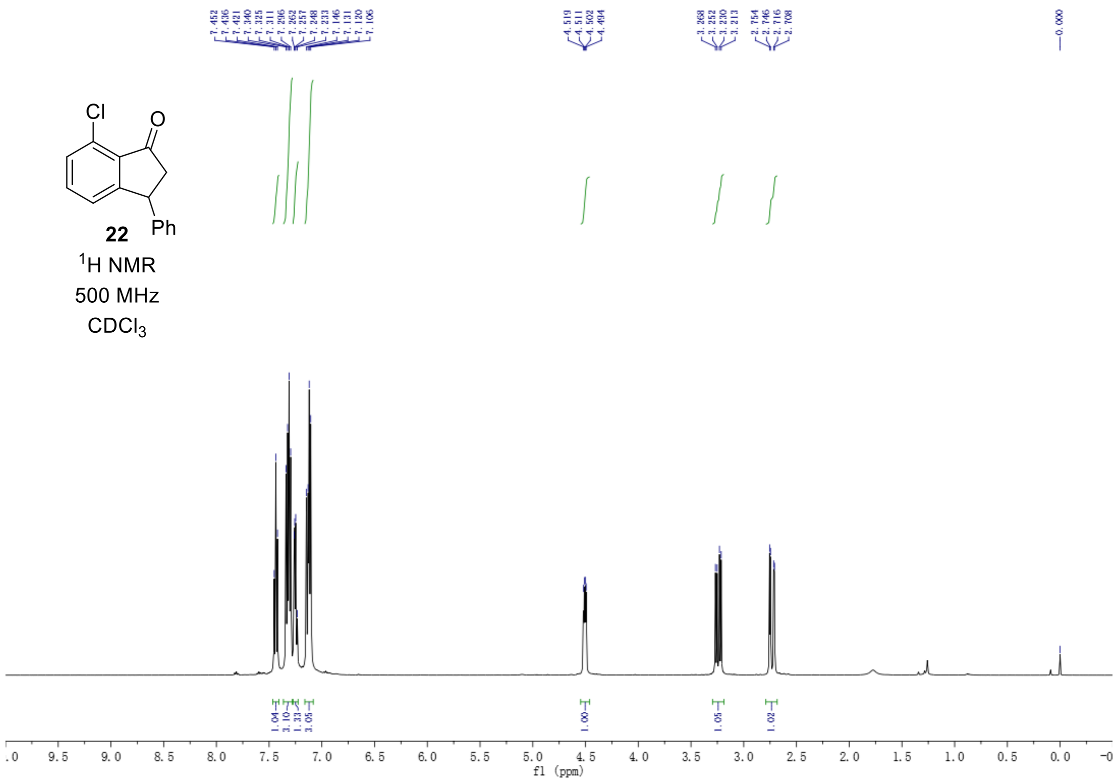

Supplementary Fig. 56 ^{1}H NMR spectrum of compound **22**

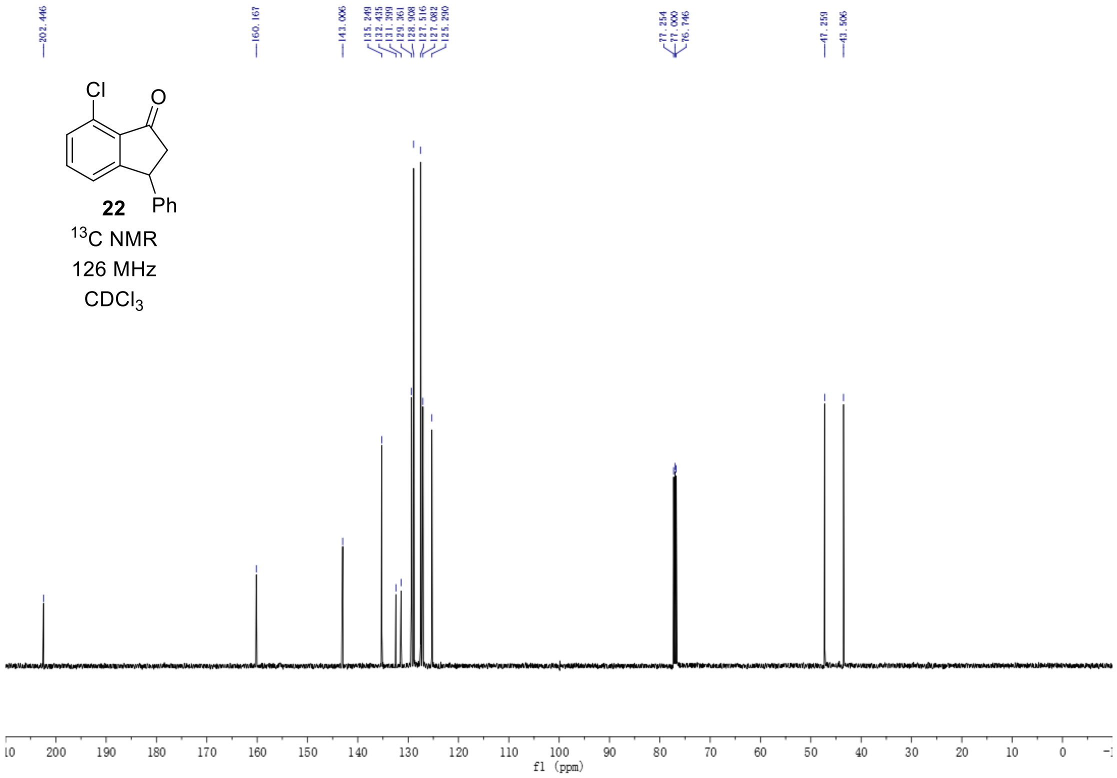

Supplementary Fig. 57 ^{13}C NMR spectrum of compound **22**

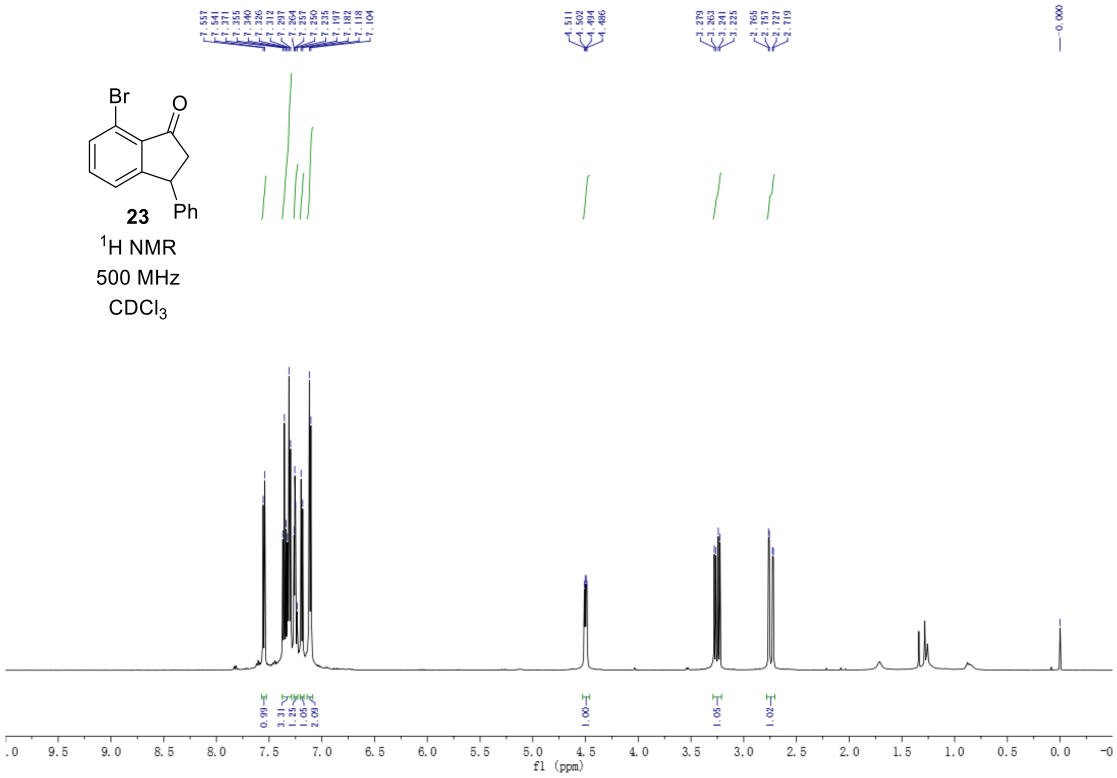

Supplementary Fig. 58 ^{1}H NMR spectrum of compound **23**

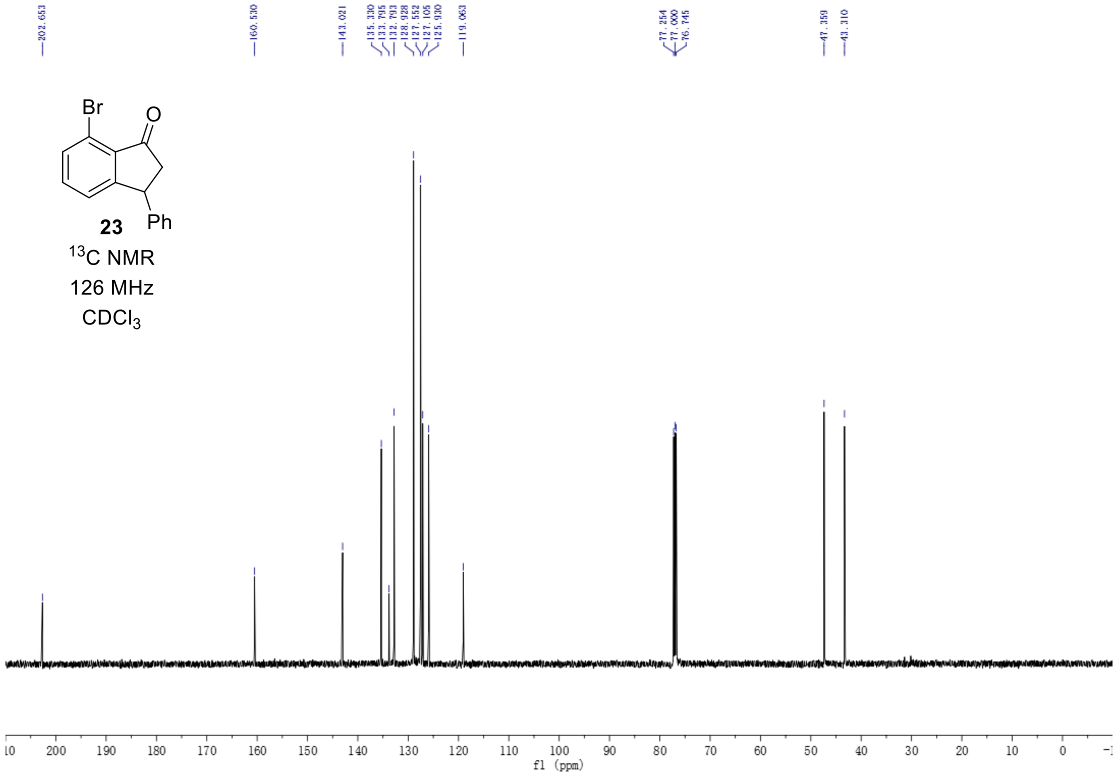

Supplementary Fig. 59 ^{13}C NMR spectrum of compound **23**

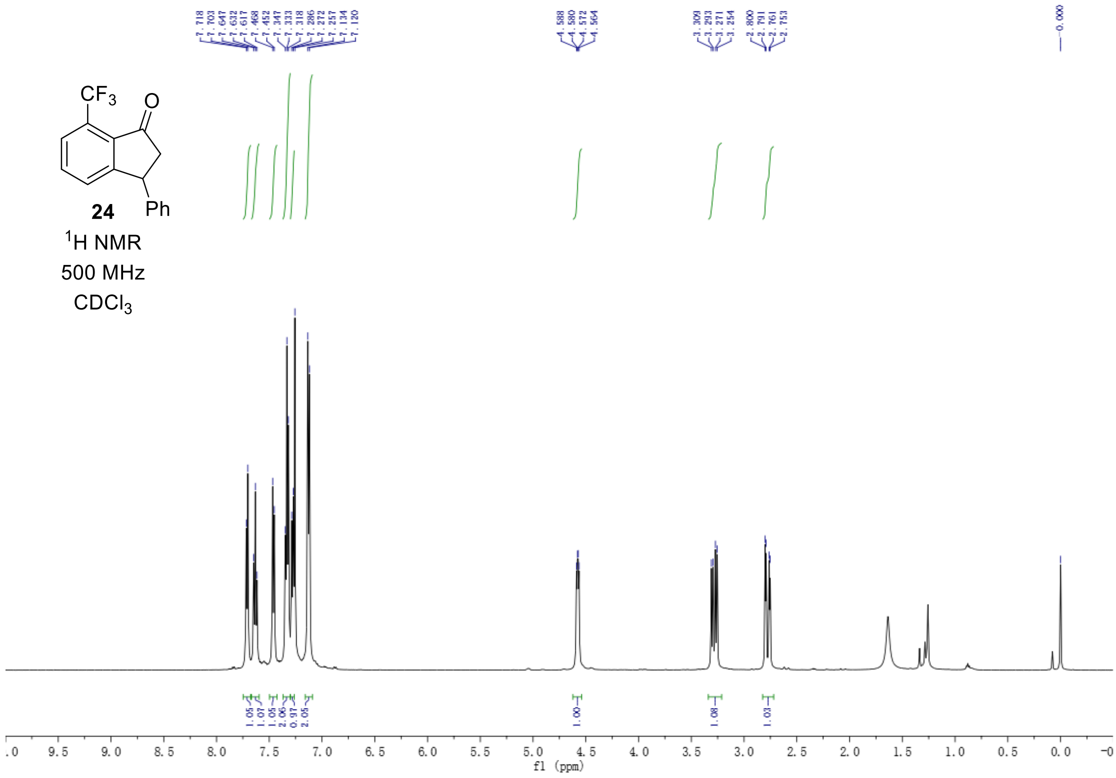

Supplementary Fig. 60 ^{1}H NMR spectrum of compound **24**

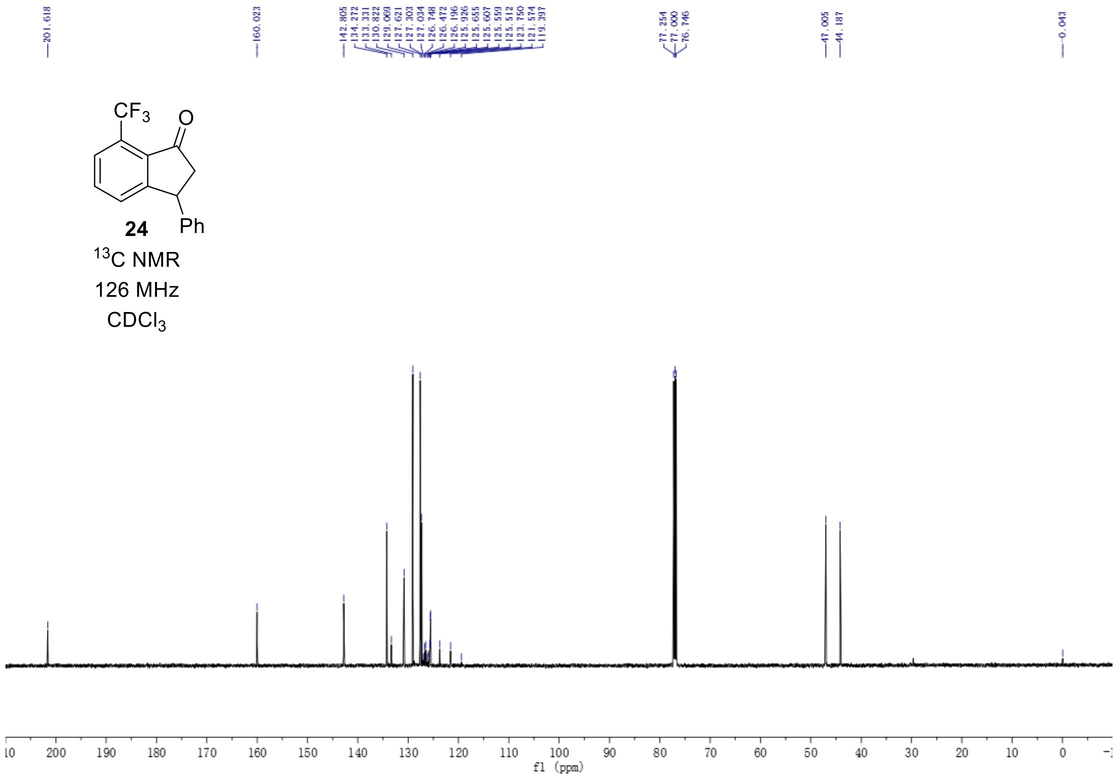

Supplementary Fig. 61 ^{13}C NMR spectrum of compound **24**

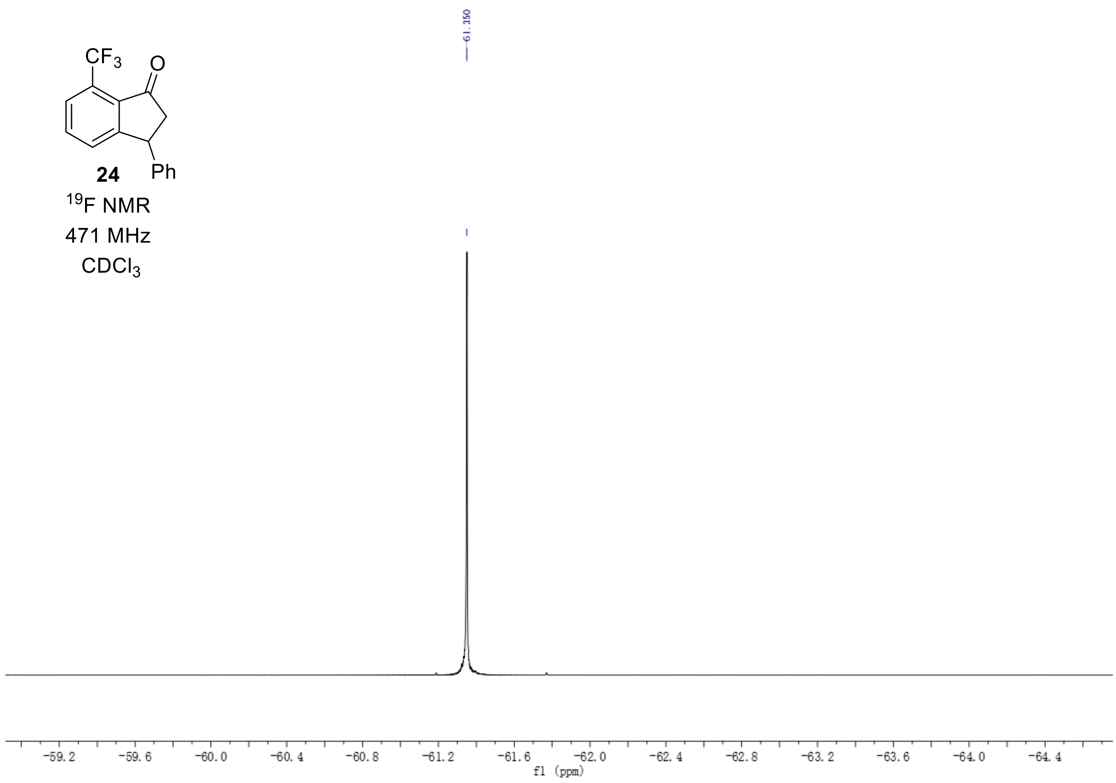

Supplementary Fig. 62 ^{19}F NMR spectrum of compound **24**

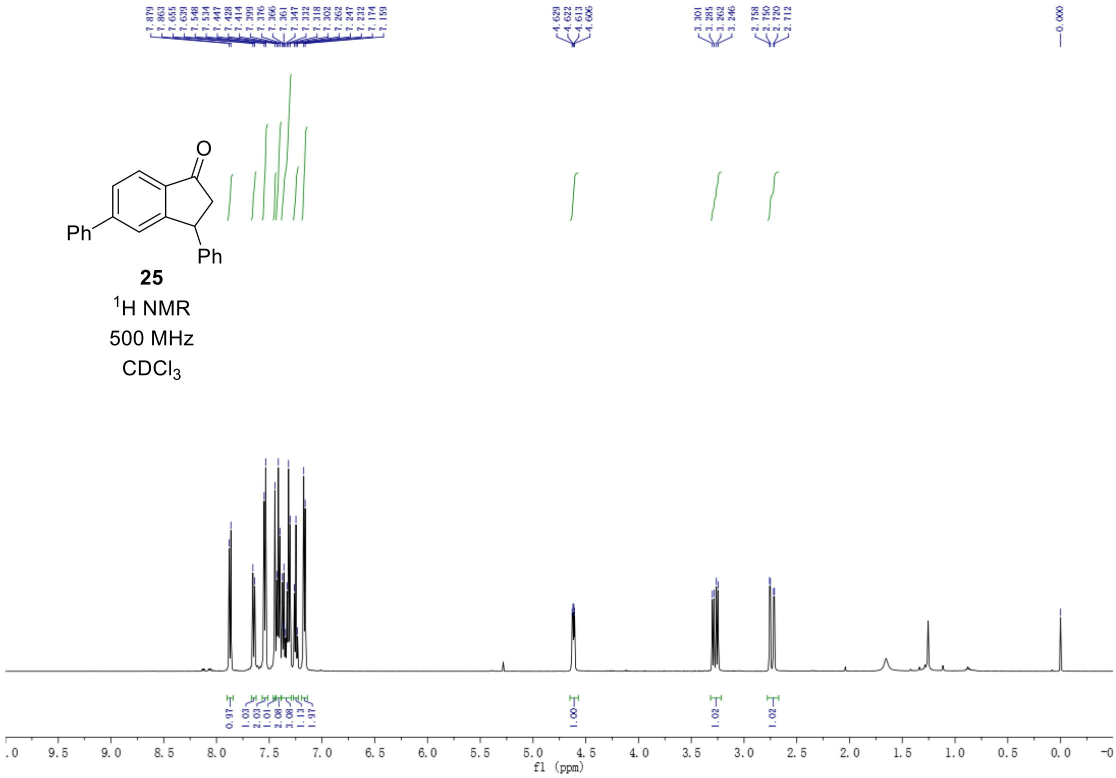

Supplementary Fig. 63 ^{1}H NMR spectrum of compound **25**

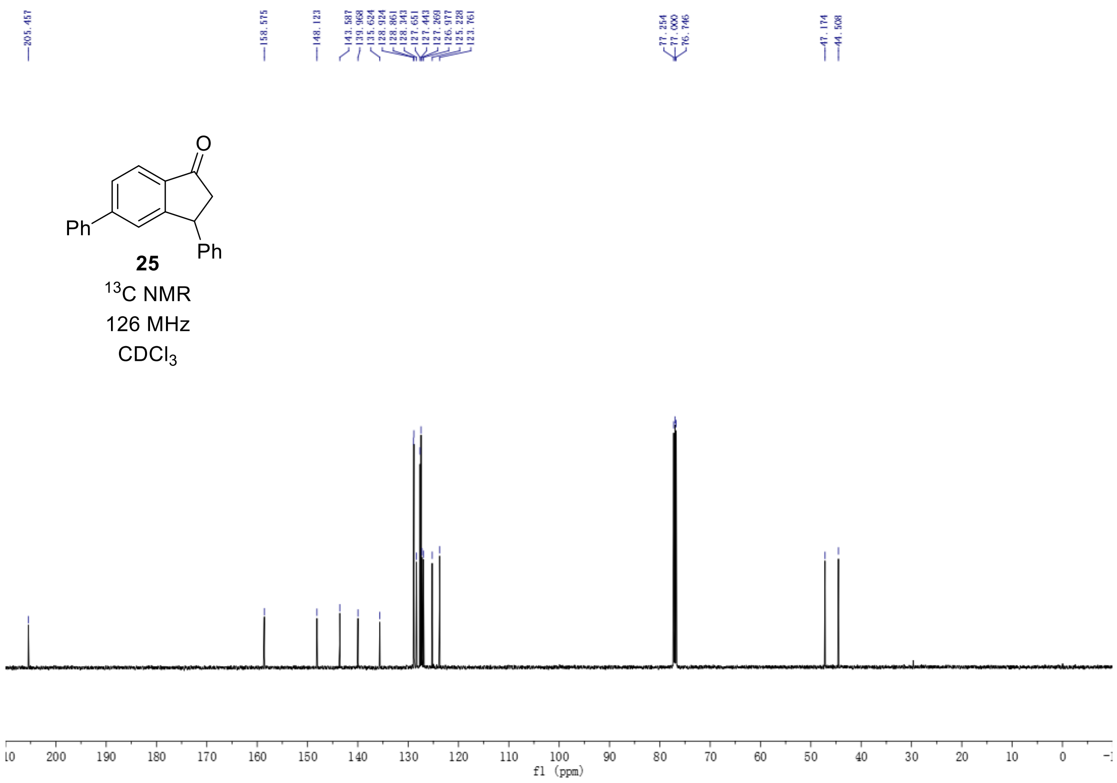

Supplementary Fig. 64 ^{13}C NMR spectrum of compound **25**

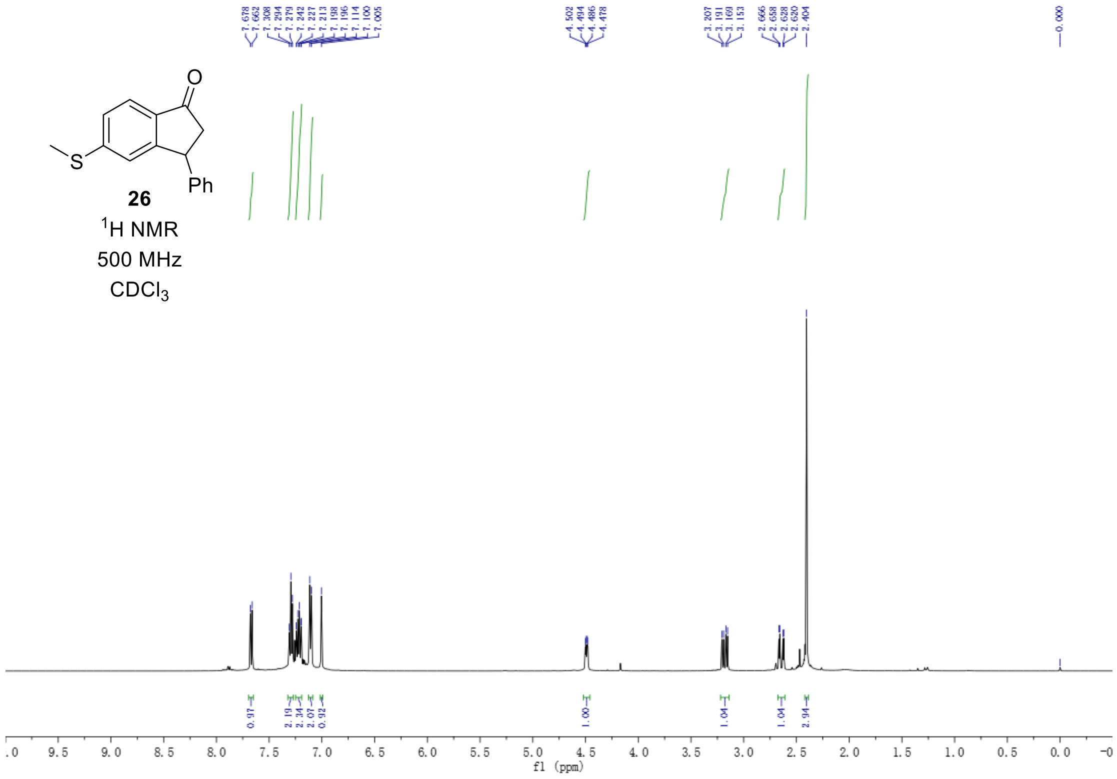

Supplementary Fig. 65 ^{1}H NMR spectrum of compound **26**

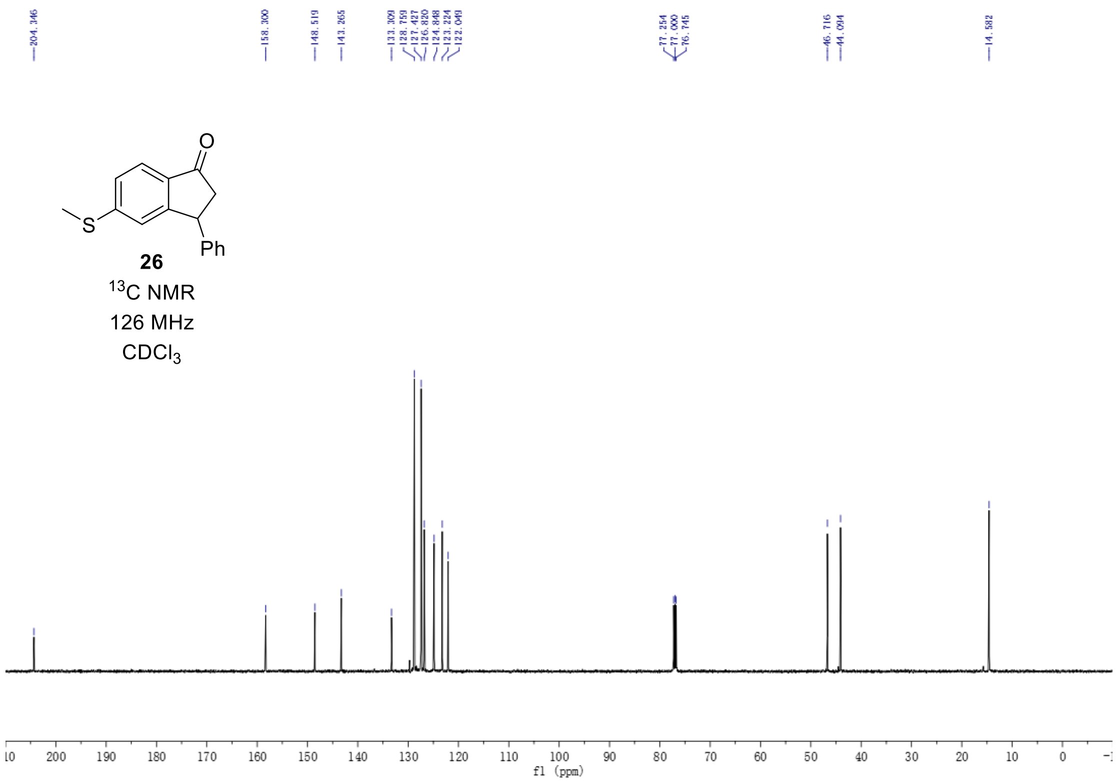

Supplementary Fig. 66 ^{13}C NMR spectrum of compound **26**

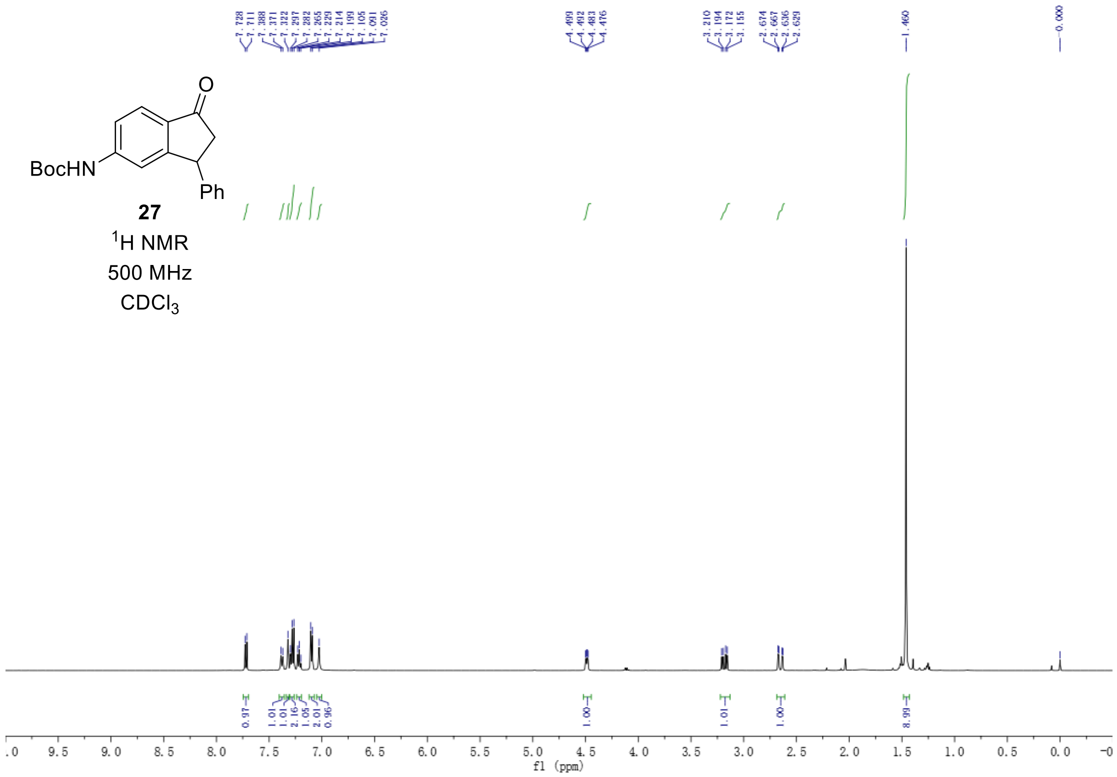

Supplementary Fig. 67 ^{1}H NMR spectrum of compound **27**

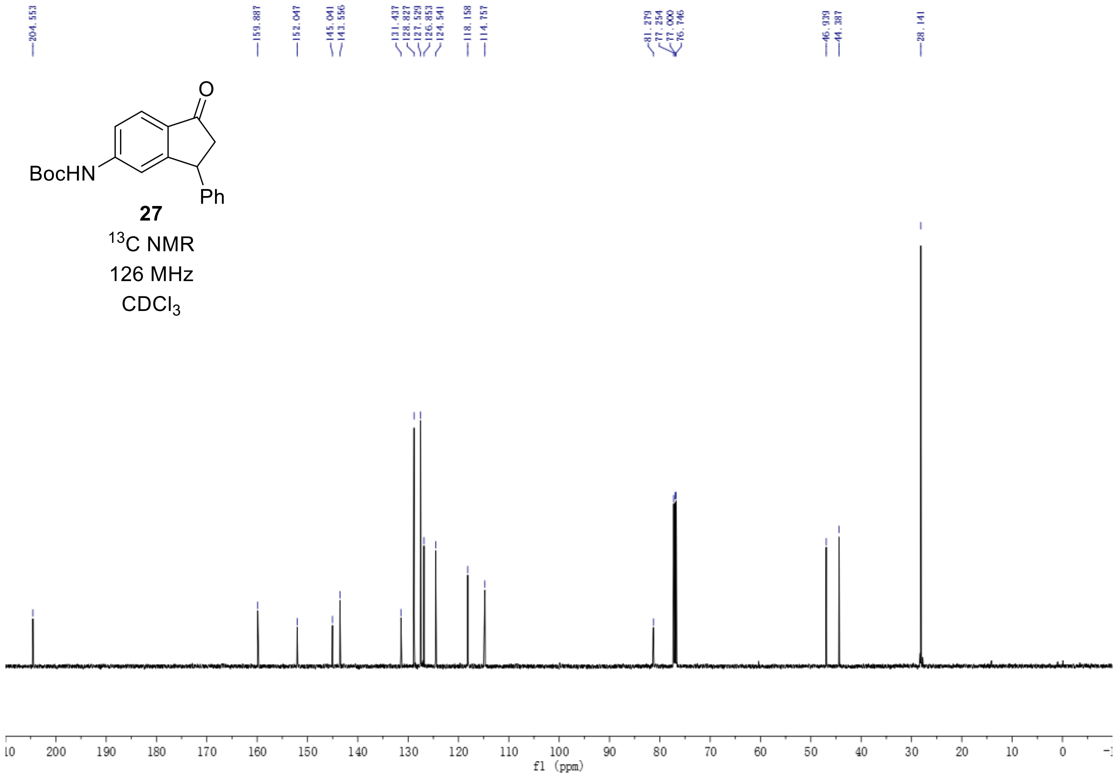

Supplementary Fig. 68 ^{13}C NMR spectrum of compound **27**

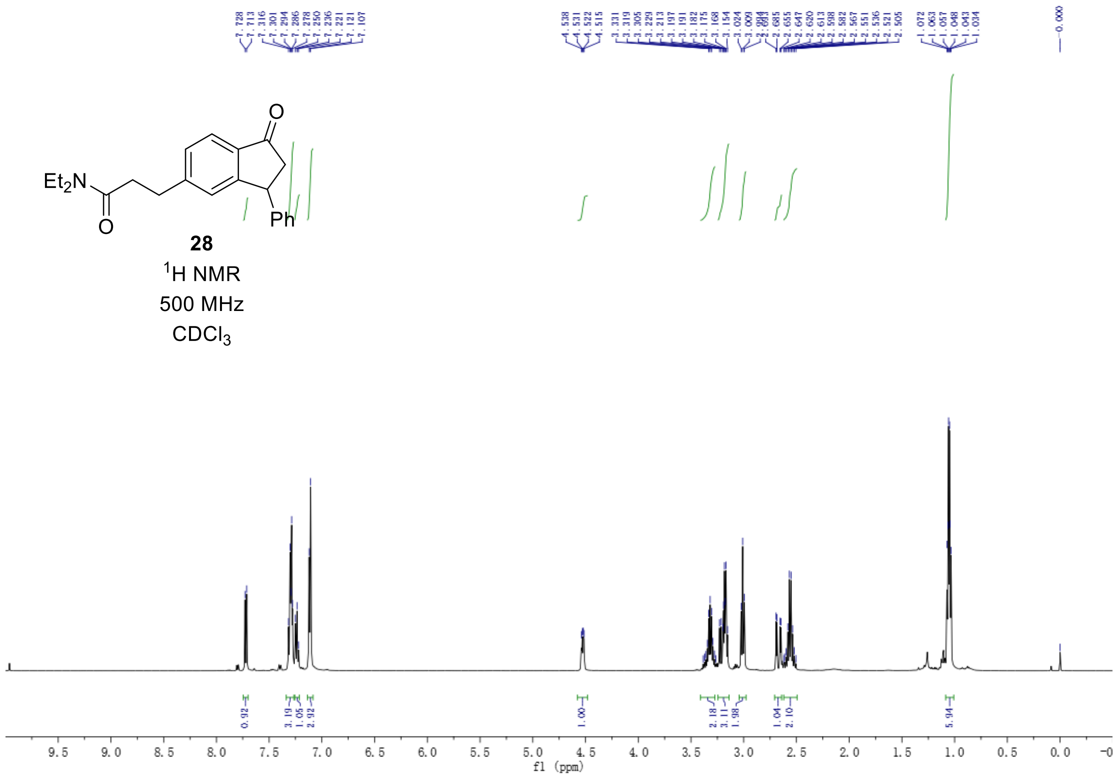

Supplementary Fig. 69 ^{1}H NMR spectrum of compound **28**

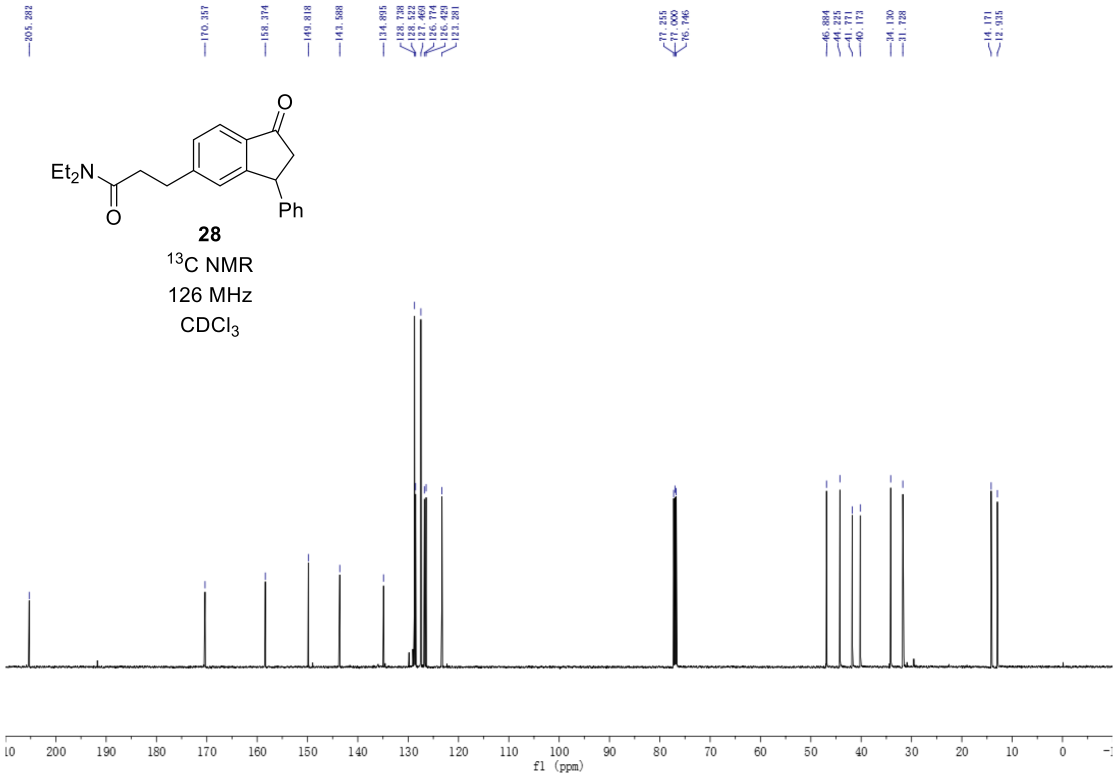

Supplementary Fig. 70 ^{13}C NMR spectrum of compound **28**

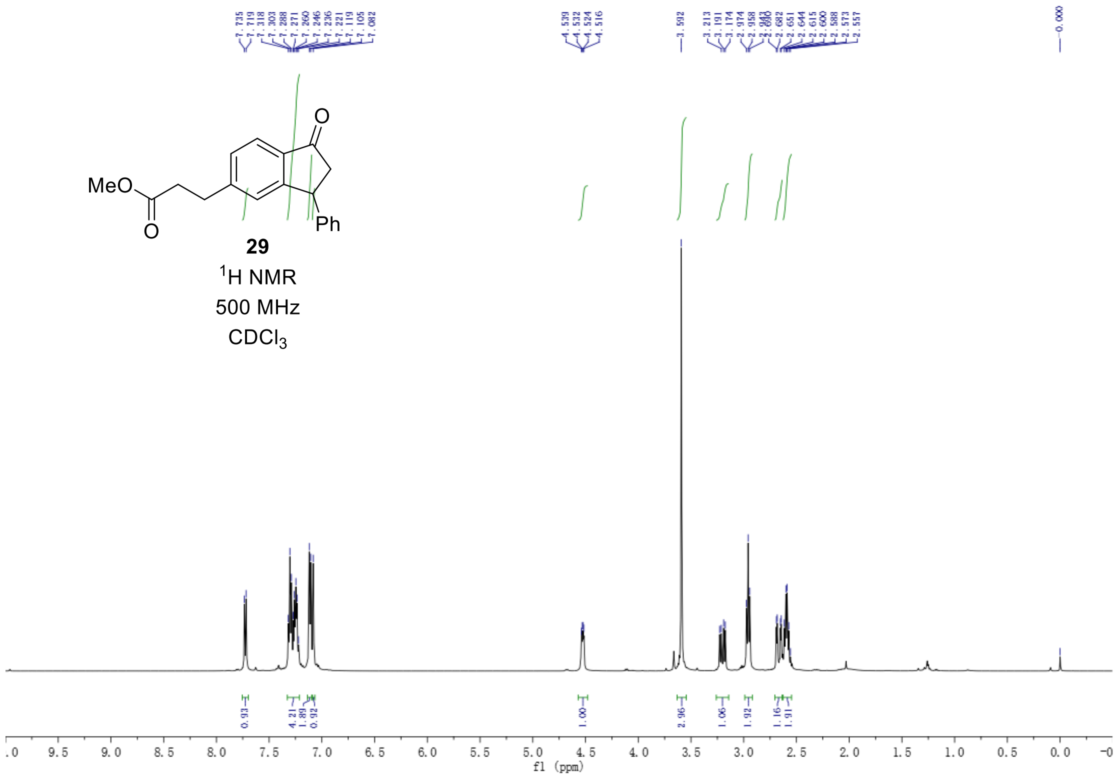

Supplementary Fig. 71 ^{1}H NMR spectrum of compound **29**

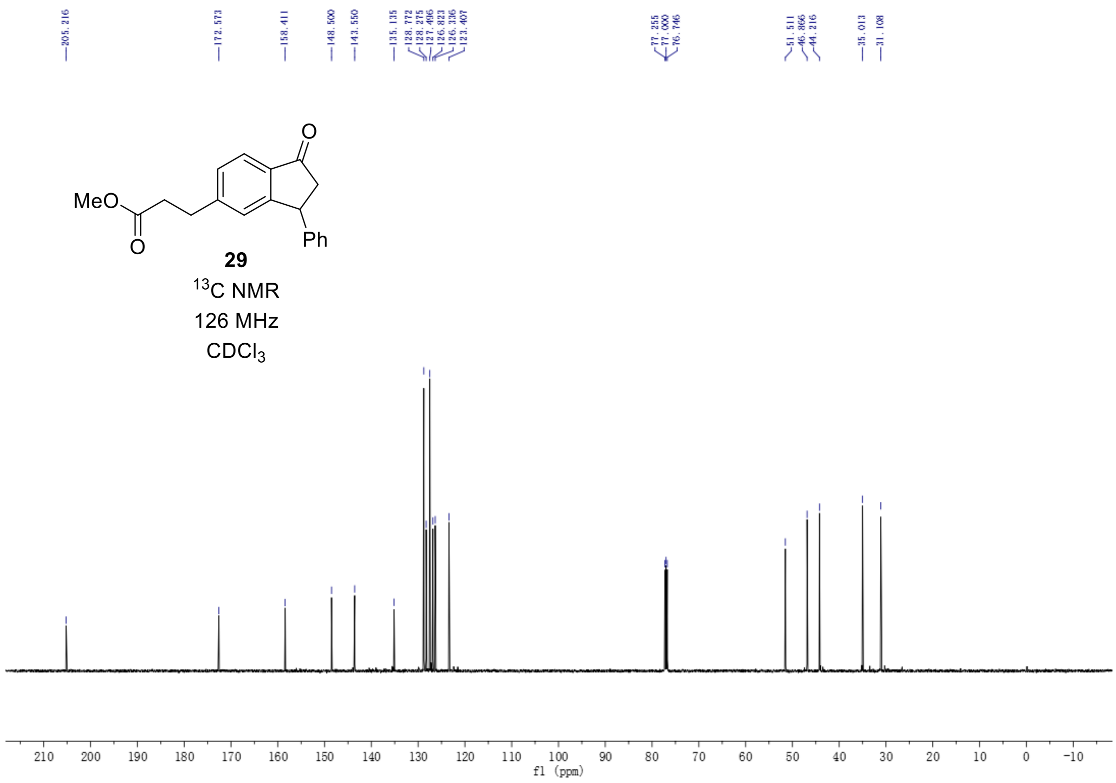

Supplementary Fig. 72 ^{13}C NMR spectrum of compound **29**

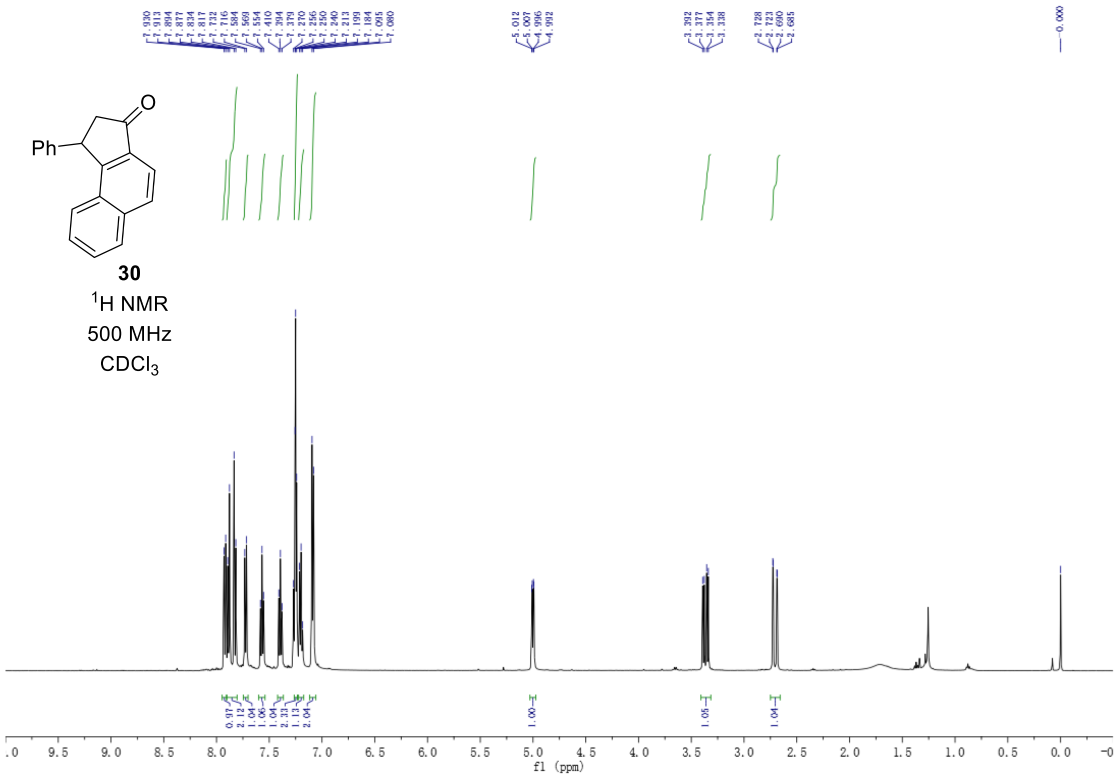

Supplementary Fig. 73 ^{1}H NMR spectrum of compound **30**

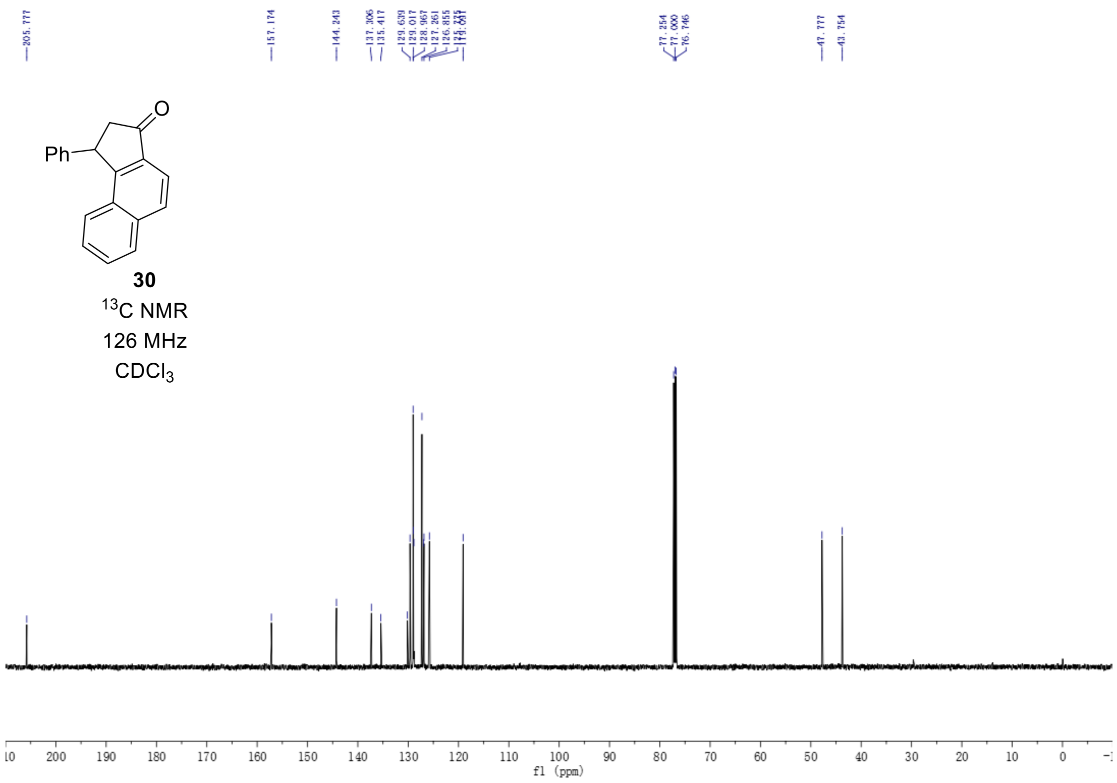

Supplementary Fig. 74 ^{13}C NMR spectrum of compound **30**

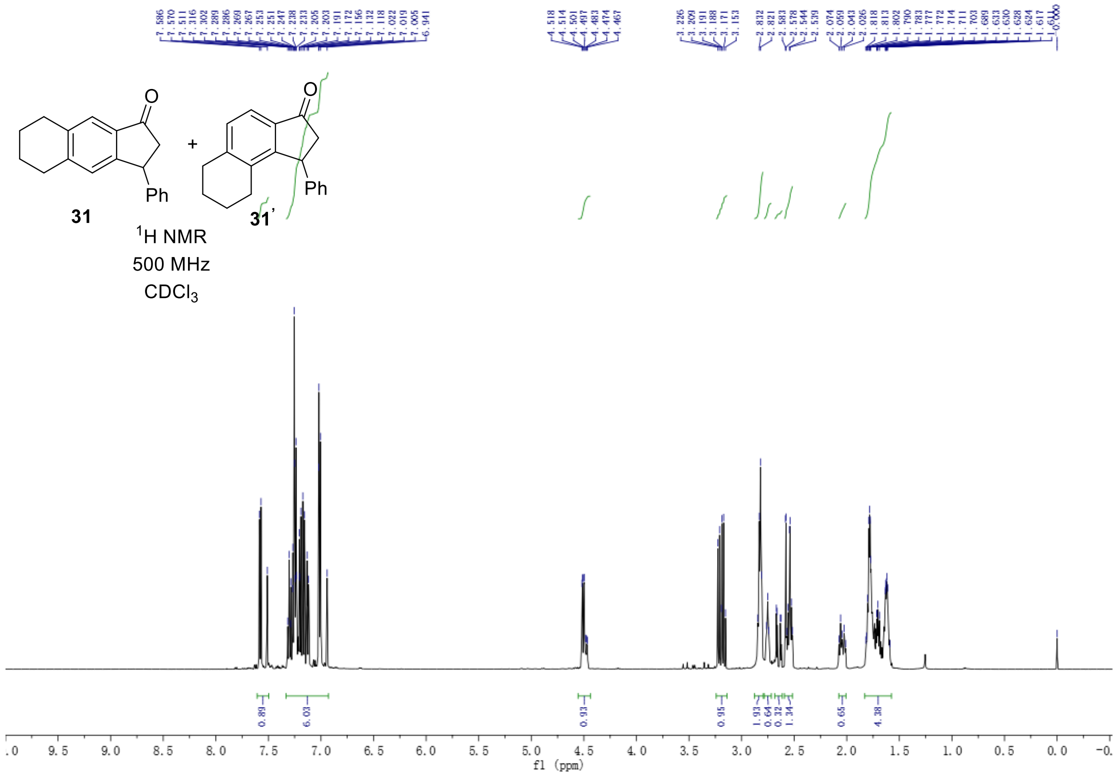

Supplementary Fig. 75 ^{1}H NMR spectrum of compound **31** and **31’**

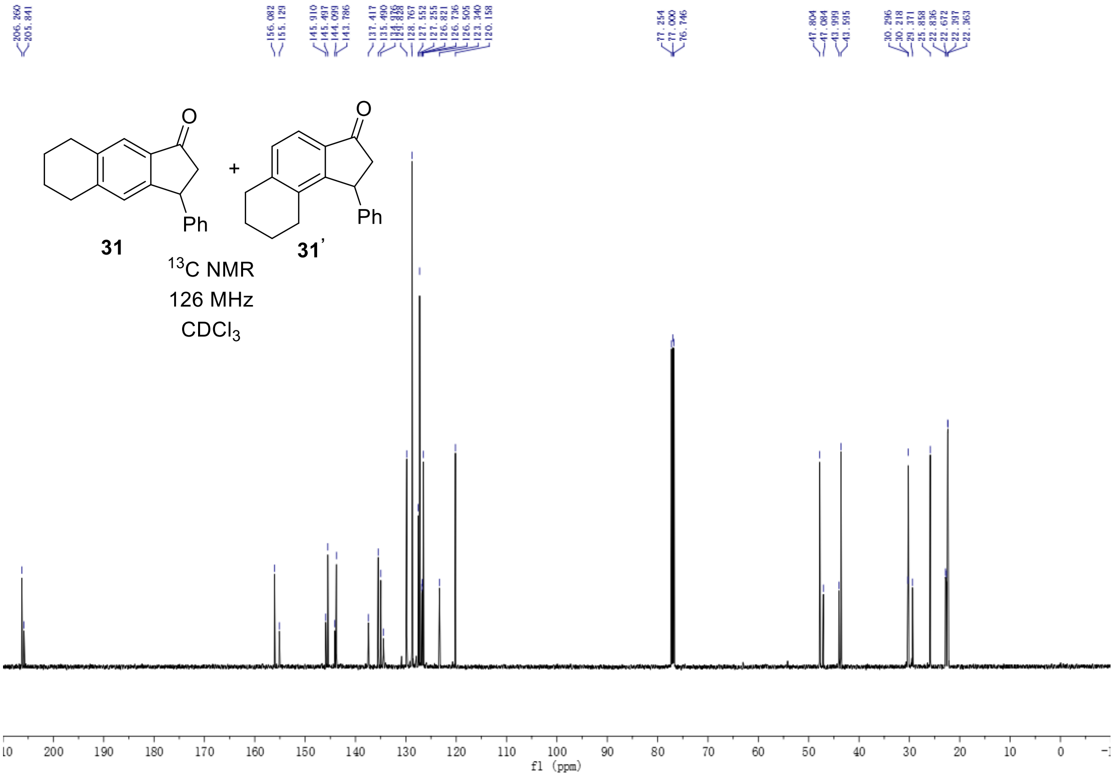

Supplementary Fig. 76 ^{13}C NMR spectrum of compound **31** and **31’**

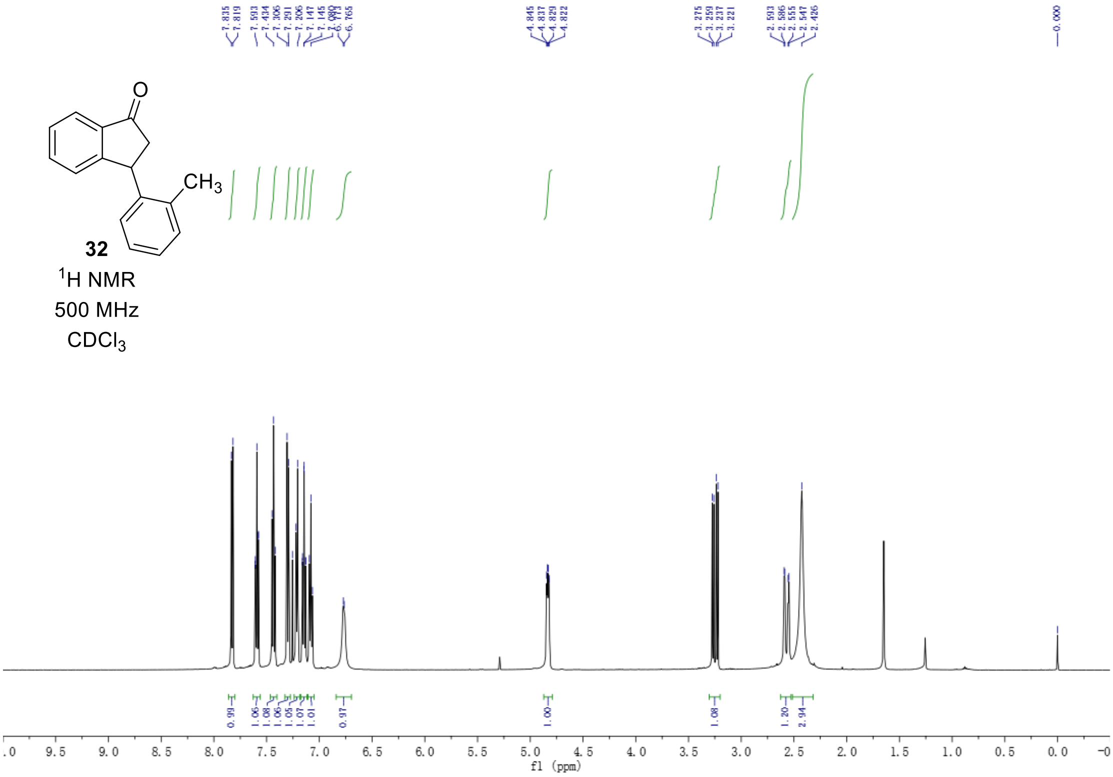

Supplementary Fig. 77 ^{1}H NMR spectrum of compound **32**

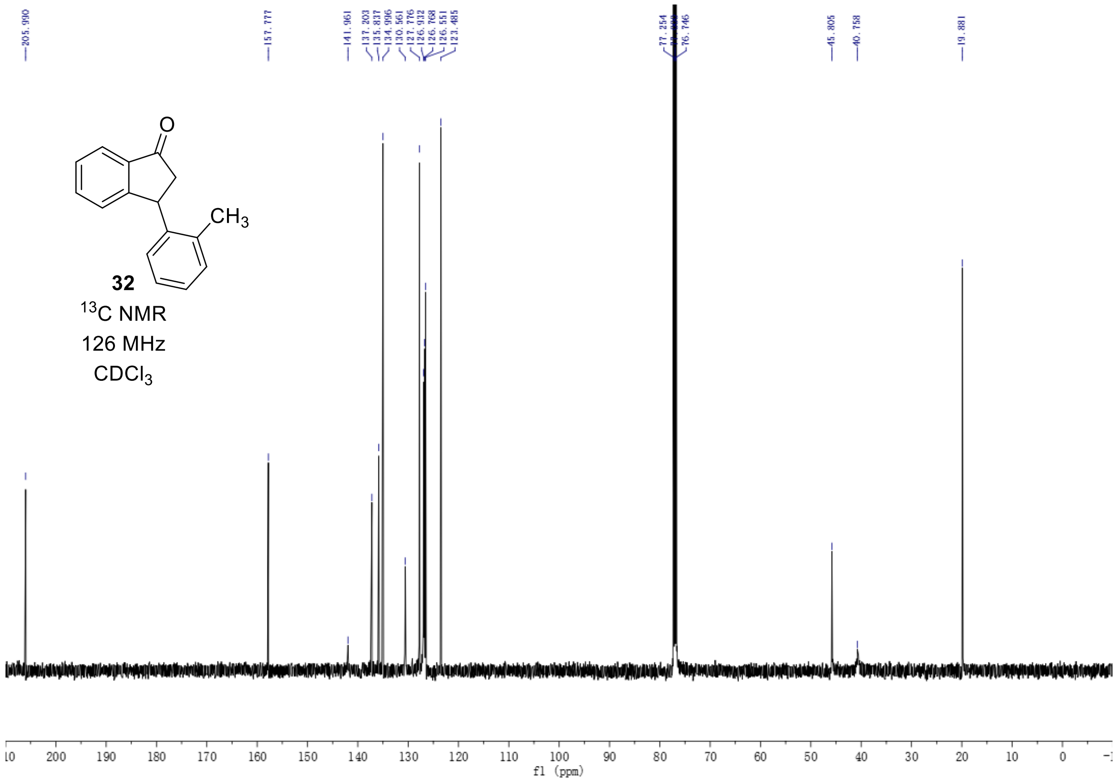

Supplementary Fig. 78 ^{13}C NMR spectrum of compound **32**

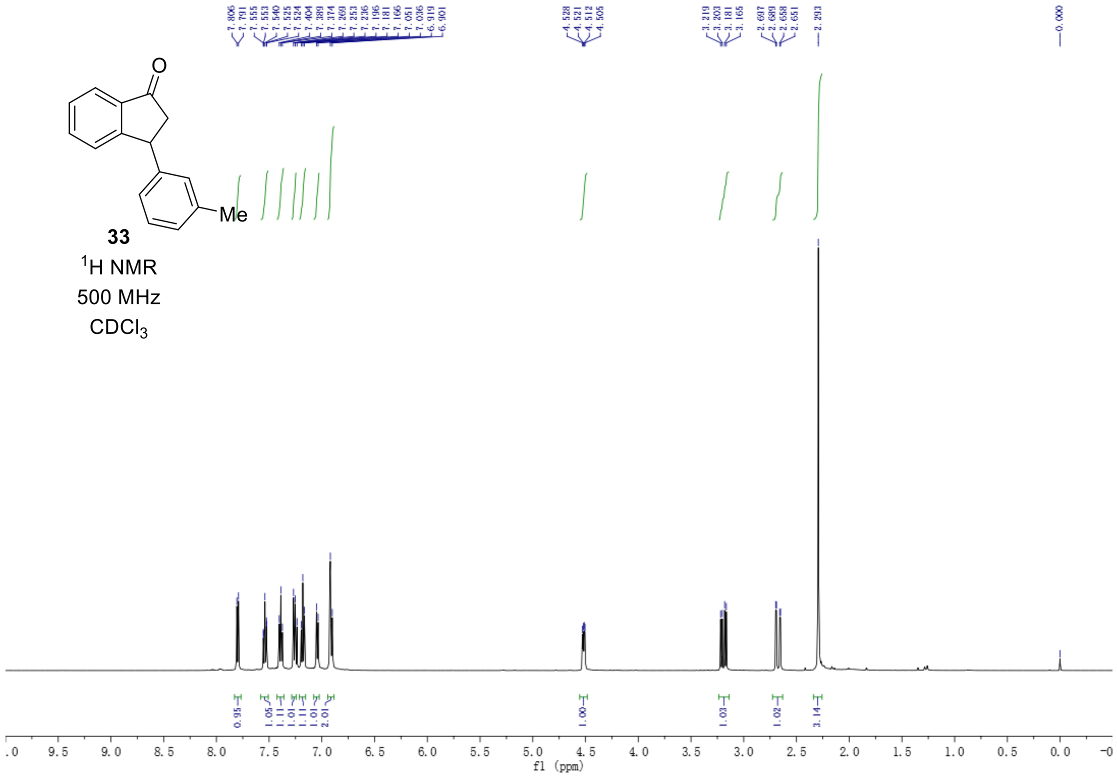

Supplementary Fig. 79 ^{1}H NMR spectrum of compound **33**

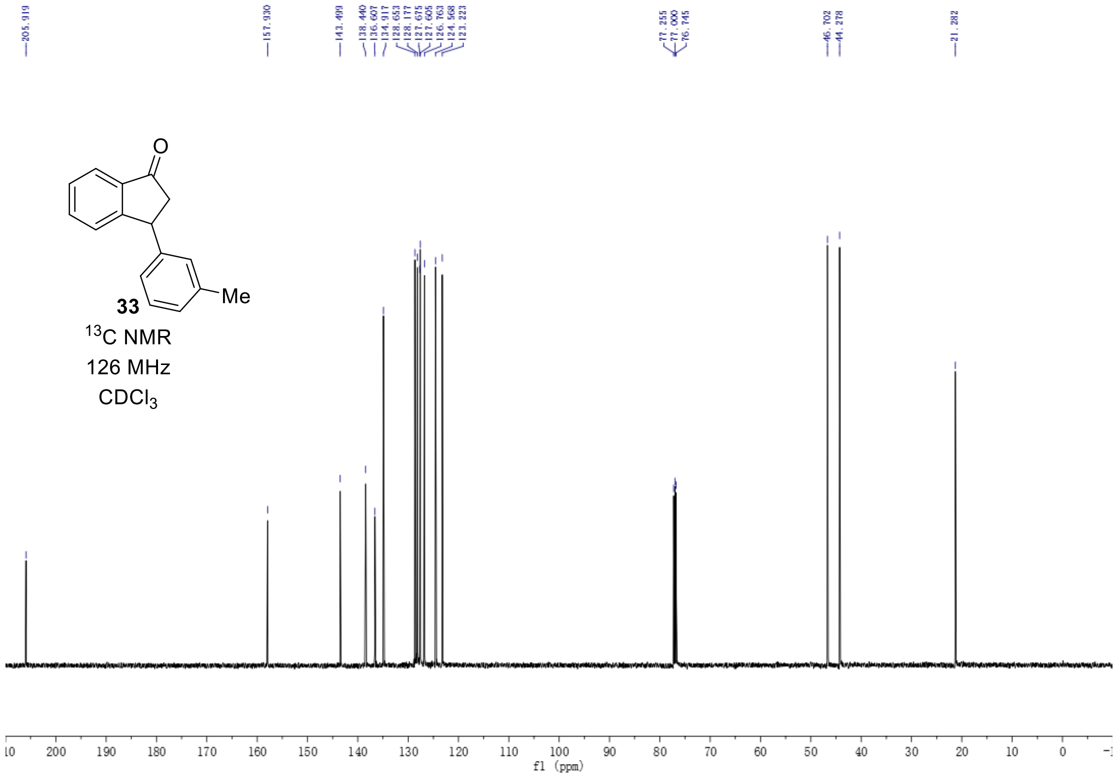

Supplementary Fig. 80 ^{13}C NMR spectrum of compound **33**

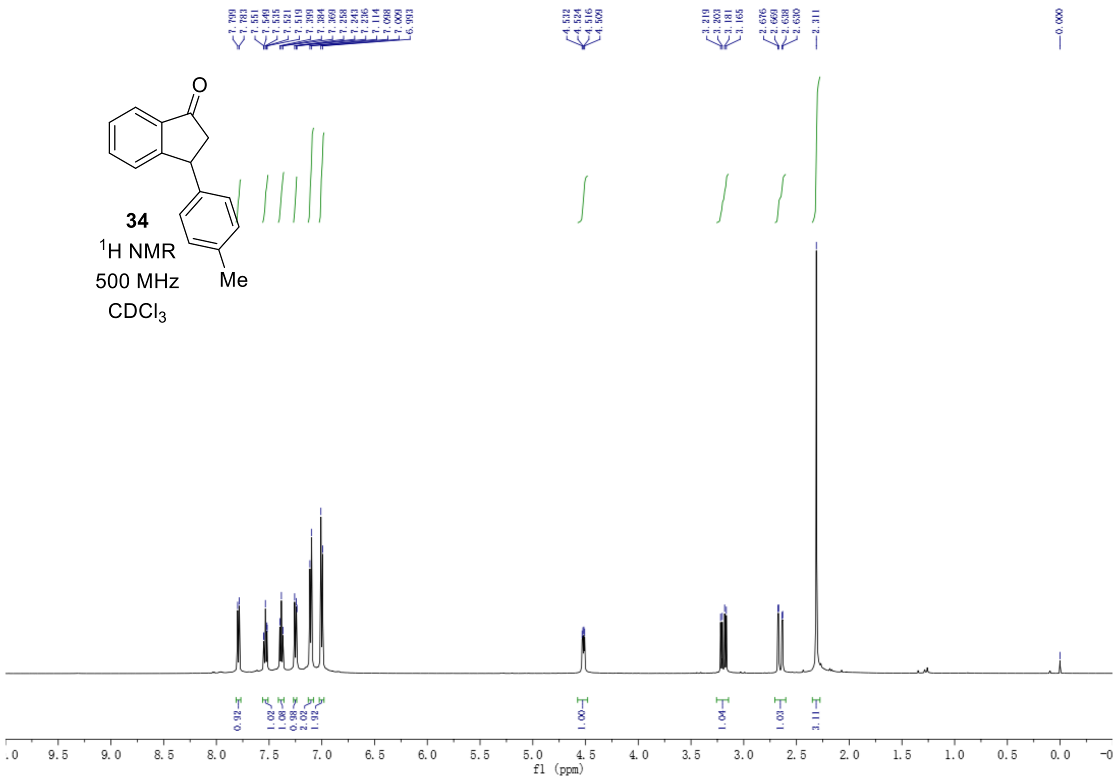

Supplementary Fig. 81 ^{1}H NMR spectrum of compound **34**

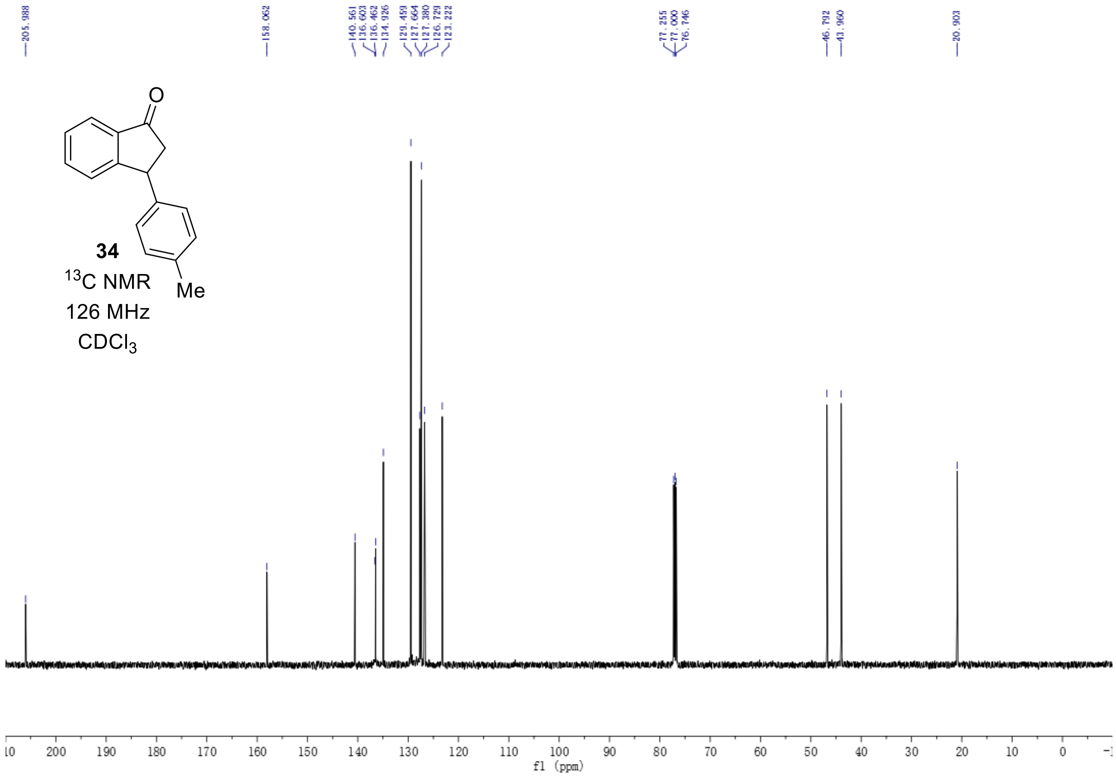

Supplementary Fig. 82 ^{13}C NMR spectrum of compound **34**

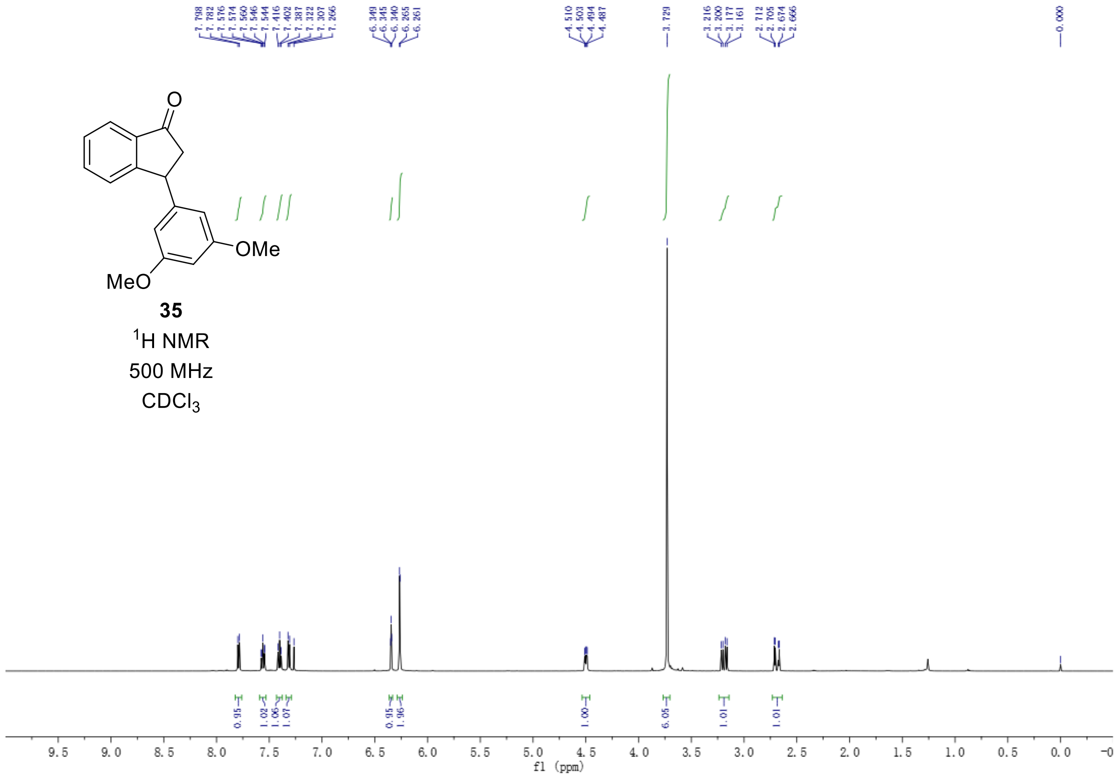

Supplementary Fig. 83 ^{1}H NMR spectrum of compound **35**

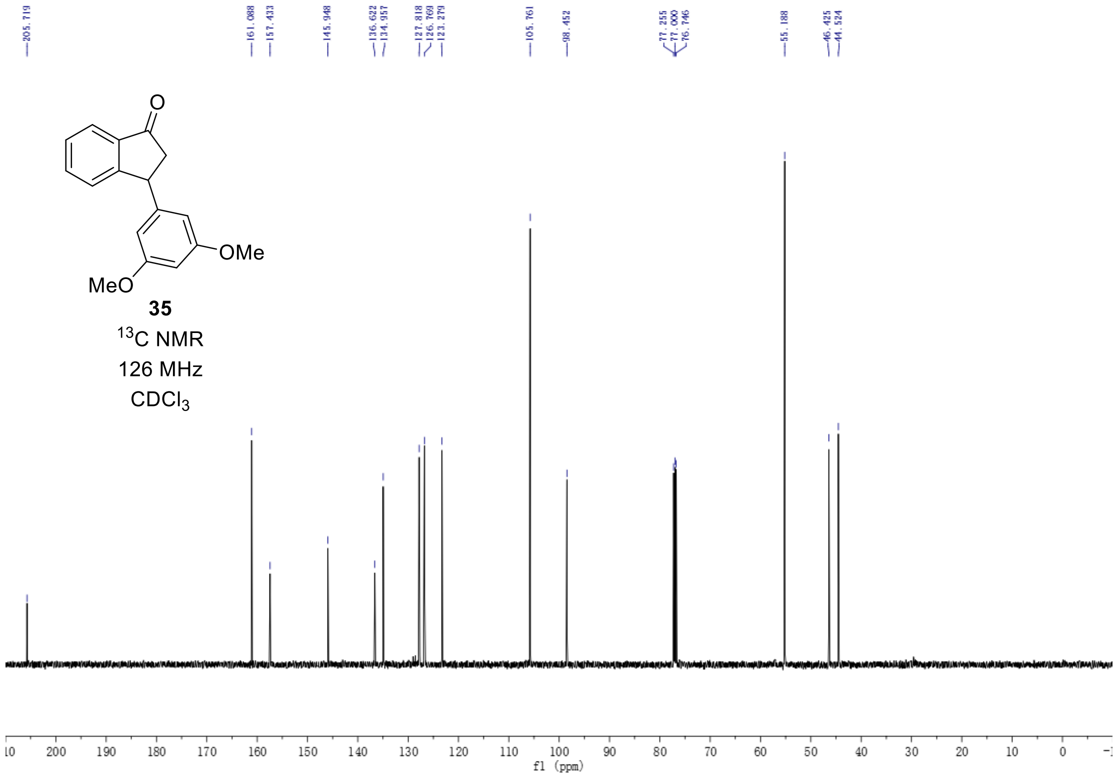

Supplementary Fig. 84 ^{13}C NMR spectrum of compound **35**

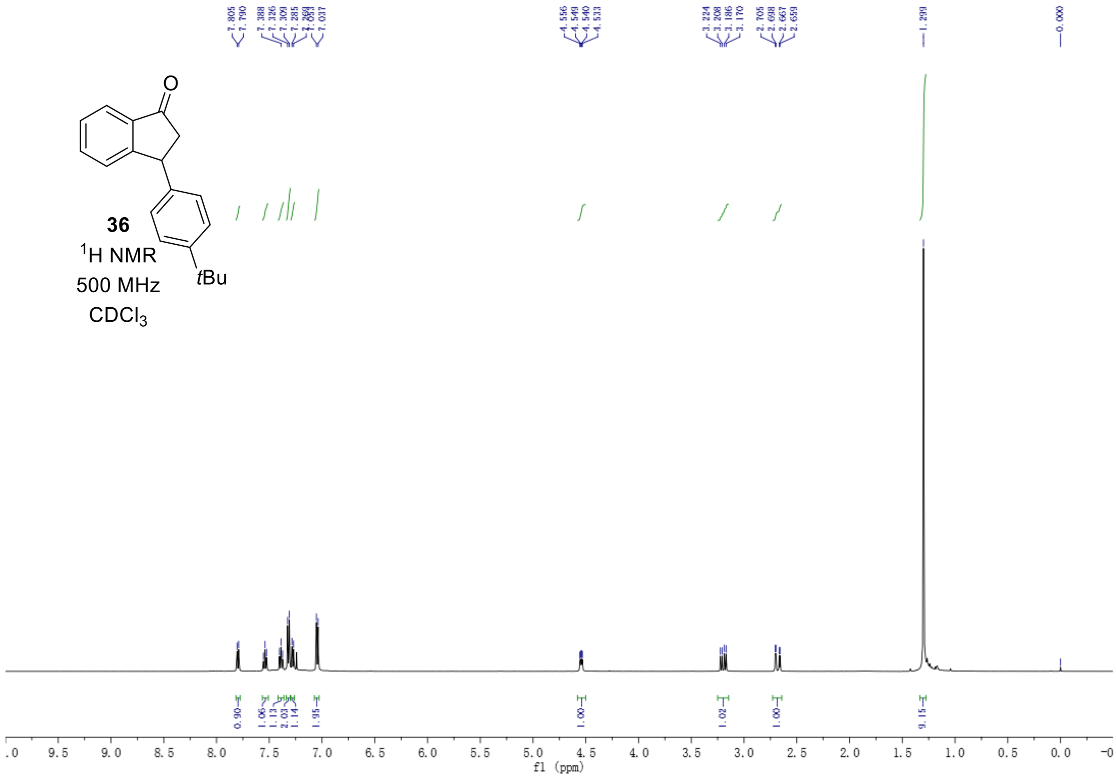

Supplementary Fig. 85 ^{1}H NMR spectrum of compound **36**

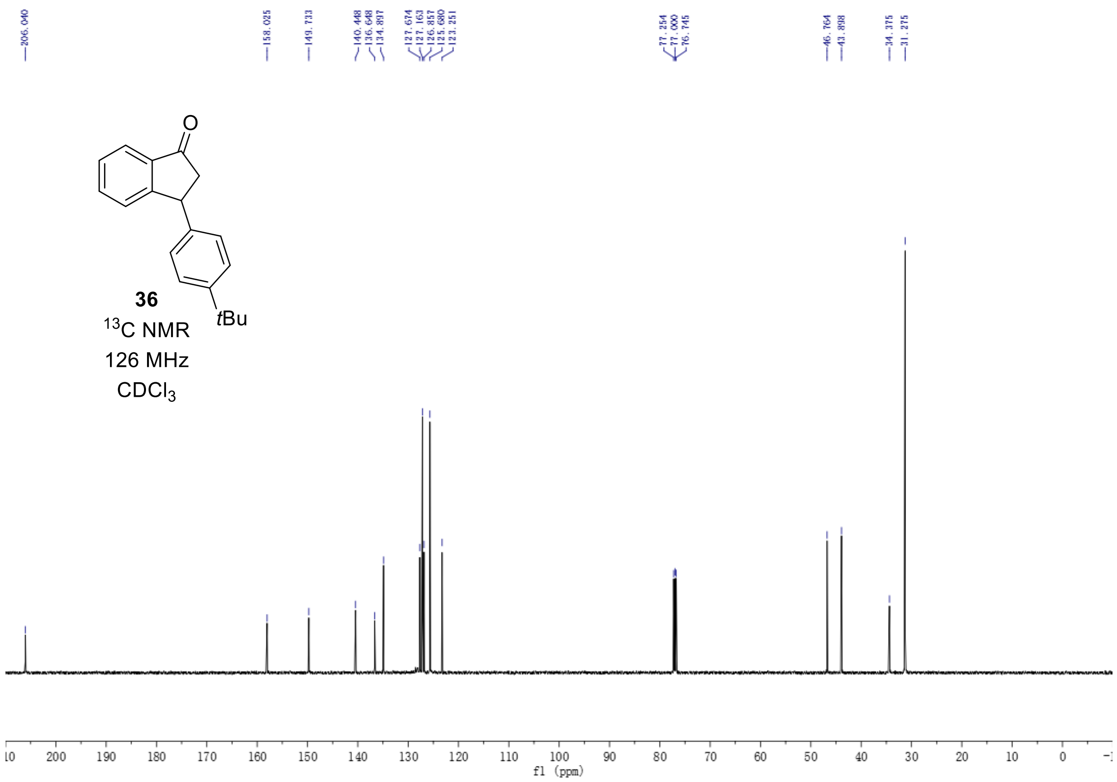

Supplementary Fig. 86 ^{13}C NMR spectrum of compound **36**

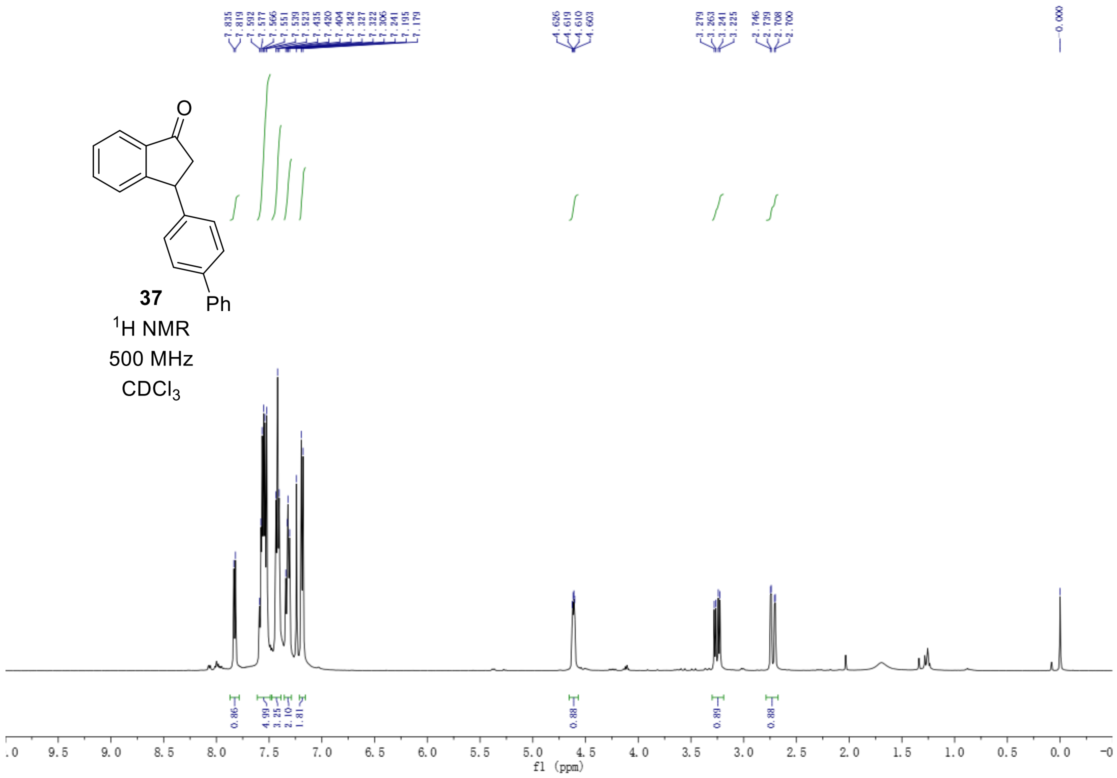

Supplementary Fig. 87 ^{1}H NMR spectrum of compound **37**

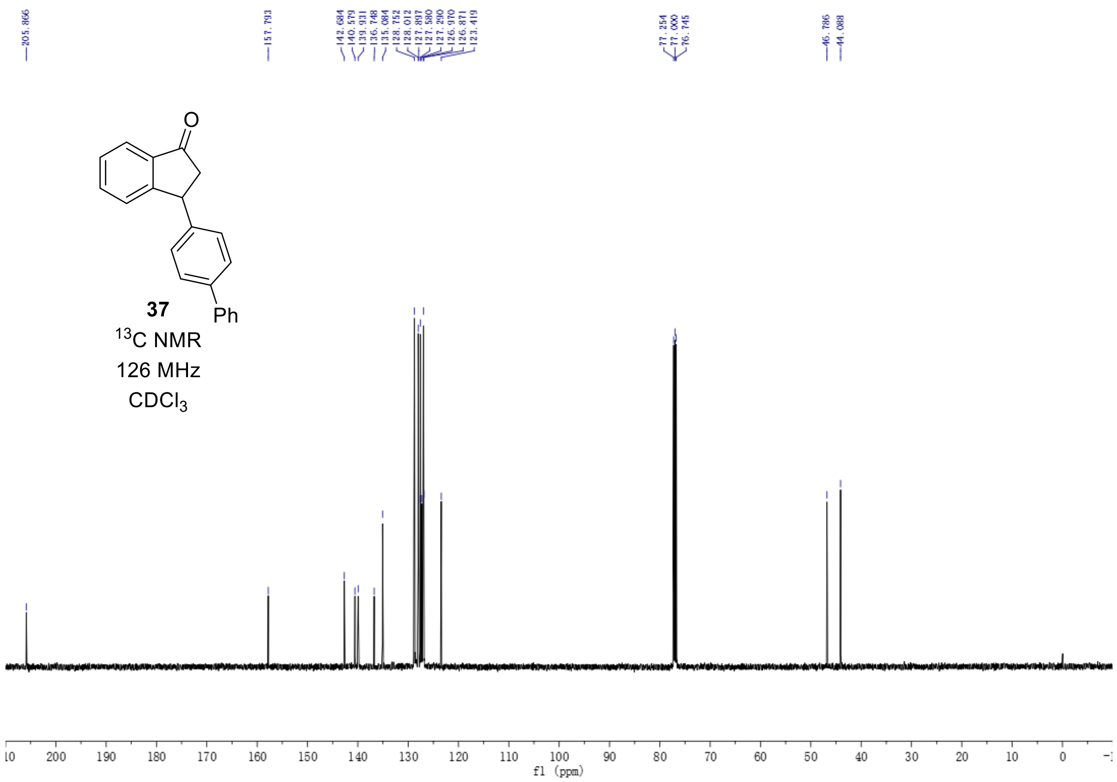

Supplementary Fig. 88 ^{13}C NMR spectrum of compound **37**

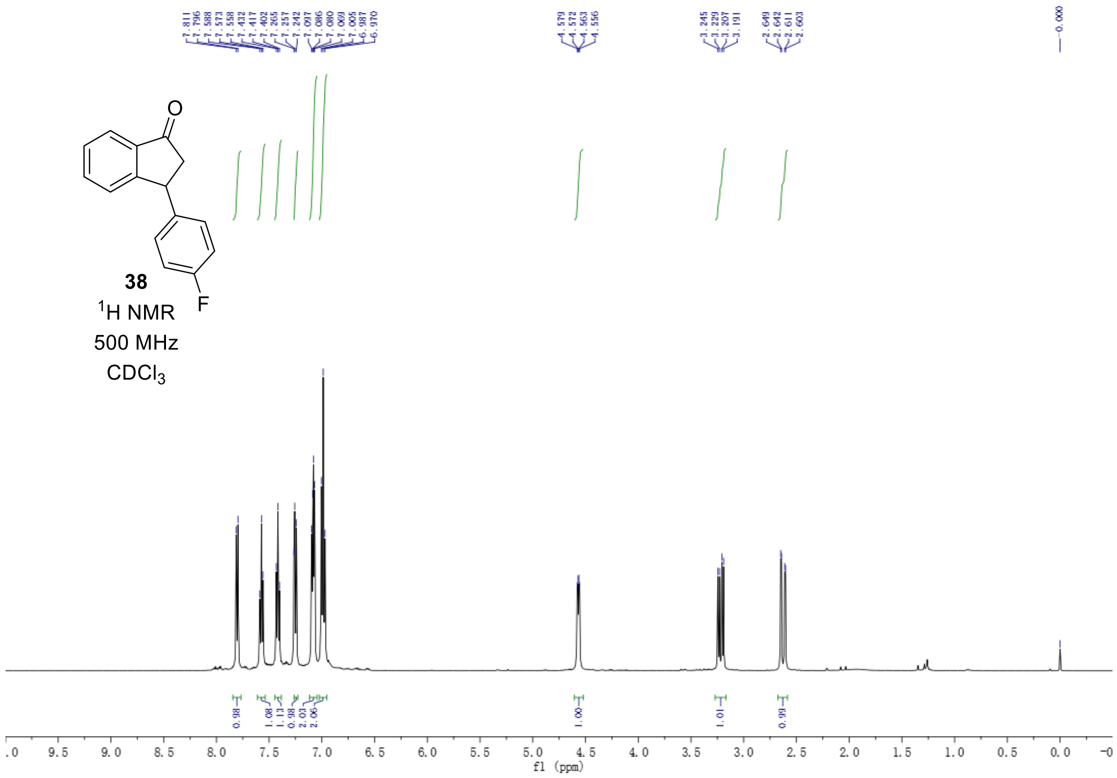

Supplementary Fig. 89 ^{1}H NMR spectrum of compound **38**

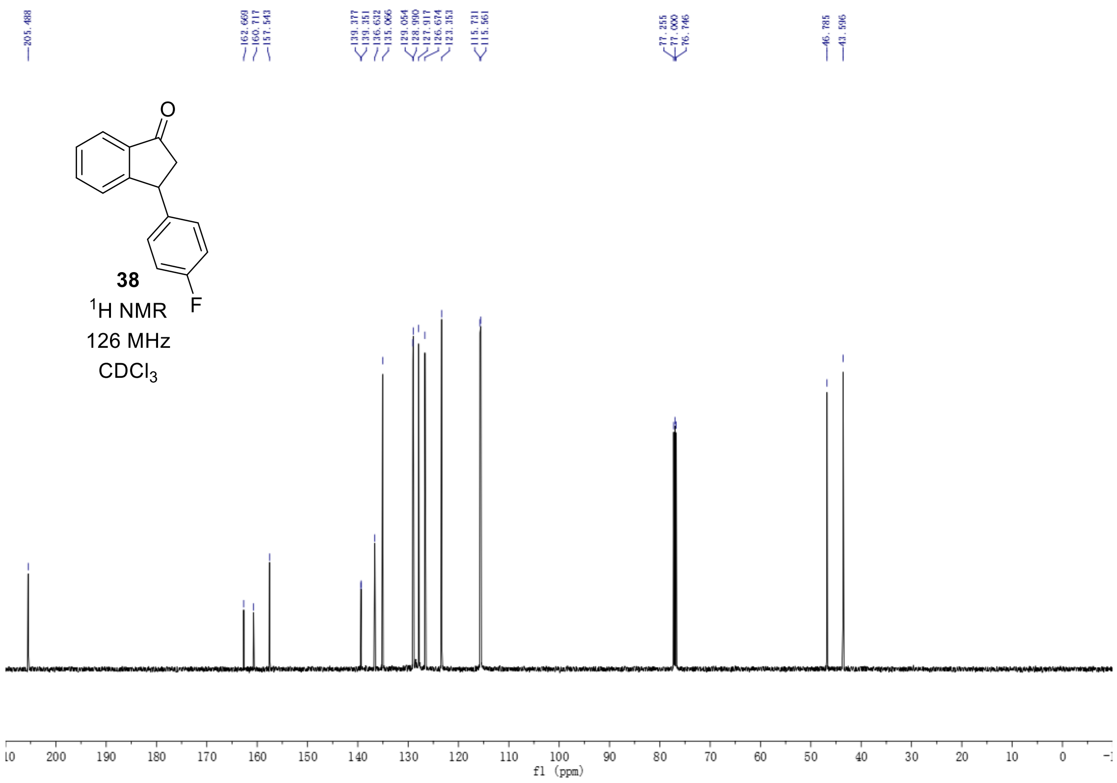

Supplementary Fig. 90 ^{13}C NMR spectrum of compound **38**

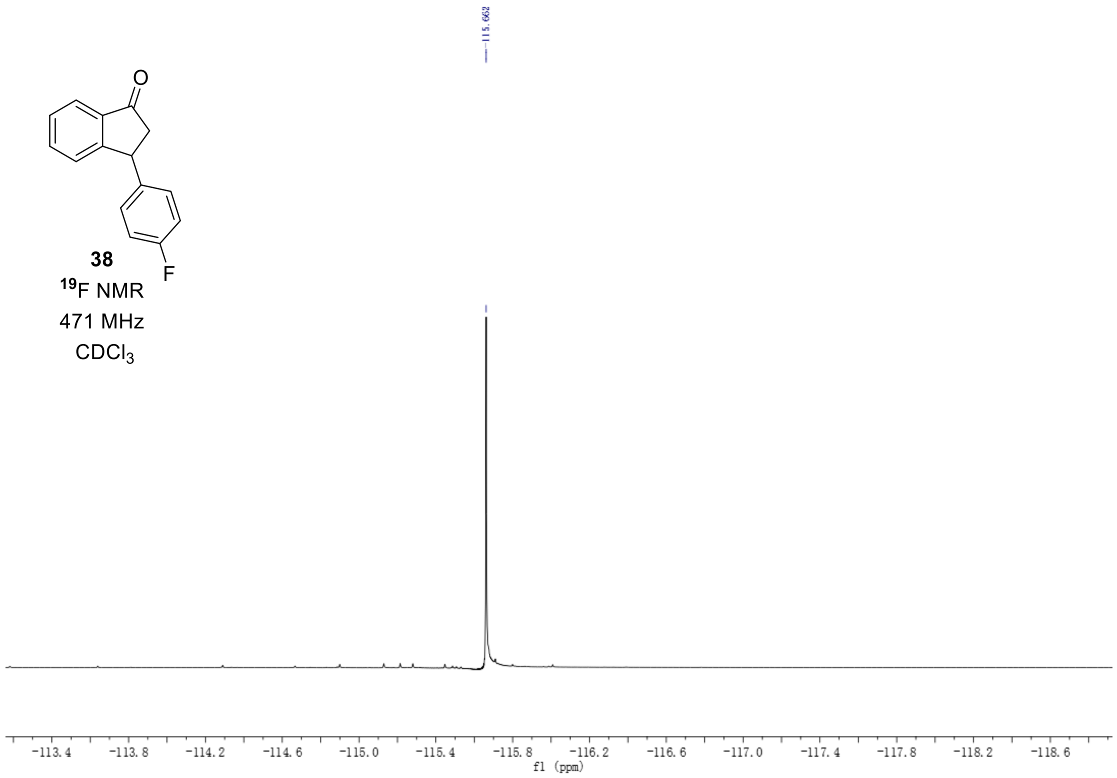

Supplementary Fig. 91 ^{19}F NMR spectrum of compound **38**

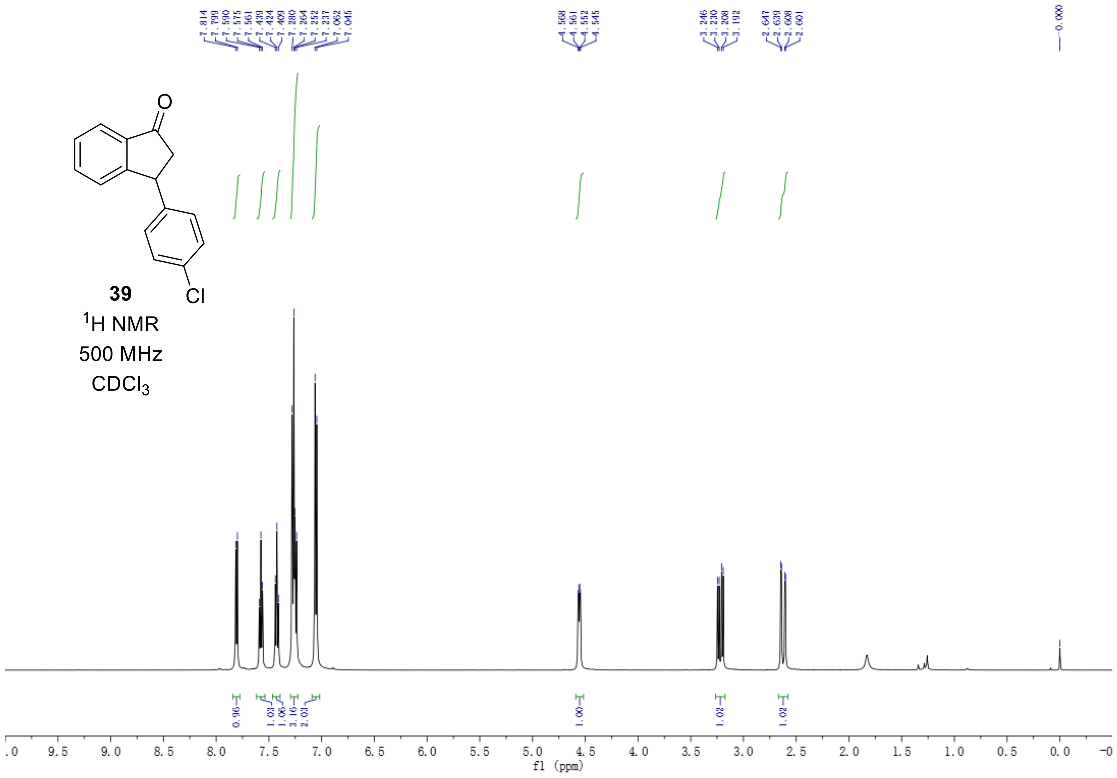

Supplementary Fig. 92 ^{1}H NMR spectrum of compound **39**

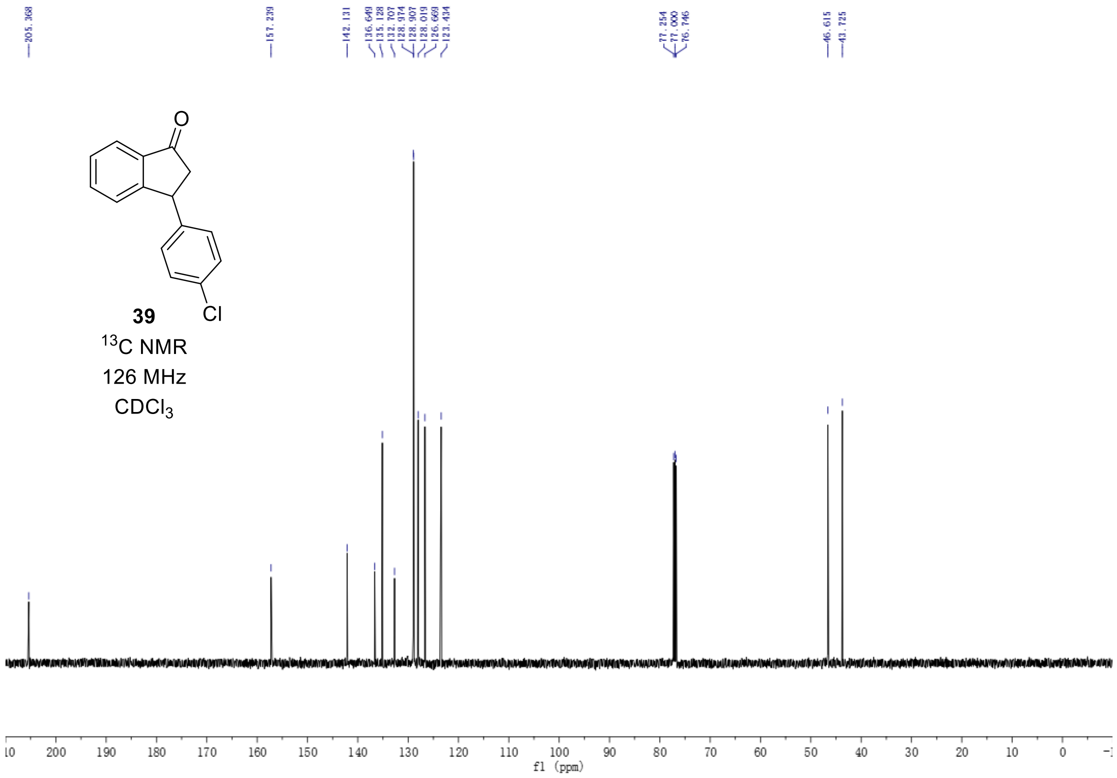

Supplementary Fig. 93 ^{13}C NMR spectrum of compound **39**

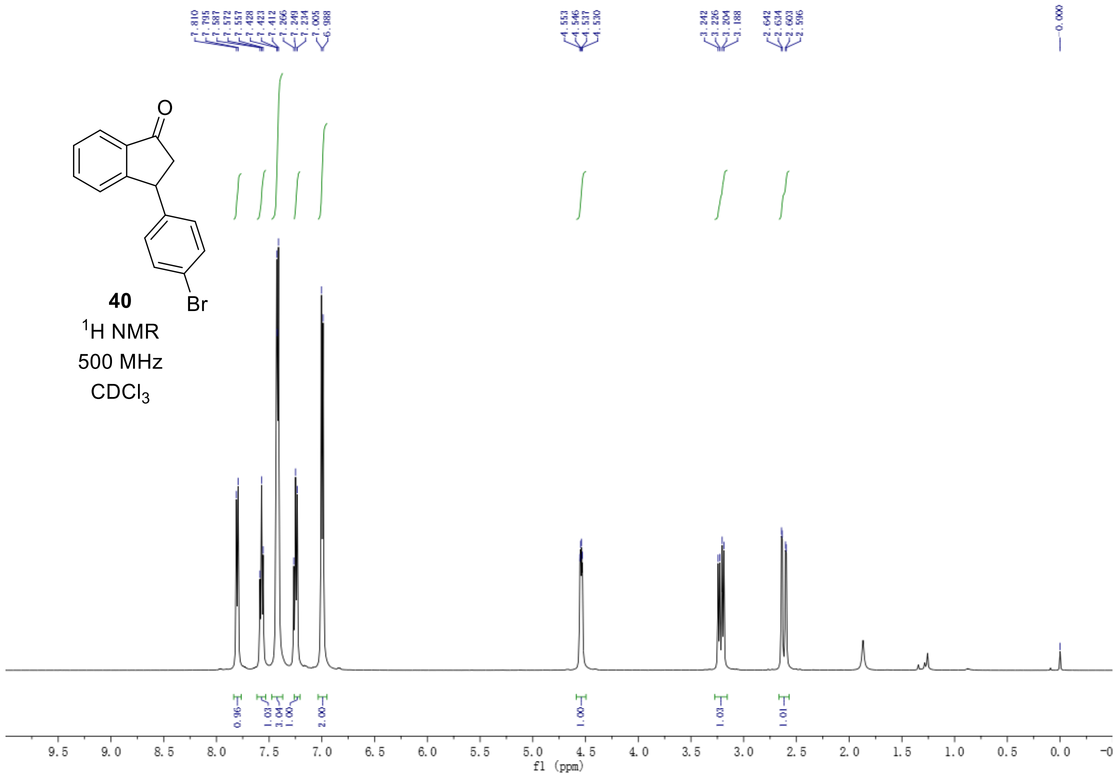

Supplementary Fig. 94 ^{1}H NMR spectrum of compound **40**

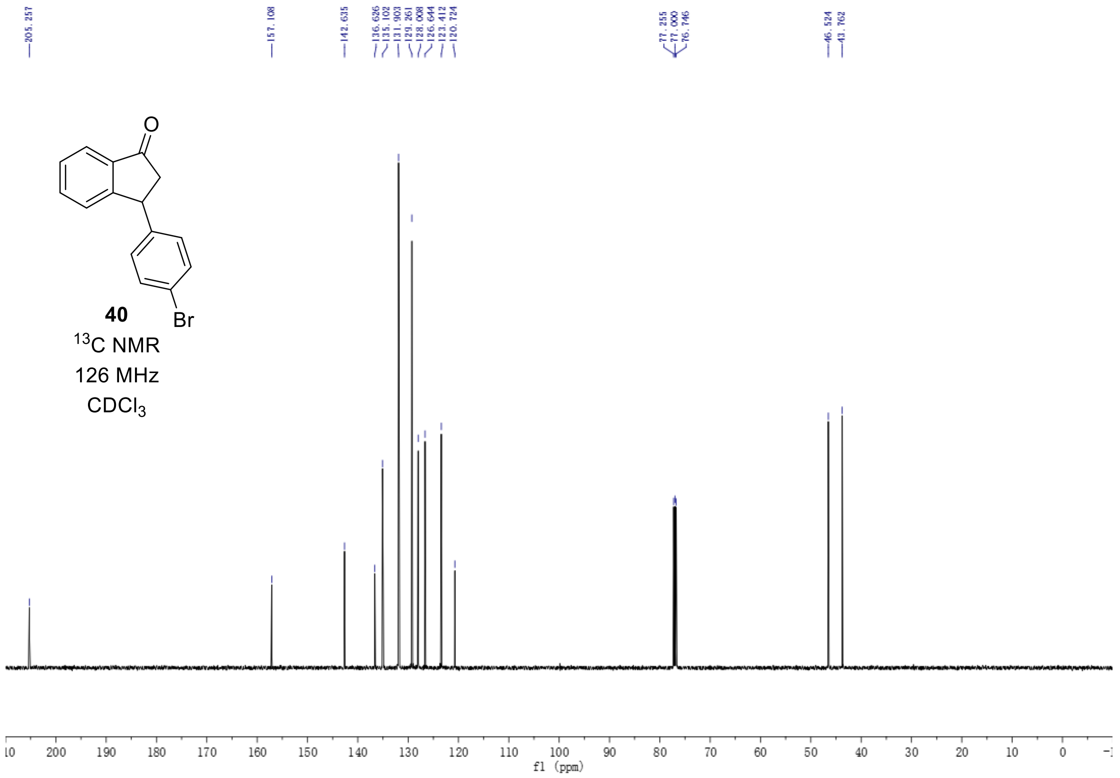

Supplementary Fig. 95 ^{13}C NMR spectrum of compound **40**

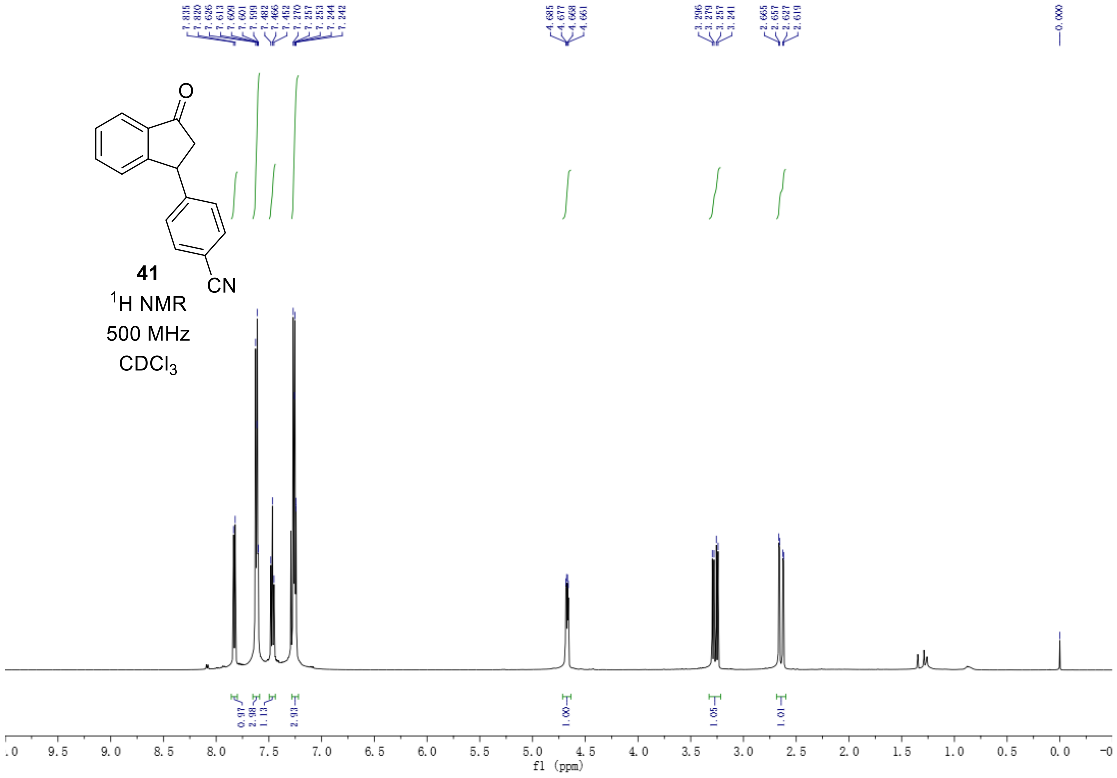

Supplementary Fig. 96 ^{1}H NMR spectrum of compound **41**

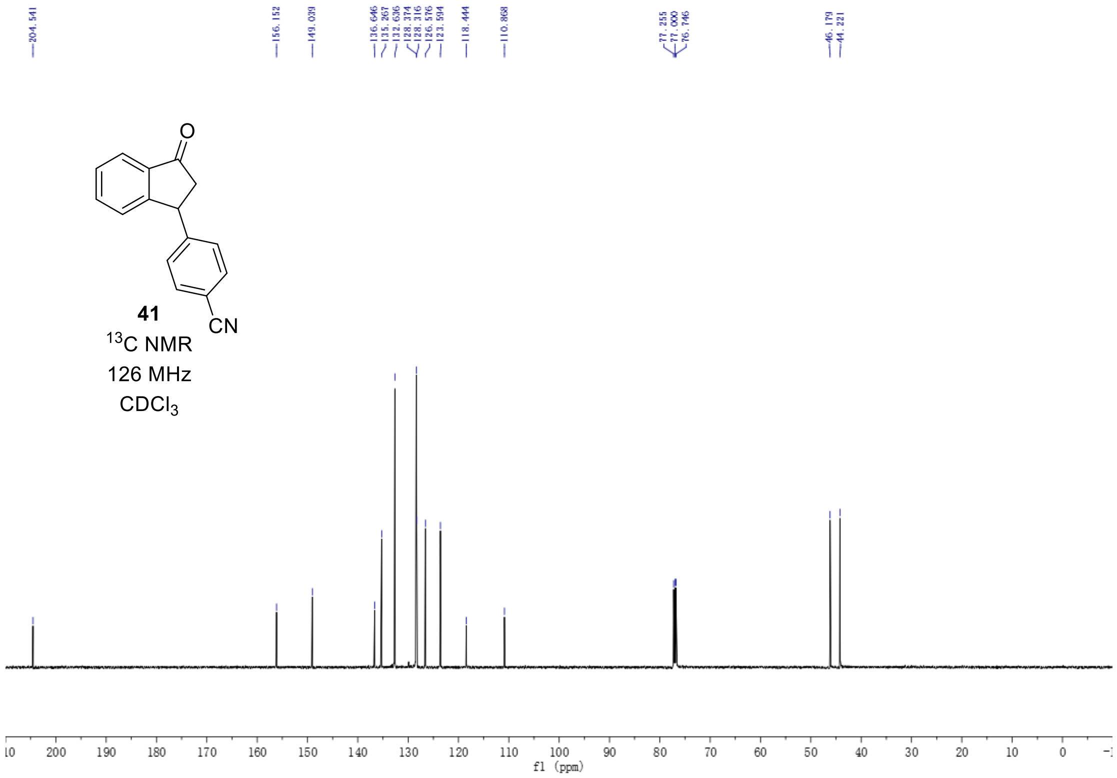

Supplementary Fig. 97 ^{13}C NMR spectrum of compound **41**

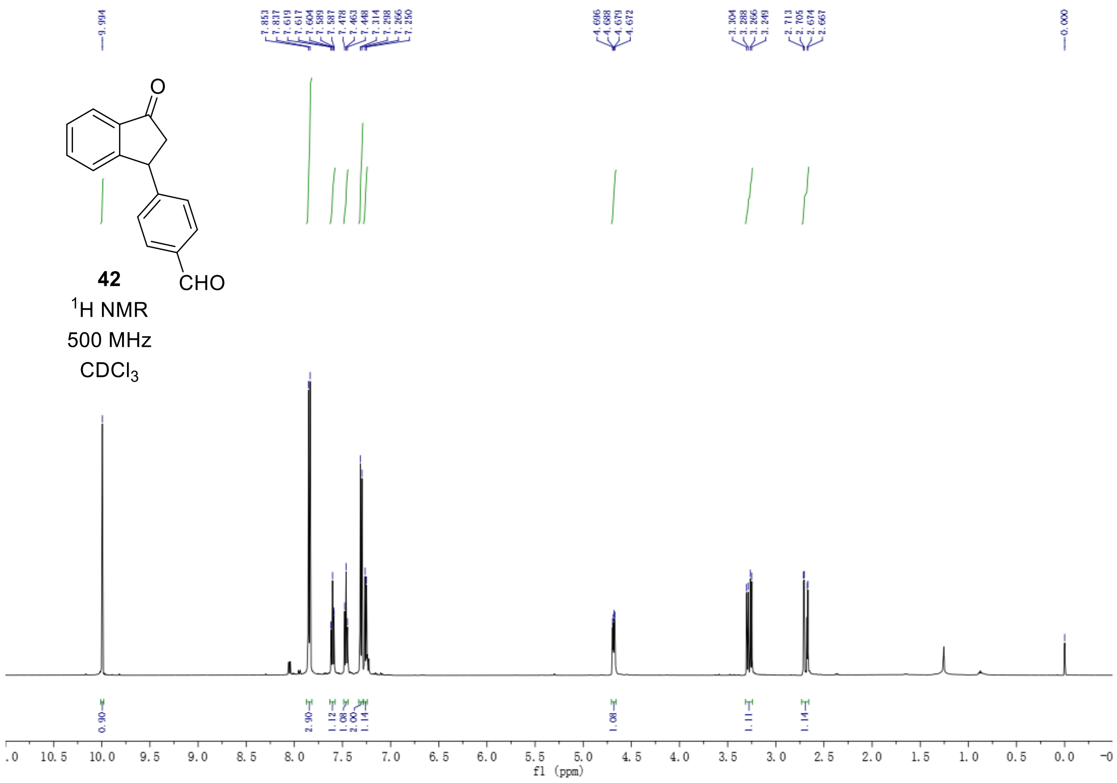

Supplementary Fig. 98 ^{1}H NMR spectrum of compound **42**

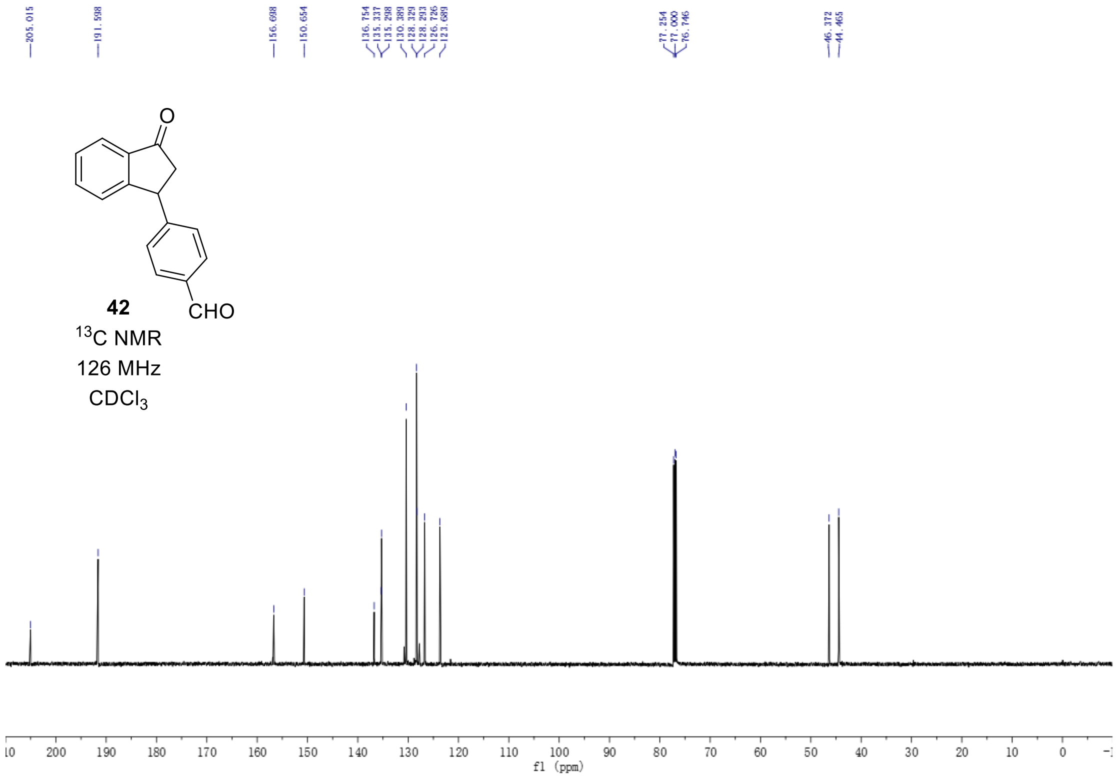

Supplementary Fig. 99 ^{13}C NMR spectrum of compound **42**

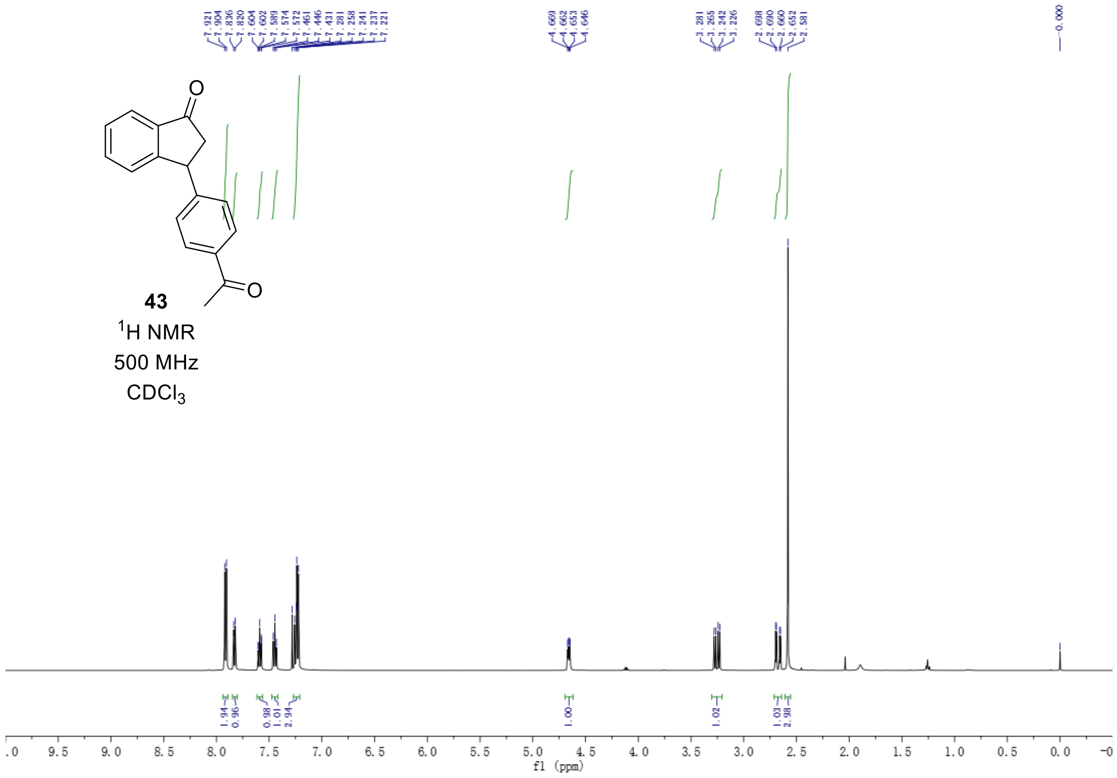

Supplementary Fig. 100 ^{1}H NMR spectrum of compound **43**

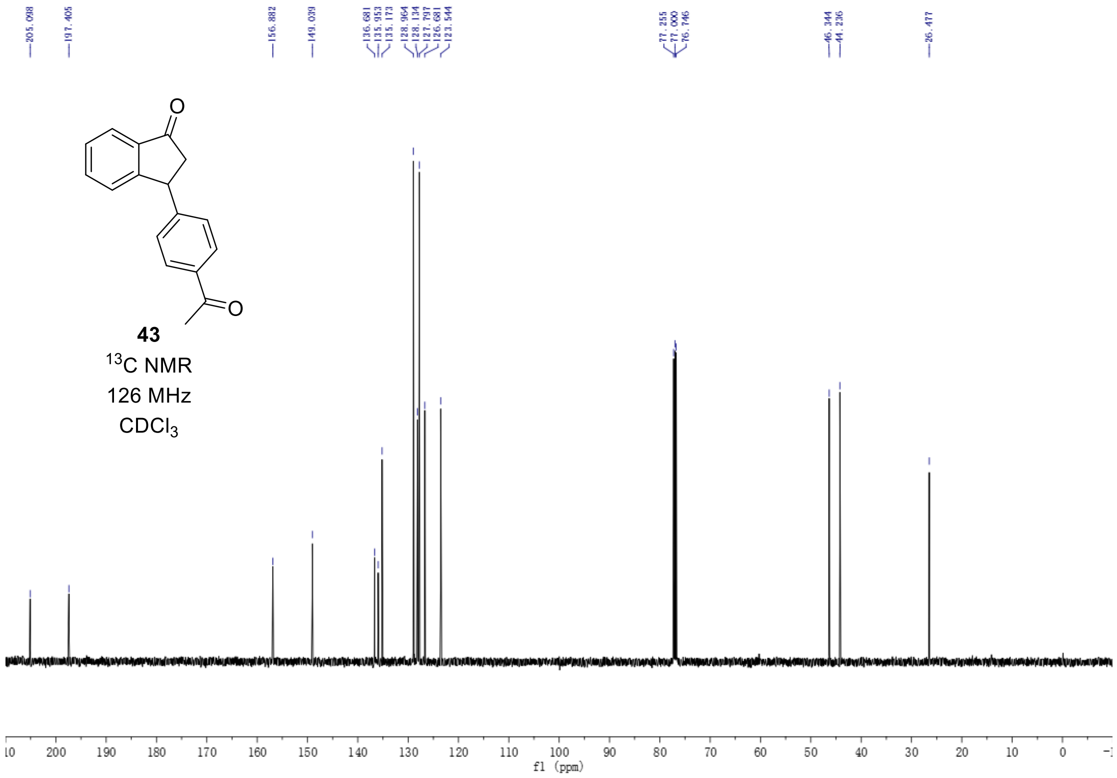

Supplementary Fig. 101 ^{13}C NMR spectrum of compound **43**

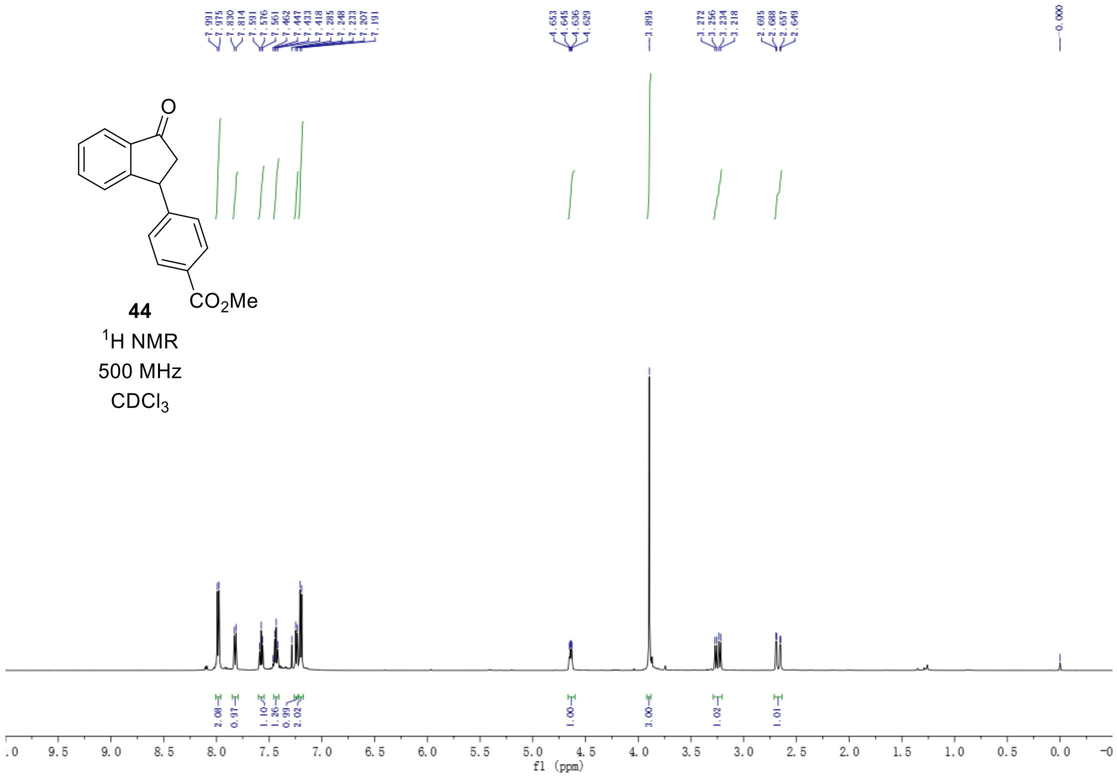

Supplementary Fig. 102 ^{1}H NMR spectrum of compound **44**

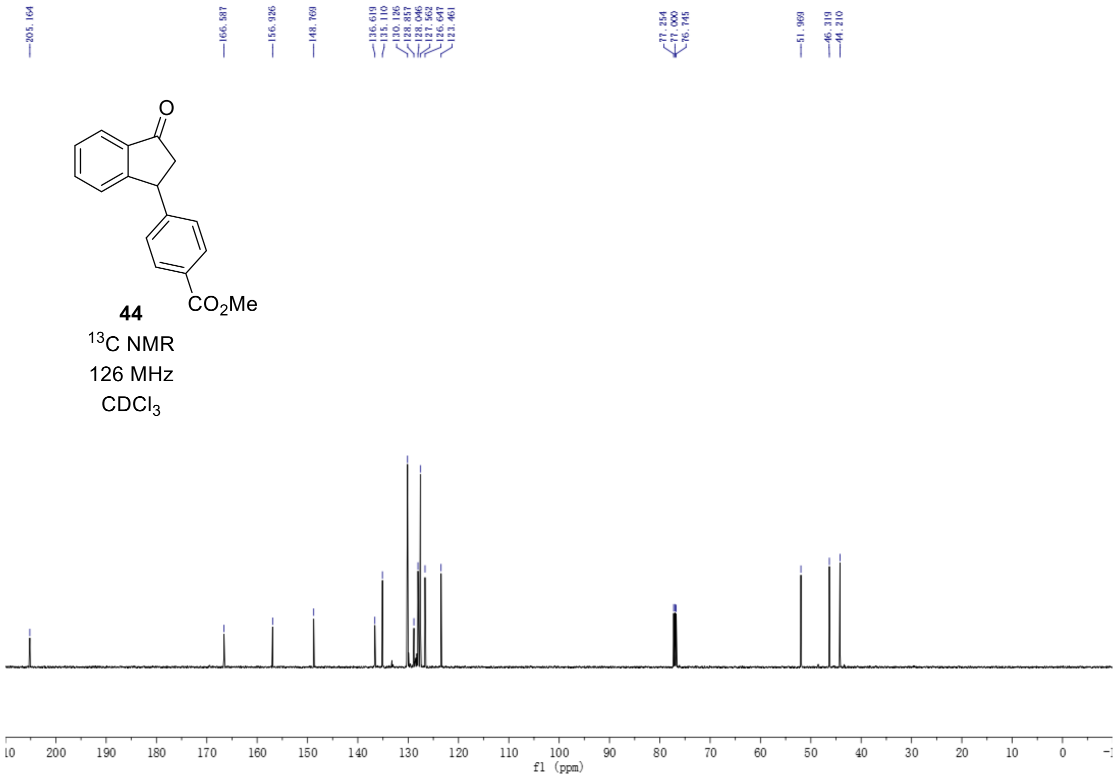

Supplementary Fig. 103 ^{13}C NMR spectrum of compound **44**

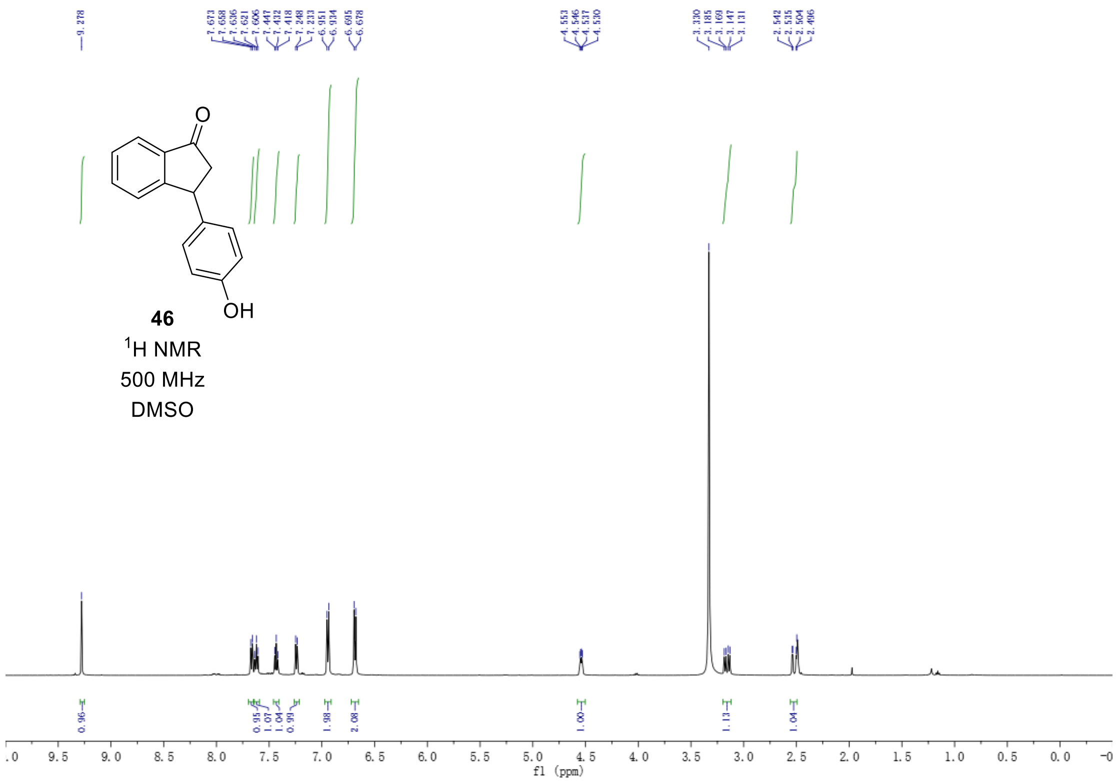

Supplementary Fig.104 ^{1}H NMR spectrum of compound **46**

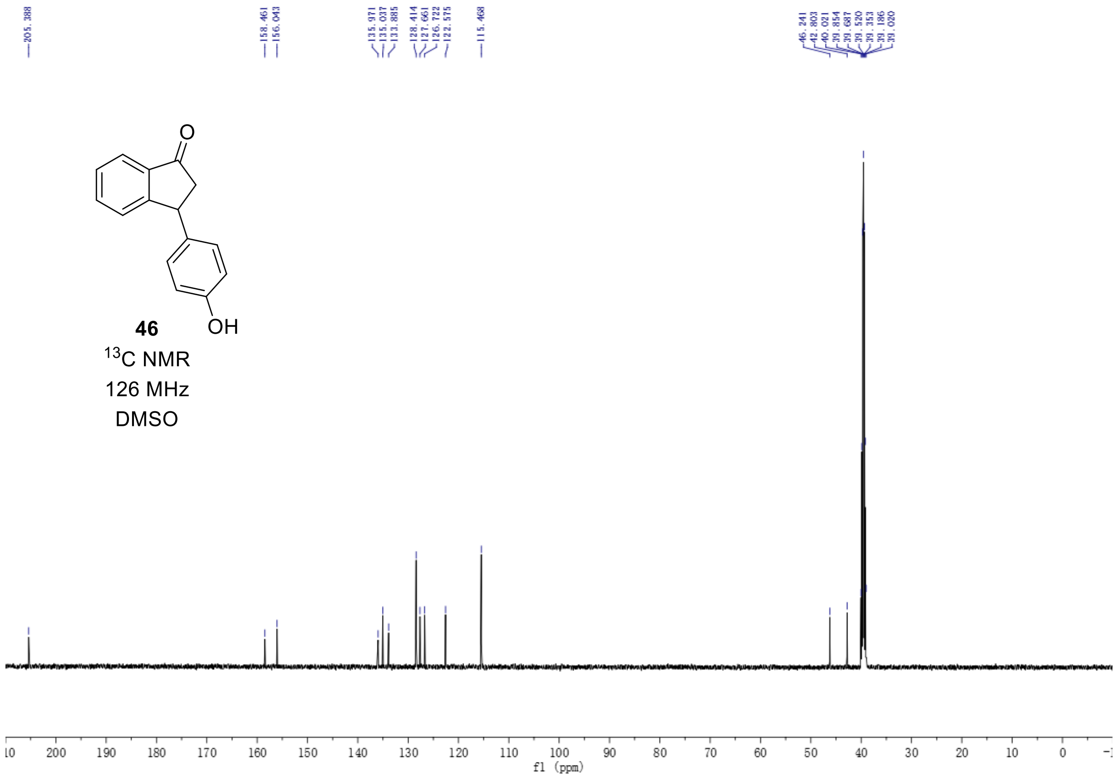

Supplementary Fig. 105 ^{13}C NMR spectrum of compound **46**

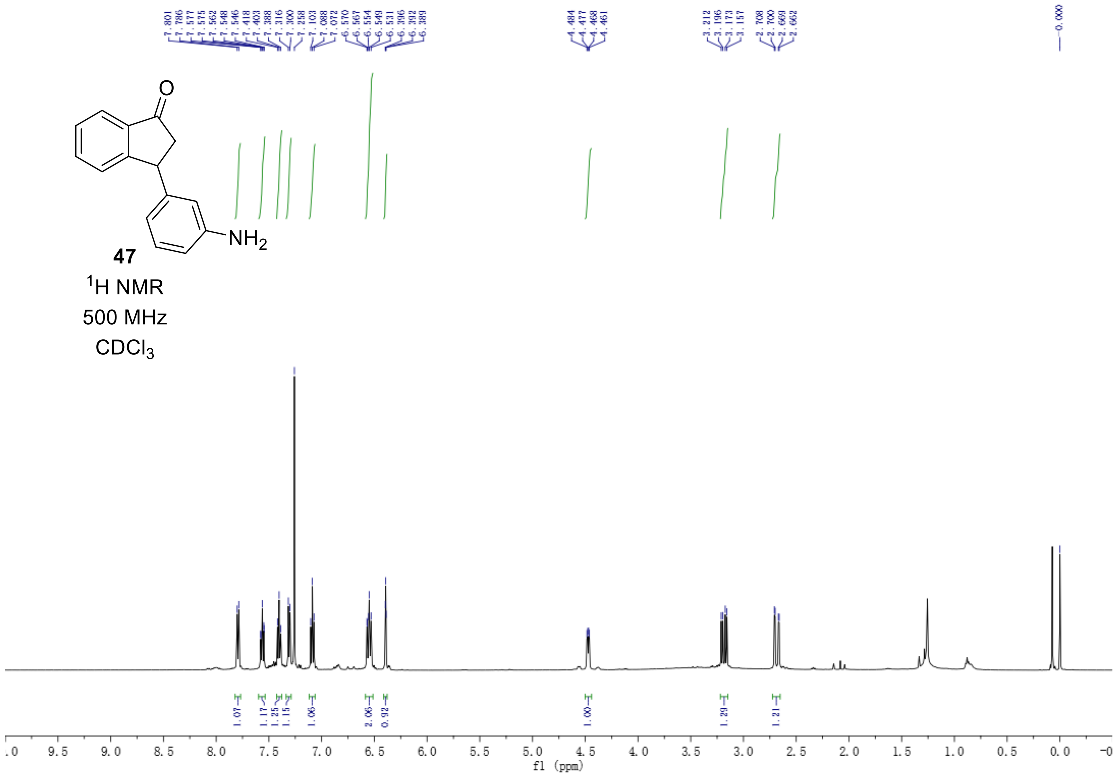

Supplementary Fig. 106 ^{1}H NMR spectrum of compound **47**

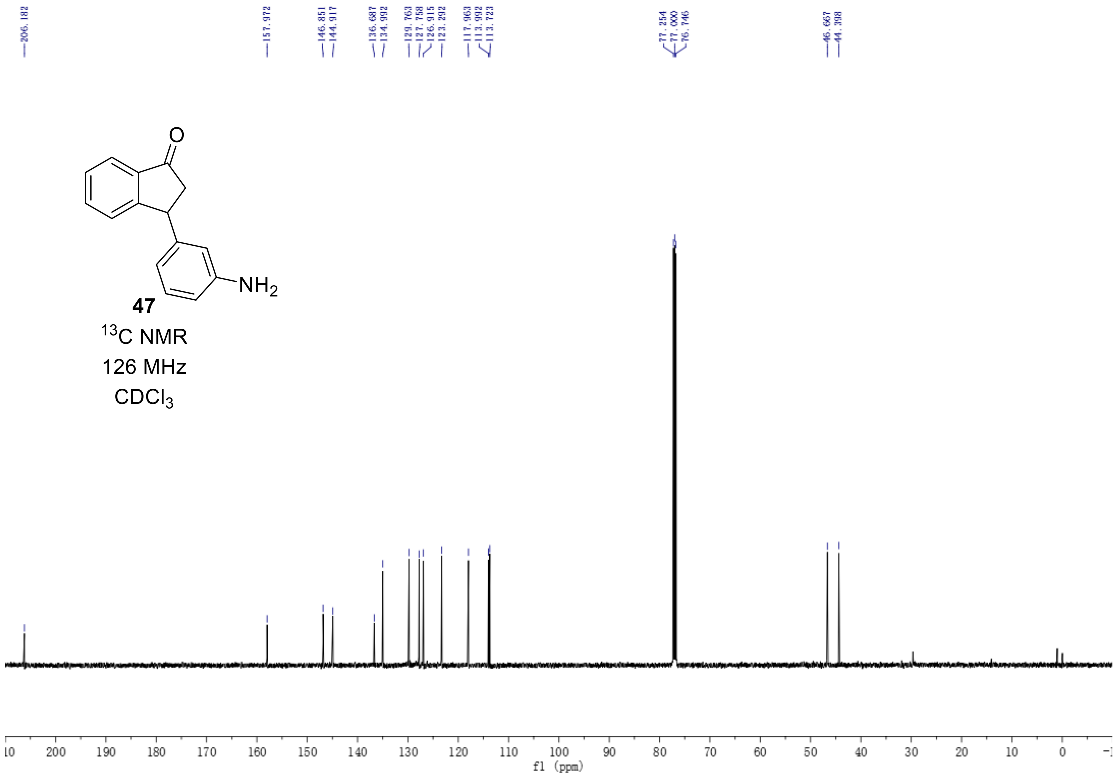

Supplementary Fig. 107 ^{13}C NMR spectrum of compound **47**

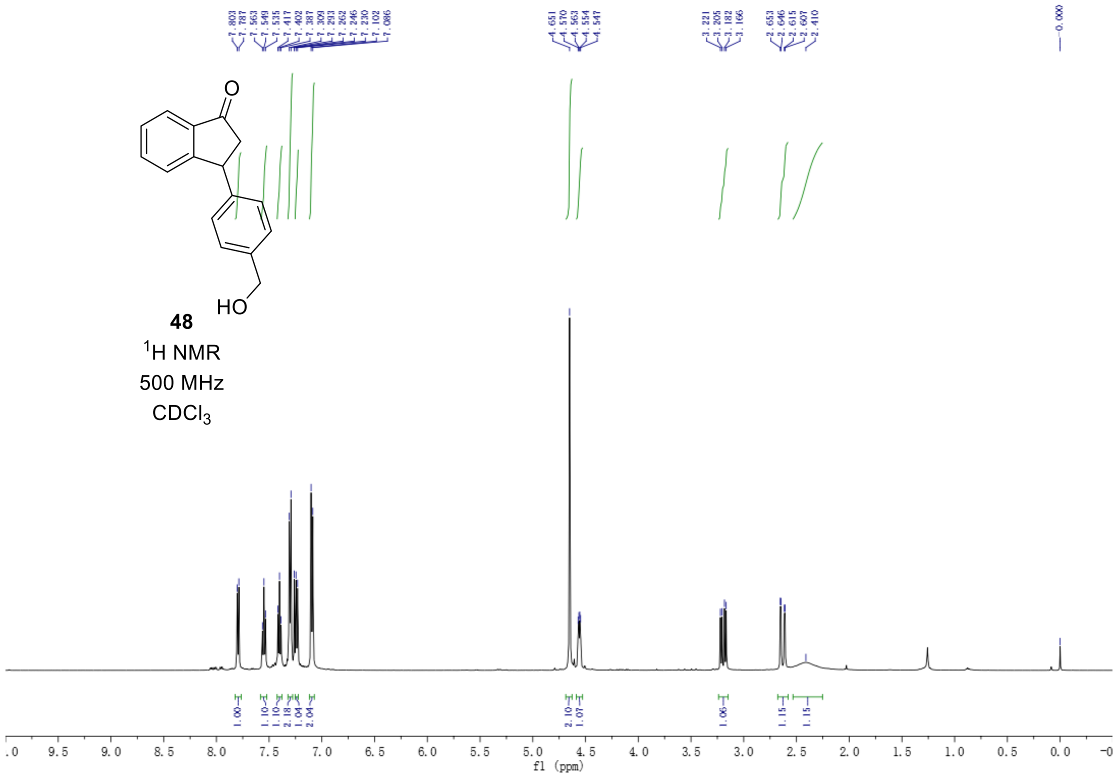

Supplementary Fig. 108 ^{1}H NMR spectrum of compound **48**

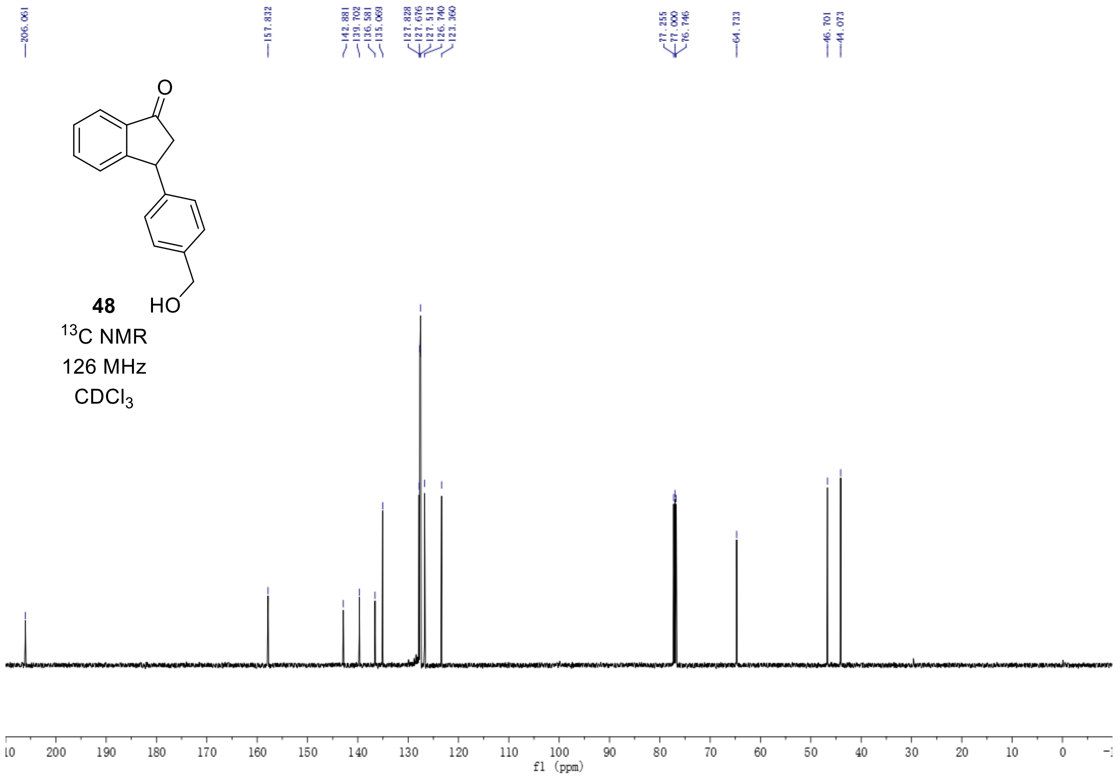

Supplementary Fig. 109 ^{13}C NMR spectrum of compound **48**

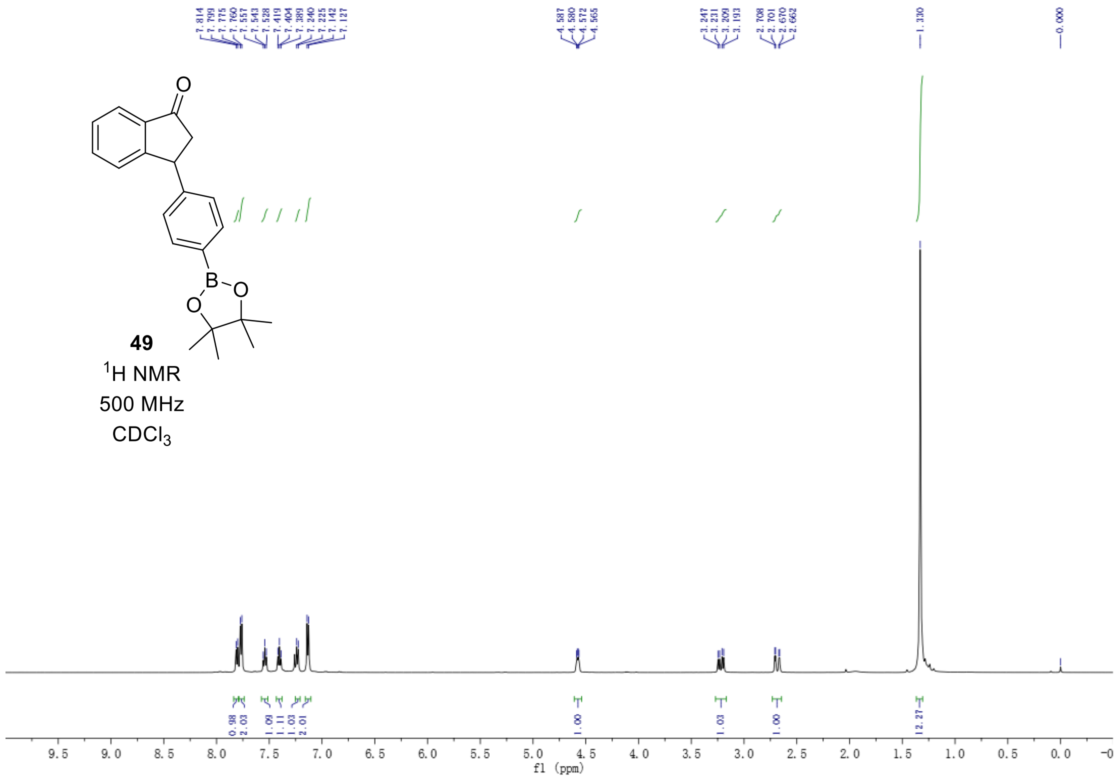

Supplementary Fig. 110 ^{1}H NMR spectrum of compound **49**

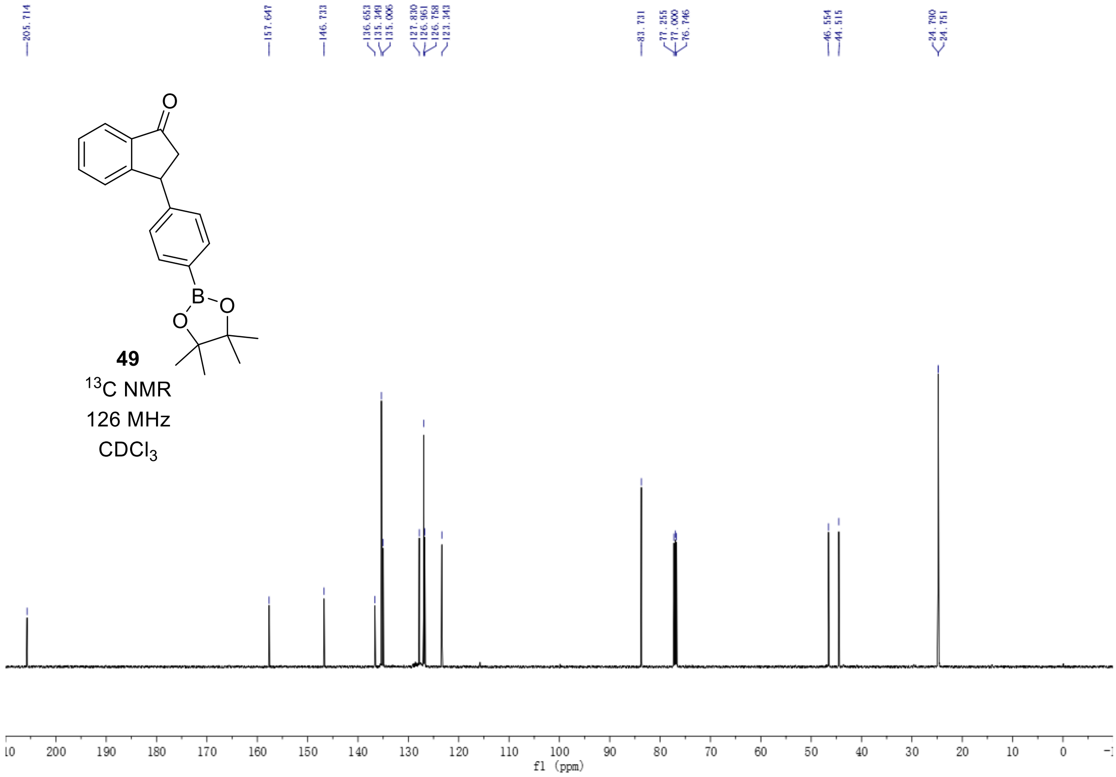

Supplementary Fig. 111 ^{13}C NMR spectrum of compound **49**

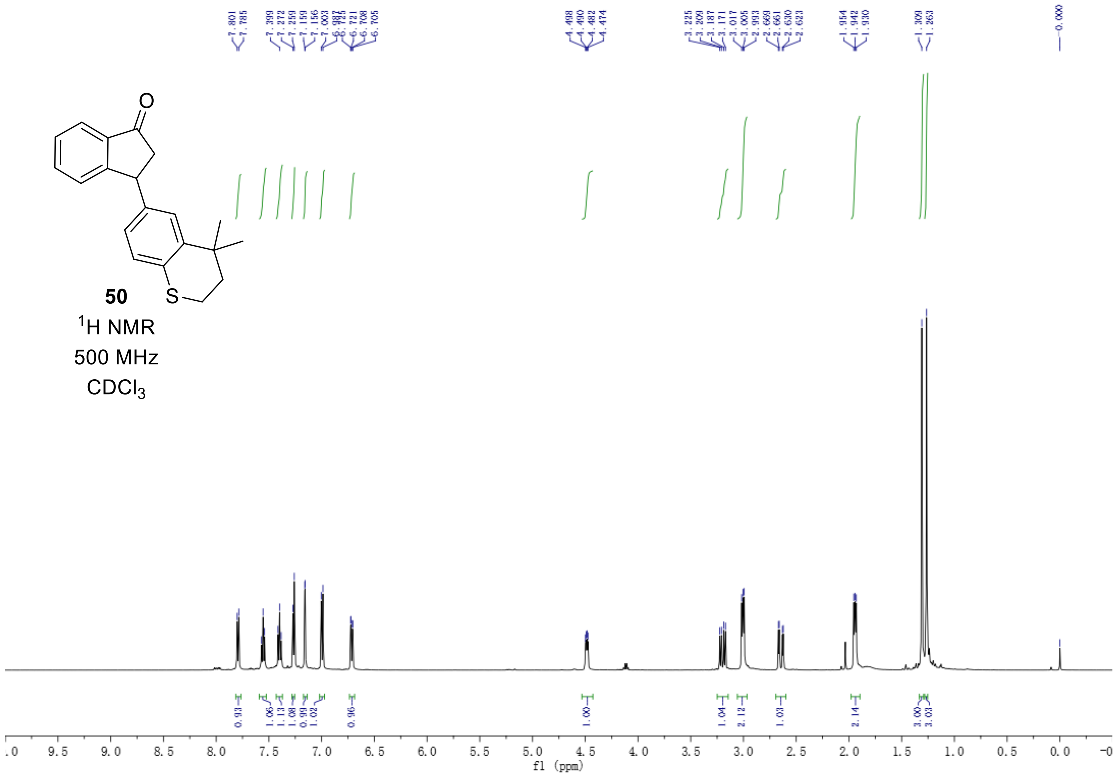

Supplementary Fig. 112 ^{1}H NMR spectrum of compound **50**

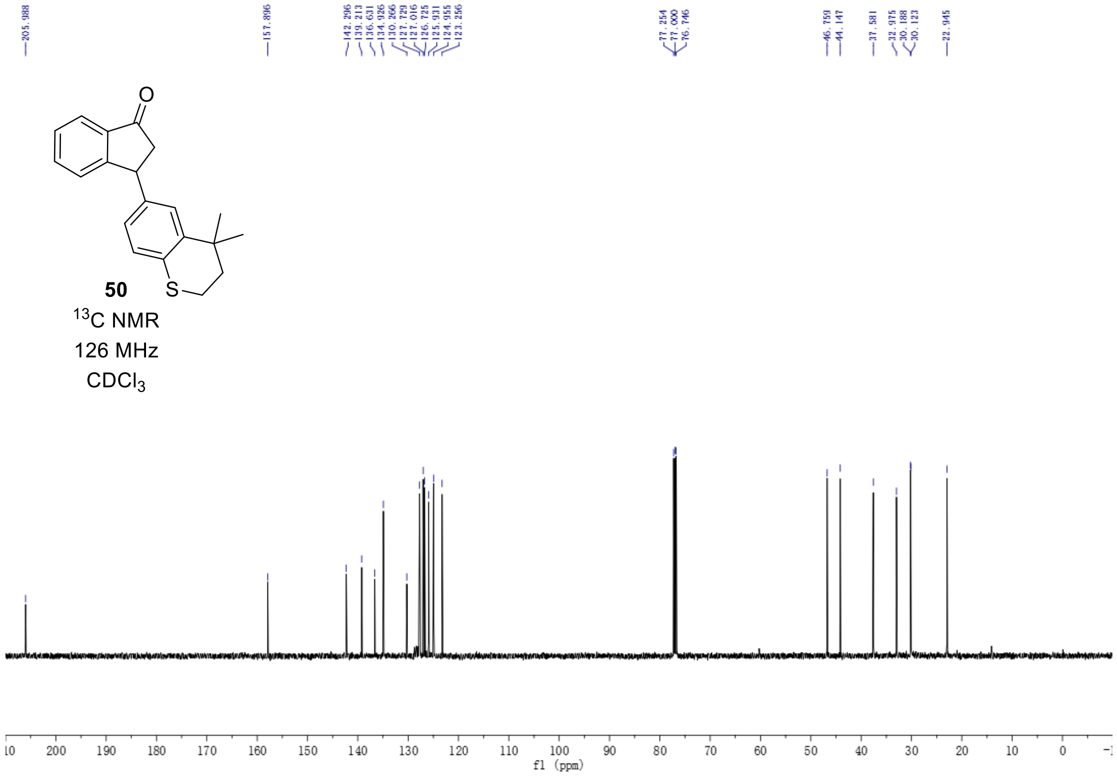

Supplementary Fig. 113 ^{13}C NMR spectrum of compound **50**

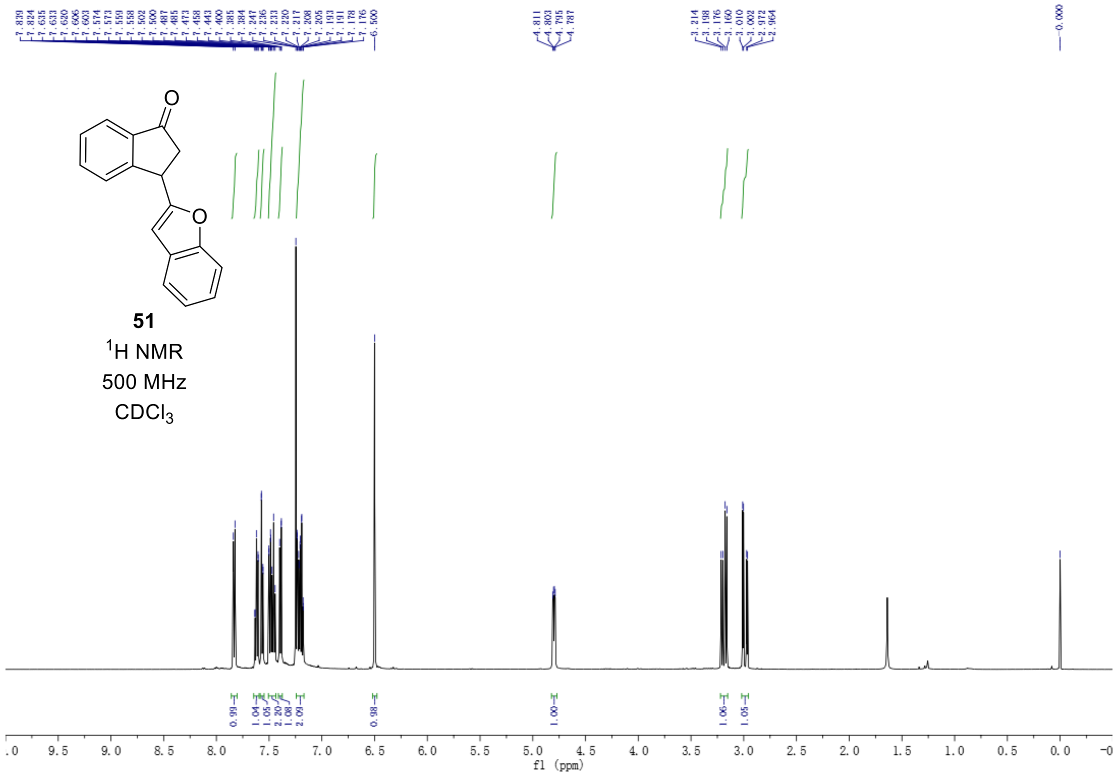

Supplementary Fig. 114 ^{1}H NMR spectrum of compound **51**

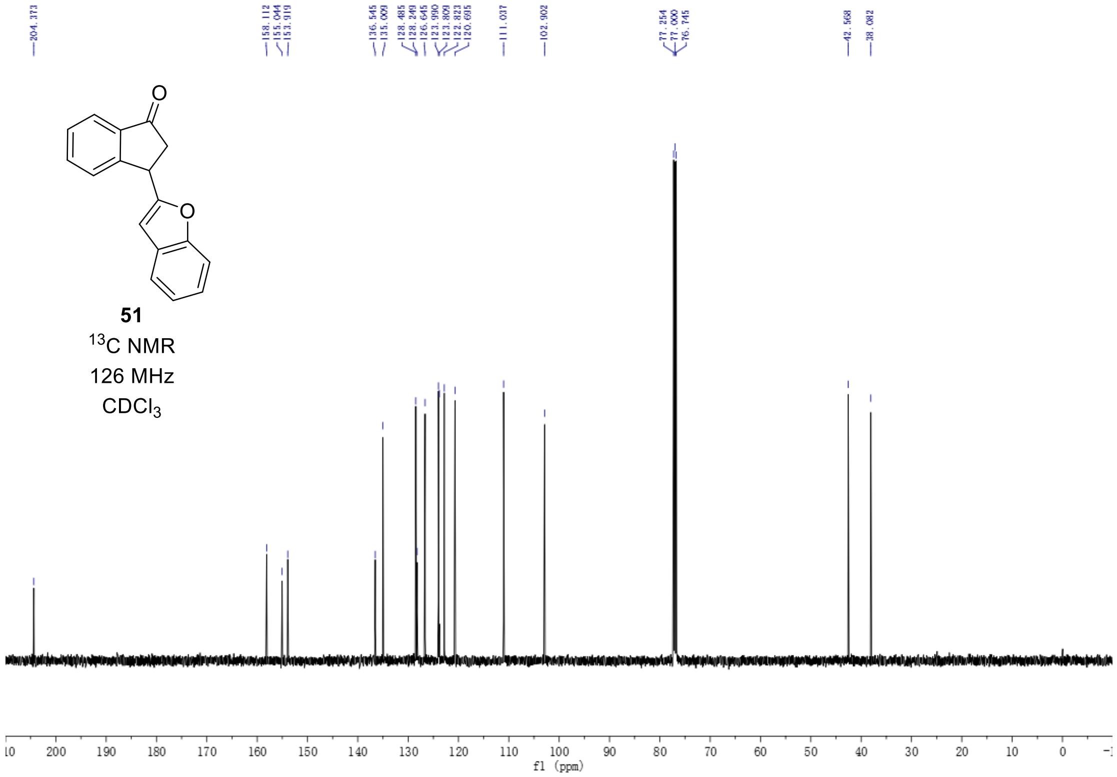

Supplementary Fig. 115 ^{13}C NMR spectrum of compound **51**

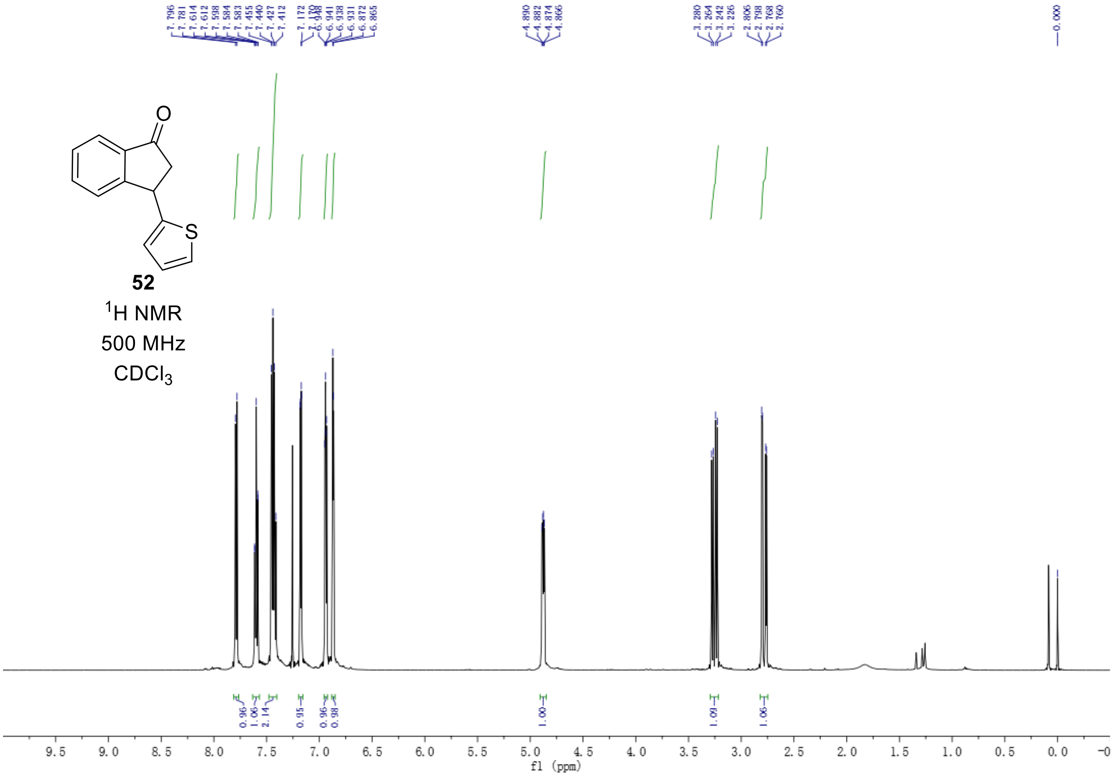

Supplementary Fig.116 ^{1}H NMR spectrum of compound **52**

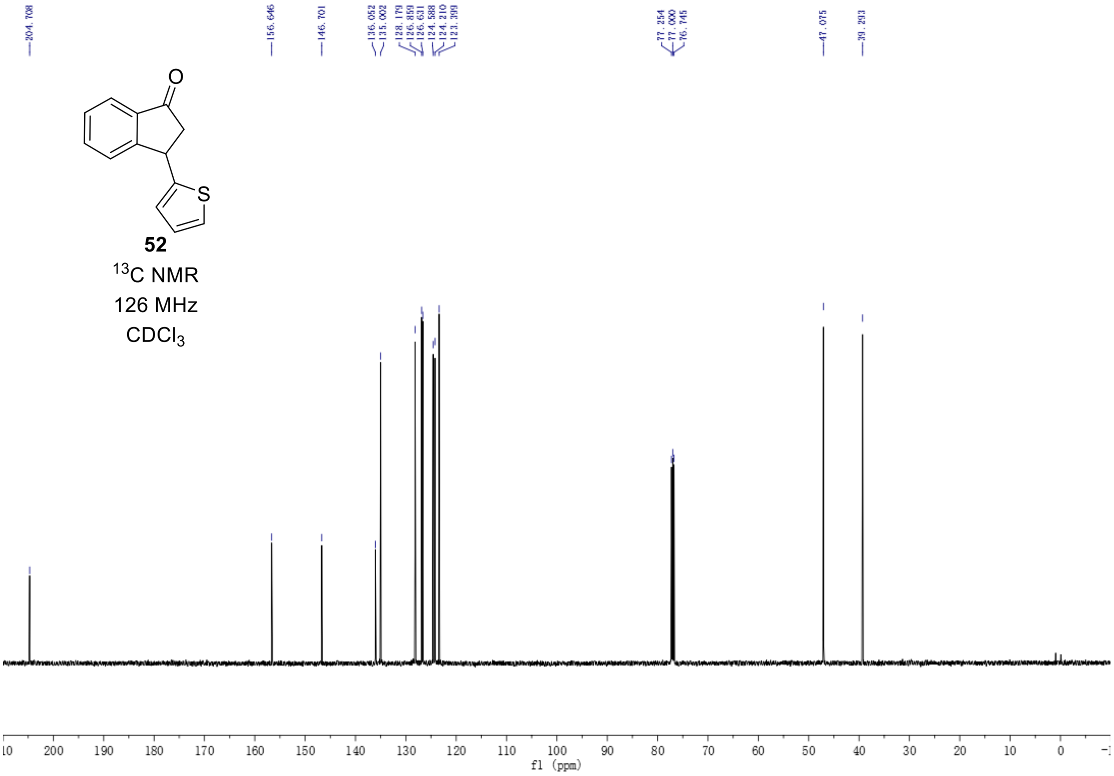

Supplementary Fig. 117 ^{13}C NMR spectrum of compound **52**

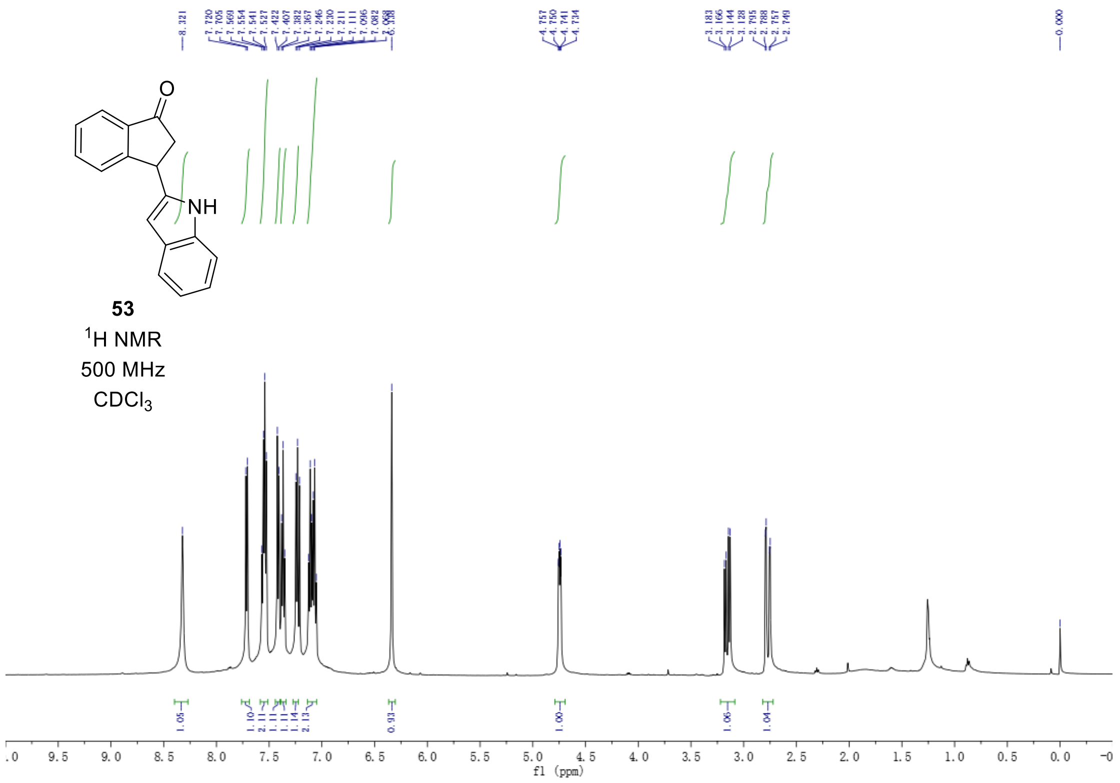

Supplementary Fig. 118 ^{1}H NMR spectrum of compound **53**

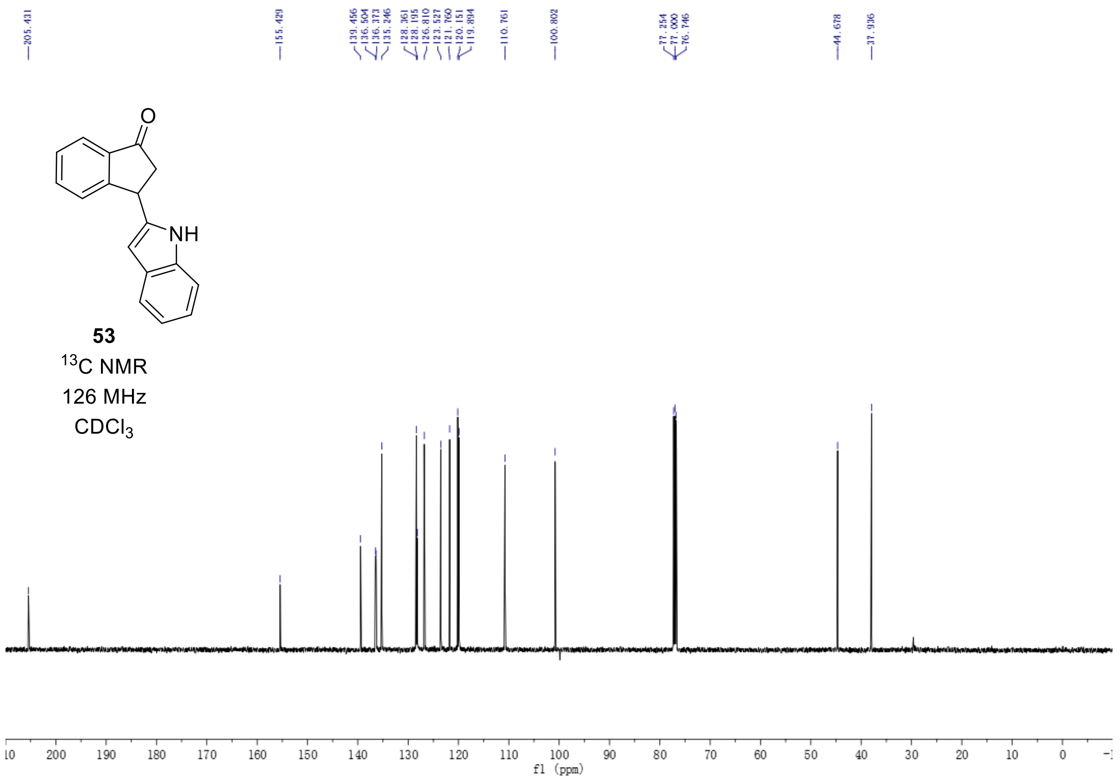

Supplementary Fig. 119 ^{13}C NMR spectrum of compound **53**

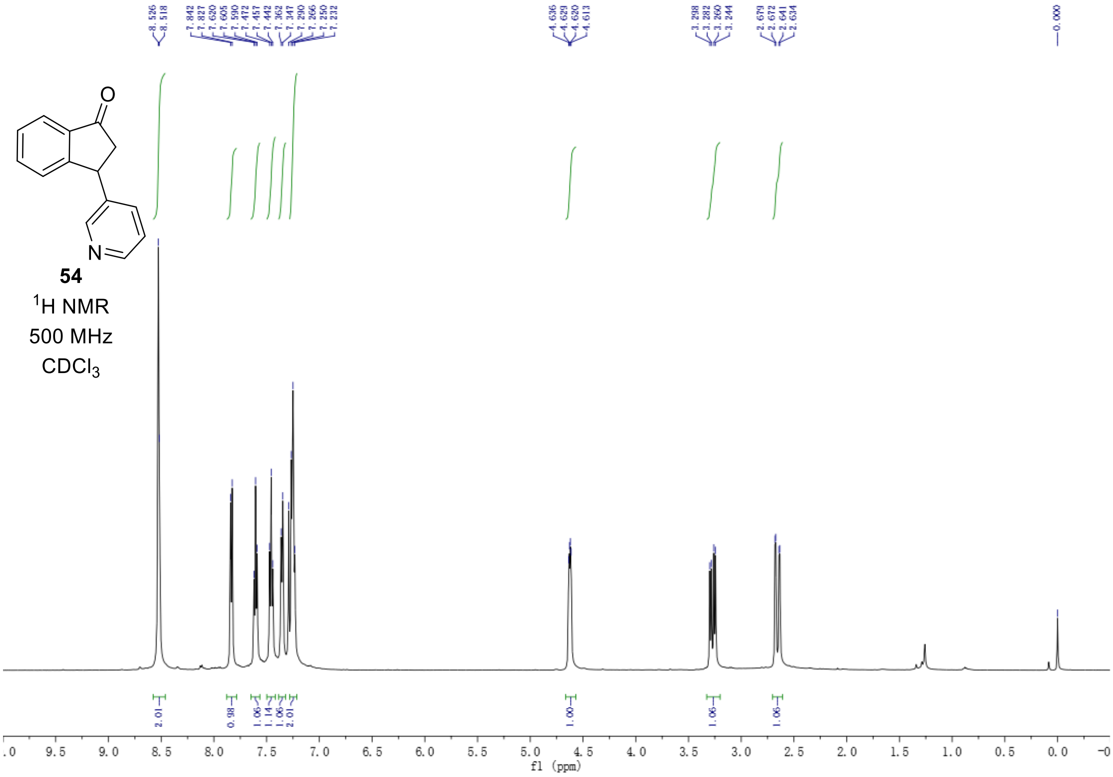

Supplementary Fig. 120 ^{1}H NMR spectrum of compound **54**

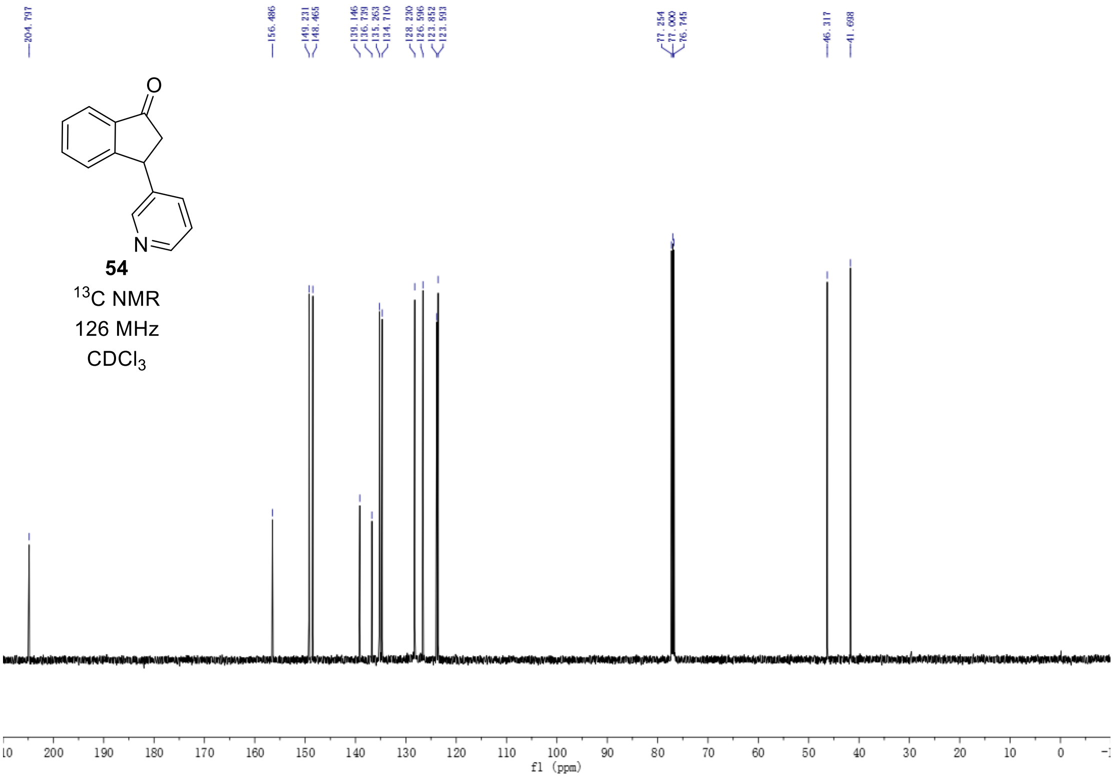

Supplementary Fig. 121 ^{13}C NMR spectrum of compound **54**

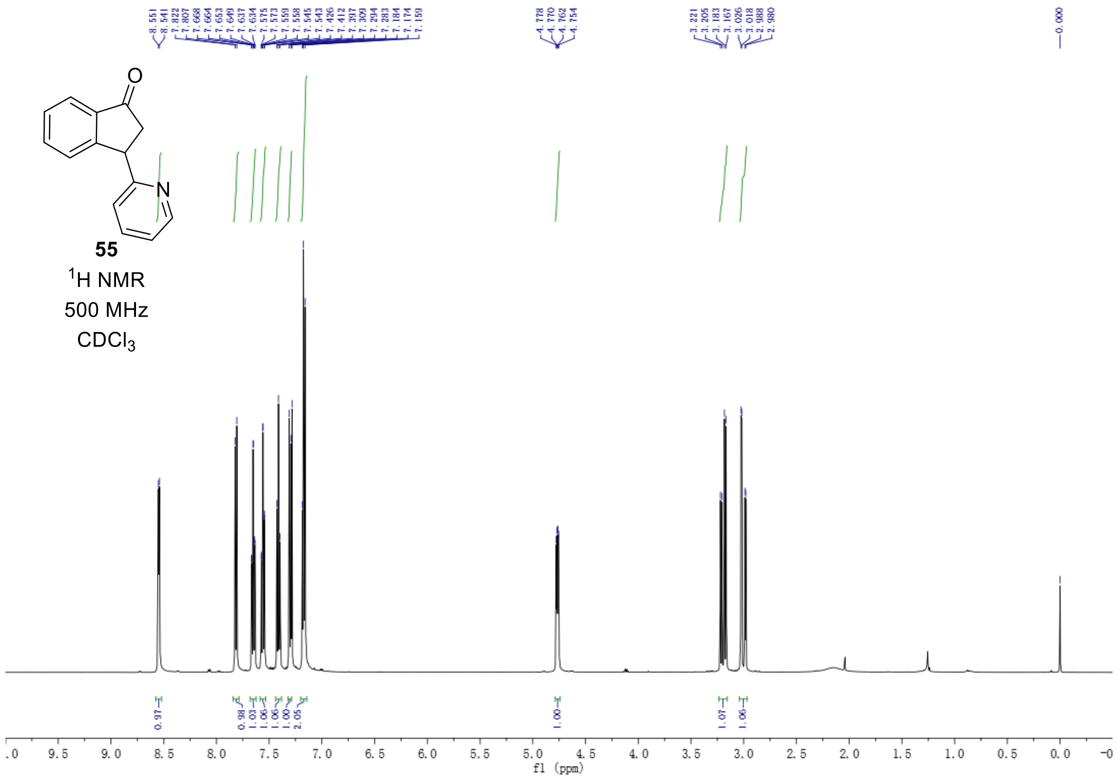

Supplementary Fig. 122 ^{1}H NMR spectrum of compound **55**

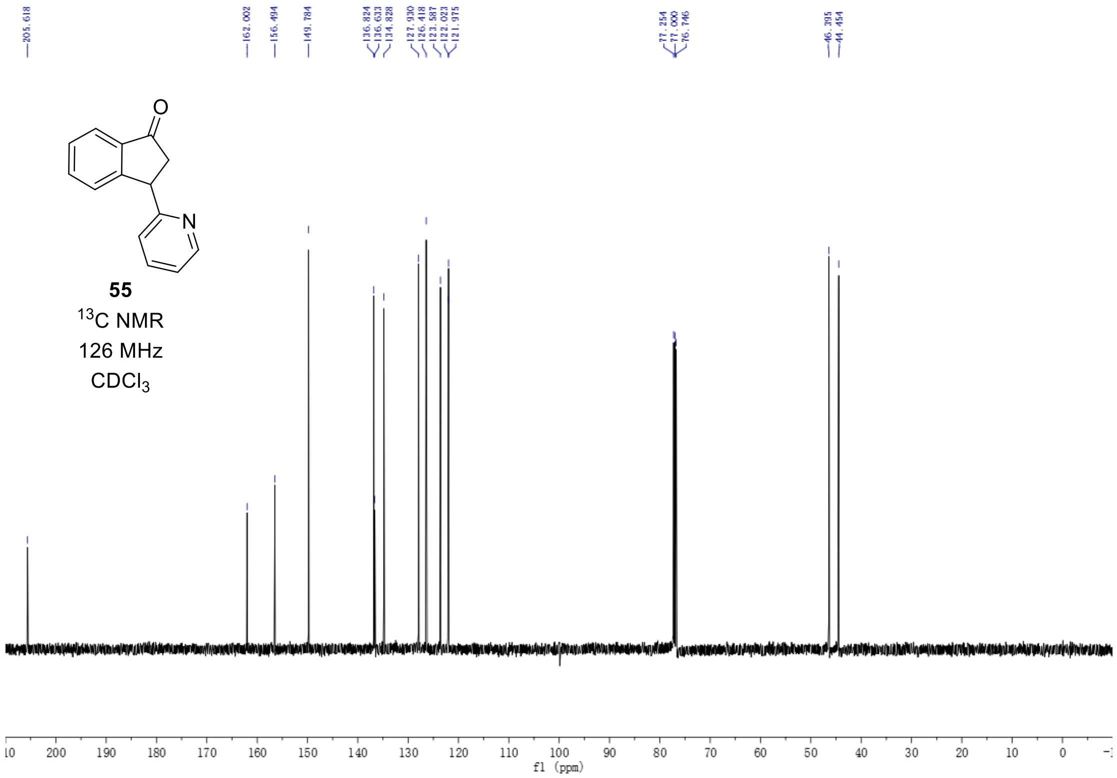

Supplementary Fig. 123 ^{13}C NMR spectrum of compound **55**

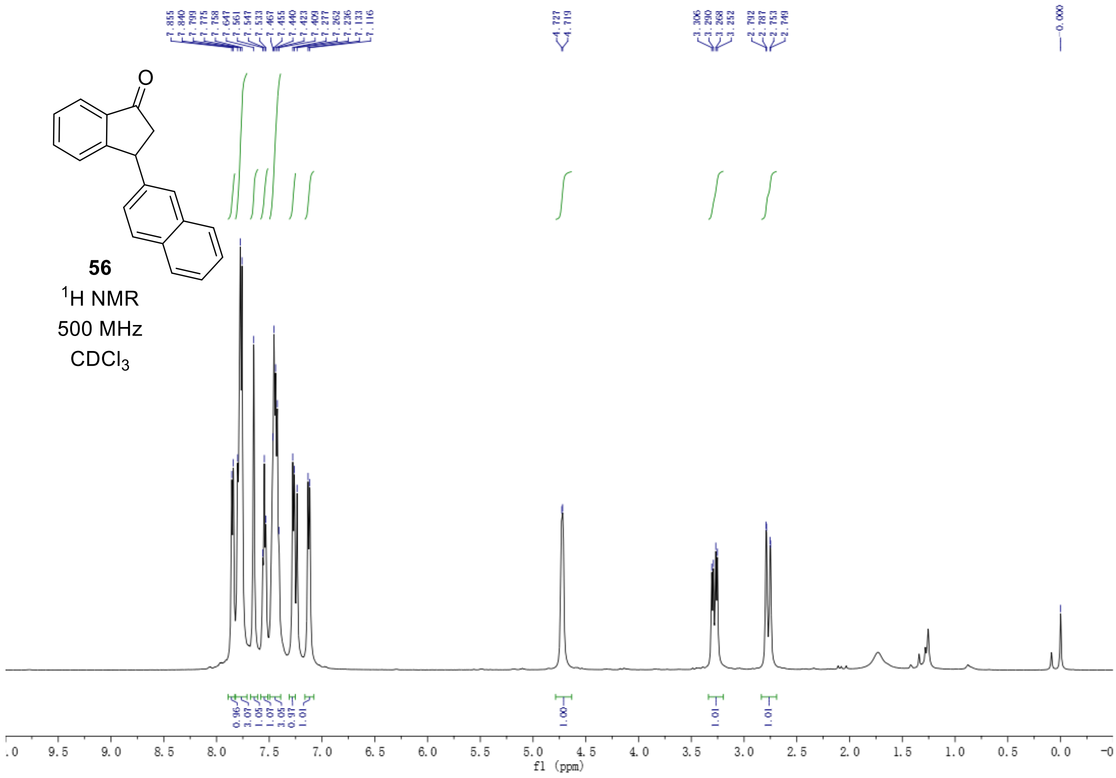

Supplementary Fig. 124 ^{1}H NMR spectrum of compound **56**

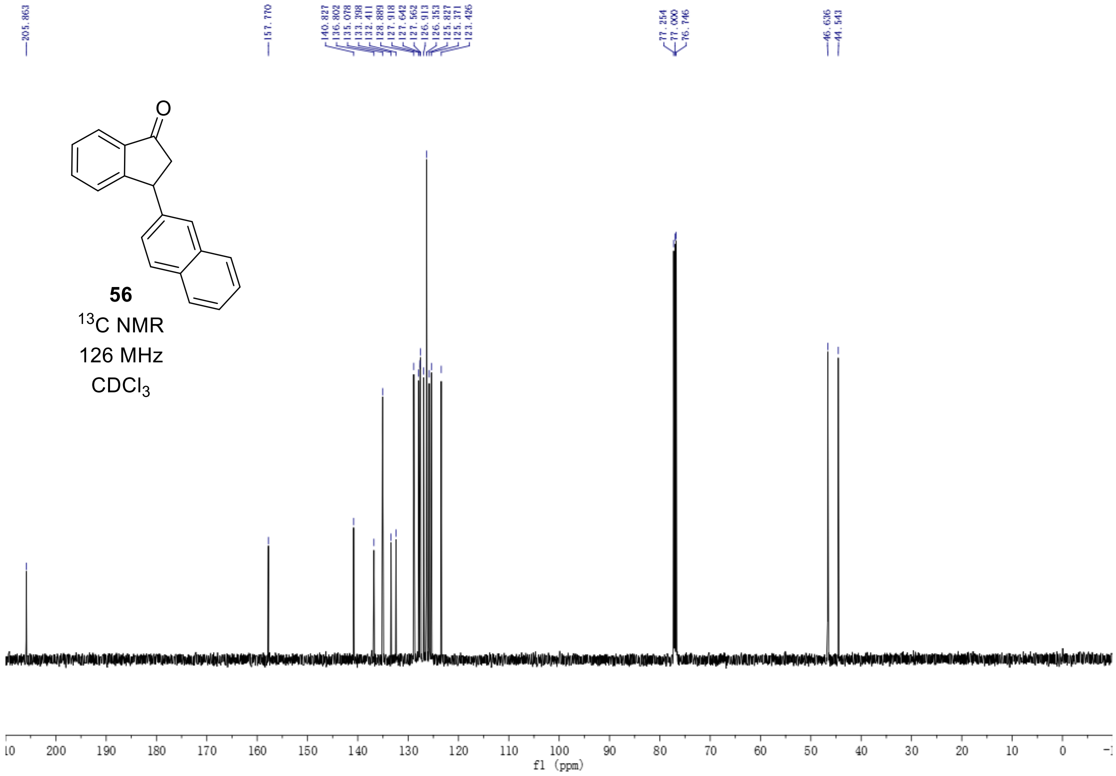

Supplementary Fig. 125 ^{13}C NMR spectrum of compound **56**

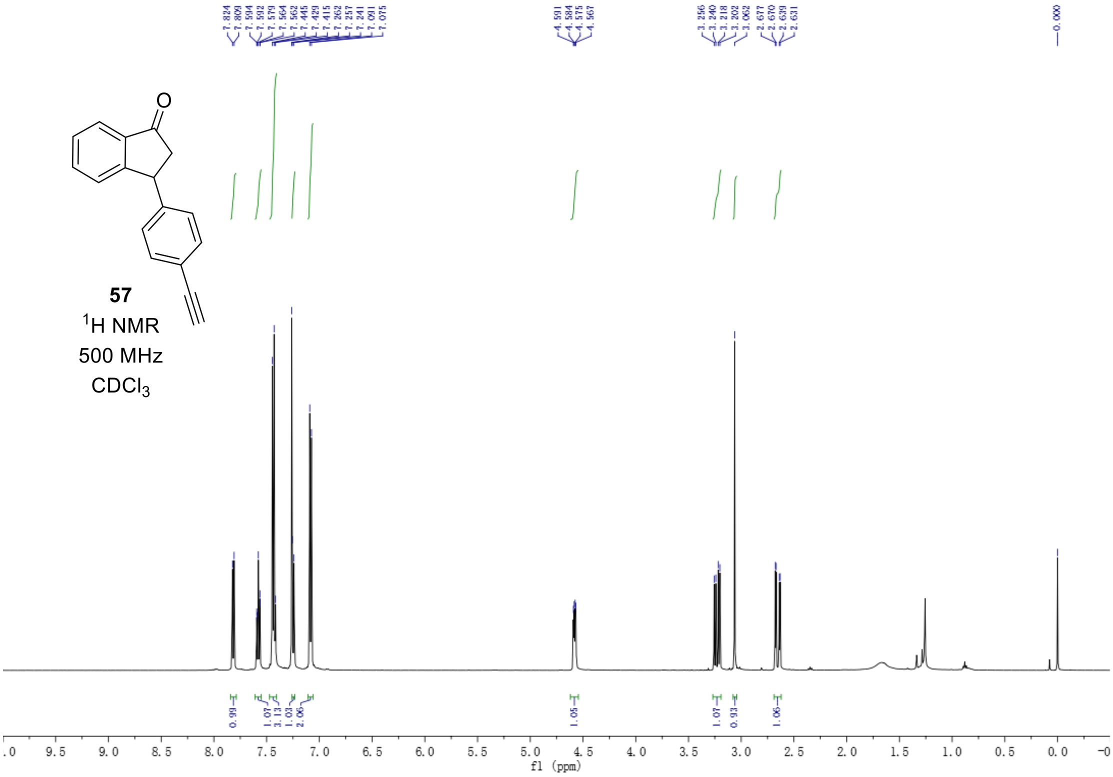

Supplementary Fig. 126 ^{1}H NMR spectrum of compound **57**

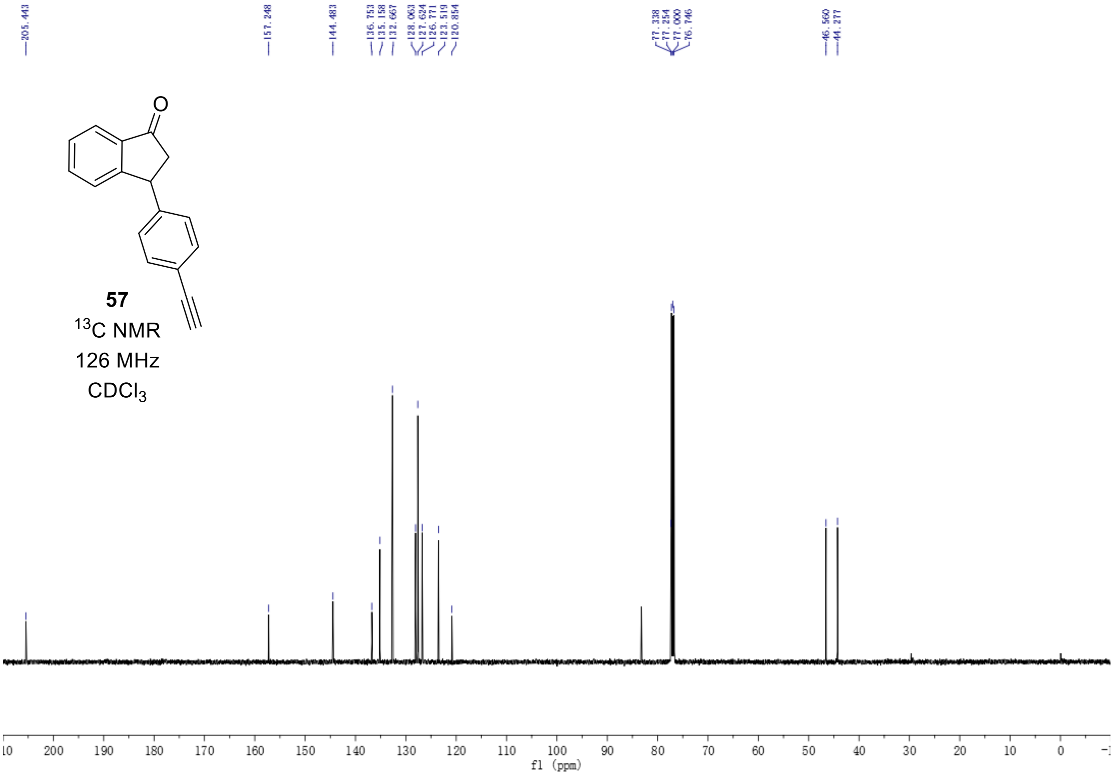

Supplementary Fig. 127 ^{13}C NMR spectrum of compound **57**

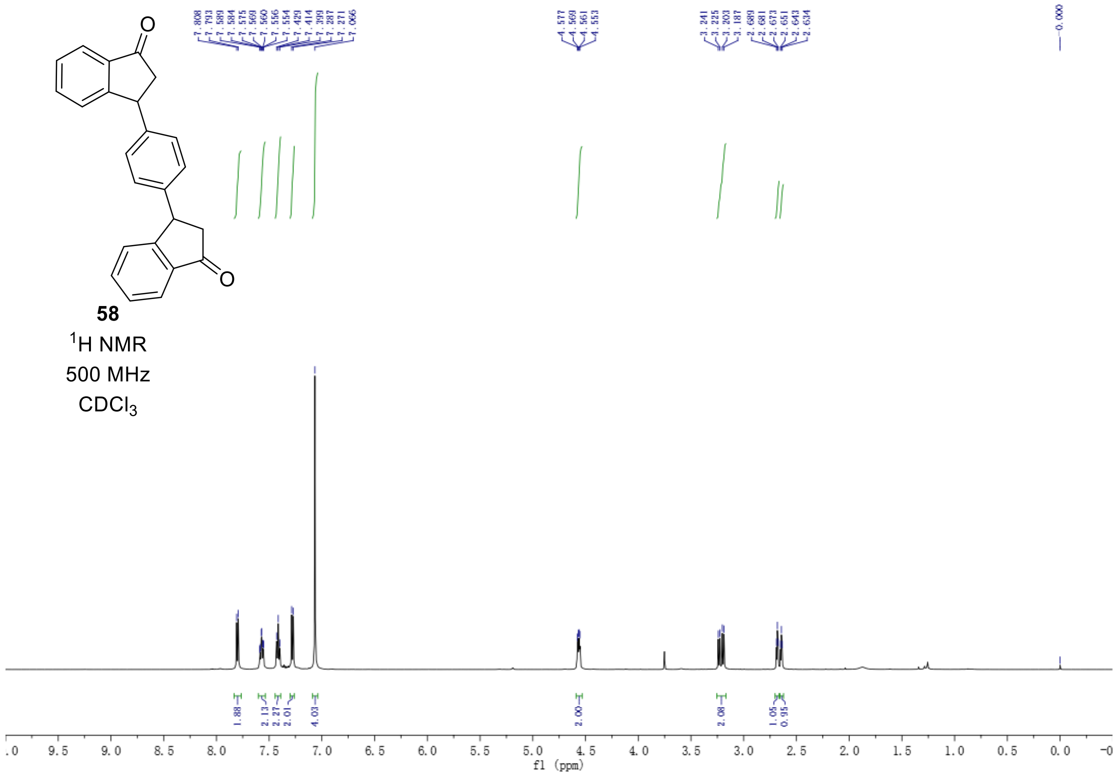

Supplementary Fig. 128 ^{1}H NMR spectrum of compound **58**

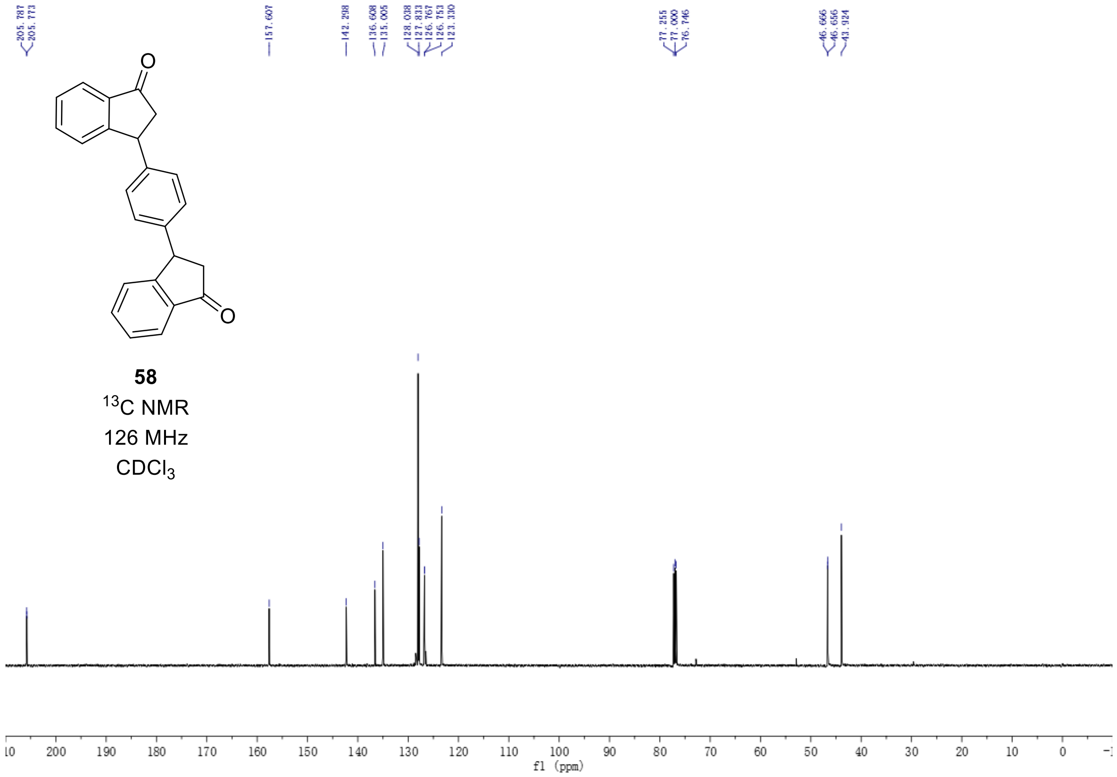

Supplementary Fig. 129 ^{13}C NMR spectrum of compound **58**

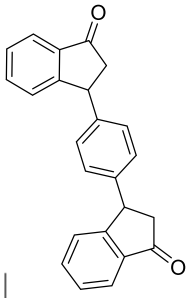

58

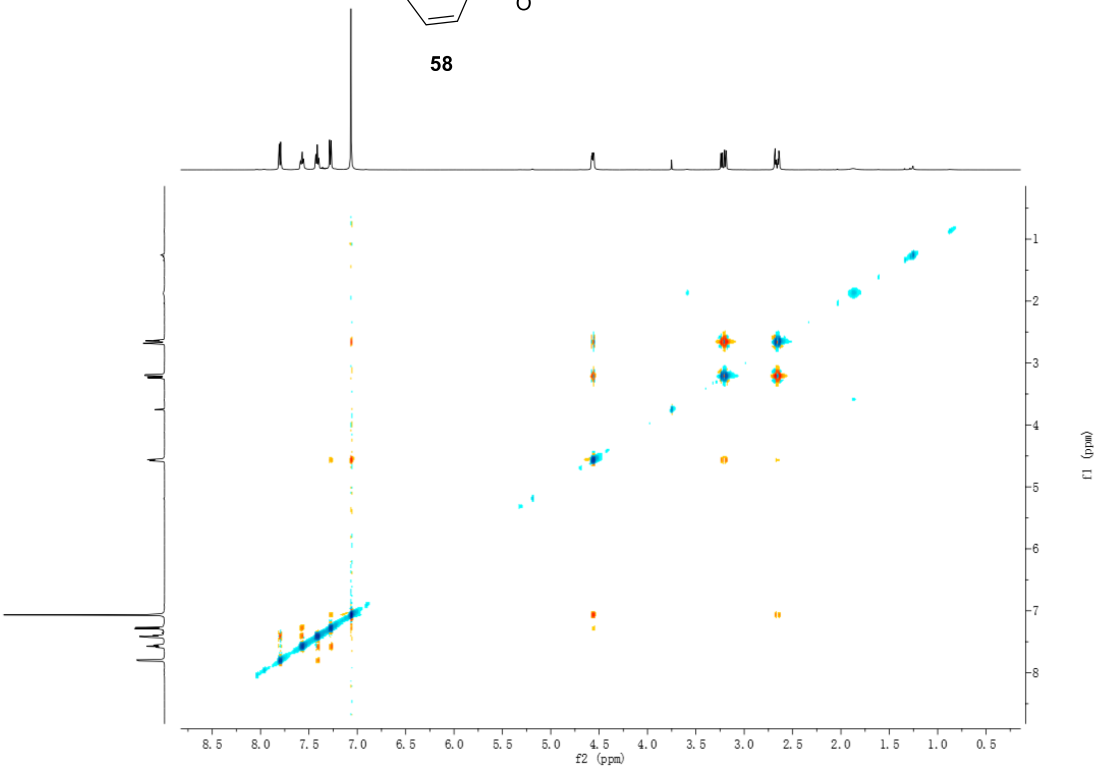

Supplementary Fig. 130 NOESY spectrum of compound **58**

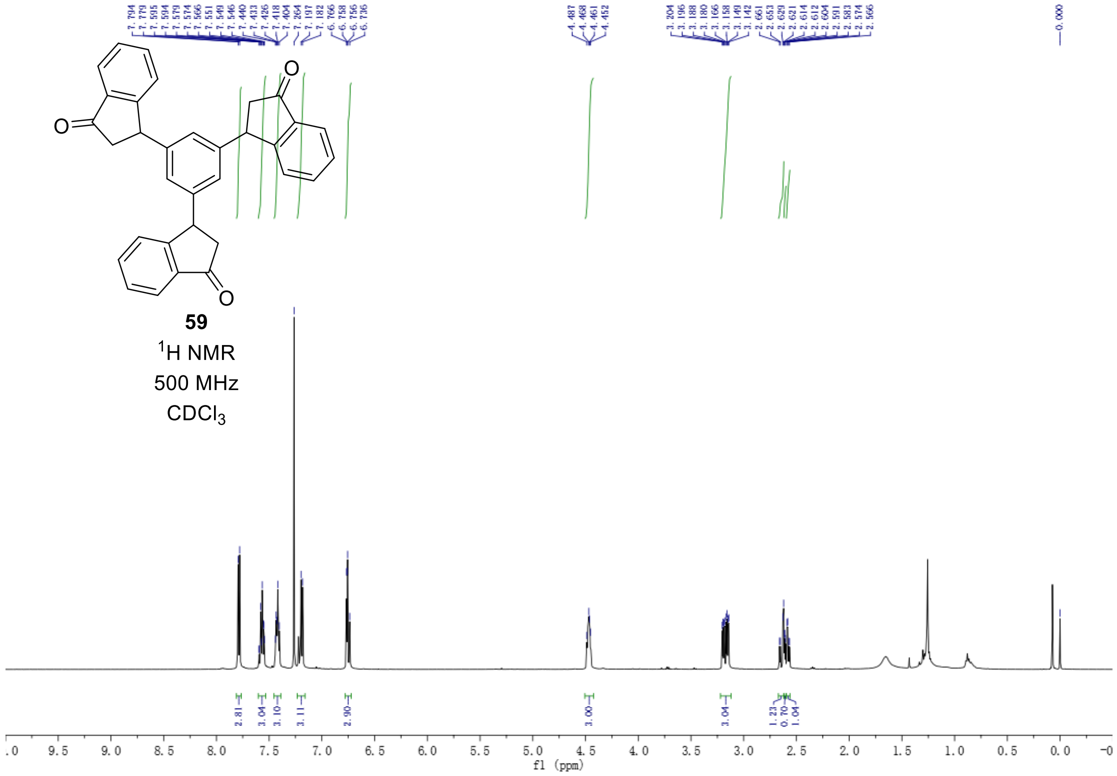

Supplementary Fig. 131 ^{1}H NMR spectrum of compound **59**

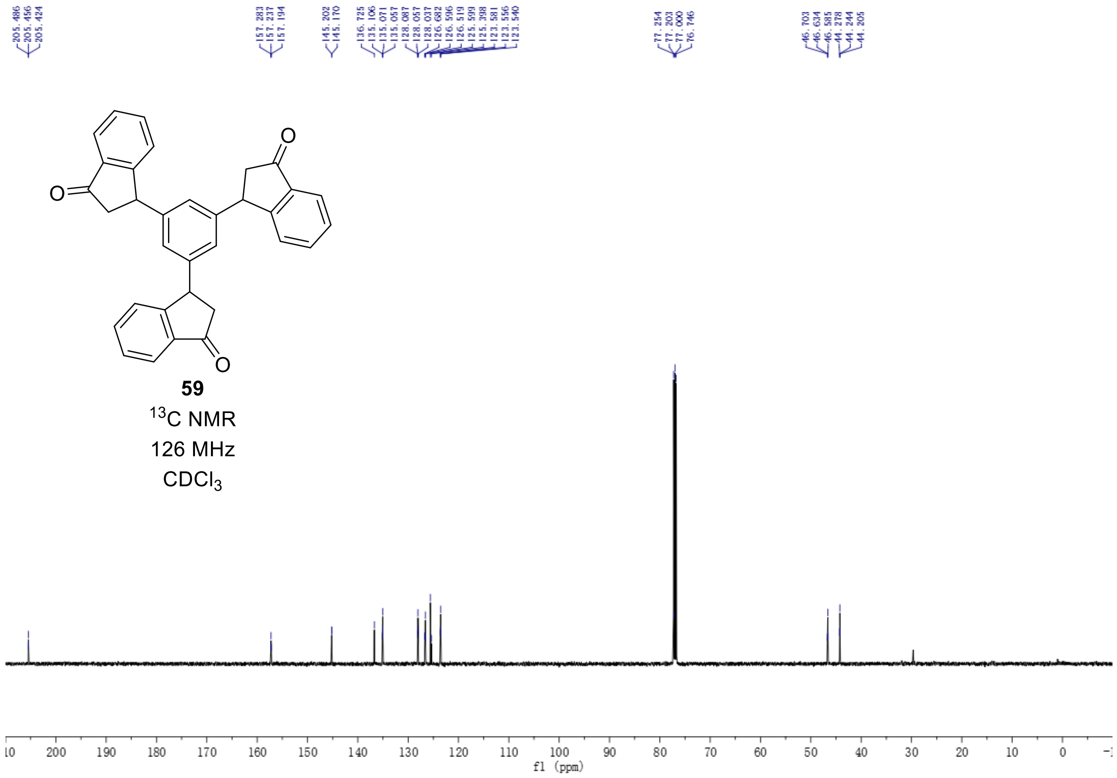

Supplementary Fig. 132 ^{13}C NMR spectrum of compound **59**

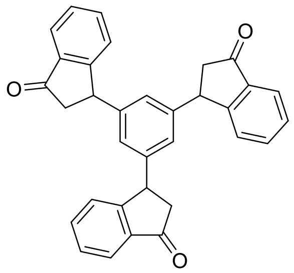

59

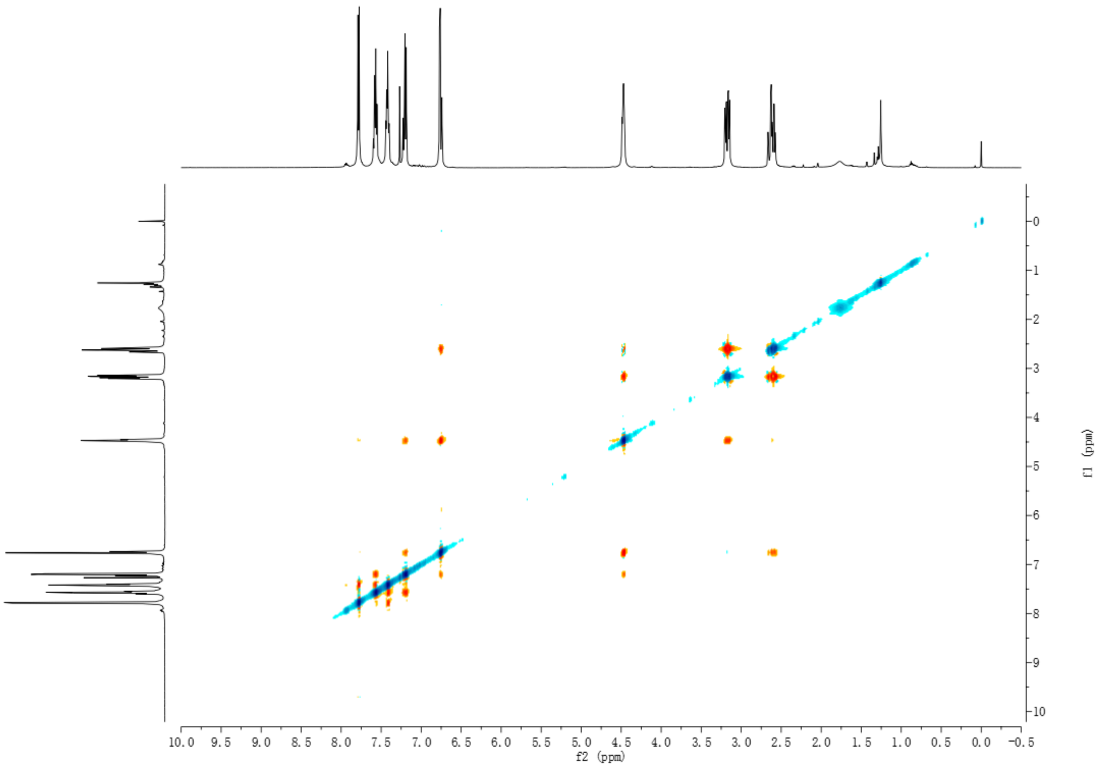

Supplementary Fig. 133 NOESY spectrum of compound **59**

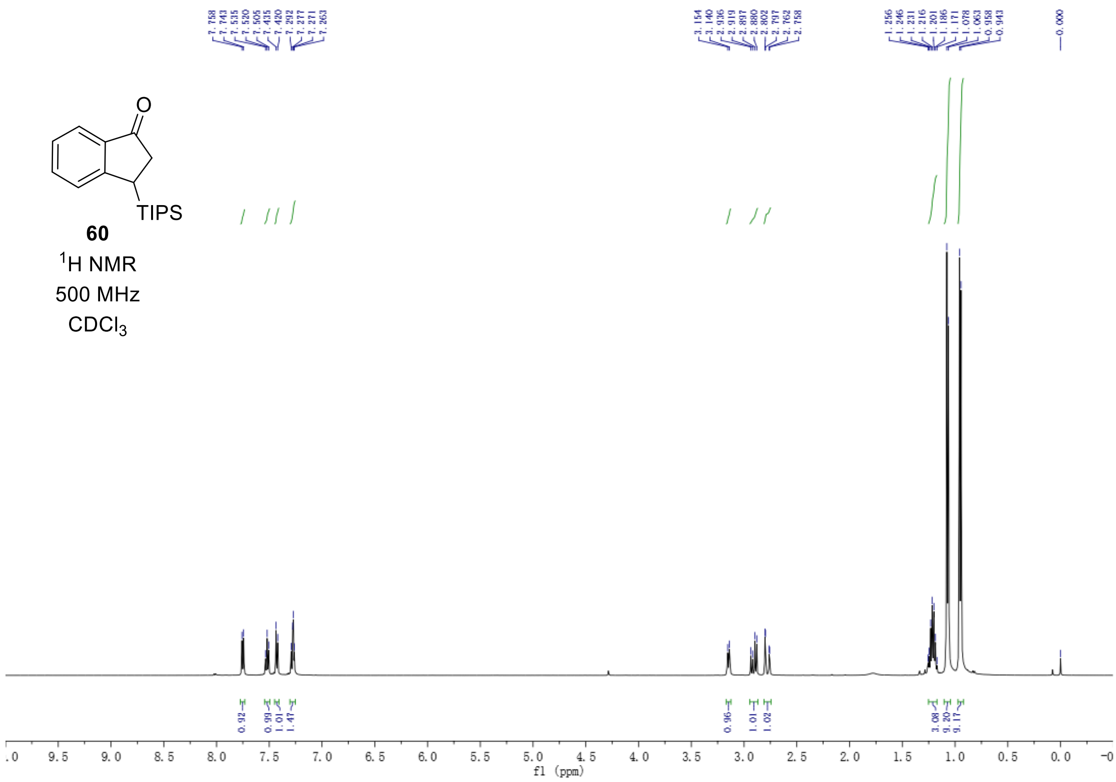

Supplementary Fig. 134 ^{1}H NMR spectrum of compound **60**

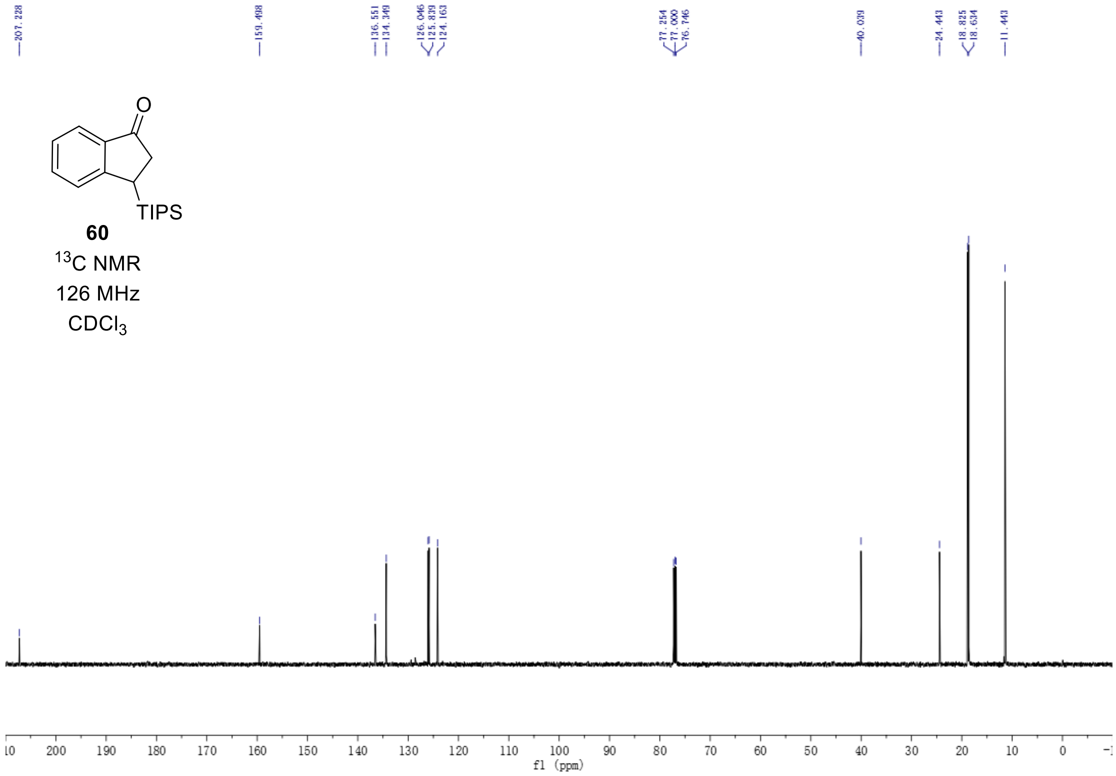

Supplementary Fig. 135 ^{13}C NMR spectrum of compound **60**

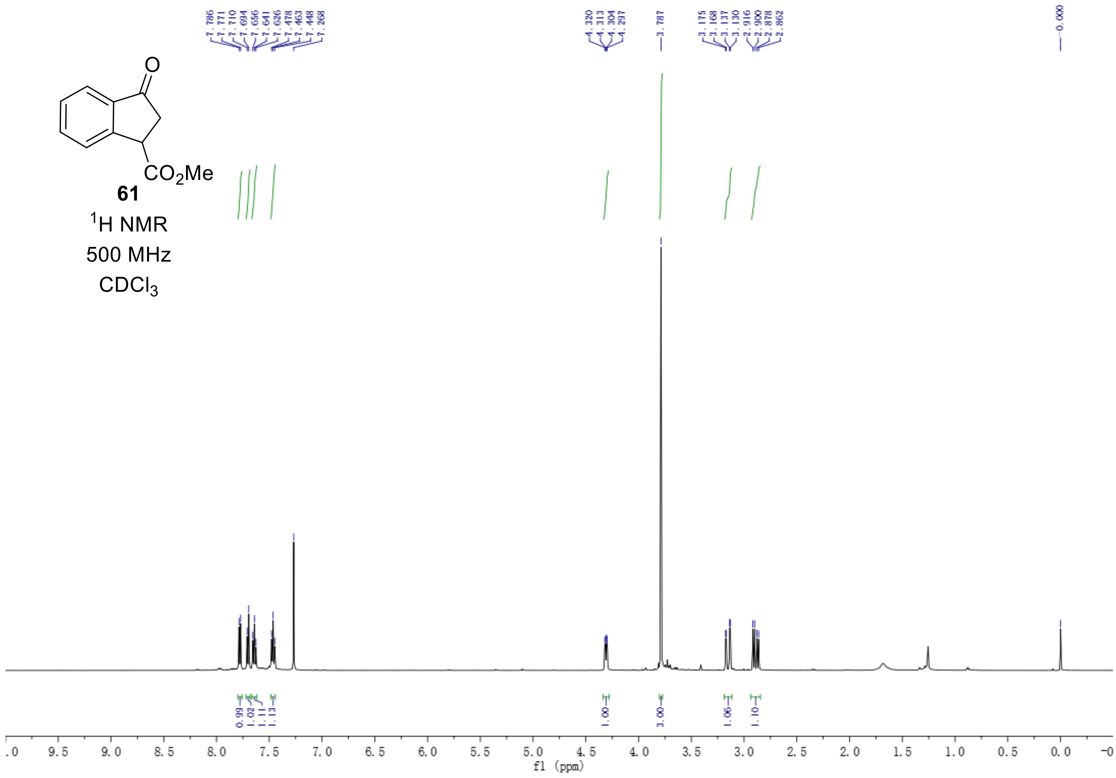

Supplementary Fig. 136 ^{1}H NMR spectrum of compound **61**

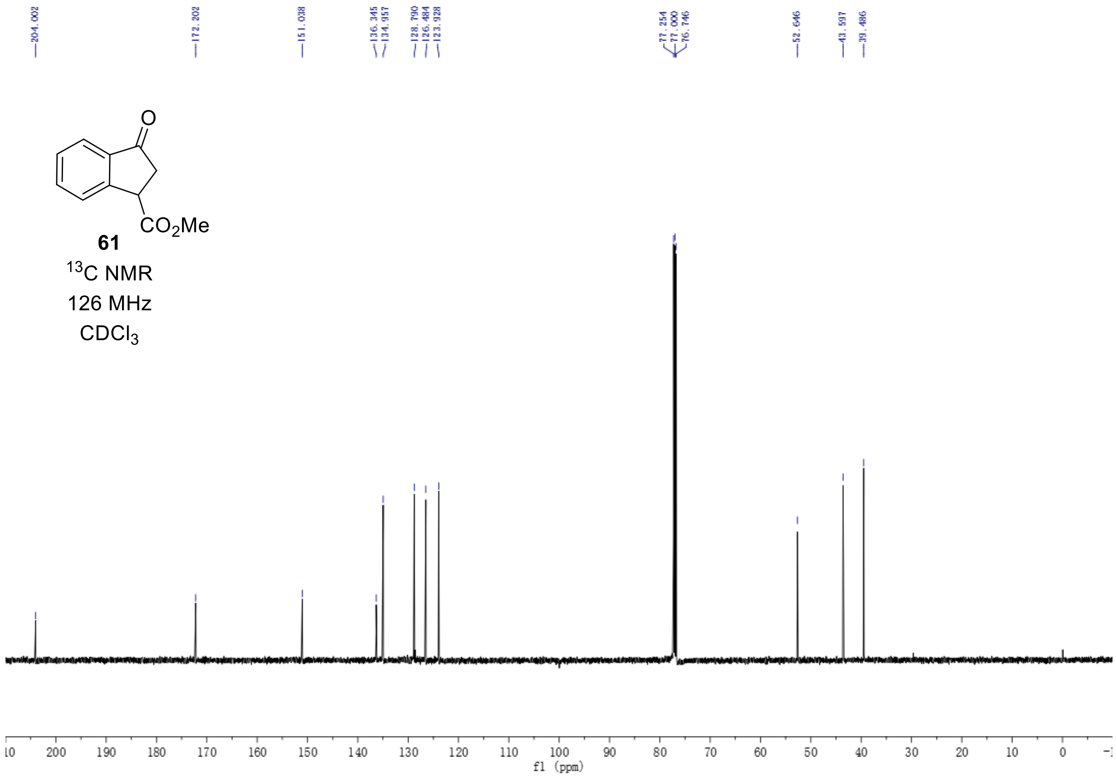

Supplementary Fig. 137 ^{13}C NMR spectrum of compound **61**

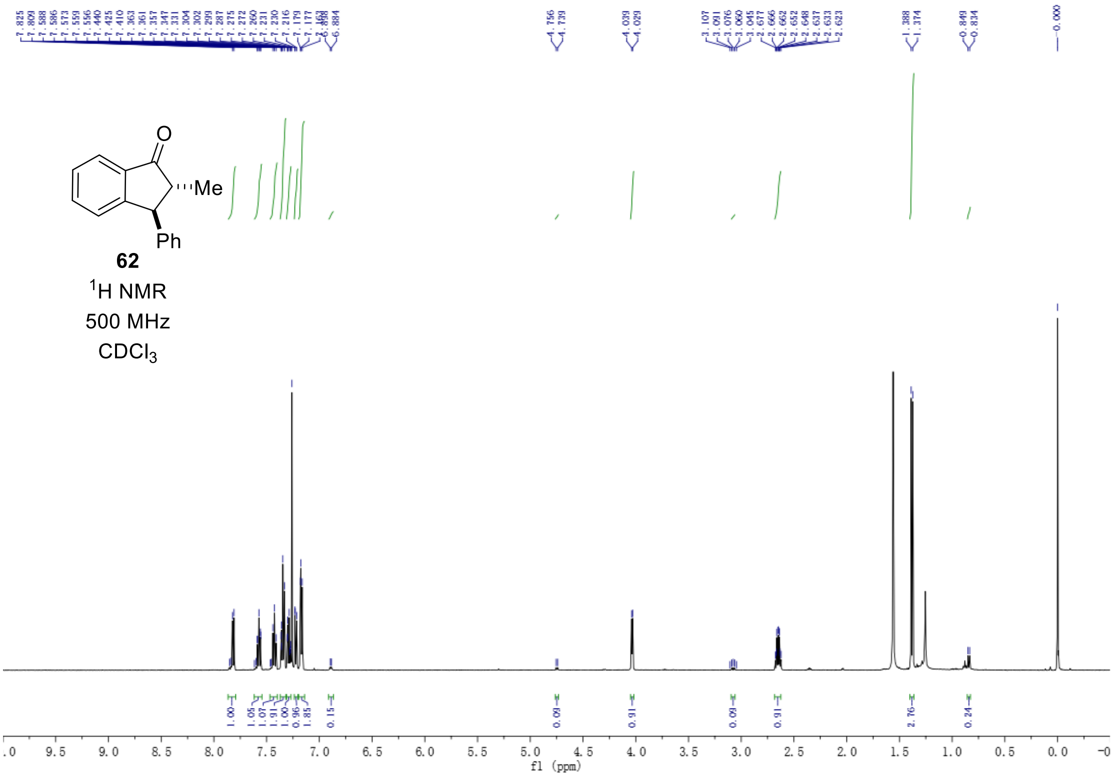

Supplementary Fig. 138 ^{1}H NMR spectrum of compound **62**

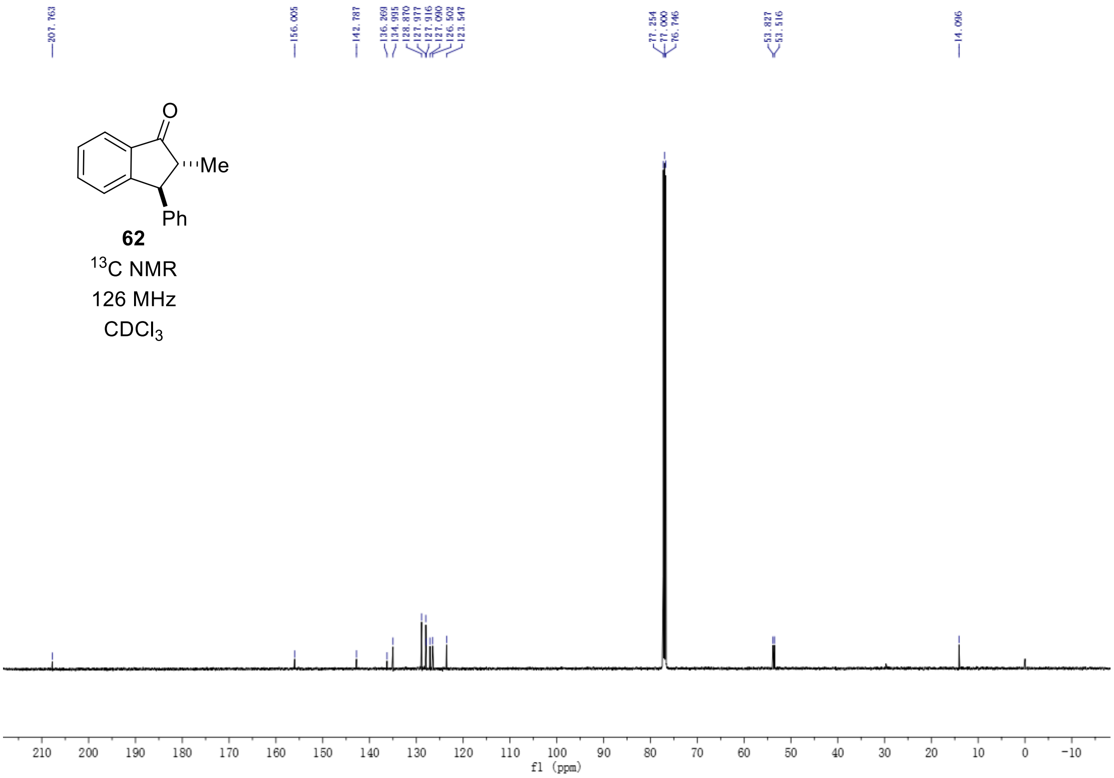

Supplementary Fig. 139 ^{13}C NMR spectrum of compound **62**

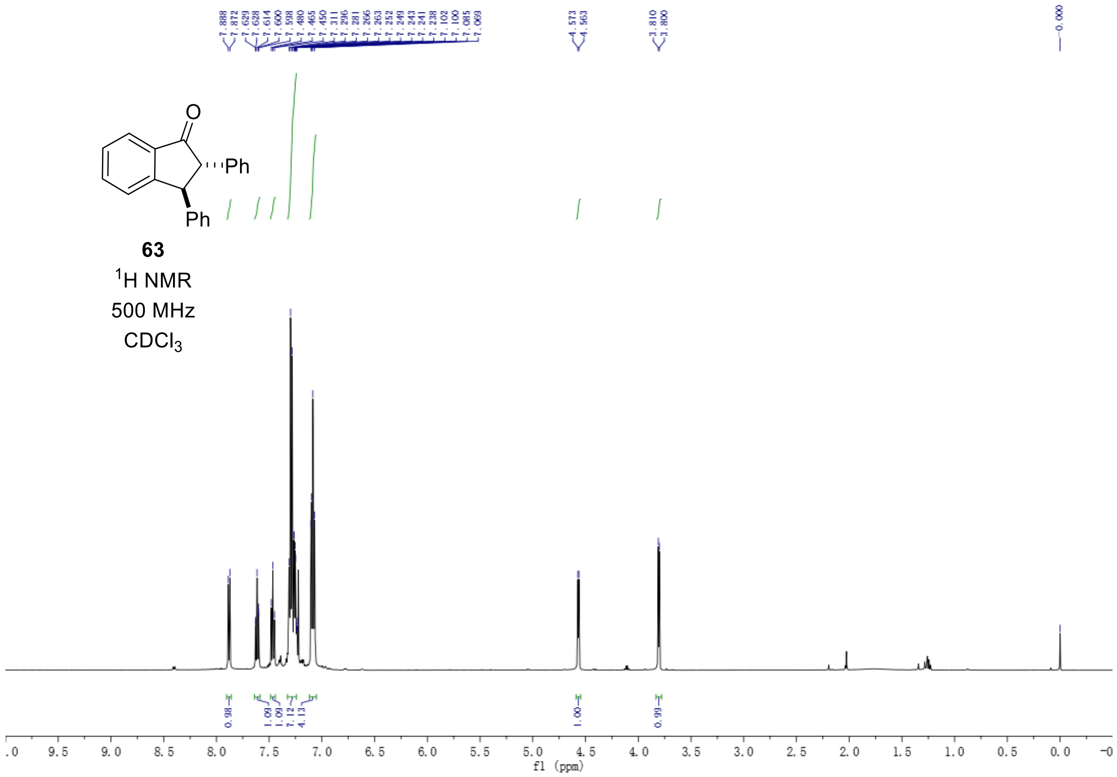

Supplementary Fig. 140 ^{1}H NMR spectrum of compound **63**

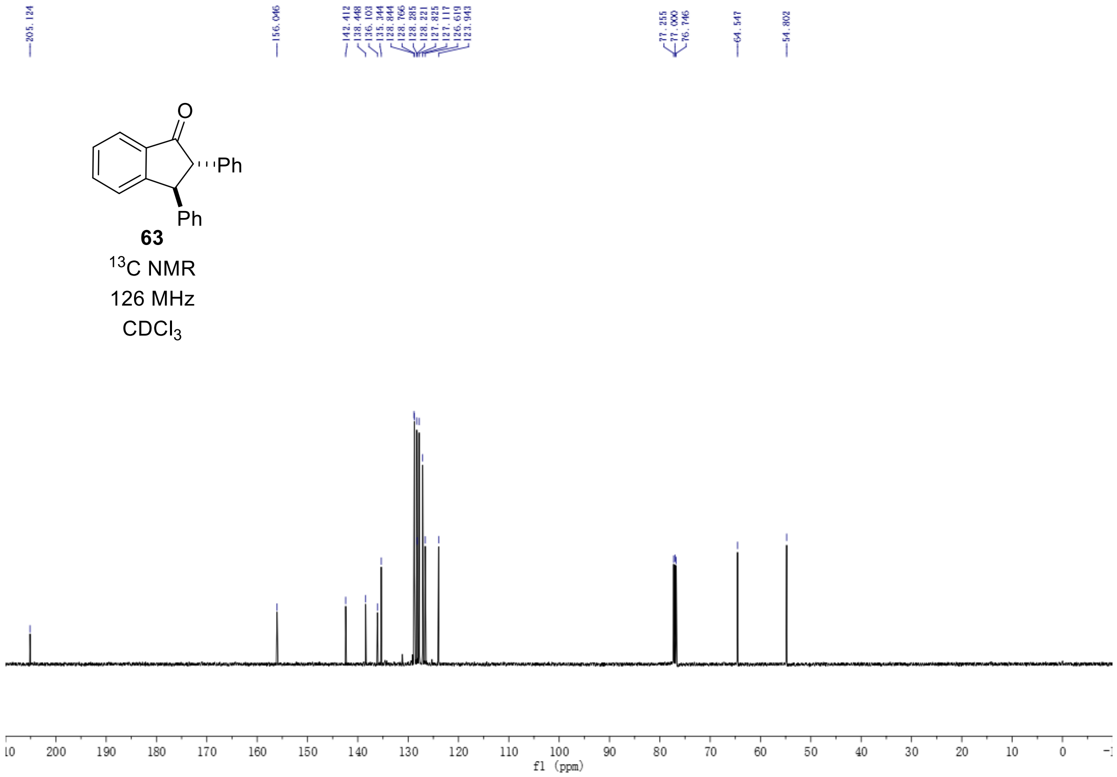

Supplementary Fig. 141 ^{13}C NMR spectrum of compound **63**

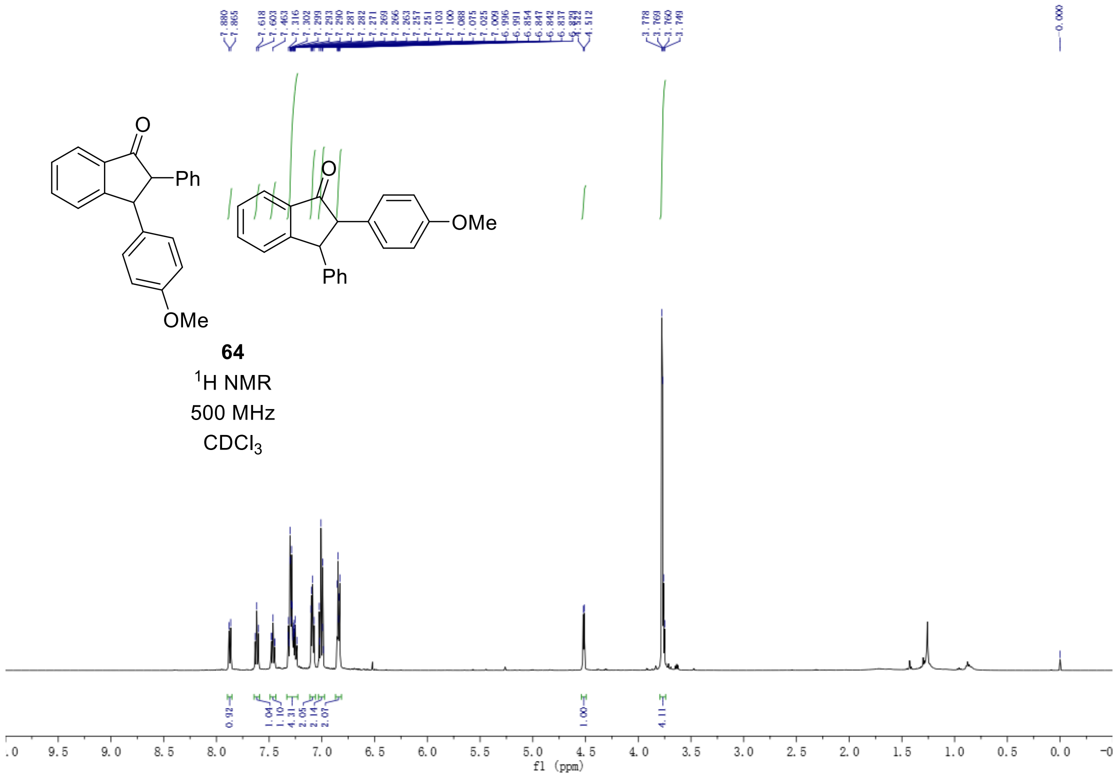

Supplementary Fig. 142 ^{1}H NMR spectrum of compound **64**

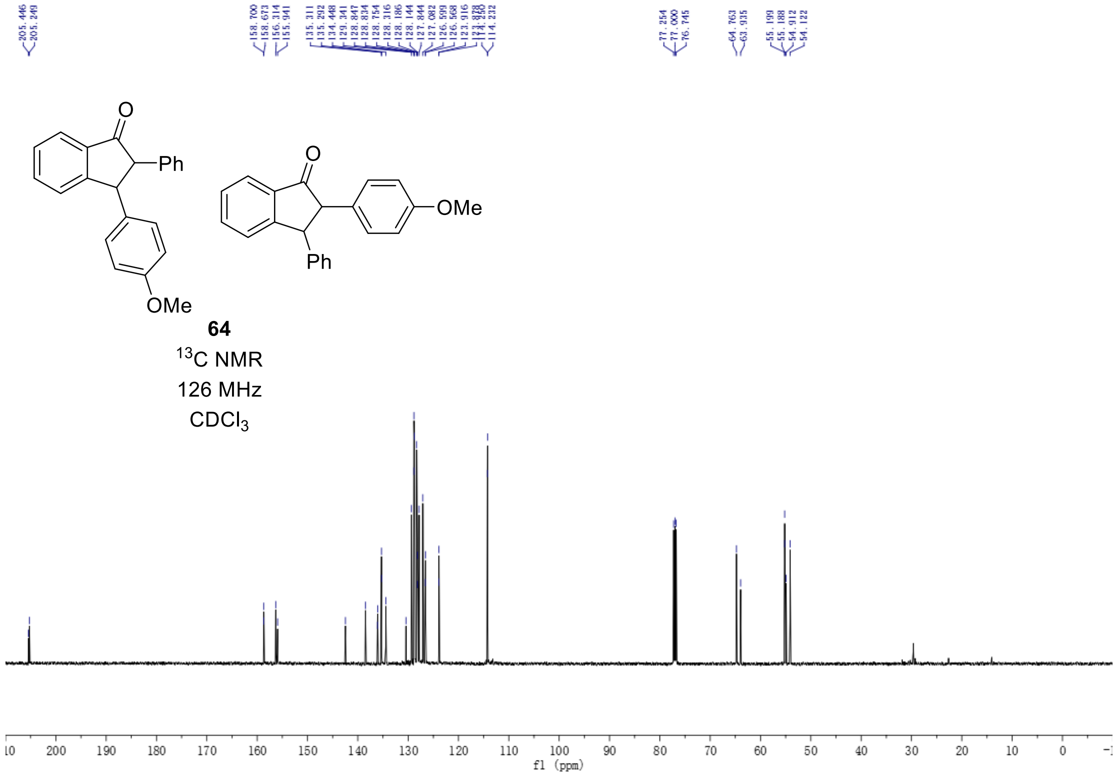

Supplementary Fig. 143 ^{13}C NMR spectrum of compound **69**

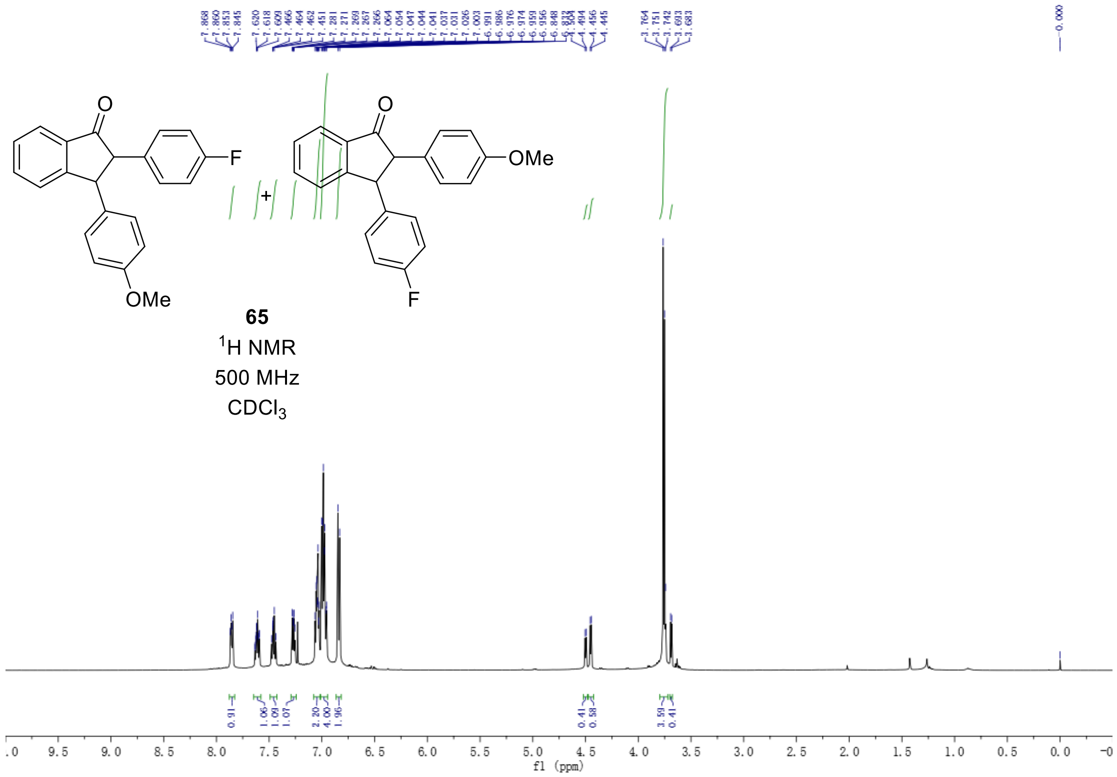

Supplementary Fig. 144 ^{1}H NMR spectrum of compound **65**

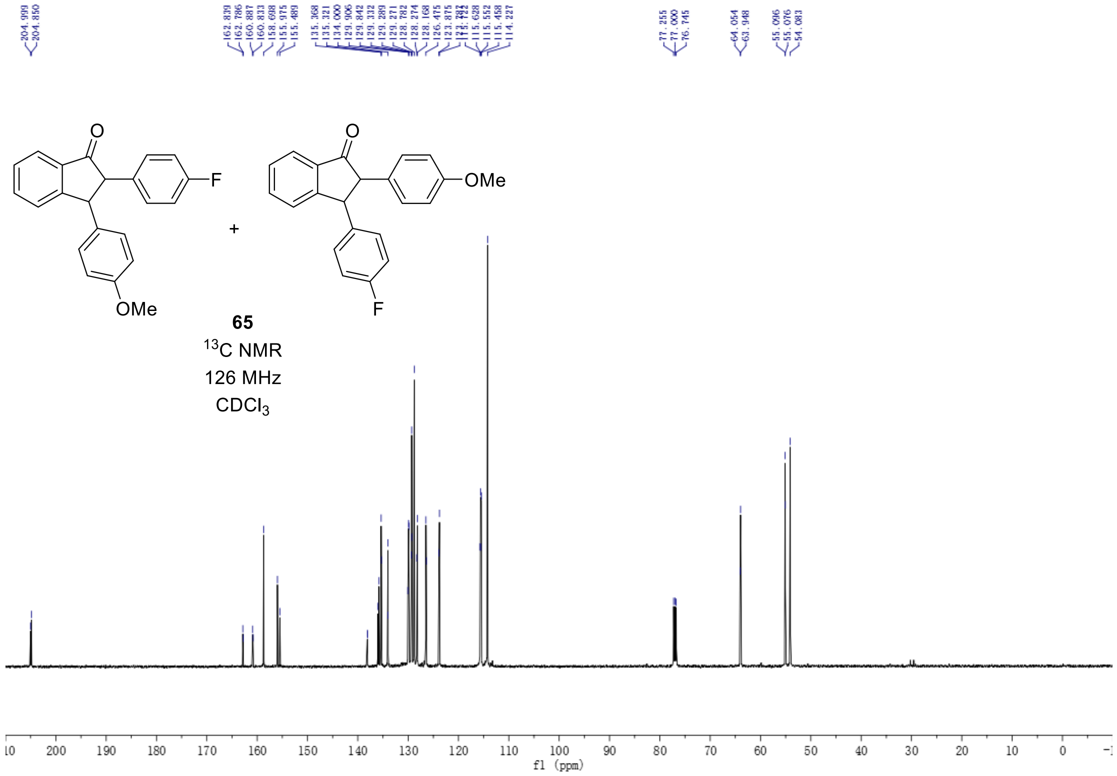

Supplementary Fig. 145 ^{13}C NMR spectrum of compound **65**

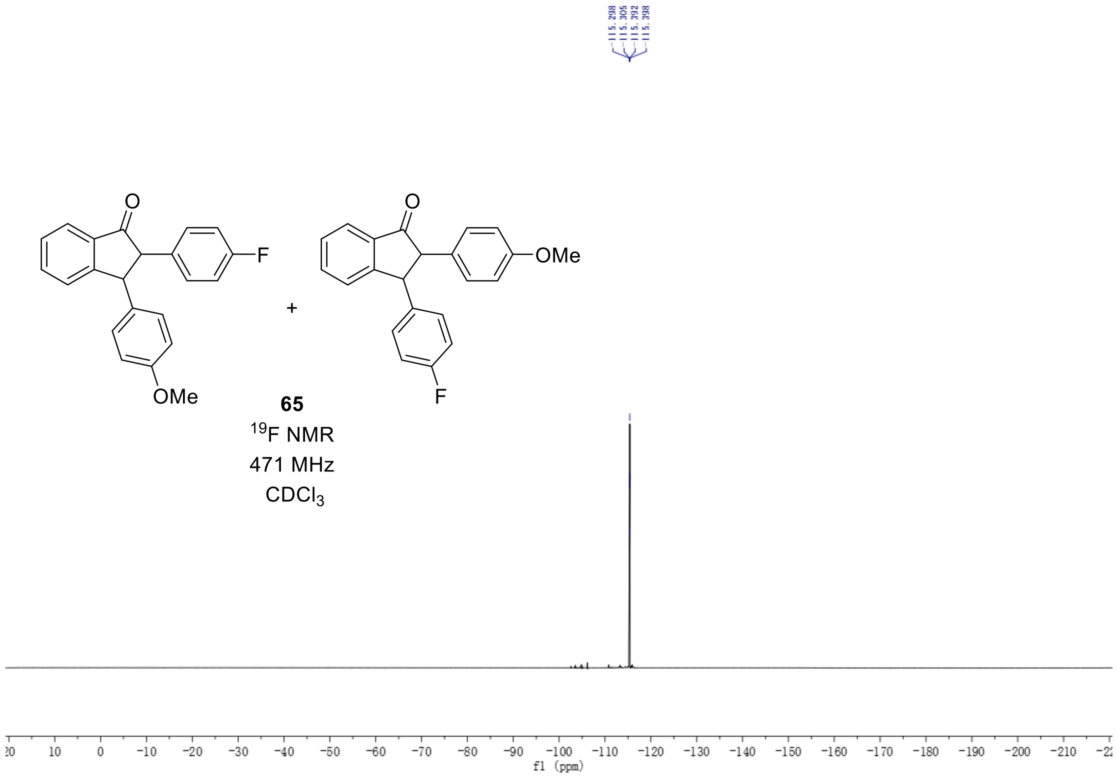

Supplementary Fig. 146 ^{19}F NMR spectrum of compound **65**

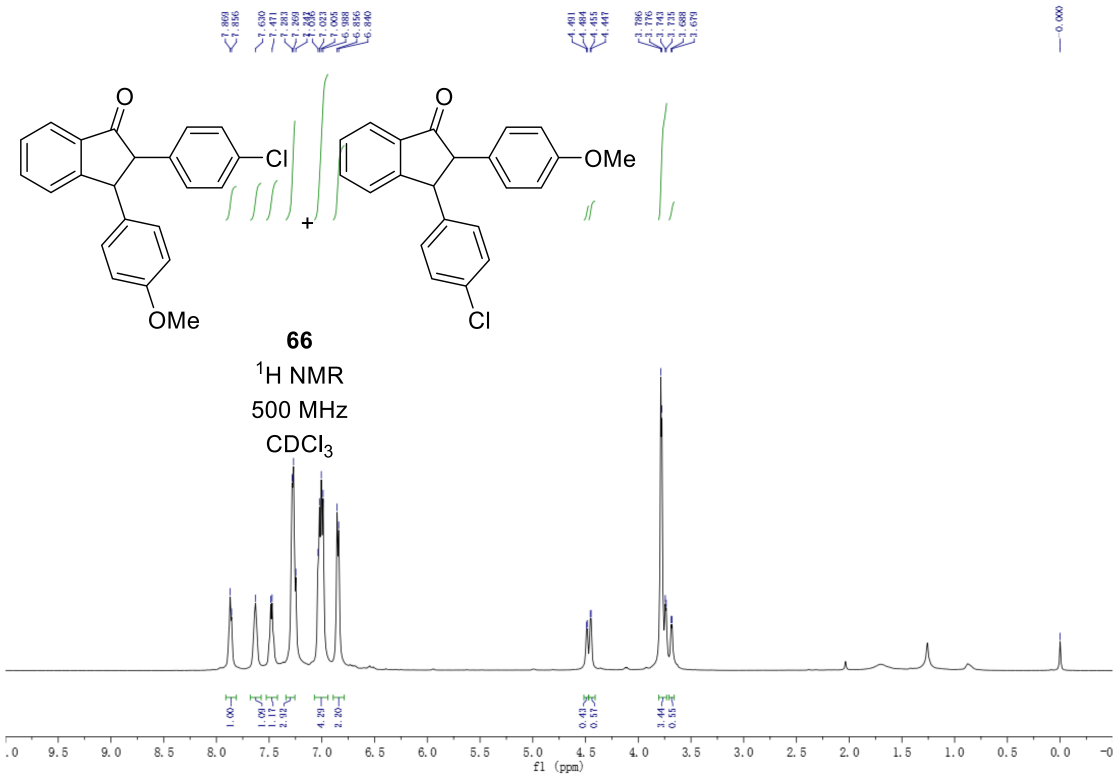

Supplementary Fig. 147 ^{1}H NMR spectrum of compound **66**

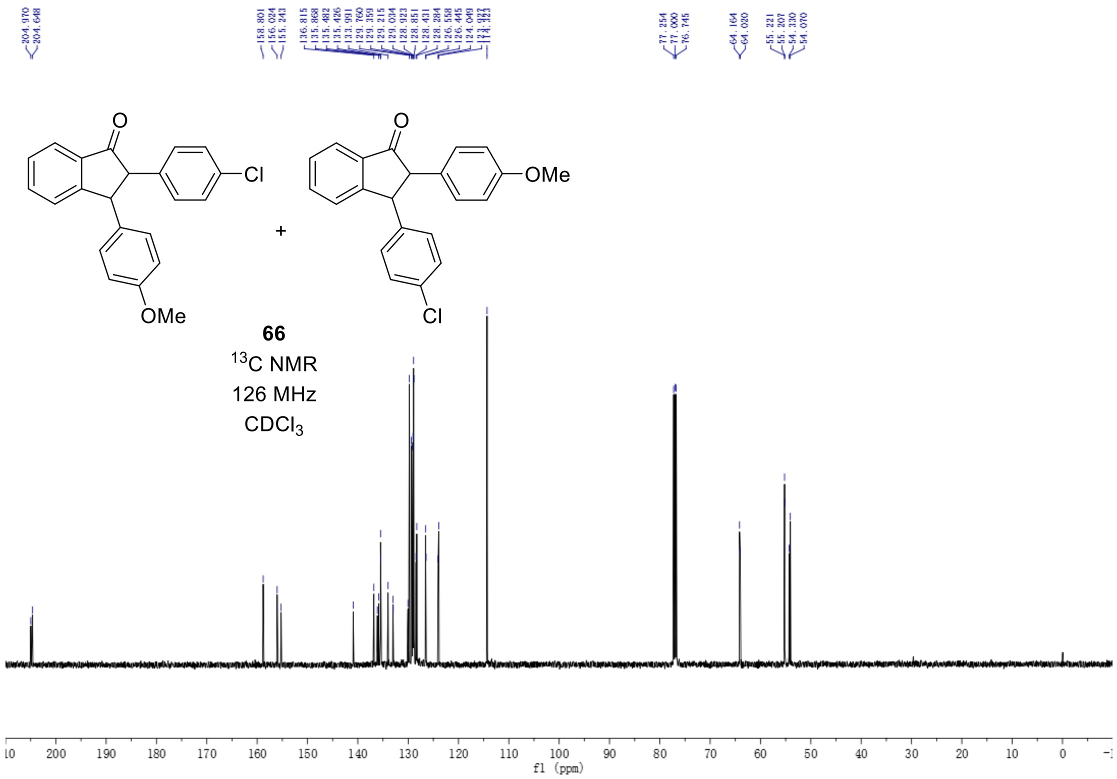

Supplementary Fig. 148 ^{13}C NMR spectrum of compound **66**

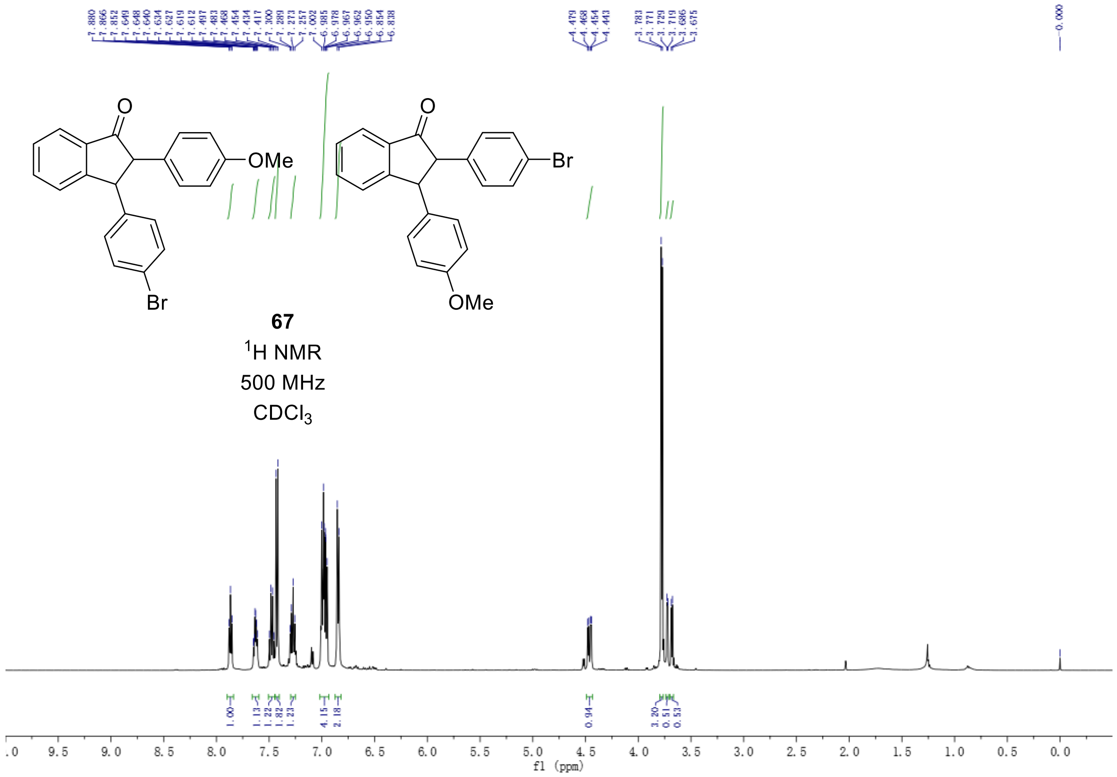

Supplementary Fig. 149 ^{1}H NMR spectrum of compound **67**

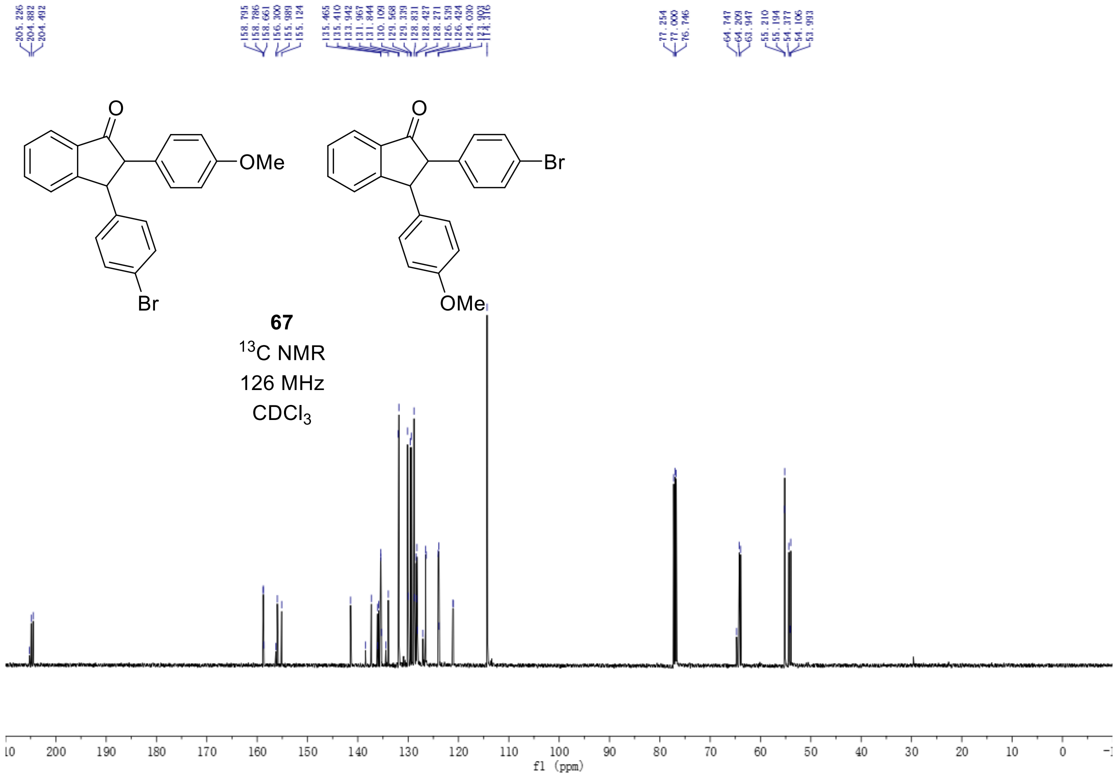

Supplementary Fig. 150 ^{13}C NMR spectrum of compound **67**

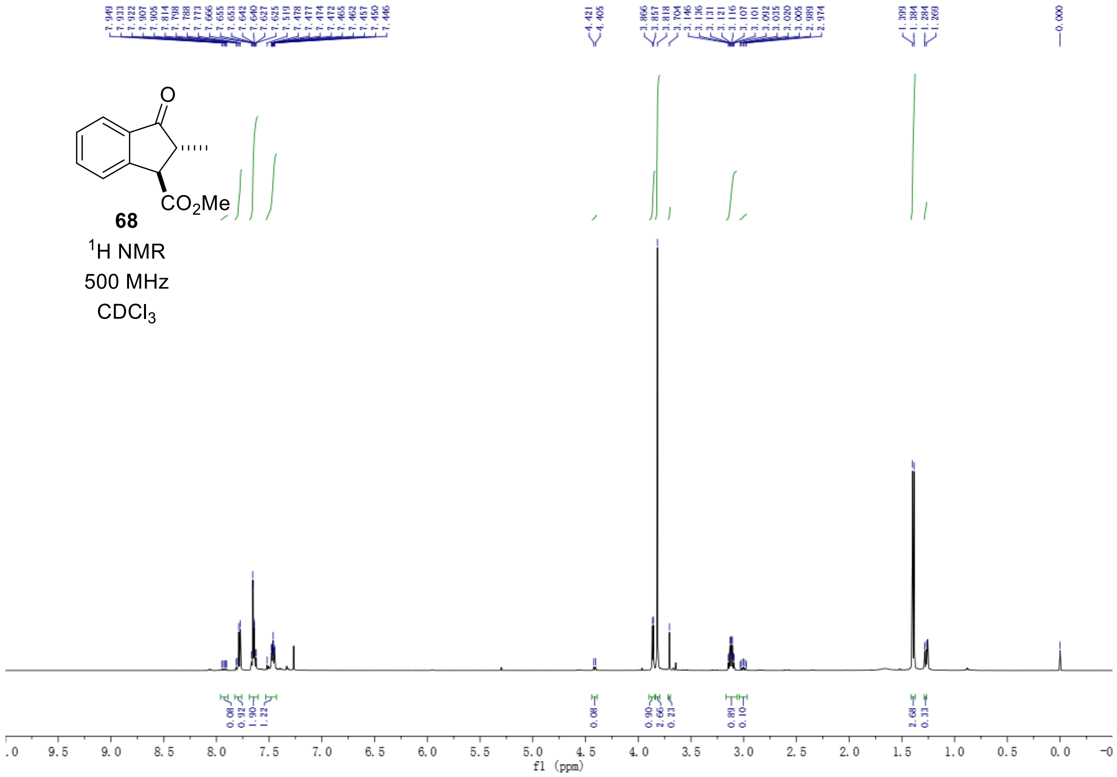

Supplementary Fig. 151 ^{1}H NMR spectrum of compound **68**

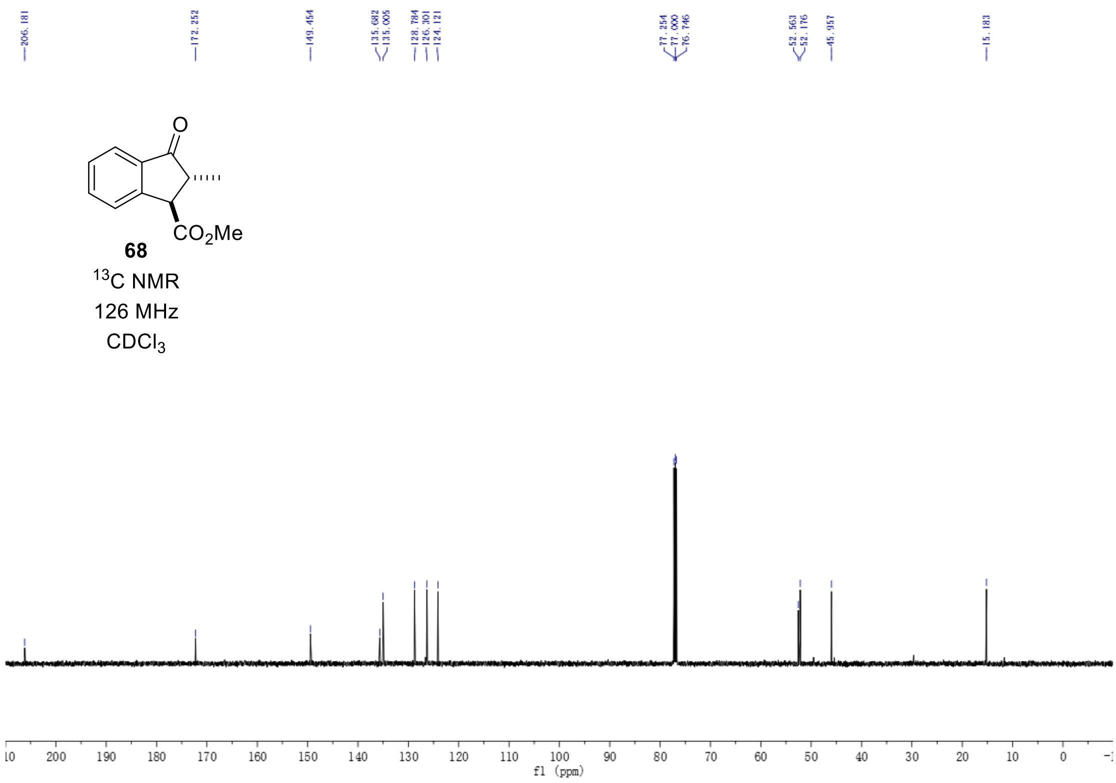

Supplementary Fig. 152 ^{13}C NMR spectrum of compound **68**

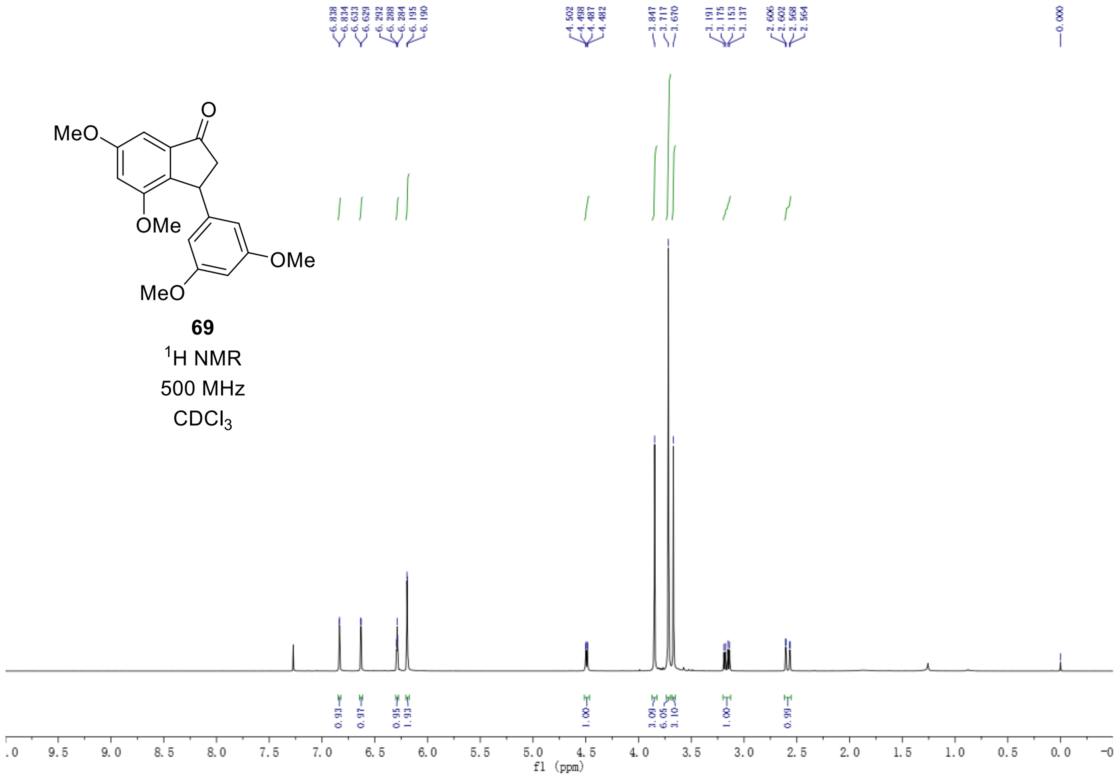

Supplementary Fig. 153 ^{1}H NMR spectrum of compound **69**

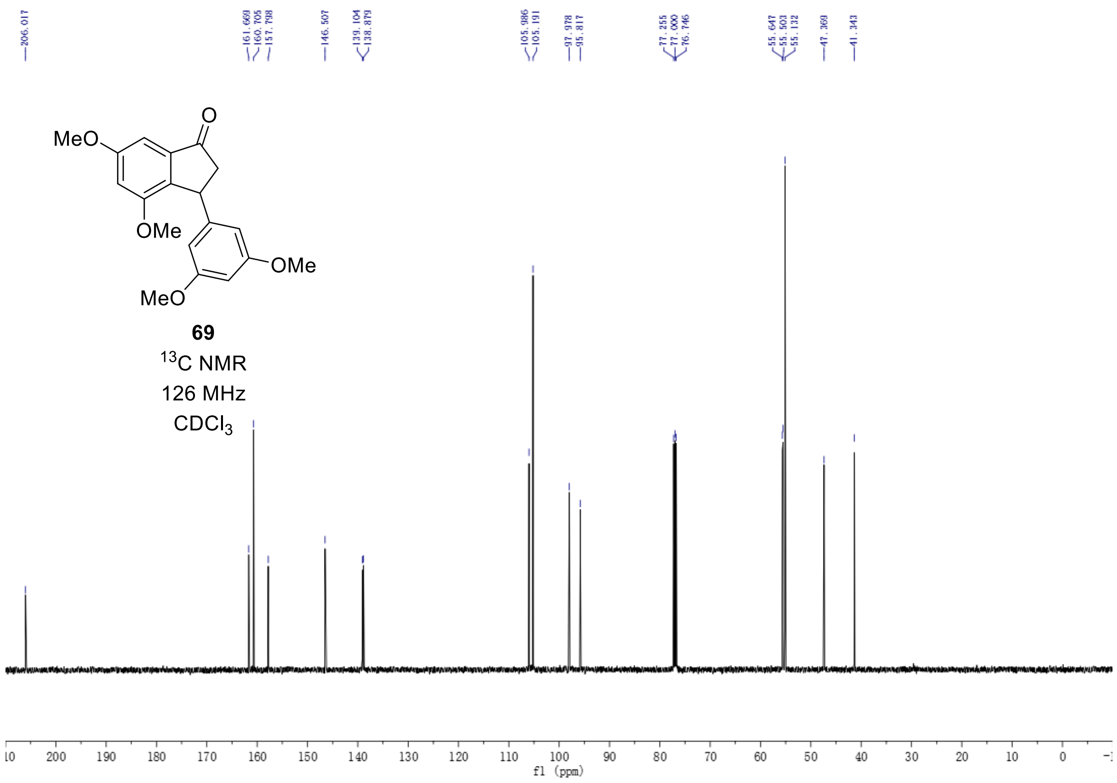

Supplementary Fig. 154 ^{13}C NMR spectrum of compound **69**

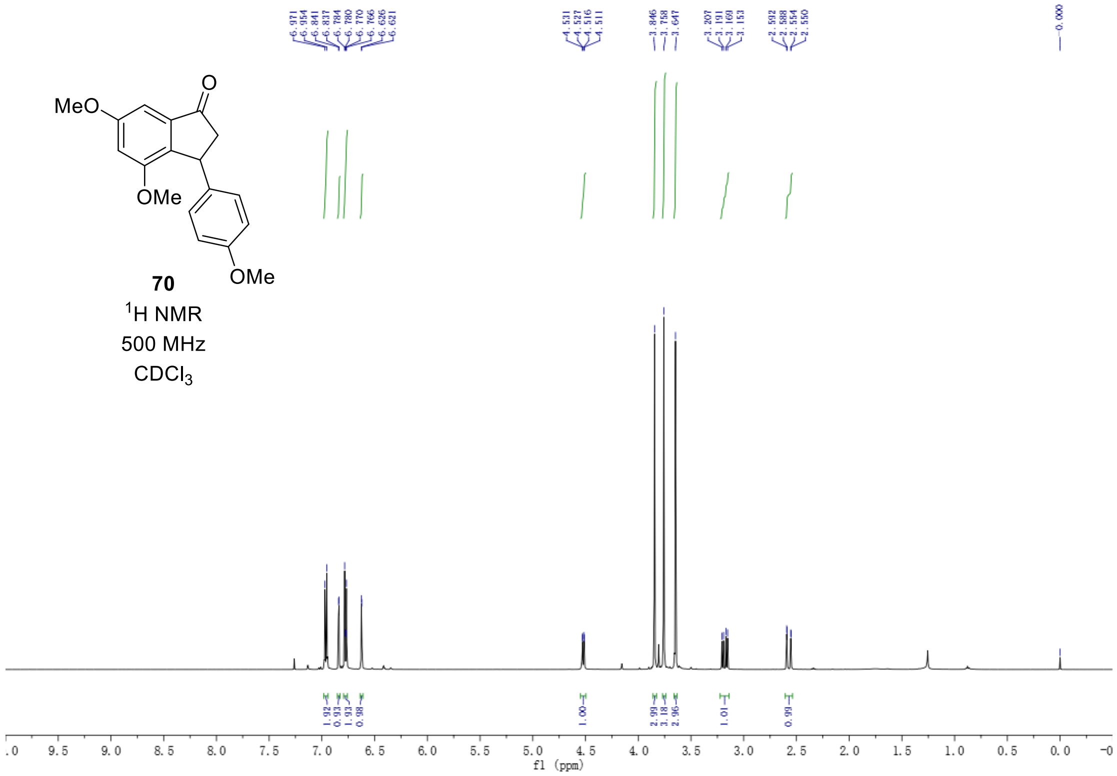

Supplementary Fig. 155 ^{1}H NMR spectrum of compound **70**

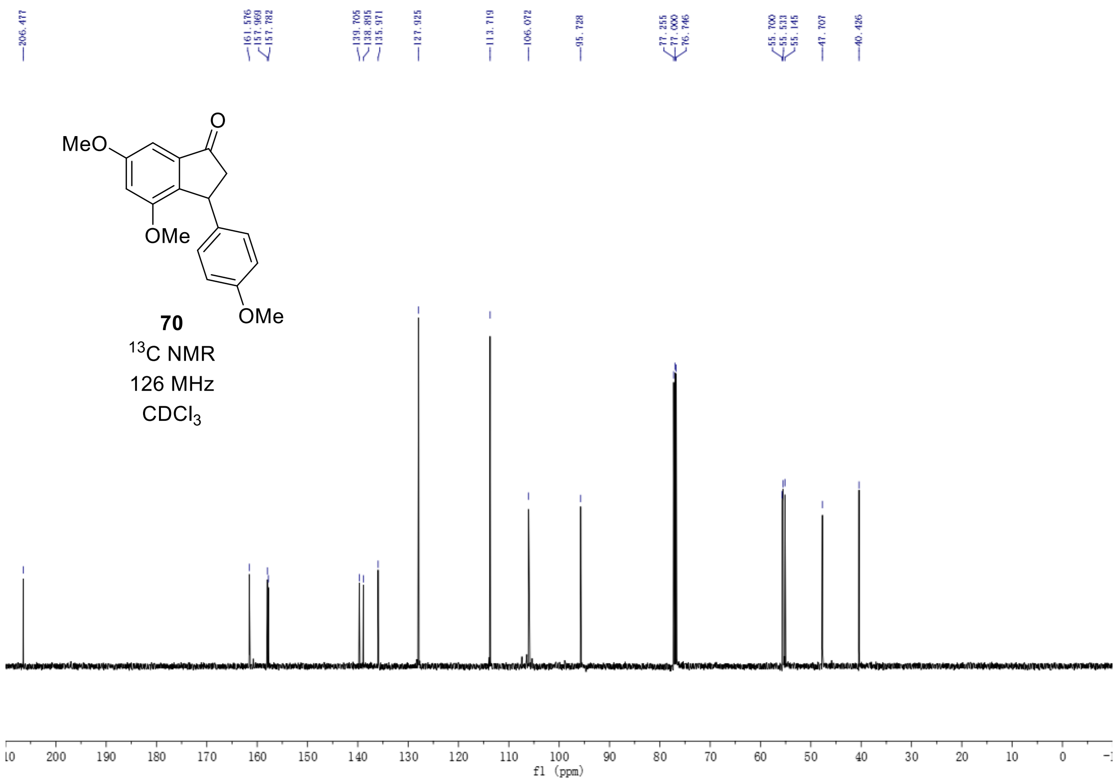

Supplementary Fig. 156 ^{13}C NMR spectrum of compound **70**

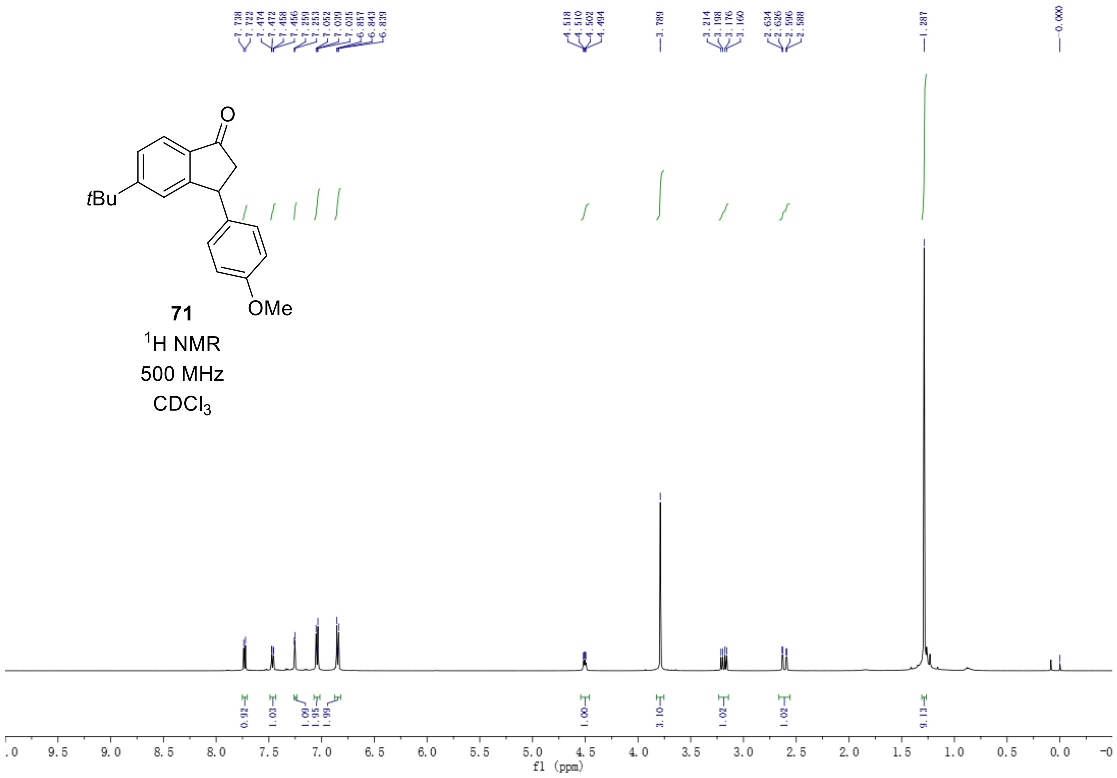

Supplementary Fig. 157 ^{1}H NMR spectrum of compound **71**

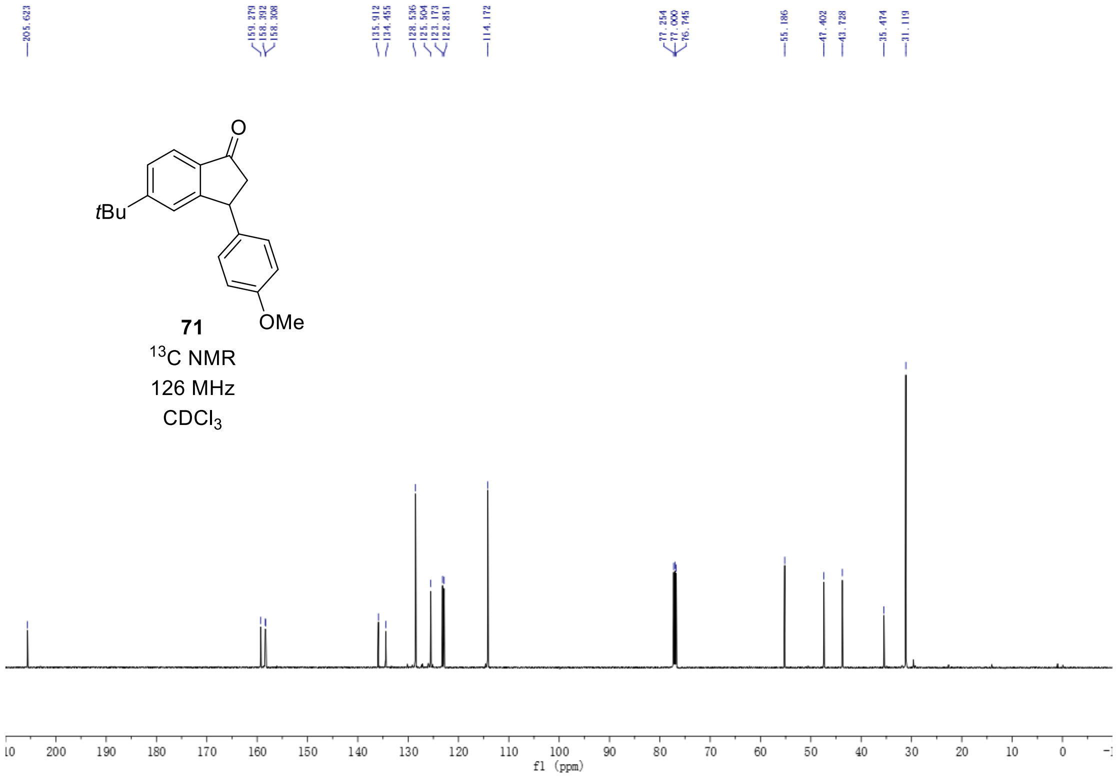

Supplementary Fig. 158 ^{13}C NMR spectrum of compound **71**

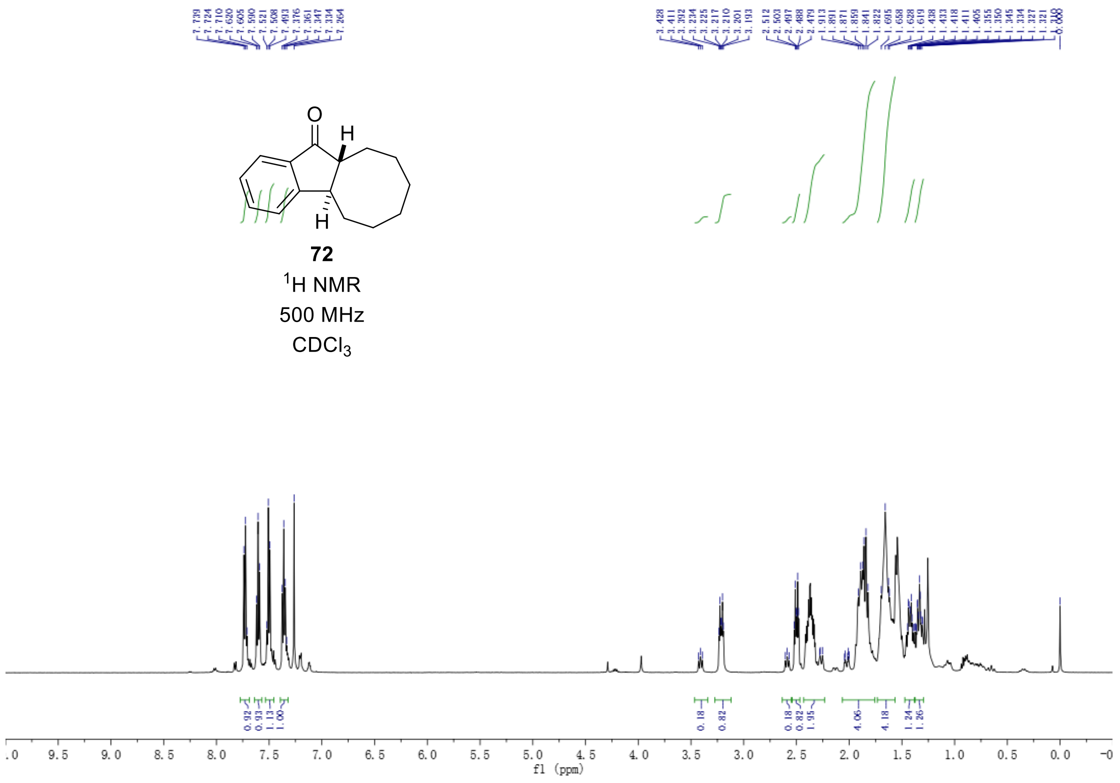

Supplementary Fig. 159 ^{1}H NMR spectrum of compound **72**

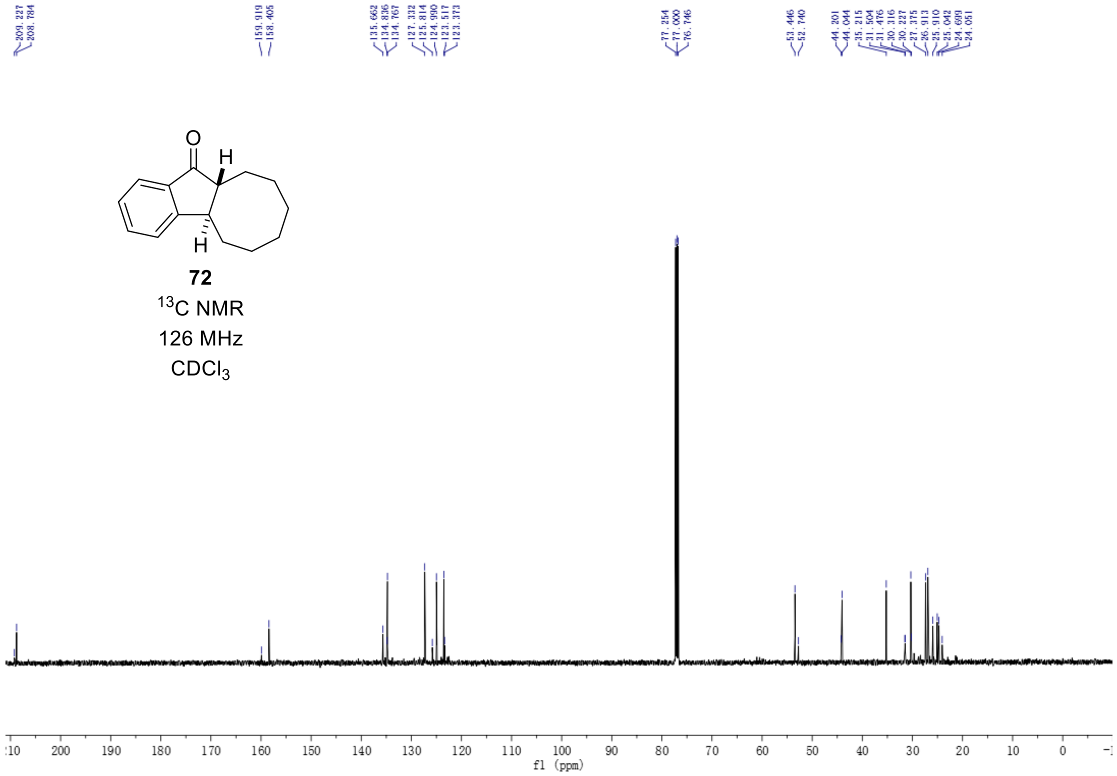

Supplementary Fig. 160 ^{13}C NMR spectrum of compound **72**

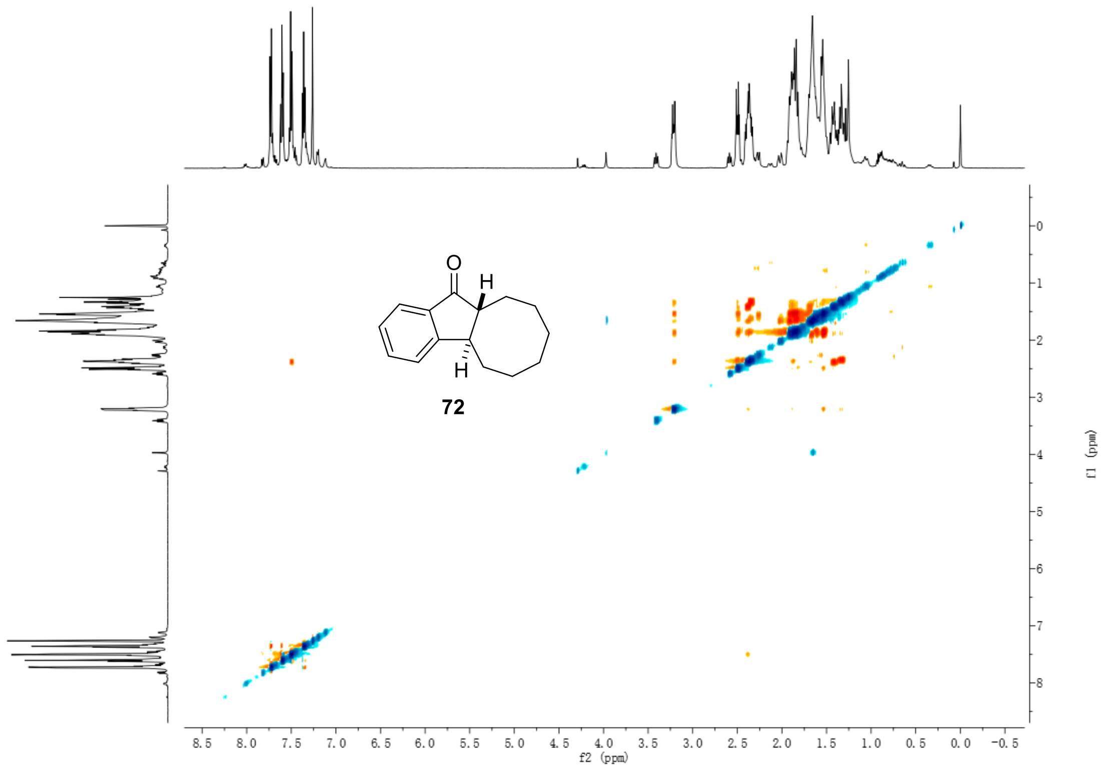

Supplementary Fig. 161 NOESY spectrum of compound **72**

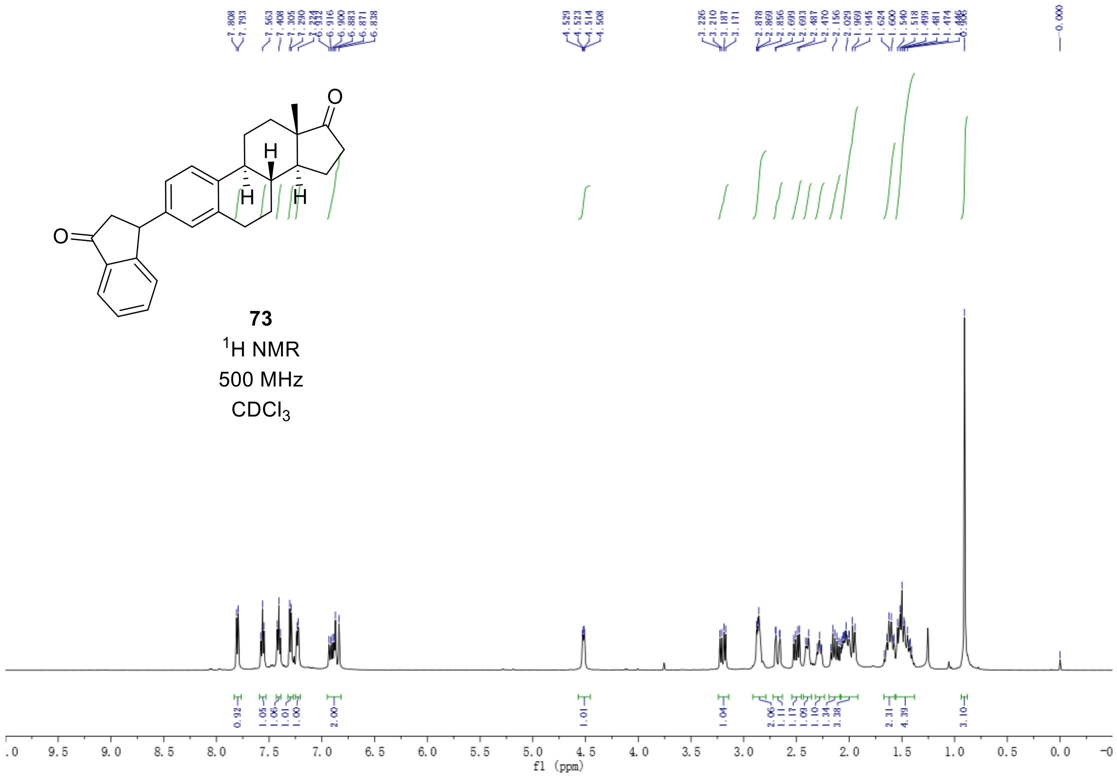

Supplementary Fig. 162 ^{1}H NMR spectrum of compound **73**

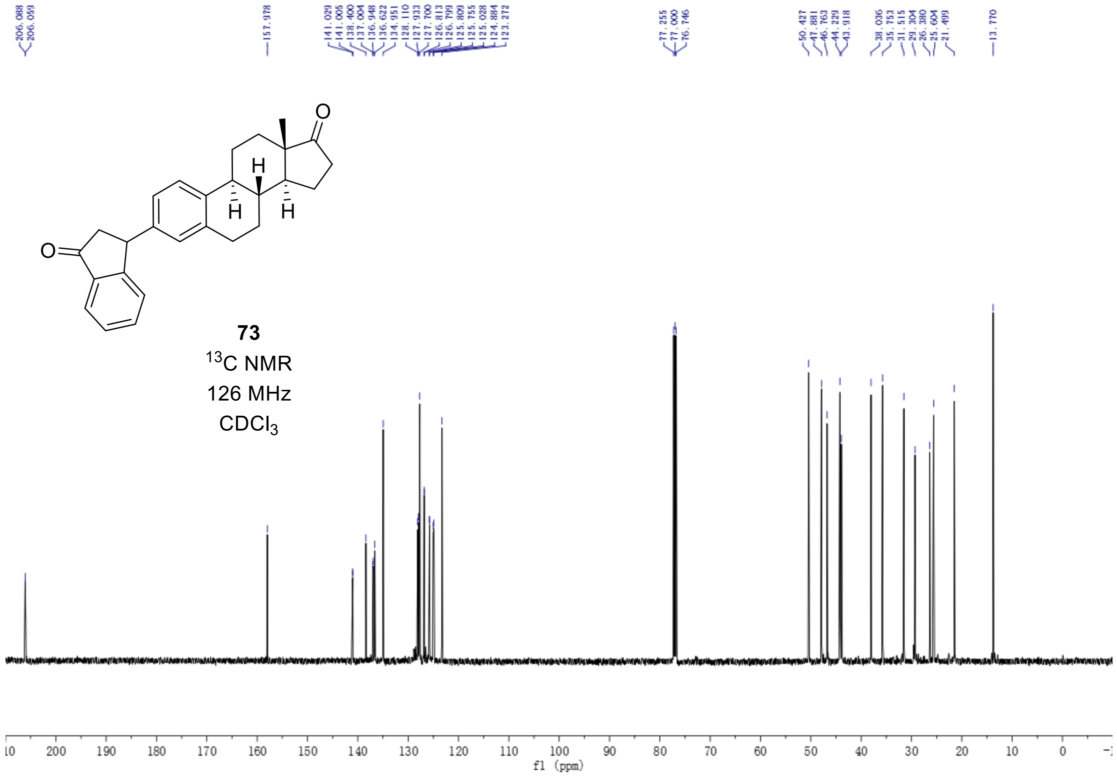

Supplementary Fig. 163 ^{13}C NMR spectrum of compound **73**

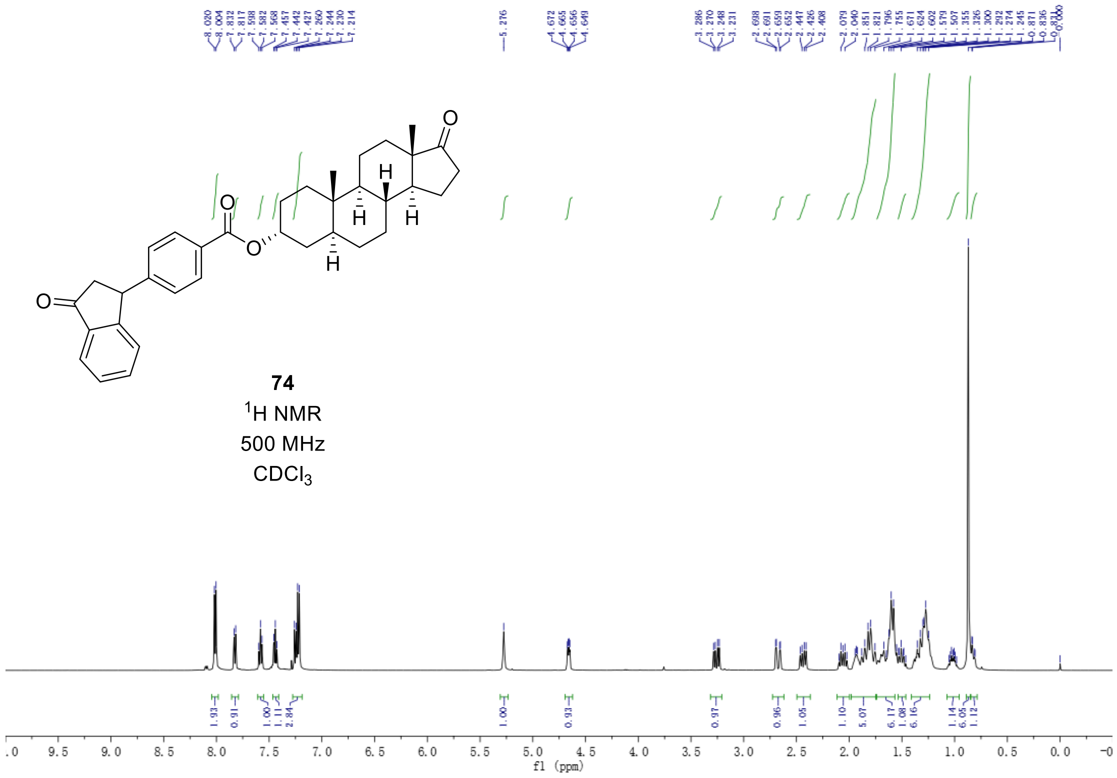

Supplementary Fig. 164 ^{1}H NMR spectrum of compound **74**

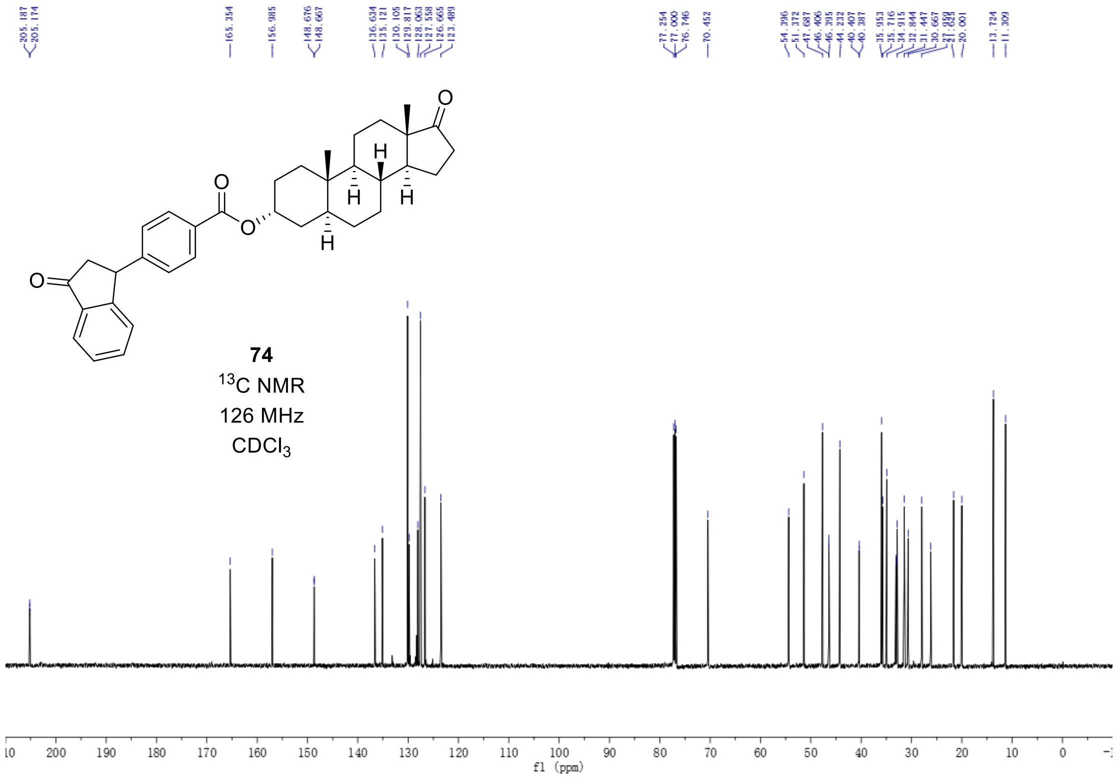

Supplementary Fig. 165 ^{13}C NMR spectrum of compound **74**

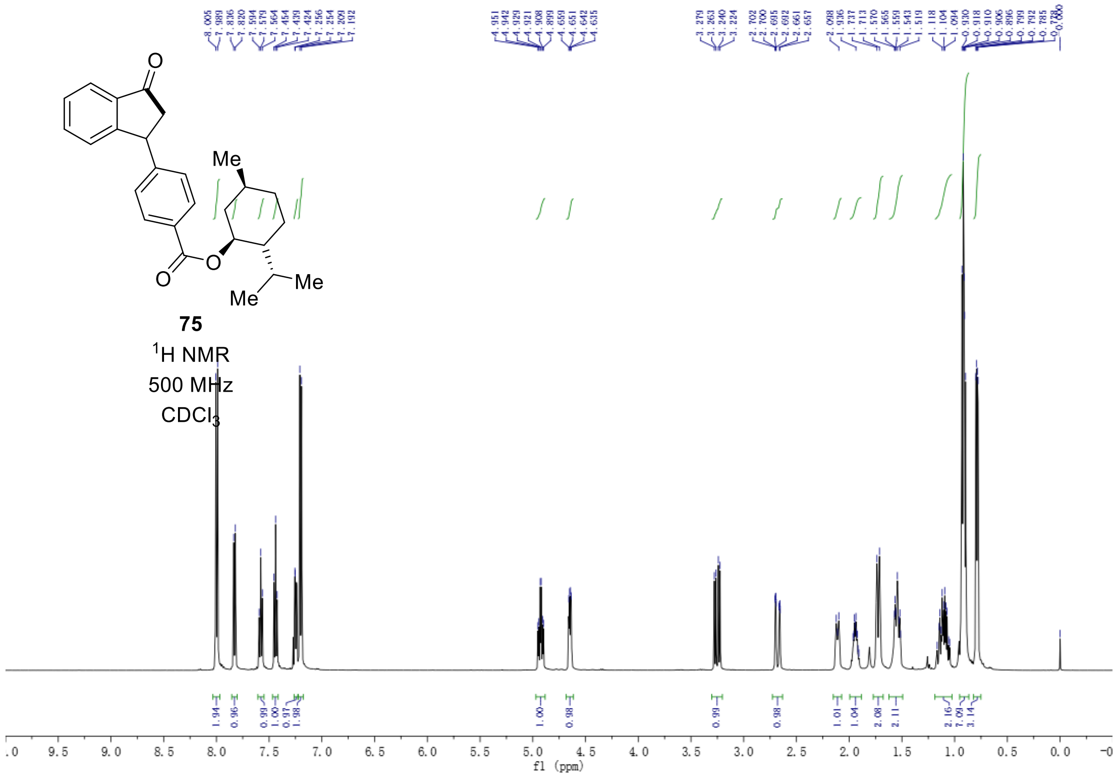

Supplementary Fig. 166 ^{1}H NMR spectrum of compound **75**

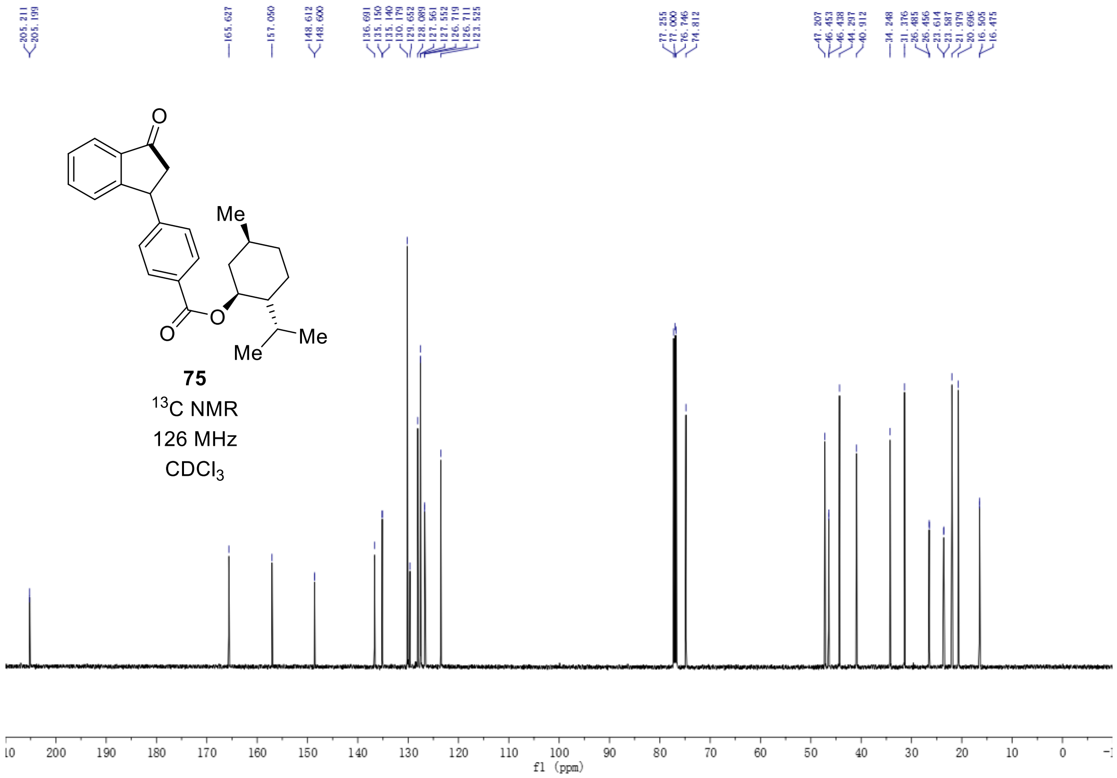

Supplementary Fig. 167 ^{13}C NMR spectrum of compound **75**

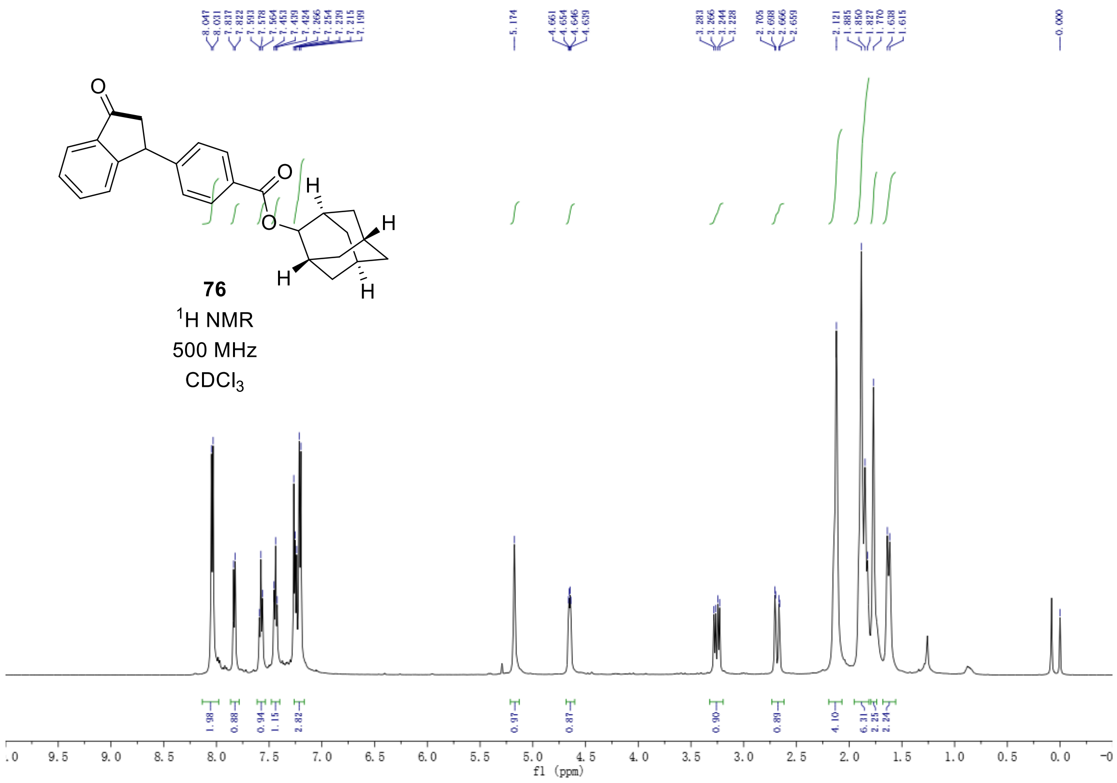

Supplementary Fig. 168 ^{1}H NMR spectrum of compound **76**

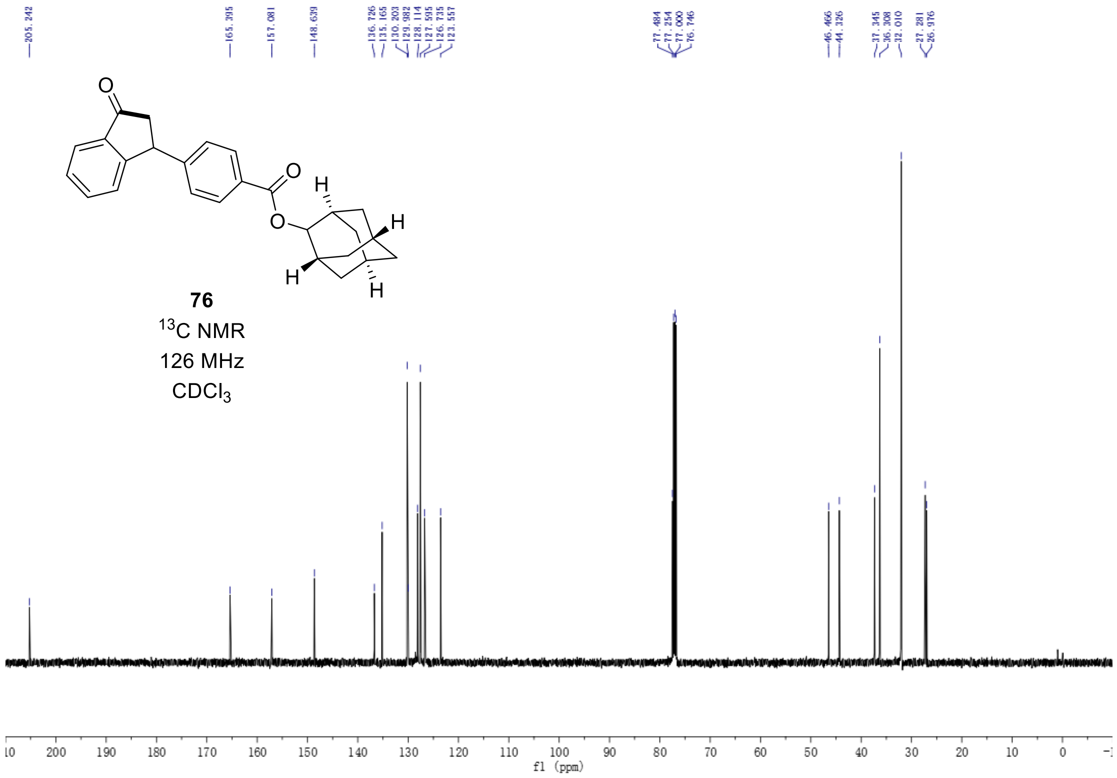

Supplementary Fig. 169 ^{13}C NMR spectrum of compound **76**

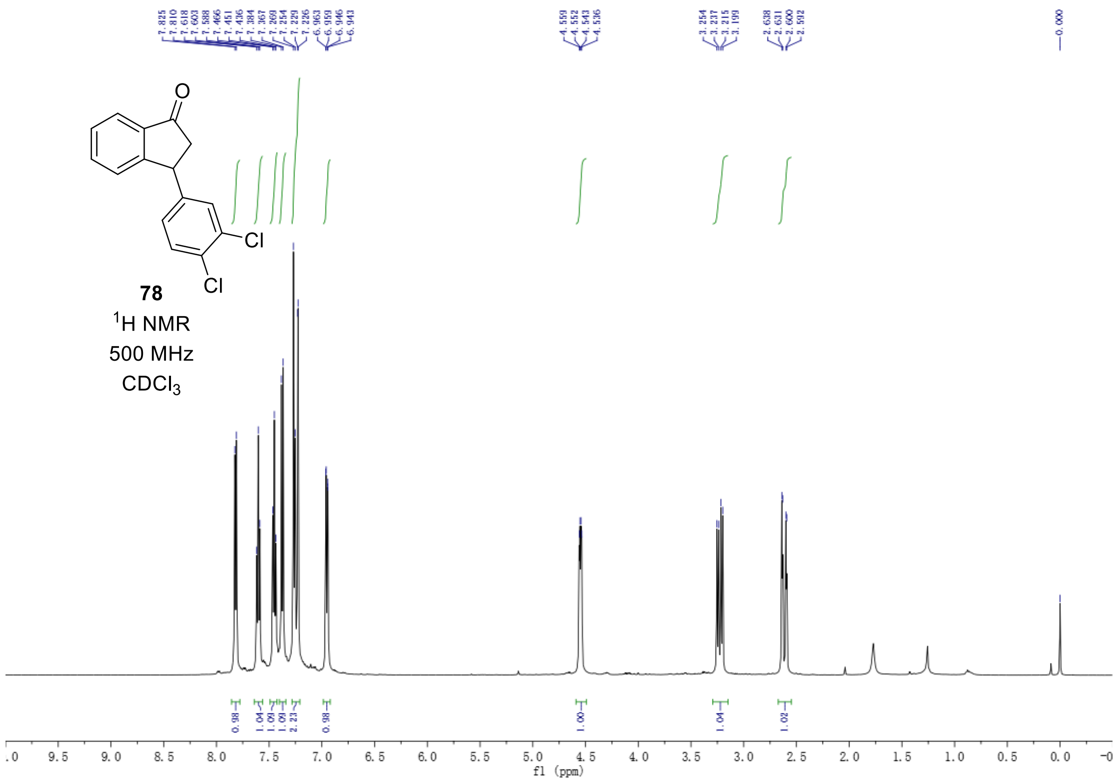

Supplementary Fig. 170 ^{1}H NMR spectrum of compound **78**

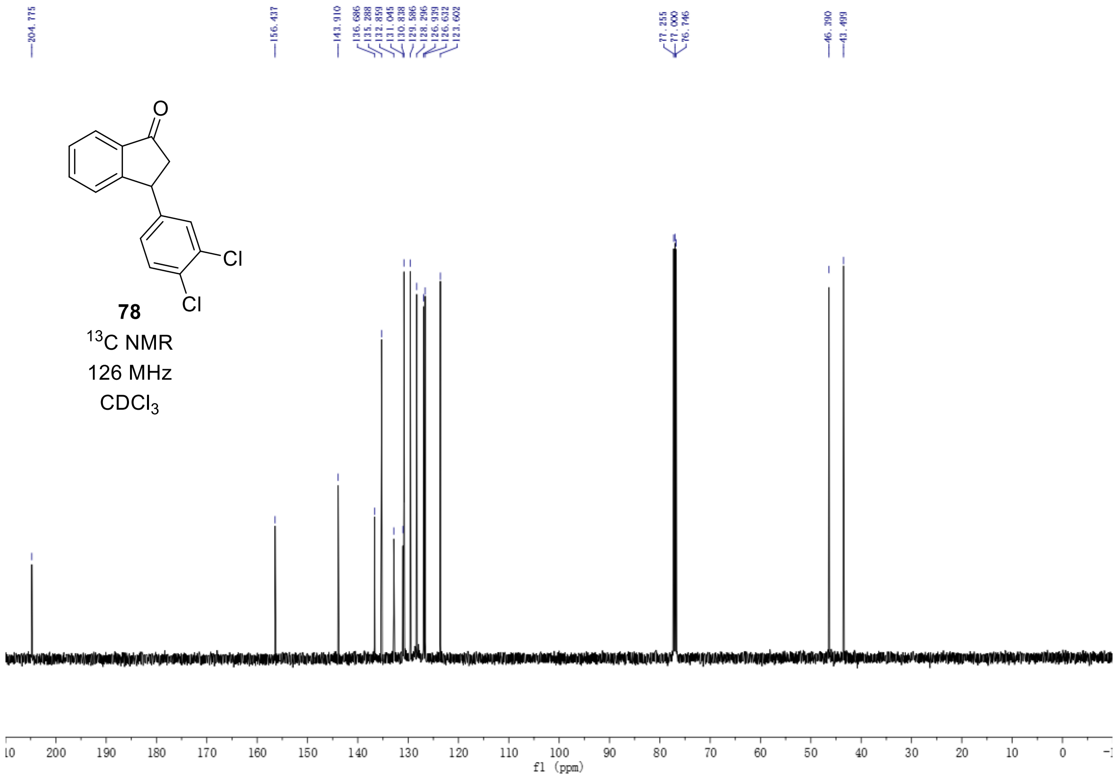

Supplementary Fig. 171 ^{13}C NMR spectrum of compound **78**

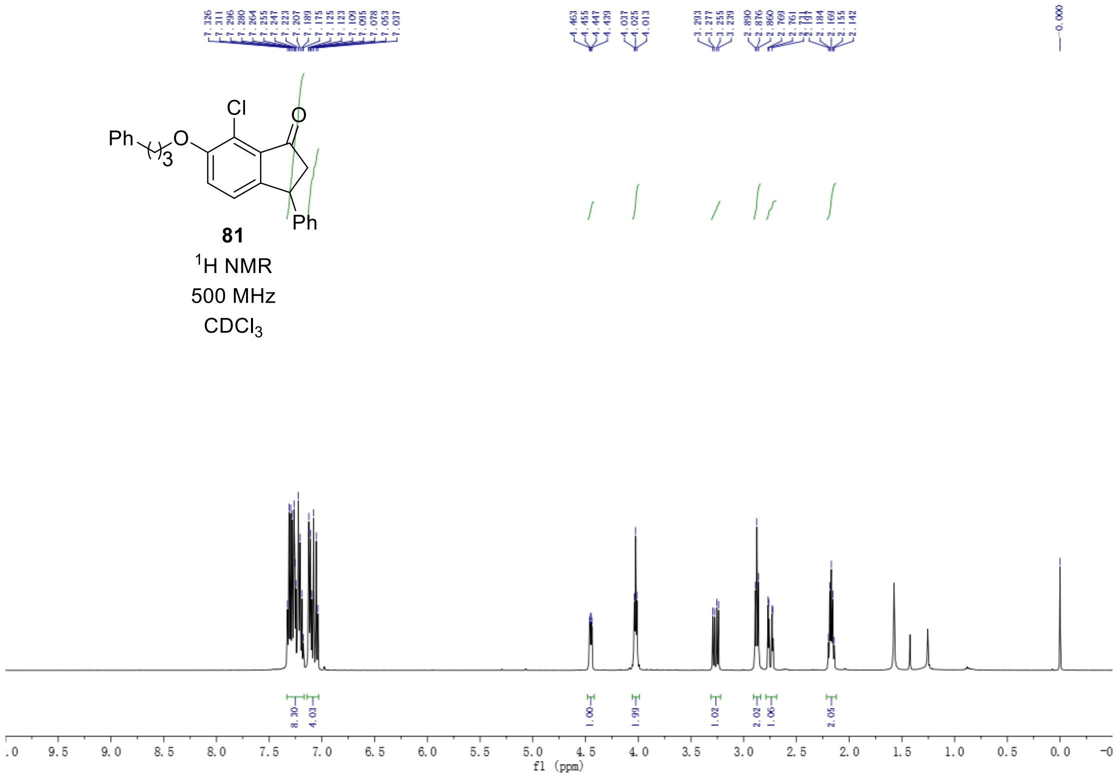

Supplementary Fig. 172 ^{1}H NMR spectrum of compound **81**

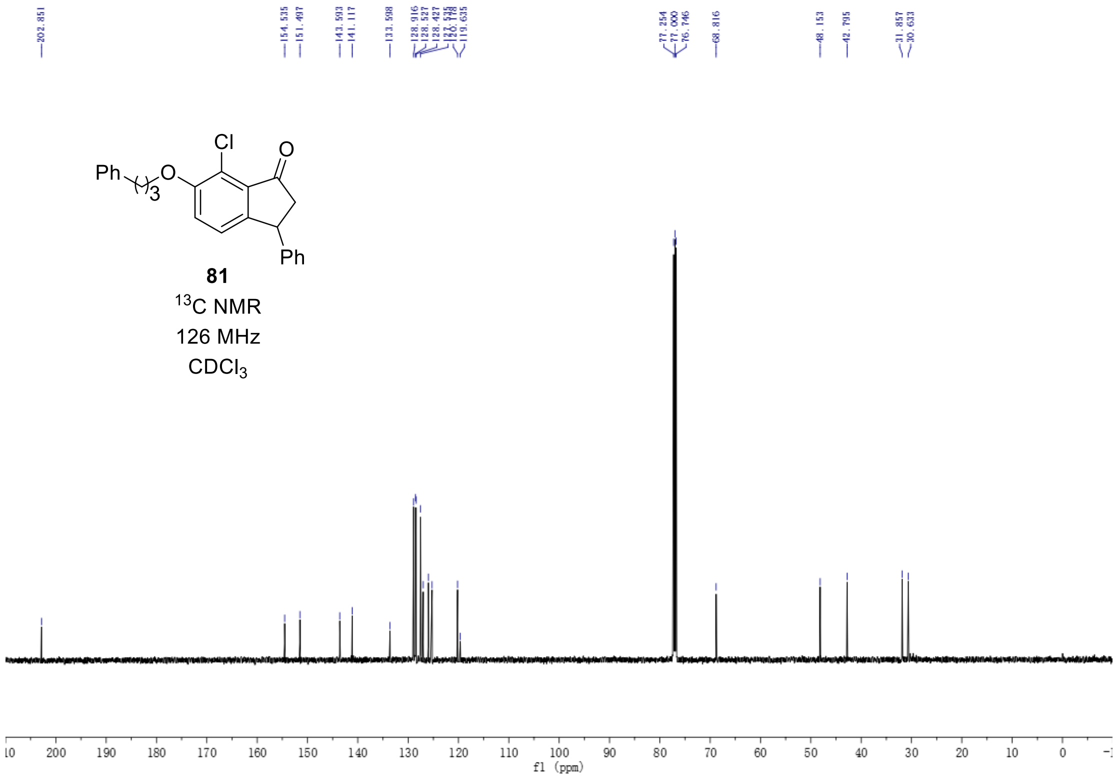

Supplementary Fig. 173 ^{13}C NMR spectrum of compound **81**

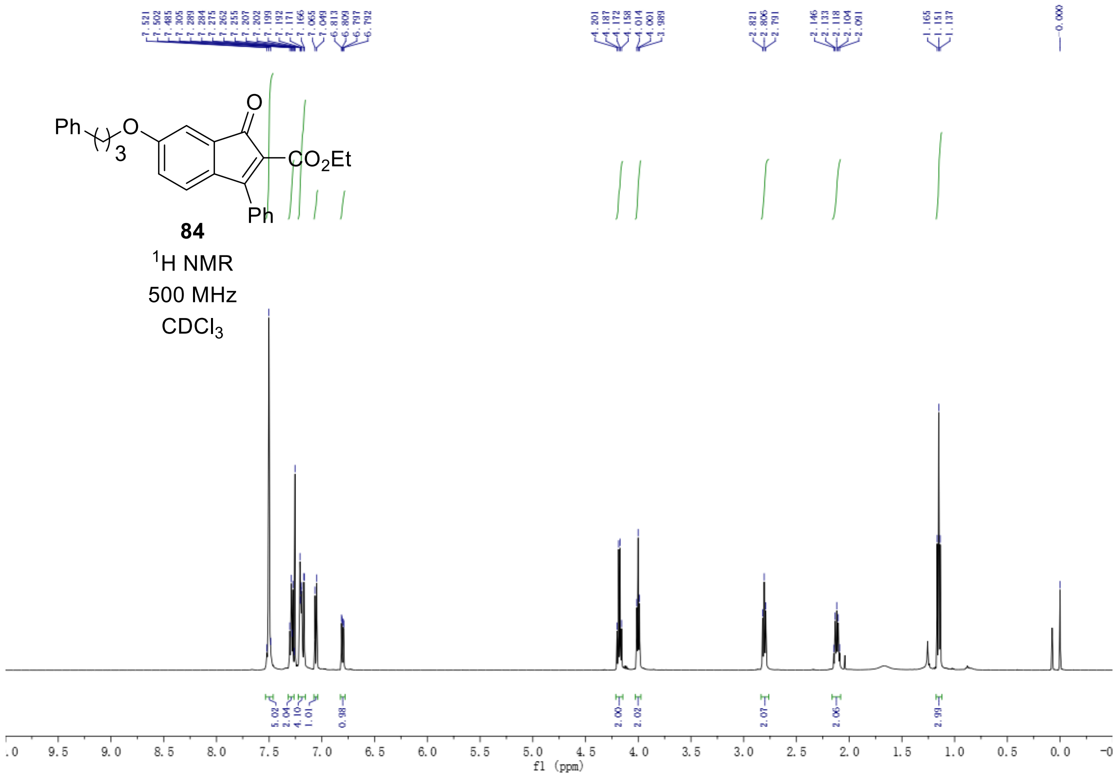

Supplementary Fig. 174 ^{1}H NMR spectrum of compound **84**

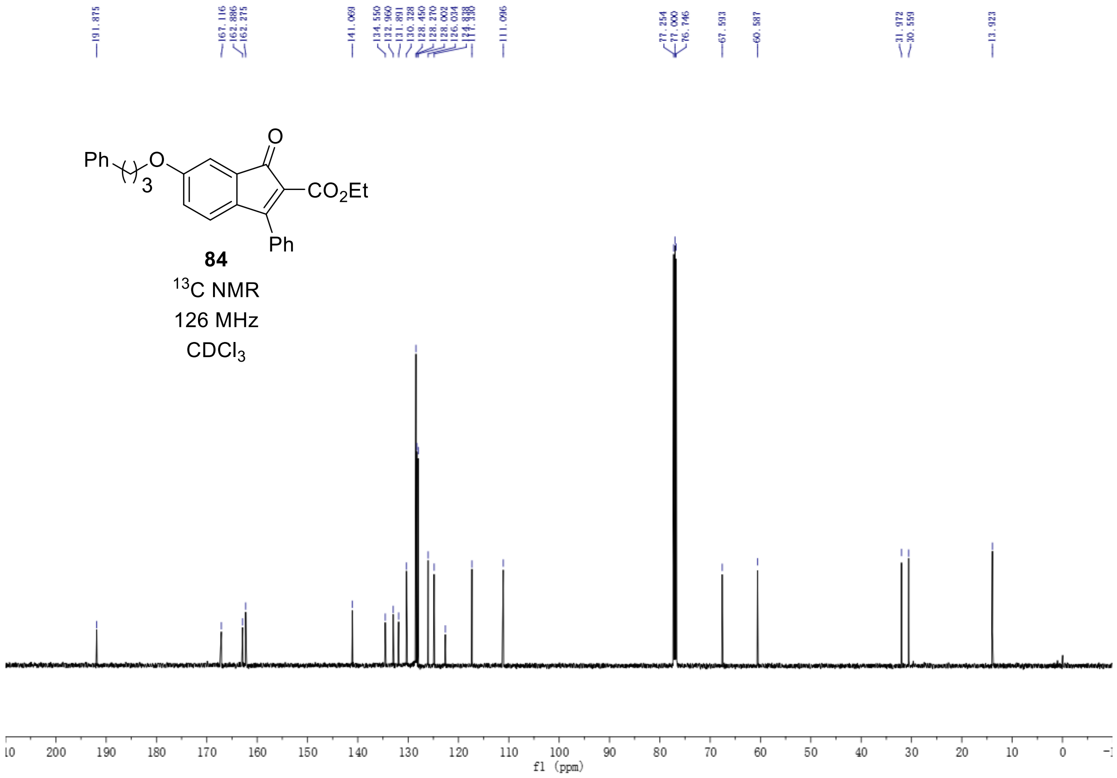

Supplementary Fig. 175 ^{13}C NMR spectrum of compound **84**

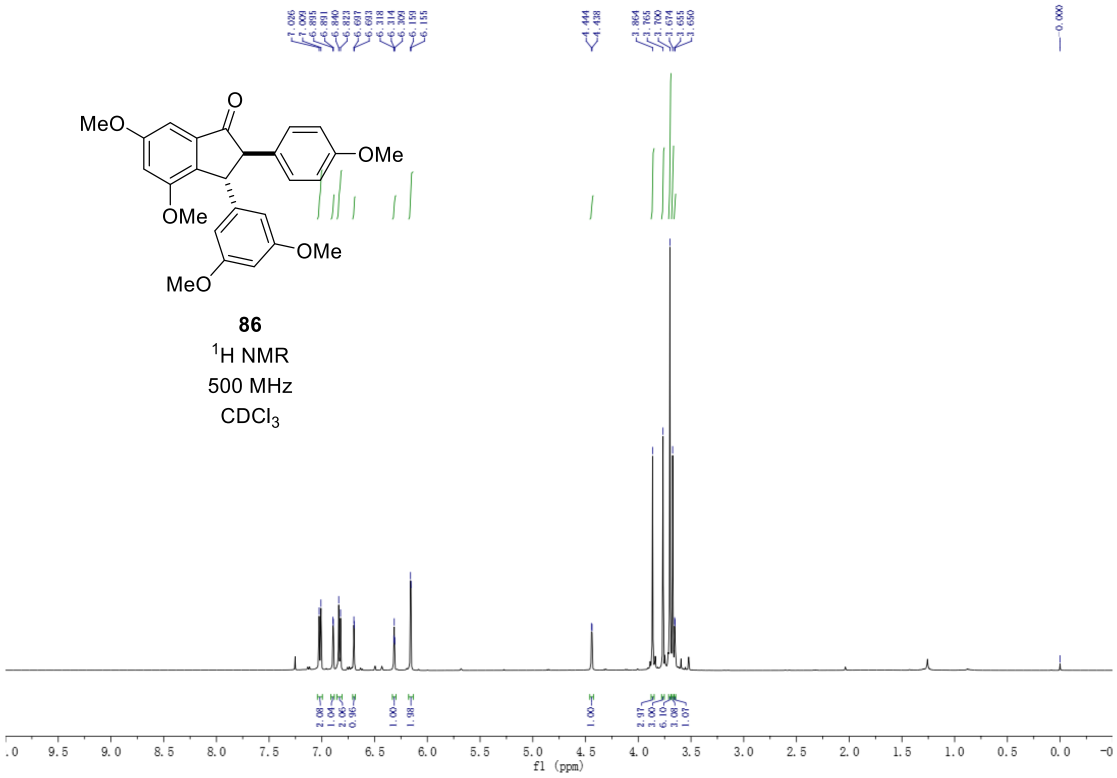

Supplementary Fig. 176 ^{1}H NMR spectrum of compound **86**

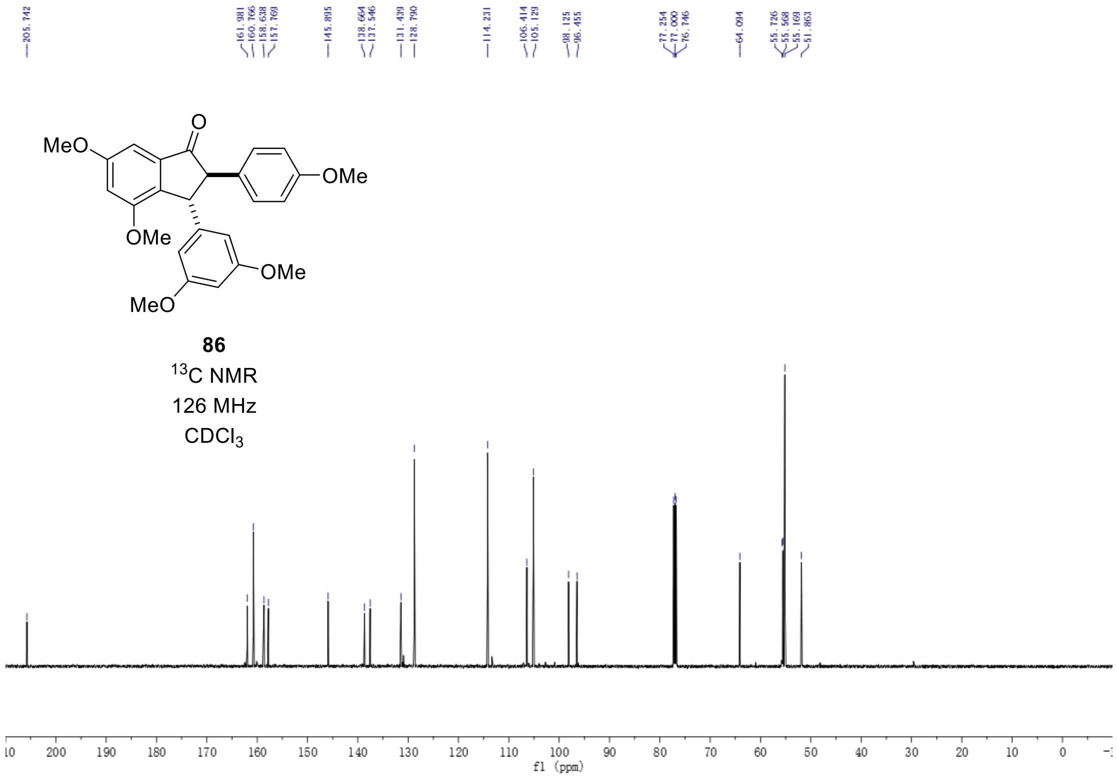

Supplementary Fig. 177 ^{13}C NMR spectrum of compound **86**

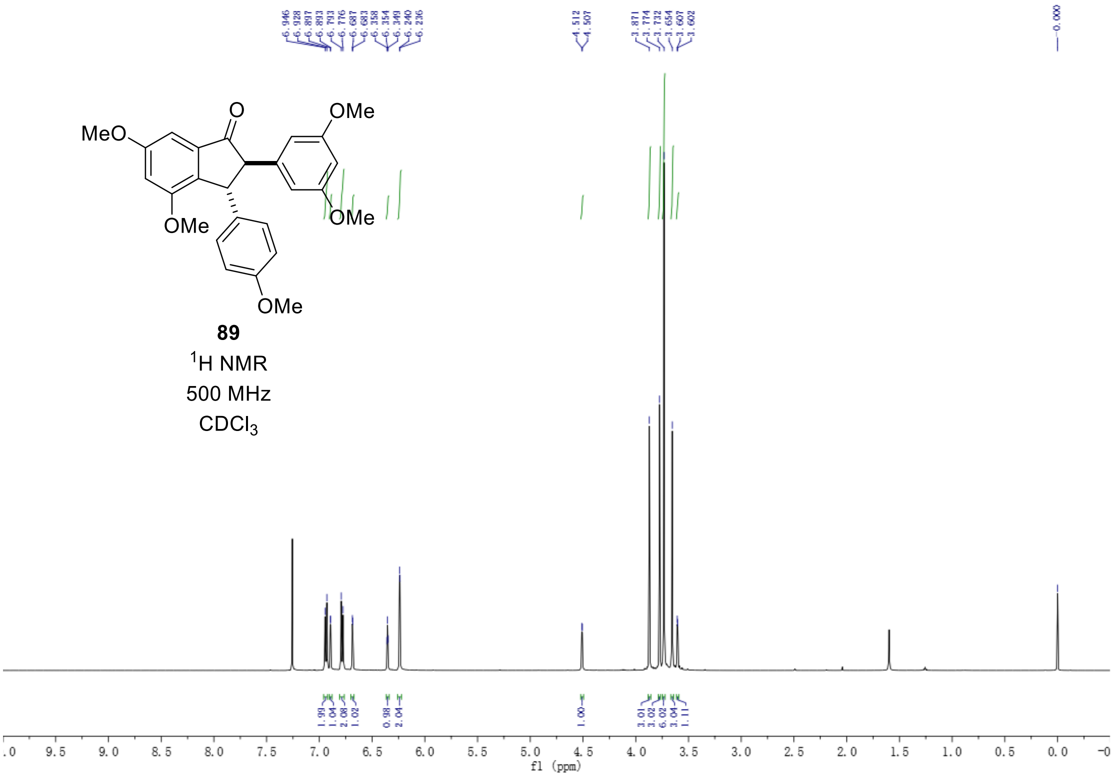

Supplementary Fig. 178 ^{1}H NMR spectrum of compound **89**

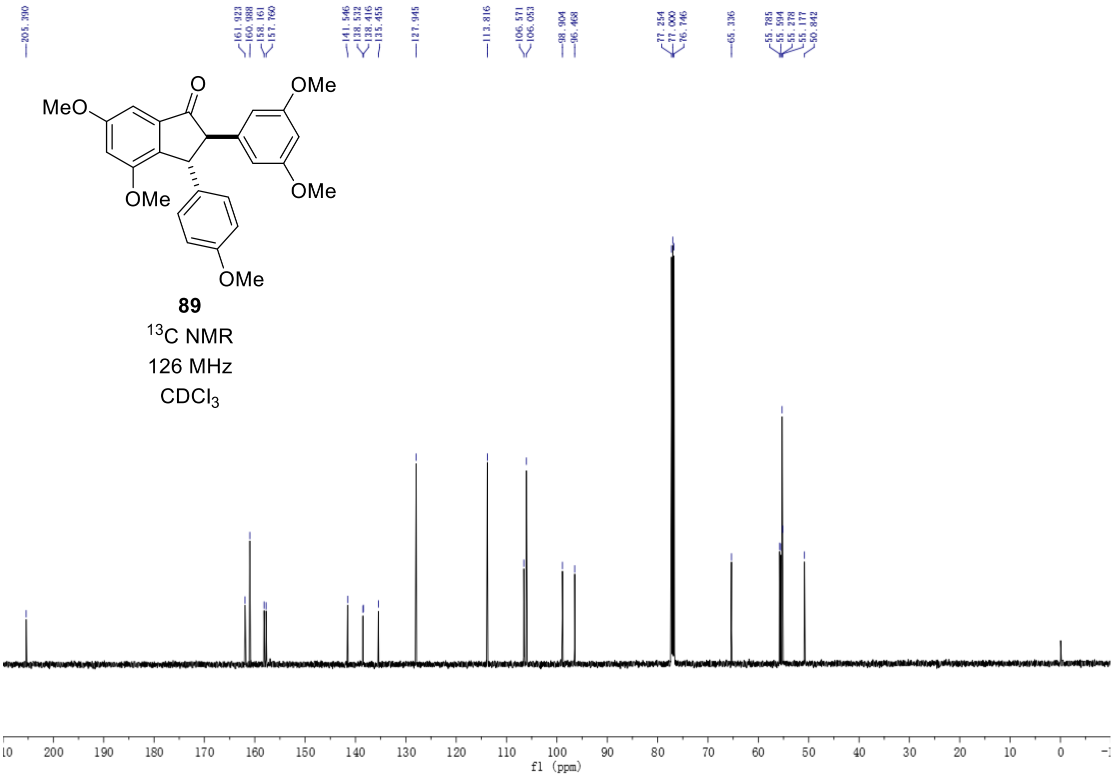

Supplementary Fig. 179 ^{13}C NMR spectrum of compound **89**

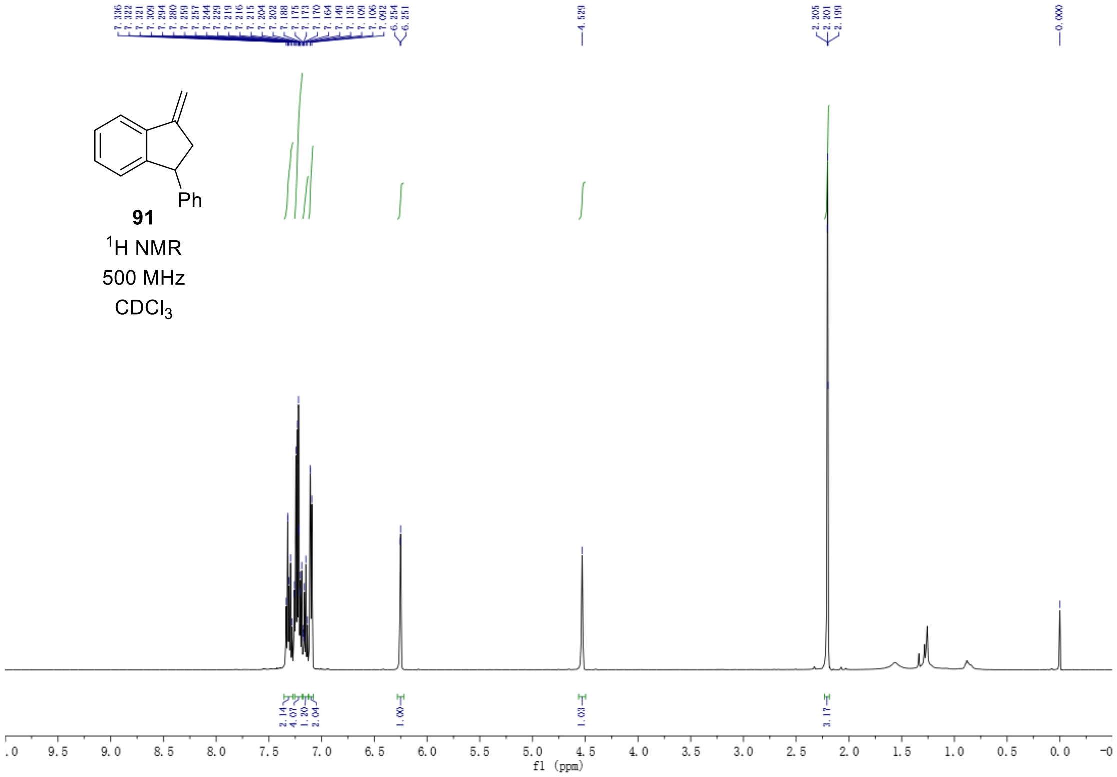

Supplementary Fig. 180 ^{1}H NMR spectrum of compound **91**

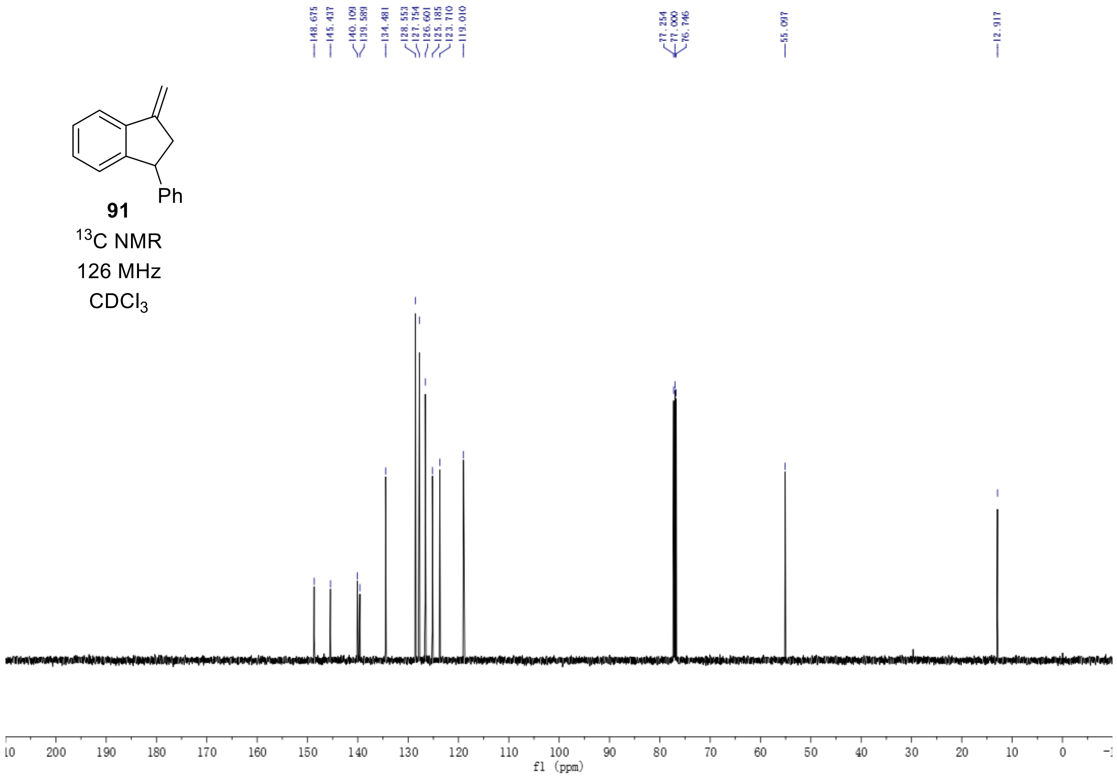

Supplementary Fig. 181 ^{13}C NMR spectrum of compound **91**

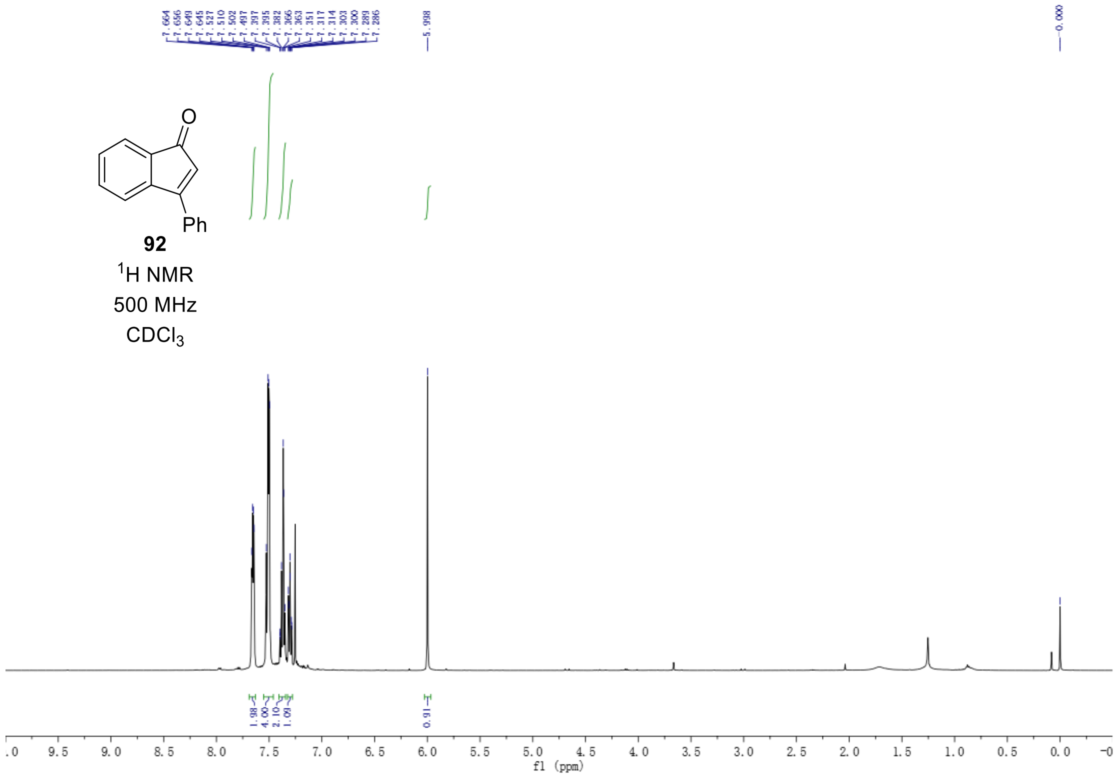

Supplementary Fig. 182 ^{1}H NMR spectrum of compound **72**

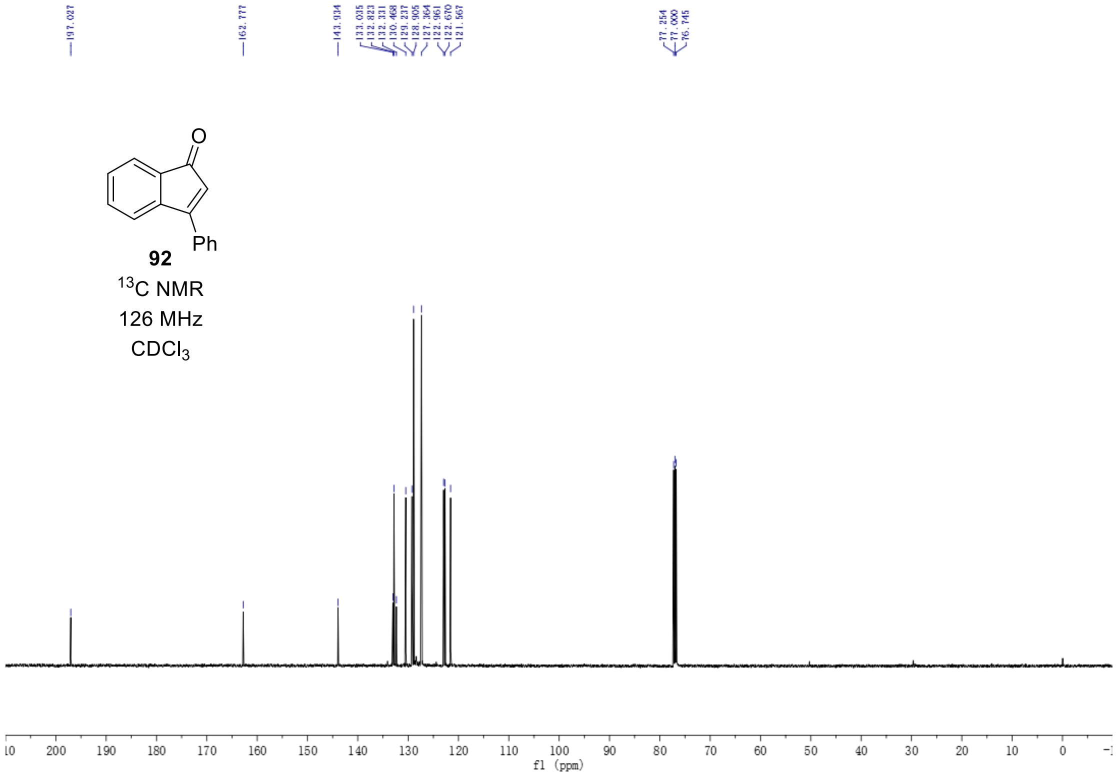

Supplementary Fig. 183 ^{13}C NMR spectrum of compound **92**

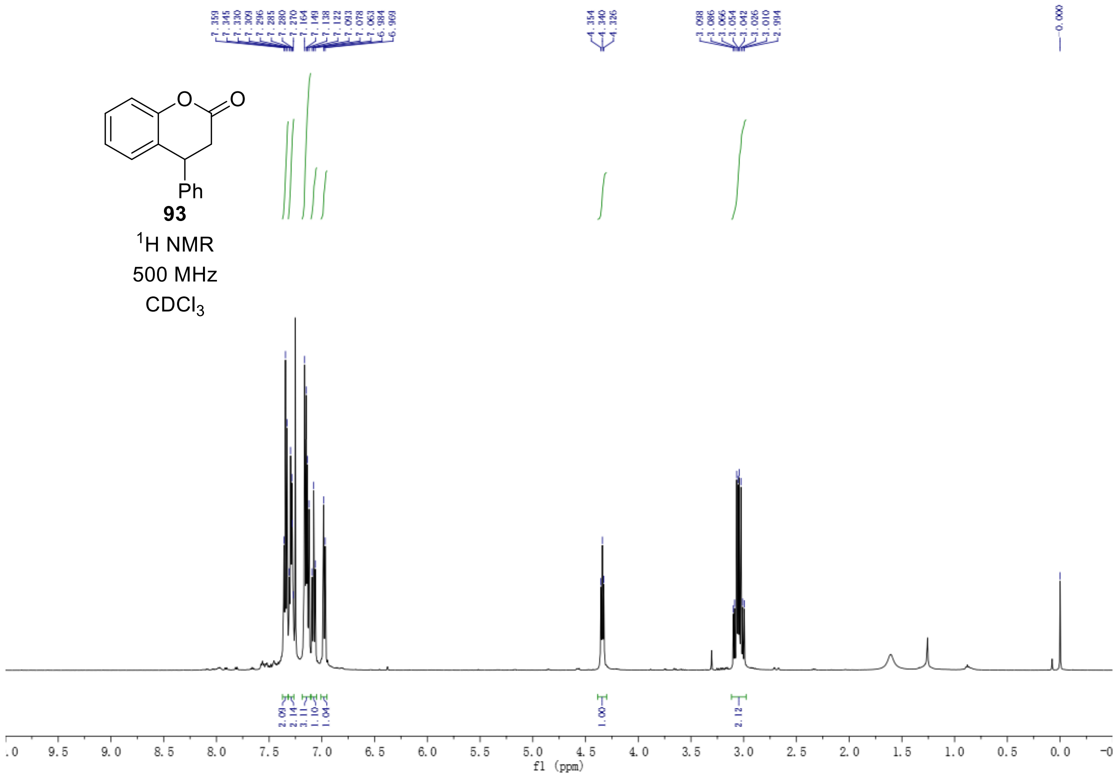

Supplementary Fig. 184 ^{1}H NMR spectrum of compound **93**

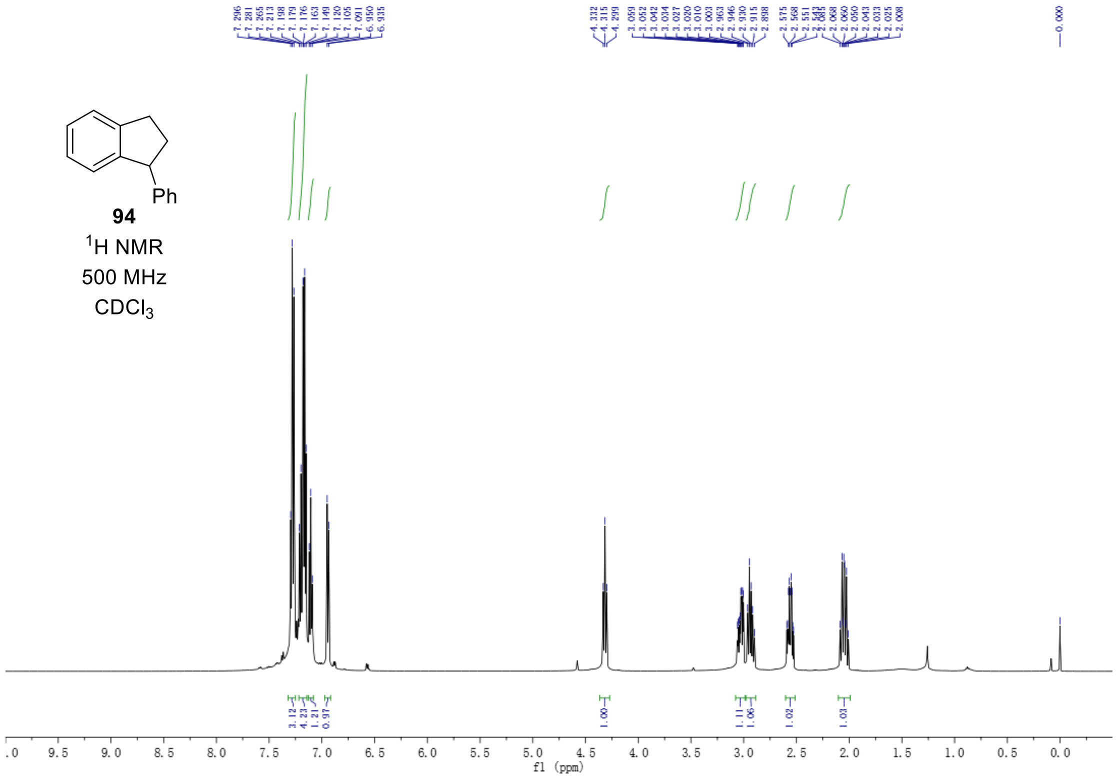

Supplementary Fig. 185 ^{1}H NMR spectrum of compound **94**

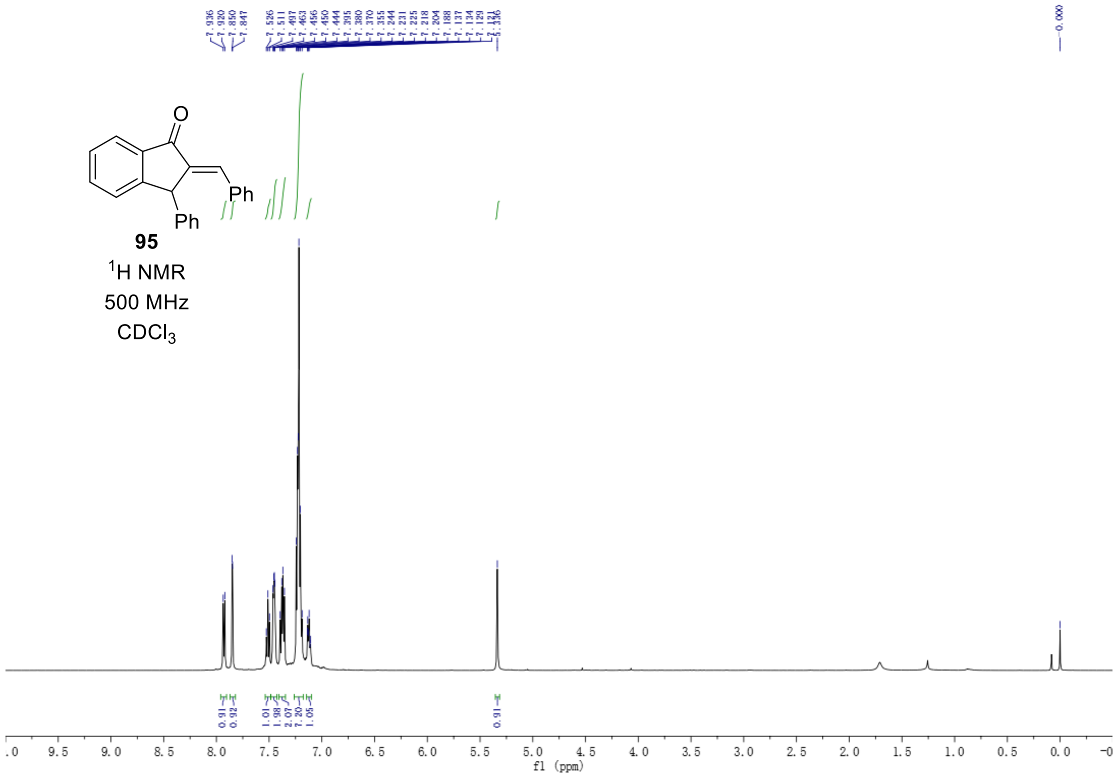

Supplementary Fig. 186 ^{1}H NMR spectrum of compound **95**

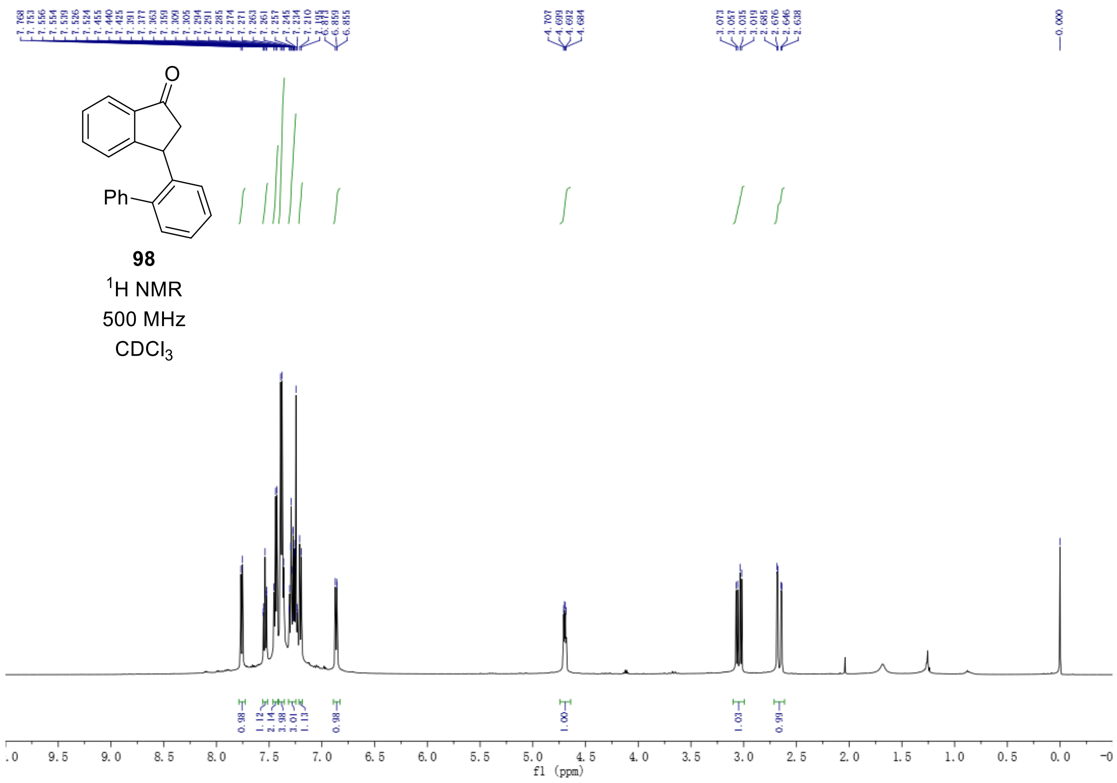

Supplementary Fig. 187 ^{1}H NMR spectrum of compound **98**

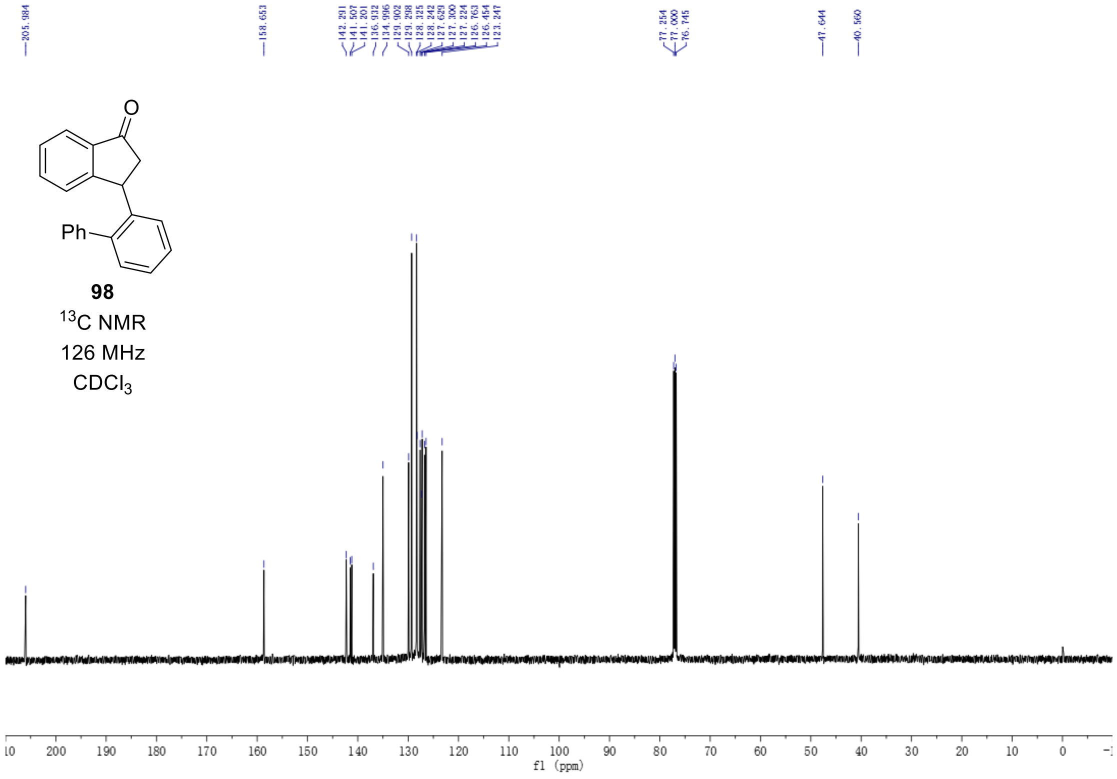

Supplementary Fig. 188 ^{13}C NMR spectrum of compound **98**

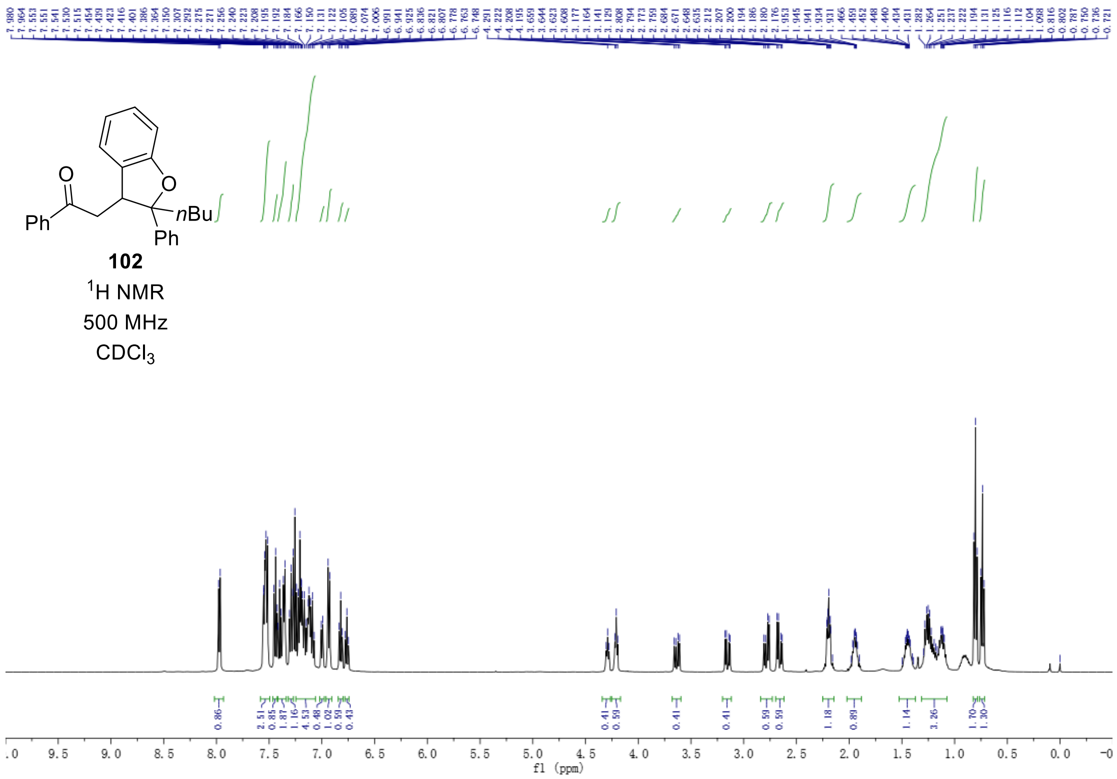

Supplementary Fig. 189 ^{1}H NMR spectrum of compound **102**

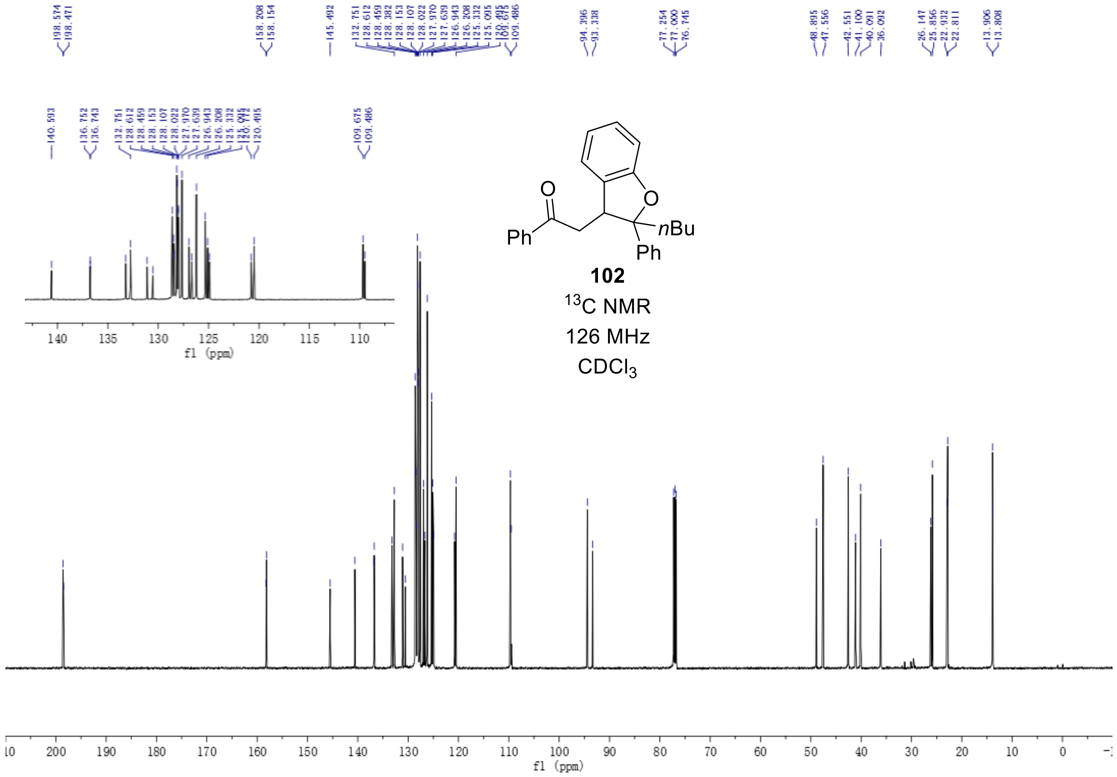

Supplementary Fig. 190 ^{13}C NMR spectrum of compound **102**

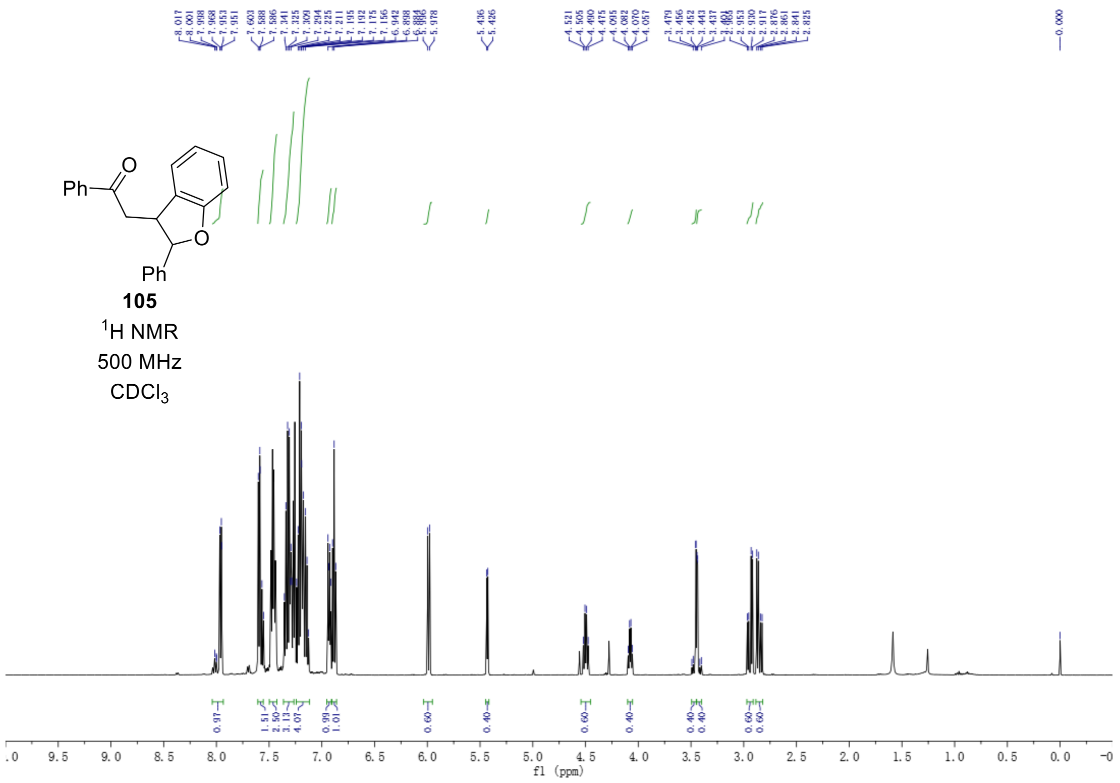

Supplementary Fig. 191 ^{1}H NMR spectrum of compound **105**

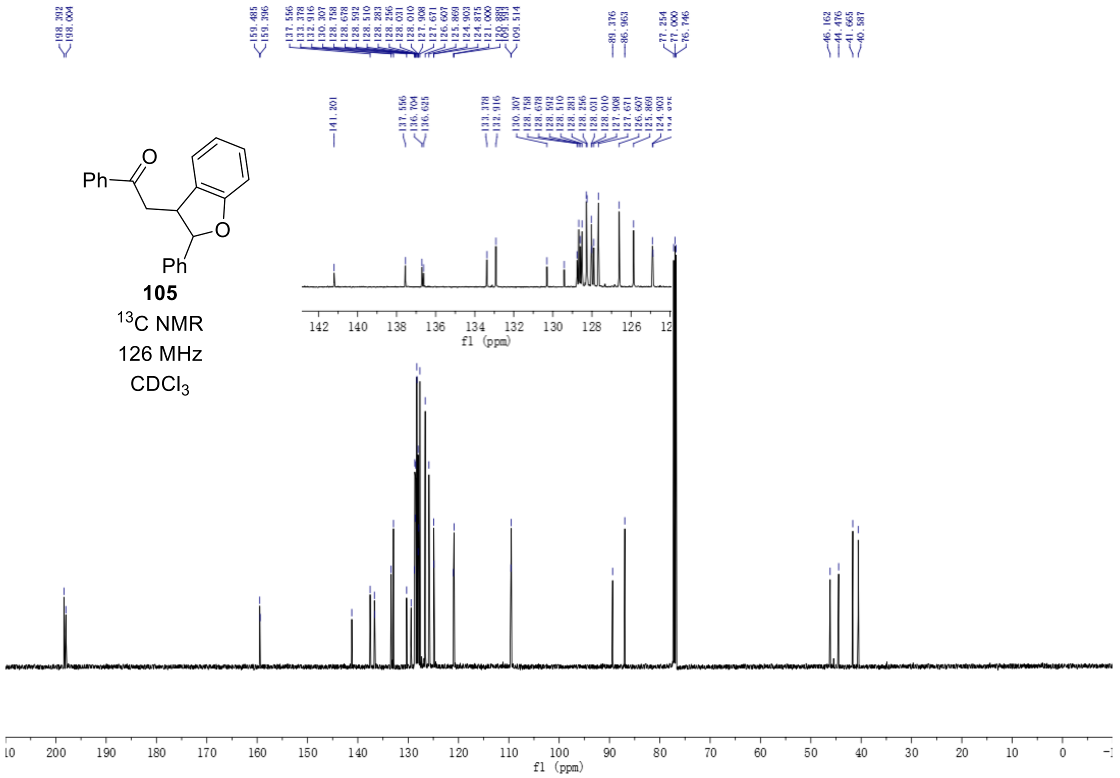

Supplementary Fig. 192 ^{13}C NMR spectrum of compound **105**

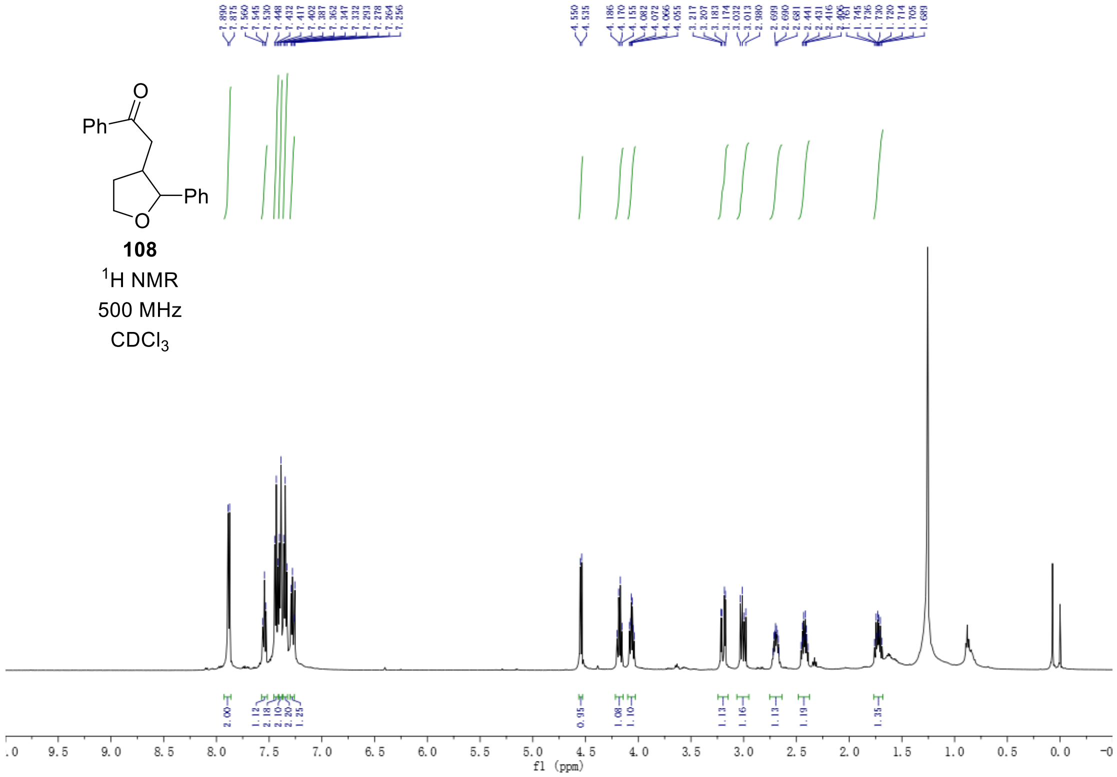

Supplementary Fig. 193 ^{1}H NMR spectrum of compound **108**

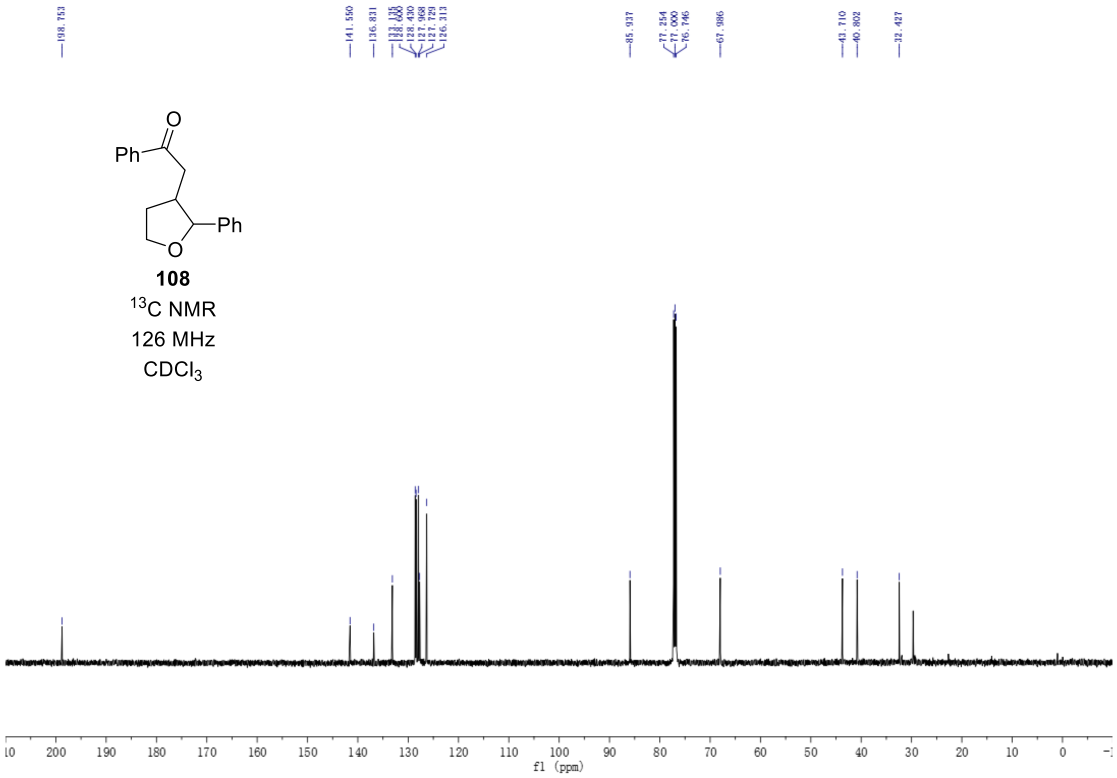

Supplementary Fig. 194 ^{13}C NMR spectrum of compound **108**

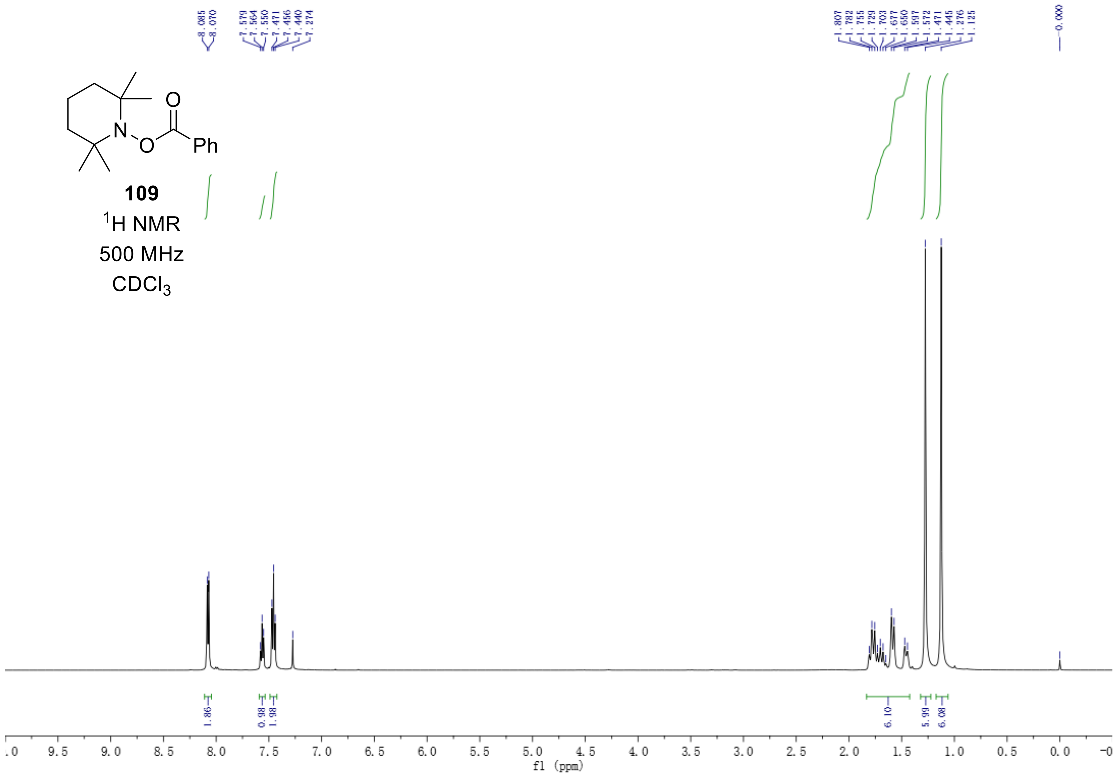

Supplementary Fig. 195 ^{1}H NMR spectrum of compound **109**

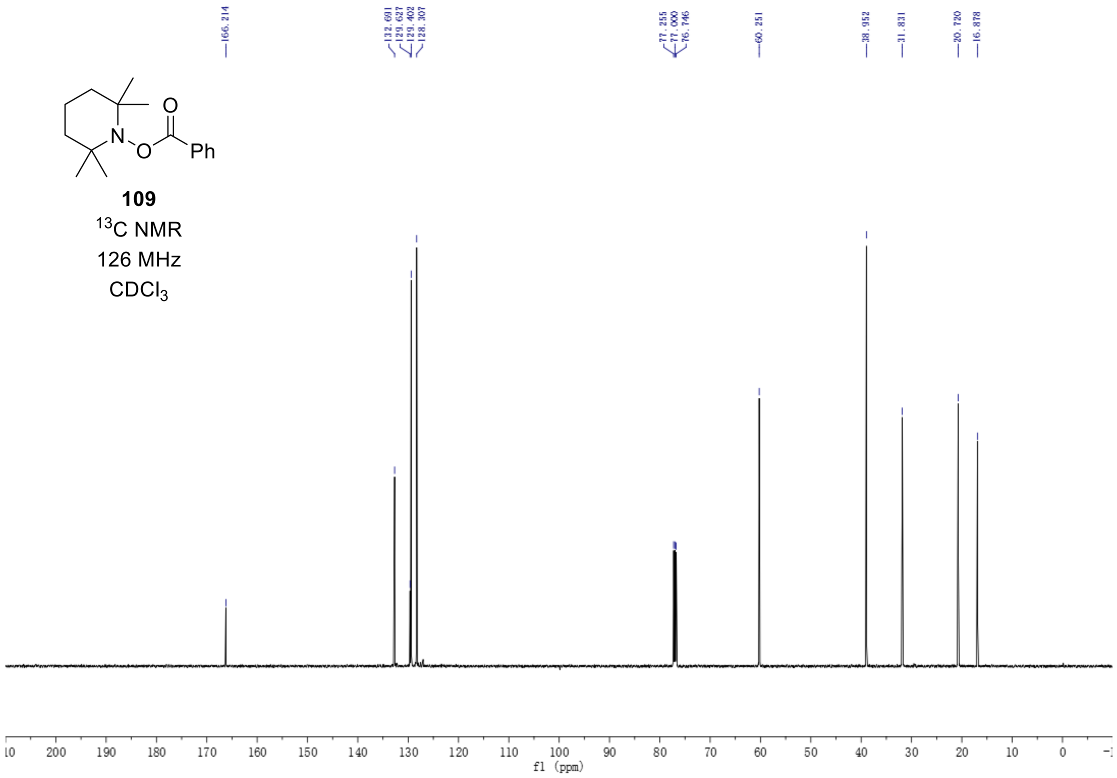

Supplementary Fig. 196 ^{13}C NMR spectrum of compound **109**

Supplementary References

[1] Wadhwa, K.; Yang, C.; West, P. R.; Deming, K. C.; Chemburkar, S. R. & Reddy, R. E. Synthesis of Arylglyoxylic Acids and Their Collision-Induced Dissociation. *Synth. Commun.* **38**, 4434-4444 (2008).

[2] Wang, J.; Peng, Y.-B.; Tao, N.; Zeng, R. & Zhao, Y. Nickel-Catalyzed, *para*-Selective, Radical-Based Alkylation of Aromatic Ketones. *Org. Lett.* **22**, 854-857 (2020).

[3] Prakash, R. & S. Gogoi. Copper-Catalyzed C-N, C-O Coupling Reaction of Arylglyoxylic Acids with Istains. *Adv. Syn. Catal.* **358**, 3046-3049 (2016).

[4] Streit, U.; Birbaum, F.; Quattropani, A. & Bochet, C. G. Photocycloaddition of Arenes and Allenes. *J. Org. Chem*. **78**, 6890-6910 (2013).

[5] Xie, S.; Li, D.; Huang, H.; Zhang, F. & Chen, Y. Intermolecular Radical Addition to Ketoacids Enabled by Boron Activation. *J. Am. Chem. Soc.* **141**, 16237-16242 (2019).

[6] Mo, X.; Morgan, T. D. R.; Ang, H. T. & Hall, D. G. Scope and Mechanism of a True Organocatalytic Beckmann Rearrangement with a Boronic Acid/Perfluoropinacol System Under Ambient Conditions. *J. Am. Chem. Soc.* **140**, 5264-5271(2018).

[7] Ikeda, S.-I.; Watanabe, H. & Sato, Y. Nickel- and Zinc-Promoted [2+2+2] Cycloaddition of Diynes and α, β-Enones. *J. Org. Chem*. **63**, 7026-7029 (1998).

[8] Xiong, X. & Yeung, Y.-Y. Ammonium Salt-Catalyzed Highly Practical *Ortho*-Selective Monohalogenation and Phenylselenation of Phenols: Scope and Applications. *ACS Catal*. **8**, 4033-4043 (2018).

[9] Wang, Z.; Yang, M. & Wang, Y. Ir(III)-Catalyzed Oxidative Annulation of Phenylglyoxylic Acids with Benzo[*b*]thiophenes. *Org. Lett.* **20**, 3001-3005 (2018).

[10] Yang, F.; Rauch, K.; Kettelhoit, K. & L. Ackermann. Aldehyde-Assisted Ruthenium(II)-Catalyzed C-H Oxygenations. *Angew. Chem. Int. Ed.* **53**, 11285-11288 (**2014**).

[11] Nagamochi, M.; Fang, Y.-Q. & Lautens, M. A General and Practical Method of Alkynyl Indole and Benzofuran Synthesis via Tandem Cu- and Pd-Catalyzed Cross-Couplings. *Org. Lett*. **9**,2955-2958 (2007).

[12] Morri, A. K.; Thummala, Y. & Doddi, V. R. The Dual Role of 1,8-Diazabicyclo[5.4.0]undec-7-ene (DBU) in the Synthesis of Terminal Aryl- and Styryl-Acetylenes via Umpolung Reactivity. *Org. Lett*. **17**, 4640-4643 (2015).

[13] Gupta, S.; Koley, D.; Ravikumar, K. & Kundu, B. Counter Ion Effect in Au/Ag-Catalyzed Chemoselective 6-*endo*-*dig* N-and O-Cyclizations of Enyne-Urea System: Diversity-Oriented Synthesis of Annulated Indoles. *J. Org. Chem.* **78**, 8624-8633 (2013).

[14] Zhang, Z.; Liu, B.; Wang, C.-Y. & B.-F. Shi. Cobalt (III)-Catalyzed C2-Selective C–H Alkynylation of Indoles. *Org, Lett*. **17**, 4094-4097 (2015).

[15] Zhang, Z. & Jiang. X. Oxidative Coupling of Terminal Alkyne with α-Hydroxy Ketone: An

Expedient Approach toward Ynediones. *Org. Lett*. **16**, 4400-4403 (2014).

[16] Bestmann, H. J. & Frey, H. Neue Aufbaumoglichkeiten fur 1-Bromacetylene und aromatische sowie konjugierte Enine. *Liebigs Annalen der Chemie*. **12**, 2061-2071 (1980).

[17] Thummala, Y.; Karunakar, G. V. & Doddi, V. R. DBU-Mediated Synthesis of Aryl Acetylenes or 1-Bromoethynylarenes from Aldehydes. *Adv. Syn. Catal*. **361**, 611-616 (2019).

[18] Naveen, K.; Nikson, S. A. & Perumal, P. T. Palladium-Catalyzed Synthesis of Tetrasubstituted Olefins by Triple Domino Process. *Adv. Syn. Catal*. **359**, 2407-2413 (2017).

[19] Mori, K.; Kawasaki, T.; Sueoka, S. & Akiyama, T. Expeditious Synthesis of Benzopyrans via Lewis Acid-Catalyzed C−H Functionalization: Remarkable Enhancement of Reactivity by an *Ortho* Substituent. *Org. Lett*. **12**, 1732-1735 (2010).

[20] Shen, H.; Fu, J.; Yuan, H.; Gong, J. & Yang, Z. Synthesis of 2,3-Disubstituted Indoles and Benzofurans by the Tandem Reaction of Rhodium(II)-Catalyzed Intramolecular C–H Insertion and Oxygen-Mediated Oxidation. *J. Org. Chem*. **81**, 10180-10192 (2016).

[21] Yue, G.; Lei, K.; Hirao, H. & Zhou, J. Palladium-Catalyzed Asymmetric Reductive Heck Reaction of Aryl Halides. *Angew. Chem. Int. Ed*. **54**, 6531-6535 (2015).

[22] Zhang, G.; Hu, Z.; Bertoli, G. & Gooßen, L. J. Iridium-Catalyzed Synthesis of Substituted Indanones from Aromatic Carboxylates and Unsaturated Ketones. *ACS Catal*. **9**, 8153-8158 (2019).

[23] C.-D. Wang, Y.-F. Hsieh, R.-S. Liu. *Adv. Syn. Catal*. **2014**, *356*, 144.

[24] Gorbunova, Y.; Zakusilo, D. N. & Vasilyev, A. V. Cinnamonitrile as a precursor of a bi-centered electrophile in reactions with arenes in triflic acid. *Tetrahedron Lett*. **60**, 961-964 (2019).

[25] Wei, Y.; Ma, Y.; Xu, J. & Y. Zhao. Microwave-Assisted One-Pot Synthesis of 1-Indanones from Arenes and α,β-Unsaturated Acyl Chlorides. *J. Org. Chem*. **71**, 4312-4315 (2006).

[26] Harmata, M.; Barnes, C. L.; Brackley, J.; Bohnert, G.; Kirchhoefer, P.; Kürti, L. & Rashatasakhon, P. Generation of Cyclopentadienones from 2-Bromocyclopentenones. *J .Org. Chem*. **66**, 5232-5236 (2001).

[27] Shotter, R. G. &. Johnston, K. M. Polyphosphoic acid-catalyzed cyclisations of aryl styryl ketones. *Tetrahedron* **29**, 2163-2166 (1973).

[28] Qin, X.; Lee, M. W. Y. & Zhou, J. Asymmetric Hydroarylation of Enones via Nickel-Catalyzed 5-*Endo-Trig* Cyclization. *Org. Lett*. **21**, 5990-5994 (2019).

[29] Parveen, N. & Sekar, G. Palladium Nanoparticles-Catalyzed Synthesis of Indanone Derivatives via Intramolecular Reductive Heck Reaction. *Adv. Synth. Catal.* **361**, 4581-4595 (2019).

[30] Yu, Y.-N. & Xu, M.-H. Enantioselective Synthesis of Chiral 3-Aryl-1-indanones through Rhodium-Catalyzed Asymmetric Intramolecular 1,4-Addition. *J. Org. Chem*. **78**, 2736-2741 (2013).

[31] Koltunov, K. Y.; Walspurger, S. & Sommer, J. Cyclization of 1-phenyl-2-propen-1-ones into 1-indanones using H-zeolite and other solid acids. The role of mono- and dicationic intermediate. *Tetrahedron Letters*. **46**, 8391-8394 (2005).

[32] Gao, D. & Li, Y. Identification and preliminary structure–activity relationships of 1-Indanone

derivatives as novel indoleamine-2,3-dioxygenase 1 (IDO1) inhibitors. *Bioorgan. Med. Chem*. **25**, 3780-3791 (2017).

[33] Jongcharoenkamol, J.; Chuathong, P.; Amako, Y.; Kono, M.; Poonswat, K.; Ruchirawat, S. & Ploypradith, P. Selective Divergent Synthesis of Indanols, Indanones, and Indenes via Acid-Mediated Cyclization of (*Z*)- and (*E*)-(2-Stilbenyl)methanols and Its Application for the Synthesis of Paucifloral F Derivatives. *J. Org. Chem.* **83**, 13184-13210 (2018).

[34] Negishi, E.-C.; Coperet, C.; Ma, S.; Mita, T.; Sugihara, T. & Tour, J. M. Palladium-Catalyzed Carbonylative Cyclization of 1-Iodo-2-alkenylbenzenes. *J. Am. Chem. Soc*. **118**, 5904-5918 (1996).

[35] Lee, B. H.; Choi, Y. L.; Shin, S. & Heo, J.-N.Stereoselective Palladium-Catalyzed α-Arylation of 3-Aryl-1-Indanones: An Asymmetric Synthesis of (+)-Pauciflorol F. *J. Org. Chem*. **76**, 6611-6618 (2011).

[36] OHTA, S.; Yamashita, M.; Arita, K.; Kajiura, T.; Kawasaki, I.; Noda, K. & Izumi, M. Conversion of ketone trimethylsilylcyanohydrins to several types of compounds. *Chem. Pharm. Bull*. **43**, 1294-1301 (1995).

[37] Dethe, D. H. & Murhade, G. M. $FeCl_3$ mediated synthesis of substituted indenones by a formal [2+2] cycloaddition/ring opening cascade of *o*-keto-cinnamates. *Chem. Commun*. **51**, 10891-10894 (2015).

[38] Zhou, X.; Zhao, Y.; Cao, Y. & He, L. Catalytic Efficient Nazarov Reaction of Unactivated Aryl Vinyl Ketones via a Bidentate Diiron Lewis Acid Activation Strategy. *Adv. Syn. Catal.* **359**, 3325-3331 (2017).

[39] Tanaka, S.; Kunisawa, T.; Yoshii, Y. & Hattori, T. Acylation of Alkenes with the Aid of $AlCl_3$ and 2,6-Dibromopyridine. *Org. Lett.* **21**, 8509-8513 (2019).

[40] Parveen, N. & Sekar, G. A Simple Transformation of 1-(Isoxazol-3-yl)ureas to 5-(2-oxoalkyl)-2,4-dihydro-3H-1,2,4-triazol-3-ones through Base-Promoted Boulton-Katritzky Rearrangement. *Adv. Syn. Catal*. **361**, 481-484 (2019).

[41] Wang, G.-Z.; Shang, R.; Cheng, W.-M. & Fu, Y. Decarboxylative 1,4-Addition of α-Oxocarboxylic Acids with Michael Acceptors Enabled by Photoredox Catalysis. *Org. Lett*. **17**, 4830-4833 (2015).

[42] Zheng, H.; Xiao, Z.-F.; Yao, C.-Z.; Li, Q.-Q.; Ning, X.-S.; Kang, Y.-B. & Tang, Y. Transition-Metal-Free Self-Hydrogen-Transferring Allylic Isomerization. *Org. Lett*. **17**, 6102-6105 (2015).

[43] Frisch, M. J.; Trucks, G. W.; Schlegel, H. B.; Scuseria, G. E.; Robb, M. A.; Cheeseman, J. R.; Scalmani, G.; Barone, V.; Petersson, G. A.; Nakatsuji, H.; Li, X.; Caricato, M.; Marenich, A. V.; Bloino, J.; Janesko, B. G.; Gomperts, R.; Mennucci, B.; Hratchian, H. P.; Ortiz, J. V.; Izmaylov, A. F.; Sonnenberg, J. L.; Williams-Young, D.; Ding, F.; Lipparini, F.; Egidi, F.; Goings, J.; Peng, B.; Petrone, A.; Henderson, T.; Ranasinghe, D.; Zakrzewski, V. G.; Gao, J.; Rega, N.; Zheng, G.; Liang, W.; Hada, M.; Ehara, M.; Toyota, K.; Fukuda, R.; Hasegawa, J.; Ishida, M.; Nakajima, T.; Honda,

Y.; Kitao, O.; Nakai, H.; Vreven, T.; Throssell, K.; Montgomery Jr., J. A.; Peralta, J. E.; Ogliaro, F.; Bearpark, M. J.; Heyd, J. J.; Brothers, E. N.; Kudin, K. N.; Staroverov, V. N.; Keith, T. A.; Kobayashi, R.; Normand, J.; Raghavachari, K.; Rendell, A. P.; Burant, J. C.; Iyengar, S. S.; Tomasi, J.; Cossi, M.; Millam, J. M.; Klene, M.; Adamo, C.; Cammi, R.; Ochterski, J. W.; Martin, R. L.; Morokuma, K.; Farkas, O.; Foresman, J. B. & Fox, D. J. *Gaussian16*, Gaussian, Inc., Wallingford, CT, **2016**.

[44] Zhao, Y. & Truhlar, D. G. "The M06 suite of density functionals for main group thermochemistry, thermochemical kinetics, noncovalent interactions, excited states, and transition elements: two new functionals and systematic testing of four M06-class functionals and 12 other functionals," *Theor. Chem. Acc*. **120**, 215-241 (2008).

[45] Furche, F & Ahlrichs, R. Adiabatic time-dependent density functional methods for excited state properties. *J. Chem. Phys.* **117**, 7433-7447 (2002).

[46] Scalmani, G. & Frisch, M. J. Geometries and properties of excited states in the gas phase and in solution: Theory and application of a time-dependent density functional theory polarizable continuum model. *J. Chem. Phys*. **124**, 094107:1-15 (2006).

[47] Fang, D.-C. THERMO; Beijing Normal University, Beijing, People's Republic of China, 2013.
