# Supplementary material for: Hydrogen radical-shuttle (HRS)-enabled photoredox synthesis of indanones via decarboxylative annulation
Source: Nat Commun. 2021 Sep 6;12:5257. doi: 10.1038/s41467-021-25594-4 (PMC8421331; doi:10.1038/s41467-021-25594-4)
Supplement: Supplementary file 2 — Description of Additional Supplementary Files [file 41467_2021_25594_MOESM2_ESM.docx]

Description of Additional Supplementary Files

Title: Supplementary Data 1

Description: The coordination for all the structures involved in the computational calculations
