# Supplementary material for: Hydrogen radical-shuttle (HRS)-enabled photoredox synthesis of indanones via decarboxylative annulation
Source: Nat Commun. 2021 Sep 6;12:5257. doi: 10.1038/s41467-021-25594-4 (PMC8421331; doi:10.1038/s41467-021-25594-4)
Supplement: Supplementary file 3 — Supplementary Data 1 [file 41467_2021_25594_MOESM3_ESM.zip › Supplementary Data 1/Supplementary Data 1.docx]

**Supplementary Data 1**. The coordination for all the structures involved in the calculation.

| **I**  Ir -0.82584 0.00375 -0.00387  C 2.14871 -0.02902 0.72397  C 3.32344 -0.00088 1.47381  C 3.27808 -0.05770 2.86685  C 2.01017 -0.13609 3.45489  C 0.87998 -0.14928 2.65660  C 2.14185 0.01638 -0.76541  C 3.30129 -0.01940 -1.52865  C 3.24156 0.03009 -2.92686  C 1.97295 0.11089 -3.50000  C 0.84989 0.13505 -2.68270  H 4.27616 0.07403 0.96830  H 1.88803 -0.18391 4.53114  H -0.11648 -0.20648 3.08470  H 4.26410 -0.09469 -1.03824  H 1.83291 0.15263 -4.57239  H -0.15053 0.19577 -3.10150  N 0.94279 -0.09894 1.31924  N 0.92579 0.09085 -1.35046  C 4.53038 -0.03530 3.73815  C 4.56601 -1.32020 4.58429  C 5.81171 0.04199 2.90340  C 4.46586 1.19191 4.66435  H 3.69346 -1.39896 5.23850  H 4.59870 -2.20742 3.94517  H 5.45990 -1.31624 5.21490  H 5.84882 0.95604 2.30250  H 6.67546 0.04927 3.57322  H 5.91315 -0.82114 2.23792  H 5.35648 1.21606 5.29914  H 4.43182 2.11761 4.08234  H 3.58852 1.16271 5.31627  C 4.52955 -0.00433 -3.74422  C 5.39066 1.21207 -3.36071  C 5.29174 -1.30146 -3.42190  C 4.24977 0.04273 -5.24890  H 4.86376 2.14590 -3.57800  H 5.65439 1.20488 -2.29942  H 6.31937 1.19777 -3.93875  H 4.69054 -2.17971 -3.67441  H 6.21455 -1.33535 -4.00836  H 5.56423 -1.36282 -2.36464  H 5.19925 0.01629 -5.79000  H 3.65416 -0.81541 -5.57538  H 3.72781 0.96124 -5.53431  C -2.53537 1.25887 1.95605  C -2.24299 -0.01437 1.39651  C -2.91665 -1.13667 1.88356  C -3.84427 -0.98460 2.90466  C -4.14842 0.24105 3.47935  C -3.47926 1.34405 2.98380  C -1.79545 2.39098 1.39345  C -0.16525 2.95130 -0.19646  C -0.26155 4.29528 0.11681  C -1.15879 4.69532 1.10374  C -1.92840 3.74027 1.74464  H -2.73803 -2.13105 1.48717  H -4.87427 0.33649 4.27618  H 0.50954 2.59246 -0.96596  H -1.25933 5.74422 1.36177  H -2.63287 4.03098 2.50887  N -0.91109 2.03046 0.42291  C 0.62782 5.29011 -0.56814  F 0.00194 6.45989 -0.74528  F 1.02651 4.84897 -1.76902  F 1.73302 5.53898 0.14959  F -3.76668 2.53316 3.53780  F -4.48015 -2.06916 3.36546  C -2.57340 -1.23713 -1.93899  C -2.26096 0.03408 -1.38577  C -2.92962 1.16220 -1.86620  C -3.87228 1.01756 -2.87464  C -4.19605 -0.20597 -3.44301  C -3.53125 -1.31464 -2.95428  C -1.83732 -2.37573 -1.38446  C -0.19019 -2.94903 0.18336  C -0.30472 -4.29279 -0.12514  C -1.22054 -4.68589 -1.09749  C -1.98972 -3.72446 -1.72962  H -2.73559 2.15551 -1.47442  H -4.93278 -0.29578 -4.23048  H 0.49908 -2.59516 0.94236  H -1.33608 -5.73440 -1.35065  H -2.70895 -4.00992 -2.48207  N -0.93521 -2.02214 -0.42763  C 0.58581 -5.29375 0.54940  F -0.03957 -6.46500 0.71757  F 0.98661 -4.86269 1.75336  F 1.69007 -5.53589 -0.17202  F -3.83661 -2.50173 -3.50288  F -4.50342 2.10758 -3.32905 | **II**  Ir -0.88099 -0.14109 -0.00103  C 2.05664 0.19391 0.69862  C 3.24163 0.21694 1.49021  C 3.21730 -0.05265 2.83407  C 1.94712 -0.35703 3.41854  C 0.82926 -0.37984 2.62871  C 2.01583 0.48632 -0.69066  C 3.15051 0.82516 -1.47589  C 3.05270 1.09362 -2.82108  C 1.75761 1.01824 -3.40745  C 0.68058 0.69349 -2.61910  H 4.17676 0.45801 1.00213  H 1.84654 -0.57645 4.47449  H -0.14142 -0.62070 3.05149  H 4.11884 0.86946 -0.98988  H 1.59062 1.21474 -4.45755  H -0.31589 0.64087 -3.04714  N 0.85165 -0.12844 1.29805  N 0.77188 0.43727 -1.29681  C 4.46206 -0.03978 3.71485  C 4.63869 -1.42941 4.35166  C 5.72780 0.29903 2.92313  C 4.27745 1.01104 4.82360  H 3.77376 -1.70888 4.95948  H 4.77602 -2.19341 3.58024  H 5.52116 -1.43162 4.99933  H 5.66115 1.29031 2.46357  H 6.58886 0.29895 3.59742  H 5.91759 -0.43754 2.13606  H 5.16083 1.03005 5.46979  H 4.14401 2.00804 4.39264  H 3.40761 0.78899 5.44802  C 4.30191 1.45639 -3.62742  C 4.93010 2.73057 -3.03864  C 5.31279 0.30051 -3.54080  C 3.97538 1.71031 -5.10272  H 4.22834 3.56861 -3.09226  H 5.21597 2.59039 -1.99235  H 5.82942 2.99768 -3.60292  H 4.88422 -0.62172 -3.94498  H 6.20989 0.54554 -4.11845  H 5.61766 0.10955 -2.50793  H 4.89427 1.96544 -5.63827  H 3.54484 0.82386 -5.57901  H 3.27567 2.54338 -5.22277  C -2.65604 0.54091 2.15296  C -2.16260 -0.59416 1.45017  C -2.57449 -1.88012 1.80967  C -3.48612 -2.02199 2.84625  C -3.99886 -0.93893 3.54663  C -3.57164 0.32901 3.18192  C -2.14803 1.84912 1.72331  C -0.70179 2.86734 0.17955  C -0.98152 4.12138 0.69501  C -1.87617 4.23598 1.75384  C -2.46289 3.09300 2.27480  H -2.21096 -2.76619 1.30051  H -4.71133 -1.07265 4.35105  H -0.01940 2.71973 -0.65081  H -2.11477 5.21005 2.16750  H -3.15791 3.16027 3.09787  N -1.27870 1.77213 0.68110  C -0.28935 5.33255 0.13698  F -1.10821 6.39044 0.12512  F 0.13428 5.11968 -1.11449  F 0.77951 5.66548 0.87231  F -4.07436 1.36404 3.86238  F -3.89239 -3.24444 3.18940  C -2.24024 -1.36942 -2.21579  C -2.19179 -0.14013 -1.50133  C -3.00756 0.92878 -1.87899  C -3.88056 0.75613 -2.94376  C -3.96935 -0.43280 -3.65453  C -3.14347 -1.47880 -3.27202  C -1.32493 -2.42613 -1.76747  C 0.32093 -2.90656 -0.16347  C 0.51694 -4.17037 -0.69504  C -0.23596 -4.56847 -1.79470  C -1.16321 -3.69125 -2.33687  H -2.98532 1.88230 -1.36276  H -4.66032 -0.54704 -4.48046  H 0.87480 -2.54698 0.69765  H -0.10308 -5.55658 -2.22186  H -1.75530 -3.97969 -3.19175  N -0.57949 -2.07017 -0.68762  C 1.56793 -5.06948 -0.10747  F 1.19953 -6.35286 -0.17815  F 1.79925 -4.77170 1.17678  F 2.73117 -4.95327 -0.76084  F -3.24155 -2.62025 -3.96139  F -4.67076 1.76699 -3.30533 |
| --- | --- |
| **III**  Ir -0.82381 -0.10105 -0.00505  C 2.10885 0.14332 0.70013  C 3.30396 0.10849 1.48079  C 3.28312 -0.19867 2.81824  C 2.01367 -0.48295 3.41008  C 0.88774 -0.44658 2.62154  C 2.07097 0.46551 -0.68773  C 3.21505 0.79253 -1.46931  C 3.11983 1.09789 -2.80859  C 1.82554 1.07320 -3.39715  C 0.74205 0.75354 -2.60517  H 4.24382 0.33245 0.99292  H 1.91832 -0.72852 4.46093  H -0.09352 -0.66510 3.03813  H 4.18759 0.79876 -0.98879  H 1.66353 1.29786 -4.44248  H -0.26316 0.72730 -3.02104  N 0.90151 -0.15626 1.30932  N 0.82785 0.46390 -1.29943  C 4.53874 -0.24701 3.68454  C 4.68594 -1.65925 4.27730  C 5.80622 0.08241 2.89146  C 4.39742 0.77221 4.82849  H 3.82133 -1.93123 4.88899  H 4.78857 -2.40357 3.48145  H 5.57751 -1.70779 4.91104  H 5.76251 1.08968 2.46518  H 6.67423 0.03573 3.55554  H 5.96633 -0.63199 2.07775  H 5.28720 0.74645 5.46604  H 4.28802 1.78585 4.43047  H 3.52723 0.55531 5.45414  C 4.38000 1.43969 -3.60967  C 5.08053 2.65010 -2.97042  C 5.33157 0.23148 -3.59408  C 4.05609 1.78333 -5.06728  H 4.42074 3.52333 -2.96910  H 5.37589 2.44501 -1.93773  H 5.98329 2.90247 -3.53653  H 4.85215 -0.64390 -4.04342  H 6.23726 0.45922 -4.16597  H 5.63006 -0.02932 -2.57483  H 4.98136 2.02819 -5.59715  H 3.58611 0.94136 -5.58483  H 3.38945 2.64864 -5.13683  C -2.71261 0.61784 2.06731  C -2.21539 -0.51768 1.36926  C -2.70843 -1.78102 1.71297  C -3.65517 -1.89727 2.71926  C -4.15776 -0.80993 3.42122  C -3.66750 0.43412 3.07192  C -2.15051 1.91001 1.66509  C -0.60424 2.89937 0.20537  C -0.89799 4.16410 0.68524  C -1.85453 4.30070 1.68804  C -2.48263 3.17000 2.18032  H -2.36720 -2.68192 1.21151  H -4.89772 -0.92463 4.20231  H 0.12564 2.73470 -0.58037  H -2.10866 5.28207 2.07425  H -3.22923 3.25517 2.95498  N -1.21667 1.81030 0.67909  C -0.15287 5.35362 0.15944  F -0.92306 6.44966 0.15849  F 0.27775 5.15199 -1.09355  F 0.92650 5.63310 0.90644  F -4.14922 1.49233 3.74854  F -4.11581 -3.11586 3.03893  C -2.36176 -1.35731 -2.11173  C -2.25742 -0.12931 -1.40067  C -3.12186 0.91591 -1.74311  C -4.04646 0.73628 -2.76073  C -4.17065 -0.44744 -3.47578  C -3.31489 -1.47542 -3.12763  C -1.42897 -2.41182 -1.70364  C 0.31723 -2.88559 -0.21103  C 0.44398 -4.17257 -0.70324  C -0.39851 -4.58913 -1.73145  C -1.33764 -3.70611 -2.23400  H -3.08876 1.87280 -1.23060  H -4.90071 -0.56666 -4.26551  H 0.94156 -2.50942 0.59254  H -0.32289 -5.59543 -2.12999  H -2.00111 -4.00945 -3.02913  N -0.59532 -2.03684 -0.69551  C 1.50528 -5.08407 -0.16570  F 1.06108 -6.34481 -0.06950  F 1.92017 -4.70100 1.04958  F 2.58449 -5.11074 -0.96297  F -3.43033 -2.62550 -3.81627  F -4.86723 1.74869 -3.07843 | **S1**  Ir -0.86188 0.08173 0.01527  C 2.19501 -0.14353 0.73848  C 3.38726 -0.00960 1.44813  C 3.36759 0.20747 2.82592  C 2.10914 0.28366 3.43525  C 0.96357 0.16055 2.66941  C 2.15454 -0.38385 -0.72869  C 3.28030 -0.72389 -1.46679  C 3.19618 -0.92392 -2.84954  C 1.93722 -0.76976 -3.43020  C 0.84655 -0.44667 -2.63356  H 4.32970 -0.05905 0.91985  H 2.00810 0.44665 4.50240  H -0.02561 0.22726 3.11316  H 4.23066 -0.85097 -0.96304  H 1.77901 -0.90218 -4.49264  H -0.14382 -0.33073 -3.06349  N 1.00146 -0.04992 1.34702  N 0.94549 -0.25584 -1.31482  C 4.63547 0.37050 3.65769  C 4.65739 -0.71410 4.74862  C 5.90238 0.24045 2.80785  C 4.61742 1.76275 4.31266  H 3.79662 -0.63579 5.41840  H 4.65866 -1.71399 4.30452  H 5.56389 -0.60446 5.35130  H 5.95155 1.01141 2.03247  H 6.77808 0.36061 3.45132  H 5.97071 -0.74248 2.33122  H 5.52144 1.89433 4.91478  H 4.59172 2.54977 3.55297  H 3.75253 1.89028 4.96948  C 4.44370 -1.30028 -3.64266  C 5.50398 -0.20046 -3.45633  C 4.98493 -2.63777 -3.10748  C 4.14287 -1.44971 -5.13649  H 5.13450 0.76273 -3.82008  H 5.79164 -0.08520 -2.40752  H 6.40218 -0.46167 -4.02342  H 4.24163 -3.43219 -3.22187  H 5.87980 -2.91867 -3.67051  H 5.25933 -2.57267 -2.05081  H 5.06343 -1.71423 -5.66330  H 3.41163 -2.24203 -5.32381  H 3.76674 -0.51652 -5.56712  C -2.34373 1.79627 1.75191  C -2.11094 0.38574 1.47280  C -2.82914 -0.59990 2.18259  C -3.75487 -0.20807 3.11993  C -4.04343 1.14584 3.38221  C -3.35117 2.10681 2.68635  C -1.53932 2.72615 1.02953  C 0.22339 2.84506 -0.55227  C 0.25476 4.21771 -0.46739  C -0.65991 4.88628 0.38720  C -1.54446 4.13881 1.12338  H -2.67371 -1.65796 2.00134  H -4.79780 1.42392 4.10835  H 0.89299 2.30570 -1.21431  H -0.65726 5.96832 0.45467  H -2.25036 4.61861 1.78626  N -0.64534 2.10595 0.15439  C 1.23357 5.00613 -1.26886  F 0.61805 5.91993 -2.04000  F 1.97192 4.23146 -2.07758  F 2.08587 5.69066 -0.48504  F -3.65617 3.39347 2.92132  F -4.42652 -1.12860 3.82304  C -2.77041 -1.15692 -1.73107  C -2.21807 0.12094 -1.41173  C -2.68072 1.26684 -2.07295  C -3.67911 1.13343 -3.02219  C -4.25582 -0.09131 -3.34652  C -3.78838 -1.21256 -2.68705  C -2.20108 -2.30379 -1.03029  C -0.55447 -2.90159 0.53516  C -0.85451 -4.24342 0.39502  C -1.87252 -4.62399 -0.48180  C -2.54550 -3.65208 -1.19920  H -2.28129 2.25259 -1.85872  H -5.04130 -0.17014 -4.08736  H 0.21273 -2.55445 1.21953  H -2.13423 -5.67052 -0.59623  H -3.33340 -3.92562 -1.88465  N -1.21809 -1.96163 -0.14753  C -0.07468 -5.27370 1.15599  F -0.86935 -6.26310 1.58370  F 0.53355 -4.74174 2.22424  F 0.87558 -5.83584 0.39497  F -4.35374 -2.38730 -2.99736  F -4.11919 2.22139 -3.66178 |
| **2**  C -2.86526 0.88506 -0.50876  O -2.35649 0.61326 -1.58489  C -3.50552 2.22647 -0.29343  C -3.50258 3.14119 -1.35146  C -4.05658 2.59774 0.93669  C -4.05013 4.40780 -1.18893  H -3.06320 2.83579 -2.29574  C -4.59875 3.86973 1.10162  H -4.07394 1.87725 1.74687  C -4.59861 4.77357 0.04095  H -4.05020 5.11181 -2.01510  H -5.02636 4.15493 2.05754  H -5.02580 5.76309 0.17128  C -2.88659 -0.15766 0.63851  O -4.02962 -0.39353 1.09146  O -1.78608 -0.65296 0.94523 | **3**  C -0.13406 -0.23045 0.04366  C 1.21565 -0.26445 -0.22524  C 1.95093 0.97248 -0.28678  C 1.25359 2.21303 -0.06509  C -0.09706 2.18588 0.19986  C -0.81268 0.97948 0.25958  H -0.67908 -1.16843 0.08784  H 1.73130 -1.20286 -0.39060  H 1.79795 3.14876 -0.10930  H -0.61330 3.12656 0.36548  H -1.87554 0.98218 0.46970  C 3.28337 0.96910 -0.55020  O 4.42945 0.96619 -0.77678  CO_2_  C 2.21336 1.11765 0.00000  O 3.37615 1.11765 0.00000  O 1.05049 1.11765 0.00000 |
| **4**  C -0.09720 2.62807 1.16126  C 0.69109 1.80004 0.77265  H -0.79331 3.36091 1.50567  C 1.62977 0.81565 0.30987  C 1.93843 -0.29489 1.10793  C 2.24268 0.95920 -0.94282  C 2.84714 -1.24516 0.65640  H 1.46204 -0.40256 2.07669  C 3.15029 0.00429 -1.38681  H 2.00117 1.81992 -1.55749  C 3.45416 -1.09814 -0.58972  H 3.08217 -2.10252 1.27841  H 3.62159 0.12068 -2.35712  H 4.16288 -1.84165 -0.93920  **2H_2_O**  O 2.78250 1.71294 -0.16726  H 3.42912 0.99830 -0.13432  H 1.93362 1.26577 -0.26458  O 3.25576 3.21140 -2.52104  H 3.09303 2.69632 -1.71122  H 3.22758 4.12580 -2.22222 | **TS1**  C 2.42234 -0.95687 0.14590  C 1.80333 0.07797 -0.56350  C 0.98930 -0.20365 -1.66188  C 0.78519 -1.52410 -2.04654  C 1.39681 -2.55739 -1.33662  C 2.21549 -2.27557 -0.24254  H 3.05428 -0.71190 0.99390  H 0.52152 0.61540 -2.19914  H 0.15160 -1.74952 -2.89786  H 1.23546 -3.58743 -1.63791  H 2.68820 -3.08414 0.30519  C 2.06600 1.48823 -0.16600  O 2.79753 1.86418 0.69749  C 0.22110 2.72104 -0.34789  C -0.66018 1.97309 0.05880  H 0.68688 3.62648 -0.67854  C -1.53197 0.94849 0.52764  C -2.22852 0.13327 -0.38180  C -1.68912 0.73338 1.90816  C -3.06302 -0.87417 0.08637  H -2.10657 0.29915 -1.44731  C -2.52609 -0.27645 2.36419  H -1.14821 1.36196 2.60758  C -3.21494 -1.08208 1.45725  H -3.59784 -1.50003 -0.62048  H -2.64115 -0.43717 3.43107  H -3.86821 -1.86962 1.81807 |
| **5**  C -0.17841 1.47379 -0.06729  C -0.48122 0.16811 0.33350  C -1.59763 -0.06659 1.14201  C -2.39164 0.99916 1.55615  C -2.07110 2.29997 1.17375  C -0.96339 2.53748 0.35975  H 0.68292 1.63556 -0.70707  H -1.86403 -1.07852 1.42666  H -3.26279 0.81327 2.17538  H -2.68824 3.12904 1.50492  H -0.71675 3.54979 0.05737  C 0.40058 -0.92918 -0.17221  O 1.01226 -0.80590 -1.21947  C 0.58399 -2.18296 0.62833  C 0.30489 -2.30264 1.89865  H 1.07666 -2.98405 0.07179  C 0.02287 -2.45063 3.23814  C -1.23098 -2.98110 3.66231  C 0.97114 -2.06602 4.23091  C -1.50751 -3.11320 5.01020  H -1.95592 -3.28529 2.91444  C 0.66699 -2.20918 5.57129  H 1.92616 -1.65945 3.91675  C -0.56774 -2.73075 5.97358  H -2.46444 -3.52051 5.32015  H 1.39646 -1.91130 6.31748  H -0.79490 -2.83830 7.02851 | **TS2**  C 1.15622 0.85975 -0.75498  C 2.19640 -0.07149 -0.46394  C 3.16917 0.21971 0.48210  C 3.12115 1.42435 1.18359  C 2.07623 2.32803 0.94664  C 1.11887 2.07060 -0.02204  H 0.69305 0.85986 -1.74052  H 3.92014 -0.53164 0.70814  H 3.87164 1.64752 1.93398  H 2.03696 3.25772 1.50560  H 0.36069 2.81267 -0.25036  C 1.96118 -1.47602 -0.88827  O 2.82504 -2.31496 -1.08054  C 0.49708 -1.73587 -0.91507  C -0.27563 -0.71568 -0.55876  H 0.12888 -2.71690 -1.21075  C -1.64554 -0.38807 -0.30791  C -2.39399 -1.15971 0.60252  C -2.24797 0.73017 -0.91501  C -3.71446 -0.82847 0.87667  H -1.92645 -2.01251 1.08402  C -3.57110 1.04601 -0.63936  H -1.67611 1.33761 -1.60982  C -4.30823 0.27151 0.25697  H -4.28373 -1.43063 1.57733  H -4.02863 1.90382 -1.12104  H -5.33960 0.52729 0.47542 |
| **6**  C -1.19878 -0.36105 1.36322  C -0.70509 -0.21078 -0.04007  C -0.91043 0.93020 -0.77899  C -1.47214 2.05282 -0.16091  C -1.72257 2.05051 1.24904  C -1.53109 0.94531 2.01533  H -2.16921 -0.90259 1.33390  H -0.54877 0.97997 -1.80232  H -1.63666 2.96157 -0.72817  H -2.06255 2.97005 1.71488  H -1.76262 0.96452 3.07484  C 0.21045 -1.31652 -0.34789  O 0.70929 -1.58493 -1.43576  C 0.45860 -1.99319 0.94787  C -0.25082 -1.41864 1.94022  H 1.14759 -2.82238 1.06110  C -0.24827 -1.84594 3.35090  C -0.30705 -3.21055 3.66448  C -0.15006 -0.91370 4.39206  C -0.27788 -3.63245 4.98942  H -0.39211 -3.93555 2.86054  C -0.11255 -1.33903 5.71657  H -0.06477 0.14373 4.16460  C -0.18205 -2.69745 6.01854  H -0.33194 -4.69170 5.21852  H -0.02544 -0.60792 6.51367  H -0.15989 -3.02648 7.05234 | **TS3**  C 1.49502 -0.06210 -0.22459  C 2.57604 -0.92998 0.22173  C 3.90803 -0.56829 0.03314  C 4.19621 0.68464 -0.48472  C 3.15821 1.61220 -0.78977  C 1.83832 1.29099 -0.61441  H 1.34397 -0.51004 -1.42129  H 4.70038 -1.24917 0.33084  H 5.22864 0.98377 -0.63285  H 3.42508 2.59710 -1.15835  H 1.05809 1.99999 -0.87357  C 1.99942 -2.10769 0.86107  O 2.61661 -3.09806 1.29803  C 0.54793 -1.87237 0.87199  C 0.24427 -0.66498 0.30163  H -0.17096 -2.55613 1.31257  C -1.10150 -0.10265 0.09992  C -2.15303 -0.91886 -0.33924  C -1.35695 1.25149 0.35503  C -3.42835 -0.39204 -0.51740  H -1.95403 -1.96252 -0.56656  C -2.63411 1.77538 0.17974  H -0.55750 1.88750 0.72278  C -3.67221 0.95584 -0.25913  H -4.23180 -1.03312 -0.86589  H -2.82009 2.82380 0.38963  H -4.66686 1.36579 -0.40130  O 1.22109 -1.11251 -2.67653  H 0.74974 -1.97322 -2.49585  H 0.62151 -0.57894 -3.21624  O -0.04743 -3.27743 -1.88130  H 0.26659 -4.13430 -2.19625  H 0.16666 -3.24754 -0.93674 |
| **116**  C -0.69609 0.56346 -0.24257  C -1.89572 -0.13611 -0.55516  C -3.14515 0.36761 -0.19259  C -3.21055 1.57333 0.50310  C -2.02553 2.26272 0.83639  C -0.77676 1.76895 0.47722  H -2.42706 -0.08545 1.99250  H -4.04603 -0.18690 -0.44139  H -4.17094 1.98405 0.79698  H -2.09109 3.19229 1.39311  H 0.11906 2.30596 0.77342  C -1.54740 -1.41707 -1.20185  O -2.35567 -2.27287 -1.62957  C -0.09521 -1.45317 -1.19592  C 0.42393 -0.25104 -0.67971  H 0.48974 -2.22904 -1.67751  C 1.84230 0.08194 -0.55281  C 2.79709 -0.92937 -0.34439  C 2.29893 1.40647 -0.66311  C 4.14976 -0.62690 -0.24360  H 2.46451 -1.95888 -0.24276  C 3.65304 1.70732 -0.55759  H 1.58697 2.19917 -0.87013  C 4.58605 0.69392 -0.34514  H 4.86744 -1.42452 -0.07750  H 3.98162 2.73760 -0.65396  H 5.64210 0.92926 -0.26289  O -1.75736 -0.41439 2.62216  H -0.98464 -1.31345 2.05803  H -1.25192 0.36924 2.88553  O -0.31416 -2.07388 1.60419  H -0.65304 -2.97246 1.73935  H -0.17914 -1.91690 0.61034 | **TS4**  C -0.72019 0.62648 -0.25831  C -1.92372 -0.06326 -0.55970  C -3.17205 0.45439 -0.22239  C -3.23169 1.67499 0.44522  C -2.04292 2.35986 0.77101  C -0.79582 1.85313 0.43306  H -2.32776 -0.09178 2.00169  H -4.07282 -0.10276 -0.46441  H -4.18960 2.09957 0.72618  H -2.10579 3.29995 1.31006  H 0.10055 2.38824 0.72908  C -1.59076 -1.37702 -1.15855  O -2.40413 -2.20881 -1.58638  C -0.13469 -1.48116 -1.04840  C 0.39332 -0.20786 -0.64458  H 0.43540 -2.21352 -1.61192  C 1.80643 0.11396 -0.52827  C 2.75211 -0.90675 -0.31133  C 2.27706 1.43404 -0.65825  C 4.10664 -0.61575 -0.21272  H 2.40839 -1.93103 -0.19807  C 3.63360 1.72055 -0.55942  H 1.57490 2.23096 -0.88029  C 4.55583 0.69965 -0.33254  H 4.81662 -1.41781 -0.03635  H 3.97416 2.74531 -0.67096  H 5.61422 0.92546 -0.25451  O -1.64762 -0.42819 2.60868  H -0.84945 -1.50593 1.96417  H -1.09282 0.34021 2.80162  O -0.27830 -2.22925 1.49190  H -0.68741 -3.09687 1.62789  H -0.20622 -1.98990 0.36261 |
| **7**  C -0.09140 -0.57204 -0.19866  C 0.96487 0.31139 0.21042  C -1.51130 -0.32937 -0.22186  C 0.94480 1.56281 0.86470  C 2.22464 -0.27059 -0.07397  C 0.52025 -1.86612 -0.67147  H 0.17950 -2.16929 -1.66748  C -2.40855 -1.41908 -0.26594  C -2.05750 0.97272 -0.24568  C 2.14824 2.18732 1.15568  H 0.01446 2.02304 1.17431  C 3.42681 0.36023 0.21636  C 2.02576 -1.60821 -0.66209  H 0.30952 -2.70463 0.00608  C -3.78068 -1.21593 -0.29036  H -2.01872 -2.43205 -0.26100  C -3.43102 1.16873 -0.27534  H -1.39830 1.83115 -0.29504  C 3.38631 1.60829 0.82766  H 2.13357 3.14735 1.66214  H 4.36676 -0.12707 -0.02474  O 2.88662 -2.37423 -1.04811  C -4.30158 0.07865 -0.28822  H -4.44884 -2.07098 -0.31025  H -3.82620 2.17906 -0.30332  H 4.30494 2.13083 1.07090  H -5.37473 0.23626 -0.30903 | **8**  C 0.11511 0.43705 0.13372  C -0.93274 -0.47986 -0.05266  C 1.53334 0.23494 0.17148  C -0.98546 -1.91109 -0.17510  C -2.21159 0.18694 -0.07018  C -0.50418 1.80727 0.31260  C 2.18165 -0.95979 -0.24563  C 2.39860 1.27544 0.61025  C -2.19585 -2.54788 -0.33987  H -0.08912 -2.51472 -0.10112  C -3.42425 -0.48297 -0.24614  C -2.00999 1.60680 0.12989  H -0.34296 2.23904 1.31231  C 3.56067 -1.10743 -0.18592  H 1.59462 -1.76656 -0.66646  C 3.77646 1.11934 0.66350  H 1.96610 2.22115 0.92414  C -3.43371 -1.85787 -0.39456  H -2.20114 -3.63284 -0.42219  H -4.34626 0.09512 -0.25505  O -2.85644 2.50275 0.16521  C 4.38246 -0.07764 0.27628  H 4.00370 -2.04229 -0.52120  H 4.38890 1.94628 1.01503  H -4.35934 -2.40584 -0.53187  H 5.45950 -0.19946 0.32138  H -0.14420 2.56192 -0.40113 |
| **TS4**′  C 0.66527 -0.52790 -0.33942  C 1.81331 0.14225 -0.80579  C 3.05152 -0.49266 -0.86636  C 3.13898 -1.82448 -0.45833  C 1.99958 -2.49537 0.00977  C 0.76262 -1.85454 0.08529  H 2.79927 -0.39490 1.53625  H 3.92432 0.04839 -1.22017  H 4.09039 -2.34446 -0.49869  H 2.08535 -3.52896 0.32990  H -0.10223 -2.38055 0.47848  C 1.45418 1.54898 -1.12335  O 2.22756 2.42354 -1.56476  C 0.05587 1.66901 -0.81166  C -0.44469 0.45549 -0.24042  H -0.52285 2.57422 -0.95478  C -1.88947 0.10359 -0.19543  C -2.80829 1.00452 0.36014  C -2.37053 -1.10999 -0.70130  C -4.16578 0.70612 0.40297  H -2.44907 1.94728 0.76664  C -3.72943 -1.41322 -0.64993  H -1.67787 -1.81221 -1.15506  C -4.63271 -0.50835 -0.09842  H -4.85998 1.41927 0.83651  H -4.08224 -2.35839 -1.05111  H -5.69083 -0.74587 -0.05934  O 2.44339 -0.25552 2.42879  H 1.09049 0.49258 2.37280  H 2.32454 -1.14295 2.79106  O 0.18384 0.94255 2.26753  H 0.28779 1.88349 2.47020  H -0.15771 0.79582 1.06505 | **7**′  C -0.97347 -0.46423 -0.38202  C -1.94646 0.42335 0.07633  C -3.05855 -0.01413 0.78975  C -3.17900 -1.37712 1.04128  C -2.20123 -2.27114 0.58781  C -1.08822 -1.82472 -0.12464  H 0.15301 -0.04083 -2.17989  H -3.80174 0.69689 1.13758  H -4.03269 -1.75394 1.59486  H -2.31196 -3.33041 0.79689  H -0.32976 -2.52298 -0.46668  C -1.57466 1.81210 -0.31400  O -2.20898 2.83981 -0.07989  C -0.31632 1.70085 -1.02742  C 0.13276 0.27297 -1.12678  H 0.22720 2.54271 -1.43628  C 1.51469 0.04386 -0.53613  C 2.54157 -0.50280 -1.30425  C 1.76391 0.38078 0.79813  C 3.80307 -0.71235 -0.74821  H 2.35363 -0.76631 -2.34149  C 3.02173 0.17416 1.35325  H 0.96315 0.80234 1.40177  C 4.04563 -0.37386 0.57982  H 4.59480 -1.14049 -1.35456  H 3.20517 0.43965 2.38947  H 5.02746 -0.53523 1.01291 |
| **TS2**′′  C -0.59269 0.29814 -0.44298  C 0.15465 1.14279 -1.15565  C -2.02943 0.15596 -0.25986  C 1.63769 1.07030 -1.14316  H -0.25936 1.92608 -1.79014  H 0.20778 -0.53283 0.18865  C -2.92651 1.11981 -0.75019  C -2.53185 -0.95947 0.42320  C 2.27839 0.00170 -0.31041  O 2.31111 1.85362 -1.79275  C 1.50066 -0.88004 0.42024  C -4.29296 0.95981 -0.56529  H -2.54804 1.99511 -1.26901  C -3.90173 -1.11682 0.60505  H -1.84009 -1.70355 0.80774  C 3.67014 -0.12509 -0.25870  C 2.02918 -1.88843 1.20288  C -4.78372 -0.15832 0.11104  H -4.97894 1.70951 -0.94546  H -4.28007 -1.98559 1.13327  C 4.23457 -1.13014 0.52023  H 4.28403 0.56494 -0.82944  C 3.42274 -2.00577 1.24506  H 1.39717 -2.56739 1.76575  H -5.85254 -0.27865 0.25472  H 5.31318 -1.23634 0.56610  H 3.87600 -2.78645 1.84847 | **6**′′  C -0.76670 0.05429 -0.20874  C 0.04964 0.90294 -0.85204  C -2.23484 0.10981 -0.19562  C 1.52839 0.88311 -0.89136  H -0.34971 1.72880 -1.43474  H -0.33884 -0.76401 0.36746  C -2.96970 1.09113 -0.87856  C -2.93029 -0.86381 0.53366  C 2.35224 -0.14876 -0.17502  O 2.10032 1.74979 -1.53878  C 1.86860 -1.18707 0.59336  C -4.35721 1.09292 -0.82951  H -2.45842 1.85764 -1.45139  C -4.32076 -0.86194 0.58276  H -2.37070 -1.62805 1.06599  C 3.75176 -0.06722 -0.28470  C 2.62559 -2.12975 1.24763  C -5.03788 0.11722 -0.09926  H -4.91216 1.85786 -1.36252  H -4.84241 -1.62371 1.15254  C 4.56873 -0.99171 0.35424  H 4.17090 0.73669 -0.88124  C 4.01426 -2.02082 1.11853  H 2.17601 -2.92218 1.83697  H -6.12236 0.12270 -0.06388  H 5.64624 -0.91315 0.25839  H 4.65867 -2.73987 1.61516 |
| **TS3′′**  C -0.46178 0.57111 0.51630  C 0.22569 1.66111 0.03210  C -1.86136 0.26847 0.17554  C 1.69516 1.66101 0.08145  H -0.26587 2.44348 -0.53781  H -0.09499 0.06587 1.40870  C -2.43059 0.70420 -1.02879  C -2.64241 -0.48055 1.06425  C 2.21733 0.26376 0.06780  O 2.40779 2.65388 0.03822  C 1.28683 -0.74812 -0.07058  C -3.75914 0.42088 -1.32169  H -1.82315 1.25328 -1.74217  C -3.97215 -0.76445 0.77049  H -2.20306 -0.83367 1.99331  C 3.57110 -0.08031 0.14921  C 1.59218 -2.09131 -0.09908  C -4.53377 -0.31086 -0.42142  H -4.18969 0.76328 -2.25700  H -4.56937 -1.34044 1.46964  C 3.92945 -1.42287 0.09738  H 4.31475 0.70459 0.25431  C 2.95206 -2.41758 -0.02605  H 0.83482 -2.86311 -0.18904  H -5.57005 -0.53445 -0.65330  H 4.97460 -1.70722 0.15617  H 3.25126 -3.46080 -0.06824  **TS3-3H_2_O**  C 1.61060 -0.03601 -0.14894  C 2.73505 -0.74531 0.44629  C 4.04282 -0.31133 0.24466  C 4.25860 0.87191 -0.44477  C 3.16789 1.65816 -0.91833  C 1.86911 1.26210 -0.73693  H 1.51673 -0.73051 -1.22223  H 4.87110 -0.87784 0.66084  H 5.27100 1.22740 -0.60578  H 3.37647 2.59458 -1.42529  H 1.04903 1.86157 -1.12006  C 2.22156 -1.87228 1.21772  O 2.89307 -2.74452 1.80243  C 0.75875 -1.75089 1.15641  C 0.38882 -0.64970 0.43163  H 0.07709 -2.42674 1.66138  C -0.98755 -0.21144 0.14634  C -1.97945 -1.15312 -0.15825  C -1.33677 1.14483 0.19967  C -3.28759 -0.74952 -0.40220  H -1.70364 -2.20085 -0.23254  C -2.64731 1.54684 -0.03995  H -0.58400 1.88204 0.46227  C -3.62543 0.60160 -0.34325  H -4.04317 -1.48929 -0.64707  H -2.90601 2.59942 0.01501  H -4.64600 0.91680 -0.53500  O 1.47710 -1.70287 -2.22263  H 0.57346 -1.81451 -2.65150  H 2.11958 -1.50405 -2.91624  O -0.90538 -2.29824 -3.04323  H -0.91459 -2.72067 -3.91081  H -0.89325 -3.02941 -2.39161  O -0.05225 -3.95839 -1.08065  H 0.58582 -3.32895 -0.71131  H -0.47188 -4.37289 -0.31788  **TS4-3H_2_O**  C 0.74104 -0.65452 -0.52287  C 1.90436 0.06016 -0.91143  C 3.17502 -0.50182 -0.83392  C 3.30385 -1.80205 -0.35258  C 2.16243 -2.51174 0.07187  C 0.89170 -1.95074 0.01085  H 2.74570 0.39400 1.73906  H 4.04142 0.07411 -1.14768  H 4.28049 -2.26992 -0.28861  H 2.28010 -3.51653 0.46617  H 0.03947 -2.51063 0.38095  C 1.50622 1.41072 -1.38081  O 2.27143 2.26869 -1.84035  C 0.06511 1.49446 -1.13406  C -0.40801 0.19534 -0.72740  H -0.55933 2.19467 -1.68439  C -1.80196 -0.14978 -0.50597  C -2.73440 0.84954 -0.16482  C -2.27625 -1.46734 -0.65412  C -4.07325 0.54153 0.03875  H -2.39235 1.87270 -0.04027  C -3.61697 -1.77148 -0.44984  H -1.59495 -2.24798 -0.97514  C -4.52292 -0.77209 -0.09692  H -4.76961 1.32911 0.30972  H -3.95931 -2.79363 -0.57836  H -5.56873 -1.01209 0.06396  O 2.36113 -0.21714 2.38211  H 0.79113 0.22984 2.62539  H 2.35481 -1.06561 1.91317  O -0.12315 0.62444 2.66156  H -0.32062 0.78290 3.59330  H -0.01238 1.81626 1.91701  O 0.17460 2.61838 1.24912  H 0.13178 2.16851 0.17916  H -0.49164 3.31228 1.35586 | **(H_2_O)_3_**  O -0.87411 1.33592 1.88252  H -1.85025 1.39187 1.86314  H -0.62083 1.71961 2.72968  O -3.46190 2.08361 1.23917  H -3.83207 2.67895 1.90065  H -2.92637 2.65810 0.65640  O -1.31730 3.29984 -0.01770  H -0.87613 2.66839 0.58475  H -1.17505 2.93972 -0.90042  **TS3-H_2_O**  C -0.87179 0.39426 -0.13887  C -2.08686 -0.37314 -0.32457  C -3.33523 0.24685 -0.28789  C -3.39943 1.62402 -0.14249  C -2.21740 2.40580 -0.04798  C -0.97031 1.82611 -0.08654  H -0.80771 0.24863 1.21806  H -4.23715 -0.34930 -0.39495  H -4.36378 2.12075 -0.11112  H -2.30192 3.48276 0.05299  H -0.07791 2.43573 0.01431  C -1.72770 -1.77758 -0.50384  O -2.52050 -2.73713 -0.63353  C -0.26747 -1.80870 -0.48501  C 0.25223 -0.54628 -0.32934  H 0.31543 -2.71567 -0.59716  C 1.67839 -0.19626 -0.23597  C 2.57608 -1.05995 0.40894  C 2.17741 0.98462 -0.80395  C 3.92970 -0.75187 0.48518  H 2.19786 -1.97107 0.86365  C 3.53257 1.29298 -0.72577  H 1.50435 1.64769 -1.33870  C 4.41287 0.42801 -0.07931  H 4.60878 -1.43016 0.99217  H 3.90255 2.20848 -1.17646  H 5.46870 0.67064 -0.01654  O -0.75656 0.02422 2.47891  H -0.45443 -0.88414 2.63601  H -1.63673 0.10566 2.87905  **116-3H_2_O**  C 1.38756 -0.10131 -0.05660  C 2.49807 -0.86769 0.40222  C 3.80532 -0.53862 0.04536  C 4.02469 0.55972 -0.78347  C 2.93216 1.30764 -1.26686  C 1.62100 0.97839 -0.92407  H 2.94017 -1.81571 -2.03732  H 4.63318 -1.13682 0.41736  H 5.03399 0.83909 -1.06773  H 3.11415 2.15420 -1.92211  H 0.80005 1.55770 -1.33499  C 1.99892 -1.96036 1.26235  O 2.70495 -2.81290 1.85260  C 0.55854 -1.80374 1.26460  C 0.17991 -0.69253 0.49560  H -0.10987 -2.38396 1.89365  C -1.18932 -0.22378 0.28420  C -2.26208 -1.13315 0.25871  C -1.48719 1.14185 0.13022  C -3.56996 -0.69913 0.07676  H -2.05872 -2.19430 0.37046  C -2.79579 1.57447 -0.05765  H -0.68764 1.87215 0.19890  C -3.84528 0.65781 -0.08936  H -4.37772 -1.42449 0.05677  H -2.99846 2.63547 -0.16765  H -4.86578 0.99592 -0.23652  O 2.54532 -1.40837 -2.82502  H 1.15998 -1.64007 -2.82161  H 2.70111 -0.45729 -2.68609  O 0.12173 -1.85319 -2.74562  H -0.22634 -2.12475 -3.60748  H 0.00301 -2.64005 -2.02382  O 0.06785 -3.58986 -1.03392  H 0.27482 -3.10971 -0.19599  H -0.73622 -4.10388 -0.88292 |
